# A mild and scalable one-pot synthesis of *N*-substituted 2-aminobenzimidazoles *via* visible light mediated cyclodesulfurization

**DOI:** 10.1038/s41598-025-86772-8

**Published:** 2025-02-03

**Authors:** Tanawat Rerkrachaneekorn, Rose Malina Annuur, Soraya Pornsuwan, Mongkol Sukwattanasinitt, Sumrit Wacharasindhu

**Affiliations:** 1 Nanotec-CU Center of Excellence on Food and Agriculture, Department of Chemistry, Faculty of Science, Chulalongkorn University, Bangkok, 10330 Thailand; 2 Department of Chemistry, Center of Excellence for Innovation in Chemistry, Faculty of Science, Mahidol University, Bangkok, 10400 Thailand; 3 Green Chemistry for Fine Chemical Productions and Environmental Remediation Research Unit, Department of Chemistry, Faculty of Science, Chulalongkorn University, Bangkok, 10330 Thailand

**Keywords:** Visible light mediated, *N*-substituted 2-aminobenzimidazoles, Photocatalyst-free, Thiourea, Cyclodesulfurization, Green chemistry, Organic chemistry, Photochemistry, Chemical synthesis

## Abstract

A visible light mediated photocatalyst-free synthesis of *N*-substituted 2-aminobenzimidazoles directly from *o*-phenylenediamines and isothiocyanates is developed in a one-pot fashion. This one-pot reaction proceeds through three steps: *N*-substitution of *o*-phenylenediamines, thiourea formation and visible light mediated cyclodesulfurization. This method enables the rapid and efficient synthesis of structurally diverse *N*-substituted 2-aminobenzimidazoles, achieving yields up to 92% across 69 examples. The practicality of the reaction is demonstrated by gram-scale synthesis. The key advantages of this method include the use of less toxic solvent in aqueous media, the elimination of photocatalyst, and a simple, practical setup (one-pot, open-flask, and ambient temperature). Mechanistic insights are gathered through control experiments, including light on/off cycles and radical inhibition studies. The results indicate that the reaction involves with radical pathway mediated by visible light.

**Supplementary Information:**

The online version contains supplementary material available at 10.1038/s41598-025-86772-8.

## Introduction

**Fig. 1:**
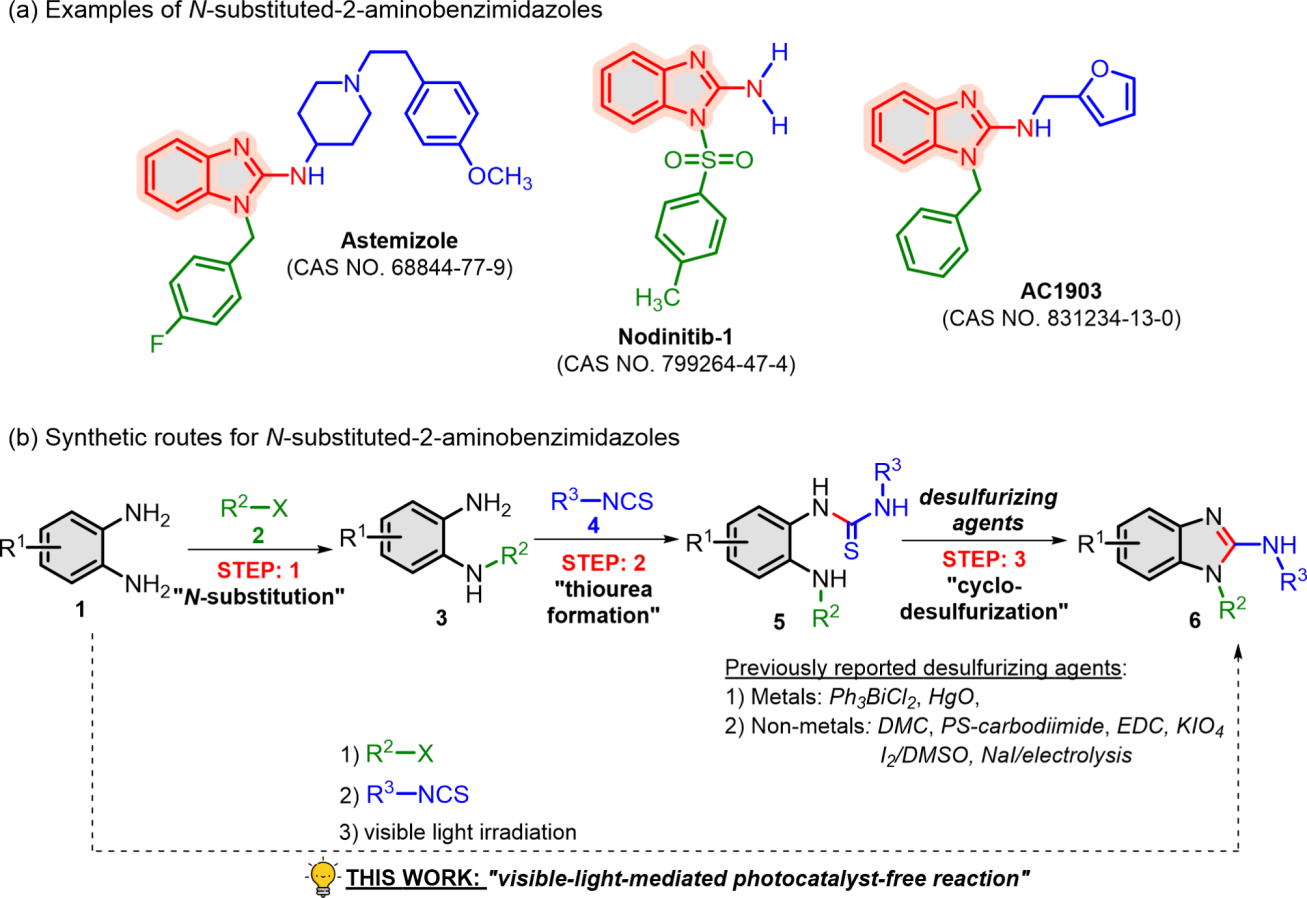
Examples and synthetic routes of *N*-substituted 2-aminobenzimidazoles.

*N*-substituted 2-aminobenzimidazoles are heterocyclic compounds recognized as crucial motifs in various therapeutic agents^1–6^. Notable examples include Astemizole, the second generation of antihistamine^3^, Nodinitib-1, a selective NOD-1 inhibitor^4^ and AC1903, a selective TRPC5 inhibitor^5,6^ (Fig. 1a). Traditionally, various synthetic routes have been developed for *N*-substituted 2-aminobenzimidazoles^7,8^; however, the most practical method utilized the reaction between *o*-phenylenediamines (**1**) or *N*-substituted *o*-phenylenediamines (**3**) and isothiocyanates (**4**), due to the wide availability of these starting materials^9–17^. This reaction typically proceeds through three steps: *N*-substitution of **1** with **2** into **3**, thiourea formation with isothiocyanates **4** and cyclodesulfurization of thioureas **5** to yield *N*-substituted 2-aminobenzimidazole products **6** (Fig. 1b). While the initial two steps can occur under mild conditions, cyclodesulfurization usually requires desulfurizing agents, including Ph_3_BiCl_2_^9^, HgO^10^, DMC^11^, PS-carbodiimide^12^, EDC^13^, KIO_4_^14^, I_2_/DMSO^15,16^ and NaI/electrolysis^17^. Despite yielding *N*-substituted 2-aminobenzimidazoles **6** efficiently, these methods pose significant drawbacks, including: (1) harsh reaction conditions, (2) use of toxic metals, halogens or high-molecular weight desulfurizing agents, (3) the need of organic solvent and 4) requiring work up and separation during multiple-step synthesis from *o*-phenylenediamines **1**. Consequently, the development of a one-pot, environmentally-friendly synthesis for *N*-substituted 2-aminobenzimidazole **6** remains an important and challenging goal.

Over the past decade, photochemical reactions mediated by visible light have emerged as an alternative method for organic synthesis^18,19.^ These reactions typically harness visible light in conjunction with transition-metal or organic-dye photocatalysts. Photoredox catalysis, especially in the oxidation of organosulfurs^20–28^ and cyclodesulfurization^29–36^, has enabled the formation of diverse heterocycles and related compounds. While photoredox methods are generally mild, they still rely on photocatalysts. Recently, catalyst-free photoreactions have gained attention in organic synthesis^37,38^ for providing milder, greener conditions. These photocatalyst-free reactions have also been applied to organosulfur oxidations yielding various organic product^39–50^.

However, the synthesis of *N*-substituted 2-aminobenzimidazoles *via* catalyst-free photoreaction has not been reported yet. In this work, we present a three-step one-pot synthesis approach (Fig. 1b): first, *o*-phenylenediamines (**1**) undergo *N*-substitution with protecting agents (**2**) to generate *N*-substituted *o*-phenylenediamines (**3**) in situ (Step: 1). Next, the addition of isothiocyanates (**4**) forms thioureas (**5**) (Step: 2), followed by visible light mediated photocatalyst-free cyclodesulfurization to yield *N*-substituted 2-aminobenzimidazoles (**6**) (Step: 3).

## Results and discussion

### Prove the concept of visible light mediated photocatalyst-free cyclodesulfurization

Even though we aimed to perform (1) *N*-substitution, (2) thiourea formation and (3) cyclodesulfurization *via* visible light mediated photocatalyst-free in one-pot reaction, the last two steps which were thiourea formation followed by cyclodesulfurization of thiourea intermediates (**5**) should be proven prior. Therefore, we selected *N*-tosyl *o*-phenylenediamine (**3a**) as our model substrate to react with 4-chlorophenyl isothiocyanate (**4a**) providing *N*-tosyl 2-aminobenzimidazole (**6a**) as shown in Table 1. Initially, the reaction mixture was stirred in a mixture of EtOH and water (9:1) using K_2_CO_3_ as a base (Table 1, entry 1). Only 9% of the desired product **6a** was isolated and remained thiourea intermediate (**5a**) was observed by visualization on TLC. Upon increasing the reaction temperature to 90 ^o^C, we observed the decomposition of thiourea intermediate (**5a**) (Table 1, entry 2). Therefore, the cyclodesulfurization step cannot proceed at room temperature or elaborated condition. Next, various traditional photocatalysts such as Rose Bengal, Riboflavin, Ru(bpy)_3_Cl_2_ and Ir(bpy)_3_ were tested under irradiating by 19 W white LED bulb as the light source (Table 1, entry 3). The reaction setup was shown in Fig. S1. The **6a** products were isolated in excellent yields (79–86% yields). When the reaction was carried in the absence of photocatalyst, the **6a** was isolated in similar good yield (Table 1, entry 4). These results confirmed our hypothesis that cyclodesulfurization step can be mediated under visible light without the need of photocatalyst.

**Table 1:** Prove the concept of visible light mediated photocatalyst-free cyclodesulfurization.


Entry	Deviations from standard condition^a^	Yield^b^ (%)
1	None	9
2	Heat at 90 ^o^C	N.P
3	Rose Bengal or Riboflavin or Ru(bpy)_3_Cl_2_ or Ir(bpy)_3_ with irradiation^c^	79–86
4	Irradiation^c^ without photocatalyst	82

^a^Reaction condition: **3a** (2.0 eq), **4a** (1.0 eq, 0.40 mmol), K_2_CO_3_ (2.0 eq), 3 mL (9:1 EtOH: H_2_O), 24 h under air at room temperature. ^b^Isolated yields by column chromatography. ^c^Irradiation was carried under 19 W white LED bulb. N.P: no product.

## Reaction optimization

After examining the concept of visible light mediated photocatalyst-free reaction, we aimed to perform all three steps reaction including (1) *N*-substitution, (2) thiourea formation and (3) cyclodesulfurization in one-pot manner. Initially, *N*-tosyl *o*-phenylenediamine (**3a**) was prepared in situ from reaction between *o*-phenylenediamine (**1a**) and TsCl (**2a**) under basic condition followed by thiourea formation upon reacting with 4-chlorophenyl isothiocyanate (**4a**). Then, the reaction mixture was irradiated under visible light to generate product **6a** (Table 2). Tosylation reaction was performed in a mixture of EtOH and water using K_2_CO_3_ as a base under open-flask at room temperature for 1 h. TLC indicated the completed conversion of **1a** and **2a** into **3a**. Upon irradiation with 19 W white LED bulb for 24 h with **4a**, it gave the desired product **6a** in 81% yield (Table 2, entry 1). To study the effect of base, both organic bases (TEA, DBU) and inorganic bases (Na_2_CO_3_, Cs_2_CO_3_, NaOH and CH_3_COONa) were employed as shown in Table 2, entries 2 and 3 and Table S1, entries 1–4. The results revealed that our reaction preferred inorganic bases especially carbonate bases. Therefore, we selected K_2_CO_3_ for further study. The reaction time for cyclodesulfurization was carefully monitored at 3, 6 and 12 h in Table 2, entries 4 and 5 and Table S1, entry 5. We found that our reaction was completed at 12 h providing **6a** as 86% yield. Hence, irradiation time at 12 h will be used for visible light mediated cyclodesulfurization. Various organic solvents such as MeCN, MeOH, EtOH, *i*PrOH, DMSO and CH_2_Cl_2_ have been investigated (Table 2, entries 6 and 7 and Table S1, entries 6–9). Among them, alcoholic solvents were considered as promising media providing **6a** in good yields. Next, the water content in reaction media was examined (Table 2, entry 8 and Table S1, entry 10). EtOH was selected as a main solvent due to its safeness^51^. We confirmed that a 9:1 ratio of EtOH to water provided the best solubility for both the starting materials and K₂CO₃, yielding product **6a** with the highest efficiency. By changing LED bulb into various LED strips such as red, green and blue (see Fig. S2 for reaction setup) resulted in no improvement of the product yields (Table S1, entries 11–13). Recently, utilizing LED equipped with glass rod fiber was more attractive among photochemical reaction due to its high energy penetration and energy saving^52^. We then carried out the reaction using 3 W white LED equipped with glass rod fiber as the light source (see Fig. S3 for the reaction setup). After 12 h of irradiation under identical conditions, product **6a** was isolated in 82% yield, confirming that the glass rod fiber can be effectively used as a light source (Table 2, entry 9). Based on the literature on photocatalyst-free reactions, the high energy light source was often employed because most organic compounds can easily absorb this energy^40,43,46^. Therefore, we switched the light source from white LED to blue LED, also equipped with glass rod fiber. This setup allowed us to reduce the reaction time from 12 to 6 h, yielding product **6a** in 82%, while the white LED only gave a 61% yield under the same conditions (Table 2, entries 10 and 11). Based on these results, we selected the reaction conditions in Table 2, entry 11, as our optimized protocol.

**Table 2:** Reaction optimization condition.


Entry	Deviations from standard condition^a^	Yield^b^ (%)
1	None	81
2	CH_3_COONa as base	18
3	TEA as base	46
4	6 h^c^	58
5	12 h^c^	86
6	EtOH, 12 h^c^	45
7	*i*PrOH, 12 h^c^	51
8	(1:1 EtOH: H_2_O), 12 h^c^	46
9	3 W white LED (glass rod fiber), 12 h^c^	82
10	3 W white LED (glass rod fiber), 6 h^c^	61
11	3 W blue LED (glass rod fiber), 6 h^c^	82

^a^Reaction condition: (First step) **1a** (1.2 eq), **2a** (1.2 eq), base (1.2 eq), 3 mL solvent, 1 h under air. (Second step) **4a** (1.0 eq, 0.40 mmol), base (1.0 eq), 24 h under air at room temperature. ^b^Isolated yields by column chromatography. ^c^Irradiation time for second step.

## Substrate scopes

**Fig. 2:**
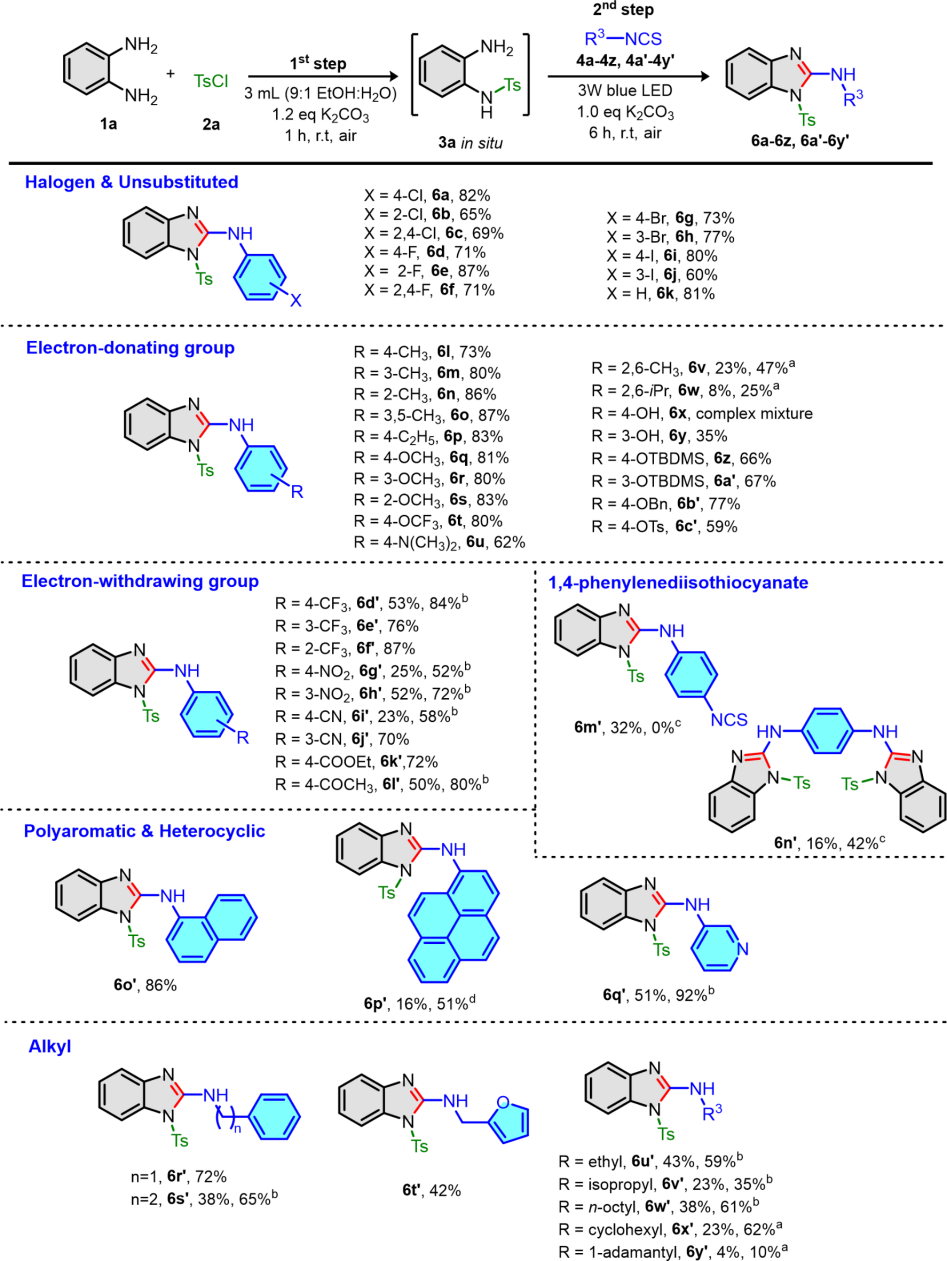
Scope of isothiocyanates. Reaction condition: (First step) **1a** (1.2 eq), **2a** (1.2 eq), K_2_CO_3_ (1.2 eq), 3 mL (9:1 EtOH: H_2_O), 1 h under air. (Second step) **4a-4z**,** 4a’-4y’** (1.0 eq, 0.40 mmol), K_2_CO_3_ (1.0 eq), 6 h under air at room temperature. ^a^Reaction was pre-stirred with isothiocyanates for 24 h. ^b^Irradiation time was 24 h. ^c^Amount of **1a**, **2a** and K_2_CO_3_ were 2.4 eq. ^d^Solvent was used as 6 mL.

With the optimized condition in our hands, we investigated the scope of the reaction using various aryl and alkyl isothiocyanates (**4a-4z** and **4a’-4y’**) as shown in Fig. 2. To begin with, **3a** was generated in situ by tosylation of **1a** followed by simultaneous thiourea formation with various aryl isothiocyanates bearing halide (**4a-4j**), unsubstituted (**4k**) and electron-donating groups (**4l-4z** and **4a’-4c’**). Then, photocatalyst-free cyclodesulfurization under visible light gave the corresponding *N*-tosyl 2-aminobenzimidazole products (**6a-6z** and **6a’-6c’**) from good to excellent yields in one-pot condition. The steric electron-donating substituent on aryl isothiocyanates (**4v** and **4w**) required longer reaction time (24 h) for thiourea formation step to improve the yields of target products **6v** and **6w**. In case of phenolic compounds such as 4-OH (**4x**) and 3-OH (**4y**) phenyl isothiocyanates, complex mixture was observed and only 35% yield of **6y** was isolated and no product for **6x**. We hypothesized that the corresponding thioureas occupying free hydroxy moiety were prone to be oxidized under our reaction condition. Therefore, the protecting groups on phenolic isothiocyanate were required. Common protecting groups such as *tert*-butyldimethylsilyl, benzyl and tosyl group (**4z** and **4a’-4c’**) were attached into phenolic isothiocyanates. The corresponding *N*-tosyl 2-aminobenzimidazoles (**6z** and **6a’-6c’**) were isolated suggesting those protecting groups were highly tolerated under reaction condition. On the other hand, electron-withdrawing group (**4d’-4l’**) gave the target product **6d’-6l’** in fair to good yields. However, the strong electron-withdrawing group, particularly CF_3_, NO_2_ and CN (**4d’**, **4g’** and **4i’**), required longer reaction to improve the yields of related *N*-tosyl 2-aminobenzimidazole products (**6d’**, **6g’** and **6i’**). We hypothesized that cyclodesulfurization should be our rate determining step. Moreover, 1,4-phenylenediisothiocyanate (**4m’**) gave the corresponding *N*-tosyl 2-aminobenzimidazole **6m’** as major product in 32% yield and bis-*N*-tosyl 2-aminobenzimidazole **6n’** as minor product in 16% yield under optimal condition. Interestingly, increasing the equivalent of **3a** from 1.2 to 2.4 eq, we were able to switch to regioselectivity of reaction providing bis-*N*-tosyl 2-aminobenzimidazole **6n’** as a sole product in 42% yield. Next, polyaromatic (**4o’**,** 4p’**) and heterocyclic (**4q’**) isothiocyanates were successfully converted to the desired products **6o’-6q’** in 16 to 92% yields. Noticeably, the low yield of **6p’** was due to the poor solubility of isothiocyanate **4p’** starting material. Therefore, when reaction was carried in lower concentration (67 mM), higher yield of product **6p’** was received in moderate yield (51%). Furthermore, less reactive alkyl isothiocyanates (**4r’-4y’**) were examined. Fortunately, we accomplished the synthesis of *N*-tosyl 2-aminobenzimidozole products **6r’-6y’** in fair to good yields. However, highly steric isothiocyanate **4y’** gave the corresponding 2-aminobenzimidazole **6y’** in poor yield and could not be improved even prolonging the reaction time for thiourea formation to 24 h.

**Fig. 3:**
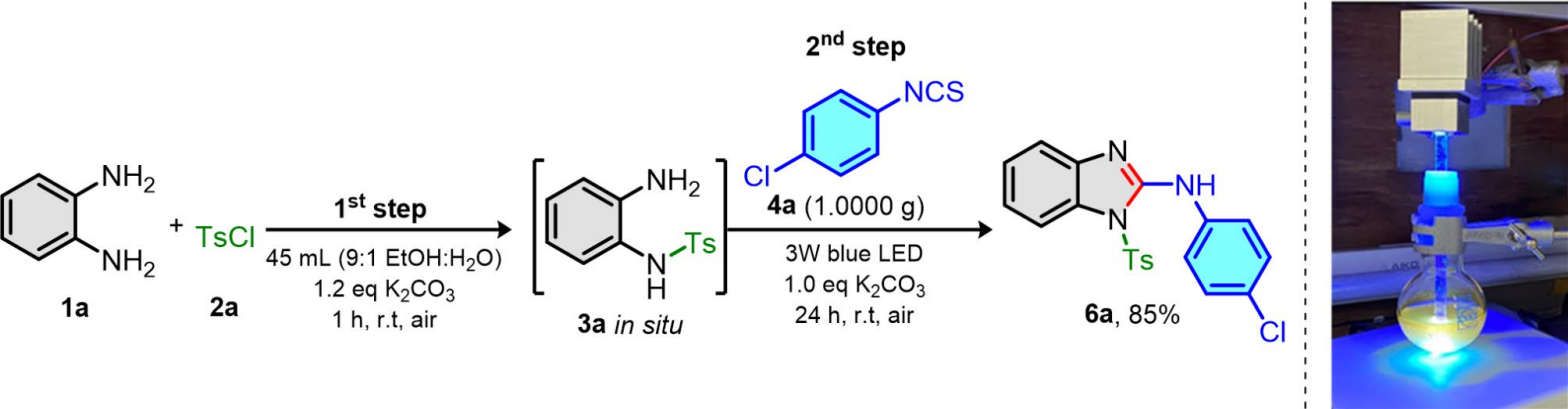
Gram-scale synthesis with reaction setup.

To demonstrate the scalability of our developed reaction, we performed our reaction using isothiocyanate **4a** in gram-scale under the optimization condition as shown in Fig. 3. Even though the scale of **4a** was increased *ca*. 15 times from optimization condition, the same 3 W blue LED equipped with glass rod fiber was able to use as light source providing the excellent yield (85%) of **6a**. From the result, this indicated that our visible light mediated photocatalyst-free cyclodesulfurization was not only scalable in industry but also very practical under the same light source setup.

**Fig. 4:**
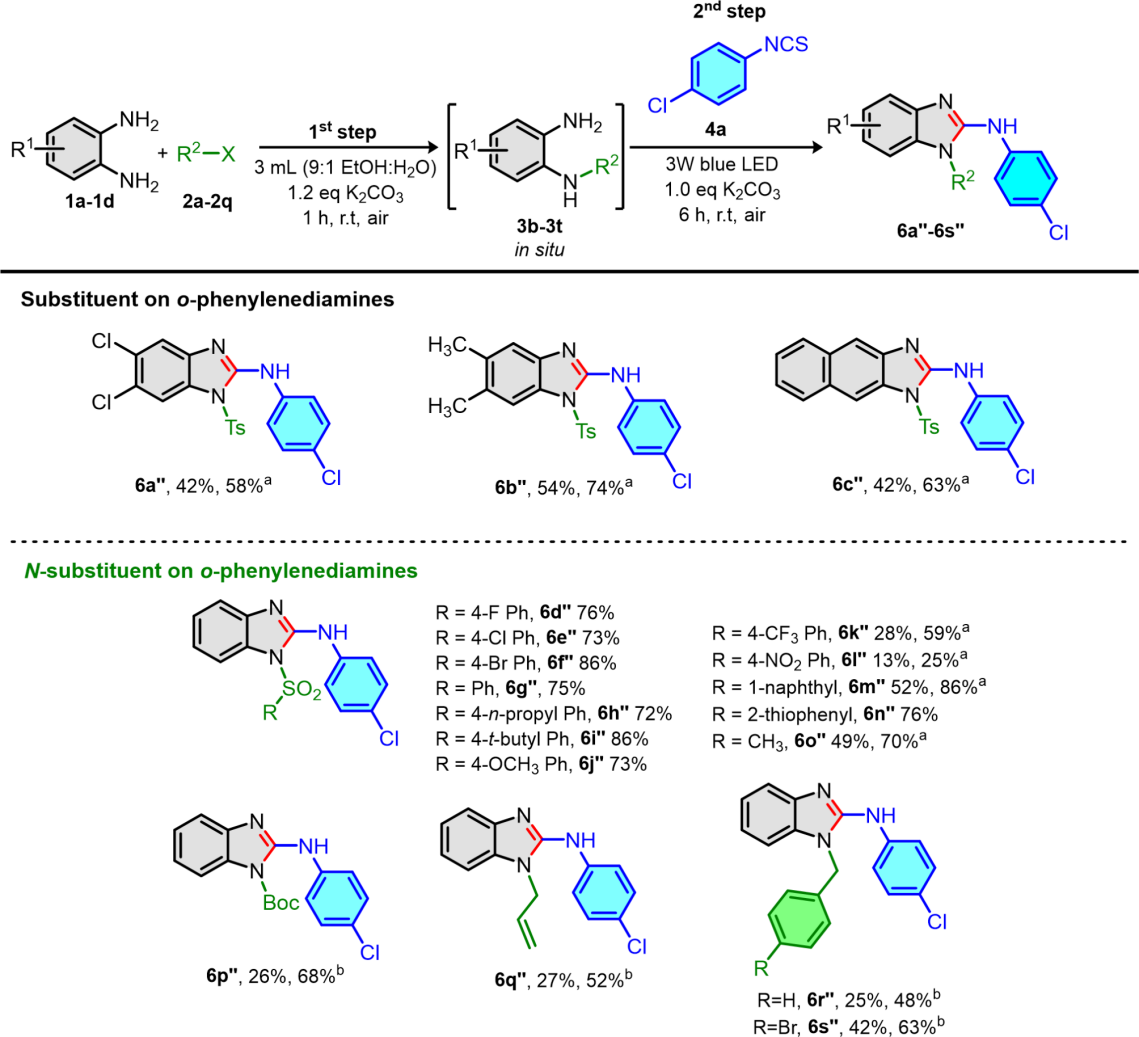
Scope of substituent/*N*-substituent on *o*-phenylenediamines. Reaction condition: (First step) **1a-1d** (1.2 eq), **2a-2q** (1.2 eq), K_2_CO_3_ (1.2 eq), 3 mL (9:1 EtOH: H_2_O), 1 h under air. (Second step) **4a** (1.0 eq, 0.40 mmol), K_2_CO_3_ (1.0 eq), 6 h under air at room temperature. ^a^Irradiation time was 24 h. ^b^First step was performed at 0 ^o^C and second step was irradiated for 24 h.

Next, we turned our attention to examine the generality of our reaction by varying substituent and *N*-substituent on *o*-phenylenediamines as depicted in Fig. 4. The *o*-phenylenediamines **1b-1d** were converted into the desired *N*-tosyl 2-aminobenzimidazoles **6a’’-6c’’** in good yields upon prolonging irradiation time to 24 h. Moreover, a variety of sulfonyl chlorides (**2b-2m**) were subjected to sulfonylation with *o*-phenylenediamine **1a** under optimization. Upon irradiation process, the reactions were smoothly proceeded to the corresponding *N*-sulfonyl 2-aminobenzimidazole products (**6d’’-6o’’**) in fair to good yields except **6k’’** and **6l’’**. We would like to note that the electron-withdrawing group on benzenesulfonyl chloride such as CF_3_ (**2i**) and NO_2_ (**2j**) could decelerate to the cyclodesulfurization step providing lower yields of product **6k’’** and **6l’’**. Therefore, increasing of reaction time improved the yields of products **6k’’** and **6l’’** (59 and 25% yields, respectively). By switching from sulfonyl chloride to (Boc)_2_O (**2n**), allyl bromide (**2o**) and benzyl bromide derivatives (**2p-2q**), the corresponding products **6p’’-6s’’** were obtained in fair yields. It was noteworthy to mention that, in case of carboxylate, allyl and benzyl substituents on *o*-phenylenediamine, performing *N*-alkylation at 0 ^o^C in an ice bath and irradiating at 24 h were required to improve the yields of the corresponding products **6p’’-6s’’** in 48 to 68% yields.

## Mechanistic studies

**Fig. 5:**
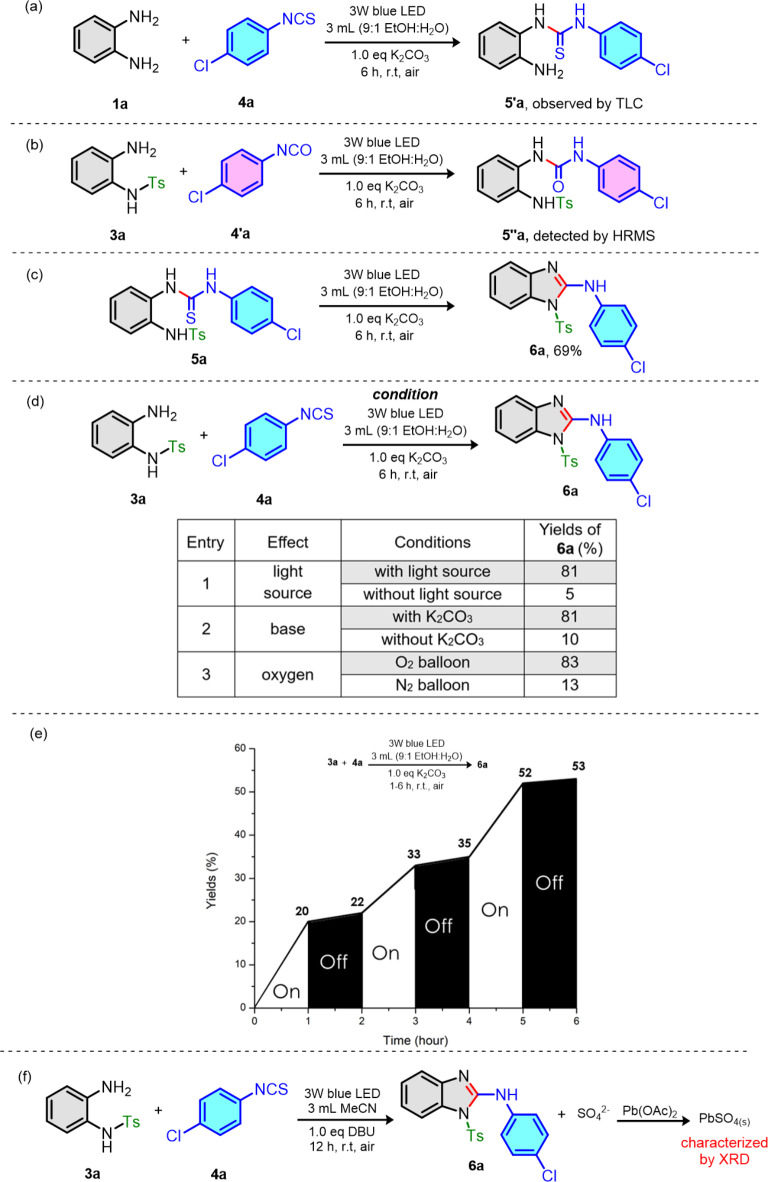
Control experiments.

To gain mechanistic information of visible light mediated photocatalyst-free cyclodesulfurization reaction, various control experiments were investigated in Fig. 5. When *o*-phenylenediamine **1a** was subjected to our visible light condition with **4a** (Fig. 5a), the target *N*-tosyl 2-aminobenzimidazole (**6a**) was not observed and only *o*-aminophenyl thiourea **5’a** was detected by TLC. Therefore, the photocatalyst-free cyclodesulfurization reaction of thiourea intermediates (**5**) required *N*-substituent group on *o*-phenylenediamine. Next, we replaced 4-chlorophenyl isothiocyanate (**4a**) with 4-chlorophenyl isocyanate (**4’a**) to study the role of sulfur atom in our reaction. **3a** was reacted with **4a’** under irradiating with 3 W blue LED (Fig. 5b). Only *o*-aminophenyl urea (**5’’a**) was detected as only product without the formation of *N*-tosyl 2-aminobenzimidazole (**6a**). This was also confirmed by HRMS of the crude reaction. Therefore, our reaction was more efficient with desulfurization than deoxygenation process. To confirm that thiourea **5a** was our intermediate, we prepared thiourea **5a** and subjected as a starting material for photocatalyst-free cyclodesulfurization (Fig. 5c). We obtained **6a** as a sole product in 69% yield. Hence, thiourea **5a** was a key intermediate. We further investigated the effect of light source, base and reaction atmosphere on our reaction as shown in Fig. 5d. We conducted the reaction in the absence of light or base comparing with standard condition as seen in Fig. 5d (entries 1 and 2). These results revealed that both light source and base were required for synthesis of **6a** product under visible light mediated cyclodesulfurization process. Moreover, we examined the role of oxygen by carrying reaction under O_2_ and N_2_ atmosphere as shown in Fig. 5d (entry 3). Comparing to our standard condition which performed reaction under open-flask, it indicated that the oxygen in air was necessary. The visible-light on/off experiment was also conducted in Fig. 5e. The yield of **6a** increased when we irradiated the light through our reaction while there was no significant improvement of **6a** among non-irradiation period. This observation could imply that our reaction was not involved with radical chain reaction process but through light-mediated process. To trap the sulfur-byproduct, we repeated the photoreaction of model substrate in acetonitrile (MeCN) and DBU as a base followed by addition of Pb(OAc)_2_. The use of acetonitrile solvent and DBU instead of EtOH: H_2_O and K_2_CO_3_ could prevent the formation of interference solid from potassium carbonate salt^31,35,36^. This resulted in the formation of white precipitation as PbSO_4_ which was confirmed by XRD (see the results in Fig. 5f and Fig. S5). Furthermore, various radical trapping agents were added to our reaction in order to detect the true oxidizing agents (Table 3). All radical trapping agents including 6-di-*tert*-butyl-4-methylphenol (BHT), 1,4-benzoquinone, 9,10-dimethylanthracene and 1,3-diphenylisobenzofuran gave much lower yields of product **6a** in comparison with optimal condition (23 to 42% yields). Although we could not identify the true oxidizing agents from above experiments, the reaction mechanism might involve the radical pathway which was also supported by EPR experiments (Fig. S6). On the other hand, we attempted to investigate the possibility of the electron donor-acceptor (EDA) complex photochemistry by UV-vis spectroscopy^37,38^. The UV**-**vis spectroscopic measurements of **3a**, **4a**, **3a** + **4a** and **5a** (thiourea intermediate) in the presence and absence of K_2_CO_3_ as shown in Fig. S7. gave no bathochromic shift or strong color development as commonly seen in EDA complex formation. Therefore, the EDA complex formation may not involve in our reaction mechanism.

**Table 3:** Radical trapping experiments. Reaction condition: (First step) **1a** (1.2 eq), **2a** (1.2 eq), K_2_CO_3_ (1.2 eq), 3 mL (9:1 EtOH: H_2_O), 1 h under air at room temperature. (Second step) **4a** (1.0 eq, 0.40 mmol), K_2_CO_3_ (1.0 eq), trapping agents (4.0 eq, 1.60 mmol), 6 h under air at room temperature.


Entry	Trapping agents	Notes	Yield^a^ (%)
0	Without trapping	–	82
1	BHT	Free radical	42
2	1,4-benzoquinone	Superoxide	23
3	9,10-dimethylanthracene	Singlet oxygen	26
4	1,3-diphenylisobenzofuran	Singlet oxygen	29

^a^Isolated yields by silica gel column chromatography.

**Fig. 6:**
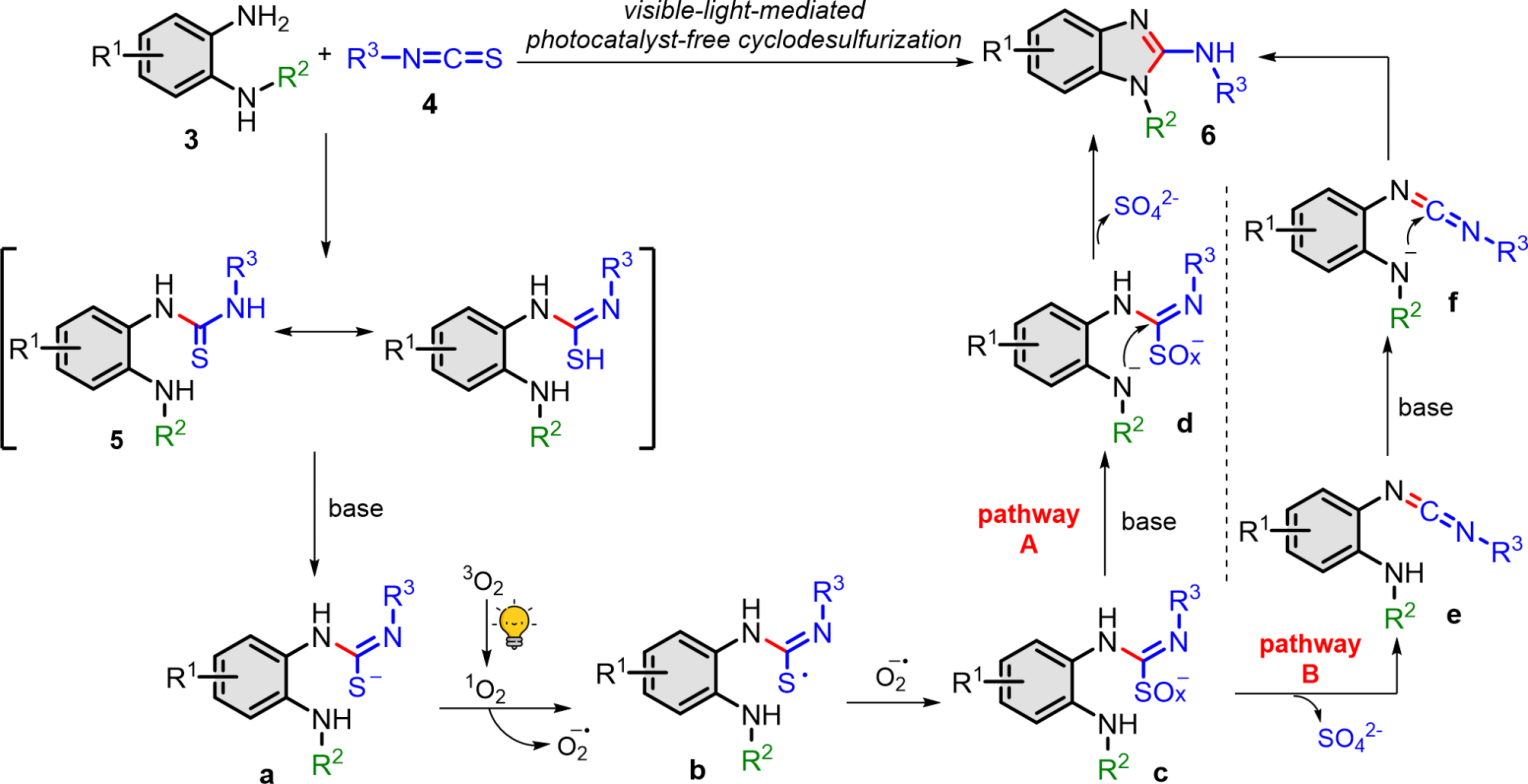
Proposed reaction mechanism.

Based on the evidences from above investigations, we therefore proposed our visible light mediated photocatalyst-free cyclodesulfurization mechanism as depicted in Fig. 6. The *o*-aminophenyl thiourea intermediate (**5**) which is formed between *N*-substituted *o*-phenylenediamine (**3**) and isothiocyanate (**4**) is deprotonated by base to generate the thiolate anion intermediate (**a**). In irradiation process, the triplet oxygen is excited to singlet oxygen^22,53^ which will further react with **a** to generate thiyl radical intermediate (**b**) and superoxide. This hypothesis is supported by the trapping experiments (Table 3). Afterwards, sulfur radical intermediate **b** will couple with superoxide forming peroxysulfur intermediate (**c**). Intermediated **c** can follow either pathway A or pathway B. For pathway A, intermediate **c** is further deprotonated into amino anion intermediate **d** which simultaneously undergoes cyclodesulfurization to generate *N*-substituted 2-aminobenzimidazoles (**6**) and liberate SO_4_^2-^ as supported by byproduct investigation (Fig. 5f). On the other hand, in pathway B, intermediate **c** can release SO_4_^2-^ and form carbodiimide intermediate **e**. The deprotonation of intermediate **e** generates amino anion intermediate **f** then cyclizing to provide target product **6**^29–36^.

## Conclusion

We have successfully developed a one-pot visible light mediated photocatalyst-free reaction for the synthesis of *N*-substituted 2-aminobenzimidazoles from *o*-phenylenediamines and isothiocyanates *via* a cyclodesulfurization process. This one-pot reaction combines three steps—*N*-substitution, thiourea formation, and cyclodesulfurization—carried out at room temperature. The method provides *N*-substituted 2-aminobenzimidazoles in fair to excellent yields and offers several advantages including high functional group tolerance, mild reaction condition and simplified, single-flask operation without the need of a photocatalyst. The scalability of the method has been demonstrated through gram-scale synthesis. Mechanistic studies suggest that the reaction proceeds *via* a radical pathway involving thiyl radical and reactive oxygen species. The coupling of these radicals results the formation of peroxysulfur intermediate which rapidly cyclizes to produce the desired product. This one-pot visible light mediated photocatalyst-free approach offers an environmentally friendly and efficient process for the preparation of *N*-substituted 2-aminobenzimidazoles which hold significant potential in pharmaceutical and medicinal chemistry.

## Methods

### General information

All chemicals and solvents were obtained from commercially available suppliers such as Eurisotop, Fluka, Merck, Sigma-Aldrich, TCI and Thermo scientific and were used without further purification, unless otherwise stated. The photochemical reactions were performed using (1) 19 W LED (Philips LED 19 W Durable Brightness Daylight E27), (2) LED strip reactor which made from 1000 mL beaker and linked with a commercial LED stirp (1.5 W × 150) and (3) 3 W LED which was placed on a glass rod fiber immersed in pyrex schlenk reactor. Analytical thin layer chromatography (TLC) was performed with precoated Merck silica gel 60 F254 plates (0.25 mm for thick layer) and visualized at 254 nm using an ultraviolet lamp. Column chromatography was performed with Silicycle silica gel 60–200 μm (70–230 mesh). ^1^H-NMR, ^13^C-NMR and ^19^F spectra were obtained with JEOL JNM-ECZ500R/S1 NMR spectrometers operating at 500 MHz for ^1^H or 126 MHz for ^13^C or 471 MHz for ^19^F nuclei. Data were reported with chemical shift, multiplicity (s = singlet, d = doublet, t = triplet, q = quartet, m = multiplet), coupling constant (Hz), and integration. EPR spectra were collected using an EPR Bruker system, ELEXSYS E-500 model at X-band microwave frequencies (approximately 9.85 GHz). High-resolution mass spectra (HRMS) were recorded using electron spray ionization (ESI) with a MicroTOF Bruker mass spectrometer. Melting point was measured by Stuart, SMP 20. X-ray diffraction (XRD) patterns were obtained on a DMAX 2200/Ultima + diffractometer (Rigaku) using Cu K*α* radiation source and operating at 40 kV and 30 mA. UV-vis spectra were measured using Agilent Cary 60 UV-vis spectrophotometer.

### General procedure for synthesis of *N*-substituted 2-aminobenzimidazoles

A mixture of *o*-phenylenediamines (1.2 eq, 0.48 mmol), protecting agents (1.2 eq, 0.48 mmol), K_2_CO_3_ (1.2 eq, 0.48 mmol) was dissolved by co-solvent between 90% ethanol and 10% water (3 mL) in pyrex schlenk reactor at room temperature for 1 hour under air. After that, the corresponding isothiocyanates (1.0 eq, 0.40 mmol) and K_2_CO_3_ (1.0 eq, 0.40 mmol) were added into pyrex schlenk reactor as single-flask operation. The reaction was irradiated by 3 W blue LED equipped with glass rod fiber at room temperature for 6 hours under air. The reaction mixture was washed water (2 × 10 mL) and the organic portion was extracted with EtOAc (3 × 10 mL). Water from organic layer was eliminated by Na_2_SO_4_. After filtration and removal of the solvent under reduced pressure, the crude product was purified by silica gel column chromatography using 3:1 of hexane: EtOAc as the mobile phase to afford *N*-substituted 2-aminobenzimidazoles **6a-6z**, **6a’-6y’** and **6a’’-6s’’** as the corresponding products.

### Characterization data of *N*-substituted 2-aminobenzimidazoles

***N*-(4-chlorophenyl)-1-tosyl-1*H*-benzo[*d*]imidazol-2-amine**^17^ (**6a**): white solid. ^1^H-NMR (500 MHz, CDCl_3_): *δ* (ppm) 8.69 (s, 1 H), 7.78 (dd, *J* = 7.9, 5.9 Hz, 3 H), 7.75–7.72 (m, 2 H), 7.44 (d, *J* = 7.7 Hz, 1 H), 7.37–7.34 (m, 2 H), 7.24 (dd, *J* = 10.6, 4.5 Hz, 3 H), 7.18–7.13 (m, 1 H), 2.33 (s, 3 H). ^13^C-NMR (126 MHz, CDCl_3_): *δ* (ppm) 147.52, 146.46, 142.17, 137.10, 134.11, 130.39, 129.97, 129.26, 128.28, 126.90, 125.27, 122.56, 120.40, 117.94, 112.83, 21.72. ESI-HRMS: m/z: found 398.07116 [M + H]^+^ (calcd for [C_20_H_17_ClN_3_O_2_S]^+^ 398.07300).

***N*-(2-chlorophenyl)-1-tosyl-1*H*-benzo[*d*]imidazol-2-amine**^17^ (**6b**): white solid. ^1^H-NMR (500 MHz, CDCl_3_): *δ* (ppm) 9.40 (s, 1 H), 8.77 (d, *J* = 8.3 Hz, 1 H), 7.88–7.84 (m, 3 H), 7.49–7.43 (m, 2 H), 7.37–7.33 (m, 1 H), 7.27–7.21 (m, 3 H), 7.19 (td, *J* = 7.8, 1.2 Hz, 1 H), 7.05–7.00 (m, 1 H), 2.32 (s, 3 H). ^13^C-NMR (126 MHz, CDCl_3_): *δ* (ppm) 147.09, 146.42, 141.94, 135.55, 134.22, 130.38, 130.00, 129.23, 127.96, 126.98, 125.19, 123.49, 122.75, 122.48, 120.05, 118.07, 112.86, 21.71. ESI-HRMS: m/z: found 398.07171 [M + H]^+^ (calcd for [C_20_H_17_ClN_3_O_2_S]^+^ 398.07300).

***N*-(2**,**4-dichlorophenyl)-1-tosyl-1*H*-benzo[*d*]imidazol-2-amine** (**6c**): white solid. melting point: 180–182 ^o^C. ^1^H-NMR (500 MHz, CDCl_3_), *δ* (ppm) 9.37 (s, 1 H), 8.75 (d, *J* = 8.9 Hz, 1 H), 7.83 (ddd, *J* = 6.7, 4.1, 2.3 Hz, 3 H), 7.45 (t, *J* = 4.7 Hz, 2 H), 7.30 (dd, *J* = 8.9, 2.4 Hz, 1 H), 7.26–7.23 (m, 3 H), 7.19 (td, *J* = 7.8, 1.2 Hz, 1 H), 2.34 (s, 3 H). ^13^C-NMR (126 MHz, CDCl_3_): *δ* (ppm) 146.78, 146.58, 141.80, 134.42, 134.18, 130.47, 129.99, 128.89, 128.04, 127.72, 127.00, 125.30, 122.98, 122.91, 120.70, 118.18, 112.91, 21.80. ESI-HRMS: m/z: found 432.03415 [M + H]^+^ (calcd for [C_20_H_16_Cl_2_N_3_O_2_S]^+^ 432.03403).

***N*-(4-fluorophenyl)-1-tosyl-1*H*-benzo[*d*]imidazol-2-amine**^17^ (**6d**): white solid. ^1^H-NMR (500 MHz, CDCl_3_): *δ* (ppm) 8.61 (s, 1 H), 7.78 (dd, *J* = 14.2, 8.2 Hz, 3 H), 7.74–7.69 (m, 2 H), 7.42 (d, *J* = 7.9 Hz, 1 H), 7.23 (ddd, *J* = 11.9, 6.5, 2.5 Hz, 3 H), 7.17–7.07 (m, 3 H), 2.34 (s, 3 H). ^13^C-NMR (126 MHz, CDCl_3_): *δ* (ppm) 159.02 (d, *J* = 242.67 Hz), 148.11, 146.43, 142.26, 134.53 (d, *J* = 1.91 Hz), 134.18, 130.39, 130.09, 126.93, 125.24, 122.39, 121.11 (d, *J* = 7.68 Hz), 117.82, 116.05, 115.87, 112.81, 21.73. ^19^F-NMR (471 MHz, CDCl_3_): *δ* (ppm) -119.12. ESI-HRMS: m/z: found 382.10249 [M + H]^+^ (calcd for [C_20_H_17_FN_3_O_2_S]^+^ 382.10255).

***N*-(2-fluorophenyl)-1-tosyl-1*H*-benzo[*d*]imidazol-2-amine**^17^ (**6e**): white solid. ^1^H-NMR (500 MHz, CDCl_3_): *δ* (ppm) 9.03 (s, 1 H), 8.63 (td, *J* = 8.3, 1.6 Hz, 1 H), 7.84 (dt, *J* = 8.6, 2.0 Hz, 3 H), 7.47 (dd, *J* = 4.3, 3.9 Hz, 1 H), 7.27–7.17 (m, 6 H), 7.08–7.03 (m, 1 H), 2.33 (s, 3 H). ^13^C-NMR (126 MHz, CDCl_3_): *δ* (ppm) 152.60 (d, *J* = 243.47 Hz), 147.22, 146.44, 142.13, 134.19, 130.39, 130.06, 127.11 (d, *J* = 9.99 Hz), 126.97, 125.21, 124.82 (d, *J* = 3.44 Hz), 123.31 (d, *J* = 7.42 Hz), 122.65, 120.58, 118.03, 114.88 (d, *J* = 18.90 Hz), 112.84, 21.71. ^19^F-NMR (471 MHz, CDCl_3_): *δ* (ppm) -131.60. ESI-HRMS: m/z: found 382.10206 [M + H]^+^ (calcd for [C_20_H_17_FN_3_O_2_S]^+^ 382.10255).

***N*-(2**,**4-difluorophenyl)-1-tosyl-1*H*-benzo[*d*]imidazol-2-amine** (**6f**): white solid. melting point: 184–186 ^o^C. ^1^H-NMR (500 MHz, CDCl_3_): *δ* (ppm) 8.84 (d, *J* = 2.6 Hz, 1 H), 8.59–8.49 (m, 1 H), 7.83–7.78 (m, 3 H), 7.42 (dd, *J* = 4.3, 3.9 Hz, 1 H), 7.26–7.21 (m, 9 H), 7.16 (td, *J* = 7.8, 1.2 Hz, 1 H), 6.99–6.91 (m, 2 H), 2.34 (s, 3 H). ^13^C-NMR (126 MHz, CDCl_3_): *δ* (ppm) 159.02 (d, *J* = 11.04 Hz), 157.07 (d, *J* = 10.89 Hz), 153.58 (d, *J* = 11.61 Hz), 151.62 (d, *J* = 12.02 Hz), 147.41, 146.54, 142.04, 134.18, 130.45, 130.13, 127.01, 125.27, 123.51 (dd, *J* = 10.27, 3.68 Hz), 122.70, 121.68 (d, *J* = 8.64 Hz), 118.01, 112.84, 111.43 (d, *J* = 3.66 Hz), 111.26 (d, *J* = 3.63 Hz), 103.79 (d, *J* = 26.82, 22.82 Hz), 21.78. ^19^F-NMR (471 MHz, CDCl_3_): *δ* (ppm) -116.43, -126.67. ESI-HRMS: m/z: found 400.09444 [M + H]^+^ (calcd for [C_20_H_16_F_2_N_3_O_2_S]^+^ 400.09313).

***N*-(4-bromophenyl)-1-tosyl-1*H*-benzo[*d*]imidazol-2-amine**^17^ (**6g**): white solid. ^1^H-NMR (500 MHz, CDCl_3_): *δ* (ppm) 8.70 (s, 1 H), 7.78 (dt, *J* = 7.5, 2.3 Hz, 3 H), 7.71–7.67 (m, 2 H), 7.52–7.48 (m, 2 H), 7.46–7.43 (m, 1 H), 7.25–7.21 (m, 3 H), 7.16 (td, *J* = 7.8, 1.2 Hz, 1 H), 2.33 (s, 3 H). ^13^C-NMR (126 MHz, CDCl_3_), *δ* (ppm) 147.46, 146.50, 142.19, 137.63, 134.14, 132.23, 130.43, 129.99, 126.93, 125.31, 122.62, 120.76, 117.99, 115.82, 112.87, 21.77. ESI-HRMS: m/z: found 442.01933 [M + H]^+^ (calcd for [C_20_H_17_BrN_3_O_2_S]^+^ 442.02249).

***N*-(3-bromophenyl)-1-tosyl-1*H*-benzo[*d*]imidazol-2-amine**^17^ (**6h**): white solid. ^1^H-NMR (500 MHz, CDCl_3_): *δ* (ppm) 8.70 (s, 1 H), 8.06 (d, *J* = 1.9 Hz, 1 H), 7.76 (dd, *J* = 8.4, 1.8 Hz, 3 H), 7.66 (dt, *J* = 7.3, 2.1 Hz, 1 H), 7.45 (d, *J* = 7.8 Hz, 1 H), 7.25–7.21 (m, 5 H), 7.15 (td, *J* = 8.1, 1.0 Hz, 1 H), 2.33 (s, 3 H). ^13^C-NMR (126 MHz, CDCl_3_): *δ* (ppm) 147.19, 146.52, 142.13, 139.80, 134.12, 130.59, 130.45, 129.96, 126.93, 126.30, 125.32, 123.02, 122.71, 121.85, 118.14, 117.59, 112.87, 21.77. ESI-HRMS: m/z: found 442.02046 [M + H]^+^ (calcd for [C_20_H_17_BrN_3_O_2_S]^+^ 442.02248).

***N*-(4-iodophenyl)-1-tosyl-1*H*-benzo[*d*]imidazol-2-amine**^17^ (**6i**): white solid. ^1^H-NMR (500 MHz, CDCl_3_): *δ* (ppm) 8.69 (s, 1 H), 7.79–7.76 (m, 3 H), 7.70–7.66 (m, 2 H), 7.59–7.56 (m, 2 H), 7.44 (dd, *J* = 7.9, 0.4 Hz, 1 H), 7.23 (ddd, *J* = 8.2, 5.5, 1.7 Hz, 3 H), 7.16 (td, *J* = 7.9, 1.2 Hz, 1 H), 2.33 (s, 3 H). ^13^C-NMR (126 MHz, CDCl_3_): *δ* (ppm) 147.29, 146.44, 142.14, 138.27, 138.09, 134.05, 130.38, 129.91, 126.87, 125.26, 122.60, 121.05, 117.97, 112.82, 86.11, 21.73. ESI-HRMS: m/z: found 490.00709 [M + H]^+^ (calcd for [C_20_H_17_IN_3_O_2_S]^+^ 490.00863).

***N*-(3-iodophenyl)-1-tosyl-1*H*-benzo[*d*]imidazol-2-amine**^17^ (**6j**): white solid. ^1^H-NMR (500 MHz, CDCl_3_): *δ* (ppm) 8.66 (s, 1 H), 8.16 (t, *J* = 1.8 Hz, 1 H), 7.78–7.74 (m, 4 H), 7.44 (dd, *J* = 10.6, 8.0 Hz, 2 H), 7.23 (ddd, *J* = 7.6, 5.3, 2.0 Hz, 3 H), 7.17–7.13 (m, 1 H), 7.10 (t, *J* = 8.0 Hz, 1 H), 2.33 (s, 3 H). ^13^C-NMR (126 MHz, CDCl_3_): *δ* (ppm) 147.14, 146.51, 142.12, 139.64, 134.08, 132.34, 130.77, 130.45, 129.94, 127.58, 126.92, 125.30, 122.68, 118.28, 118.11, 112.84, 94.56, 21.78. ESI-HRMS: m/z: found 490.00594 [M + H]^+^ (calcd for [C_20_H_17_IN_3_O_2_S]^+^ 490.00863).

***N*-phenyl-1-tosyl-1*H*-benzo[*d*]imidazol-2-amine**^17^ (**6k**): white solid. ^1^H-NMR (500 MHz, CDCl_3_): *δ* (ppm) 8.69 (s, 1 H), 7.82–7.76 (m, 5 H), 7.46–7.39 (m, 3 H), 7.23 (ddd, *J* = 7.8, 4.0, 1.2 Hz, 3 H), 7.17–7.10 (m, 2 H), 2.34 (s, 3 H). ^13^C-NMR (126 MHz, CDCl_3_): *δ* (ppm) 147.91, 146.36, 142.46, 138.51, 134.26, 130.39, 130.06, 129.38, 126.95, 125.22, 123.49, 122.36, 119.25, 117.90, 112.85, 21.74. ESI-HRMS: m/z: found 364.11087 [M + H]^+^ (calcd for [C_20_H_18_N_3_O_2_S]^+^ 364.11197).

***N*-(*p*-tolyl)-1-tosyl-1*H*-benzo[*d*]imidazol-2-amine**^17^ (**6l**): white solid. ^1^H-NMR (500 MHz, CDCl_3_): *δ* (ppm) 8.60 (s, 1 H), 7.82–7.77 (m, 3 H), 7.65–7.62 (m, 2 H), 7.43 (d, *J* = 7.7 Hz, 1 H), 7.24–7.20 (m, 5 H), 7.16–7.11 (m, 1 H), 2.36 (s, 3 H), 2.33 (s, 3 H). ^13^C-NMR (126 MHz, CDCl_3_): *δ* (ppm) 148.24, 146.29, 142.49, 135.88, 134.26, 133.24, 130.34, 130.10, 129.85, 126.93, 125.17, 122.19, 119.56, 117.75, 112.81, 21.71, 20.93. ESI-HRMS: m/z: found 378.12781 [M + H]^+^ (calcd for [C_21_H_20_N_3_O_2_S]^+^ 378.12762).

***N*-(*m*-tolyl)-1-tosyl-1*H*-benzo[*d*]imidazol-2-amine**^17^ (**6m**): white solid. ^1^H-NMR (500 MHz, CDCl_3_): *δ* (ppm) 8.69 (s, 1 H), 7.82 (d, *J* = 8.4 Hz, 3 H), 7.66–7.63 (m, 1 H), 7.61 (s, 1 H), 7.50–7.47 (m, 1 H), 7.32 (t, *J* = 7.8 Hz, 1 H), 7.25 (td, *J* = 7.8, 1.0 Hz, 1 H), 7.21 (d, *J* = 8.3 Hz, 2 H), 7.17 (td, *J* = 8.1, 1.0 Hz, 1 H), 6.97 (d, *J* = 7.5 Hz, 1 H), 2.43 (s, 3 H), 2.31 (s, 3 H). ^13^C-NMR (126 MHz, CDCl_3_): *δ* (ppm) 147.86, 146.22, 142.40, 139.19, 138.30, 134.13, 130.25, 129.96, 129.13, 126.82, 125.12, 124.29, 122.23, 119.76, 117.77, 116.32, 112.76, 21.60. ESI-HRMS: m/z: found 378.12815 [M + H]^+^ (calcd for [C_21_H_20_N_3_O_2_S]^+^ 378.12762).

***N*-(*o*-tolyl)-1-tosyl-1*H*-benzo[*d*]imidazol-2-amine**^17^ (**6n**): white solid. ^1^H-NMR (500 MHz, CDCl_3_): *δ* (ppm) 8.68 (s, 1 H), 8.40 (d, *J* = 8.2 Hz, 1 H), 7.86–7.80 (m, 3 H), 7.49–7.45 (m, 1 H), 7.34 (t, *J* = 7.7 Hz, 1 H), 7.29–7.22 (m, 4 H), 7.16 (td, *J* = 7.9, 1.2 Hz, 1 H), 7.09 (dd, *J* = 10.7, 4.2 Hz, 1 H), 2.46 (s, 3 H), 2.33 (s, 3 H). ^13^C-NMR (126 MHz, CDCl_3_): *δ* (ppm) 148.14, 146.28, 142.31, 136.88, 134.26, 130.59, 130.29, 130.07, 127.19, 127.13, 126.80, 125.09, 123.70, 122.21, 120.09, 117.81, 112.65, 21.63, 17.98. ESI-HRMS: m/z: found 378.12793 [M + H]^+^ (calcd for [C_21_H_20_N_3_O_2_S]^+^ 378.12762).

***N*-(3**,**5-dimethylphenyl)-1-tosyl-1*H*-benzo[*d*]imidazol-2-amine**^17^ (**6o**): white solid. ^1^H-NMR (500 MHz, CDCl_3_): *δ* (ppm) 8.62 (s, 1 H), 7.79 (d, *J* = 8.2 Hz, 3 H), 7.46 (d, *J* = 7.9 Hz, 1 H), 7.41 (s, 2 H), 7.22 (dd, *J* = 13.4, 8.0 Hz, 3 H), 7.14 (t, *J* = 7.8 Hz, 1 H), 6.78 (s, 1 H), 2.38 (s, 6 H), 2.32 (s, 3 H). ^13^C-NMR (126 MHz, CDCl_3_): *δ* (ppm) 147.96, 146.23, 142.50, 139.07, 138.25, 134.22, 130.30, 130.01, 126.88, 125.35, 125.13, 122.21, 117.81, 116.99, 112.80, 21.66, 21.55. ESI-HRMS: m/z: found 392.14331 [M + H]^+^ (calcd for [C_22_H_22_N_3_O_2_S]^+^ 392.14327).

***N*-(4-ethylphenyl)-1-tosyl-1*H*-benzo[*d*]imidazol-2-amine**^17^ (**6p**): colorless oil. ^1^H-NMR (500 MHz, CDCl_3_): *δ* (ppm) 8.60 (s, 1 H), 7.82–7.76 (m, 3 H), 7.66 (dd, *J* = 8.4, 2.9 Hz, 2 H), 7.43 (d, *J* = 7.8 Hz, 1 H), 7.26–7.19 (m, 5 H), 7.15–7.10 (m, 1 H), 2.66 (q, *J* = 7.6 Hz, 2 H), 2.32 (s, 3 H), 1.26 (t, *J* = 7.6 Hz, 3 H). ^13^C-NMR (126 MHz, CDCl_3_): *δ* (ppm) 148.24, 146.28, 142.48, 139.71, 136.04, 134.25, 130.33, 130.10, 128.68, 126.92, 125.16, 122.17, 119.65, 117.75, 112.79, 28.37, 21.69, 15.87. ESI-HRMS: m/z: found 392.14307 [M + H]^+^ (calcd for [C_22_H_22_N_3_O_2_S]^+^ 392.14327).

***N*-(4-methoxyphenyl)-1-tosyl-1*H*-benzo[*d*]imidazol-2-amine**^17^ (**6q**): white solid. ^1^H-NMR (500 MHz, CDCl_3_): *δ* (ppm) 8.48 (s, 1 H), 7.80 (d, *J* = 8.5 Hz, 2 H), 7.76 (d, *J* = 8.0 Hz, 1 H), 7.65–7.61 (m, 2 H), 7.40 (d, *J* = 7.9 Hz, 1 H), 7.21 (ddd, *J* = 16.9, 8.2, 0.8 Hz, 3 H), 7.14–7.09 (m, 1 H), 6.97–6.93 (m, 2 H), 3.82 (s, 3 H), 2.34 (s, 3 H). ^13^C-NMR (126 MHz, CDCl_3_): *δ* (ppm) 1156.16, 148.73, 146.28, 142.57, 134.28, 131.59, 130.43, 130.32, 126.93, 125.13, 122.04, 121.53, 117.65, 114.56, 112.76, 55.64, 21.71. ESI-HRMS: m/z: found 394.12167 [M + H]^+^ (calcd for [C_21_H_20_N_3_O_3_S]^+^ 394.12254).

***N*-(3-methoxyphenyl)-1-tosyl-1*H*-benzo[*d*]imidazol-2-amine**^17^ (**6r**): colorless oil. ^1^H-NMR (500 MHz, CDCl_3_): *δ* (ppm) 8.68 (s, 1 H), 7.79–7.75 (m, 3 H), 7.51 (t, *J* = 2.2 Hz, 1 H), 7.44–7.41 (m, 1 H), 7.27 (ddd, *J* = 11.0, 7.5, 4.6 Hz, 2 H), 7.23–7.20 (m, 3 H), 7.15–7.11 (m, 1 H), 6.67 (ddd, *J* = 7.9, 2.4, 1.1 Hz, 1 H), 3.85 (s, 3 H), 2.32 (s, 3 H). ^13^C-NMR (126 MHz, CDCl_3_): *δ* (ppm) 160.52, 147.72, 146.37, 142.44, 139.68, 134.21, 130.38, 130.06, 129.99, 126.94, 125.21, 122.40, 117.98, 112.85, 111.57, 108.89, 105.25, 55.48, 21.74. ESI-HRMS: m/z: found 394.12252 [M + H]^+^ (calcd for [C_21_H_20_N_3_O_3_S]^+^ 394.12254 ).

***N*-(2-methoxyphenyl)-1-tosyl-1*H*-benzo[*d*]imidazol-2-amine**^17^ (**6s**): colorless oil. ^1^H-NMR (500 MHz, CDCl_3_): *δ* (ppm) 9.44 (s, 1 H), 8.66–8.60 (m, 1 H), 7.84 (d, *J* = 8.4 Hz, 3 H), 7.46 (dd, *J* = 7.7, 0.4 Hz, 1 H), 7.23 (td, *J* = 7.7, 1.2 Hz, 1 H), 7.19 (d, *J* = 8.3 Hz, 2 H), 7.15 (td, *J* = 8.0, 1.1 Hz, 1 H), 7.09–7.04 (m, 2 H), 6.98–6.95 (m, 1 H), 4.02 (s, 3 H), 2.30 (s, 3 H). ^13^C-NMR (126 MHz, CDCl_3_): *δ* (ppm) 148.11, 147.70, 146.16, 142.54, 134.32, 130.24, 130.02, 128.33, 126.91, 125.04, 122.73, 122.21, 121.26, 118.56, 117.75, 112.75, 110.04, 56.11, 21.64. ESI-HRMS: m/z: found 394.12207 [M + H]^+^ (calcd for [C_21_H_20_N_3_O_3_S]^+^ 394.12254).

**1-tosyl-*N*-(4-(trifluoromethoxy)phenyl)-1*H*-benzo[*d*]imidazol-2-amine** (**6t**): white solid. melting point: 136–138 ^o^C. ^1^H-NMR (500 MHz, CDCl_3_): *δ* (ppm) 8.73 (s, 1 H), 7.79 (dd, *J* = 16.5, 8.2 Hz, 5 H), 7.44 (d, *J* = 7.9 Hz, 1 H), 7.27–7.21 (m, 5 H), 7.15 (t, *J* = 7.7 Hz, 1 H), 2.33 (s, 3 H). ^13^C-NMR (126 MHz, CDCl_3_): *δ* (ppm) 147.57, 146.54, 144.66, 142.12, 137.25, 134.14, 130.43, 130.03, 126.93, 125.31, 122.64, 122.19, 120.66 (q, *J* = 255.92 Hz), 120.25, 118.00, 112.86, 21.71. ^19^F-NMR (471 MHz, CDCl_3_): *δ* (ppm) -57.99. ESI-HRMS: m/z: found 448.09311 [M + H]^+^ (calcd for [C_21_H_17_F_3_N_3_O_3_S]^+^ 448.09427).

***N***^1^,***N***^1^**-dimethyl-*N***^4^**-(1-tosyl-1*H*-benzo[*d*]imidazol-2-yl)benzene-1**,**4-diamine** (**6u**): yellow oil. ^1^H-NMR (500 MHz, CDCl_3_): *δ* (ppm) 8.36 (s, 1 H), 7.83–7.79 (m, 2 H), 7.76 (d, *J* = 7.9 Hz, 1 H), 7.57–7.50 (m, 2 H), 7.38 (dd, *J* = 7.9, 0.5 Hz, 1 H), 7.25–7.22 (m, 2 H), 7.19 (td, *J* = 7.7, 1.2 Hz, 1 H), 7.12–7.08 (m, 1 H), 6.84–6.78 (m, 2 H), 2.95 (s, 6 H), 2.34 (s, 3 H). ^13^C-NMR (126 MHz, CDCl_3_): *δ* (ppm) 149.23, 147.83, 146.18, 142.80, 134.35, 130.32, 130.30, 128.37, 126.95, 125.07, 121.91, 121.77, 117.53, 113.71, 112.72, 41.22, 21.73. ESI-HRMS: m/z: found 407.15257 [M + H]^+^ (calcd for [C_22_H_23_N_4_O_2_S]^+^ 407.15417).

***N*-(2**,**6-dimethylphenyl)-1-tosyl-1*H*-benzo[*d*]imidazol-2-amine** (**6v**): white solid. melting point: 174–176 ^o^C. ^1^H-NMR (500 MHz, CDCl_3_): *δ* (ppm) 7.93 (d, *J* = 8.4 Hz, 2 H), 7.81 (d, *J* = 7.6 Hz, 1 H), 7.76 (s, 1 H), 7.32 (dd, *J* = 7.4, 5.2 Hz, 3 H), 7.19–7.10 (m, 5 H), 2.41 (s, 3 H), 2.22 (s, 6 H). ^13^C-NMR (126 MHz, CDCl_3_): *δ* (ppm) 149.57, 146.40, 142.46, 135.38, 134.76, 134.64, 131.03, 130.34, 128.82, 127.55, 127.17, 124.90, 121.74, 117.71, 112.44, 21.84, 18.65. ESI-HRMS: m/z: found 392.14327 [M + H]^+^ (calcd for [C_22_H_22_N_3_O_2_S]^+^ 392.14406).

***N*-(2**,**6-diisopropylphenyl)-1-tosyl-1*H*-benzo[*d*]imidazol-2-amine** (**6w**): white solid. melting point: 150–153 ^o^C. ^1^H-NMR (500 MHz, CDCl_3_): *δ* (ppm) 7.94–7.90 (m, 2 H), 7.80 (dd, *J* = 8.1, 0.6 Hz, 1 H), 7.71 (s, 1 H), 7.36–7.29 (m, 4 H), 7.23 (d, *J* = 7.7 Hz, 2 H), 7.16 (td, *J* = 7.7, 1.2 Hz, 1 H), 7.10 (td, *J* = 7.8, 1.2 Hz, 1 H), 3.07–2.97 (m, 2 H), 2.43 (s, 3 H), 1.20 (d, *J* = 6.8 Hz, 6 H), 1.14 (d, *J* = 6.8 Hz, 6 H). ^13^C-NMR (126 MHz, CDCl_3_): *δ* (ppm) 151.08, 146.34, 146.22, 142.58, 134.90, 131.95, 131.17, 130.34, 128.73, 127.11, 124.90, 124.10, 121.61, 117.76, 112.41, 28.86, 24.41, 23.17, 21.85. ESI-HRMS: m/z: found 448.20635 [M + H]^+^ (calcd for [C_26_H_30_N_3_O_2_S]^+^ 448.20587).

**3-((1-tosyl-1*H*-benzo[*d*]imidazol-2-yl)amino)phenol** (**6y**): white solid. melting point: 185–188 ^o^C. ^1^H-NMR (500 MHz, acetone-d_6_): *δ* (ppm) 8.79 (s, 1 H), 8.57 (s, 1 H), 7.95–7.92 (m, 2 H), 7.82 (ddd, *J* = 8.0, 1.0, 0.5 Hz, 1 H), 7.61 (t, *J* = 2.1 Hz, 1 H), 7.40–7.35 (m, 3 H), 7.21 (dddd, *J* = 15.5, 9.1, 7.1, 1.2 Hz, 4 H), 6.62 (ddd, *J* = 7.8, 2.3, 1.1 Hz, 1 H), 2.33 (s, 3 H). ^13^C-NMR (126 MHz, acetone-d_6_): *δ* (ppm) 158.92, 148.54, 147.68, 143.47, 140.79, 134.75, 131.29, 130.73, 130.51, 127.81, 125.99, 123.05, 118.34, 113.72, 111.12, 110.97, 107.05, 21.44. ESI-HRMS: m/z: found 380.10509 [M + H]^+^ (calcd for [C_20_H_18_N_3_O_3_S]^+^ 380.10689).

***N*-(4-((tert-butyldimethylsilyl)oxy)phenyl)-1-tosyl-1*H*-benzo[*d*]imidazol-2-amine**^17^ (**6z**): colorless oil. ^1^H-NMR (500 MHz, CDCl_3_): *δ* (ppm) 8.50 (s, 1 H), 7.80 (d, *J* = 8.4 Hz, 2 H), 7.75 (d, *J* = 8.0 Hz, 1 H), 7.62–7.57 (m, 2 H), 7.40 (d, *J* = 7.9 Hz, 1 H), 7.26–7.18 (m, 3 H), 7.11 (t, *J* = 7.7 Hz, 1 H), 6.88 (d, *J* = 8.6 Hz, 2 H), 2.35 (s, 3 H), 1.01 (s, 9 H), 0.22 (s, 6 H). ^13^C-NMR (126 MHz, CDCl_3_): *δ* (ppm) 151.95, 148.52, 146.30, 142.59, 134.30, 132.15, 130.37, 130.17, 126.98, 125.16, 122.08, 121.04, 120.70, 117.69, 112.78, 25.83, 21.77, 18.34, -4.30. ESI-HRMS: m/z: found 494.19190 [M + H]^+^ (calcd for [C_26_H_32_N_3_O_3_SSi]^+^ 494.19337).

***N*-(3-((tert-butyldimethylsilyl)oxy)phenyl)-1-tosyl-1*H*-benzo[*d*]imidazol-2-amine**^17^ (**6a’**): colorless oil. ^1^H-NMR (500 MHz, CDCl_3_): *δ* (ppm) 8.64 (s, 1 H), 7.79 (t, *J* = 8.7 Hz, 3 H), 7.48–7.41 (m, 2 H), 7.32 (dd, *J* = 8.1, 1.0 Hz, 1 H), 7.27–7.21 (m, 4 H), 7.15 (t, *J* = 7.7 Hz, 1 H), 6.63 (dd, *J* = 8.0, 0.9 Hz, 1 H), 2.34 (s, 3 H), 1.04 (s, 9 H), 0.29 (s, 6 H). ^13^C-NMR (126 MHz, CDCl_3_): *δ* (ppm) 156.57, 147.73, 146.33, 142.50, 139.55, 134.23, 130.37, 130.01, 129.94, 126.96, 125.17, 122.35, 117.92, 115.09, 112.83, 112.10, 111.11, 25.85, 21.74, 18.37, -4.27. ESI-HRMS: m/z: found 494.19281 [M + H]^+^ (calcd for [C_26_H_32_N_3_O_3_SSi]^+^ 494.19337).

***N*-(4-(benzyloxy)phenyl)-1-tosyl-1*H*-benzo[*d*]imidazol-2-amine** (**6b’**): pale yellow solid. melting point: 120–123 ^o^C. ^1^H-NMR (500 MHz, CDCl_3_): *δ* (ppm) 8.51 (s, 1 H), 7.81 (d, *J* = 8.4 Hz, 2 H), 7.78 (d, *J* = 8.0 Hz, 1 H), 7.67–7.62 (m, 2 H), 7.46 (d, *J* = 7.4 Hz, 2 H), 7.41 (t, *J* = 7.4 Hz, 3 H), 7.35 (d, *J* = 7.3 Hz, 1 H), 7.22 (dd, *J* = 14.3, 7.9 Hz, 3 H), 7.13 (t, *J* = 7.7 Hz, 1 H), 7.07–7.01 (m, 2 H), 5.09 (s, 2 H), 2.35 (s, 3 H). ^13^C-NMR (126 MHz, CDCl_3_): *δ* (ppm) 155.30, 148.62, 146.30, 142.49, 137.09, 134.25, 131.80, 130.35, 130.18, 128.68, 128.06, 127.55, 126.93, 125.16, 122.09, 121.42, 117.66, 115.64, 112.77, 70.43, 21.73. ESI-HRMS: m/z: found 470.15276 [M + H]^+^ (calcd for [C_27_H_24_N_3_O_3_S]^+^ 470.15384).

**4-((1-tosyl-1*H*-benzo[*d*]imidazol-2-yl)amino)phenyl 4-methylbenzenesulfonate** (**6c’**): white solid. melting point: 158–161 ^o^C. ^1^H-NMR (500 MHz, CDCl_3_): *δ* (ppm) 8.73 (s, 1 H), 7.77–7.69 (m, 7 H), 7.41 (d, *J* = 7.7 Hz, 1 H), 7.32 (d, *J* = 8.1 Hz, 2 H), 7.25–7.20 (m, 3 H), 7.14 (td, *J* = 7.8, 1.2 Hz, 1 H), 7.02–6.99 (m, 2 H), 2.45 (s, 3 H), 2.34 (s, 3 H). ^13^C-NMR (126 MHz, CDCl_3_): *δ* (ppm) 147.34, 146.57, 145.51, 144.97, 141.93, 137.34, 134.05, 132.36, 130.45, 129.91, 129.88, 128.66, 126.93, 125.31, 123.31, 122.68, 119.84, 117.90, 112.83, 21.83, 21.76. ESI-HRMS: m/z: found 534.11254 [M + H]^+^ (calcd for [C_27_H_24_N_3_O_5_S_2_]^+^ 534.11574).

**1-tosyl-*N*-(4-(trifluoromethyl)phenyl)-1*H*-benzo[*d*]imidazol-2-amine**^17^ (**6d’**): white solid. ^1^H-NMR (500 MHz, CDCl_3_): *δ* (ppm) 8.90 (s, 1 H), 7.91 (d, *J* = 8.5 Hz, 2 H), 7.78 (d, *J* = 8.4 Hz, 3 H), 7.64 (d, *J* = 8.6 Hz, 2 H), 7.47 (d, *J* = 7.9 Hz, 1 H), 7.27–7.21 (m, 3 H), 7.20–7.15 (m, 1 H), 2.32 (s, 3 H). ^13^C-NMR (126 MHz, CDCl_3_): *δ* (ppm) 146.97, 146.62, 141.98, 141.55, 134.06, 130.45, 129.92, 126.92, 126.60 (q, *J* = 3.92 Hz), 125.47, 124.93 (q, *J* = 33.80 Hz), 124.38 (q, *J* = 272.16 Hz), 122.92, 118.60, 118.21, 112.91, 21.70. ^19^F-NMR (471 MHz, CDCl_3_): *δ* (ppm) -61.68. ESI-HRMS: m/z: found 432.09785 [M + H]^+^ (calcd for [C_21_H_17_F_3_N_3_O_2_S]^+^ 432.09936).

**1-tosyl-*N*-(3-(trifluoromethyl)phenyl)-1*H*-benzo[*d*]imidazol-2-amine**^17^ (**6e’**): white solid. ^1^H-NMR (500 MHz, CDCl_3_): *δ* (ppm) 8.84 (s, 1 H), 8.11 (s, 1 H), 7.99 (dd, *J* = 8.2, 1.6 Hz, 1 H), 7.80–7.76 (m, 3 H), 7.52–7.45 (m, 2 H), 7.37–7.34 (m, 1 H), 7.24 (dd, *J* = 12.3, 4.2 Hz, 3 H), 7.17 (ddd, *J* = 8.1, 7.7, 0.7 Hz, 1 H), 2.33 (s, 3 H). ^13^C-NMR (126 MHz, CDCl_3_): *δ* (ppm) 147.19, 146.60, 142.00, 139.07, 134.09, 131.68 (q, *J* = 32.44 Hz), 129.94, 129.88, 126.94, 125.35, 124.09 (q, *J* = 272.51 Hz), 122.81, 122.11, 119.86 (q, *J* = 3.63 Hz), 118.20, 115.76 (q, *J* = 3.81 Hz), 112.86, 21.72. ^19^F-NMR (471 MHz, CDCl_3_): *δ* (ppm) -62.44. ESI-HRMS: m/z: found 432.09820 [M + H]^+^ (calcd for [C_21_H_17_F_3_N_3_O_2_S]^+^ 432.09936).

**1-tosyl-*N*-(2-(trifluoromethyl)phenyl)-1*H*-benzo[*d*]imidazol-2-amine** (**6f’**): white solid. melting point: 138–141 ^o^C. ^1^H-NMR (500 MHz, CDCl_3_): *δ* (ppm) 9.16 (s, 1 H), 8.70 (d, *J* = 8.4 Hz, 1 H), 7.88–7.85 (m, 2 H), 7.85–7.82 (m, 1 H), 7.69 (d, *J* = 7.9 Hz, 1 H), 7.63 (t, *J* = 7.9 Hz, 1 H), 7.47–7.44 (m, 1 H), 7.27–7.17 (m, 5 H), 2.35 (s, 3 H). ^13^C-NMR (126 MHz, CDCl_3_): *δ* (ppm) 147.51, 146.48, 141.68, 136.40, 134.22, 133.27, 130.39, 130.18, 127.05, 126.39 (q, *J* = 5.12 Hz), 125.21, 124.32 (q, *J* = 272.82 Hz), 123.28, 122.80, 122.45, 119.40 (q, *J* = 29.89 Hz), 112.84, 21.73. ^19^F-NMR (471 MHz, CDCl_3_): *δ* (ppm) -60.88. ESI-HRMS: m/z: found 432.09814 [M + H]^+^ (calcd for [C_21_H_17_F_3_N_3_O_2_S]^+^ 432.09936).

***N*-(4-nitrophenyl)-1-tosyl-1*H*-benzo[*d*]imidazol-2-amine**^17^ (**6g’**): yellow solid. ^1^H-NMR (500 MHz, CDCl_3_): *δ* (ppm) 9.12 (s, 1 H), 8.29–8.26 (m, 2 H), 7.97–7.94 (m, 2 H), 7.78–7.75 (m, 3 H), 7.50–7.48 (m, 1 H), 7.29–7.24 (m, 3 H), 7.22–7.18 (m, 1 H), 2.35 (s, 3 H). ^13^C-NMR (126 MHz, CDCl_3_): *δ* (ppm) 146.89, 146.14, 144.27, 142.72, 141.63, 133.91, 130.60, 129.83, 126.99, 125.59, 123.46, 118.54, 118.21, 112.96, 21.85. ESI-HRMS: m/z: found 409.09485 [M + H]^+^ (calcd for [C_20_H_17_N_4_O_4_S]^+^ 409.09705).

***N*-(3-nitrophenyl)-1-tosyl-1*H*-benzo[*d*]imidazol-2-amine**^17^ (**6h’**): yellow solid. ^1^H-NMR (500 MHz, CDCl_3_): *δ* (ppm) 8.92 (s, 1 H), 8.76 (t, *J* = 2.2 Hz, 1 H), 8.08 (ddd, *J* = 8.1, 2.2, 0.8 Hz, 1 H), 7.91 (ddd, *J* = 8.2, 2.2, 0.8 Hz, 1 H), 7.79–7.77 (m, 2 H), 7.76–7.74 (m, 1 H), 7.52 (t, *J* = 8.2 Hz, 1 H), 7.47 (d, *J* = 7.6 Hz, 1 H), 7.26–7.22 (m, 3 H), 7.17 (td, *J* = 7.8, 1.2 Hz, 1 H), 2.33 (s, 3 H). ^13^C-NMR (126 MHz, CDCl_3_): *δ* (ppm) 148.92, 146.77, 146.72, 141.73, 139.65, 133.93, 130.50, 130.04, 129.85, 126.93, 125.41, 124.55, 123.04, 118.34, 117.82, 113.70, 112.82, 21.77. ESI-HRMS: m/z: found 409.09459 [M + H]^+^ (calcd for [C_20_H_17_N_4_O_4_S]^+^ 409.09705).

**4-((1-tosyl-1*H*-benzo[*d*]imidazol-2-yl)amino)benzonitrile**^17^ (**6i’**): white solid. ^1^H-NMR (500 MHz, CDCl_3_): *δ* (ppm) 8.99 (s, 1 H), 7.93 (d, *J* = 8.8 Hz, 2 H), 7.77 (d, *J* = 8.4 Hz, 3 H), 7.68 (d, *J* = 8.8 Hz, 2 H), 7.48 (d, *J* = 7.7 Hz, 1 H), 7.26 (dd, *J* = 7.9, 7.4 Hz, 3 H), 7.19 (td, *J* = 7.9, 1.2 Hz, 1 H), 2.36 (s, 3 H). ^13^C-NMR (126 MHz, CDCl_3_): *δ* (ppm) 146.79, 146.38, 142.44, 141.76, 133.99, 133.64, 130.56, 129.83, 126.97, 125.51, 123.27, 119.25, 118.79, 118.41, 112.95, 105.89, 21.83. ESI-HRMS: m/z: found 389.10475 [M + H]^+^ (calcd for [C_21_H_17_N_4_O_2_S]^+^ 389.10722).

**3-((1-tosyl-1*H*-benzo[*d*]imidazol-2-yl)amino)benzonitrile** (**6j’**): pale yellow solid. melting point: 160–162 ^o^C. ^1^H-NMR (500 MHz, CDCl_3_): *δ* (ppm) 8.82 (s, 1 H), 8.35 (d, *J* = 1.5 Hz, 1 H), 7.83 (d, *J* = 8.3 Hz, 1 H), 7.76 (dd, *J* = 12.1, 5.0 Hz, 3 H), 7.46 (ddd, *J* = 8.2, 4.7, 1.5 Hz, 2 H), 7.36 (dd, *J* = 7.6, 0.7 Hz, 1 H), 7.24 (d, *J* = 7.7 Hz, 3 H), 7.17 (t, *J* = 7.8 Hz, 1 H), 2.34 (s, 3 H). ^13^C-NMR (126 MHz, CDCl_3_): *δ* (ppm) 146.79, 146.69, 141.80, 139.32, 133.97, 130.49, 130.05, 129.85, 126.92, 126.66, 125.43, 123.06, 122.99, 121.93, 118.83, 118.25, 113.31, 112.84, 21.78. ESI-HRMS: m/z: found 389.10600 [M + H]^+^ (calcd for [C_21_H_17_N_4_O_2_S]^+^ 389.10722).

**ethyl 4-((1-tosyl-1*H*-benzo[*d*]imidazol-2-yl)amino)benzoate**^17^ (**6k’**): white solid. ^1^H-NMR (500 MHz, CDCl_3_): *δ* (ppm) 8.92 (s, 1 H), 8.10–8.06 (m, 2 H), 7.87–7.83 (m, 2 H), 7.76 (d, *J* = 8.5 Hz, 3 H), 7.46 (d, *J* = 7.7 Hz, 1 H), 7.26–7.19 (m, 3 H), 7.15 (td, *J* = 8.0, 1.1 Hz, 1 H), 4.36 (q, *J* = 7.1 Hz, 2 H), 2.30 (s, 3 H), 1.39 (t, *J* = 7.1 Hz, 3 H). ^13^C-NMR (126 MHz, CDCl_3_): *δ* (ppm) 166.25, 146.79, 146.53, 142.48, 141.99, 133.99, 131.11, 130.41, 129.83, 126.89, 125.31, 124.87, 122.86 (s), 118.17, 117.98, 112.85, 60.86, 21.71, 14.45. ESI-HRMS: m/z: found 436.13067 [M + H]^+^ (calcd for [C_23_H_22_N_3_O_4_S]^+^ 436.13310).

**1-(4-((1-tosyl-1*H*-benzo[*d*]imidazol-2-yl)amino)phenyl)ethan-1-one** (**6l’**): yellow solid. melting point: 145–147 ^o^C. ^1^H-NMR (500 MHz, CDCl_3_): *δ* (ppm) 8.94 (s, 1 H), 7.99 (d, *J* = 8.7 Hz, 2 H), 7.86 (d, *J* = 8.8 Hz, 2 H), 7.78–7.74 (m, 3 H), 7.45 (d, *J* = 7.7 Hz, 1 H), 7.25–7.15 (m, 4 H), 2.57 (s, 3 H), 2.30 (s, 3 H). ^13^C-NMR (126 MHz, CDCl_3_): *δ* (ppm) 196.88, 146.64, 146.57, 142.71, 141.88, 133.91, 131.87, 130.41, 130.09, 129.79, 126.87, 125.32, 122.93, 118.18, 118.06, 112.84, 26.49, 21.70. ESI-HRMS: m/z: found 406.12086 [M + H]^+^ (calcd for [C_22_H_20_N_3_O_3_S]^+^ 406.12254).

***N*-(4-isothiocyanatophenyl)-1-tosyl-1*H*-benzo[*d*]imidazol-2-amine** (**6m’**): white solid. melting point: 139–142 ^o^C. ^1^H-NMR (500 MHz, CDCl_3_): *δ* (ppm) 8.78 (s, 1 H), 7.78–7.73 (m, 5 H), 7.47–7.42 (m, 1 H), 7.24 (ddd, *J* = 8.6, 7.0, 5.3 Hz, 5 H), 7.17–7.14 (m, 1 H), 2.34 (s, 3 H). ^13^C-NMR (126 MHz, CDCl_3_): *δ* (ppm) 147.10, 146.71, 141.63, 137.42, 134.63, 134.02, 130.53, 129.83, 126.99, 126.86, 125.94, 125.47, 122.93, 119.99, 118.02, 112.90, 21.83. ESI-HRMS: m/z: found 421.07686 [M + H]^+^ (calcd for [C_21_H_17_N_4_O_2_S_2_]^+^ 421.07930).

***N***^1^,***N***^4^**-bis(1-tosyl-1*H*-benzo[*d*]imidazol-2-yl)benzene-1**,**4-diamine** (**6n’**): white solid. melting point: 235–237 ^o^C. ^1^H-NMR (500 MHz, CDCl_3_): *δ* (ppm) 7.82 (d, *J* = 8.4 Hz, 4 H), 7.78 (dd, *J* = 8.1, 0.5 Hz, 2 H), 7.75 (s, 4 H), 7.44 (dd, *J* = 7.8, 0.5 Hz, 2 H), 7.29–7.26 (m, 4 H), 7.23 (td, *J* = 7.7, 1.2 Hz, 2 H), 7.17–7.13 (m, 2 H), 2.37 (s, 6 H). ^13^C-NMR (126 MHz, CDCl_3_): *δ* (ppm) 148.16, 146.61, 134.25, 134.12, 130.52, 129.97, 127.07, 125.39, 122.55, 121.09, 117.64, 112.93, 21.84. ESI-HRMS: m/z: found 649.16883 [M + H]^+^ (calcd for [C_21_H_17_N_4_O_2_S_2_]^+^ 649.16917).

***N*-(naphthalen-1-yl)-1-tosyl-1*H*-benzo[*d*]imidazol-2-amine**^17^ (**6o’**): colorless oil. ^1^H-NMR (500 MHz, CDCl_3_): *δ* (ppm) 9.40 (s, 1 H), 8.58 (d, *J* = 7.0 Hz, 1 H), 8.12 (d, *J* = 8.4 Hz, 1 H), 7.94 (d, *J* = 7.9 Hz, 1 H), 7.90–7.83 (m, 3 H), 7.68 (d, *J* = 8.2 Hz, 1 H), 7.64 (ddd, *J* = 8.3, 6.8, 1.3 Hz, 1 H), 7.60–7.55 (m, 2 H), 7.49 (dd, *J* = 4.4, 3.9 Hz, 1 H), 7.26 (td, *J* = 7.6, 1.1 Hz, 1 H), 7.22 (d, *J* = 8.2 Hz, 2 H), 7.19 (td, *J* = 7.8, 1.1 Hz, 1 H), 2.33 (s, 3 H). ^13^C-NMR (126 MHz, CDCl_3_): *δ* (ppm) 148.44, 146.42, 142.37, 134.28, 134.23, 133.56, 130.40, 130.14, 129.09, 126.93, 126.63, 126.27, 126.18, 125.69, 125.24, 124.12, 122.45, 120.04, 118.01, 116.69, 112.80, 21.72. ESI-HRMS: m/z: found 414.12786 [M + H]^+^ (calcd for [C_24_H_20_N_3_O_2_S]^+^ 414.12762).

***N*-(pyren-1-yl)-1-tosyl-1*H*-benzo[*d*]imidazol-2-amine** (**6p’**): yellow solid. melting point: 216–218 ^o^C. ^1^H-NMR (500 MHz, CDCl_3_): *δ* (ppm) 9.54 (s, 1 H), 8.98 (d, *J* = 8.4 Hz, 1 H), 8.25 (t, *J* = 8.3 Hz, 2 H), 8.20–8.16 (m, 3 H), 8.03 (dt, *J* = 9.3, 8.1 Hz, 3 H), 7.92 (d, *J* = 8.5 Hz, 2 H), 7.85 (d, *J* = 7.9 Hz, 1 H), 7.47 (d, *J* = 7.5 Hz, 1 H), 7.26 (dd, *J* = 11.9, 4.6 Hz, 3 H), 7.19 (td, *J* = 7.9, 1.1 Hz, 1 H), 2.34 (s, 3 H). ^13^C-NMR (126 MHz, CDCl_3_): *δ* (ppm) 148.83, 146.54, 142.37, 134.34, 131.78, 131.68, 131.05, 130.50, 130.39, 128.33, 128.17, 127.56, 127.06, 126.44, 126.37, 125.84, 125.50, 125.47, 125.33, 125.05, 124.99, 122.49, 121.77, 119.67, 119.25, 118.05, 112.86, 21.81. ESI-HRMS: m/z: found 488.14457 [M + H]^+^ (calcd for [C_30_H_22_N_3_O_2_S]^+^ 488.14327).

***N*-(pyridin-3-yl)-1-tosyl-1*H*-benzo[*d*]imidazol-2-amine**^17^ (**6q’**): light brown solid. ^1^H-NMR (500 MHz, CDCl_3_): *δ* (ppm) 8.78 (d, *J* = 2.6 Hz, 1 H), 8.71 (s, 1 H), 8.44 (ddd, *J* = 8.4, 2.7, 1.4 Hz, 1 H), 8.34 (dd, *J* = 4.7, 1.4 Hz, 1 H), 7.80–7.73 (m, 3 H), 7.42 (d, *J* = 7.8 Hz, 1 H), 7.33 (dd, *J* = 8.4, 4.7 Hz, 1 H), 7.23 (ddd, *J* = 14.2, 5.5, 3.8 Hz, 3 H), 7.15 (td, *J* = 7.9, 1.2 Hz, 1 H), 2.33 (s, 3 H). ^13^C-NMR (126 MHz, CDCl_3_): *δ* (ppm) 147.29, 146.63, 144.39, 141.97, 140.89, 135.52, 134.09, 130.49, 130.07, 126.97, 126.10, 125.37, 123.93, 122.83, 118.12, 112.87, 21.79. ESI-HRMS: m/z: found 365.10595 [M + H]^+^ (calcd for [C_19_H_17_N_4_O_2_S]^+^ 365.10722).

***N*-benzyl-1-tosyl-1*H*-benzo[*d*]imidazol-2-amine**^17^ (**6r’**): white solid. ^1^H-NMR (500 MHz, CDCl_3_): *δ* (ppm) 7.74 (dd, *J* = 11.6, 4.4 Hz, 3 H), 7.41–7.31 (m, 6 H), 7.23–7.17 (m, 3 H), 7.10–7.06 (m, 1 H), 6.72 (t, *J* = 5.5 Hz, 1 H), 4.75 (d, *J* = 5.7 Hz, 2 H), 2.36 (s, 3 H). ^13^C-NMR (126 MHz, CDCl_3_): *δ* (ppm) 152.09, 146.10, 142.64, 137.94, 134.31, 130.97, 130.19, 128.87, 127.83, 127.80, 126.97, 125.00, 121.47, 116.97, 112.59, 47.26, 21.73. ESI-HRMS: m/z: found 378.12755 [M + H]^+^ (calcd for [C_21_H_20_N_3_O_2_S]^+^ 378.127623).

***N*-phenethyl-1-tosyl-1*H*-benzo[*d*]imidazol-2-amine**^17^ (**6s’**): white solid. ^1^H-NMR (500 MHz, CDCl_3_): *δ* (ppm) 7.70–7.66 (m, 1 H), 7.53–7.50 (m, 2 H), 7.40–7.36 (m, 2 H), 7.33–7.29 (m, 4 H), 7.18–7.13 (m, 3 H), 7.06–7.02 (m, 1 H), 6.40 (t, *J* = 5.1 Hz, 1 H), 3.82 (td, *J* = 6.7, 5.4 Hz, 2 H), 3.05 (t, *J* = 6.7 Hz, 2 H), 2.34 (s, 3 H). ^13^C-NMR (126 MHz, CDCl_3_): *δ* (ppm) 152.00, 145.96, 142.78, 138.79, 134.35, 130.84, 130.17, 129.0, 128.91, 126.94, 126.83, 124.94, 121.34, 116.86, 112.52, 44.34, 35.28, 21.77. ESI-HRMS: m/z: found 392.14251 [M + H]^+^ (calcd for [C_22_H_22_N_3_O_2_S]^+^ 392.14327).

***N*-(furan-2-ylmethyl)-1-tosyl-1*H*-benzo[*d*]imidazol-2-amine** (**6t’**): yellow solid. melting point: 125–126 ^o^C. ^1^H-NMR (500 MHz, CDCl_3_): *δ* (ppm) 7.72 (dd, *J* = 12.1, 8.3 Hz, 3 H), 7.42 (dd, *J* = 1.7, 0.7 Hz, 1 H), 7.36–7.32 (m, 1 H), 7.23–7.20 (m, 2 H), 7.17 (td, *J* = 7.7, 1.1 Hz, 1 H), 7.06 (td, *J* = 8.0, 1.1 Hz, 1 H), 6.76 (t, *J* = 5.4 Hz, 1 H), 6.35 (dt, *J* = 8.7, 2.5 Hz, 2 H), 4.74 (d, *J* = 5.6 Hz, 2 H), 2.35 (s, 3 H). ^13^C-NMR (126 MHz, CDCl_3_): *δ* (ppm) 151.71, 151.04, 146.16, 142.54, 142.36, 134.24, 130.85, 130.22, 127.02, 125.06, 121.63, 117.01, 112.66, 110.64, 108.06, 40.25, 21.76. ESI-HRMS: m/z: found 368.10678 [M + H]^+^ (calcd for [C_19_H_18_N_3_O_3_S]^+^ 368.10689).

***N*-ethyl-1-tosyl-1*H*-benzo[*d*]imidazol-2-amine**^17^ (**6u’**): pale yellow solid. ^1^H-NMR (500 MHz, CDCl_3_): *δ* (ppm) 7.76 (d, *J* = 8.4 Hz, 2 H), 7.71–7.66 (m, 1 H), 7.32–7.28 (m, 1 H), 7.22 (dd, *J* = 8.6, 0.6 Hz, 2 H), 7.14 (td, *J* = 7.7, 1.2 Hz, 1 H), 7.05–7.00 (m, 1 H), 6.39 (t, *J* = 5.4 Hz, 1 H), 3.57 (qd, *J* = 7.2, 5.6 Hz, 2 H), 2.32 (s, 3 H), 1.33 (t, *J* = 7.2 Hz, 3 H). ^13^C-NMR (126 MHz, CDCl_3_): *δ* (ppm) 152.11, 146.04, 142.80, 134.36, 130.74, 130.16, 126.87, 124.89, 121.18, 116.70, 112.49, 38.17, 21.68, 14.98. ESI-HRMS: m/z: found 316.11203 [M + H]^+^ (calcd for [C_16_H_18_N_3_O_2_S]^+^ 316.11197).

***N*-isopropyl-1-tosyl-1*H*-benzo[*d*]imidazol-2-amine**^17^ (**6v’**): white solid. ^1^H-NMR (500 MHz, CDCl_3_): *δ* (ppm) 7.75 (d, *J* = 8.4 Hz, 2 H), 7.72–7.68 (m, 1 H), 7.30 (dd, *J* = 7.7, 0.4 Hz, 1 H), 7.23 (d, *J* = 8.2 Hz, 2 H), 7.14 (td, *J* = 7.7, 1.1 Hz, 1 H), 7.03 (td, *J* = 7.8, 1.1 Hz, 1 H), 6.28 (d, *J* = 7.8 Hz, 1 H), 4.24–4.14 (m, 1 H), 2.34 (s, 3 H), 1.34 (d, *J* = 6.5 Hz, 6 H). ^13^C-NMR (126 MHz, CDCl_3_): *δ* (ppm) 151.46, 146.05, 142.99, 134.38, 130.66, 130.16, 126.88, 124.94, 121.14, 116.65, 112.60, 45.21, 23.03, 21.72. ESI-HRMS: m/z: found 330.12453 [M + H]^+^ (calcd for [C_17_H_20_N_3_O_2_S]^+^ 330.12762).

***N*-octyl-1-tosyl-1*H*-benzo[*d*]imidazol-2-amine** (**6w’**): pale yellow oil. ^1^H-NMR (500 MHz): *δ* (ppm) 7.76 (d, *J* = 8.4 Hz, 2 H), 7.69 (d, *J* = 8.0 Hz, 1 H), 7.33 (d, *J* = 7.8 Hz, 1 H), 7.24 (d, *J* = 8.1 Hz, 2 H), 7.16 (td, *J* = 7.7, 1.0 Hz, 1 H), 7.04 (td, *J* = 8.1, 1.0 Hz, 1 H), 6.44 (t, *J* = 5.1 Hz, 1 H), 3.54 (q, *J* = 12.7, 7.0 Hz, 2 H), 2.36 (s, 3 H), 1.74–1.68 (m, 2 H), 1.43–1.27 (m, 10 H), 0.89 (t, *J* = 7.0 Hz, 3 H). ^13^C-NMR (126 MHz): *δ* (ppm) 152.13, 146.21, 142.26, 134.41, 130.65, 130.26, 126.96, 125.05, 121.40, 116.68, 112.55, 43.54, 31.92, 29.50, 29.38, 27.03, 22.78, 21.77, 14.23. ESI-HRMS: m/z: found 400.20521 [M + H]^+^ (calcd for [C_23_H_30_N_3_O_2_S]^+^ 400.20587).

***N*-cyclohexyl-1-tosyl-1*H*-benzo[*d*]imidazol-2-amine**^17^ (**6x’**): white solid. ^1^H-NMR (500 MHz, CDCl_3_): *δ* (ppm) 7.75 (d, *J* = 8.4 Hz, 2 H), 7.70 (d, *J* = 7.9 Hz, 1 H), 7.29 (d, *J* = 7.8 Hz, 1 H), 7.23 (d, *J* = 8.4 Hz, 2 H), 7.14 (td, *J* = 7.7, 1.1 Hz, 1 H), 7.02 (td, *J* = 7.8, 1.1 Hz, 1 H), 6.38 (d, *J* = 8.1 Hz, 1 H), 3.95–3.85 (m, 1 H), 2.34 (s, 3 H), 2.14–2.07 (m, 2 H), 1.80–1.73 (m, 2 H), 1.68–1.61 (m, 1 H), 1.50–1.24 (m, 5 H). ^13^C-NMR (126 MHz, CDCl_3_): *δ* (ppm) 151.50, 146.04, 143.04, 134.40, 130.69, 130.16, 126.90, 124.95, 121.06, 116.60, 112.60, 51.65, 33.27, 25.67, 24.75, 21.74. ESI-HRMS: m/z: found 370.15853 [M + H]^+^ (calcd for [C_20_H_24_N_3_O_2_S]^+^ 370.15892).

***N*-((3*s***,**5*s***,**7*s*)-adamantan-1-yl)-1-tosyl-1*H*-benzo[*d*]imidazol-2-amine** (**6y’**): white solid. melting point: 166–169 ^o^C. ^1^H-NMR (500 MHz): *δ* (ppm) 7.74 (d, *J* = 8.4 Hz, 2 H), 7.70 (d, *J* = 8.0 Hz, 1 H), 7.33–7.27 (m, 1 H), 7.25 (d, *J* = 8.4 Hz, 2 H), 7.14 (t, *J* = 7.4 Hz, 1 H), 7.02 (t, *J* = 7.7 Hz, 1 H), 6.48 (s, 1 H), 2.36 (s, 3 H), 2.15 (s, 9 H), 1.78–1.70 (m, 6 H). ^13^C-NMR (126 MHz): *δ* (ppm) 149.72, 146.00, 134.53, 130.15, 129.98, 126.92, 124.91, 121.15, 116.79, 112.74, 53.15, 41.82, 36.40, 29.68, 21.78. ESI-HRMS: m/z: found 422.19117 [M + H]^+^ (calcd for [C_25_H_27_N_3_O_2_S]^+^ 422.19022).

**5**,**6-dichloro-*N*-(4-chlorophenyl)-1-tosyl-1*H*-benzo[*d*]imidazol-2-amine** (**6a’’**): pale brown solid. melting point: 190–193 ^o^C. ^1^H-NMR (500 MHz, CDCl_3_): *δ* (ppm) 8.63 (s, 1 H), 7.83 (s, 1 H), 7.76 (d, *J* = 8.4 Hz, 2 H), 7.69–7.65 (m, 2 H), 7.46 (s, 1 H), 7.37–7.34 (m, 2 H), 7.29 (d, *J* = 8.3 Hz, 2 H), 2.38 (s, 3 H). ^13^C-NMR (126 MHz, CDCl_3_): *δ* (ppm) 148.74, 147.16, 141.87, 136.51, 133.59, 130.73, 129.41, 129.24, 129.00, 126.97, 125.99, 120.67, 119.10, 114.19, 21.87. ESI-HRMS: m/z: found 465.99157 [M + H]^+^ (calcd for [C_20_H_15_Cl_3_N_3_O_2_S]^+^ 465.99506).

***N*-(4-chlorophenyl)-5**,**6-dimethyl-1-tosyl-1*H*-benzo[*d*]imidazol-2-amine** (**6b’’**): pale brown solid. melting point: 203–206 ^o^C. ^1^H-NMR (500 MHz, CDCl_3_): *δ* (ppm) 8.61 (s, 1 H), 7.76 (d, *J* = 8.4 Hz, 2 H), 7.70 (d, *J* = 8.8 Hz, 2 H), 7.53 (s, 1 H), 7.34 (d, *J* = 8.8 Hz, 2 H), 7.25–7.21 (m, 3 H), 2.35 (s, 3 H), 2.33 (s, 3 H), 2.27 (s, 3 H). ^13^C-NMR (126 MHz, CDCl_3_): *δ* (ppm) 147.02, 146.33, 140.16, 137.23, 134.28, 133.97, 131.40, 130.42, 129.30, 128.14, 126.88, 120.30, 118.58, 113.54, 21.79, 20.50, 20.22. ESI-HRMS: m/z: found 426.10251 [M + H]^+^ (calcd for [C_22_H_21_ClN_3_O_2_S]^+^ 426.10430).

***N*-(4-chlorophenyl)-1-tosyl-1*H*-naphtho[2**,**3-*d*]imidazol-2-amine** (**6c’’**): pale yellow solid. melting point: 223–225 ^o^C. ^1^H-NMR (500 MHz, CDCl_3_): *δ* (ppm) 8.83 (s, 1 H), 8.17 (s, 1 H), 7.94–7.91 (m, 1 H), 7.86–7.83 (m, 1 H), 7.82–7.76 (m, 5 H), 7.45–7.41 (m, 2 H), 7.41–7.36 (m, 2 H), 7.21 (d, *J* = 8.2 Hz, 2 H), 2.31 (s, 3 H). ^13^C-NMR (126 MHz, CDCl_3_): *δ* (ppm) 149.23, 146.59, 141.82, 136.74, 133.86, 132.20, 130.48, 130.18, 129.41, 128.93, 128.17, 127.81, 126.96, 125.26, 124.62, 120.84, 114.29, 110.21, 21.77. ESI-HRMS: m/z: found 448.08584 [M + H]^+^ (calcd for [C_24_H_19_ClN_3_O_2_S]^+^ 448.08865).

***N*-(4-chlorophenyl)-1-((4-fluorophenyl)sulfonyl)-1*H*-benzo[*d*]imidazol-2-amine** (**6d’’**): white solid. melting point: 140–142 ^o^C. ^1^H-NMR (500 MHz, CDCl_3_): *δ* (ppm) 8.61 (s, 1 H), 7.92 (ddd, *J* = 10.0, 5.0, 2.6 Hz, 2 H), 7.76–7.71 (m, 3 H), 7.46–7.43 (m, 1 H), 7.38–7.34 (m, 2 H), 7.25 (td, *J* = 7.7, 1.2 Hz, 1 H), 7.18–7.11 (m, 3 H). ^13^C-NMR (126 MHz, CDCl_3_): *δ* (ppm) 167.43, 165.37, 147.36, 142.22, 136.92, 133.05, 133.03, 129.92, 129.84, 129.78, 129.33, 128.49, 125.59, 122.77, 120.44, 118.15, 117.40, 117.22, 112.77. ^19^F-NMR (471 MHz, CDCl_3_): *δ* (ppm) -104.27. ESI-HRMS: m/z: found 402.04541 [M + H]^+^ (calcd for [C_19_H_14_ClFN_3_O_2_S]^+^ 402.04793).

***N*-(4-chlorophenyl)-1-((4-chlorophenyl)sulfonyl)-1*H*-benzo[*d*]imidazol-2-amine** (**6e’’**): white solid. melting point: 146–148 ^o^C. ^1^H-NMR (500 MHz, CDCl_3_): *δ* (ppm) 8.59 (s, 1 H), 7.83–7.80 (m, 2 H), 7.75–7.70 (m, 3 H), 7.46–7.43 (m, 1 H), 7.42–7.39 (m, 2 H), 7.37–7.34 (m, 2 H), 7.25 (td, *J* = 7.7, 1.1 Hz, 1 H), 7.16 (td, *J* = 8.1, 1.1 Hz, 1 H). ^13^C-NMR (126 MHz, CDCl_3_): *δ* (ppm) 147.30, 142.20, 141.93, 136.88, 135.35, 130.16, 129.72, 129.31, 128.51, 128.25, 125.63, 122.79, 120.45, 118.17, 112.76. ESI-HRMS: m/z: found 418.01676 [M + H]^+^ (calcd for [C_19_H_14_Cl_2_N_3_O_2_S]^+^ 418.01838).

**1-((4-bromophenyl)sulfonyl)-*N*-(4-chlorophenyl)-1*H*-benzo[*d*]imidazol-2-amine** (**6f’’**): yellow solid. melting point: 166–168 ^o^C. ^1^H-NMR (500 MHz, CDCl_3_): *δ* (ppm) 8.58 (s, 1 H), 7.73 (dt, *J* = 10.8, 5.5 Hz, 5 H), 7.60–7.56 (m, 2 H), 7.44 (d, *J* = 7.9 Hz, 1 H), 7.38–7.34 (m, 2 H), 7.25 (td, *J* = 7.8, 1.0 Hz, 1 H), 7.18–7.14 (m, 1 H). ^13^C-NMR (126 MHz, CDCl_3_): *δ* (ppm) 147.33, 142.23, 136.89, 135.92, 133.19, 130.64, 129.74, 129.35, 128.56, 128.25, 125.68, 122.83, 120.48, 118.21, 112.79. ESI-HRMS: m/z: found 461.96468 [M + H]^+^ (calcd for [C_19_H_14_BrClN_3_O_2_S]^+^ 461.96786).

***N*-(4-chlorophenyl)-1-(phenylsulfonyl)-1*H*-benzo[*d*]imidazol-2-amine** (**6g’’**): colorless oil. melting point: 119–122 ^o^C. ^1^H-NMR (500 MHz, CDCl_3_): *δ* (ppm) 8.66 (s, 1 H), 7.90 (dt, *J* = 8.7, 1.6 Hz, 2 H), 7.77 (d, *J* = 7.8 Hz, 1 H), 7.74–7.70 (m, 2 H), 7.60–7.56 (m, 1 H), 7.47–7.42 (m, 3 H), 7.37–7.33 (m, 2 H), 7.25–7.21 (m, 1 H), 7.15 (td, *J* = 7.8, 1.2 Hz, 1 H). ^13^C-NMR (126 MHz, CDCl_3_): *δ* (ppm) 147.47, 142.16, 137.10, 137.03, 135.05, 129.93, 129.82, 129.30, 128.38, 126.88, 125.40, 122.67, 120.44, 118.02, 112.82. ESI-HRMS: m/z: found 384.05537 [M + H]^+^ (calcd for [C_19_H_15_ClN_3_O_2_S]^+^ 384.05735).

***N*-(4-chlorophenyl)-1-((4-propylphenyl)sulfonyl)-1*H*-benzo[*d*]imidazol-2-amine** (**6h’’**): white solid. melting point: 147–149 ^o^C. ^1^H-NMR (500 MHz, CDCl_3_): *δ* (ppm) 8.70 (s, 1 H), 7.79 (dd, *J* = 11.4, 8.4 Hz, 3 H), 7.74 (t, *J* = 5.7 Hz, 2 H), 7.44 (d, *J* = 7.8 Hz, 1 H), 7.35 (t, *J* = 5.6 Hz, 2 H), 7.26–7.21 (m, 3 H), 7.15 (t, *J* = 7.8 Hz, 1 H), 2.58–2.52 (m, 2 H), 1.60–1.51 (m, 2 H), 0.88 (t, *J* = 7.3 Hz, 3 H). ^13^C-NMR (126 MHz, CDCl_3_): *δ* (ppm) 151.04, 147.49, 142.13, 137.10, 134.29, 129.99, 129.80, 129.26, 128.26, 126.94, 125.24, 122.57, 120.40, 117.92, 112.82, 37.94, 23.96, 13.78. ESI-HRMS: m/z: found 426.10202 [M + H]^+^ (calcd for [C_22_H_21_ClN_3_O_2_S]^+^ 426.10430).

**1-((4-(*tert*-butyl)phenyl)sulfonyl)-*N*-(4-chlorophenyl)-1*H*-benzo[*d*]imidazol-2-amine** (**6i’’**): white solid. melting point: 184–187 ^o^C. ^1^H-NMR (500 MHz, CDCl_3_): *δ* (ppm) 8.71 (s, 1 H), 7.84–7.81 (m, 2 H), 7.81–7.79 (m, 1 H), 7.75–7.71 (m, 2 H), 7.46 (dd, *J* = 11.4, 4.6 Hz, 3 H), 7.37–7.34 (m, 2 H), 7.26–7.23 (m, 1 H), 7.17 (td, *J* = 8.0, 1.1 Hz, 1 H), 1.26 (s, 9 H). ^13^C-NMR (126 MHz, CDCl_3_): *δ* (ppm) 159.35, 147.54, 142.05, 137.08, 134.12, 130.06, 129.36, 128.43, 126.96, 126.85, 125.31, 122.68, 120.51, 117.94, 112.90, 35.51, 30.96. ESI-HRMS: m/z: found 440.11800 [M + H]^+^ (calcd for [C_23_H_23_ClN_3_O_2_S]^+^ 440.11995).

***N*-(4-chlorophenyl)-1-((4-methoxyphenyl)sulfonyl)-1*H*-benzo[*d*]imidazol-2-amine** (**6j’’**): white solid. melting point: 152–154 ^o^C. ^1^H-NMR (500 MHz, CDCl_3_): *δ* (ppm) 8.70 (s, 1 H), 7.85–7.81 (m, 2 H), 7.76 (d, *J* = 7.9 Hz, 1 H), 7.75–7.71 (m, 2 H), 7.44 (d, *J* = 7.7 Hz, 1 H), 7.37–7.33 (m, 2 H), 7.23 (td, *J* = 7.7, 1.1 Hz, 1 H), 7.15 (td, *J* = 8.0, 1.1 Hz, 1 H), 6.89–6.85 (m, 2 H), 3.76 (s, 3 H). ^13^C-NMR (126 MHz, CDCl_3_): *δ* (ppm) 164.72, 147.55, 142.16, 137.13, 129.97, 129.27, 128.31, 128.26, 125.22, 122.54, 120.40, 117.92, 114.97, 112.82, 55.82. ESI-HRMS: m/z: found 414.06571 [M + H]^+^ (calcd for [C_20_H_17_ClN_3_O_3_S]^+^ 414.06792).

***N*-(4-chlorophenyl)-1-((4-(trifluoromethyl)phenyl)sulfonyl)-1*H*-benzo[*d*]imidazol-2-amine** (**6k’’**): pale yellow solid. melting point: 163–165 ^o^C. ^1^H-NMR (500 MHz, CDCl_3_): *δ* (ppm) 8.56 (s, 1 H), 8.02 (d, *J* = 8.3 Hz, 2 H), 7.77–7.70 (m, 5 H), 7.45 (d, *J* = 7.7 Hz, 1 H), 7.38–7.35 (m, 2 H), 7.28–7.25 (m, 1 H), 7.18 (td, *J* = 8.1, 1.1 Hz, 1 H). ^13^C-NMR (126 MHz, CDCl_3_): *δ* (ppm) 147.27, 142.27, 140.48, 136.82, 136.52 (q, *J* = 33.02 Hz), 129.71, 129.42, 128.76, 127.52, 127.07 (q, *J* = 3.57 Hz), 125.91, 123.00, 122.76 (d, *J* = 273.78 Hz), 120.54, 118.37, 112.81^19^. F-NMR (471 MHz): *δ* (ppm) -63.36. ESI-HRMS: m/z: found 452.04249 [M + H]^+^ (calcd for [C_20_H_14_ClF_3_N_3_O_2_S]^+^ 452.04474).

***N*-(4-chlorophenyl)-1-((4-nitrophenyl)sulfonyl)-1*H*-benzo[*d*]imidazol-2-amine** (**6l’’**): orange solid. melting point: 184–187 ^o^C. ^1^H-NMR (500 MHz, CDCl_3_): *δ* (ppm) 8.50 (s, 1 H), 8.29 (d, *J* = 8.9 Hz, 2 H), 8.07 (d, *J* = 8.9 Hz, 2 H), 7.74 (d, *J* = 8.0 Hz, 1 H), 7.70 (d, *J* = 8.9 Hz, 2 H), 7.44 (d, *J* = 7.9 Hz, 1 H), 7.36 (d, *J* = 8.9 Hz, 2 H), 7.28–7.25 (m, 1 H), 7.18 (td, *J* = 7.9, 1.1 Hz, 1 H). ^13^C-NMR (126 MHz, CDCl_3_): *δ* (ppm) 151.34, 147.16, 142.31, 136.69, 129.57, 129.46, 128.93, 128.33, 126.17, 125.07, 123.16, 120.56, 118.53, 112.81. ESI-HRMS: m/z: found 429.04085 [M + H]^+^ (calcd for [C_19_H_14_ClN_4_O_4_S]^+^ 429.04243.

***N*-(4-chlorophenyl)-1-(naphthalen-2-ylsulfonyl)-1*H*-benzo[*d*]imidazol-2-amine** (**6m’’**): pale yellow solid. melting point: 175–178 ^o^C. ^1^H-NMR (500 MHz, CDCl_3_): *δ* (ppm) 8.77 (s, 1 H), 8.54 (s, 1 H), 7.93 (d, *J* = 8.1 Hz, 1 H), 7.86 (d, *J* = 8.8 Hz, 1 H), 7.82 (d, *J* = 8.0 Hz, 2 H), 7.78–7.74 (m, 3 H), 7.62 (ddd, *J* = 21.1, 7.9, 6.9 Hz, 2 H), 7.43 (d, *J* = 7.7 Hz, 1 H), 7.37 (dd, *J* = 8.7, 0.8 Hz, 2 H), 7.22 (t, *J* = 7.7 Hz, 1 H), 7.15 (t, *J* = 7.8 Hz, 1 H). ^13^C-NMR (126 MHz, CDCl_3_): *δ* (ppm) 147.56, 142.09, 137.08, 135.71, 133.83, 131.81, 130.47, 130.13, 129.95, 129.70, 129.35, 129.25, 128.44, 128.30, 128.09, 125.38, 122.68, 120.92, 120.54, 118.03, 112.81. ESI-HRMS: m/z: found 434.07158 [M + H]^+^ (calcd for [C_23_H_17_ClN_3_O_2_S]^+^ 434.07300).

***N*-(4-chlorophenyl)-1-(thiophen-2-ylsulfonyl)-1*H*-benzo[*d*]imidazol-2-amine** (**6n’’**): white solid. melting point: 138–141 ^o^C. ^1^H-NMR (500 MHz, CDCl_3_): *δ* (ppm) 8.55 (s, 1 H), 7.81 (d, *J* = 7.9 Hz, 1 H), 7.73–7.69 (m, 3 H), 7.56 (dd, *J* = 5.0, 1.3 Hz, 1 H), 7.45 (d, *J* = 7.6 Hz, 1 H), 7.36–7.32 (m, 2 H), 7.26 (dt, *J* = 7.7, 3.8 Hz, 1 H), 7.18 (td, *J* = 8.0, 1.1 Hz, 1 H), 6.99 (dd, *J* = 5.0, 4.0 Hz, 1 H). ^13^C-NMR (126 MHz, CDCl_3_): *δ* (ppm) 147.24, 142.33, 136.91, 136.28, 135.05, 134.07, 129.76, 129.27, 128.47, 128.13, 125.66, 122.71, 120.52, 118.04, 113.14. ESI-HRMS: m/z: found 390.01150 [M + H]^+^ (calcd for [C_17_H_13_ClN_3_O_2_S_2_]^+^ 390.01377).

***N*-(4-chlorophenyl)-1-(methylsulfonyl)-1*H*-benzo[*d*]imidazol-2-amine** (**6o’’**): white solid. melting point: 133–136 ^o^C. ^1^H-NMR (500 MHz, CDCl_3_): *δ* (ppm) 8.41 (s, 1 H), 7.73–7.69 (m, 2 H), 7.66 (d, *J* = 8.0 Hz, 1 H), 7.54 (d, *J* = 7.9 Hz, 1 H), 7.36–7.29 (m, 3 H), 7.20 (td, *J* = 8.1, 1.1 Hz, 1 H), 3.22 (s, 3 H). ^13^C-NMR (126 MHz, CDCl_3_): *δ* (ppm) 147.14, 142.11, 136.87, 130.05, 129.27, 128.40, 125.58, 122.85, 120.40, 118.24, 112.18, 40.81. ESI-HRMS: m/z: found 322.04076 [M + H]^+^ (calcd for [C_14_H_13_ClN_3_O_2_S]^+^ 322.04170).

***tert*-butyl 2-((4-chlorophenyl)amino)-1*H*-benzo[*d*]imidazole-1-carboxylate** (**6p’’**): white solid. melting point: 168–170 ^o^C. ^1^H-NMR (500 MHz, CDCl_3_): *δ* (ppm) 9.72 (s, 1 H), 7.79–7.75 (m, 2 H), 7.63 (s, 1 H), 7.52–7.48 (m, 1 H), 7.33–7.29 (m, 2 H), 7.25 (dd, *J* = 10.9, 4.4 Hz, 1 H), 7.12 (dd, *J* = 15.5, 0.9 Hz, 1 H), 1.75 (s, 9 H). ^13^C-NMR (126 MHz, CDCl_3_): *δ* (ppm) 151.58, 149.65, 142.22, 137.48, 129.65, 129.11, 127.63, 124.75, 121.76, 120.27, 117.62, 114.19, 86.78, 28.26. ESI-HRMS: m/z: found 344.11516 [M + H]^+^ (calcd for [C_18_H_19_ClN_3_O_2_]^+^ 344.11658).

**1-allyl-*N*-(4-chlorophenyl)-1*H*-benzo[*d*]imidazol-2-amine** (**6q’’**): light brown solid. melting point: 191–194 ^o^C. ^1^H-NMR (500 MHz, CDCl_3_): *δ* (ppm) 7.58 (d, *J* = 7.7 Hz, 1 H), 7.47 (d, *J* = 8.8 Hz, 2 H), 7.27–7.25 (m, 2 H), 7.20–7.13 (m, 3 H), 6.38 (s, 1 H), 5.98 (ddt, *J* = 17.1, 10.1, 4.9 Hz, 1 H), 5.34 (d, *J* = 10.4 Hz, 1 H), 5.22–5.17 (m, 1 H), 4.63 (dt, *J* = 4.8, 1.7 Hz, 2 H). ^13^C-NMR (126 MHz, CDCl_3_): *δ* (ppm) 149.66, 141.49, 138.79, 133.69, 131.86, 129.27, 127.32, 122.01, 121.07, 119.71, 118.32, 117.64, 108.02, 45.03. ESI-HRMS: m/z: found 284.09596 [M + H]^+^ (calcd for [C_16_H_15_ClN_3_]^+^ 284.09545).

**1-benzyl-*N*-(4-chlorophenyl)-1*H*-benzo[*d*]imidazol-2-amine** (**6r’’**): light brown solid. melting point: 214–217 ^o^C. ^1^H-NMR (500 MHz, CDCl_3_): *δ* (ppm) 7.55 (d, *J* = 7.8 Hz, 1 H), 7.37–7.31 (m, 5 H), 7.22–7.16 (m, 5 H), 7.09 (d, *J* = 8.8 Hz, 2 H), 5.30 (s, 2 H). ^13^C-NMR (126 MHz, CDCl_3_): *δ* (ppm) 149.71, 139.71, 138.18, 135.05, 133.62, 129.41, 129.16, 128.50, 127.86, 126.84, 122.47, 121.65, 120.56, 116.78, 108.61, 46.43. ESI-HRMS: m/z: found 334.11214 [M + H]^+^ (calcd for [C_20_H_17_ClN_3_]^+^ 334.11110).

**1-(4-bromobenzyl)-*N*-(4-chlorophenyl)-1*H*-benzo[*d*]imidazol-2-amine** (**6s’’**): yellow solid. melting point: 195–197 ^o^C. ^1^H-NMR (500 MHz, acetone-d_6_): *δ* (ppm) 7.93 (d, *J* = 8.9 Hz, 2 H), 7.50–7.46 (m, 3 H), 7.30 (d, *J* = 9.0 Hz, 2 H), 7.17–7.15 (m, 1 H), 7.14–7.09 (m, 3 H), 7.04–7.01 (m, 1 H), 5.48 (s, 2 H). ^13^C-NMR (126 MHz, acetone-d_6_): *δ* (ppm) 150.54, 142.84, 140.85, 137.02, 134.52, 132.55, 129.41, 129.28, 126.41, 122.31, 121.66, 121.24, 120.43, 117.58, 109.25, 45.45. ESI-HRMS: m/z: found 412.02195 [M + H]^+^ (calcd for [C_20_H_15_BrClN_3_]^+^ 412.02161).

## Electronic supplementary material

Below is the link to the electronic supplementary material.

Supplementary Material 1

## Data Availability

All data generated or analyzed during this study are included in this published article [and its supplementary information file].
