# Supplementary material for: A mild and scalable one-pot synthesis of N-substituted 2-aminobenzimidazoles via visible light mediated cyclodesulfurization
Source: Sci Rep. 2025 Feb 3;15:4096. doi: 10.1038/s41598-025-86772-8 (PMC11791095; doi:10.1038/s41598-025-86772-8)
Supplement: Supplementary file 1 — Supplementary Material 1 [file 41598_2025_86772_MOESM1_ESM.pdf]

# A mild and scalable one-pot synthesis of *N*-substituted 2-aminobenzimidazoles *via* visible light mediated cyclodesulfurization

Tanawat Rerkrachaneekorn,[1] Rose Malina Annuur,[1] Soraya Pornsuwan,[2] Mongkol Sukwattanasinitt[1] and Sumrit Wacharasindhu[1,3,*]

[1]Nanotec-CU Center of Excellence on Food and Agriculture, Department of Chemistry, Faculty of Science, Chulalongkorn University, Bangkok 10330, Thailand.

[2]Department of Chemistry and Center of Excellence for Innovation in Chemistry, Faculty of Science, Mahidol University, Bangkok 10400, Thailand.

[3]Green Chemistry for Fine Chemical Productions and Environmental Remediation Research Unit, Department of Chemistry, Faculty of Science, Chulalongkorn University, Bangkok 10330, Thailand.

[*]Correspondence: sumrit.w@chula.ac.th

## Table of Contents

Title page S1

General information S2

Additional optimization reaction S3

Reaction setup S4-S5

Experimental section S6-S54

Copies of $^{1}$H- and $^{13}$C-NMR spectra of **3a, 4**, **5a, 6** S55-S145

Copies of mass spectra of **3a, 5a, 6** S146-S217

References S218-S219

## 1. General information

All chemicals and solvents were obtained from commercially available suppliers such as Eurisotop, Fluka, Merck, Sigma-Aldrich, TCI and Thermo scientific and were used without further purification, unless otherwise stated. The photochemical reactions were performed using 1) 19W LED (Philips LED 19W Durable Brightness Daylight E27), 2) LED strip reactor which made from 1000 mL beaker and linked with a commercial LED stirp (1.5W × 150) and 3) 3W LED which was placed on a glass rod fiber immersed in pyrex schlenk reactor. Analytical thin layer chromatography (TLC) was performed with precoated Merck silica gel 60 F254 plates (0.25 mm for thick layer) and visualized at 254 nm using an ultraviolet lamp. Column chromatography was performed with Silicycle silica gel 60-200 μm (70-230 mesh). $^{1}$H-NMR, $^{13}$C-NMR and $^{19}$F spectra were obtained with JEOL JNM-ECZ500R/S1 NMR spectrometers operating at 500 MHz for $^{1}$H or 126 MHz for $^{13}$C or 471 MHz for $^{19}$F nuclei. Data were reported with chemical shift, multiplicity (br = broad, s = singlet, d = doublet, t = triplet, q = quartet, m = multiplet), coupling constant (Hz), and integration. EPR spectra were collected using an EPR Bruker system, ELEXSYS E-500 model at X-band microwave frequencies (approximately 9.85 GHz). High-resolution mass spectra (HRMS) were recorded using electron spray ionization (ESI) with a MicroTOF Bruker mass spectrometer. Melting point was measured by Stuart, SMP 20. X-ray diffraction (XRD) patterns were obtained on a DMAX 2200/Ultima + diffractometer (Rigaku) using Cu K$\alpha$ radiation source and operating at 40 kV and 30 mA. UV-vis spectra were measured using Agilent Cary 60 UV-vis spectrophotometer.

## 2. Additional optimization reaction

**Table S1.** Additional optimization for synthesis of *N*-tosyl-2-aminobenzimidazole (**6a**)

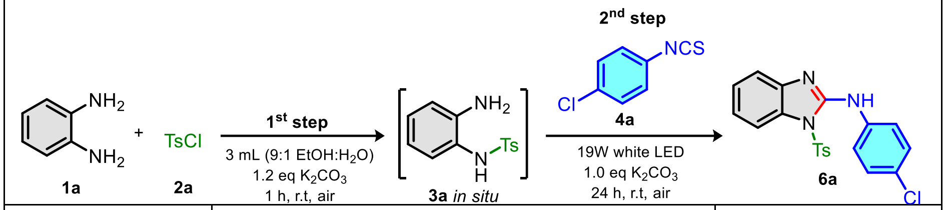


| Entry | Deviations from standard condition[a] | Yield[b] (%) |
|---|---|---|
| 0 | None | 81 |
| 1 | $Na_2CO_3$ as base | 78 |
| 2 | $Cs_2CO_3$ as base | 74 |
| 3 | NaOH as base | 53 |
| 4 | DBU as base | 61 |
| 5 | 3 h[c] | 35 |
| 6 | MeCN, 12 h[c] | 52 |
| 7 | MeOH, 12 h[c] | 50 |
| 8 | DMSO, 12 h[c] | 22 |
| 9 | $CH_2Cl_2$, 12 h[c] | 25 |
| 10 | (7:3 EtOH:$H_2O$), 12 h[c] | 61 |
| 11 | 60W red LED, 12 h[c] | 8 |
| 12 | 60W green LED, 12 h[c] | 53 |
| 13 | 60W blue LED, 12 h[c] | 78 |

[a]General condition: (First step) **1a** (1.2 eq, 0.48 mmol), **2a** (1.2 eq, 0.48 mmol), base (1.2 eq, 0.48 mmol), 3 mL solvent, 1 h under air at room temperature. (Second step) **4a** (1.0 eq, 0.40 mmol), base (1.0 eq, 0.40 mmol), 24 h under air at room temperature. [b]Isolated yield by silica gel column chromatography. [c]Irradiation time for second step was described in each entry.

3. Reaction setup

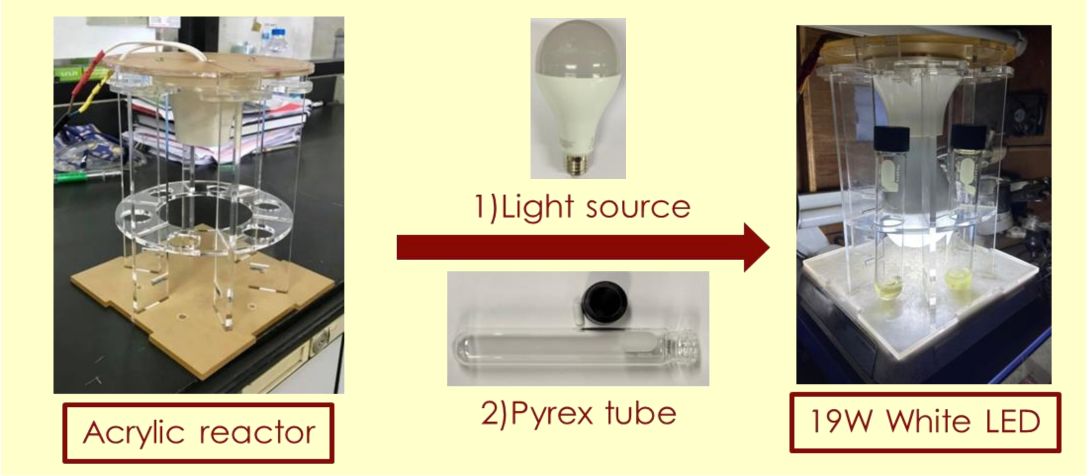


**Figure S1.** Reaction setup using 19W white LED bulb

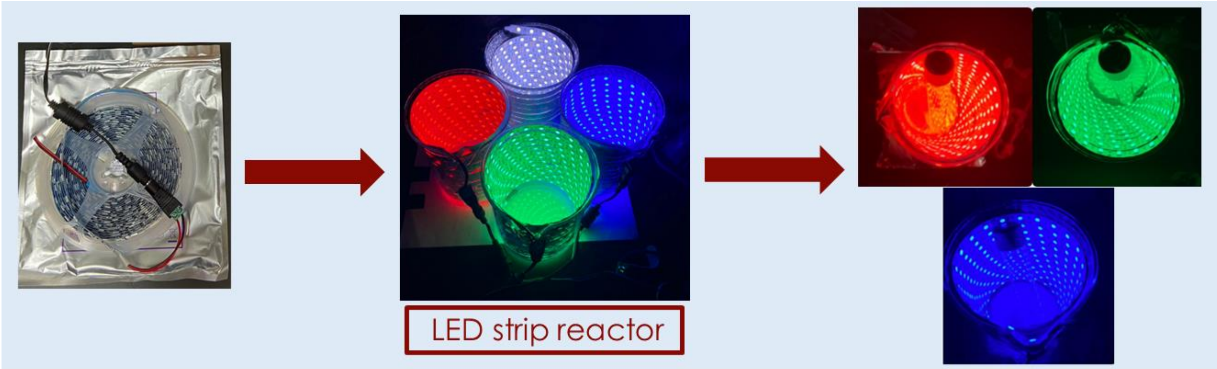


**Figure S2.** Reaction setup using LED strip reactor

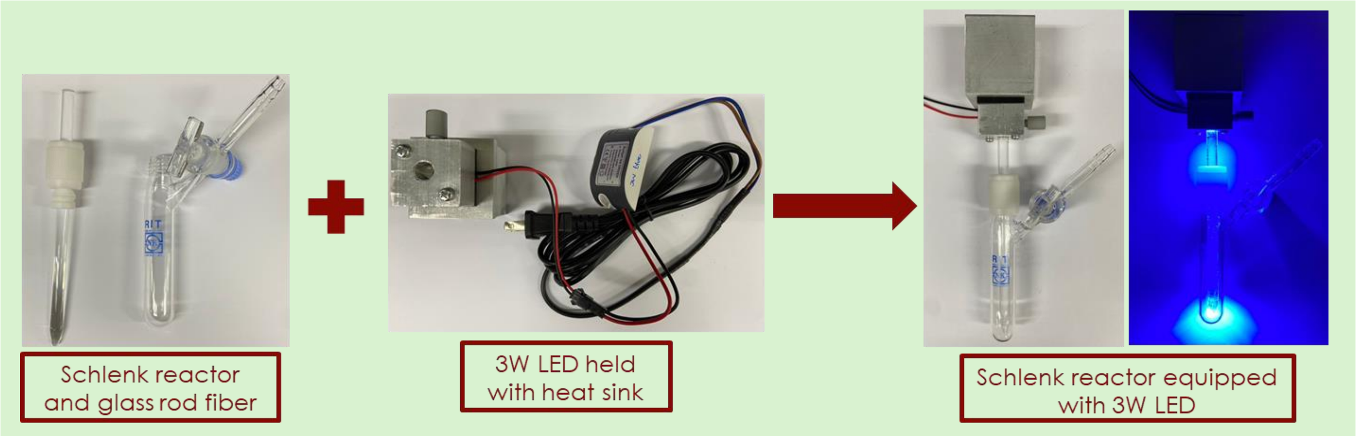


**Figure S3.** Reaction setup using LED equipped with glass rod fiber

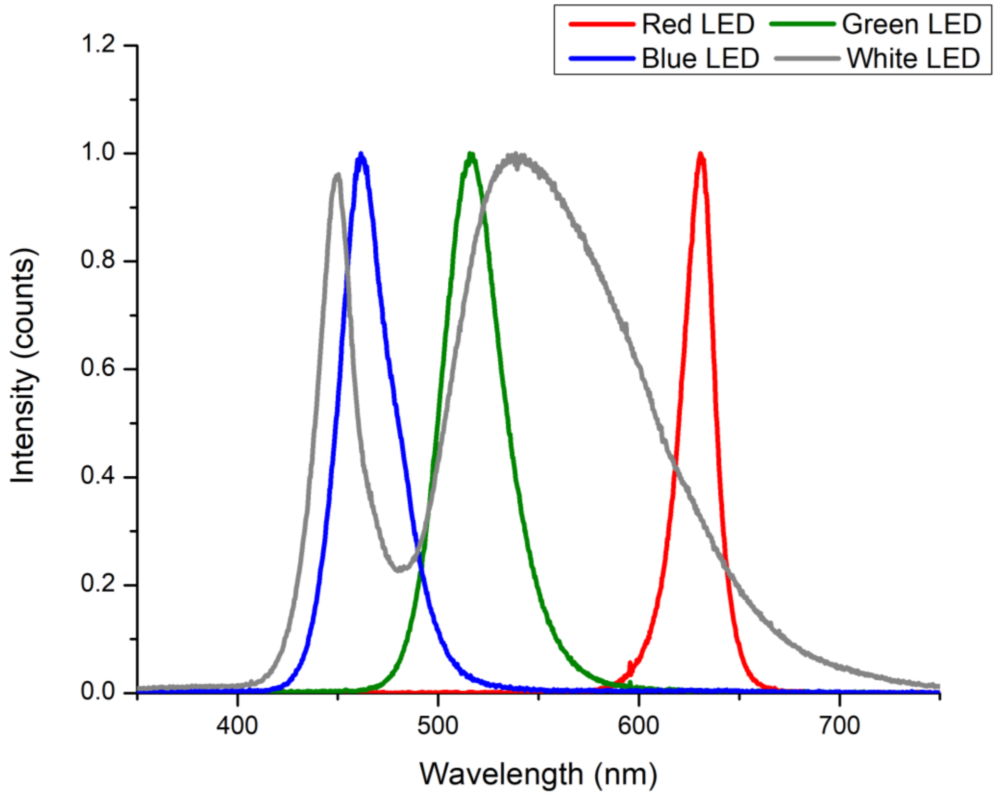


**Figure S4.** Absorption spectra of red, green, blue and white LED sources

## 4. Experimental section

### 4.1. Preparation of starting materials

#### 4.1.1. Preparation of *N*-tosyl-*o*-phenylenediamine (3a)

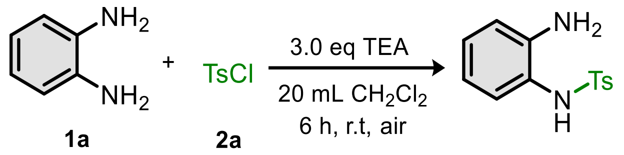


***N*-(2-aminophenyl)-4-methylbenzenesulfonamide**[1] (**3a**): A mixture of *o*-phenylenediamine [CAS NO. 95-54-5] (1.0 eq, 9.25 mmol, 1.00 g), *p*-toluenesulfonyl chloride [CAS NO. 98-59-9] (1.2 eq, 11.10 mmol, 2.12 g) and triethylamine [CAS NO. 121-44-8] (3.0 eq, 27.80 mmol, 3.87 mL) was dissolved by dichloromethane (30 mL) in a 100 mL round bottom flask. The mixture was stirred in an ice bath for 3 hours. The reaction mixture was washed with water (2x10 mL) and the organic portion was extracted with $CH_2Cl_2$ (2x10 mL). Water from organic layer was eliminated by $Na_2SO_4$. After filtration and removal of the solvent under reduced pressure, the crude product was purified by silica gel column chromatography (2:1 of hexane:EtOAc) to afford **3a** in 1.8552 g, 7.07 mmol, 77% yield as a light brown solid. $^1$H-NMR (500 MHz, $CDCl_3$): $\delta$ (ppm) 7.61 (d, *J* = 8.3 Hz, 2H), 7.22 (d, *J* = 8.1 Hz, 2H), 7.01 (ddd, *J* = 8.2, 5.9, 3.0 Hz, 1H), 6.73–6.70 (m, 1H), 6.53–6.49 (m, 2H), 3.94 (s, 2H), 2.39 (s, 3H). $^{13}$C-NMR (126 MHz, $CDCl_3$): $\delta$ (ppm) 144.40, 143.93, 135.97, 129.67, 128.89, 128.60, 127.60, 121.29, 118.71, 117.20, 21.67. ESI-HRMS: m/z: 285.06701 $[M+Na]^+$ (calcd for $[C_{13}H_{14}N_2O_2SNa]^+$ 285.06737).

#### 4.1.2. Preparation of protected aminophenol derivatives

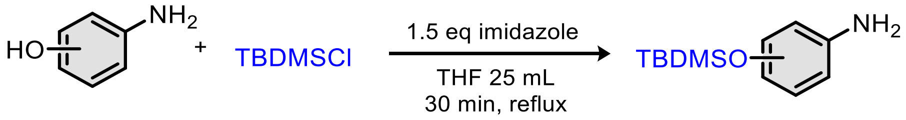


<u>General procedure A</u>: A mixture of corresponding aminophenols (1.0 eq, 13.75 mmol, 1.50 g), *tert*-butyldimethylsilyl chloride (TBDMSCl) [CAS NO. 18162-48-6] (1.2 eq, 16.50 mmol, 2.49 g) and imidazole [CAS NO. 288-32-4] (1.5 eq, 20.63 mmol, 1.40 g) was dissolved by tetrahydrofuran (25 mL) in a 100 mL round bottom flask. The mixture was refluxed for 30 minutes. The reaction mixture was washed with water (2x10 mL) and the organic portion was extracted with EtOAc (2x10 mL). Water from organic layer was eliminated by $Na_2SO_4$. After

filtration and removal of the solvent under reduced pressure, the crude product was purified by silica gel column chromatography (2:1 of hexane:EtOAc as mobile phase) to afford corresponding products.

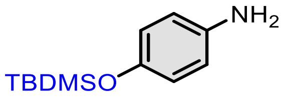


**4-((*tert*-butyldimethylsilyl)oxy)aniline**[2] : was synthesized according to general procedure A by using 4-aminophenol [CAS NO. 123-30-8] (1.0 eq, 13.75 mmol, 1.50 g), *tert*-butyldimethylsilyl chloride [CAS NO. 18162-48-6] (1.2 eq, 16.50 mmol, 2.49 g) and imidazole [CAS NO. 288-32-4] (1.5 eq, 20.63 mmol, 1.40 g), tetrahydrofuran (25 mL) to afford product in 3.0579 g, quantitative yield as a brown liquid. $^{1}$H-NMR (500 MHz, $CDCl_3$): $\delta$ (ppm) 6.68–6.65 (m, 2H), 6.59–6.56 (m, 2H), 3.31 (s, 2H), 0.98 (s, 9H), 0.16 (s, 6H). $^{13}$C-NMR (126 MHz, $CDCl_3$): $\delta$ (ppm) 148.23, 140.36, 120.75, 116.39, 25.84, 18.27, -4.40.

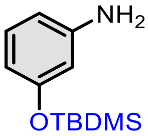


**3-((*tert*-butyldimethylsilyl)oxy)aniline**[3] : was synthesized according to general procedure A by using 3-aminophenol [CAS NO. 591-27-5] (1.0 eq, 13.75 mmol, 1.50 g), *tert*-butyldimethylsilyl chloride [CAS NO. 18162-48-6] (1.2 eq, 16.50 mmol, 2.49 g) and imidazole [CAS NO. 288-32-4] (1.5 eq, 20.63 mmol, 1.40 g), tetrahydrofuran (25 mL) to afford product in 3.4479 g, quantitative yield as a brown liquid. $^{1}$H-NMR (500 MHz, $CDCl_3$): $\delta$ (ppm) 7.03–6.98 (m, 1H), 6.33–6.25 (m, 2H), 6.21 (dd, $J$ = 4.5, 2.3 Hz, 1H), 3.61 (s, 2H), 0.99 (s, 9H), 0.20 (s, 6H). $^{13}$C-NMR (126 MHz, $CDCl_3$): $\delta$ (ppm) 156.76, 147.77, 130.01, 110.54, 108.60, 107.22, 25.80, 18.28, -4.29.

### 4.1.3. General procedure for preparation of isothiocyanate derivatives

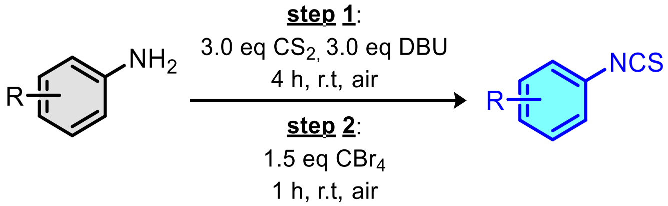


General procedure B[4]: (STEP 1): A mixture of corresponding amines (1.0 eq, 500 mg), carbon disulfide [CAS NO. 75-15-0] (3.0 eq) and DBU [CAS NO. 6674-22-2] (3.0 eq) were dissolved by acetonitrile (10 mL) in a 50 mL round bottom flask. The mixture was stirred at room

temperature for 4 hours. (STEP 2): Then, carbon tetrabromide [CAS NO. 558-13-4] (1.5 eq) was added and stirred at room temperature for 1 hour. After reaction complete, the reaction mixture was washed with water (2x5 mL) and the organic portion was extracted with EtOAc (2x5 mL). The organic layer was dried over anhydrous $Na_2SO_4$. After filtration and removal of the solvent under reduced pressure, the crude product was purified by silica gel column chromatography (3:1 of hexane:EtOAc as mobile phase) to afford corresponding isothiocyanates

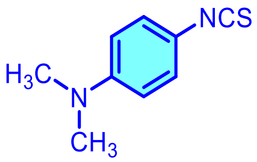


**4-isothiocyanato-*N*,*N*-dimethylaniline**[4] (**4u**): was synthesized according to general procedure B by using *N*,*N*-dimethyl-*p*-phenylenediamine sulfate salt [CAS NO. 536-47-0] (1.0 eq, 2.13 mmol, 500 mg), carbon disulfide [CAS NO. 75-15-0] (3.0 eq), DBU [CAS NO. 6674-22-2] (3.0 eq), carbon tetrabromide [CAS NO. 558-13-4] (1.5 eq) and acetonitrile (10 mL) in a 50 mL round bottom flask to afford **4u** (310.3 mg, 1.74 mmol, 82% yield) as a yellow solid. $^1$H-NMR (500 MHz, $CDCl_3$): $\delta$ (ppm) 7.11 (d, *J* = 9.1 Hz, 2H), 6.61 (d, *J* = 9.0 Hz, 2H), 2.97 (s, 6H). $^{13}$C-NMR (126 MHz, $CDCl_3$): $\delta$ (ppm) 149.39, 132.12, 126.83, 118.76, 112.47, 40.51. Melting point: 72-75$^o$C.

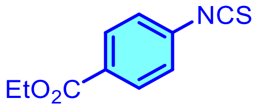


**ethyl 4-isothiocyanatobenzoate**[5] (**4k'**): was synthesized according to general procedure B by using benzocaine [CAS NO. 94-09-7] (1.0 eq, 3.03 mmol, 500 mg), carbon disulfide [CAS NO. 75-15-0] (3.0 eq), DBU [CAS NO. 6674-22-2] (3.0 eq), carbon tetrabromide [CAS NO. 558-13-4] (1.5 eq) and acetonitrile (10 mL) in a 50 mL round bottom flask to afford **4k'** (382.5 mg, 1.85 mmol, 61% yield) as a light brown solid. $^1$H-NMR (500 MHz, $CDCl_3$): $\delta$ (ppm) 8.02 (d, *J* = 8.4 Hz, 2H), 7.25 (d, *J* = 8.6 Hz, 2H), 4.36 (q, *J* = 7.1 Hz, 2H), 1.38 (t, *J* = 7.1 Hz, 3H). $^{13}$C-NMR (126 MHz, $CDCl_3$): $\delta$ (ppm) 165.53, 137.69, 135.56, 131.08, 129.11, 125.72, 61.44, 14.38. Melting point: 62-65$^o$C.

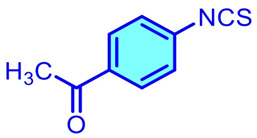


**1-(4-isothiocyanatophenyl)ethan-1-one**[6] (**4l'**): was synthesized according to general procedure B by using 4'-aminoacetophenone [CAS NO. 99-92-3] (1.0 eq, 3.70 mmol), carbon disulfide [CAS NO. 75-15-0] (3.0 eq), DBU [CAS NO. 6674-22-2] (3.0 eq), carbon tetrabromide [CAS NO. 558-13-4] (1.5 eq) and acetonitrile (10 mL) in a 50 mL round bottom flask to afford **4l'** (307.1 mg, 1.73 mmol, 47% yield) as a yellow solid. $^1$H-NMR (500 MHz, $CDCl_3$): $\delta$ (ppm) 7.92 (d, *J* = 8.6 Hz, 2H), 7.26 (d, *J* = 8.7 Hz, 2H), 2.57 (s, 3H). $^{13}$C-NMR (126 MHz, $CDCl_3$): $\delta$ (ppm) 196.56, 137.95, 135.79, 135.42, 129.89, 125.94, 26.71. Melting point: 83-86$^o$C.

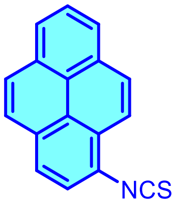


**1-isothiocyanatopyrene**[7] (**4p'**): was synthesized according to general procedure B by using 1-aminopyrene [CAS NO. 99-92-3] (1.0 eq, 2.30 mmol), carbon disulfide [CAS NO. 75-15-0] (3.0 eq), DBU [CAS NO. 6674-22-2] (3.0 eq), carbon tetrabromide [CAS NO. 558-13-4] (1.5 eq) and acetonitrile (10 mL) in a 50 mL round bottom flask to afford **4p'** (526.0 mg, 2.03 mmol, 88% yield) as a light yellow solid. $^1$H-NMR (500 MHz, $CDCl_3$): $\delta$ (ppm) 8.10 (d, *J* = 7.5 Hz, 1H), 8.06 (d, *J* = 7.2 Hz, 1H), 7.94 (ddd, *J* = 25.7, 17.2, 12.2 Hz, 4H), 7.80 (d, *J* = 8.6 Hz, 2H), 7.60 (d, *J* = 8.1 Hz, 1H). $^{13}$C-NMR (126 MHz, $CDCl_3$): $\delta$ (ppm) 136.04, 130.98, 130.67, 129.95, 128.74, 128.00, 126.79, 126.57 (d, *J* = 1.9 Hz), 125.89 (d, *J* = 13.3 Hz), 124.77, 124.55, 123.75, 123.53, 121.26. Melting point: 136-139$^o$C.

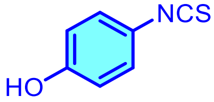


**4-isothiocyanatophenol**[5] (**4x**): was synthesized according to general procedure B by using 4-aminophenol [CAS NO. 123-30-8] (1.0 eq, 4.58 mmol), carbon disulfide [CAS NO. 75-15-0] (3.0 eq), DBU [CAS NO. 6674-22-2] (3.0 eq), carbon tetrabromide [CAS NO. 558-13-4] (1.5 eq) and acetonitrile (10 mL) in a 50 mL round bottom flask to afford **4x** (453.5 mg, 3.00 mmol, 66% yield) as a yellow liquid. $^1$H-NMR (500 MHz, $CDCl_3$): $\delta$ (ppm) 7.11 (d, *J* = 8.8 Hz, 2H), 6.79 (d, *J* = 8.8 Hz, 2H), 5.47 (s, 1H). $^{13}$C-NMR (126 MHz, $CDCl_3$): $\delta$ (ppm) 154.65, 133.77, 127.32, 123.82, 116.44.

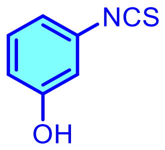

**3-isothiocyanatophenol**[5] (**4y**): was synthesized according to general procedure B by using 3-aminophenol [CAS NO. 1591-27-5] (1.0 eq, 4.58 mmol), carbon disulfide [CAS NO. 75-15-0] (3.0 eq), DBU [CAS NO. 6674-22-2] (3.0 eq), carbon tetrabromide [CAS NO. 558-13-4] (1.5 eq) and acetonitrile (10 mL) in a 50 mL round bottom flask to afford **4y** (460.8 mg, 3.05 mmol, 67% yield) as a yellow solid. $^1$H-NMR (500 MHz, $CDCl_3$): $\delta$ (ppm) 7.20 (t, *J* = 8.1 Hz, 1H), 6.82 (ddd, *J* = 8.0, 1.9, 0.8 Hz, 1H), 6.76 (ddd, *J* = 8.3, 2.5, 0.8 Hz, 1H), 6.70 (t, *J* = 2.2 Hz, 1H), 5.26 (s, 1H). $^{13}$C-NMR (126 MHz, $CDCl_3$): $\delta$ (ppm) 156.26, 135.58, 132.26, 130.64, 118.64, 114.90, 112.83. Melting point: 61-63$^o$C.

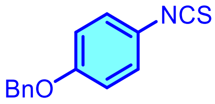

**1-(benzyloxy)-4-isothiocyanatobenzene**[8] (**4b'**): was synthesized according to general procedure B by using 4-( benzyloxy)aniline hydrochloride [CAS NO. 51388-20-6] (1.0 eq, 2.12 mmol), carbon disulfide [CAS NO. 75-15-0] (3.0 eq), DBU [CAS NO. 6674-22-2] (3.0 eq), carbon tetrabromide [CAS NO. 558-13-4] (1.5 eq) and acetonitrile (10 mL) in a 50 mL round bottom flask to afford **4b'** (467.6 mg, 1.94 mmol, 91% yield) as a pale yellow solid. $^1$H-NMR (500 MHz, $CDCl_3$): $\delta$ (ppm) 7.43–7.34 (m, 5H), 7.17 (d, *J* = 9.0 Hz, 2H), 6.93 (d, *J* = 9.0 Hz, 2H), 5.06 (s, 2H). $^{13}$C-NMR (126 MHz, $CDCl_3$): $\delta$ (ppm) 157.80, 136.35, 133.99, 128.81, 128.35, 127.59, 127.13, 123.89, 115.84, 70.41. Melting point: 67-69$^o$C.

### 4.1.4. Preparation of isothiocyanate (4c') as starting material

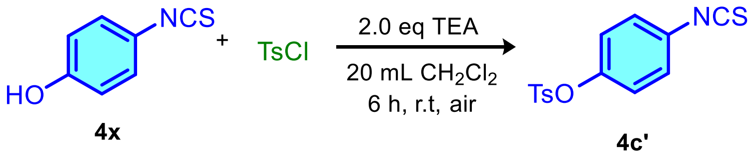


**4-isothiocyanatophenyl 4-methylbenzenesulfonate**[5] (**4c'**): A mixture of 4-isothiocyanatophenol (1.0 eq, 3.31 mmol, 500 mg), *p*-toluenesulfonyl chloride [CAS NO. 98-59-9] (1.2 eq, 3.97 mmol, 757 mg) and triethylamine [CAS NO. 121-44-8] (2.0 eq, 6.62 mmol, 0.92 mL) was dissolved by dichloromethane (20 mL) in a 50 mL round bottom flask. The mixture was

stirred in an ice bath for 1 hours. The reaction mixture was washed with water (2x10 mL) and the organic portion was extracted with $CH_2Cl_2$ (2x10 mL). Water from organic layer was eliminated by $Na_2SO_4$. After filtration and removal of the solvent under reduced pressure, the crude product was purified by silica gel column chromatography (3:1 of hexane:EtOAc) to afford **4c'** (604.1 mg, 1.98 mmol, 60% yield) as orange liquid. $^1$H-NMR (500 MHz, $CDCl_3$): $\delta$ (ppm) 7.67 (d, $J$ = 8.3 Hz, 2H), 7.31 (d, $J$ = 8.4 Hz, 2H), 7.12 (d, $J$ = 8.8 Hz, 2H), 6.95 (d, $J$ = 8.8 Hz, 2H), 2.44 (s, 3H). $^{13}$C-NMR (126 MHz, $CDCl_3$): $\delta$ (ppm) 147.93, 145.90, 136.98, 131.85, 130.34, 130.00, 128.56, 126.97, 123.84, 21.84.

### 4.1.5. General procedure for preparation of isothiocyanate derivatives

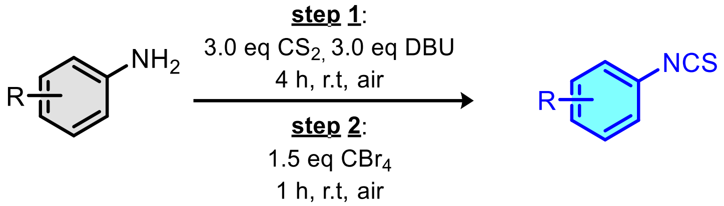


General procedure C: (STEP 1): A mixture of corresponding amines (1.0 eq, 1000 mg), carbon disulfide [CAS NO. 75-15-0] (3.0 eq) and DBU [CAS NO. 6674-22-2] (3.0 eq) were dissolved by acetonitrile (20 mL) in a 50 mL round bottom flask. The mixture was stirred at room temperature for 4 hours. (STEP 2): Then, carbon tetrabromide [CAS NO. 558-13-4] (1.5 eq) was added and stirred at room temperature for 1 hour. After reaction complete, the reaction mixture was washed with water (2x10 mL) and the organic portion was extracted with EtOAc (2x10 mL). The organic layer was dried over anhydrous $Na_2SO_4$. After filtration and removal of the solvent under reduced pressure, the crude product was purified by silica gel column chromatography (3:1 of hexane:EtOAc as mobile phase) to afford corresponding isothiocyanates.

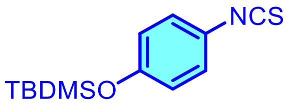


***tert*-butyl(4-isothiocyanatophenoxy)dimethylsilane**[5] (**4z**): was synthesized according to general procedure C by using 4-((*tert*-butyldimethylsilyl)oxy)aniline (1.0 eq, 4.48 mmol), carbon disulfide [CAS NO. 75-15-0] (3.0 eq), DBU [CAS NO. 6674-22-2] (3.0 eq), carbon tetrabromide [CAS NO. 558-13-4] (1.5 eq) and acetonitrile (10 mL) in a 50 mL round bottom flask to afford **4z** (996.4 mg, 3.75 mmol, 84% yield) as a pale yellow liquid. $^1$H-NMR (500 MHz, $CDCl_3$): $\delta$ (ppm)

7.11 (d, $J$ = 8.8 Hz, 2H), 6.79 (d, $J$ = 8.8 Hz, 2H), 0.97 (s, 9H), 0.20 (s, 6H). $^{13}$C-NMR (126 MHz, $CDCl_3$): $\delta$ (ppm) 155.02, 134.03, 127.07, 124.27, 121.11, 25.71, 18.31, -4.34.

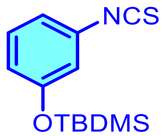


***tert*-butyl(3-isothiocyanatophenoxy)dimethylsilane**[9] (**4a'**): was synthesized according to general procedure C by using 3-((*tert*-butyldimethylsilyl)oxy)aniline (1.0 eq, 4.48 mmol), carbon disulfide [CAS NO. 75-15-0] (3.0 eq), DBU [CAS NO. 6674-22-2] (3.0 eq), carbon tetrabromide [CAS NO. 558-13-4] (1.5 eq) and acetonitrile (20 mL) in a 50 mL round bottom flask to afford **4a'** (748.0 mg, 2.82 mmol, 63% yield) as a pale yellow liquid. $^{1}$H-NMR (500 MHz, $CDCl_3$): $\delta$ (ppm) 7.18 (t, $J$ = 8.1 Hz, 1H), 6.84 (ddd, $J$ = 8.0, 1.9, 0.9 Hz, 1H), 6.76 (ddd, $J$ = 8.3, 2.4, 0.9 Hz, 1H), 6.69 (t, $J$ = 2.1 Hz, 1H), 0.98 (s, 9H), 0.21 (s, 6H). $^{13}$C-NMR (126 MHz, $CDCl_3$): $\delta$ (ppm) 156.59, 135.03, 131.98, 130.28, 119.66, 119.08, 117.53, 25.71, 18.29, -4.32

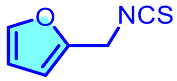


**2-(isothiocyanatomethyl)furan**[10] (**4t'**): was synthesized according to general procedure B by using furfurylamine (1.0 eq,  10.3 mmol), carbon disulfide [CAS NO. 75-15-0] (3.0 eq) and DBU [CAS NO. 6674-22-2] (3.0 eq) and acetonitrile (20 mL) in a 50 mL round bottom flask to afford **4t'** (1.1995 g, 8.62 mmol, 84% yield) as a yellow liquid. $^{1}$H-NMR (500 MHz, $CDCl_3$): $\delta$ (ppm) 7.41 (s, 1H), 6.36 (s, 1H), 6.34 (s, 1H), 4.64 (s, 2H). $^{13}$C-NMR (126 MHz, $CDCl_3$): $\delta$ (ppm) 147.35, 143.34, 134.86, 110.74, 108.86, 41.98.

**4.1.6. Preparation of o-aminophenyl thiourea (5a)**

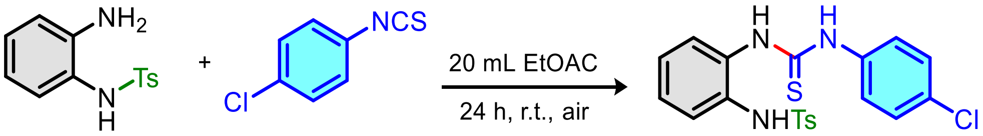


***N*-(2-(3-(4-chlorophenyl)thioureido)phenyl)-4-methylbenzenesulfonamide** (**5a**): A mixture of *N*-(2-aminophenyl)-4-methylbenzenesulfonamide (**3a**) (1.0 eq, 3.81 mmol, 1000 mg), 4-chlorophenyl isothiocyanate (**4a**) (1.2 eq, 4.57 mmol, 776 mg) were dissolved by ethyl acetate 20 mL in 50 round bottom flask. The mixture was stirred at room temperature for 24 hours until

observing white precipitate. After that, the reaction mixture was recrystallized and washed with 7:3 (hexane:EtOAc) and 100% hexane, respectively to afford **5a** (858 mg, 2.16 mmol, 57% yield). $^{1}$H-NMR (500 MHz, DMSO-d$_6$): $\delta$ (ppm) 10.13 (s, 1H), 9.54 (s, 1H), 9.08 (s, 1H), 7.59 (d, *J* = 8.1 Hz, 3H), 7.52 (d, *J* = 8.7 Hz, 2H), 7.37 (d, *J* = 8.7 Hz, 2H), 7.30 (d, *J* = 8.1 Hz, 2H), 7.11 (t, *J* = 7.5 Hz, 1H), 7.03 (t, *J* = 7.6 Hz, 1H), 6.85 (d, *J* = 7.9 Hz, 1H), 2.30 (s, 3H). $^{13}$C-NMR (126 MHz, DMSO-d$_6$): $\delta$ (ppm) 179.87, 143.34, 138.16, 136.74, 133.60, 130.86, 129.70, 128.57, 128.46, 128.09, 126.89, 126.15, 125.82, 125.31, 124.73, 21.06. Melting point: 164-167°C. ESI-HRMS: m/z: 454.04084 $[M+Na]^+$ (calcd for $[C_{20}H_{18}ClN_3O_2S_2Na]^+$ 454.04267).

### 4.2. General procedure for visible-light-mediated photocatalyst-free reaction for synthesis of *N*-substituted-2-aminobenzimidazoles (6a-6z and 6a'-6y')

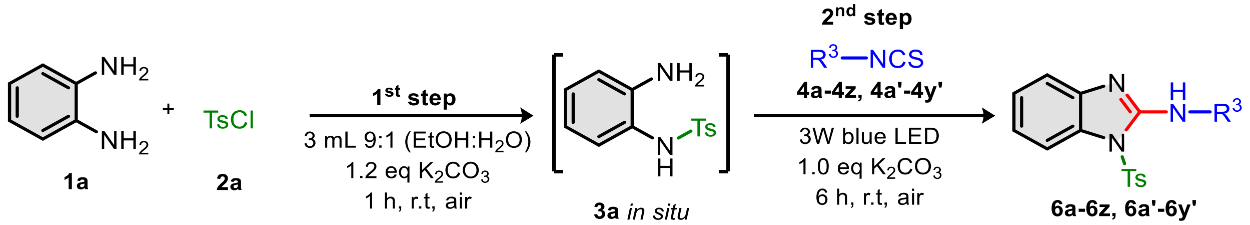


<u>General procedure D</u>: (STEP 1): A mixture of *o*-phenylenediamine (**1a**) [CAS NO. 95-54-5] (1.2 eq, 0.48 mmol, 52 mg), tosyl chloride (**2a**) [CAS NO. 98-59-9] (1.2 eq, 0.48 mmol, 92 mg) and potassium carbonate [CAS NO. 584-08-7] (1.2 eq, 0.48 mmol, 66.3 mg) was dissolved by co-solvent between 90% ethanol and 10% water (3.0 mL) in pyrex schlenk reactor at room temperature for 1 hour under air. (STEP 2): After that, the corresponding isothiocyanate (**4a-4z, 4a'-4y'**) (1.0 eq, 0.40 mmol) and potassium carbonate (1.0 eq, 0.40 mmol, 55.3 mg) were added into pyrex schlenk reactor as single-flask operation. The reaction was irradiated by 3W blue LED equipped with glass rod fiber at room temperature for 6 hours under air. The reaction mixture was washed water (2x10 mL) and the organic portion was extracted with EtOAc (3x10 mL). Water from organic layer was eliminated by $Na_2SO_4$. After filtration and removal of the solvent under reduced pressure, the crude product was purified by silica gel column chromatography (3:1 of hexane:EtOAc as mobile phase) to afford *N*-substituted-2-aminobenzimidazoles **6a-6z, 6a'-6y'** as corresponding products.

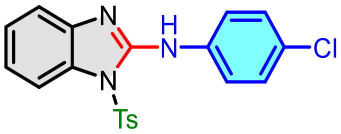


**N-(4-chlorophenyl)-1-tosyl-1H-benzo[d]imidazol-2-amine**[11] (**6a**): was synthesized according to general procedure D by using 4-chlorophenyl isothiocyanate (**4a**) [CAS NO. 2131-55-7] (1.0 eq, 0.40 mmol), *o*-phenylenediamine (**1a**) [CAS NO. 95-54-5] (1.2 eq, 0.48 mmol, 52 mg), tosyl chloride (**2a**) [CAS NO. 98-59-9] (1.2 eq, 0.48 mmol, 92 mg) and potassium carbonate [CAS NO. 584-08-7] (1.2 eq & 1.0 eq) in co-solvent between 90% ethanol and 10% water (3.00 mL) to afford **6a** (129.9 mg, 0.33 mmol, 82% yield) as a white solid. $^1$H-NMR (500 MHz, $CDCl_3$): $\delta$ (ppm) 8.69 (s, 1H), 7.78 (dd, $J$ = 7.9, 5.9 Hz, 3H), 7.75–7.72 (m, 2H), 7.44 (d, $J$ = 7.7 Hz, 1H), 7.37–7.34 (m, 2H), 7.24 (dd, $J$ = 10.6, 4.5 Hz, 3H), 7.18–7.13 (m, 1H), 2.33 (s, 3H). $^{13}$C-NMR (126 MHz, $CDCl_3$): $\delta$ (ppm) 147.52, 146.46, 142.17, 137.10, 134.11, 130.39, 129.97, 129.26, 128.28, 126.90, 125.27, 122.56, 120.40, 117.94, 112.83, 21.72. ESI-HRMS: m/z: found 398.07116 $[M+H]^+$ (calcd for $[C_{20}H_{17}ClN_3O_2S]^+$ 398.07300).

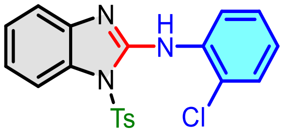


**N-(2-chlorophenyl)-1-tosyl-1H-benzo[d]imidazol-2-amine**[11] (**6b**): was synthesized according to general procedure D by using 2-chlorophenyl isothiocyanate (**4b**) [CAS NO. 2740-81-0] (1.0 eq, 0.40 mmol), *o*-phenylenediamine (**1a**) [CAS NO. 95-54-5] (1.2 eq, 0.48 mmol, 52 mg), tosyl chloride (**2a**) [CAS NO. 98-59-9] (1.2 eq, 0.48 mmol, 92 mg) and potassium carbonate [CAS NO. 584-08-7] (1.2 eq & 1.0 eq) in co-solvent between 90% ethanol and 10% water (3.00 mL) to afford **6b** (102.6 mg, 0.26 mmol, 65% yield) as a white solid. $^1$H-NMR (500 MHz, $CDCl_3$): $\delta$ (ppm) 9.40 (s, 1H), 8.77 (d, $J$ = 8.3 Hz, 1H), 7.88–7.84 (m, 3H), 7.49–7.43 (m, 2H), 7.37–7.33 (m, 1H), 7.27–7.21 (m, 3H), 7.19 (td, $J$ = 7.8, 1.2 Hz, 1H), 7.05–7.00 (m, 1H), 2.32 (s, 3H). $^{13}$C-NMR (126 MHz, $CDCl_3$): $\delta$ (ppm) 147.09, 146.42, 141.94, 135.55, 134.22, 130.38, 130.00, 129.23, 127.96, 126.98, 125.19, 123.49, 122.75, 122.48, 120.05, 118.07, 112.86, 21.71. ESI-HRMS: m/z: found 398.07171 $[M+H]^+$ (calcd for $[C_{20}H_{17}ClN_3O_2S]^+$ 398.07300).

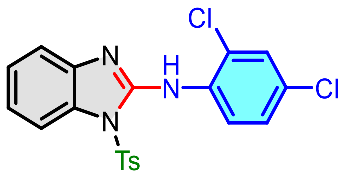

***N*-(2,4-dichlorophenyl)-1-tosyl-1*H*-benzo[*d*]imidazol-2-amine** (**6c**): was synthesized according to general procedure D by using 2,4-dichlorophenyl isothiocyanate (**4c**) [CAS NO. 6590-96-1] (1.0 eq, 0.40 mmol), *o*-phenylenediamine (**1a**) [CAS NO. 95-54-5] (1.2 eq, 0.48 mmol, 52 mg), tosyl chloride (**2a**) [CAS NO. 98-59-9] (1.2 eq, 0.48 mmol, 92 mg) and potassium carbonate [CAS NO. 584-08-7] (1.2 eq & 1.0 eq) in co-solvent between 90% ethanol and 10% water (3.00 mL) to afford **6c** (118.5 mg, 0.276 mmol, 69% yield) as a white solid. $^{1}$H-NMR (500 MHz, $CDCl_3$): $\delta$ (ppm) 9.37 (s, 1H), 8.75 (d, *J* = 8.9 Hz, 1H), 7.83 (ddd, *J* = 6.7, 4.1, 2.3 Hz, 3H), 7.45 (t, *J* = 4.7 Hz, 2H), 7.30 (dd, *J* = 8.9, 2.4 Hz, 1H), 7.26–7.23 (m, 3H), 7.19 (td, *J* = 7.8, 1.2 Hz, 1H), 2.34 (s, 3H). $^{13}$C-NMR (126 MHz, $CDCl_3$): $\delta$ (ppm) 146.78, 146.58, 141.80, 134.42, 134.18, 130.47, 129.99, 128.89, 128.04, 127.72, 127.00, 125.30, 122.98, 122.91, 120.70, 118.18, 112.91, 21.80. Melting point: 180-182°C. ESI-HRMS: m/z: found 432.03415 $[M+H]^+$ (calcd for $[C_{20}H_{16}Cl_2N_3O_2S]^+$ 432.03403).

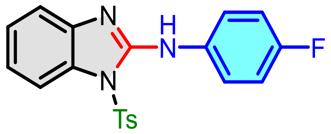

***N*-(4-fluorophenyl)-1-tosyl-1*H*-benzo[*d*]imidazol-2-amine**[11] (**6d**): was synthesized according to general procedure D by using 4-fluorophenyl isothiocyanate (**4d**) [CAS NO. 1544-68-9] (1.0 eq, 0.40 mmol), *o*-phenylenediamine (**1a**) [CAS NO. 95-54-5] (1.2 eq, 0.48 mmol, 52 mg), tosyl chloride (**2a**) [CAS NO. 98-59-9] (1.2 eq, 0.48 mmol, 92 mg) and potassium carbonate [CAS NO. 584-08-7] (1.2 eq & 1.0 eq) in co-solvent between 90% ethanol and 10% water (3.00 mL) to afford **6d** (108.7 mg, 0.284 mmol, 71% yield) as a white solid. $^{1}$H-NMR (500 MHz, $CDCl_3$): $\delta$ (ppm) 8.61 (s, 1H), 7.78 (dd, *J* = 14.2, 8.2 Hz, 3H), 7.74–7.69 (m, 2H), 7.42 (d, *J* = 7.9 Hz, 1H), 7.23 (ddd, *J* = 11.9, 6.5, 2.5 Hz, 3H), 7.17–7.07 (m, 3H), 2.34 (s, 3H). $^{13}$C-NMR (126 MHz, $CDCl_3$): $\delta$ (ppm) 159.02 (d, *J* = 242.67 Hz), 148.11, 146.43, 142.26, 134.53 (d, *J* = 1.91 Hz), 134.18, 130.39, 130.09, 126.93, 125.24, 122.39, 121.11 (d, *J* = 7.68 Hz), 117.82, 116.05, 115.87, 112.81, 21.73. $^{19}$F-NMR (471 MHz, $CDCl_3$): $\delta$ (ppm) -119.12 (s). ESI-HRMS: m/z: found 382.10249 $[M+H]^+$ (calcd for $[C_{20}H_{17}FN_3O_2S]^+$ 382.10255).

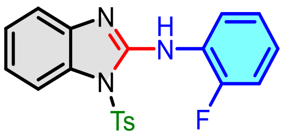

**$N$-(2-fluorophenyl)-1-tosyl-1$H$-benzo[$d$]imidazol-2-amine**[11] (**6e**): was synthesized according to general procedure D by using 2-fluorophenyl isothiocyanate (**4e**) [CAS NO. 38985-64-7] (1.0 eq, 0.40 mmol), *o*-phenylenediamine (**1a**) [CAS NO. 95-54-5] (1.2 eq, 0.48 mmol, 52 mg), tosyl chloride (**2a**) [CAS NO. 98-59-9] (1.2 eq, 0.48 mmol, 92 mg) and potassium carbonate [CAS NO. 584-08-7] (1.2 eq & 1.0 eq) in co-solvent between 90% ethanol and 10% water (3.00 mL) to afford **6e** (132.3 mg, 0.35 mmol, 87 yield) as a white solid. $^1$H-NMR (500 MHz, $CDCl_3$): $\delta$ (ppm) 9.03 (s, 1H), 8.63 (td, $J$ = 8.3, 1.6 Hz, 1H), 7.84 (dt, $J$ = 8.6, 2.0 Hz, 3H), 7.47 (dd, $J$ = 4.3, 3.9 Hz, 1H), 7.27–7.17 (m, 6H), 7.08–7.03 (m, 1H), 2.33 (s, 3H). $^{13}$C-NMR (126 MHz, $CDCl_3$): $\delta$ (ppm) 152.60 (d, $J$ = 243.47 Hz), 147.22, 146.44, 142.13, 134.19, 130.39, 130.06, 127.11 (d, $J$ = 9.99 Hz), 126.97, 125.21, 124.82 (d, $J$ = 3.44 Hz), 123.31 (d, $J$ = 7.42 Hz), 122.65, 120.58, 118.03, 114.88 (d, $J$ = 18.90 Hz), 112.84, 21.71. $^{19}$F-NMR (471 MHz, $CDCl_3$): $\delta$ (ppm) -131.60. ESI-HRMS: m/z: found 382.10206 $[M+H]^+$ (calcd for $[C_{20}H_{17}FN_3O_2S]^+$ 382.10255).

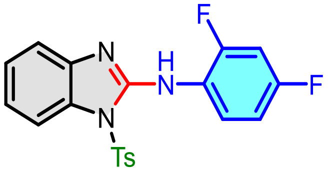

**$N$-(2,4-difluorophenyl)-1-tosyl-1$H$-benzo[$d$]imidazol-2-amine** (**6f**): was synthesized according to general procedure D by using 2,4-difluorophenyl isothiocyanate (**4f**) [CAS NO. 141106-52-7] (1.0 eq, 0.40 mmol), *o*-phenylenediamine (**1a**) [CAS NO. 95-54-5] (1.2 eq, 0.48 mmol, 52 mg), tosyl chloride (**2a**) [CAS NO. 98-59-9] (1.2 eq, 0.48 mmol, 92 mg) and potassium carbonate [CAS NO. 584-08-7] (1.2 eq & 1.0 eq) in co-solvent between 90% ethanol and 10% water (3.00 mL) to afford **6f** (113.3 mg, 0.284 mmol, 71% yield) as a white solid. $^1$H-NMR (500 MHz, $CDCl_3$): $\delta$ (ppm) 8.84 (d, $J$ = 2.6 Hz, 1H), 8.59–8.49 (m, 1H), 7.83–7.78 (m, 3H), 7.42 (dd, $J$ = 4.3, 3.9 Hz, 1H), 7.26–7.21 (m, 9H), 7.16 (td, $J$ = 7.8, 1.2 Hz, 1H), 6.99–6.91 (m, 2H), 2.34 (s, 3H). $^{13}$C-NMR (126 MHz, $CDCl_3$): $\delta$ (ppm) 159.02 (d, $J$ = 11.04 Hz), 157.07 (d, $J$ = 10.89 Hz), 153.58 (d, $J$ = 11.61 Hz), 151.62 (d, $J$ = 12.02 Hz), 147.41, 146.54, 142.04, 134.18, 130.45, 130.13, 127.01, 125.27, 123.51 (dd, $J$ = 10.27, 3.68 Hz), 122.70, 121.68 (d, $J$ = 8.64 Hz), 118.01, 112.84, 111.43 (d, $J$ = 3.66 Hz), 111.26 (d,

*J* = 3.63 Hz), 103.79 (d, *J* = 26.82, 22.82 Hz), 21.78. $^{19}$F-NMR (471 MHz, $CDCl_3$): $\delta$ (ppm) -116.43, -126.67. Melting point: 184-186°C. ESI-HRMS: m/z: found 400.09444 $[M+H]^+$ (calcd for $[C_{20}H_{16}F_2N_3O_2S]^+$ 400.09313).

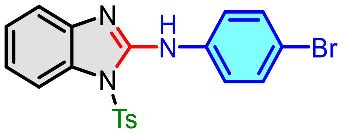


***N*-(4-bromophenyl)-1-tosyl-1*H*-benzo[*d*]imidazol-2-amine**[11] (**6g**): was synthesized according to general procedure D by using 4-bromophenyl isothiocyanate (**4g**) [CAS NO. 1985-12-2] (1.0 eq, 0.40 mmol), *o*-phenylenediamine (**1a**) [CAS NO. 95-54-5] (1.2 eq, 0.48 mmol, 52 mg), tosyl chloride (**2a**) [CAS NO. 98-59-9] (1.2 eq, 0.48 mmol, 92 mg) and potassium carbonate [CAS NO. 584-08-7] (1.2 eq & 1.0 eq) in co-solvent between 90% ethanol and 10% water (3.00 mL) to afford **6g** (116.3 mg, 0.292 mmol, 73% yield) as a white solid. $^1$H-NMR (500 MHz, $CDCl_3$): $\delta$ (ppm) 8.70 (s, 1H), 7.78 (dt, *J* = 7.5, 2.3 Hz, 3H), 7.71–7.67 (m, 2H), 7.52–7.48 (m, 2H), 7.46–7.43 (m, 1H), 7.25–7.21 (m, 3H), 7.16 (td, *J* = 7.8, 1.2 Hz, 1H), 2.33 (s, 3H). $^{13}$C-NMR (126 MHz, $CDCl_3$): $\delta$ (ppm) 147.46, 146.50, 142.19, 137.63, 134.14, 132.23, 130.43, 129.99, 126.93, 125.31, 122.62, 120.76, 117.99, 115.82, 112.87, 21.77. ESI-HRMS: m/z: found 442.01933 $[M+H]^+$ (calcd for $[C_{20}H_{17}BrN_3O_2S]^+$ 442.02249).

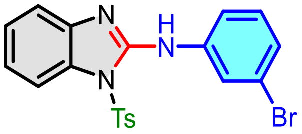


***N*-(3-bromophenyl)-1-tosyl-1*H*-benzo[*d*]imidazol-2-amine**[11] (**6h**): was synthesized according to general procedure D by using 3-bromophenyl isothiocyanate (**4h**) [CAS NO. 2131-59-1] (1.0 eq, 0.40 mmol), *o*-phenylenediamine (**1a**) [CAS NO. 95-54-5] (1.2 eq, 0.48 mmol, 52 mg), tosyl chloride (**2a**) [CAS NO. 98-59-9] (1.2 eq, 0.48 mmol, 92 mg) and potassium carbonate [CAS NO. 584-08-7] (1.2 eq & 1.0 eq) in co-solvent between 90% ethanol and 10% water (3.00 mL) to afford **6h** (136.1 mg, 0.308 mmol, 77% yield) as a white solid. $^1$H-NMR (500 MHz, $CDCl_3$): $\delta$ (ppm) 8.70 (s, 1H), 8.06 (d, *J* = 1.9 Hz, 1H), 7.76 (dd, *J* = 8.4, 1.8 Hz, 3H), 7.66 (dt, *J* = 7.3, 2.1 Hz, 1H), 7.45 (d, *J* = 7.8 Hz, 1H), 7.25–7.21 (m, 5H), 7.15 (td, *J* = 8.1, 1.0 Hz, 1H), 2.33 (s, 3H). $^{13}$C-NMR (126 MHz, $CDCl_3$): $\delta$ (ppm) 147.19, 146.52, 142.13, 139.80, 134.12, 130.59, 130.45, 129.96, 126.93,

126.30, 125.32, 123.02, 122.71, 121.85, 118.14, 117.59, 112.87, 21.77. ESI-HRMS: m/z: found 442.02046 $[M+H]^+$ (calcd for $[C_{20}H_{17}BrN_3O_2S]^+$ 442.02248).

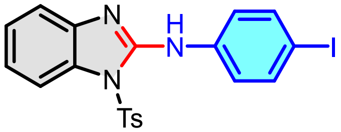


***N*-(4-iodophenyl)-1-tosyl-1*H*-benzo[*d*]imidazol-2-amine**[11] (**6i**): was synthesized according to general procedure D by using 4-iodophenyl isothiocyanate (**4i**) [CAS NO. 2059-76-9] (1.0 eq, 0.40 mmol), *o*-phenylenediamine (**1a**) [CAS NO. 95-54-5] (1.2 eq, 0.48 mmol, 52 mg), tosyl chloride (**2a**) [CAS NO. 98-59-9] (1.2 eq, 0.48 mmol, 92 mg) and potassium carbonate [CAS NO. 584-08-7] (1.2 eq & 1.0 eq) in co-solvent between 90% ethanol and 10% water (3.00 mL) to afford **6i** (155.7 mg, 0.32 mmol, 80% yield) as a white solid. $^1$H-NMR (500 MHz, $CDCl_3$): $\delta$ (ppm) 8.69 (s, 1H), 7.79–7.76 (m, 3H), 7.70–7.66 (m, 2H), 7.59–7.56 (m, 2H), 7.44 (dd, *J* = 7.9, 0.4 Hz, 1H), 7.23 (ddd, *J* = 8.2, 5.5, 1.7 Hz, 3H), 7.16 (td, *J* = 7.9, 1.2 Hz, 1H), 2.33 (s, 3H). $^{13}$C-NMR (126 MHz, $CDCl_3$): $\delta$ (ppm) 147.29, 146.44, 142.14, 138.27, 138.09, 134.05, 130.38, 129.91, 126.87, 125.26, 122.60, 121.05, 117.97, 112.82, 86.11, 21.73. ESI-HRMS: m/z: found 490.00709 $[M+H]^+$ (calcd for $[C_{20}H_{17}IN_3O_2S]^+$ 490.00863).

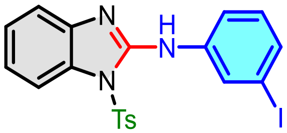


***N*-(3-iodophenyl)-1-tosyl-1*H*-benzo[*d*]imidazol-2-amine**[11] (**6j**): was synthesized according to general procedure D by using 3-iodophenyl isothiocyanate (**4j**) [CAS NO. 3125-73-3] (1.0 eq, 0.40 mmol), *o*-phenylenediamine (**1a**) [CAS NO. 95-54-5] (1.2 eq, 0.48 mmol, 52 mg), tosyl chloride (**2a**) [CAS NO. 98-59-9] (1.2 eq, 0.48 mmol, 92 mg) and potassium carbonate [CAS NO. 584-08-7] (1.2 eq & 1.0 eq) in co-solvent between 90% ethanol and 10% water (3.00 mL) to afford **6j** (117.1 mg, 0.24 mmol, 60% yield) as a white solid. $^1$H-NMR (500 MHz, $CDCl_3$): $\delta$ (ppm) 8.66 (s, 1H), 8.16 (t, *J* = 1.8 Hz, 1H), 7.78–7.74 (m, 4H), 7.44 (dd, *J* = 10.6, 8.0 Hz, 2H), 7.23 (ddd, *J* = 7.6, 5.3, 2.0 Hz, 3H), 7.17–7.13 (m, 1H), 7.10 (t, *J* = 8.0 Hz, 1H), 2.33 (s, 3H). $^{13}$C-NMR (126 MHz, $CDCl_3$): $\delta$ (ppm) 147.14, 146.51, 142.12, 139.64, 134.08, 132.34, 130.77, 130.45, 129.94, 127.58, 126.92, 125.30, 122.68, 118.28, 118.11, 112.84, 94.56, 21.78. ESI-HRMS: m/z: found 490.00594 $[M+H]^+$ (calcd for $[C_{20}H_{17}IN_3O_2S]^+$ 490.00863).

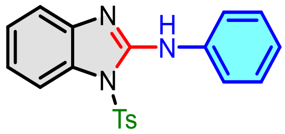

***N*-phenyl-1-tosyl-1*H*-benzo[*d*]imidazol-2-amine**[11] (**6k**): was synthesized according to general procedure D by using phenyl isothiocyanate (**4k**) [CAS NO. 103-72-0] (1.0 eq, 0.40 mmol), *o*-phenylenediamine (**1a**) [CAS NO. 95-54-5] (1.2 eq, 0.48 mmol, 52 mg), tosyl chloride (**2a**) [CAS NO. 98-59-9] (1.2 eq, 0.48 mmol, 92 mg) and potassium carbonate [CAS NO. 584-08-7] (1.2 eq & 1.0 eq) in co-solvent between 90% ethanol and 10% water (3.00 mL) to afford **6k** (118.4 mg, 0.324 mmol, 81% yield) as a white solid. $^1$H-NMR (500 MHz, $CDCl_3$): $\delta$ (ppm) 8.69 (s, 1H), 7.82–7.76 (m, 5H), 7.46–7.39 (m, 3H), 7.23 (ddd, *J* = 7.8, 4.0, 1.2 Hz, 3H), 7.17–7.10 (m, 2H), 2.34 (s, 3H). $^{13}$C-NMR (126 MHz, $CDCl_3$): $\delta$ (ppm) 147.91, 146.36, 142.46, 138.51, 134.26, 130.39, 130.06, 129.38, 126.95, 125.22, 123.49, 122.36, 119.25, 117.90, 112.85, 21.74. ESI-HRMS: m/z: found 364.11087 $[M+H]^+$ (calcd for $[C_{20}H_{18}N_3O_2S]^+$ 364.11197).

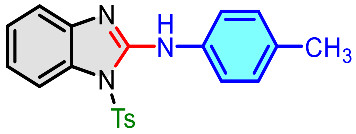

***N*-(*p*-tolyl)-1-tosyl-1*H*-benzo[*d*]imidazol-2-amine**[11] (**6l**): was synthesized according to general procedure D by using 4-methylphenyl isothiocyanate (**4l**) [CAS NO. 622-59-3] (1.0 eq, 0.40 mmol), *o*-phenylenediamine (**1a**) [CAS NO. 95-54-5] (1.2 eq, 0.48 mmol, 52 mg), tosyl chloride (**2a**) [CAS NO. 98-59-9] (1.2 eq, 0.48 mmol, 92 mg) and potassium carbonate [CAS NO. 584-08-7] (1.2 eq & 1.0 eq) in co-solvent between 90% ethanol and 10% water (3.00 mL) to afford **6l** (110.1 mg, 0.292 mmol, 73% yield) as a white solid. $^1$H-NMR (500 MHz, $CDCl_3$): $\delta$ (ppm) 8.60 (s, 1H), 7.82–7.77 (m, 3H), 7.65–7.62 (m, 2H), 7.43 (d, *J* = 7.7 Hz, 1H), 7.24–7.20 (m, 5H), 7.16–7.11 (m, 1H), 2.36 (s, 3H), 2.33 (s, 3H). $^{13}$C-NMR (126 MHz, $CDCl_3$): $\delta$ (ppm) 148.24, 146.29, 142.49, 135.88, 134.26, 133.24, 130.34, 130.10, 129.85, 126.93, 125.17, 122.19, 119.56, 117.75, 112.81, 21.71, 20.93. ESI-HRMS: m/z: found 378.12781 $[M+H]^+$ (calcd for $[C_{21}H_{20}N_3O_2S]^+$ 378.12762).

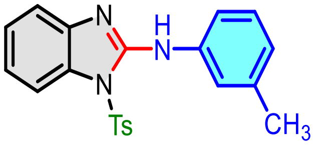

**N-(m-tolyl)-1-tosyl-1H-benzo[d]imidazol-2-amine**[11] (**6m**): was synthesized according to general procedure D by using 3-methylphenyl isothiocyanate (**4m**) [CAS NO. 621-30-7] (1.0 eq, 0.40 mmol), *o*-phenylenediamine (**1a**) [CAS NO. 95-54-5] (1.2 eq, 0.48 mmol, 52 mg), tosyl chloride (**2a**) [CAS NO. 98-59-9] (1.2 eq, 0.48 mmol, 92 mg) and potassium carbonate [CAS NO. 584-08-7] (1.2 eq & 1.0 eq) in co-solvent between 90% ethanol and 10% water (3.00 mL) to afford **6m** (120.2 mg, 0.32 mmol, 80% yield) as a white solid. $^1$H-NMR (500 MHz, $CDCl_3$): $\delta$ (ppm) 8.69 (s, 1H), 7.82 (d, $J$ = 8.4 Hz, 3H), 7.66–7.63 (m, 1H), 7.61 (s, 1H), 7.50–7.47 (m, 1H), 7.32 (t, $J$ = 7.8 Hz, 1H), 7.25 (td, $J$ = 7.8, 1.0 Hz, 1H), 7.21 (d, $J$ = 8.3 Hz, 2H), 7.17 (td, $J$ = 8.1, 1.0 Hz, 1H), 6.97 (d, $J$ = 7.5 Hz, 1H), 2.43 (s, 3H), 2.31 (s, 3H). $^{13}$C-NMR (126 MHz, $CDCl_3$): $\delta$ (ppm) 147.86, 146.22, 142.40, 139.19, 138.30, 134.13, 130.25, 129.96, 129.13, 126.82, 125.12, 124.29, 122.23, 119.76, 117.77, 116.32, 112.76, 21.60. ESI-HRMS: m/z: found 378.12815 $[M+H]^+$ (calcd for $[C_{21}H_{20}N_3O_2S]^+$ 378.12762).

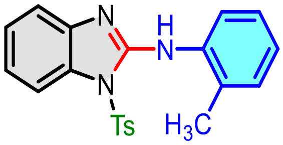

**N-(o-tolyl)-1-tosyl-1H-benzo[d]imidazol-2-amine**[11] (**6n**): was synthesized according to general procedure D by using 2-methylphenyl isothiocyanate (**4n**) [CAS NO. 614-69-7] (1.0 eq, 0.40 mmol), *o*-phenylenediamine (**1a**) [CAS NO. 95-54-5] (1.2 eq, 0.48 mmol, 52 mg), tosyl chloride (**2a**) [CAS NO. 98-59-9] (1.2 eq, 0.48 mmol, 92 mg) and potassium carbonate [CAS NO. 584-08-7] (1.2 eq & 1.0 eq) in co-solvent between 90% ethanol and 10% water (3.00 mL) to afford **6n** (130.1 mg, 0.344 mmol, 86 yield) as a white solid. $^1$H-NMR (500 MHz, $CDCl_3$): $\delta$ (ppm) 8.68 (s, 1H), 8.40 (d, $J$ = 8.2 Hz, 1H), 7.86–7.80 (m, 3H), 7.49–7.45 (m, 1H), 7.34 (t, $J$ = 7.7 Hz, 1H), 7.29–7.22 (m, 4H), 7.16 (td, $J$ = 7.9, 1.2 Hz, 1H), 7.09 (dd, $J$ = 10.7, 4.2 Hz, 1H), 2.46 (s, 3H), 2.33 (s, 3H). $^{13}$C-NMR (126 MHz, $CDCl_3$): $\delta$ (ppm) 148.14, 146.28, 142.31, 136.88, 134.26, 130.59, 130.29, 130.07, 127.19, 127.13, 126.80, 125.09, 123.70, 122.21, 120.09, 117.81, 112.65, 21.63, 17.98. ESI-HRMS: m/z: found 378.12793 $[M+H]^+$ (calcd for $[C_{21}H_{20}N_3O_2S]^+$ 378.12762).

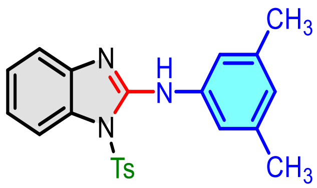


***N*-(3,5-dimethylphenyl)-1-tosyl-1*H*-benzo[*d*]imidazol-2-amine**[11] (**6o**): was synthesized according to general procedure D by using 3,5-dimethylphenyl isothiocyanate (**4o**) [CAS NO. 40046-30-8] (1.0 eq, 0.40 mmol), *o*-phenylenediamine (**1a**) [CAS NO. 95-54-5] (1.2 eq, 0.48 mmol, 52 mg), tosyl chloride (**2a**) [CAS NO. 98-59-9] (1.2 eq, 0.48 mmol, 92 mg) and potassium carbonate [CAS NO. 584-08-7] (1.2 eq & 1.0 eq) in co-solvent between 90% ethanol and 10% water (3.00 mL) to afford **6o** (135.5 mg, 0.348 mmol, 87% yield) as a white solid. $^{1}$H-NMR (500 MHz, $CDCl_3$): $\delta$ (ppm) 8.62 (s, 1H), 7.79 (d, *J* = 8.2 Hz, 3H), 7.46 (d, *J* = 7.9 Hz, 1H), 7.41 (s, 2H), 7.22 (dd, *J* = 13.4, 8.0 Hz, 3H), 7.14 (t, *J* = 7.8 Hz, 1H), 6.78 (s, 1H), 2.38 (s, 6H), 2.32 (s, 3H). $^{13}$C-NMR (126 MHz, $CDCl_3$): $\delta$ (ppm) 147.96, 146.23, 142.50, 139.07, 138.25, 134.22, 130.30, 130.01, 126.88, 125.35, 125.13, 122.21, 117.81, 116.99, 112.80, 21.66, 21.55. ESI-HRMS: m/z: found 392.14331 $[M+H]^+$ (calcd for $[C_{22}H_{22}N_3O_2S]^+$ 392.14327).

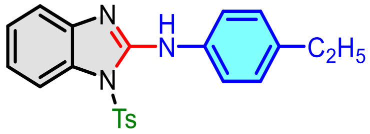


***N*-(4-ethylphenyl)-1-tosyl-1*H*-benzo[*d*]imidazol-2-amine**[11] (**6p**): was synthesized according to general procedure D by using 4-ethylphenyl isothiocyanate (**4p**) [CAS NO. 18856-63-8] (1.0 eq, 0.40 mmol), *o*-phenylenediamine (**1a**) [CAS NO. 95-54-5] (1.2 eq, 0.48 mmol, 52 mg), tosyl chloride (**2a**) [CAS NO. 98-59-9] (1.2 eq, 0.48 mmol, 92 mg) and potassium carbonate [CAS NO. 584-08-7] (1.2 eq & 1.0 eq) in co-solvent between 90% ethanol and 10% water (3.00 mL) to afford **6p** (130.0 mg, 0.332 mmol, 83% yield) as a colorless oil. $^{1}$H-NMR (500 MHz, $CDCl_3$): $\delta$ (ppm) 8.60 (s, 1H), 7.82–7.76 (m, 3H), 7.66 (dd, *J* = 8.4, 2.9 Hz, 2H), 7.43 (d, *J* = 7.8 Hz, 1H), 7.26–7.19 (m, 5H), 7.15–7.10 (m, 1H), 2.66 (q, *J* = 7.6 Hz, 2H), 2.32 (s, 3H), 1.26 (t, *J* = 7.6 Hz, 3H). $^{13}$C-NMR (126 MHz, $CDCl_3$): $\delta$ (ppm) 148.24, 146.28, 142.48, 139.71, 136.04, 134.25, 130.33, 130.10, 128.68, 126.92, 125.16, 122.17, 119.65, 117.75, 112.79, 28.37, 21.69, 15.87. ESI-HRMS: m/z: found 392.14307 $[M+H]^+$ (calcd for $[C_{22}H_{22}N_3O_2S]^+$ 392.14327).

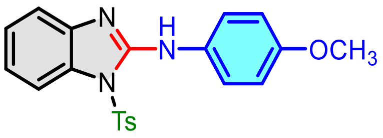

**N-(4-methoxyphenyl)-1-tosyl-1H-benzo[d]imidazol-2-amine**[11] (**6q**): was synthesized according to general procedure D by using 4-metohxyphenyl isothiocyanate (**4q**) [CAS NO. 2284-20-0] (1.0 eq, 0.40 mmol), *o*-phenylenediamine (**1a**) [CAS NO. 95-54-5] (1.2 eq, 0.48 mmol, 52 mg), tosyl chloride (**2a**) [CAS NO. 98-59-9] (1.2 eq, 0.48 mmol, 92 mg) and potassium carbonate [CAS NO. 584-08-7] (1.2 eq & 1.0 eq) in co-solvent between 90% ethanol and 10% water (3.00 mL) to afford **6q** (127.9 mg, 0.324 mmol, 81% yield) as a white solid. $^1$H-NMR (500 MHz, $CDCl_3$): $\delta$ (ppm) 8.48 (s, 1H), 7.80 (d, *J* = 8.5 Hz, 2H), 7.76 (d, *J* = 8.0 Hz, 1H), 7.65–7.61 (m, 2H), 7.40 (d, *J* = 7.9 Hz, 1H), 7.21 (ddd, *J* = 16.9, 8.2, 0.8 Hz, 3H), 7.14–7.09 (m, 1H), 6.97–6.93 (m, 2H), 3.82 (s, 3H), 2.34 (s, 3H). $^{13}$C-NMR (126 MHz, $CDCl_3$): $\delta$ (ppm) 1156.16, 148.73, 146.28, 142.57, 134.28, 131.59, 130.43, 130.32, 126.93, 125.13, 122.04, 121.53, 117.65, 114.56, 112.76, 55.64, 21.71. ESI-HRMS: m/z: found 394.12167 $[M+H]^+$ (calcd for $[C_{21}H_{20}N_3O_3S]^+$ 394.12254).

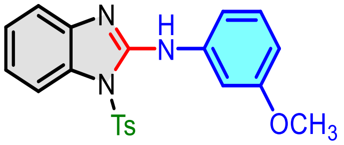

**N-(3-methoxyphenyl)-1-tosyl-1H-benzo[d]imidazol-2-amine**[11] (**6r**): was synthesized according to general procedure D by using 3-metohxyphenyl isothiocyanate (**4r**) [CAS NO. 3125-64-2] (1.0 eq, 0.40 mmol), *o*-phenylenediamine (**1a**) [CAS NO. 95-54-5] (1.2 eq, 0.48 mmol, 52 mg), tosyl chloride (**2a**) [CAS NO. 98-59-9] (1.2 eq, 0.48 mmol, 92 mg) and potassium carbonate [CAS NO. 584-08-7] (1.2 eq & 1.0 eq) in co-solvent between 90% ethanol and 10% water (3.00 mL) to afford **6r** (125.5 mg, 0.32 mmol, 80% yield) as a colorless oil. $^1$H-NMR (500 MHz, $CDCl_3$): $\delta$ (ppm) 8.68 (s, 1H), 7.79–7.75 (m, 3H), 7.51 (t, *J* = 2.2 Hz, 1H), 7.44–7.41 (m, 1H), 7.27 (ddd, *J* = 11.0, 7.5, 4.6 Hz, 2H), 7.23–7.20 (m, 3H), 7.15–7.11 (m, 1H), 6.67 (ddd, *J* = 7.9, 2.4, 1.1 Hz, 1H), 3.85 (s, 3H), 2.32 (s, 3H). $^{13}$C-NMR (126 MHz, $CDCl_3$): $\delta$ (ppm) 160.52, 147.72, 146.37, 142.44, 139.68, 134.21, 130.38, 130.06, 129.99, 126.94, 125.21, 122.40, 117.98, 112.85, 111.57, 108.89, 105.25, 55.48, 21.74. ESI-HRMS: m/z: found 394.12252 $[M+H]^+$ (calcd for $[C_{21}H_{20}N_3O_3S]^+$ 394.12254 ).

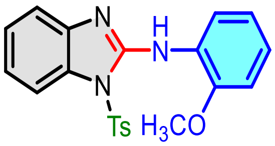


**$N$-(2-methoxyphenyl)-1-tosyl-1$H$-benzo[$d$]imidazol-2-amine**[11] (**6s**): was synthesized according to general procedure D by using 2-methoxyphenyl isothiocyanate (**4s**) [CAS NO. 3288-04-8] (1.0 eq, 0.40 mmol), *o*-phenylenediamine (**1a**) [CAS NO. 95-54-5] (1.2 eq, 0.48 mmol, 52 mg), tosyl chloride (**2a**) [CAS NO. 98-59-9] (1.2 eq, 0.48 mmol, 92 mg) and potassium carbonate [CAS NO. 584-08-7] (1.2 eq & 1.0 eq) in co-solvent between 90% ethanol and 10% water (3.00 mL) to afford **6s** (130.3 mg, 0.332 mmol, 83% yield) as a colorless oil. $^1$H-NMR (500 MHz, $CDCl_3$): $\delta$ (ppm) 9.44 (s, 1H), 8.66–8.60 (m, 1H), 7.84 (d, $J$ = 8.4 Hz, 3H), 7.46 (dd, $J$ = 7.7, 0.4 Hz, 1H), 7.23 (td, $J$ = 7.7, 1.2 Hz, 1H), 7.19 (d, $J$ = 8.3 Hz, 2H), 7.15 (td, $J$ = 8.0, 1.1 Hz, 1H), 7.09–7.04 (m, 2H), 6.98–6.95 (m, 1H), 4.02 (s, 3H), 2.30 (s, 3H). $^{13}$C-NMR (126 MHz, $CDCl_3$): $\delta$ (ppm) 148.11, 147.70, 146.16, 142.54, 134.32, 130.24, 130.02, 128.33, 126.91, 125.04, 122.73, 122.21, 121.26, 118.56, 117.75, 112.75, 110.04, 56.11, 21.64. ESI-HRMS: m/z: found 394.12207 $[M+H]^+$ (calcd for $[C_{21}H_{20}N_3O_3S]^+$ 394.12254).

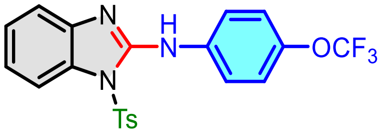


**1-tosyl-$N$-(4-(trifluoromethoxy)phenyl)-1$H$-benzo[$d$]imidazol-2-amine** (**6t**): was synthesized according to general procedure D by using 4-(trifluoromethoxy)phenyl isothiocyanate (**4t**) [CAS NO. 64285-95-6] (1.0 eq, 0.40 mmol), *o*-phenylenediamine (**1a**) [CAS NO. 95-54-5] (1.2 eq, 0.48 mmol, 52 mg), tosyl chloride (**2a**) [CAS NO. 98-59-9] (1.2 eq, 0.48 mmol, 92 mg) and potassium carbonate [CAS NO. 584-08-7] (1.2 eq & 1.0 eq) in co-solvent between 90% ethanol and 10% water (3.00 mL) to afford **6t** (142.3 mg, 0.32 mmol, 80% yield) as a white solid. $^1$H-NMR (500 MHz, $CDCl_3$): $\delta$ (ppm) 8.73 (s, 1H), 7.79 (dd, $J$ = 16.5, 8.2 Hz, 5H), 7.44 (d, $J$ = 7.9 Hz, 1H), 7.27–7.21 (m, 5H), 7.15 (t, $J$ = 7.7 Hz, 1H), 2.33 (s, 3H). $^{13}$C-NMR (126 MHz, $CDCl_3$): $\delta$ (ppm) 147.57, 146.54, 144.66, 142.12, 137.25, 134.14, 130.43, 130.03, 126.93, 125.31, 122.64, 122.19, 120.66 (q, $J$ = 255.92 Hz), 120.25, 118.00, 112.86, 21.71. $^{19}$F-NMR (471 MHz, $CDCl_3$): $\delta$ (ppm) -57.99. Melting point: 136-138°C. ESI-HRMS: m/z: found 448.09311 $[M+H]^+$ (calcd for $[C_{21}H_{17}F_3N_3O_3S]^+$ 448.09427).

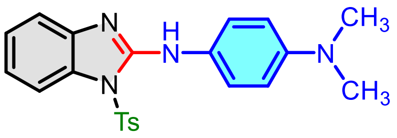


***N*¹,*N*¹-dimethyl-*N*⁴-(1-tosyl-1*H*-benzo[*d*]imidazol-2-yl)benzene-1,4-diamine** (**6u**): was synthesized according to general procedure D using 4-isothiocyanato-*N*,*N*-dimethylaniline (**4u**) (1.0 eq, 0.40 mmol), *o*-phenylenediamine (**1a**) [CAS NO. 95-54-5] (1.2 eq, 0.48 mmol, 52 mg), tosyl chloride (**2a**) [CAS NO. 98-59-9] (1.2 eq, 0.48 mmol, 92 mg) and potassium carbonate [CAS NO. 584-08-7] (1.2 eq & 1.0 eq) in co-solvent between 90% ethanol and 10% water (3.00 mL) to afford **6u** (100.6 mg, 0.248 mmol, 62% yield) as a yellow oil. $^{1}$H-NMR (500 MHz, $CDCl_3$): $\delta$ (ppm) 8.36 (s, 1H), 7.83–7.79 (m, 2H), 7.76 (d, $J$ = 7.9 Hz, 1H), 7.57–7.50 (m, 2H), 7.38 (dd, $J$ = 7.9, 0.5 Hz, 1H), 7.25–7.22 (m, 2H), 7.19 (td, $J$ = 7.7, 1.2 Hz, 1H), 7.12–7.08 (m, 1H), 6.84–6.78 (m, 2H), 2.95 (s, 6H), 2.34 (s, 3H). $^{13}$C-NMR (126 MHz, $CDCl_3$): $\delta$ (ppm) 149.23, 147.83, 146.18, 142.80, 134.35, 130.32, 130.30, 128.37, 126.95, 125.07, 121.91, 121.77, 117.53, 113.71, 112.72, 41.22, 21.73. ESI-HRMS: m/z: found 407.15257 [M+H]$^+$ (calcd for $[C_{22}H_{23}N_4O_2S]^+$ 407.15417).

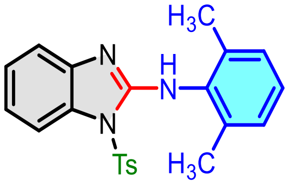


***N*-(2,6-dimethylphenyl)-1-tosyl-1*H*-benzo[*d*]imidazol-2-amine** (**6v**): was synthesized according to general procedure D by using 2,6-dimethylphenyl isothiocyanate (**4v**) [CAS NO. 19241-16-8] (1.0 eq, 0.40 mmol), *o*-phenylenediamine (**1a**) [CAS NO. 95-54-5] (1.2 eq, 0.48 mmol, 52 mg), tosyl chloride (**2a**) [CAS NO. 98-59-9] (1.2 eq, 0.48 mmol, 92 mg) and potassium carbonate [CAS NO. 584-08-7] (1.2 eq & 1.0 eq) in co-solvent between 90% ethanol and 10% water (3.00 mL) to afford **6v** (36.4 mg, 0.092 mmol, 23% yield) as a white solid. $^{1}$H-NMR (500 MHz, $CDCl_3$): $\delta$ (ppm) 7.93 (d, $J$ = 8.4 Hz, 2H), 7.81 (d, $J$ = 7.6 Hz, 1H), 7.76 (s, 1H), 7.32 (dd, $J$ = 7.4, 5.2 Hz, 3H), 7.19 – 7.10 (m, 5H), 2.41 (s, 3H), 2.22 (s, 6H). $^{13}$C-NMR (126 MHz, $CDCl_3$): $\delta$ (ppm) 149.57, 146.40, 142.46, 135.38, 134.76, 134.64, 131.03, 130.34, 128.82, 127.55, 127.17, 124.90, 121.74, 117.71, 112.44, 21.84, 18.65. Melting point: 174-176°C. ESI-HRMS: m/z: found 392.14327 [M+H]$^+$ (calcd for $[C_{22}H_{22}N_3O_2S]^+$ 392.14406).

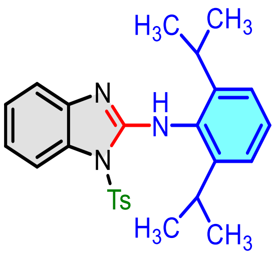


**N-(2,6-diisopropylphenyl)-1-tosyl-1H-benzo[d]imidazol-2-amine** (**6w**): was synthesized according to general procedure D by using 1,3-diisopropylphenyl isothiocyanate (**4w**) [CAS NO. 25343-70-8] (1.0 eq, 0.40 mmol), *o*-phenylenediamine (**1a**) [CAS NO. 95-54-5] (1.2 eq, 0.48 mmol, 52 mg), tosyl chloride (**2a**) [CAS NO. 98-59-9] (1.2 eq, 0.48 mmol, 92 mg) and potassium carbonate [CAS NO. 584-08-7] (1.2 eq & 1.0 eq) in co-solvent between 90% ethanol and 10% water (3.00 mL) to afford **6w** (14.1 mg, 0.032 mmol, 8% yield) as a white solid. $^{1}$H-NMR (500 MHz, $CDCl_3$): $\delta$ (ppm) 7.94–7.90 (m, 2H), 7.80 (dd, *J* = 8.1, 0.6 Hz, 1H), 7.71 (s, 1H), 7.36–7.29 (m, 4H), 7.23 (d, *J* = 7.7 Hz, 2H), 7.16 (td, *J* = 7.7, 1.2 Hz, 1H), 7.10 (td, *J* = 7.8, 1.2 Hz, 1H), 3.07–2.97 (m, 2H), 2.43 (s, 3H), 1.20 (d, *J* = 6.8 Hz, 6H), 1.14 (d, *J* = 6.8 Hz, 6H). $^{13}$C-NMR (126 MHz, $CDCl_3$): $\delta$ (ppm) 151.08, 146.34, 146.22, 142.58, 134.90, 131.95, 131.17, 130.34, 128.73, 127.11, 124.90, 124.10, 121.61, 117.76, 112.41, 28.86, 24.41, 23.17, 21.85. Melting point: 150-153°C. ESI-HRMS: m/z: found 448.20635 $[M+H]^+$ (calcd for $[C_{26}H_{30}N_3O_2S]^+$ 448.20587).

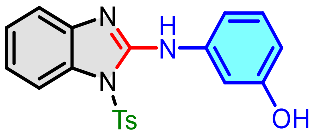


**3-((1-tosyl-1H-benzo[d]imidazol-2-yl)amino)phenol** (**6y**): was synthesized according to general procedure D by using 3-hydroxyphenyl isothiocyanate (**4y**) (1.0 eq, 0.40 mmol), *o*-phenylenediamine (**1a**) [CAS NO. 95-54-5] (1.2 eq, 0.48 mmol, 52 mg), tosyl chloride (**2a**) [CAS NO. 98-59-9] (1.2 eq, 0.48 mmol, 92 mg) and potassium carbonate [CAS NO. 584-08-7] (1.2 eq & 1.0 eq) in co-solvent between 90% ethanol and 10% water (3.00 mL) to afford **6y** (52.6 mg, 0.14 mmol, 35% yield) as a white solid. $^{1}$H-NMR (500 MHz, acetone-$d_6$): $\delta$ (ppm) 8.79 (s), 8.57 (s), 7.95–7.92 (m), 7.82 (ddd, *J* = 8.0, 1.0, 0.5 Hz), 7.61 (t, *J* = 2.1 Hz), 7.40–7.35 (m), 7.21 (dddd, *J* = 15.5, 9.1, 7.1, 1.2 Hz), 6.62 (ddd, *J* = 7.8, 2.3, 1.1 Hz), 2.33 (s). $^{13}$C-NMR (126 MHz, acetone-$d_6$), $\delta$ (ppm) 158.92, 148.54, 147.68, 143.47, 140.79, 134.75, 131.29, 130.73, 130.51, 127.81, 125.99,

123.05, 118.34, 113.72, 111.12, 110.97, 107.05, 21.44. Melting point: 185-188°C. ESI-HRMS: m/z: found 380.10509 $[M+H]^+$ (calcd for $[C_{20}H_{18}N_3O_3S]^+$ 380.10689).

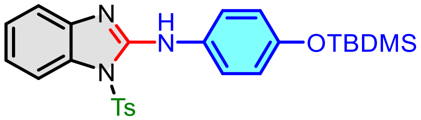


**N-(4-((tert-butyldimethylsilyl)oxy)phenyl)-1-tosyl-1H-benzo[d]imidazol-2-amine**[11] (**6z**): was synthesized according to general procedure D by using *tert*-butyl(4-isothiocyanatophenoxy)dimethylsilane (**4z**) (1.0 eq, 0.40 mmol), *o*-phenylenediamine (**1a**) [CAS NO. 95-54-5] (1.2 eq, 0.48 mmol, 52 mg), tosyl chloride (**2a**) [CAS NO. 98-59-9] (1.2 eq, 0.48 mmol, 92 mg) and potassium carbonate [CAS NO. 584-08-7] (1.2 eq & 1.0 eq) in co-solvent between 90% ethanol and 10% water (3.00 mL) to afford **6z** (131.0 mg, 0.264 mmol, 66% yield) as a colorless oil. $^1$H-NMR (500 MHz, $CDCl_3$): $\delta$ (ppm) 8.50 (s, 1H), 7.80 (d, $J$ = 8.4 Hz, 2H), 7.75 (d, $J$ = 8.0 Hz, 1H), 7.62–7.57 (m, 2H), 7.40 (d, $J$ = 7.9 Hz, 1H), 7.26–7.18 (m, 3H), 7.11 (t, $J$ = 7.7 Hz, 1H), 6.88 (d, $J$ = 8.6 Hz, 2H), 2.35 (s, 3H), 1.01 (s, 9H), 0.22 (s, 6H). $^{13}$C-NMR (126 MHz, $CDCl_3$): $\delta$ (ppm) 151.95, 148.52, 146.30, 142.59, 134.30, 132.15, 130.37, 130.17, 126.98, 125.16, 122.08, 121.04, 120.70, 117.69, 112.78, 25.83, 21.77, 18.34, -4.30. ESI-HRMS: m/z: found 494.19190 $[M+H]^+$ (calcd for $[C_{26}H_{32}N_3O_3SSi]^+$ 494.19337).

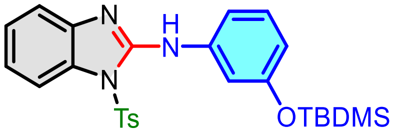


**N-(3-((tert-butyldimethylsilyl)oxy)phenyl)-1-tosyl-1H-benzo[d]imidazol-2-amine**[11] (**6a'**): was synthesized according to general procedure D by using *tert*-butyl(3-isothiocyanatophenoxy)dimethylsilane (**4a'**) (1.0 eq, 0.40 mmol), *o*-phenylenediamine (**1a**) [CAS NO. 95-54-5] (1.2 eq, 0.48 mmol, 52 mg), tosyl chloride (**2a**) [CAS NO. 98-59-9] (1.2 eq, 0.48 mmol, 92 mg) and potassium carbonate [CAS NO. 584-08-7] (1.2 eq & 1.0 eq) in co-solvent between 90% ethanol and 10% water (3.00 mL) to afford **6a'** (133.1 mg, 0.268 mmol, 67% yield) as a colorless oil. $^1$H-NMR (500 MHz, $CDCl_3$): $\delta$ (ppm) 8.64 (s, 1H), 7.79 (t, $J$ = 8.7 Hz, 3H), 7.48–7.41 (m, 2H), 7.32 (dd, $J$ = 8.1, 1.0 Hz, 1H), 7.27–7.21 (m, 4H), 7.15 (t, $J$ = 7.7 Hz, 1H), 6.63 (dd, $J$ = 8.0, 0.9 Hz, 1H), 2.34 (s, 3H), 1.04 (s, 9H), 0.29 (s, 6H). $^{13}$C-NMR (126 MHz, $CDCl_3$): $\delta$ (ppm) 156.57, 147.73, 146.33, 142.50, 139.55, 134.23, 130.37, 130.01, 129.94, 126.96, 125.17, 122.35, 117.92,

115.09, 112.83, 112.10, 111.11, 25.85, 21.74, 18.37, -4.27. ESI-HRMS: m/z: found 494.19281 $[M+H]^+$ (calcd for $[C_{26}H_{32}N_3O_3SSi]^+$ 494.19337).

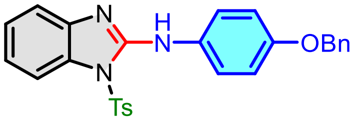


**N-(4-(benzyloxy)phenyl)-1-tosyl-1*H*-benzo[*d*]imidazol-2-amine** (**6b'**): was synthesized according to general procedure D by using 1-(benzyloxy)-4-isothiocyanatobenzene (**4b'**) [CAS NO. 6590-96-1] (1.0 eq, 0.40 mmol), *o*-phenylenediamine (**1a**) [CAS NO. 95-54-5] (1.2 eq, 0.48 mmol, 52 mg), tosyl chloride (**2a**) [CAS NO. 98-59-9] (1.2 eq, 0.48 mmol, 92 mg) and potassium carbonate [CAS NO. 584-08-7] (1.2 eq & 1.0 eq) in co-solvent between 90% ethanol and 10% water (3.00 mL) to afford **6b'** (143.9 mg, 0.308 mmol, 77% yield) as a pale yellow solid. $^1$H-NMR (500 MHz, $CDCl_3$): $\delta$ (ppm) 8.51 (s, 1H), 7.81 (d, $J$ = 8.4 Hz, 2H), 7.78 (d, $J$ = 8.0 Hz, 1H), 7.67–7.62 (m, 2H), 7.46 (d, $J$ = 7.4 Hz, 2H), 7.41 (t, $J$ = 7.4 Hz, 3H), 7.35 (d, $J$ = 7.3 Hz, 1H), 7.22 (dd, $J$ = 14.3, 7.9 Hz, 3H), 7.13 (t, $J$ = 7.7 Hz, 1H), 7.07–7.01 (m, 2H), 5.09 (s, 2H), 2.35 (s, 3H). $^{13}$C-NMR (126 MHz, $CDCl_3$): $\delta$ (ppm) 155.30, 148.62, 146.30, 142.49, 137.09, 134.25, 131.80, 130.35, 130.18, 128.68, 128.06, 127.55, 126.93, 125.16, 122.09, 121.42, 117.66, 115.64, 112.77, 70.43, 21.73. Melting point: 120-123°C. ESI-HRMS: m/z: found 470.15276 $[M+H]^+$ (calcd for $[C_{27}H_{24}N_3O_3S]^+$ 470.15384).

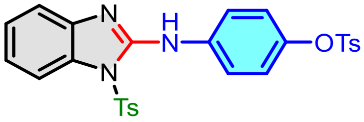


**4-((1-tosyl-1*H*-benzo[*d*]imidazol-2-yl)amino)phenyl 4-methylbenzenesulfonate** (**6c'**): was synthesized according to general procedure D by using 4-isothiocyanatophenyl 4-methylbenzenesulfonate (**4c'**) (1.0 eq, 0.40 mmol), *o*-phenylenediamine (**1a**) [CAS NO. 95-54-5] (1.2 eq, 0.48 mmol, 52 mg), tosyl chloride (**2a**) [CAS NO. 98-59-9] (1.2 eq, 0.48 mmol, 92 mg) and potassium carbonate [CAS NO. 584-08-7] (1.2 eq & 1.0 eq) in co-solvent between 90% ethanol and 10% water (3.00 mL) to afford **6c'** (126.2 mg, 0.236 mmol, 59% yield) as a white solid. $^1$H-NMR (500 MHz, $CDCl_3$): $\delta$ (ppm) 8.73 (s, 1H), 7.77–7.69 (m, 7H), 7.41 (d, $J$ = 7.7 Hz, 1H), 7.32 (d, $J$ = 8.1 Hz, 2H), 7.25–7.20 (m, 3H), 7.14 (td, $J$ = 7.8, 1.2 Hz, 1H), 7.02–6.99 (m, 2H), 2.45 (s, 3H), 2.34 (s, 3H). $^{13}$C-NMR (126 MHz, $CDCl_3$): $\delta$ (ppm) 147.34, 146.57, 145.51, 144.97, 141.93, 137.34, 134.05, 132.36, 130.45, 129.91, 129.88, 128.66, 126.93, 125.31, 123.31, 122.68, 119.84, 117.90, 112.83,

21.83, 21.76. Melting point: 158-161°C. ESI-HRMS: m/z: found 534.11254 $[M+H]^+$ (calcd for $[C_{27}H_{24}N_3O_5S_2]^+$ 534.11574).

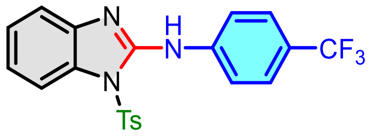

**1-tosyl-*N*-(4-(trifluoromethyl)phenyl)-1*H*-benzo[*d*]imidazol-2-amine**[11] (**6d'**): was synthesized according to general procedure D by using 4-(trifluoromethyl)phenyl isothiocyanate (**4d'**) [CAS NO. 1645-65-4] (1.0 eq, 0.40 mmol), *o*-phenylenediamine (**1a**) [CAS NO. 95-54-5] (1.2 eq, 0.48 mmol, 52 mg), tosyl chloride (**2a**) [CAS NO. 98-59-9] (1.2 eq, 0.48 mmol, 92 mg) and potassium carbonate [CAS NO. 584-08-7] (1.2 eq & 1.0 eq) in co-solvent between 90% ethanol and 10% water (3.00 mL) to afford **6d'** (91.5 mg, 0.212 mmol, 53% yield) as a white solid. $^1$H-NMR (500 MHz, $CDCl_3$): $\delta$ (ppm) 8.90 (s, 1H), 7.91 (d, $J$ = 8.5 Hz, 2H), 7.78 (d, $J$ = 8.4 Hz, 3H), 7.64 (d, $J$ = 8.6 Hz, 2H), 7.47 (d, $J$ = 7.9 Hz, 1H), 7.27–7.21 (m, 3H), 7.20–7.15 (m, 1H), 2.32 (s, 3H). $^{13}$C-NMR (126 MHz, $CDCl_3$): $\delta$ (ppm) 146.97, 146.62, 141.98, 141.55, 134.06, 130.45, 129.92, 126.92, 126.60 (q, $J$ = 3.92 Hz), 125.47, 124.93 (q, $J$ = 33.80 Hz), 124.38 (q, $J$ = 272.16 Hz), 122.92, 118.60, 118.21, 112.91, 21.70. $^{19}$F-NMR (471 MHz, $CDCl_3$): $\delta$ (ppm) -61.68. ESI-HRMS: m/z: found 432.09785 $[M+H]^+$ (calcd for $[C_{21}H_{17}F_3N_3O_2S]^+$ 432.09936).

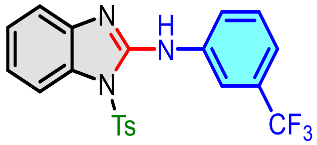

**1-tosyl-*N*-(3-(trifluoromethyl)phenyl)-1*H*-benzo[*d*]imidazol-2-amine**[11] (**6e'**): was synthesized according to general procedure D by using 3-(trifluoromethyl)phenyl isothiocyanate (**4e'**) [CAS NO. 23165-29-9] (1.0 eq, 0.40 mmol), *o*-phenylenediamine (**1a**) [CAS NO. 95-54-5] (1.2 eq, 0.48 mmol, 52 mg), tosyl chloride (**2a**) [CAS NO. 98-59-9] (1.2 eq, 0.48 mmol, 92 mg) and potassium carbonate [CAS NO. 584-08-7] (1.2 eq & 1.0 eq) in co-solvent between 90% ethanol and 10% water (3.00 mL) to afford **6e'** (130.3 mg, 0.304 mmol, 76% yield) as a white solid. $^1$H-NMR (500 MHz, $CDCl_3$): $\delta$ (ppm) 8.84 (s, 1H), 8.11 (s, 1H), 7.99 (dd, $J$ = 8.2, 1.6 Hz, 1H), 7.80–7.76 (m, 3H), 7.52–7.45 (m, 2H), 7.37–7.34 (m, 1H), 7.24 (dd, $J$ = 12.3, 4.2 Hz, 3H), 7.17 (ddd, $J$ = 8.1, 7.7, 0.7 Hz, 1H), 2.33 (s, 3H). $^{13}$C-NMR (126 MHz, $CDCl_3$): $\delta$ (ppm) 147.19, 146.60, 142.00, 139.07, 134.09, 131.68 (q, $J$ = 32.44 Hz), 129.94, 129.88, 126.94, 125.35, 124.09 (q, $J$ = 272.51 Hz), 122.81, 122.11,

119.86 (q, $J$ = 3.63 Hz), 118.20, 115.76 (q, $J$ = 3.81 Hz), 112.86, 21.72. $^{19}$F-NMR (471 MHz, $CDCl_3$): $\delta$ (ppm) -62.44. ESI-HRMS: m/z: found 432.09820 $[M+H]^+$ (calcd for $[C_{21}H_{17}F_3N_3O_2S]^+$ 432.09936).

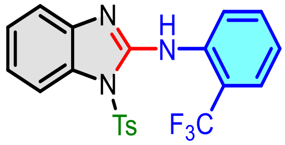


**1-tosyl-*N*-(2-(trifluoromethyl)phenyl)-1*H*-benzo[*d*]imidazol-2-amine** (**6f'**): was synthesized according to general procedure D by using 2-(trifluoromethyl)phenyl isothiocyanate (**4f'**) [CAS NO. 1743-86-8] (1.0 eq, 0.40 mmol), *o*-phenylenediamine (**1a**) [CAS NO. 95-54-5] (1.2 eq, 0.48 mmol, 52 mg), tosyl chloride (**2a**) [CAS NO. 98-59-9] (1.2 eq, 0.48 mmol, 92 mg) and potassium carbonate [CAS NO. 584-08-7] (1.2 eq & 1.0 eq) in co-solvent between 90% ethanol and 10% water (3.00 mL) to afford **6f'** (149.6 mg, 0.348 mmol, 87% yield) as a white solid. $^1$H-NMR (500 MHz, $CDCl_3$): $\delta$ (ppm) 9.16 (s, 1H), 8.70 (d, $J$ = 8.4 Hz, 1H), 7.88–7.85 (m, 2H), 7.85–7.82 (m, 1H), 7.69 (d, $J$ = 7.9 Hz, 1H), 7.63 (t, $J$ = 7.9 Hz, 1H), 7.47–7.44 (m, 1H), 7.27–7.17 (m, 5H), 2.35 (s, 3H). $^{13}$C-NMR (126 MHz, $CDCl_3$): $\delta$ (ppm) 147.51, 146.48, 141.68, 136.40, 134.22, 133.27, 130.39, 130.18, 127.05, 126.39 (q, $J$ = 5.12 Hz), 125.21, 124.32 (q, $J$ = 272.82 Hz), 123.28, 122.80, 122.45, 119.40 (q, $J$ = 29.89 Hz), 112.84, 21.73. $^{19}$F-NMR (471 MHz, $CDCl_3$): $\delta$ (ppm) -60.88. Melting point: 138-141$^{\circ}$C. ESI-HRMS: m/z: found 432.09814 $[M+H]^+$ (calcd for $[C_{21}H_{17}F_3N_3O_2S]^+$ 432.09936).

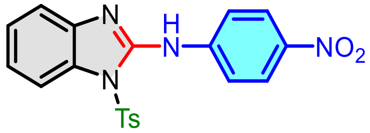


***N*-(4-nitrophenyl)-1-tosyl-1*H*-benzo[*d*]imidazol-2-amine**[11] (**6g'**): was synthesized according to general procedure D by using 4-nitrophenyl isothiocyanate (**4g'**) [CAS NO. 2131-61-5] (1.0 eq, 0.40 mmol), *o*-phenylenediamine (**1a**) [CAS NO. 95-54-5] (1.2 eq, 0.48 mmol, 52 mg), tosyl chloride (**2a**) [CAS NO. 98-59-9] (1.2 eq, 0.48 mmol, 92 mg) and potassium carbonate [CAS NO. 584-08-7] (1.2 eq & 1.0 eq) in co-solvent between 90% ethanol and 10% water (3.00 mL) to afford **6g'** (40.9 mg, 0.10 mmol, 25% yield) as a yellow solid. $^1$H-NMR (500 MHz, $CDCl_3$): $\delta$ (ppm) 9.12 (s, 1H), 8.29–8.26 (m, 2H), 7.97–7.94 (m, 2H), 7.78–7.75 (m, 3H), 7.50–7.48 (m, 1H), 7.29–7.24 (m, 3H), 7.22–7.18 (m, 1H), 2.35 (s, 3H). $^{13}$C-NMR (126 MHz, $CDCl_3$): $\delta$ (ppm) 146.89, 146.14, 144.27, 142.72, 141.63, 133.91, 130.60, 129.83, 126.99, 125.59, 123.46, 118.54, 118.21, 112.96, 21.85. ESI-HRMS: m/z: found 409.09485 $[M+H]^+$ (calcd for $[C_{20}H_{17}N_4O_4S]^+$ 409.09705).

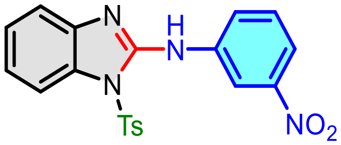


***N*-(3-nitrophenyl)-1-tosyl-1*H*-benzo[*d*]imidazol-2-amine**[11] (**6h'**): was synthesized according to general procedure D by using 3-nitrophenyl isothiocyanate (**4h'**) [CAS NO. 3529-82-6] (1.0 eq, 0.40 mmol), *o*-phenylenediamine (**1a**) [CAS NO. 95-54-5] (1.2 eq, 0.48 mmol, 52 mg), tosyl chloride (**2a**) [CAS NO. 98-59-9] (1.2 eq, 0.48 mmol, 92 mg) and potassium carbonate [CAS NO. 584-08-7] (1.2 eq & 1.0 eq) in co-solvent between 90% ethanol and 10% water (3.00 mL) to afford **6h'** (84.6 mg, 0.208 mmol, 52% yield) as a yellow solid. $^1$H-NMR (500 MHz, $CDCl_3$): $\delta$ (ppm) 8.92 (s, 1H), 8.76 (t, *J* = 2.2 Hz, 1H), 8.08 (ddd, *J* = 8.1, 2.2, 0.8 Hz, 1H), 7.91 (ddd, *J* = 8.2, 2.2, 0.8 Hz, 1H), 7.79–7.77 (m, 2H), 7.76–7.74 (m, 1H), 7.52 (t, *J* = 8.2 Hz, 1H), 7.47 (d, *J* = 7.6 Hz, 1H), 7.26–7.22 (m, 3H), 7.17 (td, *J* = 7.8, 1.2 Hz, 1H), 2.33 (s, 3H). $^{13}$C-NMR (126 MHz, $CDCl_3$): $\delta$ (ppm) 148.92, 146.77, 146.72, 141.73, 139.65, 133.93, 130.50, 130.04, 129.85, 126.93, 125.41, 124.55, 123.04, 118.34, 117.82, 113.70, 112.82, 21.77. ESI-HRMS: m/z: found 409.09459 $[M+H]^+$ (calcd for $[C_{20}H_{17}N_4O_4S]^+$ 409.09705).

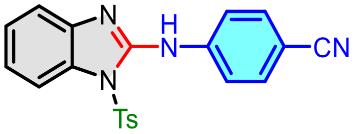


**4-((1-tosyl-1*H*-benzo[*d*]imidazol-2-yl)amino)benzonitrile**[11] (**6i'**): was synthesized according to general procedure D by using 4-cyanophenyl isothiocyanate (**4i'**) [CAS NO. 2719-32-6] (1.0 eq, 0.40 mmol), *o*-phenylenediamine (**1a**) [CAS NO. 95-54-5] (1.2 eq, 0.48 mmol, 52 mg), tosyl chloride (**2a**) [CAS NO. 98-59-9] (1.2 eq, 0.48 mmol, 92 mg) and potassium carbonate [CAS NO. 584-08-7] (1.2 eq & 1.0 eq) in co-solvent between 90% ethanol and 10% water (3.00 mL) to afford **6i'** (36.2 mg, 0.092 mmol, 23% yield) as a white solid. $^1$H-NMR (500 MHz, $CDCl_3$): $\delta$ (ppm) 8.99 (s, 1H), 7.93 (d, *J* = 8.8 Hz, 2H), 7.77 (d, *J* = 8.4 Hz, 3H), 7.68 (d, *J* = 8.8 Hz, 2H), 7.48 (d, *J* = 7.7 Hz, 1H), 7.26 (dd, *J* = 7.9, 7.4 Hz, 3H), 7.19 (td, *J* = 7.9, 1.2 Hz, 1H), 2.36 (s, 3H). $^{13}$C-NMR (126 MHz, $CDCl_3$): $\delta$ (ppm) 146.79, 146.38, 142.44, 141.76, 133.99, 133.64, 130.56, 129.83, 126.97, 125.51, 123.27, 119.25, 118.79, 118.41, 112.95, 105.89, 21.83. ESI-HRMS: m/z: found 389.10475 $[M+H]^+$ (calcd for $[C_{21}H_{17}N_4O_2S]^+$ 389.10722).

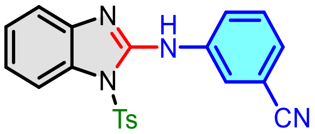


**3-((1-tosyl-1*H*-benzo[*d*]imidazol-2-yl)amino)benzonitrile** (**6j'**): was synthesized according to general procedure D by using 3-cyanophenyl isothiocyanate (**4j'**) [CAS NO. 3125-78-8] (1.0 eq, 0.40 mmol), *o*-phenylenediamine (**1a**) [CAS NO. 95-54-5] (1.2 eq, 0.48 mmol, 52 mg), tosyl chloride (**2a**) [CAS NO. 98-59-9] (1.2 eq, 0.48 mmol, 92 mg) and potassium carbonate [CAS NO. 584-08-7] (1.2 eq & 1.0 eq) in co-solvent between 90% ethanol and 10% water (3.00 mL) to afford **6j'** (108.2 mg, 0.28 mmol, 70% yield) as a pale yellow solid. $^{1}$H-NMR (500 MHz, $CDCl_3$): $\delta$ (ppm) 8.82 (s, 1H), 8.35 (d, *J* = 1.5 Hz, 1H), 7.83 (d, *J* = 8.3 Hz, 1H), 7.76 (dd, *J* = 12.1, 5.0 Hz, 3H), 7.46 (ddd, *J* = 8.2, 4.7, 1.5 Hz, 2H), 7.36 (dd, *J* = 7.6, 0.7 Hz, 1H), 7.24 (d, *J* = 7.7 Hz, 3H), 7.17 (t, *J* = 7.8 Hz, 1H), 2.34 (s, 3H). $^{13}$C-NMR (126 MHz, $CDCl_3$): $\delta$ (ppm) 146.79, 146.69, 141.80, 139.32, 133.97, 130.49, 130.05, 129.85, 126.92, 126.66, 125.43, 123.06, 122.99, 121.93, 118.83, 118.25, 113.31, 112.84, 21.78. Melting point: 160-162°C. ESI-HRMS: m/z: found 389.10600 $[M+H]^+$ (calcd for $[C_{21}H_{17}N_4O_2S]^+$ 389.10722).

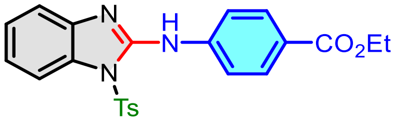


**ethyl 4-((1-tosyl-1*H*-benzo[*d*]imidazol-2-yl)amino)benzoate**[11] (**6k'**): was synthesized according to general procedure D by using ethyl 4-isothiocyanatobenzoate (**4k'**) [CAS NO. 2131-59-1] (1.0 eq, 0.40 mmol), *o*-phenylenediamine (**1a**) [CAS NO. 95-54-5] (1.2 eq, 0.48 mmol, 52 mg), tosyl chloride (**2a**) [CAS NO. 98-59-9] (1.2 eq, 0.48 mmol, 92 mg) and potassium carbonate [CAS NO. 584-08-7] (1.2 eq & 1.0 eq) in co-solvent between 90% ethanol and 10% water (3.00 mL) to afford **6k'** (125.9 mg, 0.288 mmol, 72% yield) as a white solid. $^{1}$H-NMR (500 MHz, $CDCl_3$): $\delta$ (ppm) 8.92 (s, 1H), 8.10–8.06 (m, 2H), 7.87–7.83 (m, 2H), 7.76 (d, *J* = 8.5 Hz, 3H), 7.46 (d, *J* = 7.7 Hz, 1H), 7.26–7.19 (m, 3H), 7.15 (td, *J* = 8.0, 1.1 Hz, 1H), 4.36 (q, *J* = 7.1 Hz, 2H), 2.30 (s, 3H), 1.39 (t, *J* = 7.1 Hz, 3H). $^{13}$C-NMR (126 MHz, $CDCl_3$): $\delta$ (ppm) 166.25, 146.79, 146.53, 142.48, 141.99, 133.99, 131.11, 130.41, 129.83, 126.89, 125.31, 124.87, 122.86, 118.17, 117.98, 112.85, 60.86, 21.71, 14.45. ESI-HRMS: m/z: found 436.13067 $[M+H]^+$ (calcd for $[C_{23}H_{22}N_3O_4S]^+$ 436.13310).

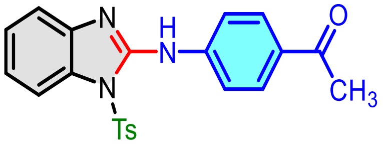


**1-(4-((1-tosyl-1*H*-benzo[*d*]imidazol-2-yl)amino)phenyl)ethan-1-one** (**6l'**): was synthesized according to general procedure D by using 1-(4-isothiocyanatophenyl)ethan-1-one (**4l'**) (1.0 eq, 0.40 mmol), *o*-phenylenediamine (**1a**) [CAS NO. 95-54-5] (1.2 eq, 0.48 mmol, 52 mg), tosyl chloride (**2a**) [CAS NO. 98-59-9] (1.2 eq, 0.48 mmol, 92 mg) and potassium carbonate [CAS NO. 584-08-7] (1.2 eq & 1.0 eq) in co-solvent between 90% ethanol and 10% water (3.00 mL) to afford **6l'** (81.0 mg, 0.20 mmol, 50% yield) as a yellow solid. $^{1}$H-NMR (500 MHz, $CDCl_3$): $\delta$ (ppm) 8.94 (s, 1H), 7.99 (d, *J* = 8.7 Hz, 2H), 7.86 (d, *J* = 8.8 Hz, 2H), 7.78–7.74 (m, 3H), 7.45 (d, *J* = 7.7 Hz, 1H), 7.25–7.15 (m, 4H), 2.57 (s, 3H), 2.30 (s, 3H). $^{13}$C-NMR (126 MHz, $CDCl_3$): $\delta$ (ppm) 196.88, 146.64, 146.57, 142.71, 141.88, 133.91, 131.87, 130.41, 130.09, 129.79, 126.87, 125.32, 122.93, 118.18, 118.06, 112.84, 26.49, 21.70. Melting point: 145-147°C. ESI-HRMS: m/z: found 406.12086 $[M+H]^+$ (calcd for $[C_{22}H_{20}N_3O_3S]^+$ 406.12254).

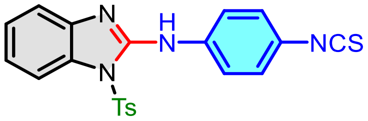


***N*-(4-isothiocyanatophenyl)-1-tosyl-1*H*-benzo[*d*]imidazol-2-amine** (**6m'**): was synthesized according to general procedure D by using 1,4-phenylene diisothiocyanate (**4m'**) [CAS NO. 4044-65-9] (1.0 eq, 0.40 mmol), *o*-phenylenediamine (**1a**) [CAS NO. 95-54-5] (1.2 eq, 0.48 mmol, 52 mg), tosyl chloride (**2a**) [CAS NO. 98-59-9] (1.2 eq, 0.48 mmol, 92 mg) and potassium carbonate [CAS NO. 584-08-7] (1.2 eq & 1.0 eq) in co-solvent between 90% ethanol and 10% water (3.00 mL) to afford **6m'** (54.7 mg, 0.13 mmol, 32% yield) as a white solid. $^{1}$H-NMR (500 MHz, $CDCl_3$): $\delta$ (ppm) 8.78 (s, 1H), 7.78–7.73 (m, 5H), 7.47–7.42 (m, 1H), 7.24 (ddd, *J* = 8.6, 7.0, 5.3 Hz, 5H), 7.17–7.14 (m, 1H), 2.34 (s, 3H). $^{13}$C-NMR (126 MHz, $CDCl_3$): $\delta$ (ppm) 147.10, 146.71, 141.63, 137.42, 134.63, 134.02, 130.53, 129.83, 126.99, 126.86, 125.94, 125.47, 122.93, 119.99, 118.02, 112.90, 21.83. Melting point: 139-142°C. ESI-HRMS: m/z: found 421.07686 $[M+H]^+$ (calcd for $[C_{21}H_{17}N_4O_2S_2]^+$ 421.07930).

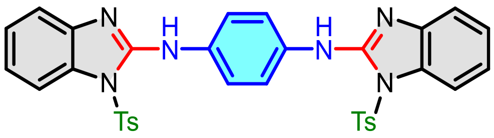

**$N^1$,$N^4$-bis(1-tosyl-1*H*-benzo[*d*]imidazol-2-yl)benzene-1,4-diamine** (**6n'**): was synthesized according to general procedure D by using 1,4-phenylene diisothiocyanate (**4m'**) [CAS NO. 4044-65-9] (1.0 eq, 0.40 mmol), *o*-phenylenediamine (**1a**) [CAS NO. 95-54-5] (1.2 eq, 0.48 mmol, 52 mg), tosyl chloride (**2a**) [CAS NO. 98-59-9] (1.2 eq, 0.48 mmol, 92 mg) and potassium carbonate [CAS NO. 584-08-7] (1.2 eq & 1.0 eq) in co-solvent between 90% ethanol and 10% water (3.00 mL) to afford **6n'** (42.0 mg, 0.065 mmol, 16% yield) as a white solid. $^1$H-NMR (500 MHz, $CDCl_3$): $\delta$ (ppm) 7.82 (d, *J* = 8.4 Hz, 4H), 7.78 (dd, *J* = 8.1, 0.5 Hz, 2H), 7.75 (s, 4H), 7.44 (dd, *J* = 7.8, 0.5 Hz, 2H), 7.29 – 7.26 (m, 4H), 7.23 (td, *J* = 7.7, 1.2 Hz, 2H), 7.17–7.13 (m, 2H), 2.37 (s, 6H). $^{13}$C-NMR (126 MHz, $CDCl_3$): $\delta$ (ppm) 148.16, 146.61, 134.25, 134.12, 130.52, 129.97, 127.07, 125.39, 122.55, 121.09, 117.64, 112.93, 21.84. Melting point: 235-237°C. ESI-HRMS: m/z: found 649.16883 $[M+H]^+$ (calcd for $[C_{21}H_{17}N_4O_2S_2]^+$ 649.16917).

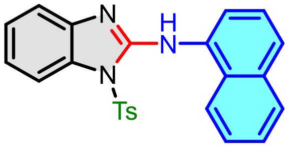

***N*-(naphthalen-1-yl)-1-tosyl-1*H*-benzo[*d*]imidazol-2-amine**[11] (**6o'**): was synthesized according to general procedure D by using 1-naphthyl isothiocyanate (**4o'**) [CAS NO. 551-06-4] (1.0 eq, 0.40 mmol), *o*-phenylenediamine (**1a**) [CAS NO. 95-54-5] (1.2 eq, 0.48 mmol, 52 mg), tosyl chloride (**2a**) [CAS NO. 98-59-9] (1.2 eq, 0.48 mmol, 92 mg) and potassium carbonate [CAS NO. 584-08-7] (1.2 eq & 1.0 eq) in co-solvent between 90% ethanol and 10% water (3.00 mL) to afford **6o'** (142.4 mg, 0.344 mmol, 86% yield) as a colorless oil. $^1$H-NMR (500 MHz, $CDCl_3$): $\delta$ (ppm) 9.40 (s, 1H), 8.58 (d, *J* = 7.0 Hz, 1H), 8.12 (d, *J* = 8.4 Hz, 1H), 7.94 (d, *J* = 7.9 Hz, 1H), 7.90–7.83 (m, 3H), 7.68 (d, *J* = 8.2 Hz, 1H), 7.64 (ddd, *J* = 8.3, 6.8, 1.3 Hz, 1H), 7.60–7.55 (m, 2H), 7.49 (dd, *J* = 4.4, 3.9 Hz, 1H), 7.26 (td, *J* = 7.6, 1.1 Hz, 1H), 7.22 (d, *J* = 8.2 Hz, 2H), 7.19 (td, *J* = 7.8, 1.1 Hz, 1H), 2.33 (s, 3H). $^{13}$C-NMR (126 MHz, $CDCl_3$): $\delta$ (ppm) 148.44, 146.42, 142.37, 134.28, 134.23, 133.56, 130.40, 130.14, 129.09, 126.93, 126.63, 126.27, 126.18, 125.69, 125.24, 124.12, 122.45, 120.04, 118.01, 116.69, 112.80, 21.72. ESI-HRMS: m/z: found 414.12786 $[M+H]^+$ (calcd for $[C_{24}H_{20}N_3O_2S]^+$ 414.12762).

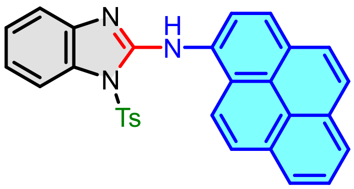

**N-(pyren-1-yl)-1-tosyl-1H-benzo[d]imidazol-2-amine** (**6p'**): was synthesized according to general procedure D by using 1-isothiocyanatopyrene (**4p'**) [CAS NO. 24722-90-5] (1.0 eq, 0.40 mmol), *o*-phenylenediamine (**1a**) [CAS NO. 95-54-5] (1.2 eq, 0.48 mmol, 52 mg), tosyl chloride (**2a**) [CAS NO. 98-59-9] (1.2 eq, 0.48 mmol, 92 mg) and potassium carbonate [CAS NO. 584-08-7] (1.2 eq & 1.0 eq) in co-solvent between 90% ethanol and 10% water (3.00 mL) to afford **6p'** (30.5 mg, 0.064 mmol, 16% yield) as a yellow solid. $^1$H-NMR (500 MHz, $CDCl_3$): $\delta$ (ppm) 9.54 (s, 1H), 8.98 (d, *J* = 8.4 Hz, 1H), 8.25 (t, *J* = 8.3 Hz, 2H), 8.20–8.16 (m, 3H), 8.03 (dt, *J* = 9.3, 8.1 Hz, 3H), 7.92 (d, *J* = 8.5 Hz, 2H), 7.85 (d, *J* = 7.9 Hz, 1H), 7.47 (d, *J* = 7.5 Hz, 1H), 7.26 (dd, *J* = 11.9, 4.6 Hz, 3H), 7.19 (td, *J* = 7.9, 1.1 Hz, 1H), 2.34 (s, 3H). $^{13}$C-NMR (126 MHz, $CDCl_3$): $\delta$ (ppm) 148.83, 146.54, 142.37, 134.34, 131.78, 131.68, 131.05, 130.50, 130.39, 128.33, 128.17, 127.56, 127.06, 126.44, 126.37, 125.84, 125.50, 125.47, 125.33, 125.05, 124.99, 122.49, 121.77, 119.67, 119.25, 118.05, 112.86, 21.81. Melting point: 216-218°C. ESI-HRMS: m/z: found 488.14457 $[M+H]^+$ (calcd for $[C_{30}H_{22}N_3O_2S]^+$ 488.14327).

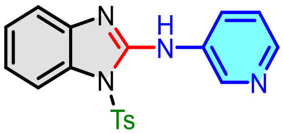

**N-(pyridin-3-yl)-1-tosyl-1H-benzo[d]imidazol-2-amine**[11] (**6q'**): was synthesized according to general procedure D by using 3-pyridyl isothiocyanate (**4q'**) [CAS NO. 17452-27-6] (1.0 eq, 0.40 mmol), *o*-phenylenediamine (**1a**) [CAS NO. 95-54-5] (1.2 eq, 0.48 mmol, 52 mg), tosyl chloride (**2a**) [CAS NO. 98-59-9] (1.2 eq, 0.48 mmol, 92 mg) and potassium carbonate [CAS NO. 584-08-7] (1.2 eq & 1.0 eq) in co-solvent between 90% ethanol and 10% water (3.00 mL) to afford **6q'** (74.2 mg, 0.204 mmol, 51% yield) as a light brown solid. $^1$H-NMR (500 MHz, $CDCl_3$): $\delta$ (ppm) 8.78 (d, *J* = 2.6 Hz, 1H), 8.71 (s, 1H), 8.44 (ddd, *J* = 8.4, 2.7, 1.4 Hz, 1H), 8.34 (dd, *J* = 4.7, 1.4 Hz, 1H), 7.80–7.73 (m, 3H), 7.42 (d, *J* = 7.8 Hz, 1H), 7.33 (dd, *J* = 8.4, 4.7 Hz, 1H), 7.23 (ddd, *J* = 14.2, 5.5, 3.8 Hz, 3H), 7.15 (td, *J* = 7.9, 1.2 Hz, 1H), 2.33 (s, 3H). $^{13}$C-NMR (126 MHz, $CDCl_3$): $\delta$ (ppm) 147.29, 146.63, 144.39, 141.97, 140.89, 135.52, 134.09, 130.49, 130.07, 126.97, 126.10, 125.37, 123.93,

122.83, 118.12, 112.87, 21.79. ESI-HRMS: m/z: found 365.10595 $[M+H]^+$ (calcd for $[C_{19}H_{17}N_4O_2S]^+$ 365.10722).

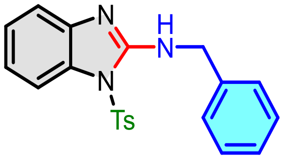


***N*-benzyl-1-tosyl-1*H*-benzo[*d*]imidazol-2-amine**[11] (**6r'**): was synthesized according to general procedure D by using benzyl isothiocyanate (**4r'**) [CAS NO. 622-78-6] (1.0 eq, 0.40 mmol), *o*-phenylenediamine (**1a**) [CAS NO. 95-54-5] (1.2 eq, 0.48 mmol, 52 mg), tosyl chloride (**2a**) [CAS NO. 98-59-9] (1.2 eq, 0.48 mmol, 92 mg) and potassium carbonate [CAS NO. 584-08-7] (1.2 eq & 1.0 eq) in co-solvent between 90% ethanol and 10% water (3.00 mL) to afford **6r'** (109.0 mg, 0.288 mmol, 72% yield) as a white solid. $^1$H-NMR (500 MHz, $CDCl_3$): $\delta$ (ppm) 7.74 (dd, *J* = 11.6, 4.4 Hz, 3H), 7.41–7.31 (m, 6H), 7.23–7.17 (m, 3H), 7.10–7.06 (m, 1H), 6.72 (t, *J* = 5.5 Hz, 1H), 4.75 (d, *J* = 5.7 Hz, 2H), 2.36 (s, 3H). $^{13}$C-NMR (126 MHz, $CDCl_3$): $\delta$ (ppm) 152.09, 146.10, 142.64, 137.94, 134.31, 130.97, 130.19, 128.87, 127.83, 127.80, 126.97, 125.00, 121.47, 116.97, 112.59, 47.26, 21.73. ESI-HRMS: m/z: found 378.12755 $[M+H]^+$ (calcd for $[C_{21}H_{20}N_3O_2S]^+$ 378.127623).

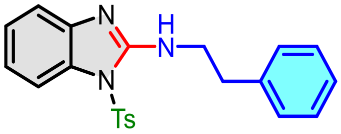


***N*-phenethyl-1-tosyl-1*H*-benzo[*d*]imidazol-2-amine**[11] (**6s'**): was synthesized according to general procedure D by using phenethyl isothiocyanate (**4s'**) [CAS NO. 2257-09-2] (1.0 eq, 0.40 mmol), *o*-phenylenediamine (**1a**) [CAS NO. 95-54-5] (1.2 eq, 0.48 mmol, 52 mg), tosyl chloride (**2a**) [CAS NO. 98-59-9] (1.2 eq, 0.48 mmol, 92 mg) and potassium carbonate [CAS NO. 584-08-7] (1.2 eq & 1.0 eq) in co-solvent between 90% ethanol and 10% water (3.00 mL) to afford **6s'** (58.9 mg, 0.152 mmol, 38% yield) as a white solid. $^1$H-NMR (500 MHz, $CDCl_3$): $\delta$ (ppm) 7.70–7.66 (m, 1H), 7.53–7.50 (m, 2H), 7.40–7.36 (m, 2H), 7.33–7.29 (m, 4H), 7.18–7.13 (m, 3H), 7.06–7.02 (m, 1H), 6.40 (t, *J* = 5.1 Hz, 1H), 3.82 (td, *J* = 6.7, 5.4 Hz, 2H), 3.05 (t, *J* = 6.7 Hz, 2H), 2.34 (s, 3H). $^{13}$C-NMR (126 MHz, $CDCl_3$): $\delta$ (ppm) 152.00, 145.96, 142.78, 138.79, 134.35, 130.84, 130.17, 129.0, 128.91, 126.94, 126.83, 124.94, 121.34, 116.86, 112.52, 44.34, 35.28, 21.77. ESI-HRMS: m/z: found 392.14251 $[M+H]^+$ (calcd for $[C_{22}H_{22}N_3O_2S]^+$ 392.14327).

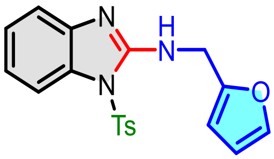


***N*-(furan-2-ylmethyl)-1-tosyl-1*H*-benzo[*d*]imidazol-2-amine** (**6t'**): was synthesized according to general procedure D by using 2-furfuryl isothiocyanate (**4t'**) (1.0 eq, 0.40 mmol), *o*-phenylenediamine (**1a**) [CAS NO. 95-54-5] (1.2 eq, 0.48 mmol, 52 mg), tosyl chloride (**2a**) [CAS NO. 98-59-9] (1.2 eq, 0.48 mmol, 92 mg) and potassium carbonate [CAS NO. 584-08-7] (1.2 eq & 1.0 eq) in co-solvent between 90% ethanol and 10% water (3.00 mL) to afford **6t'** (61.1 mg, 0.168 mmol, 42% yield) as a yellow solid. $^1$H-NMR (500 MHz, $CDCl_3$): $\delta$ (ppm) 7.72 (dd, *J* = 12.1, 8.3 Hz, 3H), 7.42 (dd, *J* = 1.7, 0.7 Hz, 1H), 7.36–7.32 (m, 1H), 7.23 – 7.20 (m, 2H), 7.17 (td, *J* = 7.7, 1.1 Hz, 1H), 7.06 (td, *J* = 8.0, 1.1 Hz, 1H), 6.76 (t, *J* = 5.4 Hz, 1H), 6.35 (dt, *J* = 8.7, 2.5 Hz, 2H), 4.74 (d, *J* = 5.6 Hz, 2H), 2.35 (s, 3H). $^{13}$C-NMR (126 MHz, $CDCl_3$): $\delta$ (ppm) 151.71, 151.04, 146.16, 142.54, 142.36, 134.24, 130.85, 130.22, 127.02, 125.06, 121.63, 117.01, 112.66, 110.64, 108.06, 40.25, 21.76. Melting point: 125-126$^o$C. ESI-HRMS: m/z: found 368.10678 $[M+H]^+$ (calcd for $[C_{19}H_{18}N_3O_3S]^+$ 368.10689).

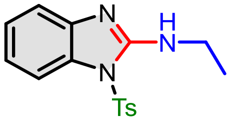


***N*-ethyl-1-tosyl-1*H*-benzo[*d*]imidazol-2-amine**[11] (**6u'**): was synthesized according to general procedure D by using ethyl isothiocyanate (**4u'**) [CAS NO. 542-85-8] (1.0 eq, 0.40 mmol), *o*-phenylenediamine (**1a**) [CAS NO. 95-54-5] (1.2 eq, 0.48 mmol, 52 mg), tosyl chloride (**2a**) [CAS NO. 98-59-9] (1.2 eq, 0.48 mmol, 92 mg) and potassium carbonate [CAS NO. 584-08-7] (1.2 eq & 1.0 eq) in co-solvent between 90% ethanol and 10% water (3.00 mL) to afford **6u'** (54.2 mg, 0.172 mmol, 43% yield) as a pale yellow solid. $^1$H-NMR (500 MHz, $CDCl_3$), $\delta$ 7.76 (d, *J* = 8.4 Hz, 2H), 7.71–7.66 (m, 1H), 7.32–7.28 (m, 1H), 7.22 (dd, *J* = 8.6, 0.6 Hz, 2H), 7.14 (td, *J* = 7.7, 1.2 Hz, 1H), 7.05–7.00 (m, 1H), 6.39 (t, *J* = 5.4 Hz, 1H), 3.57 (qd, *J* = 7.2, 5.6 Hz, 2H), 2.32 (s, 3H), 1.33 (t, *J* = 7.2 Hz, 3H). $^{13}$C-NMR (126 MHz, $CDCl_3$): $\delta$ (ppm) 152.11, 146.04, 142.80, 134.36, 130.74, 130.16, 126.87, 124.89, 121.18, 116.70, 112.49, 38.17, 21.68, 14.98. ESI-HRMS: m/z: found 316.11203 $[M+H]^+$ (calcd for $[C_{16}H_{18}N_3O_2S]^+$ 316.11197).

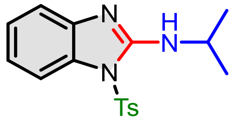

**N-isopropyl-1-tosyl-1H-benzo[d]imidazol-2-amine**[11] (**6v'**): was synthesized according to general procedure D by using isopropyl isothiocyanate (**4v'**) [CAS NO. 2253-73-8] (1.0 eq, 0.40 mmol), *o*-phenylenediamine (**1a**) [CAS NO. 95-54-5] (1.2 eq, 0.48 mmol, 52 mg), tosyl chloride (**2a**) [CAS NO. 98-59-9] (1.2 eq, 0.48 mmol, 92 mg) and potassium carbonate [CAS NO. 584-08-7] (1.2 eq & 1.0 eq) in co-solvent between 90% ethanol and 10% water (3.00 mL) to afford **6v'** (30.0 mg, 0.092 mmol, 23% yield) as a white solid. $^{1}$H-NMR (500 MHz, $CDCl_3$): $\delta$ (ppm) 7.75 (d, $J$ = 8.4 Hz, 2H), 7.72–7.68 (m, 1H), 7.30 (dd, $J$ = 7.7, 0.4 Hz, 1H), 7.23 (d, $J$ = 8.2 Hz, 2H), 7.14 (td, $J$ = 7.7, 1.1 Hz, 1H), 7.03 (td, $J$ = 7.8, 1.1 Hz, 1H), 6.28 (d, $J$ = 7.8 Hz, 1H), 4.24–4.14 (m, 1H), 2.34 (s, 3H), 1.34 (d, $J$ = 6.5 Hz, 6H). $^{13}$C-NMR (126 MHz, $CDCl_3$): $\delta$ (ppm) 151.46, 146.05, 142.99, 134.38, 130.66, 130.16, 126.88, 124.94, 121.14, 116.65, 112.60, 45.21, 23.03, 21.72. ESI-HRMS: m/z: found 330.12453 $[M+H]^+$ (calcd for $[C_{17}H_{20}N_3O_2S]^+$ 330.12762).

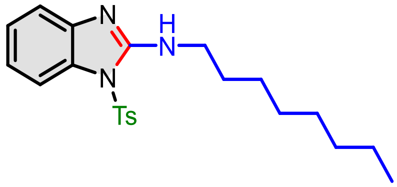

**N-octyl-1-tosyl-1H-benzo[d]imidazol-2-amine** (**6w'**): was synthesized according to general procedure D by using *n*-octyl isothiocyanate (**4w'**) [CAS NO. 4430-45-9] (1.0 eq, 0.40 mmol), *o*-phenylenediamine (**1a**) [CAS NO. 95-54-5] (1.2 eq, 0.48 mmol, 52 mg), tosyl chloride (**2a**) [CAS NO. 98-59-9] (1.2 eq, 0.48 mmol, 92 mg) and potassium carbonate [CAS NO. 584-08-7] (1.2 eq & 1.0 eq) in co-solvent between 90% ethanol and 10% water (3.00 mL) to afford **6w'** (60.5 mg, 0.151 mmol, 38% yield) as a pale yellow oil. $^{1}$H-NMR (500 MHz): $\delta$ (ppm) 7.76 (d, $J$ = 8.4 Hz, 2H), 7.69 (d, $J$ = 8.0 Hz, 1H), 7.33 (d, $J$ = 7.8 Hz, 1H), 7.24 (d, $J$ = 8.1 Hz, 2H), 7.16 (td, $J$ = 7.7, 1.0 Hz, 1H), 7.04 (td, $J$ = 8.1, 1.0 Hz, 1H), 6.44 (t, $J$ = 5.1 Hz, 1H), 3.54 (q, $J$ = 12.7, 7.0 Hz, 2H), 2.36 (s, 3H), 1.74–1.68 (m, 2H), 1.43–1.27 (m, 10H), 0.89 (t, $J$ = 7.0 Hz, 3H). $^{13}$C-NMR (126 MHz): $\delta$ (ppm) 152.13, 146.21, 142.26, 134.41, 130.65, 130.26, 126.96, 125.05, 121.40, 116.68, 112.55, 43.54, 31.92, 29.50, 29.38, 27.03, 22.78, 21.77, 14.23. ESI-HRMS: m/z: found 400.20521 $[M+H]^+$ (calcd for $[C_{23}H_{30}N_3O_2S]^+$ 400.20587).

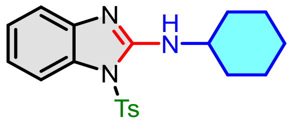

***N*-cyclohexyl-1-tosyl-1*H*-benzo[*d*]imidazol-2-amine**[11] (**6x'**): was synthesized according to general procedure D by using cyclohexyl isothiocyanate (**4x'**) [CAS NO. 1122-82-3] (1.0 eq, 0.40 mmol), *o*-phenylenediamine (**1a**) [CAS NO. 95-54-5] (1.2 eq, 0.48 mmol, 52 mg), tosyl chloride (**2a**) [CAS NO. 98-59-9] (1.2 eq, 0.48 mmol, 92 mg) and potassium carbonate [CAS NO. 584-08-7] (1.2 eq & 1.0 eq) in co-solvent between 90% ethanol and 10% water (3.00 mL) to afford **6x'** (34.3 mg, 0.092 mmol, 23% yield) as a white solid. $^{1}$H-NMR (500 MHz, $CDCl_3$): $\delta$ (ppm) 7.75 (d, $J$ = 8.4 Hz, 2H), 7.70 (d, $J$ = 7.9 Hz, 1H), 7.29 (d, $J$ = 7.8 Hz, 1H), 7.23 (d, $J$ = 8.4 Hz, 2H), 7.14 (td, $J$ = 7.7, 1.1 Hz, 1H), 7.02 (td, $J$ = 7.8, 1.1 Hz, 1H), 6.38 (d, $J$ = 8.1 Hz, 1H), 3.95–3.85 (m, 1H), 2.34 (s, 3H), 2.14–2.07 (m, 2H), 1.80–1.73 (m, 2H), 1.68–1.61 (m, 1H), 1.50–1.24 (m, 5H). $^{13}$C-NMR (126 MHz, $CDCl_3$): $\delta$ (ppm) 151.50, 146.04, 143.04, 134.40, 130.69, 130.16, 126.90, 124.95, 121.06, 116.60, 112.60, 51.65, 33.27, 25.67, 24.75, 21.74. ESI-HRMS: m/z: found 370.15853 $[M+H]^+$ (calcd for $[C_{20}H_{24}N_3O_2S]^+$ 370.15892).

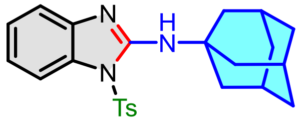

***N*-((3*s*,5*s*,7*s*)-adamantan-1-yl)-1-tosyl-1*H*-benzo[*d*]imidazol-2-amine** (**6y'**): was synthesized according to general procedure D by using 1-adamantyl isothiocyanate (**4y'**) [CAS NO. 4411-26-1] (1.0 eq, 0.40 mmol), *o*-phenylenediamine (**1a**) [CAS NO. 95-54-5] (1.2 eq, 0.48 mmol, 52 mg), tosyl chloride (**2a**) [CAS NO. 98-59-9] (1.2 eq, 0.48 mmol, 92 mg) and potassium carbonate [CAS NO. 584-08-7] (1.2 eq & 1.0 eq) in co-solvent between 90% ethanol and 10% water (3.00 mL) to afford **6y'** (6.3 mg, 0.001 mmol, 4% yield) as a white solid. $^{1}$H-NMR (500 MHz): $\delta$ (ppm) 7.74 (d, $J$ = 8.4 Hz), 7.70 (d, $J$ = 8.0 Hz), 7.33–7.27 (m), 7.25 (d, $J$ = 8.4 Hz), 7.14 (t, $J$ = 7.4 Hz), 7.02 (t, $J$ = 7.7 Hz), 6.48 (s), 2.36 (s), 2.15 (s), 1.78–1.70 (m). $^{13}$C-NMR (126 MHz): $\delta$ (ppm) 149.72, 146.00, 134.53, 130.15, 129.98, 126.92, 124.91, 121.15, 116.79, 112.74, 53.15, 41.82, 36.40, 29.68, 21.78. Melting point: 166-169°C. ESI-HRMS: m/z: found 422.19117 $[M+H]^+$ (calcd for $[C_{25}H_{27}N_3O_2S]^+$ 422.19022).

### 4.3. General procedure for visible-light-mediated photocatalyst-free reaction for synthesis of *N*-substituted-2-aminobenzimidazoles (6a''-6c'')

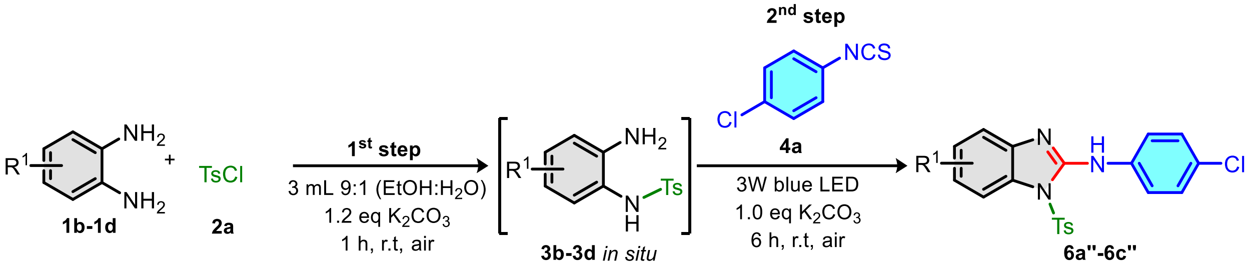


General procedure E: (STEP 1): A mixture of corresponding *o*-phenylenediamine (**1b-1d**) (1.2 eq, 0.48 mmol), tosyl chloride (**2a**) [CAS NO. 98-59-9] (1.2 eq, 0.48 mmol, 92 mg) and potassium carbonate [CAS NO. 584-08-7] (1.2 eq, 0.48 mmol, 66.3 mg) was dissolved by co-solvent between 90% ethanol and 10% water (3.0 mL) in pyrex schlenk reactor at room temperature for 1 hour under air. (STEP 2): After that, 4-chlorophenyl isothiocyanate (**4a**) (1.0 eq, 0.40 mmol, 68 mg) and potassium carbonate (1.0 eq, 0.40 mmol, 55.3 mg) were added into pyrex schlenk reactor as single-flask operation. The reaction was irradiated by 3W blue LED equipped with glass rod fiber at room temperature for 6 hours under air. The reaction mixture was washed water (2x10 mL) and the organic portion was extracted with EtOAc (3x10 mL). Water from organic layer was eliminated by $Na_2SO_4$. After filtration and removal of the solvent under reduced pressure, the crude product was purified by silica gel column chromatography (3:1 of hexane:EtOAc as mobile phase) to afford *N*-substituted-2-aminobenzimidazoles **6a''-6c''** as corresponding products.

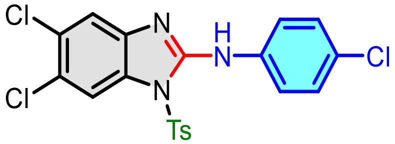


**5,6-dichloro-*N*-(4-chlorophenyl)-1-tosyl-1*H*-benzo[*d*]imidazol-2-amine** (**6a''**): was synthesized according to general procedure E by using 4,5-dichloro-1,2-phenylenediamine (**1b**) [CAS NO. 5348-42-5] (1.2 eq, 0.48 mmol), tosyl chloride (**2a**) [CAS NO. 98-59-9] (1.2 eq, 0.48 mmol, 92 mg), potassium carbonate [CAS NO. 584-08-7] (1.2 eq & 1.0 eq) and 4-chlorophenyl isothiocyanate (**4a**) (1.0 eq, 0.40 mmol, 68 mg) in co-solvent between 90% ethanol and 10% water (3.00 mL) to afford **6a''** (77.7 mg, 0.168 mmol, 42% yield) as a pale brown solid. $^1$H-NMR (500 MHz, $CDCl_3$): $\delta$ (ppm) 8.63 (s, 1H), 7.83 (s, 1H), 7.76 (d, *J* = 8.4 Hz, 2H), 7.69–7.65 (m, 2H),

7.46 (s, 1H), 7.37–7.34 (m, 2H), 7.29 (d, *J* = 8.3 Hz, 2H), 2.38 (s, 3H). $^{13}$C-NMR (126 MHz, $CDCl_3$): $\delta$ (ppm) 148.74, 147.16, 141.87, 136.51, 133.59, 130.73, 129.41, 129.24, 129.00, 126.97, 125.99, 120.67, 119.10, 114.19, 21.87. Melting point: 190-193°C. ESI-HRMS: m/z: found 465.99157 $[M+H]^+$ (calcd for $[C_{20}H_{15}Cl_3N_3O_2S]^+$ 465.99506).

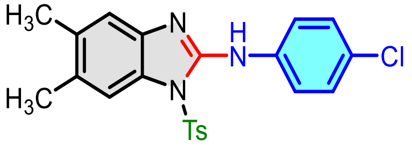


**_N_-(4-chlorophenyl)-5,6-dimethyl-1-tosyl-1_H_-benzo[_d_]imidazol-2-amine** (**6b''**): was synthesized according to general procedure E by using 4,5-dimethyl-1,2-phenylenediamine (**1c**) [CAS NO. 3171-45-7] (1.2 eq, 0.48 mmol), tosyl chloride (**2a**) [CAS NO. 98-59-9] (1.2 eq, 0.48 mmol, 92 mg), potassium carbonate [CAS NO. 584-08-7] (1.2 eq & 1.0 eq) and 4-chlorophenyl isothiocyanate (**4a**) (1.0 eq, 0.40 mmol, 68 mg) in co-solvent between 90% ethanol and 10% water (3.00 mL) to afford **6b''** (91.6 mg, 0.16 mmol, 54% yield) as a pale brown solid. $^1$H-NMR (500 MHz, $CDCl_3$): $\delta$ (ppm) 8.61 (s, 1H), 7.76 (d, *J* = 8.4 Hz, 2H), 7.70 (d, *J* = 8.8 Hz, 2H), 7.53 (s, 1H), 7.34 (d, *J* = 8.8 Hz, 2H), 7.25–7.21 (m, 3H), 2.35 (s, 3H), 2.33 (s, 3H), 2.27 (s, 3H). $^{13}$C-NMR (126 MHz, $CDCl_3$): $\delta$ (ppm) 147.02, 146.33, 140.16, 137.23, 134.28, 133.97, 131.40, 130.42, 129.30, 128.14, 126.88, 120.30, 118.58, 113.54, 21.79, 20.50, 20.22. Melting point: 203-206°C. ESI-HRMS: m/z: found 426.10251 $[M+H]^+$ (calcd for $[C_{22}H_{21}ClN_3O_2S]^+$ 426.10430).

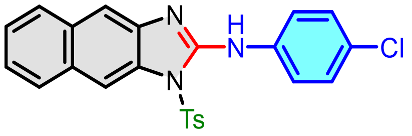


**_N_-(4-chlorophenyl)-1-tosyl-1_H_-naphtho[2,3-_d_]imidazol-2-amine** (**6c''**): was synthesized according to general procedure E by using 2,3-diaminonaphthalene (**1d**) [CAS NO. 771-97-1] (1.2 eq, 0.48 mmol), tosyl chloride (**2a**) [CAS NO. 98-59-9] (1.2 eq, 0.48 mmol, 92 mg), potassium carbonate [CAS NO. 584-08-7] (1.2 eq & 1.0 eq) and 4-chlorophenyl isothiocyanate (**4a**) (1.0 eq, 0.40 mmol, 68 mg) in co-solvent between 90% ethanol and 10% water (3.00 mL) to afford **6c''** (75.0 mg, 0.168 mmol, 42% yield) as a pale yellow solid. $^1$H-NMR (500 MHz, $CDCl_3$): $\delta$ (ppm) 8.83 (s, 1H), 8.17 (s, 1H), 7.94–7.91 (m, 1H), 7.86–7.83 (m, 1H), 7.82–7.76 (m, 5H), 7.45–7.41 (m, 2H), 7.41–7.36 (m, 2H), 7.21 (d, *J* = 8.2 Hz, 2H), 2.31 (s, 3H). $^{13}$C-NMR (126 MHz, $CDCl_{3,}$): $\delta$ (ppm) 149.23, 146.59, 141.82, 136.74, 133.86, 132.20, 130.48, 130.18, 129.41, 128.93, 128.17, 127.81,

126.96, 125.26, 124.62, 120.84, 114.29, 110.21, 21.77. Melting point: 223-225°C. ESI-HRMS: m/z: found 448.08584 $[M+H]^+$ (calcd for $[C_{24}H_{19}ClN_3O_2S]^+$ 448.08865).

### 4.4. General procedure for visible-light-mediated photocatalyst-free reaction for synthesis of *N*-substituted-2-aminobenzimidazoles (6d''-6s'')

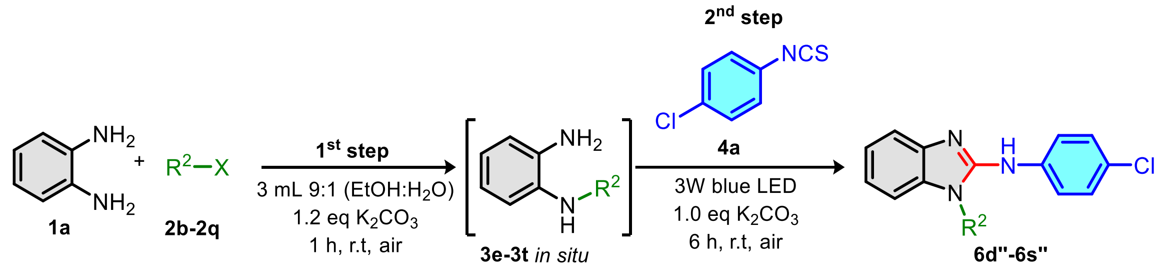


General procedure F: (STEP 1): A mixture of *o*-phenylenediamine (**1a**) [CAS NO. 95-54-5] (1.2 eq, 0.48 mmol, 52 mg), the corresponding electrophile (**2b-2q**) (1.2 eq, 0.48 mmol) and potassium carbonate [CAS NO. 584-08-7] (1.2 eq, 0.48 mmol, 66.3 mg) was dissolved by co-solvent between 90% ethanol and 10% water (3.0 mL) in pyrex schlenk reactor at room temperature for 1 hour under air. (STEP 2): After that, 4-chlorophenyl isothiocyanate (**4a**) (1.0 eq, 0.40 mmol, 68 mg) and potassium carbonate (1.0 eq, 0.40 mmol, 55.3 mg) were added into pyrex schlenk reactor as single-flask operation. The reaction was irradiated by 3W blue LED equipped with glass rod fiber at room temperature for 6 hours under air. The reaction mixture was washed water (2x10 mL) and the organic portion was extracted with EtOAc (3x10 mL). Water from organic layer was eliminated by $Na_2SO_4$. After filtration and removal of the solvent under reduced pressure, the crude product was purified by silica gel column chromatography (3:1 of hexane:EtOAc as mobile phase) to afford *N*-substituted-2-aminobenzimidazoles **6d''-6s''** as corresponding products.

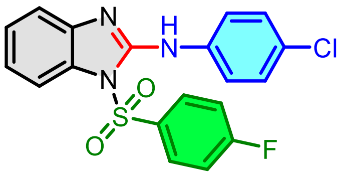

**_N_-(4-chlorophenyl)-1-((4-fluorophenyl)sulfonyl)-1_H_-benzo[_d_]imidazol-2-amine** (**6d''**): was synthesized according to general procedure F by using 4-fluorobenzenesulfonyl chloride (**2b**) [CAS NO. 349-88-2] (1.2 eq, 0.48 mmol), _o_-phenylenediamine (**1a**) [CAS NO. 95-54-5] (1.2 eq, 0.48 mmol, 52 mg), potassium carbonate [CAS NO. 584-08-7] (1.2 eq & 1.0 eq) and 4-chlorophenyl isothiocyanate (**4a**) (1.0 eq, 0.40 mmol, 68 mg) in co-solvent between 90% ethanol and 10% water (3.00 mL) to afford **6d''** (122.3 mg, 0.304 mmol, 76% yield) as a white solid. $^{1}$H-NMR (500 MHz, $CDCl_3$): $\delta$ (ppm) 8.61 (s, 1H), 7.92 (ddd, _J_ = 10.0, 5.0, 2.6 Hz, 2H), 7.76–7.71 (m, 3H), 7.46–7.43 (m, 1H), 7.38–7.34 (m, 2H), 7.25 (td, _J_ = 7.7, 1.2 Hz, 1H), 7.18–7.11 (m, 3H). $^{13}$C-NMR (126 MHz, $CDCl_3$): $\delta$ (ppm) 167.43, 165.37, 147.36, 142.22, 136.92, 133.05, 133.03, 129.92, 129.84, 129.78, 129.33, 128.49, 125.59, 122.77, 120.44, 118.15, 117.40, 117.22, 112.77. $^{19}$F-NMR (471 MHz, $CDCl_3$): $\delta$ (ppm) -104.27. Melting point: 140-142°C. ESI-HRMS: m/z: found 402.04541 $[M+H]^+$ (calcd for $[C_{19}H_{14}ClFN_3O_2S]^+$ 402.04793).

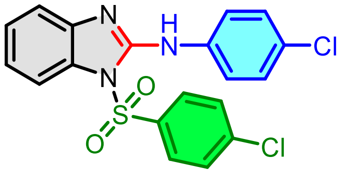

**_N_-(4-chlorophenyl)-1-((4-chlorophenyl)sulfonyl)-1_H_-benzo[_d_]imidazol-2-amine** (**6e''**): was synthesized according to general procedure F by using 4-chlorobenzenesulfonyl chloride (**2c**) [CAS NO. 98-60-2] (1.2 eq, 0.48 mmol), _o_-phenylenediamine (**1a**) [CAS NO. 95-54-5] (1.2 eq, 0.48 mmol, 52 mg), potassium carbonate [CAS NO. 584-08-7] (1.2 eq & 1.0 eq) and 4-chlorophenyl isothiocyanate (**4a**) (1.0 eq, 0.40 mmol, 68 mg) in co-solvent between 90% ethanol and 10% water (3.00 mL) to afford **6e''** (122.7 mg, 0.292 mmol, 73% yield) as a white solid. $^{1}$H-NMR (500 MHz, $CDCl_3$): $\delta$ (ppm) 8.59 (s, 1H), 7.83–7.80 (m, 2H), 7.75–7.70 (m, 3H), 7.46–7.43 (m, 1H), 7.42–7.39 (m, 2H), 7.37–7.34 (m, 2H), 7.25 (td, _J_ = 7.7, 1.1 Hz, 1H), 7.16 (td, _J_ = 8.1, 1.1 Hz, 1H). $^{13}$C-NMR (126 MHz, $CDCl_3$): $\delta$ (ppm) 147.30, 142.20, 141.93, 136.88, 135.35, 130.16, 129.72, 129.31, 128.51, 128.25, 125.63, 122.79, 120.45, 118.17, 112.76. Melting point: 146-148°C. ESI-HRMS: m/z: found 418.01676 $[M+H]^+$ (calcd for $[C_{19}H_{14}Cl_2N_3O_2S]^+$ 418.01838).

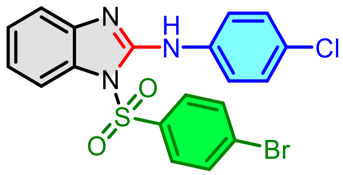

**1-((4-bromophenyl)sulfonyl)-*N*-(4-chlorophenyl)-1*H*-benzo[*d*]imidazol-2-amine** (**6f''**): was synthesized according to general procedure by F using 4-bromobenzenesulfonyl chloride (**2d**) [CAS NO. 98-58-8] (1.2 eq, 0.48 mmol), *o*-phenylenediamine (**1a**) [CAS NO. 95-54-5] (1.2 eq, 0.48 mmol, 52 mg), potassium carbonate [CAS NO. 584-08-7] (1.2 eq & 1.0 eq) and 4-chlorophenyl isothiocyanate (**4a**) (1.0 eq, 0.40 mmol, 68 mg) in co-solvent between 90% ethanol and 10% water (3.00 mL) to afford **6f''** (161.2 mg, 0.344 mmol, 86% yield) as a yellow solid. $^{1}$H-NMR (500 MHz, $CDCl_3$): $\delta$ (ppm) 8.58 (s, 1H), 7.73 (dt, *J* = 10.8, 5.5 Hz, 5H), 7.60–7.56 (m, 2H), 7.44 (d, *J* = 7.9 Hz, 1H), 7.38–7.34 (m, 2H), 7.25 (td, *J* = 7.8, 1.0 Hz, 1H), 7.18–7.14 (m, 1H). $^{13}$C-NMR (126 MHz, $CDCl_3$): $\delta$ (ppm) 147.33, 142.23, 136.89, 135.92, 133.19, 130.64, 129.74, 129.35, 128.56, 128.25, 125.68, 122.83, 120.48, 118.21, 112.79. Melting point: 166-168°C. ESI-HRMS: m/z: found 461.96468 $[M+H]^+$ (calcd for $[C_{19}H_{14}BrClN_3O_2S]^+$ 461.96786).

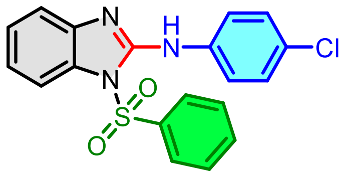

***N*-(4-chlorophenyl)-1-(phenylsulfonyl)-1*H*-benzo[*d*]imidazol-2-amine** (**6g''**): was synthesized according to general procedure F by using benzenesulfonyl chloride (**2e**) [CAS NO. 98-09-9] (1.0 eq, 0.40 mmol), *o*-phenylenediamine (**1a**) [CAS NO. 95-54-5] (1.2 eq, 0.48 mmol, 52 mg), potassium carbonate [CAS NO. 584-08-7] (1.2 eq & 1.0 eq) and 4-chlorophenyl isothiocyanate (**4a**) (1.0 eq, 0.40 mmol, 68 mg) in co-solvent between 90% ethanol and 10% water (3.00 mL) to afford **6g''** (115.4 mg, 0.30 mmol, 75% yield) as a colorless oil. $^{1}$H-NMR (500 MHz, $CDCl_3$): $\delta$ (ppm) 8.66 (s, 1H), 7.90 (dt, *J* = 8.7, 1.6 Hz, 2H), 7.77 (d, *J* = 7.8 Hz, 1H), 7.74–7.70 (m, 2H), 7.60–7.56 (m, 1H), 7.47–7.42 (m, 3H), 7.37–7.33 (m, 2H), 7.25–7.21 (m, 1H), 7.15 (td, *J* = 7.8, 1.2 Hz, 1H). $^{13}$C-NMR (126 MHz, $CDCl_3$): $\delta$ (ppm) 147.47, 142.16, 137.10, 137.03, 135.05, 129.93, 129.82, 129.30, 128.38, 126.88, 125.40, 122.67, 120.44, 118.02, 112.82. Melting point: 119-122°C. ESI-HRMS: m/z: found 384.05537 $[M+H]^+$ (calcd for $[C_{19}H_{15}ClN_3O_2S]^+$ 384.05735).

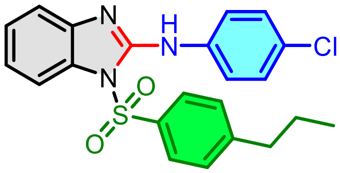


**N-(4-chlorophenyl)-1-((4-propylphenyl)sulfonyl)-1*H*-benzo[*d*]imidazol-2-amine (6h'')**: was synthesized according to general procedure F by using 4-propylbenzenesulfonyl chloride (**2f**) [CAS NO. 146949-07-7] (1.2 eq, 0.48 mmol), *o*-phenylenediamine (**1a**) [CAS NO. 95-54-5] (1.2 eq, 0.48 mmol, 52 mg), potassium carbonate [CAS NO. 584-08-7] (1.2 eq & 1.0 eq) and 4-chlorophenyl isothiocyanate (**4a**) (1.0 eq, 0.40 mmol, 68 mg) in co-solvent between 90% ethanol and 10% water (3.00 mL) to afford **6h''** (121.9 mg, 0.288 mmol, 72% yield) as a white solid. $^1$H-NMR (500 MHz, $CDCl_3$): $\delta$ (ppm) 8.70 (s, 1H), 7.79 (dd, *J* = 11.4, 8.4 Hz, 3H), 7.74 (t, *J* = 5.7 Hz, 2H), 7.44 (d, *J* = 7.8 Hz, 1H), 7.35 (t, *J* = 5.6 Hz, 2H), 7.26–7.21 (m, 3H), 7.15 (t, *J* = 7.8 Hz, 1H), 2.58–2.52 (m, 2H), 1.60–1.51 (m, 2H), 0.88 (t, *J* = 7.3 Hz, 3H). $^{13}$C-NMR (126 MHz, $CDCl_3$): $\delta$ (ppm) 151.04, 147.49, 142.13, 137.10, 134.29, 129.99, 129.80, 129.26, 128.26, 126.94, 125.24, 122.57, 120.40, 117.92, 112.82, 37.94, 23.96, 13.78. Melting point: 147-149°C. ESI-HRMS: m/z: found 426.10202 $[M+H]^+$ (calcd for $[C_{22}H_{21}ClN_3O_2S]^+$ 426.10430).

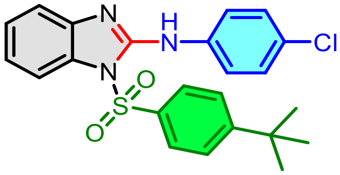


**1-((4-(*tert*-butyl)phenyl)sulfonyl)-*N*-(4-chlorophenyl)-1*H*-benzo[*d*]imidazol-2-amine (6i'')**: was synthesized according to general procedure F by using 4-*tert*-butylbenzenesulfonyl chloride (**2g**) [CAS NO. 15084-51-2] (1.2 eq, 0.48 mmol), *o*-phenylenediamine (**1a**) [CAS NO. 95-54-5] (1.2 eq, 0.48 mmol, 52 mg), potassium carbonate [CAS NO. 584-08-7] (1.2 eq & 1.0 eq) and 4-chlorophenyl isothiocyanate (**4a**) (1.0 eq, 0.40 mmol, 68 mg) in co-solvent between 90% ethanol and 10% water (3.00 mL) to afford **6i''** (150.9 mg, 0.344 mmol, 86% yield) as a white solid. $^1$H-NMR (500 MHz, $CDCl_3$): $\delta$ (ppm) 8.71 (s, 1H), 7.84–7.81 (m, 2H), 7.81–7.79 (m, 1H), 7.75–7.71 (m, 2H), 7.46 (dd, *J* = 11.4, 4.6 Hz, 3H), 7.37–7.34 (m, 2H), 7.26–7.23 (m, 1H), 7.17 (td, *J* = 8.0, 1.1 Hz, 1H), 1.26 (s, 9H). $^{13}$C-NMR (126 MHz, $CDCl_3$): $\delta$ (ppm) 159.35, 147.54, 142.05, 137.08, 134.12, 130.06, 129.36, 128.43, 126.96, 126.85, 125.31, 122.68, 120.51, 117.94, 112.90, 35.51, 30.96.

Melting point: 184-187°C. ESI-HRMS: m/z: found 440.11800 $[M+H]^+$ (calcd for $[C_{23}H_{23}ClN_3O_2S]^+$ 440.11995).

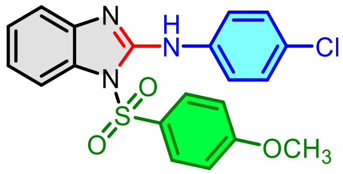


**N-(4-chlorophenyl)-1-((4-methoxyphenyl)sulfonyl)-1*H*-benzo[*d*]imidazol-2-amine (6j'')**: was synthesized according to general procedure F by using 4-methoxybenzenesulfonyl chloride (**2h**) [CAS NO. 98-68-0] (1.2 eq, 0.48 mmol), *o*-phenylenediamine (**1a**) [CAS NO. 95-54-5] (1.2 eq, 0.48 mmol, 52 mg), potassium carbonate [CAS NO. 584-08-7] (1.2 eq & 1.0 eq) and 4-chlorophenyl isothiocyanate (**4a**) (1.0 eq, 0.40 mmol, 68 mg) in co-solvent between 90% ethanol and 10% water (3.00 mL) to afford **6j''** (121.3 mg, 0.292 mmol, 73% yield) as a white solid. $^1$H-NMR (500 MHz, $CDCl_3$): $\delta$ (ppm) 8.70 (s, 1H), 7.85–7.81 (m, 2H), 7.76 (d, $J$ = 7.9 Hz, 1H), 7.75–7.71 (m, 2H), 7.44 (d, $J$ = 7.7 Hz, 1H), 7.37–7.33 (m, 2H), 7.23 (td, $J$ = 7.7, 1.1 Hz, 1H), 7.15 (td, $J$ = 8.0, 1.1 Hz, 1H), 6.89–6.85 (m, 2H), 3.76 (s, 3H). $^{13}$C-NMR (126 MHz, $CDCl_3$): $\delta$ (ppm) 164.72, 147.55, 142.16, 137.13, 129.97, 129.27, 128.31, 128.26, 125.22, 122.54, 120.40, 117.92, 114.97, 112.82, 55.82. Melting point: 152-154°C. ESI-HRMS: m/z: found 414.06571 $[M+H]^+$ (calcd for $[C_{20}H_{17}ClN_3O_3S]^+$ 414.06792).

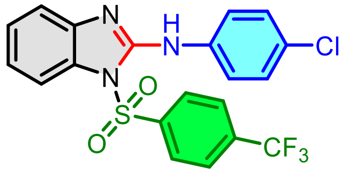


**N-(4-chlorophenyl)-1-((4-(trifluoromethyl)phenyl)sulfonyl)-1*H*-benzo[*d*]imidazol-2-amine (6k'')**: was synthesized according to general procedure F by using 4-(trifluoromethyl)benzenesulfonyl chloride (**2i**) [CAS NO. 2991-42-6] (1.2 eq, 0.48 mmol), *o*-phenylenediamine (**1a**) [CAS NO. 95-54-5] (1.2 eq, 0.48 mmol, 52 mg), potassium carbonate [CAS NO. 584-08-7] (1.2 eq & 1.0 eq) and 4-chlorophenyl isothiocyanate (**4a**) (1.0 eq, 0.40 mmol, 68 mg) in co-solvent between 90% ethanol and 10% water (3.00 mL) to afford **6k''** (49.9 mg, 0.112 mmol, 28% yield) as a pale yellow solid. $^1$H-NMR (500 MHz, $CDCl_3$): $\delta$ (ppm) 8.56 (s, 1H), 8.02 (d, $J$ = 8.3 Hz, 2H), 7.77–7.70 (m, 5H), 7.45 (d, $J$ = 7.7 Hz, 1H), 7.38–7.35 (m, 2H), 7.28–7.25 (m, 1H), 7.18 (td, $J$ = 8.1, 1.1 Hz, 1H). $^{13}$C-NMR (126 MHz, $CDCl_3$): $\delta$ (ppm) 147.27, 142.27, 140.48, 136.82,

136.52 (q, *J* = 33.02 Hz), 129.71, 129.42, 128.76, 127.52, 127.07 (q, *J* = 3.57 Hz), 125.91, 123.00, 122.76 (d, *J* = 273.78 Hz), 120.54, 118.37, 112.81. $^{19}$F-NMR (471 MHz): $\delta$(ppm) -63.36. Melting point: 163-165°C. ESI-HRMS: m/z: found 452.04249 $[M+H]^+$ (calcd for $[C_{20}H_{14}ClF_3N_3O_2S]^+$ 452.04474).

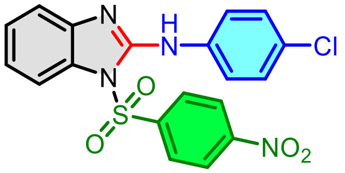


***N*-(4-chlorophenyl)-1-((4-nitrophenyl)sulfonyl)-1*H*-benzo[*d*]imidazol-2-amine** (**6l''**): was synthesized according to general procedure F by using 4-nitrobenzenesulfonyl chloride (**2j**) [CAS NO. 98-74-8] (1.2 eq, 0.48 mmol), *o*-phenylenediamine (**1a**) [CAS NO. 95-54-5] (1.2 eq, 0.48 mmol, 52 mg), potassium carbonate [CAS NO. 584-08-7] (1.2 eq & 1.0 eq) and 4-chlorophenyl isothiocyanate (**4a**) (1.0 eq, 0.40 mmol, 68 mg) in co-solvent between 90% ethanol and 10% water (3.00 mL) to afford **6l''** (21.8 mg, 0.052 mmol, 13% yield) as an orange solid. $^{1}$H-NMR (500 MHz, $CDCl_3$): $\delta$(ppm) 8.50 (s, 1H), 8.29 (d, *J* = 8.9 Hz, 2H), 8.07 (d, *J* = 8.9 Hz, 2H), 7.74 (d, *J* = 8.0 Hz, 1H), 7.70 (d, *J* = 8.9 Hz, 2H), 7.44 (d, *J* = 7.9 Hz, 1H), 7.36 (d, *J* = 8.9 Hz, 2H), 7.28–7.25 (m, 1H), 7.18 (td, *J* = 7.9, 1.1 Hz, 1H). $^{13}$C-NMR (126 MHz, $CDCl_3$): $\delta$(ppm) 151.34, 147.16, 142.31, 136.69, 129.57, 129.46, 128.93, 128.33, 126.17, 125.07, 123.16, 120.56, 118.53, 112.81. Melting point: 184-187°C. ESI-HRMS: m/z: found 429.04085 $[M+H]^+$ (calcd for $[C_{19}H_{14}ClN_4O_4S]^+$ 429.04243.

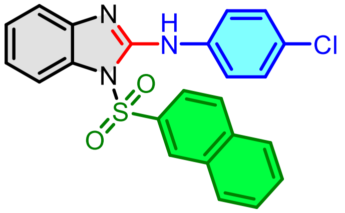


***N*-(4-chlorophenyl)-1-(naphthalen-2-ylsulfonyl)-1*H*-benzo[*d*]imidazol-2-amine** (**6m''**): was synthesized according to general procedure F using 2-naphthalenesulfonyl chloride (**2k**) [CAS NO. 93-11-8] (1.2 eq, 0.48 mmol), *o*-phenylenediamine (**1a**) [CAS NO. 95-54-5] (1.2 eq, 0.48 mmol, 52 mg), potassium carbonate [CAS NO. 584-08-7] (1.2 eq & 1.0 eq) and 4-chlorophenyl isothiocyanate (**4a**) (1.0 eq, 0.40 mmol, 68 mg) in co-solvent between 90% ethanol and 10% water (3.00 mL) to afford **6m''** (90.0 mg, 0.208 mmol, 52% yield) as a pale yellow solid. $^{1}$H-NMR (500 MHz, $CDCl_3$): $\delta$(ppm) 8.77 (s, 1H), 8.54 (s, 1H), 7.93 (d, *J* = 8.1 Hz, 1H), 7.86 (d, *J* = 8.8 Hz,

1H), 7.82 (d, $J$ = 8.0 Hz, 2H), 7.78–7.74 (m, 3H), 7.62 (ddd, $J$ = 21.1, 7.9, 6.9 Hz, 2H), 7.43 (d, $J$ = 7.7 Hz, 1H), 7.37 (dd, $J$ = 8.7, 0.8 Hz, 2H), 7.22 (t, $J$ = 7.7 Hz, 1H), 7.15 (t, $J$ = 7.8 Hz, 1H). $^{13}$C-NMR (126 MHz, $CDCl_3$): $\delta$ (ppm) 147.56, 142.09, 137.08, 135.71, 133.83, 131.81, 130.47, 130.13, 129.95, 129.70, 129.35, 129.25, 128.44, 128.30, 128.09, 125.38, 122.68, 120.92, 120.54, 118.03, 112.81. Melting point: 175-178°C. ESI-HRMS: m/z: found 434.07158 $[M+H]^+$ (calcd for $[C_{23}H_{17}ClN_3O_2S]^+$ 434.07300).

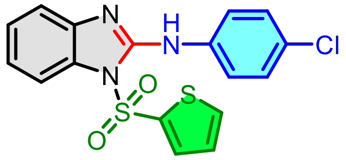


***N*-(4-chlorophenyl)-1-(thiophen-2-ylsulfonyl)-1*H*-benzo[*d*]imidazol-2-amine** (**6n''**): was synthesized according to general procedure F by using 2-thiophenesulfonyl chloride (**2l**) [CAS NO. 16629-19-9] (1.2 eq, 0.48 mmol), *o*-phenylenediamine (**1a**) [CAS NO. 95-54-5] (1.2 eq, 0.48 mmol, 52 mg), potassium carbonate [CAS NO. 584-08-7] (1.2 eq & 1.0 eq) and 4-chlorophenyl isothiocyanate (**4a**) (1.0 eq, 0.40 mmol, 68 mg) in co-solvent between 90% ethanol and 10% water (3.00 mL) to afford **6n''** (117.9 mg, 0.304 mmol, 76% yield) as a white solid. $^{1}$H-NMR (500 MHz, $CDCl_3$): $\delta$ (ppm) 8.55 (s, 1H), 7.81 (d, $J$ = 7.9 Hz, 1H), 7.73–7.69 (m, 3H), 7.56 (dd, $J$ = 5.0, 1.3 Hz, 1H), 7.45 (d, $J$ = 7.6 Hz, 1H), 7.36–7.32 (m, 2H), 7.26 (dt, $J$ = 7.7, 3.8 Hz, 1H), 7.18 (td, $J$ = 8.0, 1.1 Hz, 1H), 6.99 (dd, $J$ = 5.0, 4.0 Hz, 1H). $^{13}$C-NMR (126 MHz, $CDCl_3$): $\delta$ (ppm) 147.24, 142.33, 136.91, 136.28, 135.05, 134.07, 129.76, 129.27, 128.47, 128.13, 125.66, 122.71, 120.52, 118.04, 113.14. Melting point: 138-141°C. ESI-HRMS: m/z: found 390.01150 $[M+H]^+$ (calcd for $[C_{17}H_{13}ClN_3O_2S_2]^+$ 390.01377).

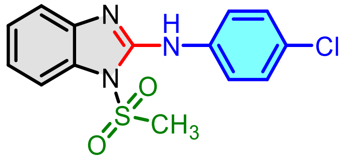


***N*-(4-chlorophenyl)-1-(methylsulfonyl)-1*H*-benzo[*d*]imidazol-2-amine** (**6o''**): was synthesized according to general procedure F by using methanesulfonyl chloride (**2m**) [CAS NO. 124-63-0] (1.2 eq, 0.48 mmol), *o*-phenylenediamine (**1a**) [CAS NO. 95-54-5] (1.2 eq, 0.48 mmol, 52 mg), potassium carbonate [CAS NO. 584-08-7] (1.2 eq & 1.0 eq) and 4-chlorophenyl isothiocyanate (**4a**) (1.0 eq, 0.40 mmol, 68 mg) in co-solvent between 90% ethanol and 10% water (3.00 mL) to

afford **6o''** (63.4 mg, 0.196 mmol, 49% yield) as a white solid. $^{1}$H-NMR (500 MHz, $CDCl_3$): $\delta$ (ppm) 8.41 (s, 1H), 7.73–7.69 (m, 2H), 7.66 (d, *J* = 8.0 Hz, 1H), 7.54 (d, *J* = 7.9 Hz, 1H), 7.36–7.29 (m, 3H), 7.20 (td, *J* = 8.1, 1.1 Hz, 1H), 3.22 (s, 3H). $^{13}$C-NMR (126 MHz, $CDCl_3$): $\delta$ (ppm) 147.14, 142.11, 136.87, 130.05, 129.27, 128.40, 125.58, 122.85, 120.40, 118.24, 112.18, 40.81. Melting point: 133-136$^{o}$C. ESI-HRMS: m/z: found 322.04076 $[M+H]^+$ (calcd for $[C_{14}H_{13}ClN_3O_2S]^+$ 322.04170).

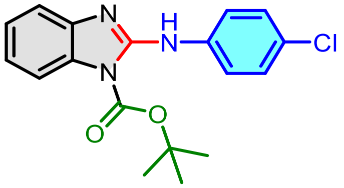


***tert*-butyl 2-((4-chlorophenyl)amino)-1*H*-benzo[*d*]imidazole-1-carboxylate** (**6p''**): was synthesized according to general procedure F by using di-*tert*-butyl dicarbonate (**2n**) [CAS NO. 24424-99-5] (1.2 eq, 0.48 mmol), *o*-phenylenediamine (**1a**) [CAS NO. 95-54-5] (1.2 eq, 0.48 mmol, 52 mg), potassium carbonate [CAS NO. 584-08-7] (1.2 eq & 1.0 eq) and 4-chlorophenyl isothiocyanate (**4a**) (1.0 eq, 0.40 mmol, 68 mg) in co-solvent between 90% ethanol and 10% water (3.00 mL) to afford **6p''** (35.9 mg, 0.101 mmol, 26% yield) as a white solid. $^{1}$H-NMR (500 MHz, $CDCl_3$): $\delta$ (ppm) 9.72 (s, 1H), 7.79–7.75 (m, 2H), 7.63 (s, 1H), 7.52–7.48 (m, 1H), 7.33–7.29 (m, 2H), 7.25 (dd, *J* = 10.9, 4.4 Hz, 1H), 7.12 (dd, *J* = 15.5, 0.9 Hz, 1H), 1.75 (s, 9H). $^{13}$C-NMR (126 MHz, $CDCl_3$): $\delta$ (ppm) 151.58, 149.65, 142.22, 137.48, 129.65, 129.11, 127.63, 124.75, 121.76, 120.27, 117.62, 114.19, 86.78, 28.26. Melting point: 168-170$^{o}$C. ESI-HRMS: m/z: found 344.11516 $[M+H]^+$ (calcd for $[C_{18}H_{19}ClN_3O_2]^+$ 344.11658).

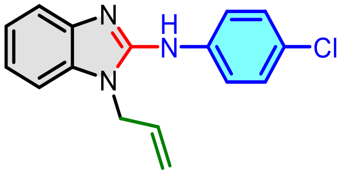


**1-allyl-*N*-(4-chlorophenyl)-1*H*-benzo[*d*]imidazol-2-amine** (**6q''**): was synthesized according to general procedure F by using allyl bromide (**2o**) [CAS NO. 106-95-6] (1.2 eq, 0.48 mmol), *o*-phenylenediamine (**1a**) [CAS NO. 95-54-5] (1.2 eq, 0.48 mmol, 52 mg), potassium carbonate [CAS NO. 584-08-7] (1.2 eq & 1.0 eq) and 4-chlorophenyl isothiocyanate (**4a**) (1.0 eq, 0.40 mmol, 68 mg) in co-solvent between 90% ethanol and 10% water (3.00 mL) to afford **6q''** (30.6 mg, 0.108 mmol, 27% yield) as a light brown solid. $^{1}$H-NMR (500 MHz, $CDCl_3$): $\delta$ (ppm) 7.58 (d, *J* = 7.7 Hz, 1H), 7.47 (d, *J* = 8.8 Hz, 2H), 7.27–7.25 (m, 2H), 7.20–7.13 (m, 3H), 6.38 (s, 1H), 5.98 (ddt, *J* = 17.1,

10.1, 4.9 Hz, 1H), 5.34 (d, *J* = 10.4 Hz, 1H), 5.22–5.17 (m, 1H), 4.63 (dt, *J* = 4.8, 1.7 Hz, 2H). $^{13}$C-NMR (126 MHz, $CDCl_3$): $\delta$ (ppm) 149.66, 141.49, 138.79, 133.69, 131.86, 129.27, 127.32, 122.01, 121.07, 119.71, 118.32, 117.64, 108.02, 45.03. Melting point: 191-194°C. ESI-HRMS: m/z: found 284.09596 $[M+H]^+$ (calcd for $[C_{16}H_{15}ClN_3]^+$ 284.09545).

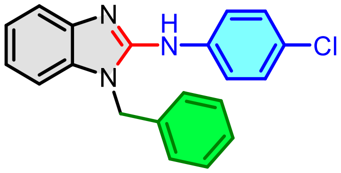


**1-benzyl-*N*-(4-chlorophenyl)-1*H*-benzo[*d*]imidazol-2-amine** (**6r''**): was synthesized according to general procedure F by using benzyl bromide (**2p**) [CAS NO. 100-39-0] (1.2 eq, 0.48 mmol), *o*-phenylenediamine (**1a**) [CAS NO. 95-54-5] (1.2 eq, 0.48 mmol, 52 mg), potassium carbonate [CAS NO. 584-08-7] (1.2 eq & 1.0 eq) and 4-chlorophenyl isothiocyanate (**4a**) (1.0 eq, 0.40 mmol, 68 mg) in co-solvent between 90% ethanol and 10% water (3.00 mL) to afford **6r''** (34.0 mg, 0.10 mmol, 25% yield) as a light brown solid. $^{1}$H-NMR (500 MHz, $CDCl_3$): $\delta$ (ppm) 7.55 (d, *J* = 7.8 Hz, 1H), 7.37–7.31 (m, 5H), 7.22–7.16 (m, 5H), 7.09 (d, *J* = 8.8 Hz, 2H), 5.30 (s, 2H). $^{13}$C-NMR (126 MHz, $CDCl_3$): $\delta$ (ppm) 149.71, 139.71, 138.18, 135.05, 133.62, 129.41, 129.16, 128.50, 127.86, 126.84, 122.47, 121.65, 120.56, 116.78, 108.61, 46.43. Melting point: 214-217°C. ESI-HRMS: m/z: found 334.11214 $[M+H]^+$ (calcd for $[C_{20}H_{17}ClN_3]^+$ 334.11110).

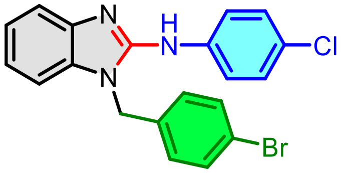


**1-(4-bromobenzyl)-*N*-(4-chlorophenyl)-1*H*-benzo[*d*]imidazol-2-amine** (**6s''**): was synthesized according to general procedure F by using 4-bromobenzyl bromide (**2q**) [CAS NO. 589-15-1] (1.2 eq, 0.48 mmol), *o*-phenylenediamine (**1a**) [CAS NO. 95-54-5] (1.2 eq, 0.48 mmol, 52 mg), potassium carbonate [CAS NO. 584-08-7] (1.2 eq & 1.0 eq) and 4-chlorophenyl isothiocyanate (**4a**) (1.0 eq, 0.40 mmol, 68 mg) in co-solvent between 90% ethanol and 10% water (3.00 mL) to afford **6s''** (69.5 mg, 0.168 mmol, 42% yield) as a yellow solid. $^{1}$H-NMR (500 MHz, acetone-$d_6$): $\delta$ (ppm) 7.93 (d, *J* = 8.9 Hz, 2H), 7.50–7.46 (m, 3H), 7.30 (d, *J* = 9.0 Hz, 2H), 7.17–7.15 (m, 1H), 7.14–7.09 (m, 3H), 7.04 – 7.01 (m, 1H), 5.48 (s, 2H). $^{13}$C-NMR (126 MHz, acetone-$d_6$): $\delta$ (ppm) 150.54, 142.84, 140.85, 137.02, 134.52, 132.55, 129.41, 129.28, 126.41, 122.31, 121.66, 121.24, 120.43,

117.58, 109.25, 45.45. Melting point: 195-197°C. ESI-HRMS: m/z: found 412.02195 $[M+H]^+$ (calcd for $[C_{20}H_{15}BrClN_3]^+$ 412.02161).

### 4.5. Gram-scale synthesis of *N*-substituted-2-aminobenzimidazole (6a)

(STEP 1): A mixture of *o*-phenylenediamine (**1a**) [CAS NO. 95-54-5] (1.2 eq, 7.08 mmol, 765.6 mg), tosyl chloride (**2a**) (1.2 eq, 7.08 mmol, 1349.8 mg) and potassium carbonate [CAS NO. 584-08-7] (1.2 eq, 7.08 mmol, 978.5 mg) was dissolved by co-solvent between 90% ethanol and 10% water (45.0 mL) in 100 mL round bottom flask at room temperature for 1 hour under air. (STEP 2): After that, 4-chlorophenyl isothiocyanate (**4a**) (1.0 eq, 5.90 mmol, 1000 mg) and potassium carbonate (1.0 eq, 5.90 mmol, 814.7 mg) were added into 100 mL round bottom flask as single-flask operation. The reaction was irradiated by 3W blue LED equipped with glass rod fiber at room temperature for 24 hours under air. The reaction mixture was washed water (2x20 mL) and the organic portion was extracted with EtOAc (3x20 mL). Water from organic layer was eliminated by $Na_2SO_4$. After filtration and removal of the solvent under reduced pressure, the crude product was purified by silica gel column chromatography (3:1 of hexane:EtOAc as mobile phase) to afford **6a** in 1.8889 g, 4.75 mmol, 85% yield.

### 4.6 X-ray diffraction (XRD) characterization

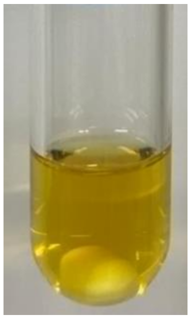

Before adding $Pb(OAc)_2$

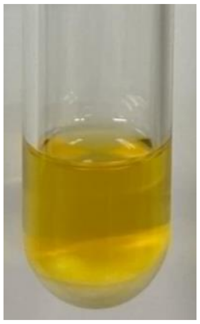

After adding $Pb(OAc)_2$

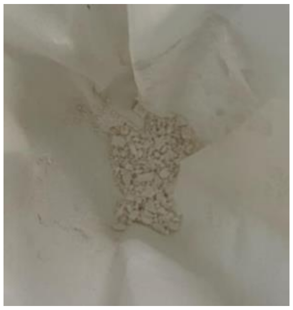

After filtration and drying

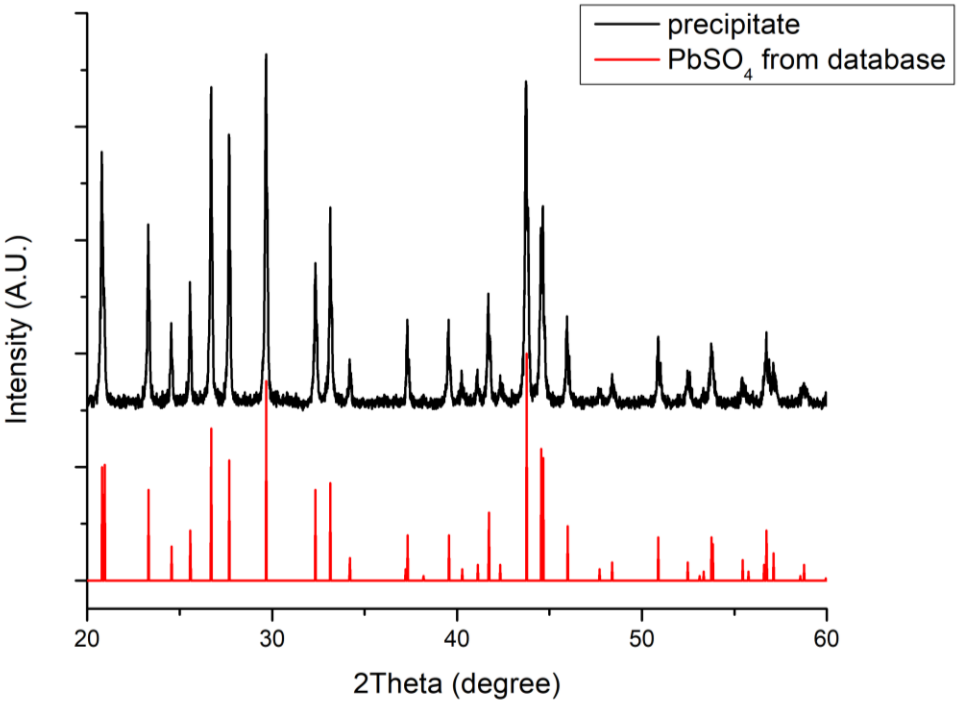


**Figure S5.** Precipitation of sulfur-byproduct and XRD pattern after adding $Pb(OAc)_2$ into reaction

## 4.7. Radical trapping experiments

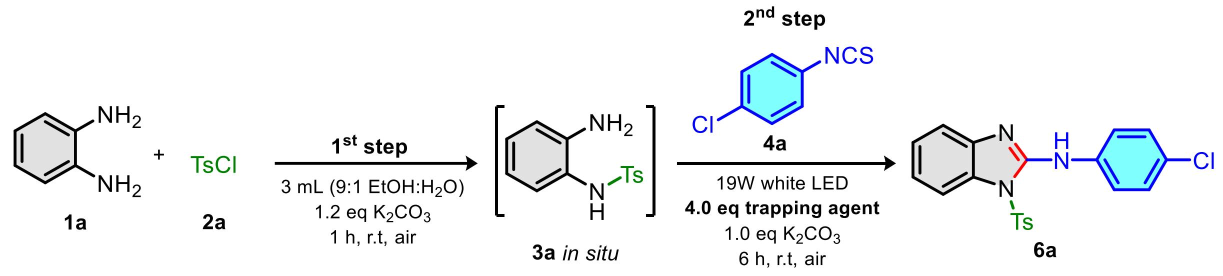


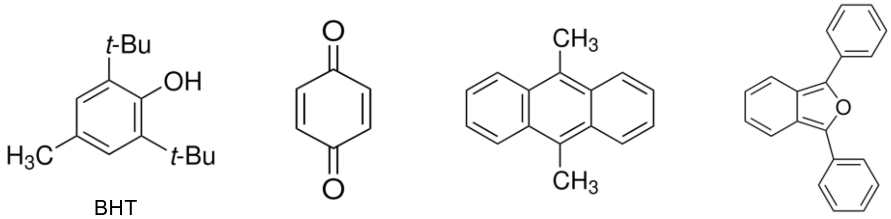


BHT (MW. 220.35)

1,4-benzoquinone (MW. 108.10)

1,9-dimethylanthracene (MW. 206.28)

1,3-diphenylisobenzofuran (MW. 270.33)

A mixture of *o*-phenylenediamine (**1a**) [CAS NO. 95-54-5] (1.2 eq, 0.48 mmol, 52 mg), tosyl chloride (**2a**) [CAS NO. 98-59-9] (1.2 eq, 0.48 mmol, 92 mg) and potassium carbonate [CAS NO. 584-08-7] (1.2 eq, 0.48 mmol, 52 mg) was dissolved by co-solvent between 90% ethanol and 10% water (3.0 mL) in pyrex schlenk reactor at room temperature for 1 hour under air. (STEP 2): After that, 4-chlorophenyl isothiocyanate (**4a**) (1.0 eq, 0.40 mmol, 68 mg), corresponding radical trapping (4.0 eq, 1.60 mmol) and potassium carbonate (1.0 eq, 0.40 mmol, 55.3 mg) were added into pyrex schlenk reactor as single-flask operation. The reaction was irradiated by 3W blue LED equipped with glass rod fiber at room temperature for 6 hours under air. The reaction mixture was washed water (2x10 mL) and the organic portion was extracted with EtOAc (3x10 mL). Water from organic layer was eliminated by $Na_2SO_4$. After filtration and removal of the solvent under reduced pressure, the crude product was purified by silica gel column chromatography (3:1 of hexane:EtOAc) to afford *N*-substituted-2-aminobenzimidazoles **6a** as corresponding product.

## 4.8 EPR experiments

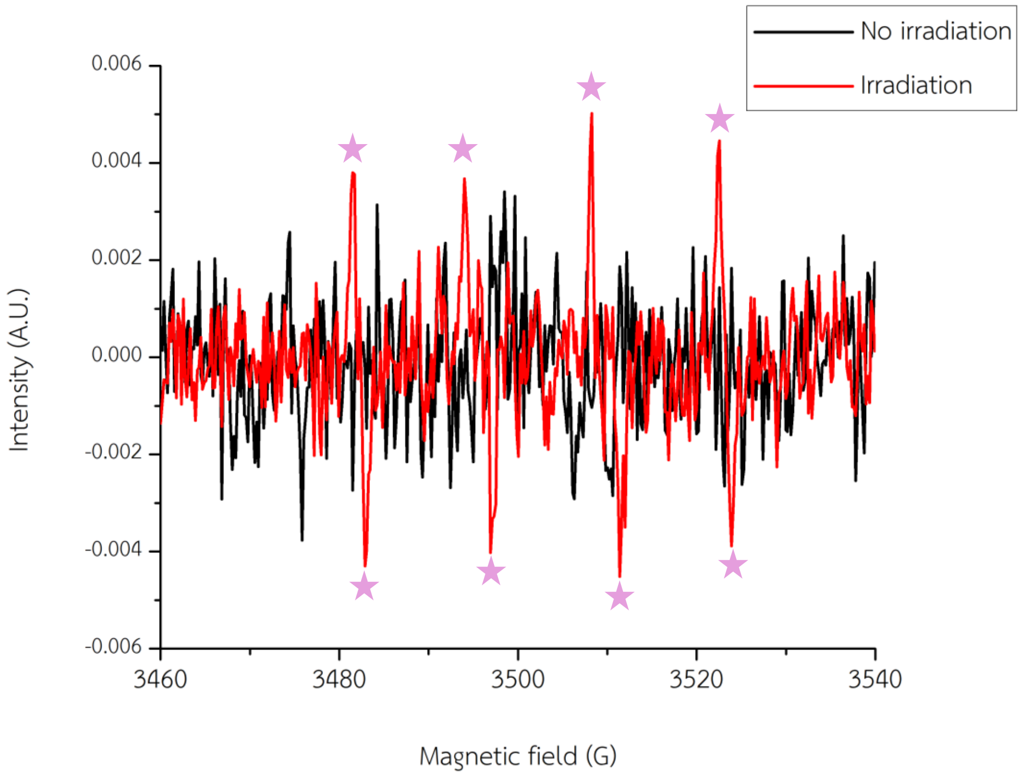


**Figure S6.** EPR spectrum: **3a** (0.80 mmol), **4a** (0.40 mmol), DMPO (0.80 mmol), $K_2CO_3$ (0.80 mmol), 3 mL (9:1 EtOH:$H_2O$), r.t and air under 19W white LED irradiation for 30 min. (black) no irradiation and (red) irradiation

### 4.9 UV-vis spectroscopic measurements

The UV-vis absorption spectra of **3a** (5 mM), **4a** (5 mM), **3a**+**4a** (5 mM) and **5a** (5 mM) with and without $K_2CO_3$ in 9:1 (EtOH:$H_2O$) were recorded in 1 cm path quartz cuvettes by UV-vis spectrophotometer.

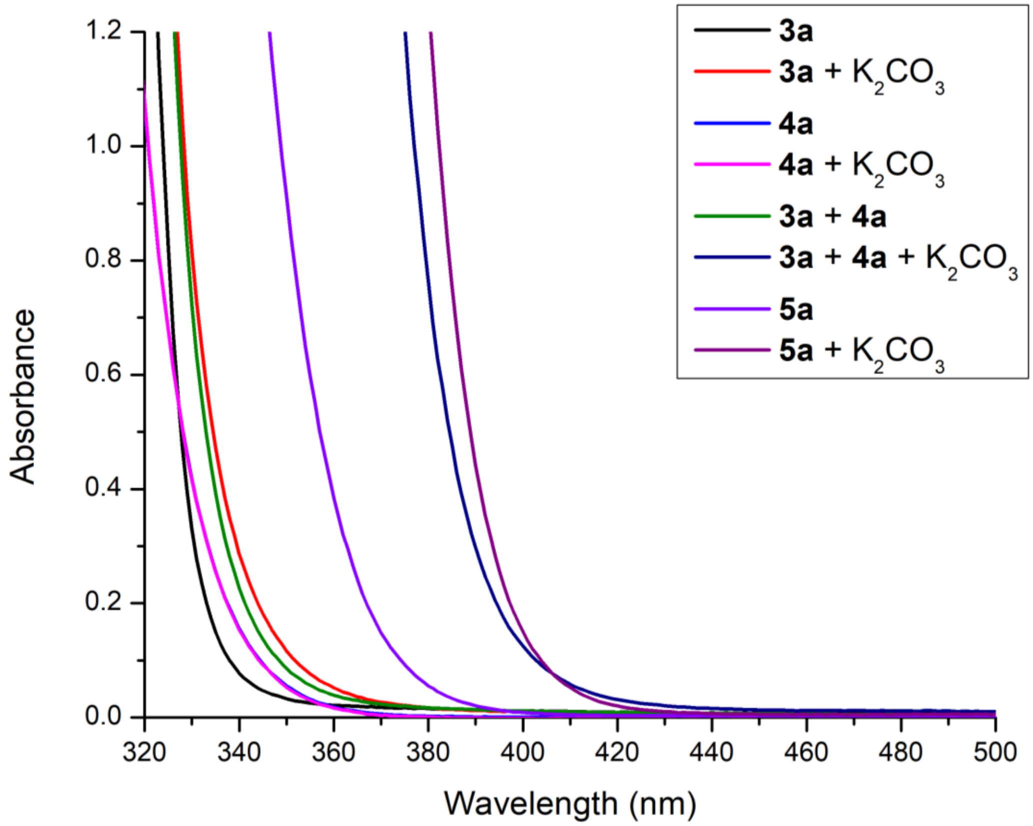


**Figure S7**. UV-vis absorption spectra of **3a**, **4a**, **3a**+**4a** and **5a** with and without $K_2CO_3$ in 9:1 (EtOH:$H_2O$) at 5 mM

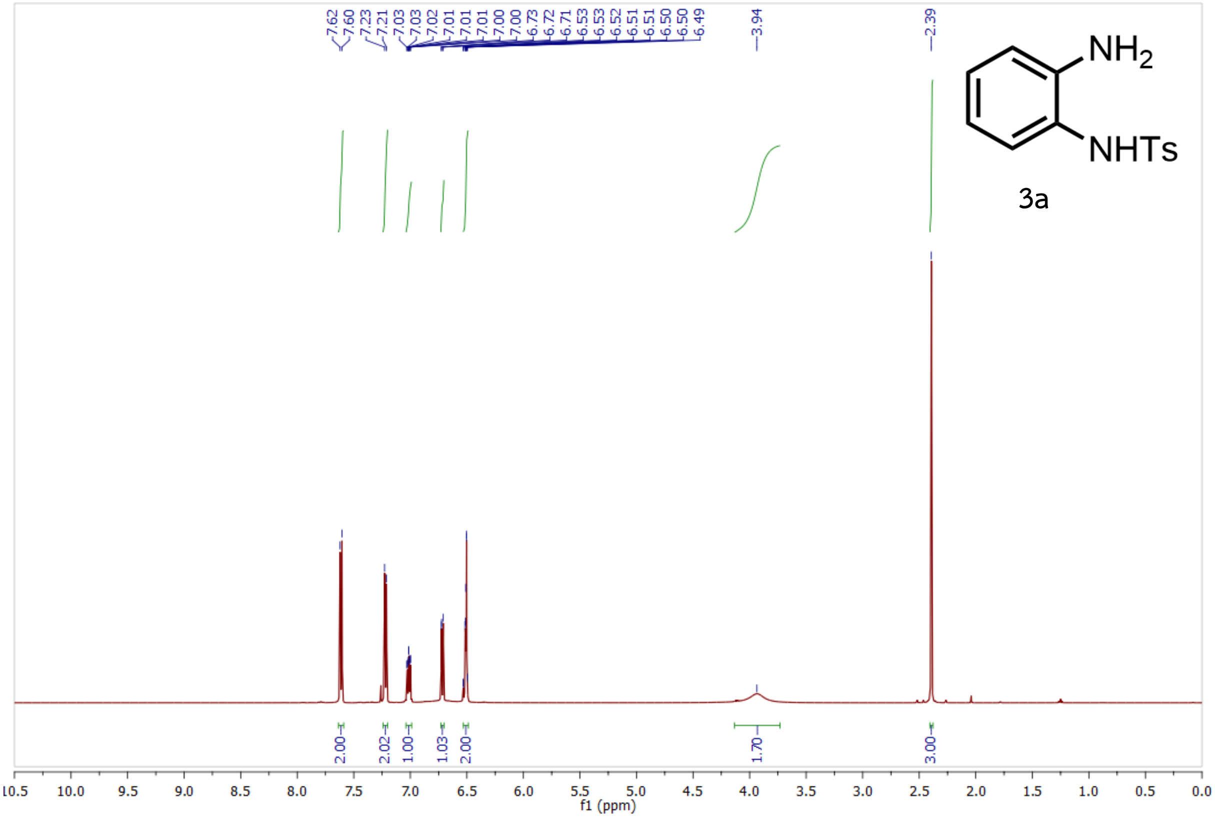


**Figure S8** $^{1}$H-NMR spectrum of **3a** ($CDCl_3$, 500 MHz)

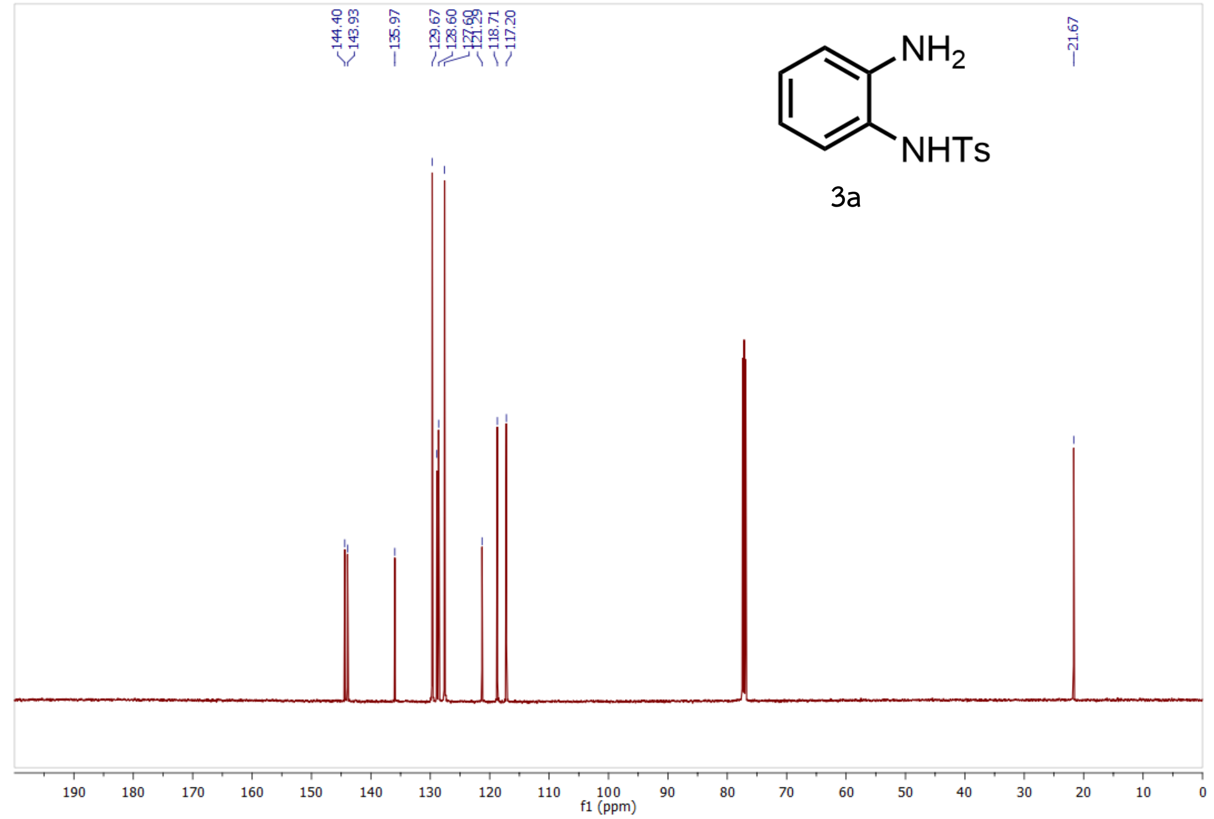


**Figure S9** $^{13}$C-NMR spectrum of **3a** ($CDCl_3$, 126 MHz)

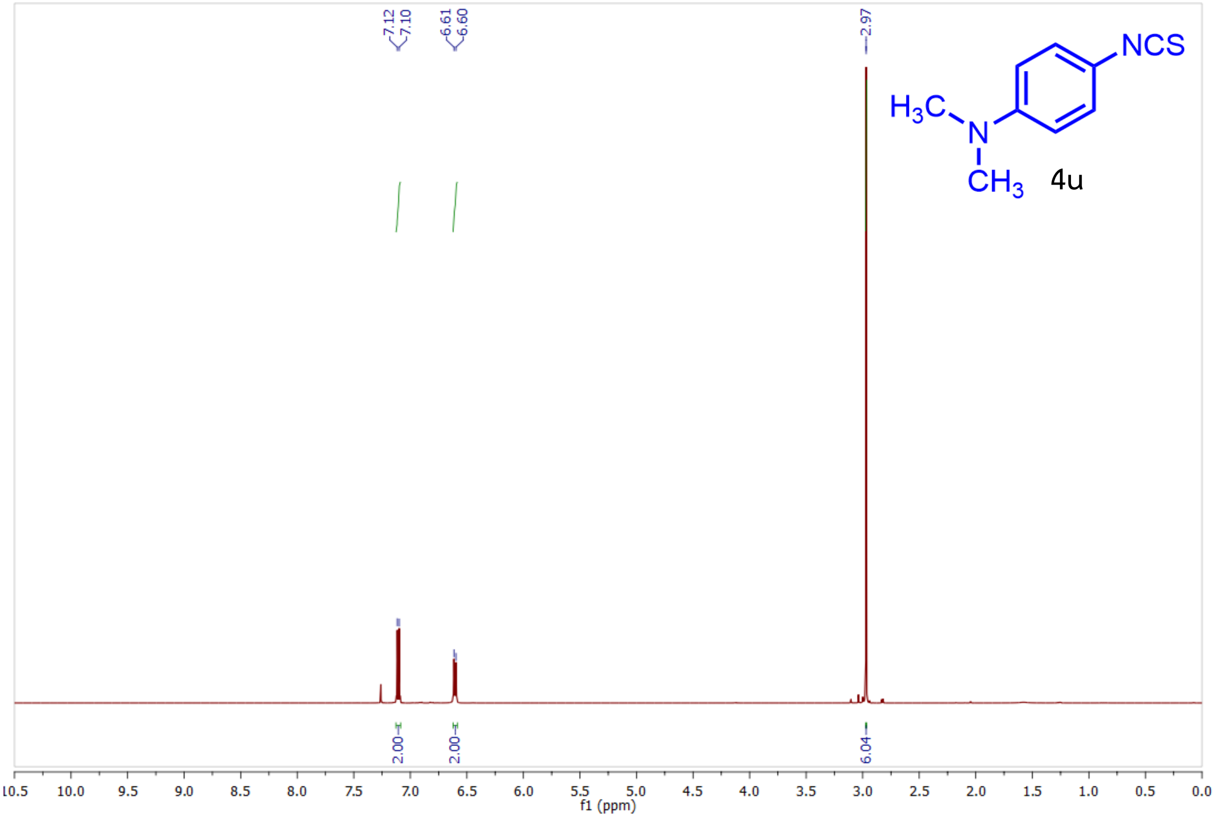


**Figure S10** $^{1}$H-NMR spectrum of **4u** ($CDCl_3$, 500 MHz)

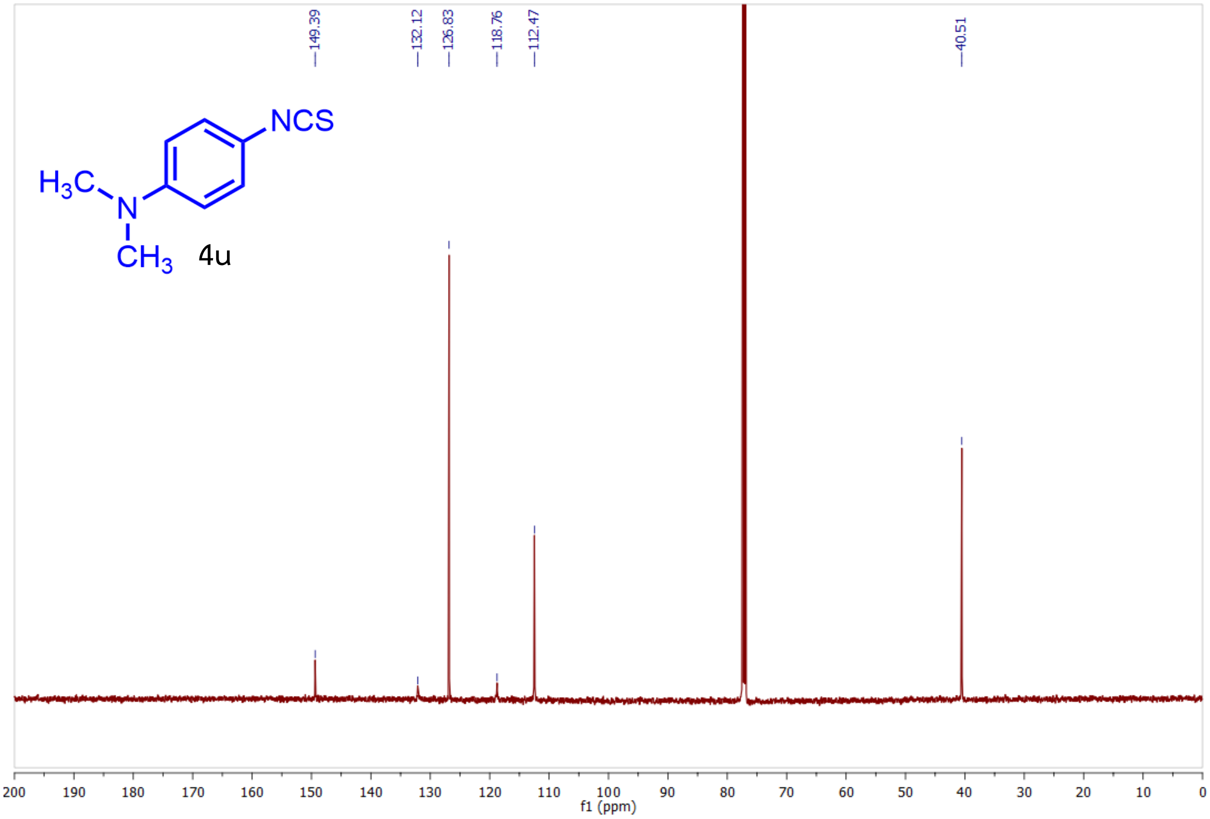


**Figure S11** $^{13}$C-NMR spectrum of **4u** ($CDCl_3$, 126 MHz)

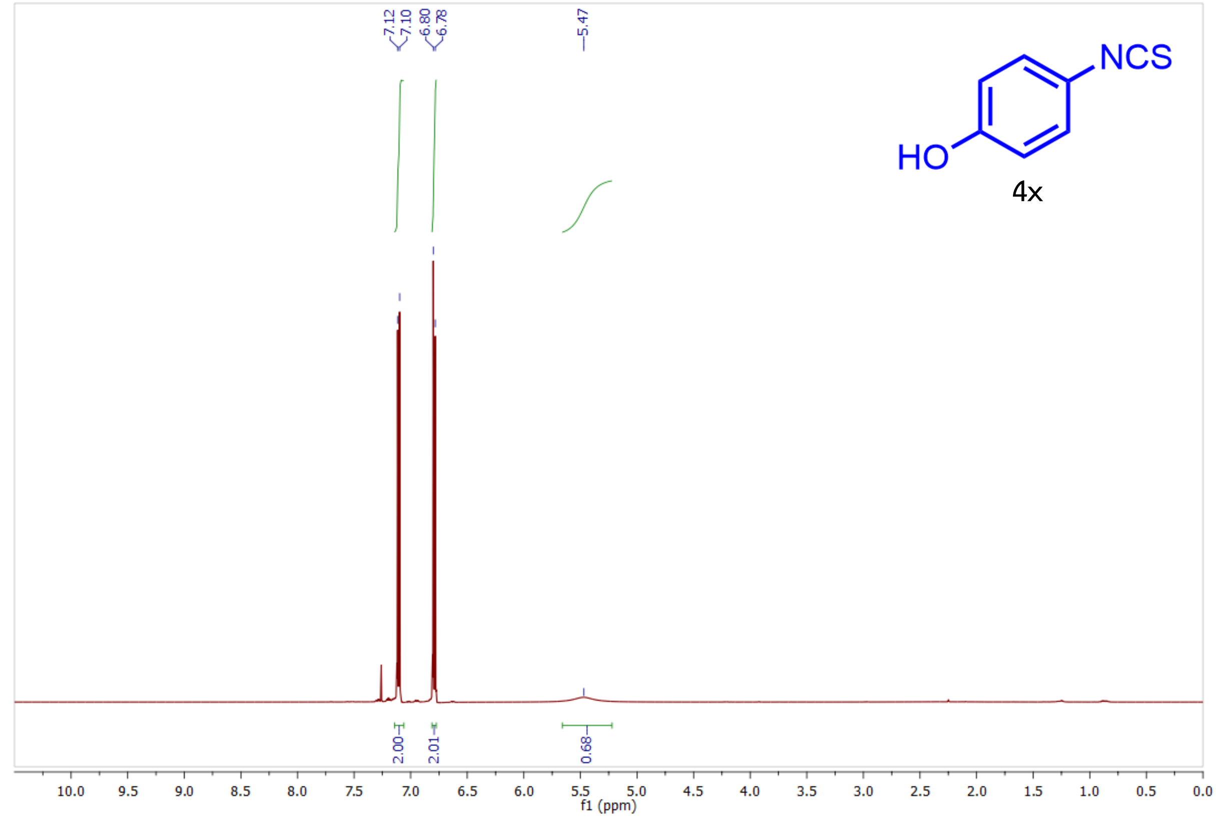


**Figure S12** $^{1}$H-NMR spectrum of **4x** ($CDCl_3$, 500 MHz)

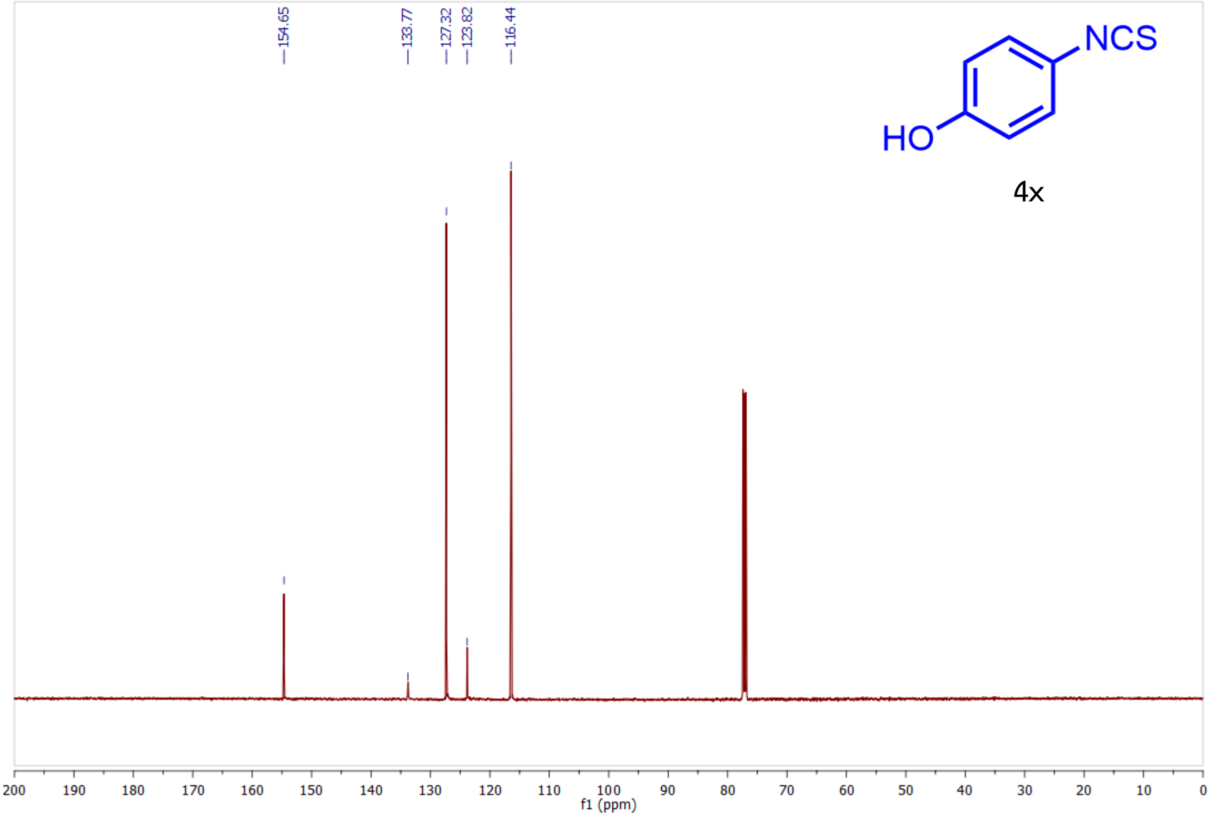


**Figure S13** $^{13}$C-NMR spectrum of **4x** ($CDCl_3$, 126 MHz)

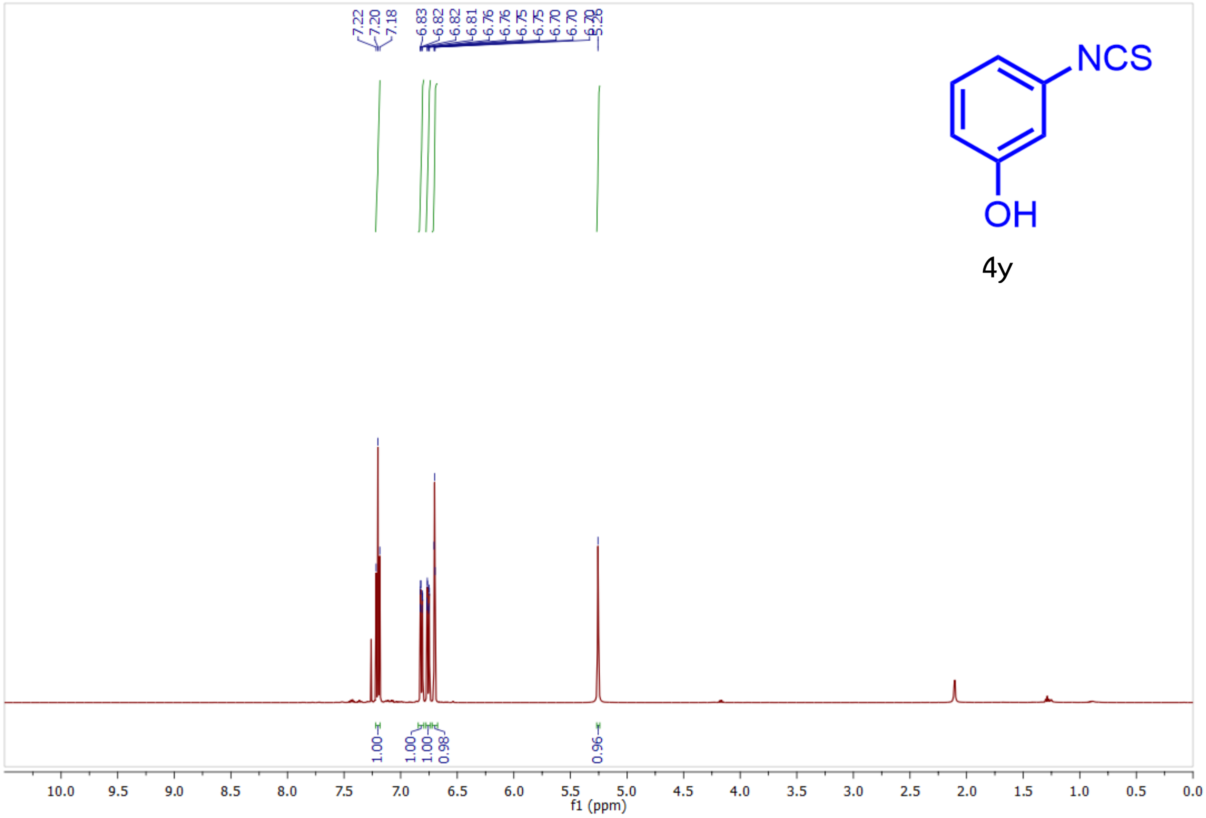


**Figure S14** $^{1}$H-NMR spectrum of **4y** ($CDCl_3$, 500 MHz)

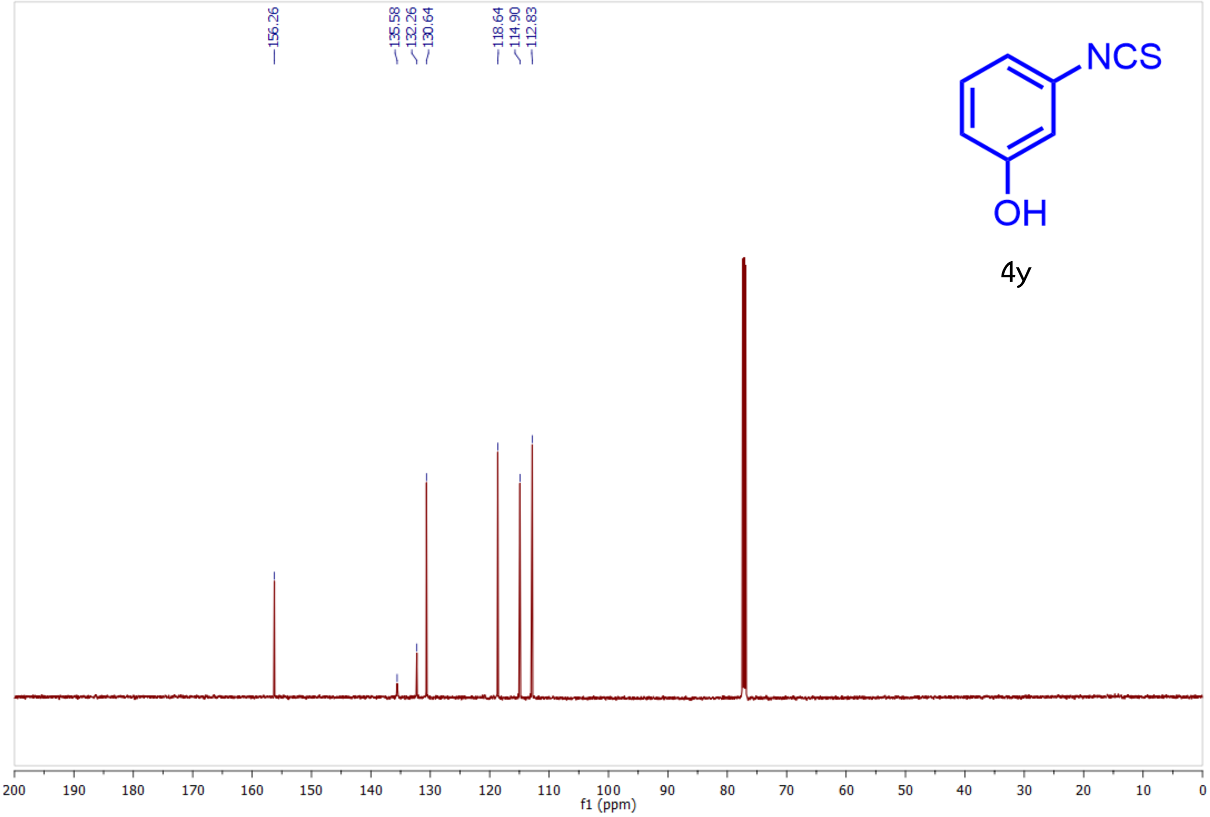


**Figure S15** $^{13}$C-NMR spectrum of **4y** ($CDCl_3$, 126 MHz)

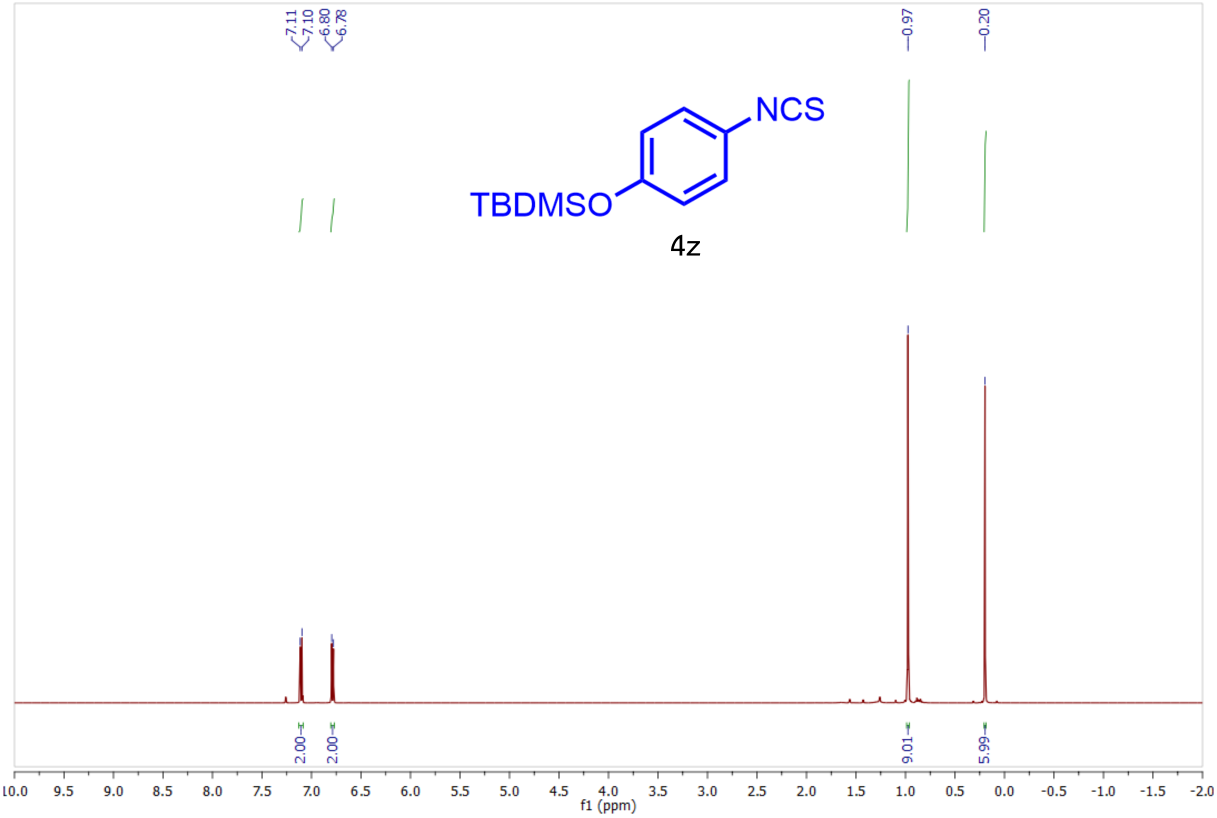


**Figure S16** $^{1}$H-NMR spectrum of **4z** ($CDCl_3$, 500 MHz)

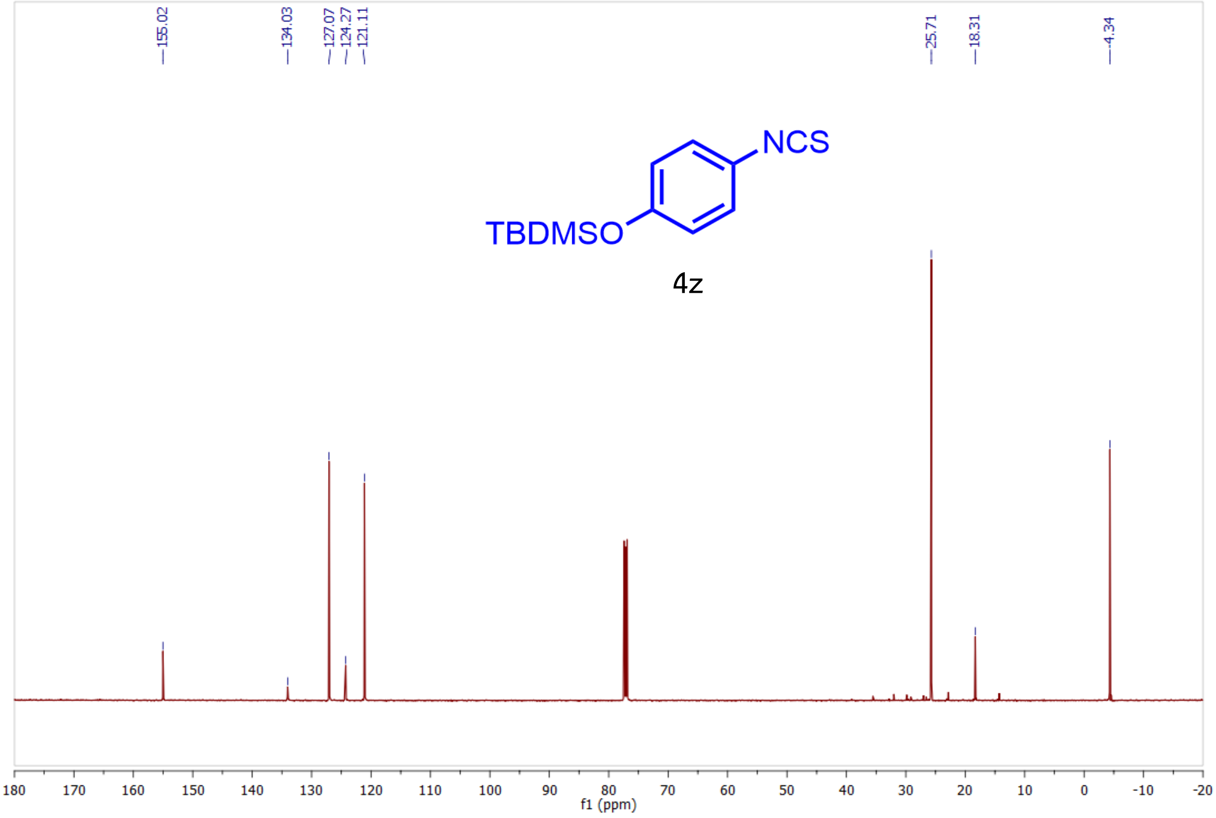


**Figure S17** $^{13}$C-NMR spectrum of **4z** ($CDCl_3$, 126 MHz)

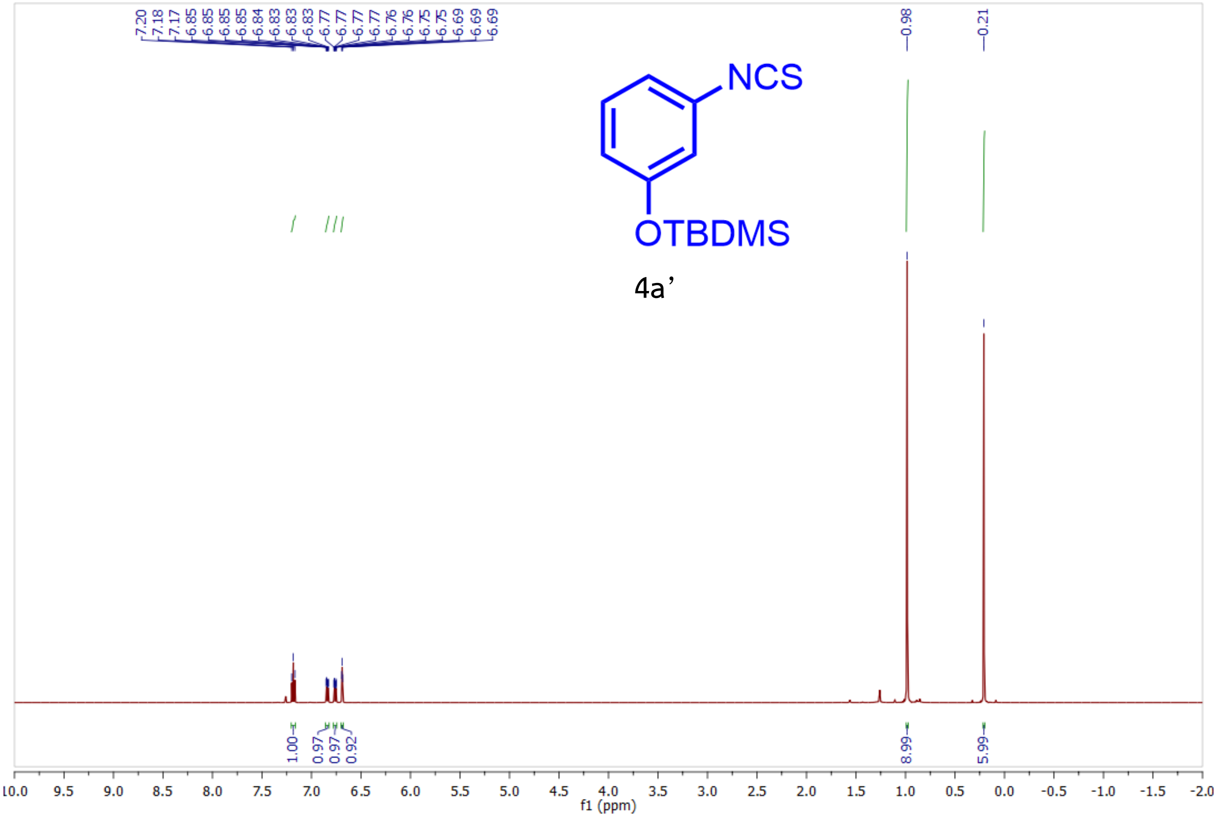

**Figure S18** $^{1}$H-NMR spectrum of 4**a'** (CDCl$_3$, 500 MHz)

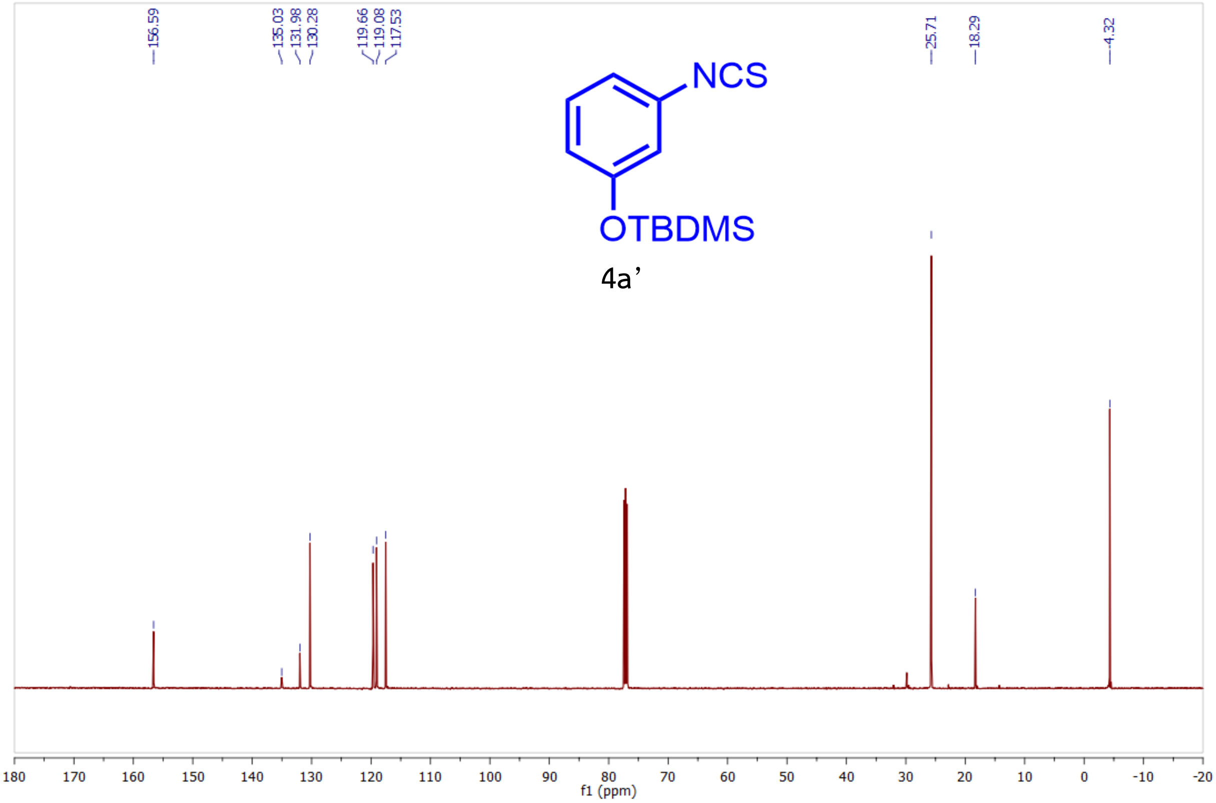

**Figure S19** $^{13}$C-NMR spectrum of **4a'** (CDCl$_3$, 126 MHz)

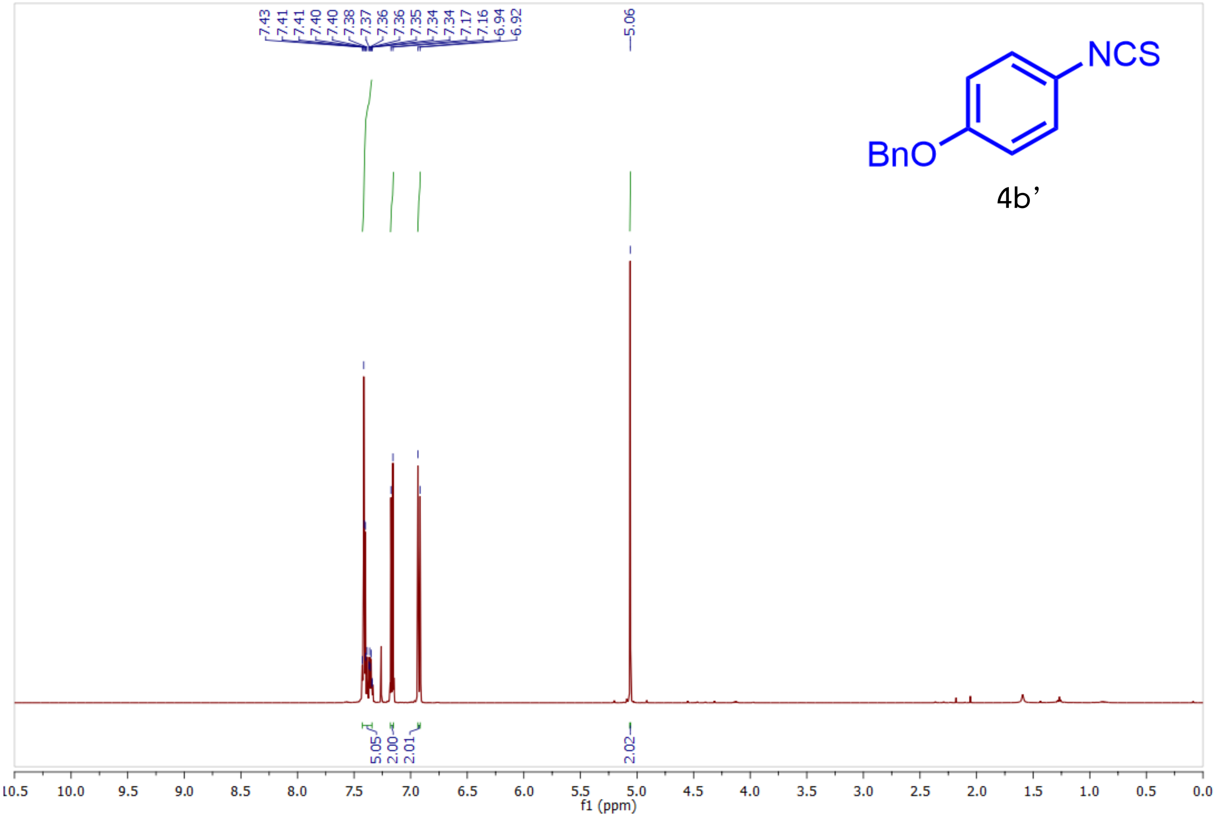


**Figure S20** $^{1}$H-NMR spectrum of **4b’** ($CDCl_3$, 500 MHz)

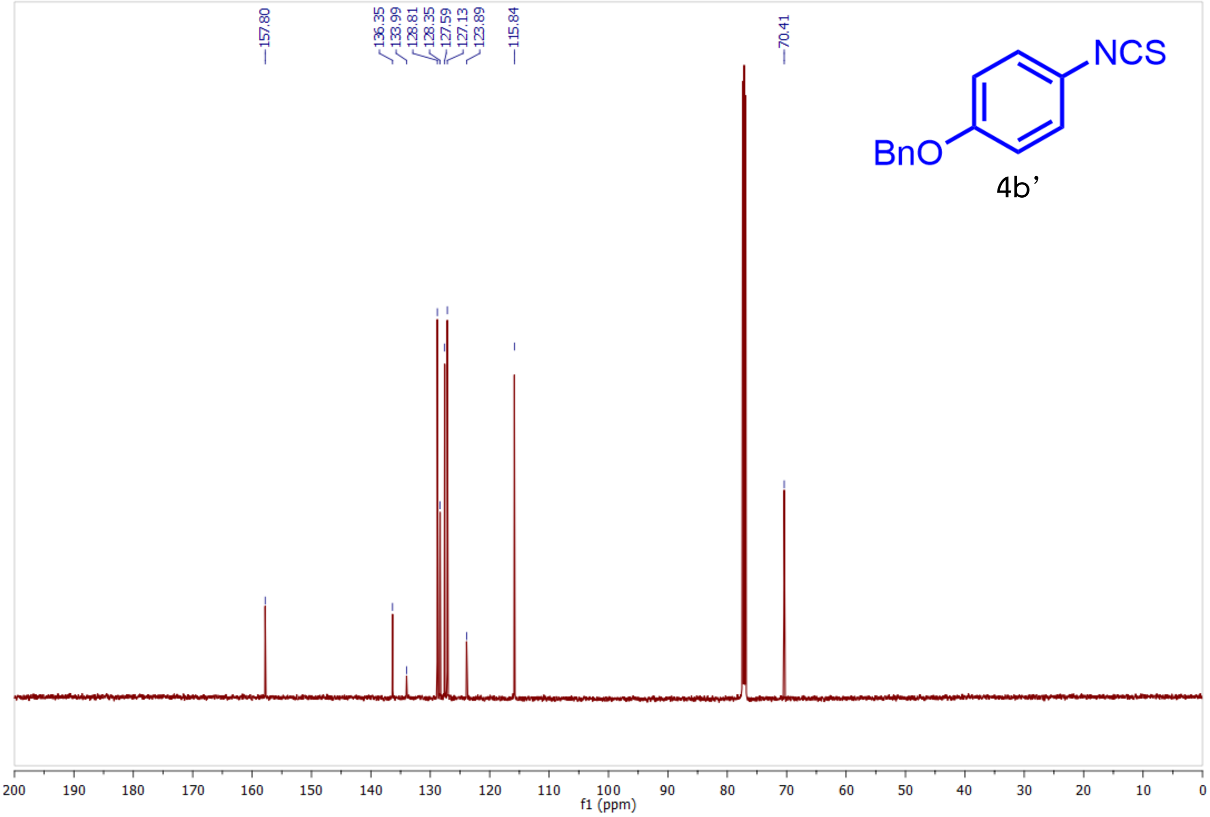


**Figure S21** $^{13}$C-NMR spectrum of **4b’** ($CDCl_3$, 126 MHz)

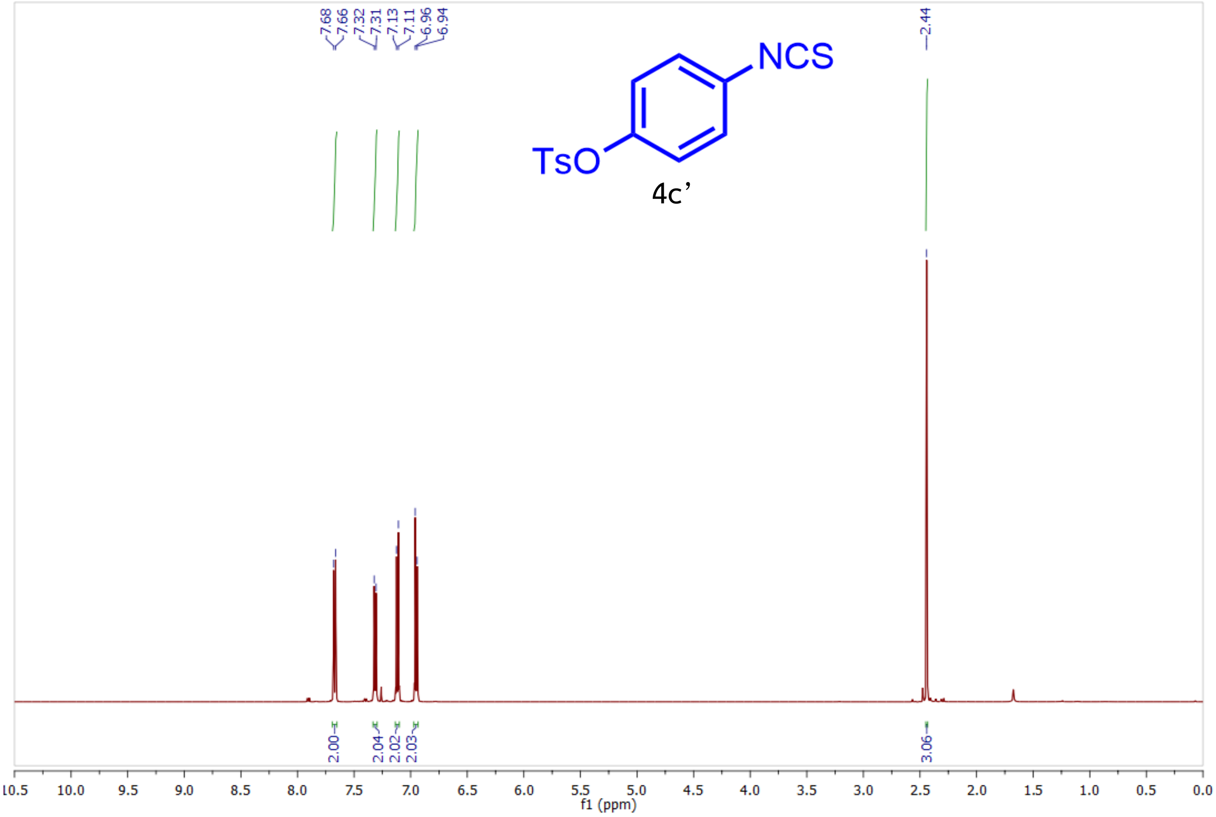


**Figure S22** $^{1}$H-NMR spectrum of **4c'** ($CDCl_3$, 500 MHz)

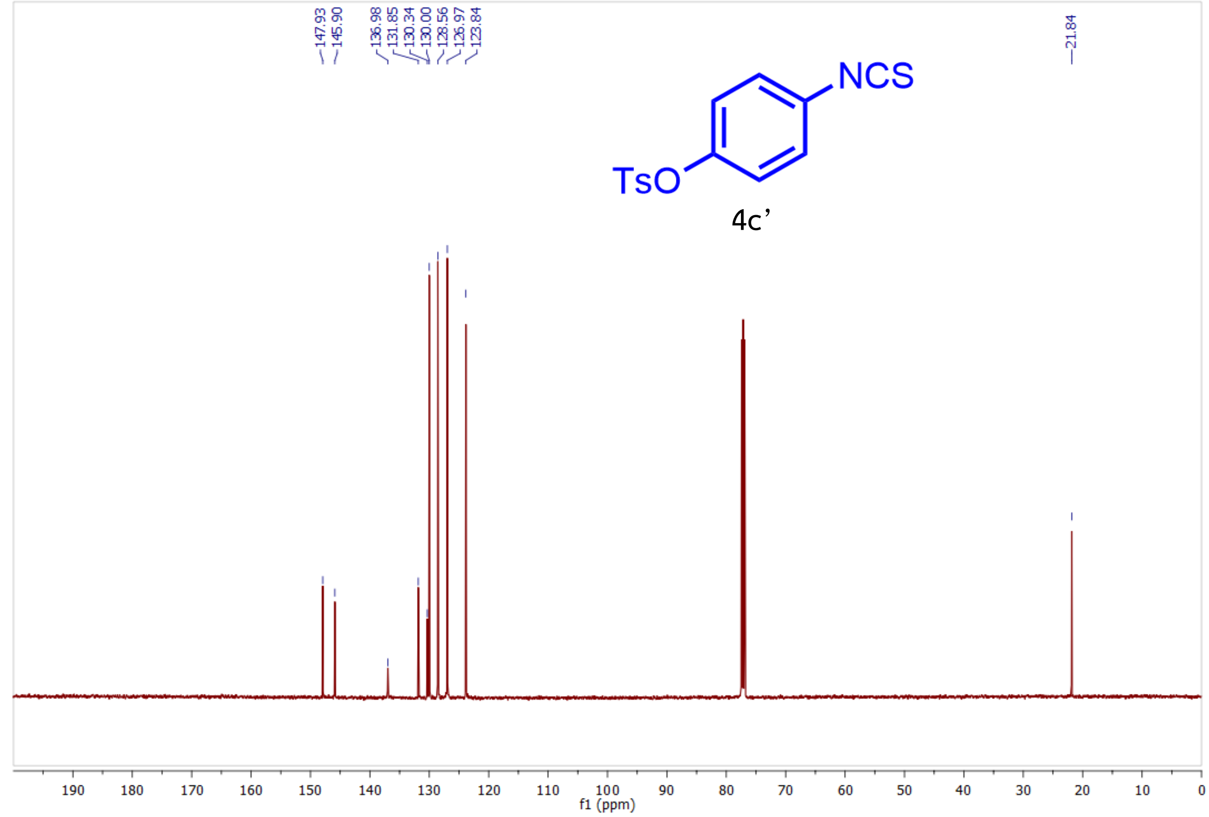


**Figure S23** $^{13}$C-NMR spectrum of **4c'** ($CDCl_3$, 126 MHz)

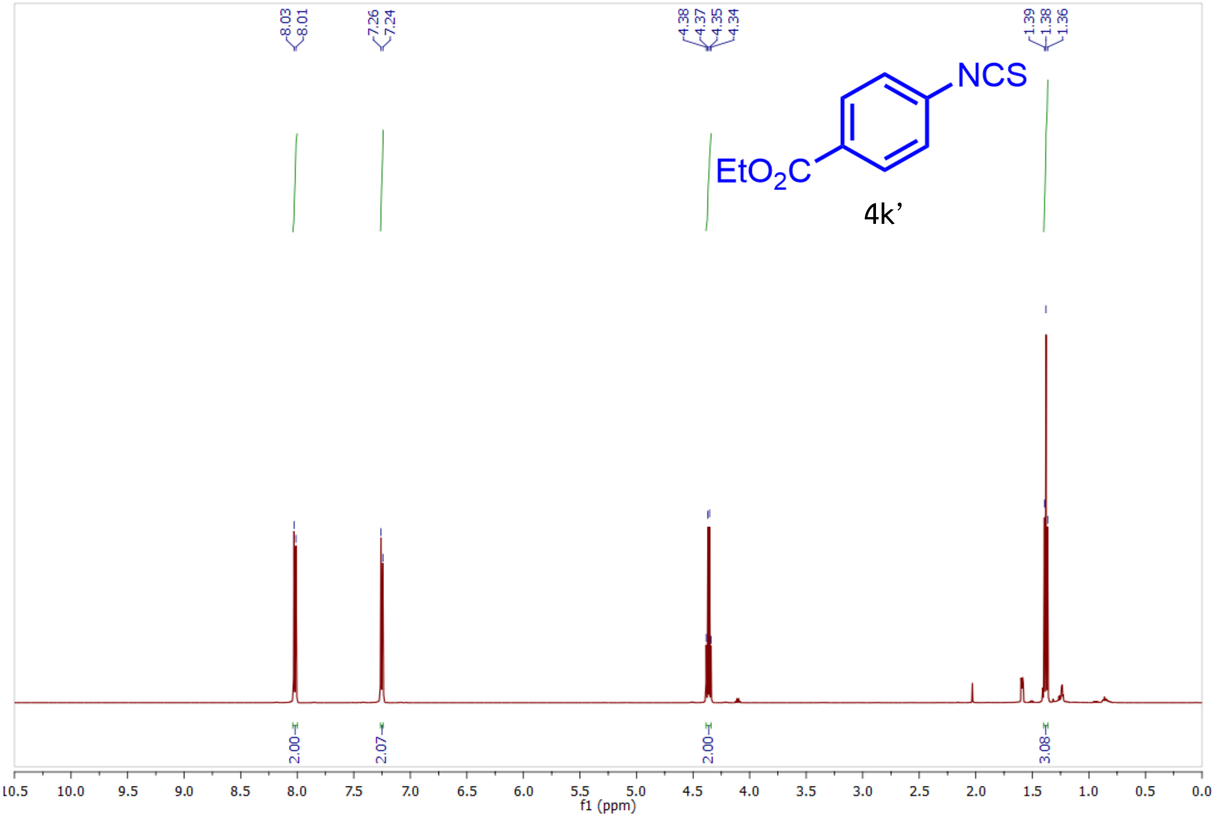


**Figure S24** $^{1}$H-NMR spectrum of **4k’** ($CDCl_3$, 500 MHz)

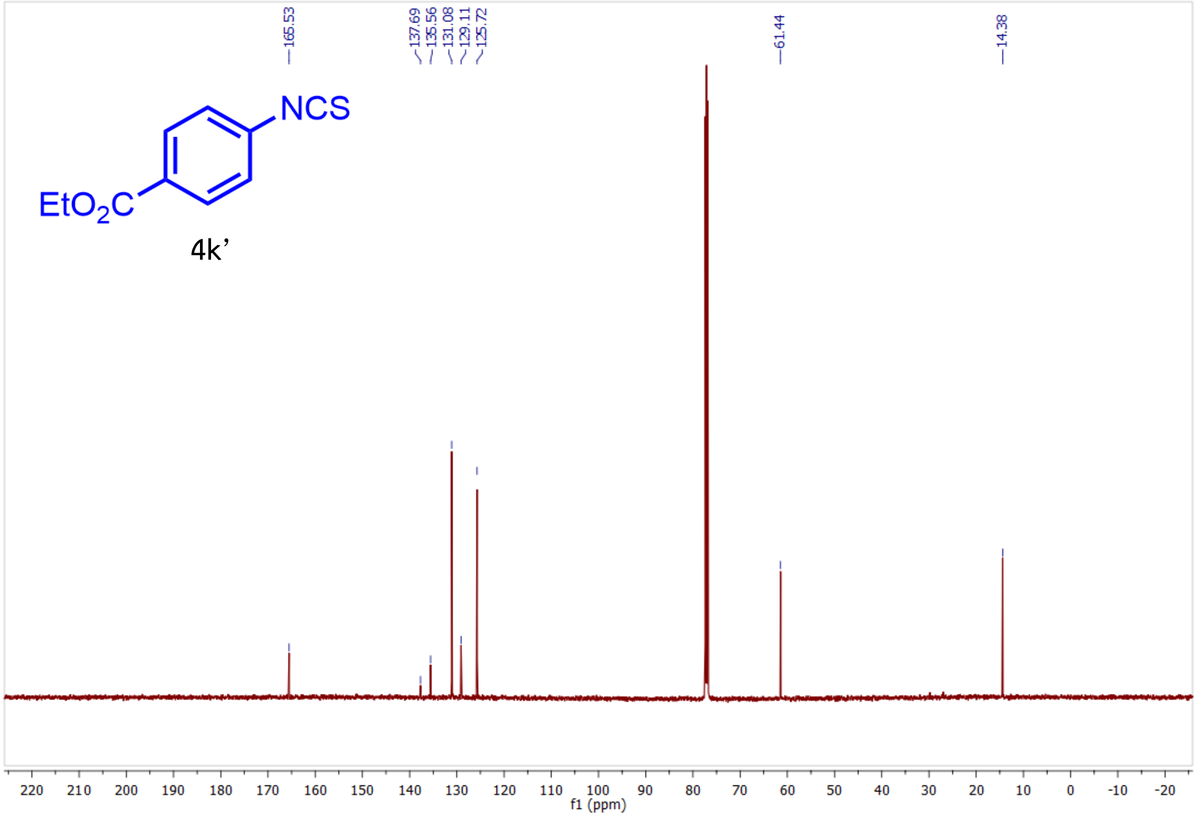


**Figure S25** $^{13}$C-NMR spectrum of **4k’** ($CDCl_3$, 126 MHz)

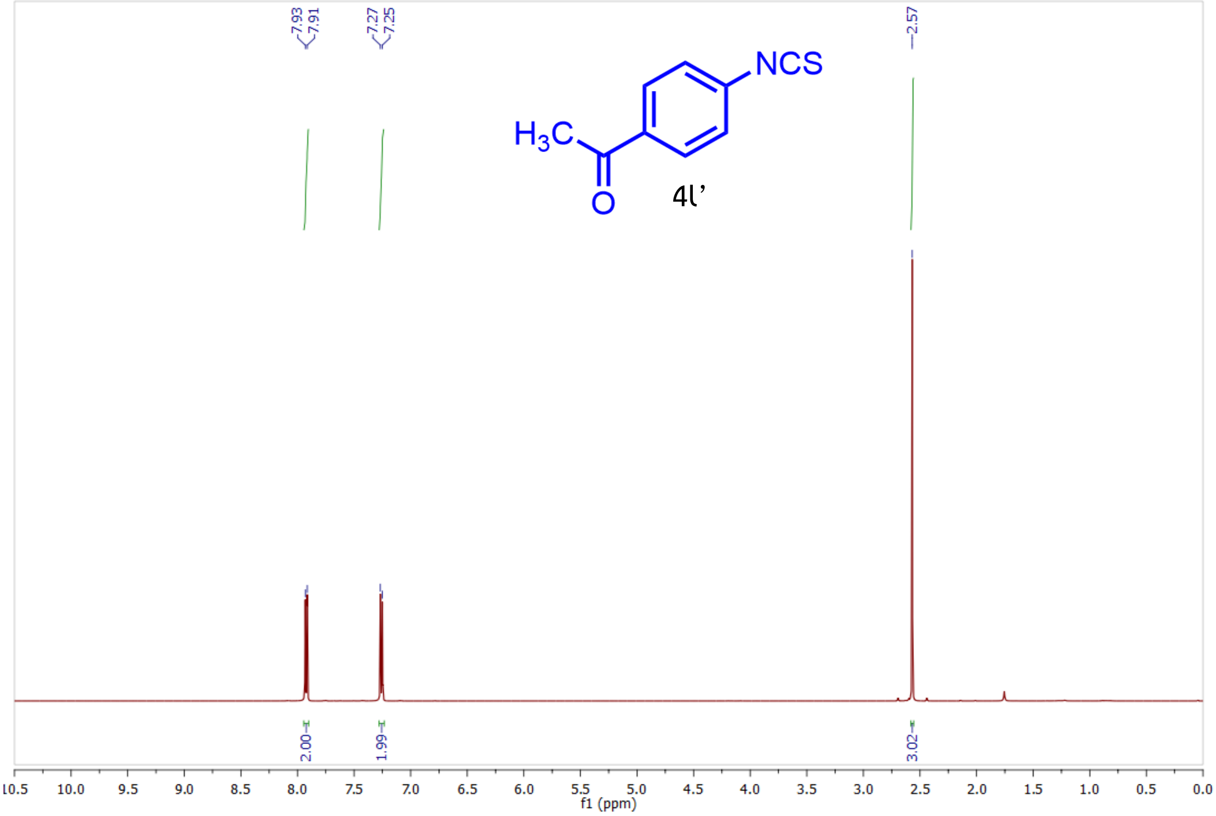


**Figure S26** $^{1}$H-NMR spectrum of **4l'** ($CDCl_3$, 500 MHz)

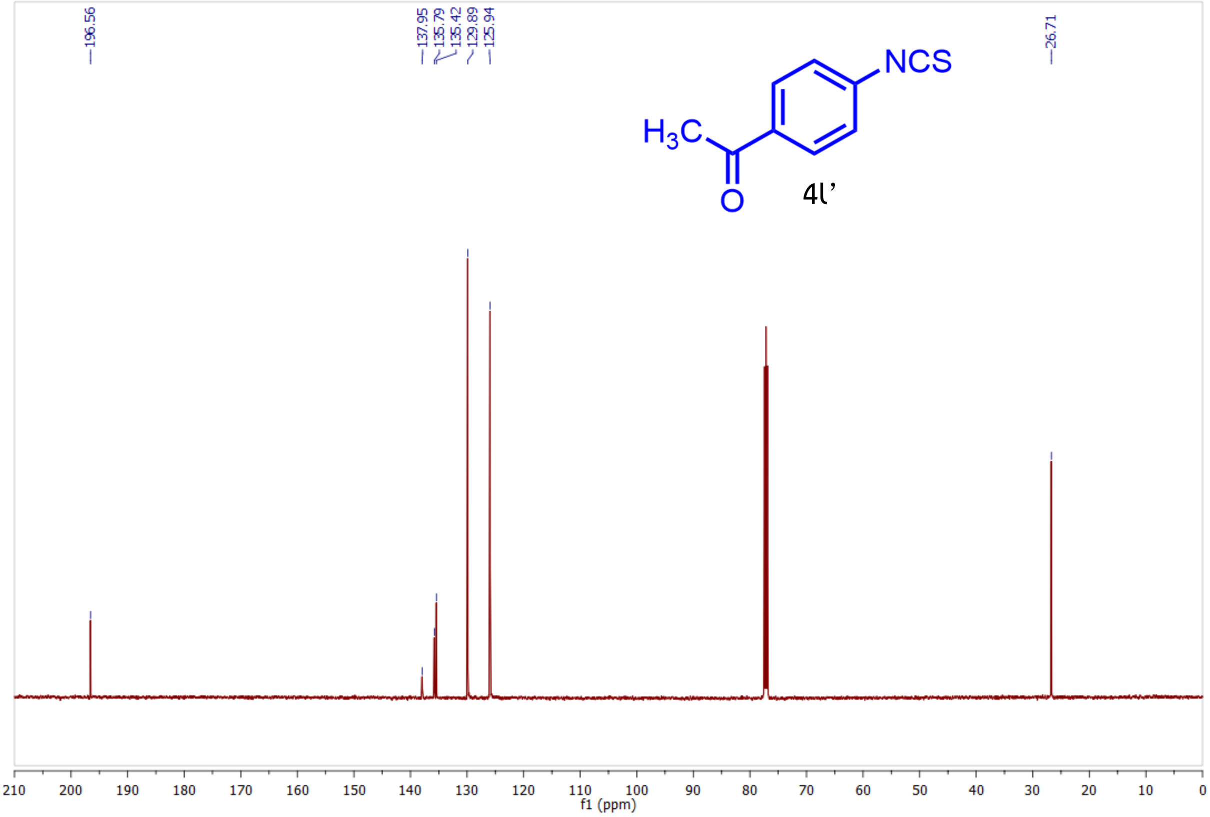


**Figure S27** $^{13}$C-NMR spectrum of **4l'** ($CDCl_3$, 126 MHz)

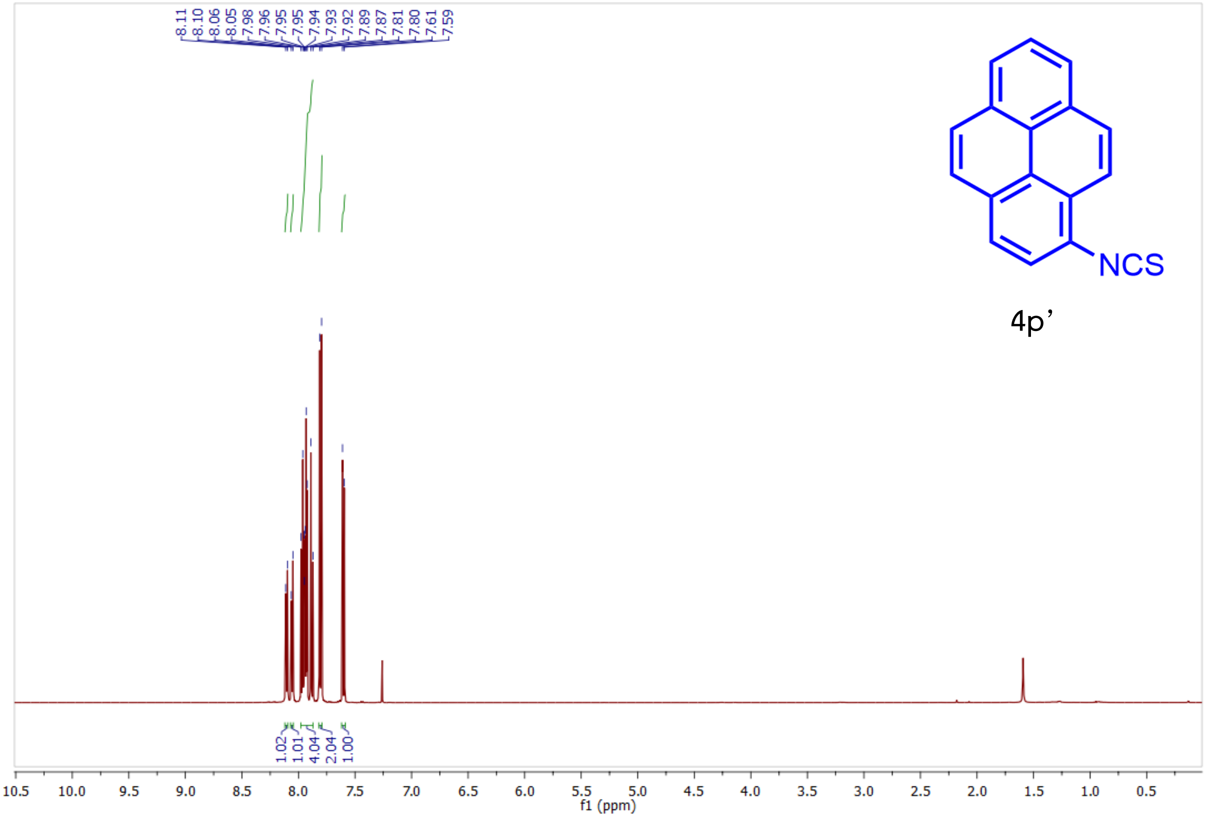


**Figure S28** $^{1}$H-NMR spectrum of **4p'** ($CDCl_3$, 500 MHz)

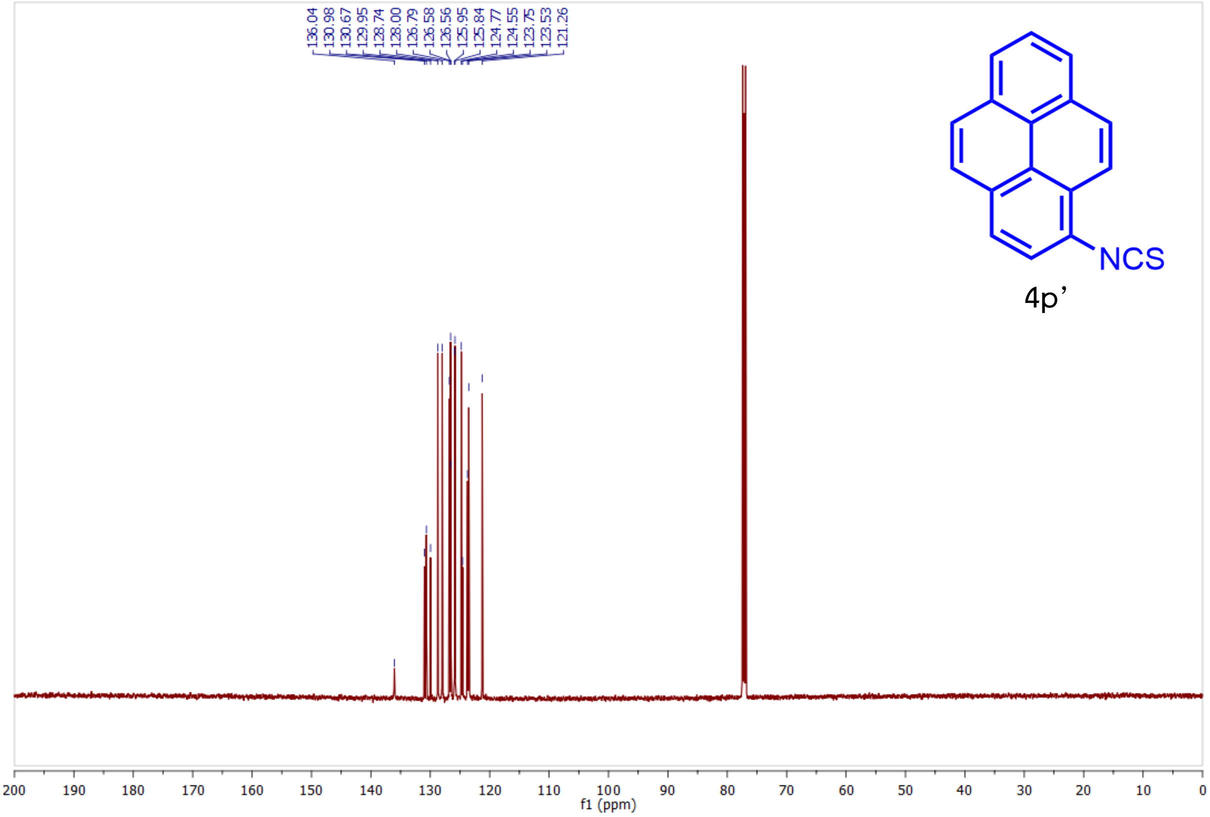


**Figure S29** $^{13}$C-NMR spectrum of **4p'** ($CDCl_3$, 126 MHz)

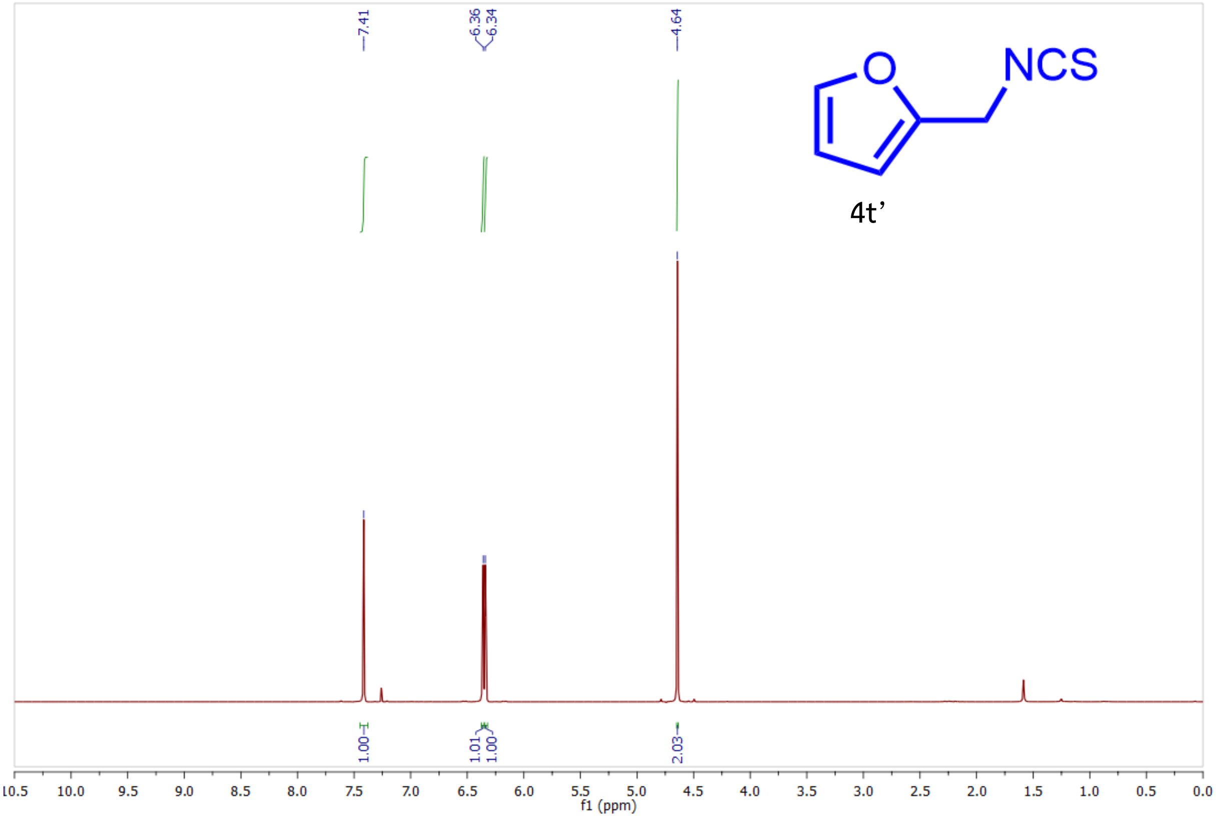


**Figure S30** $^{1}$H-NMR spectrum of **4t’** ($CDCl_3$, 500 MHz)

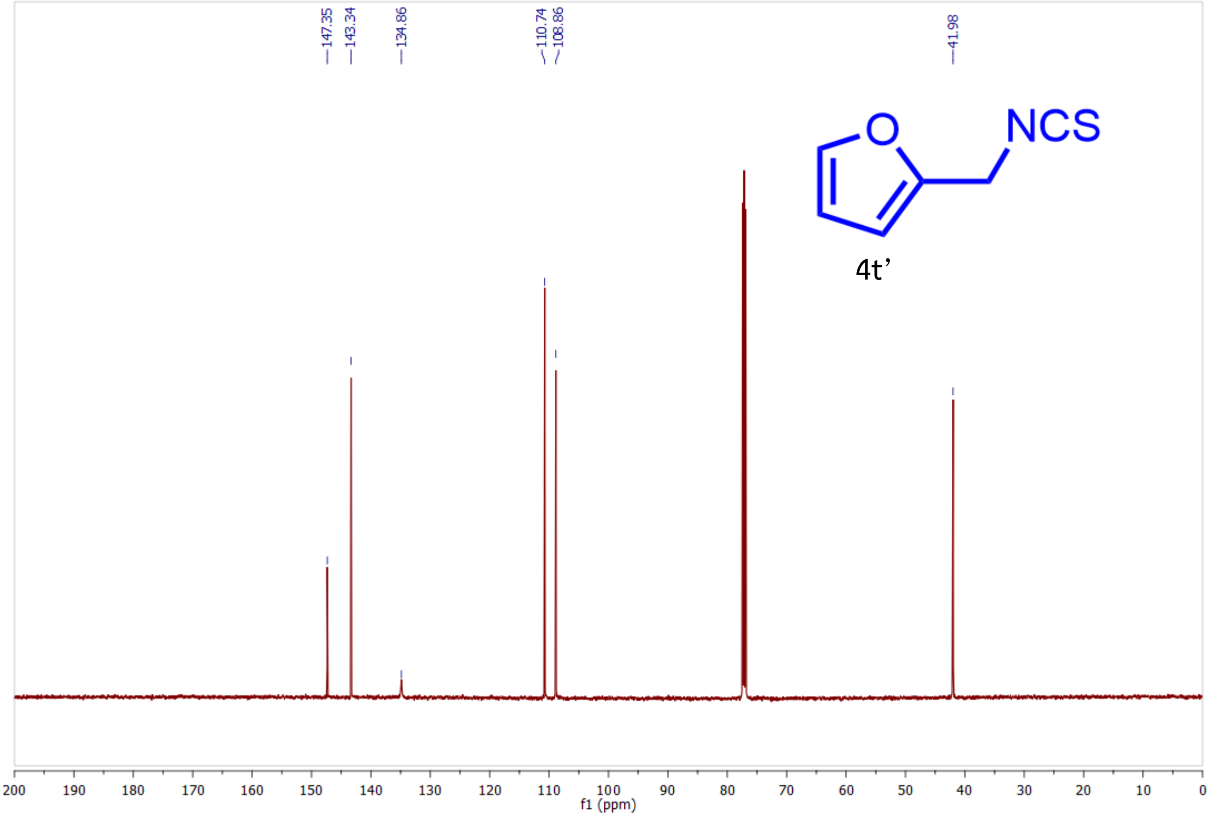


**Figure S31** $^{13}$C-NMR spectrum of **4t’** ($CDCl_3$, 126 MHz)

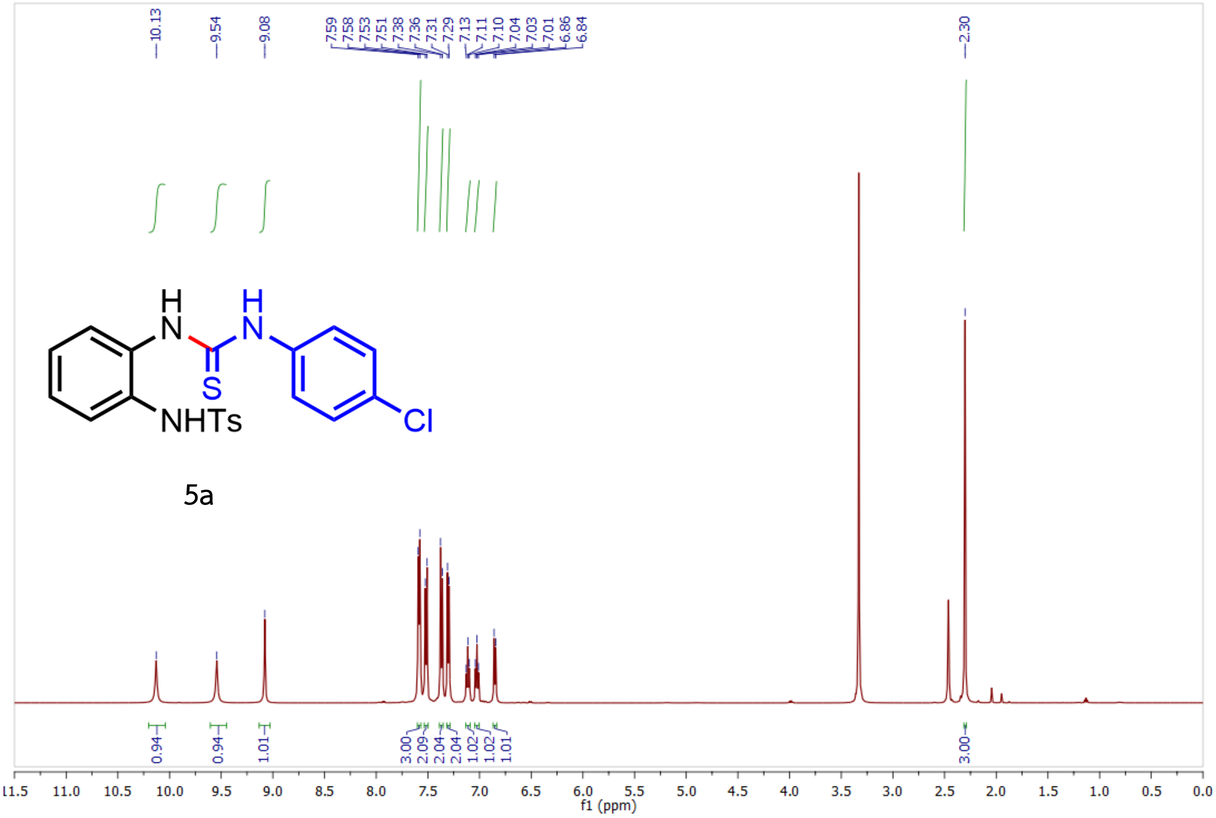


**Figure S32** $^{1}$H-NMR spectrum of **5a** (DMSO-$d_6$, 500 MHz)

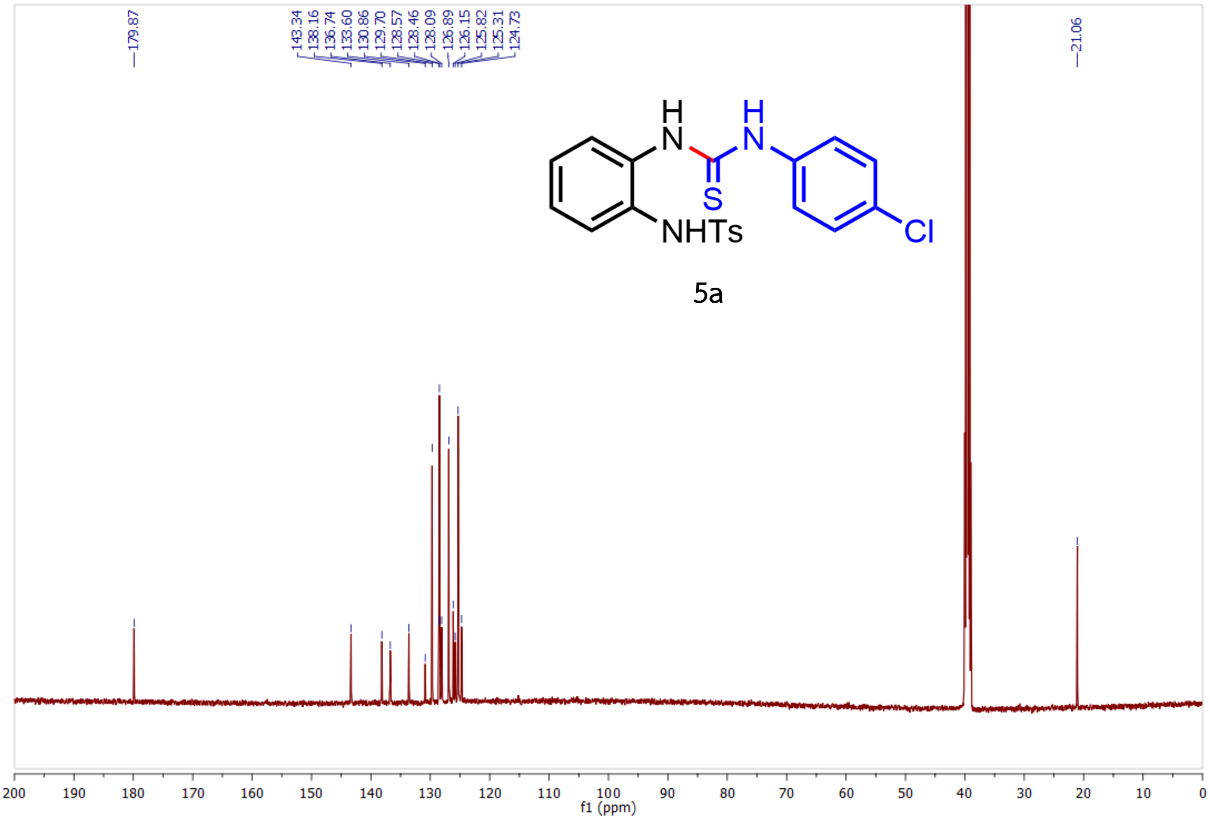


**Figure S33** $^{13}$C-NMR spectrum of **5a** (DMSO-$d_6$, 126 MHz)

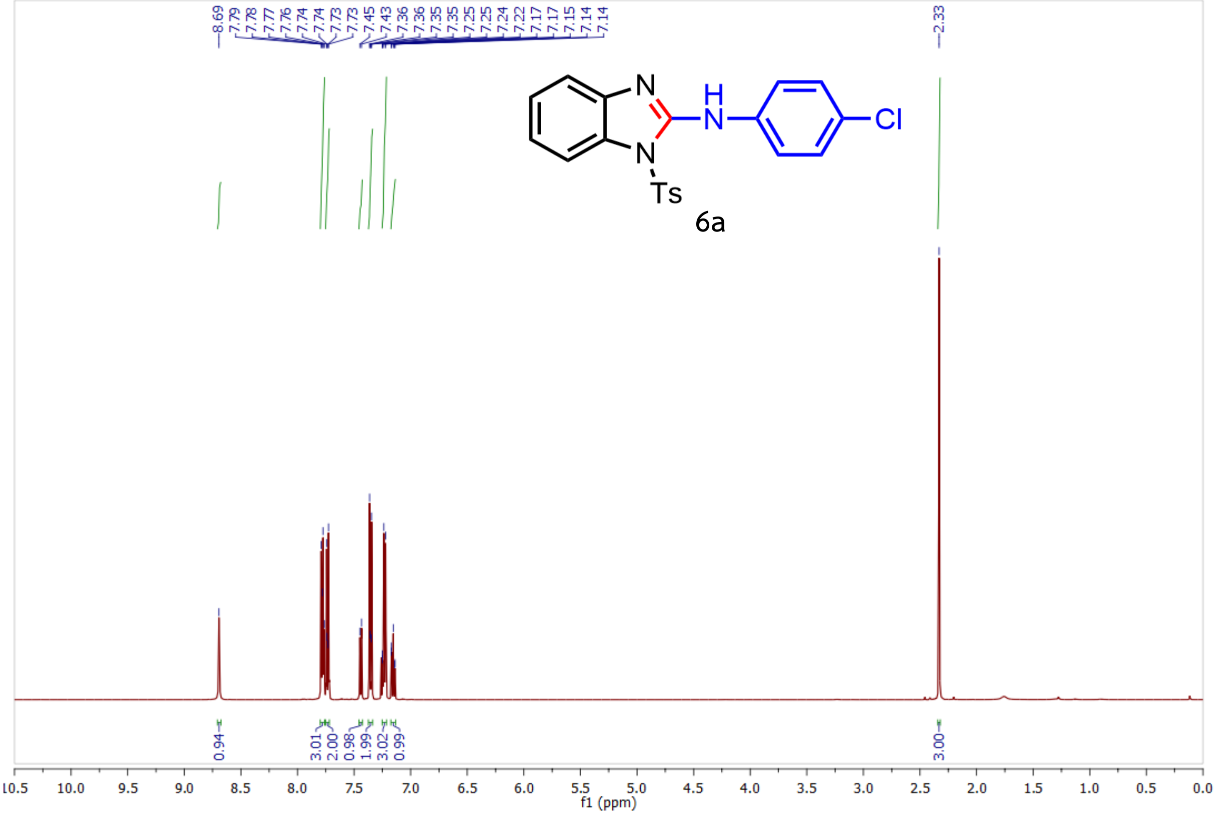


**Figure S34** $^{1}$H-NMR spectrum of **6a** ($CDCl_3$, 500 MHz)

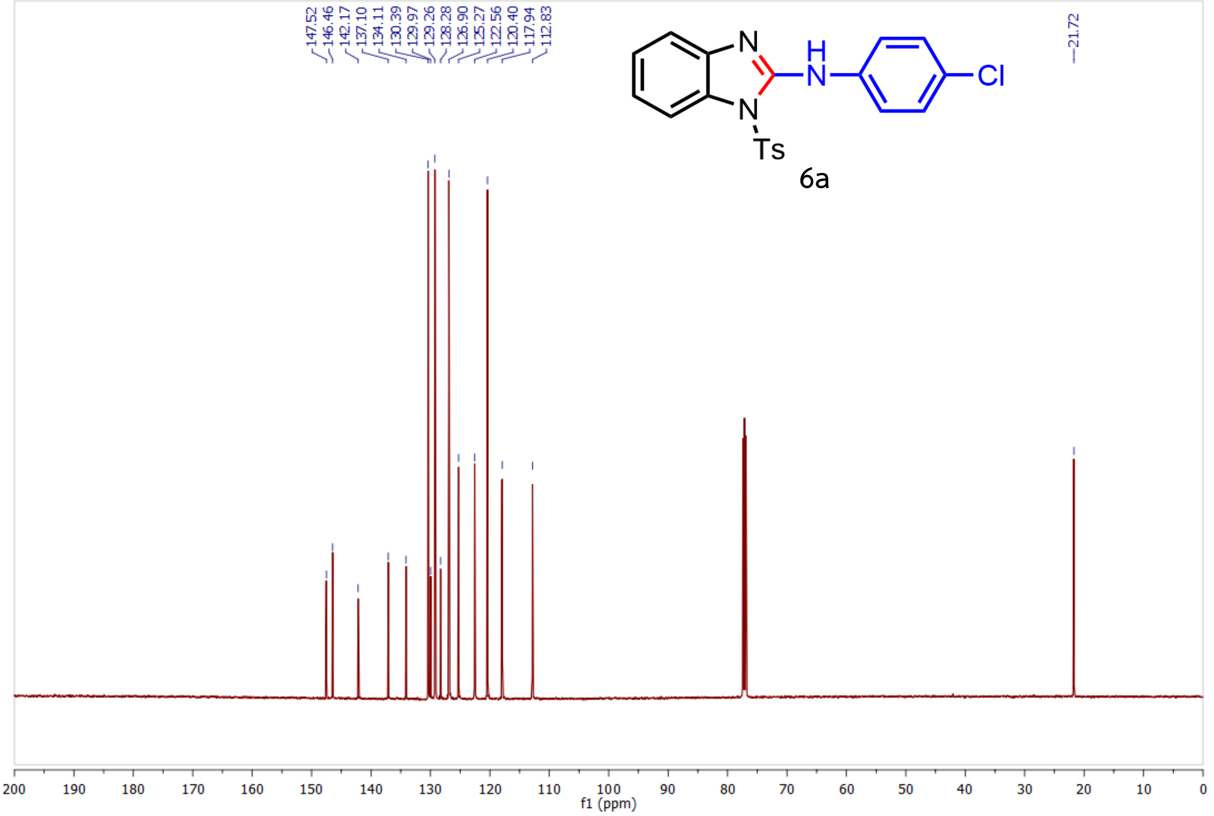


**Figure S35** $^{13}$C-NMR spectrum of **6a** ($CDCl_3$, 126 MHz)

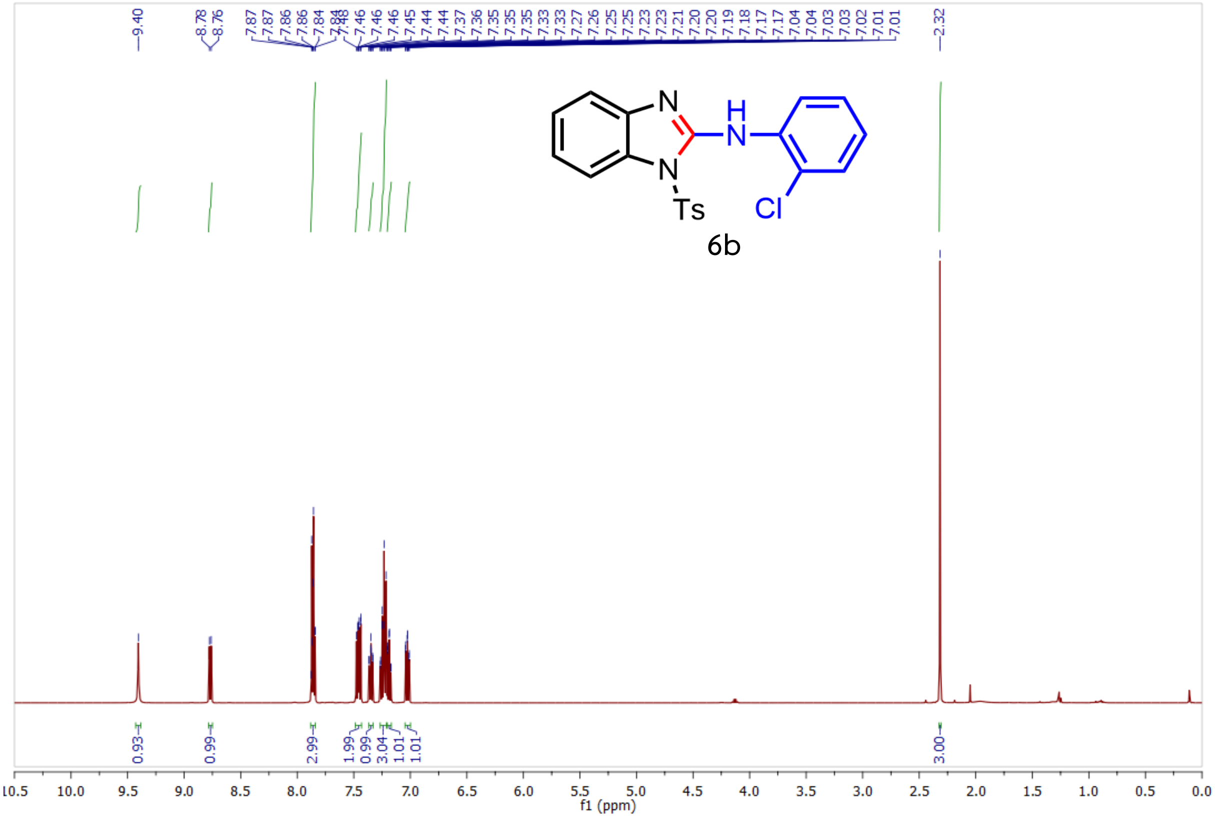

**Figure S36** $^{1}$H-NMR spectrum of **6b** ($CDCl_3$, 500 MHz)

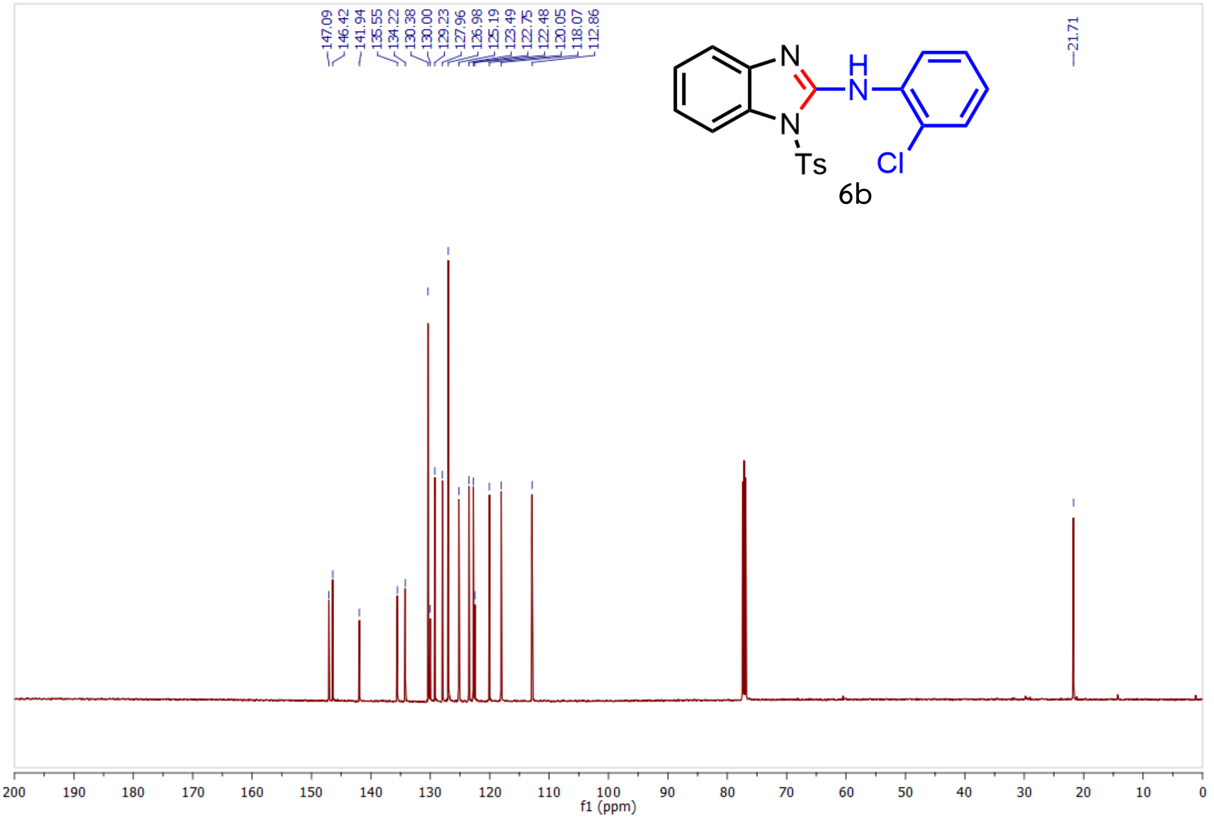

**Figure S37** $^{13}$C-NMR spectrum of **6b** ($CDCl_3$, 126 MHz)

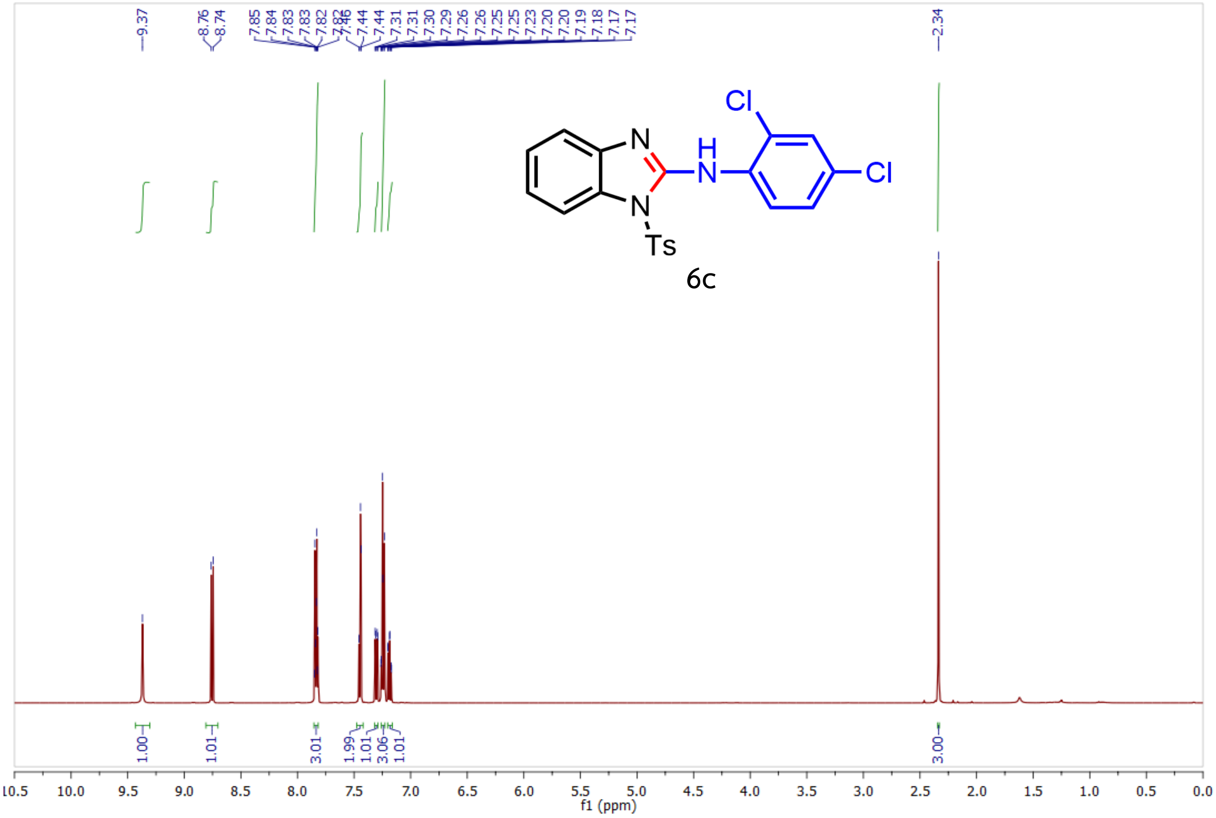


**Figure S38** $^{1}$H-NMR spectrum of **6c** ($CDCl_3$, 500 MHz)

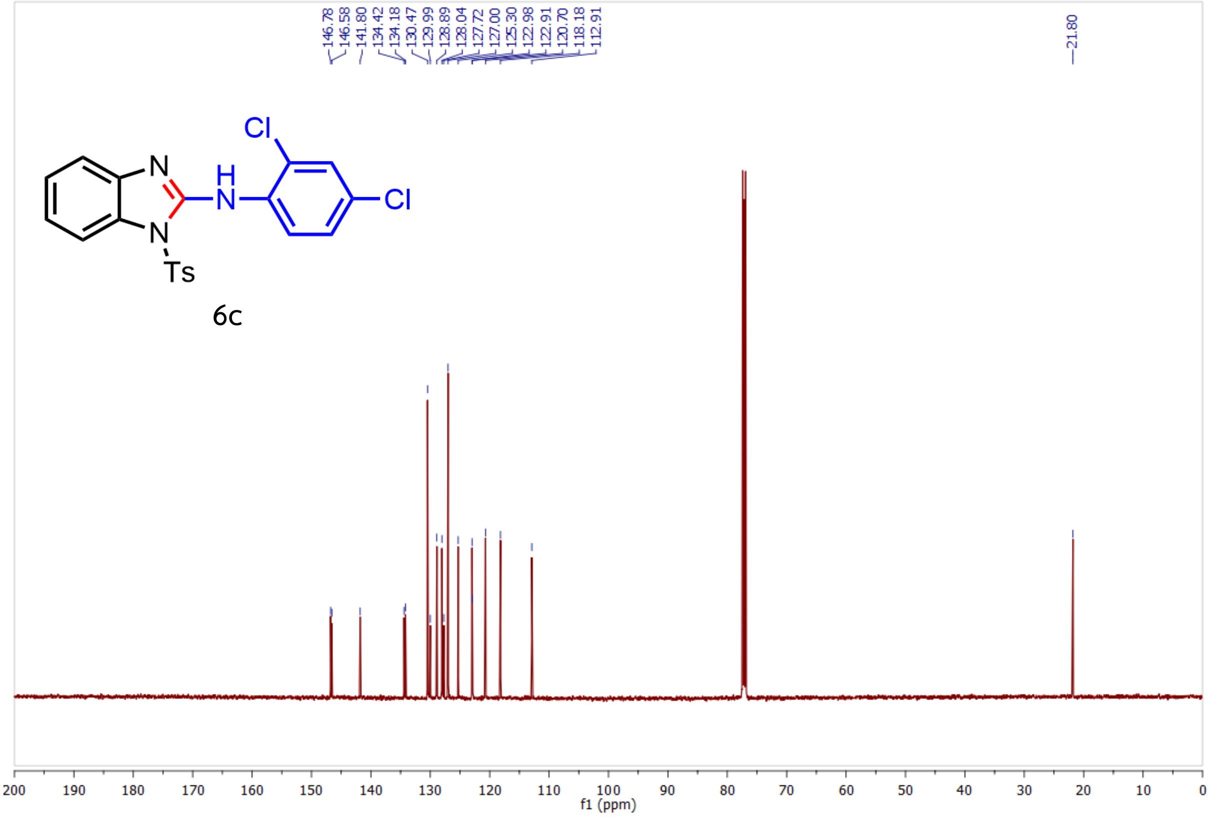


**Figure S39** $^{13}$C-NMR spectrum of **6c** ($CDCl_3$, 126 MHz)

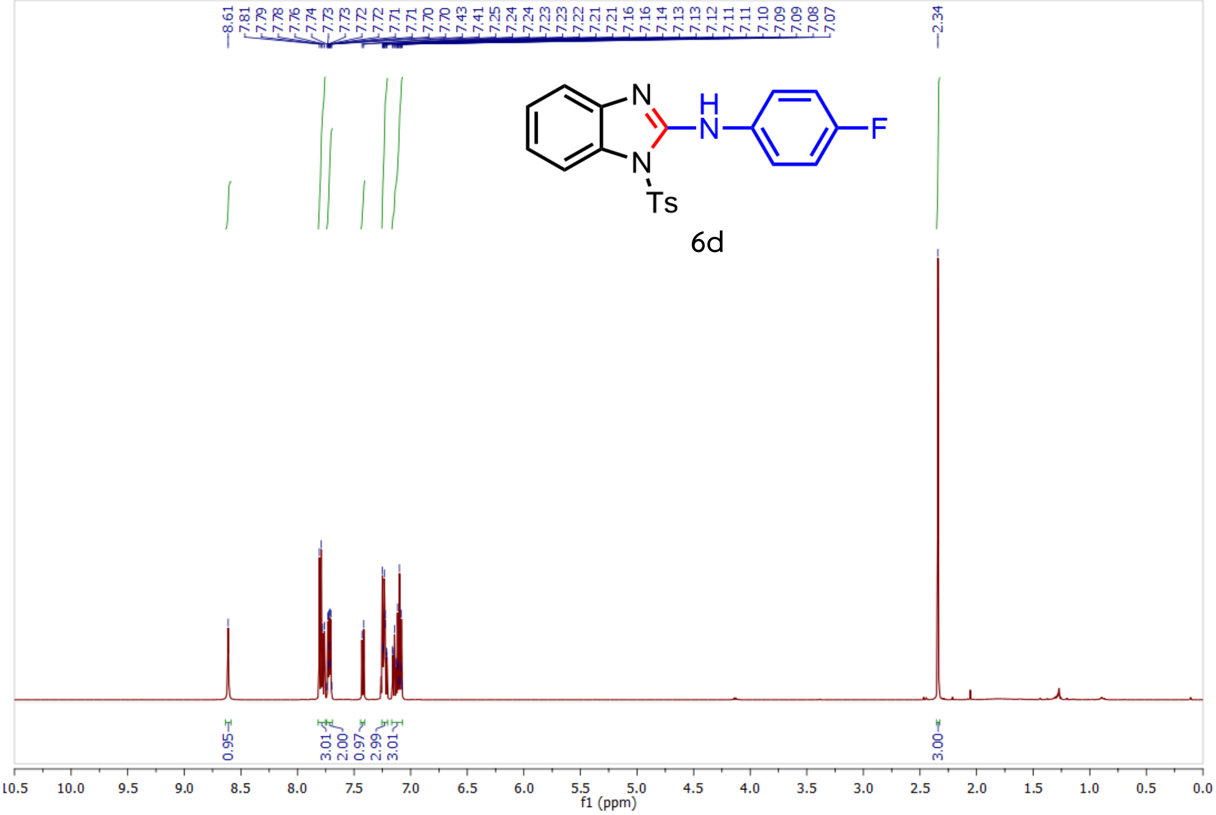

**Figure S40** $^{1}$H-NMR spectrum of **6d** ($CDCl_3$, 500 MHz)

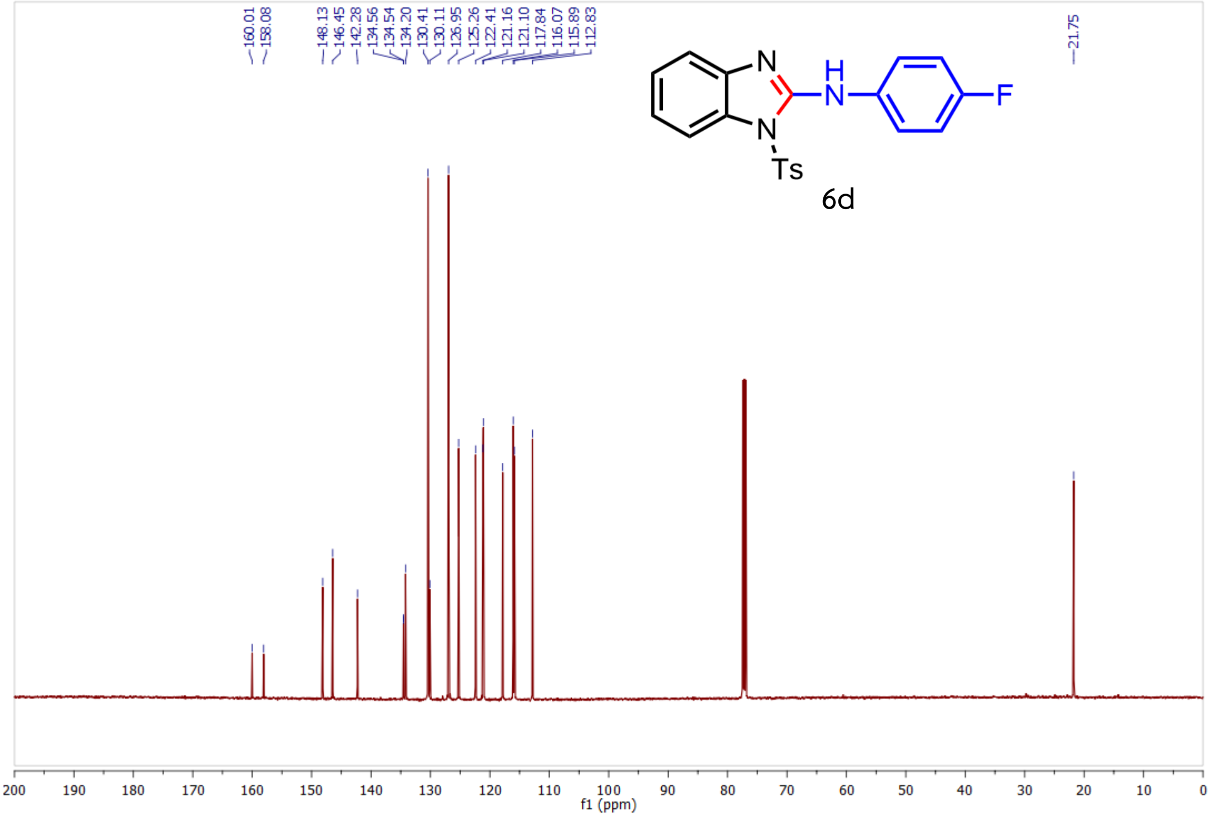

**Figure S41** $^{13}$C-NMR spectrum of **6d** ($CDCl_3$, 126 MHz)

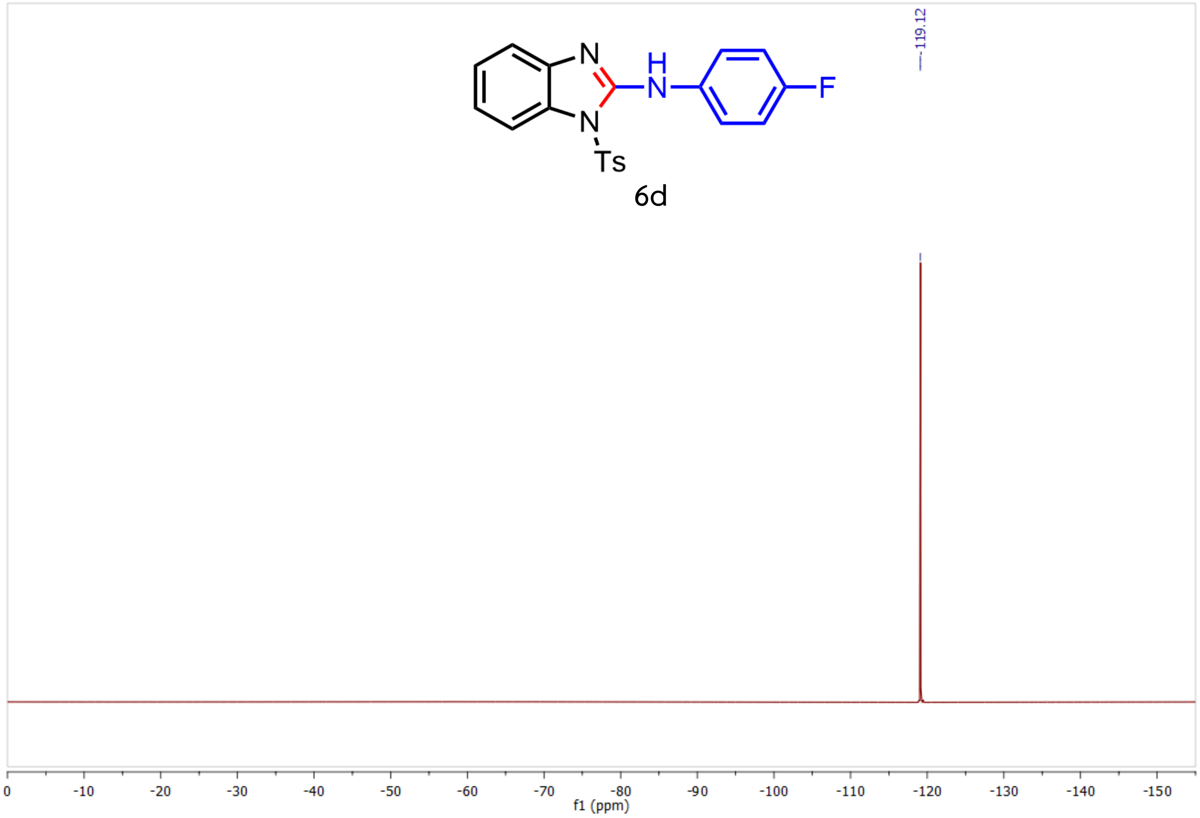


**Figure S42** $^{19}$F-NMR spectrum of **6d** ($CDCl_3$, 471 MHz)

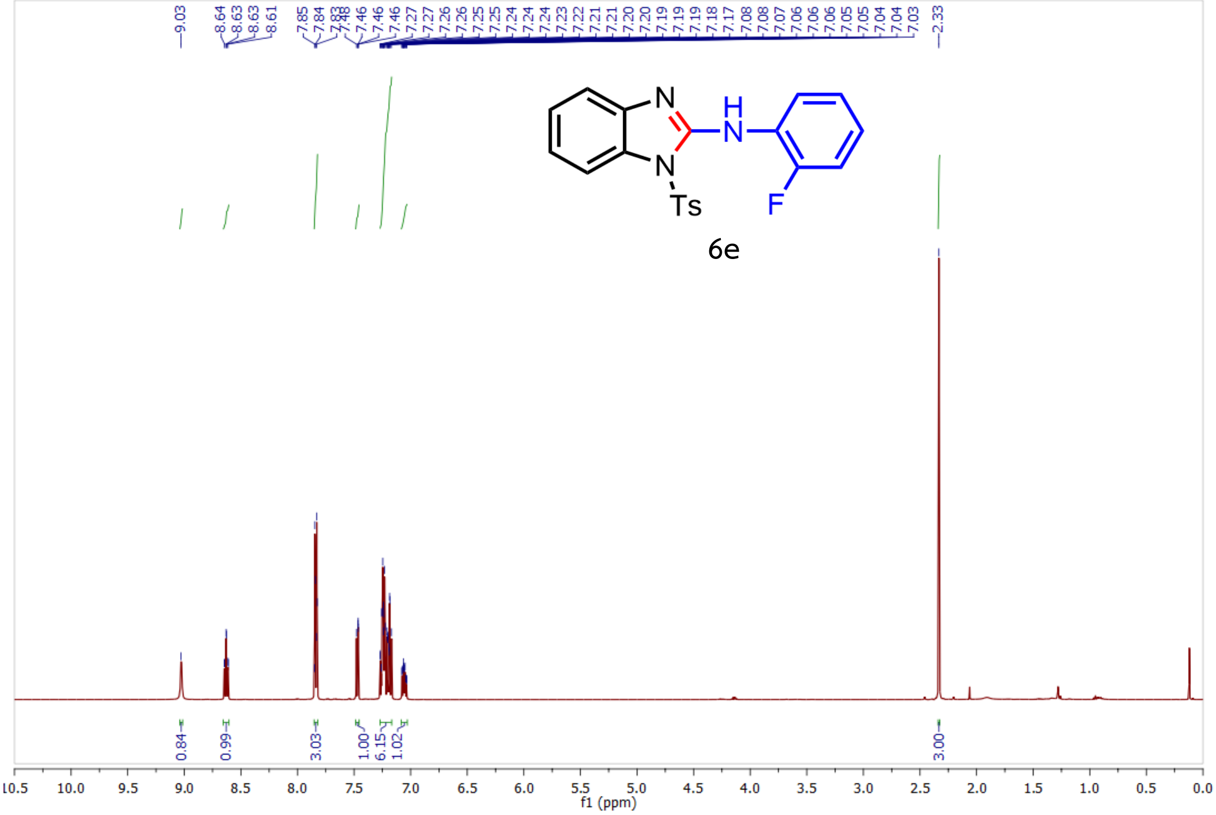


**Figure S43** $^{1}$H-NMR spectrum of **6e** ($CDCl_3$, 500 MHz)

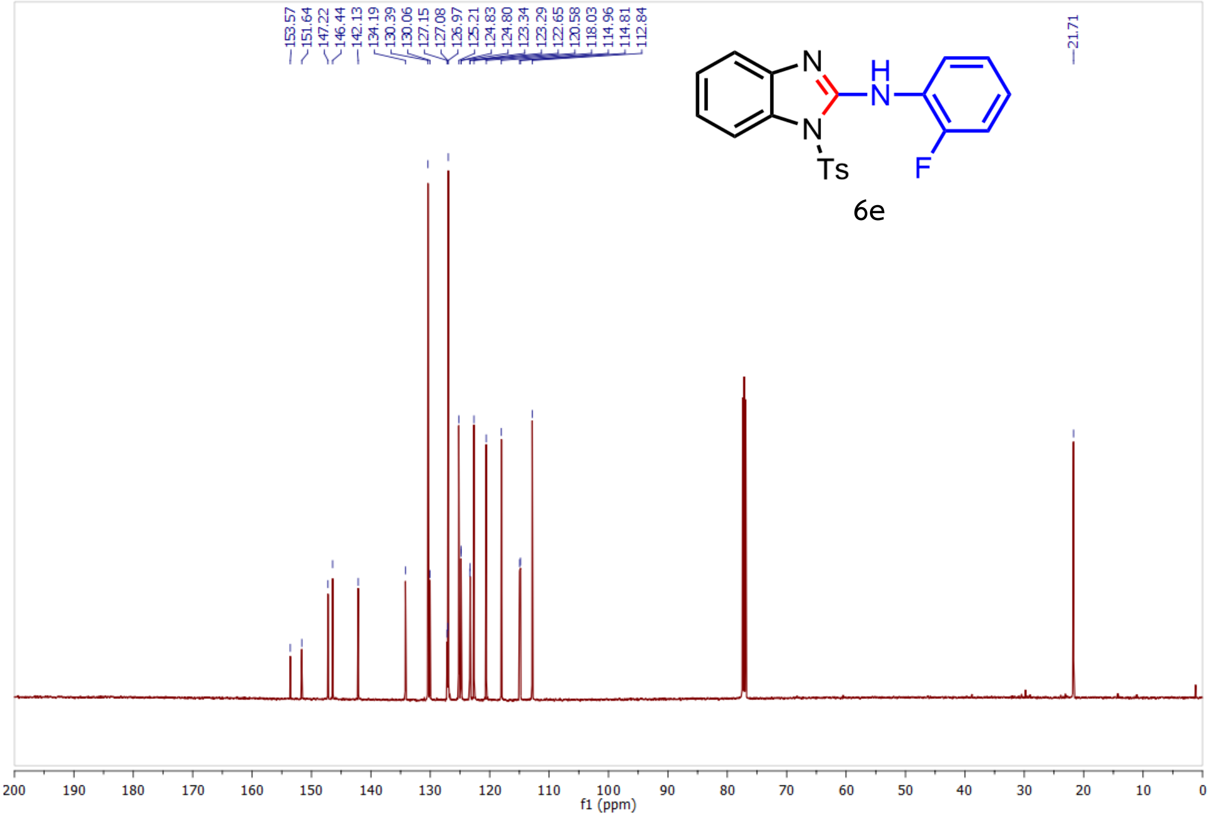


**Figure S44** $^{13}$C-NMR spectrum of **6e** ($CDCl_3$, 126 MHz)

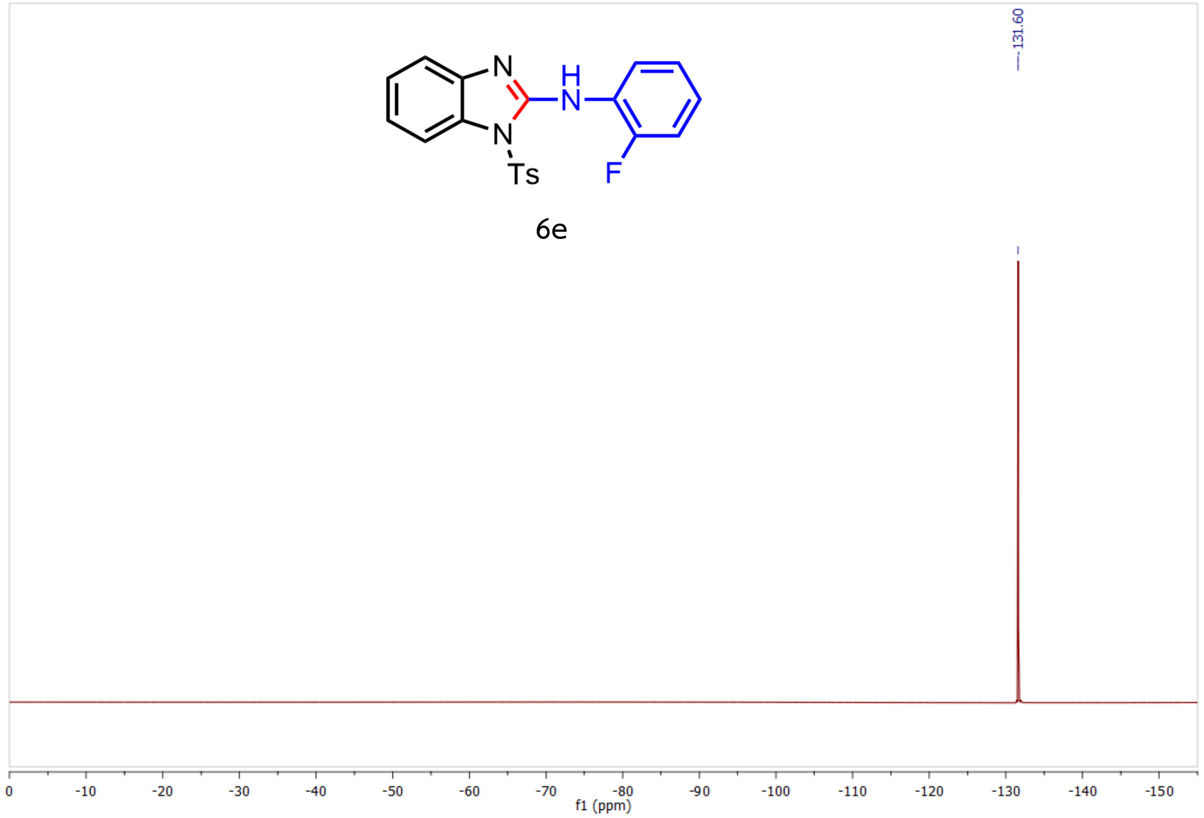


**Figure S45** $^{19}$F-NMR spectrum of **6e** ($CDCl_3$, 471 MHz)

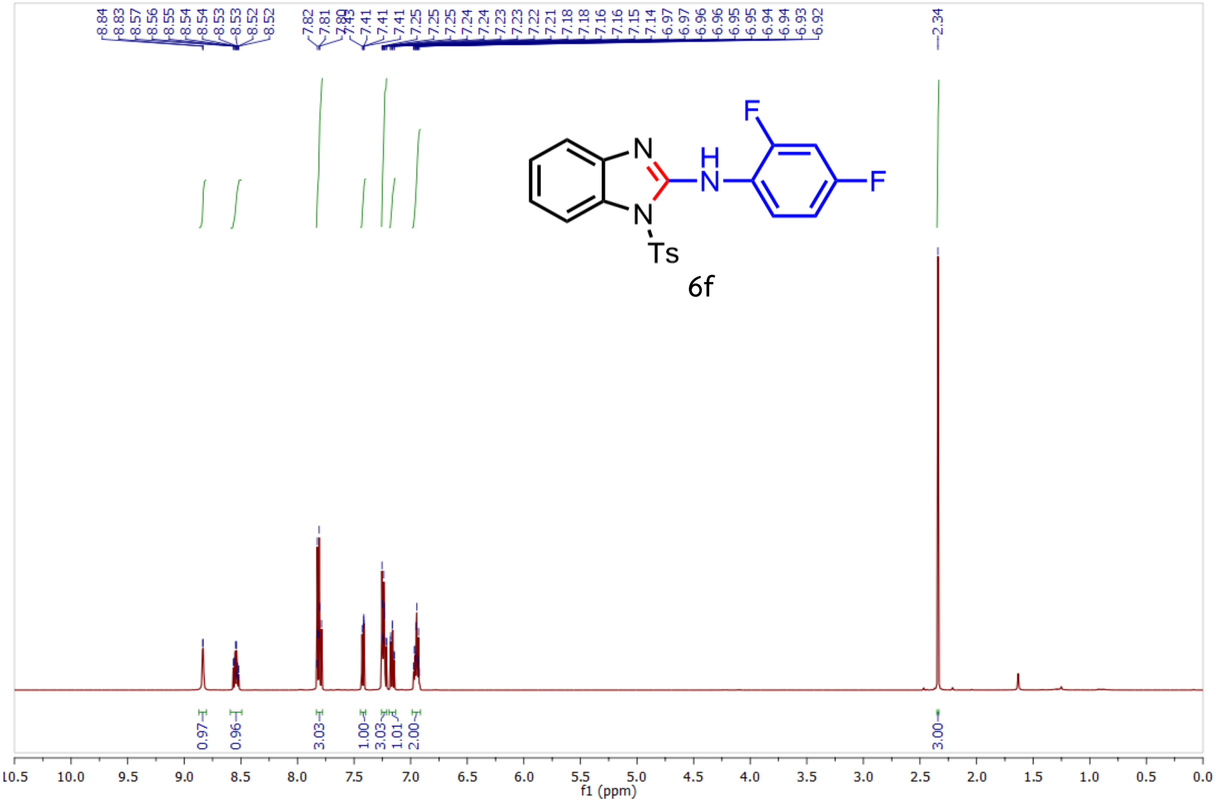


**Figure S46** $^{1}$H-NMR spectrum of **6f** ($CDCl_3$, 500 MHz)

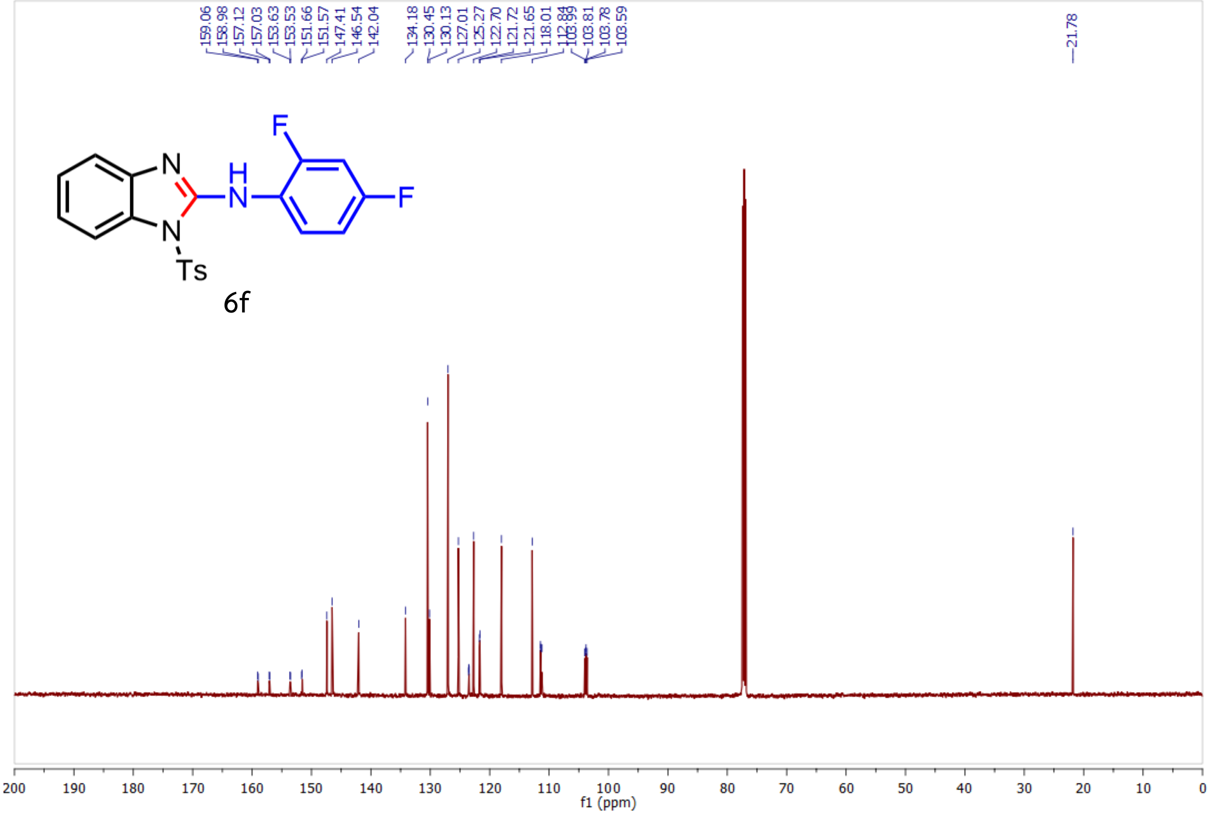


**Figure S47** $^{13}$C-NMR spectrum of **6f** ($CDCl_3$, 126 MHz)

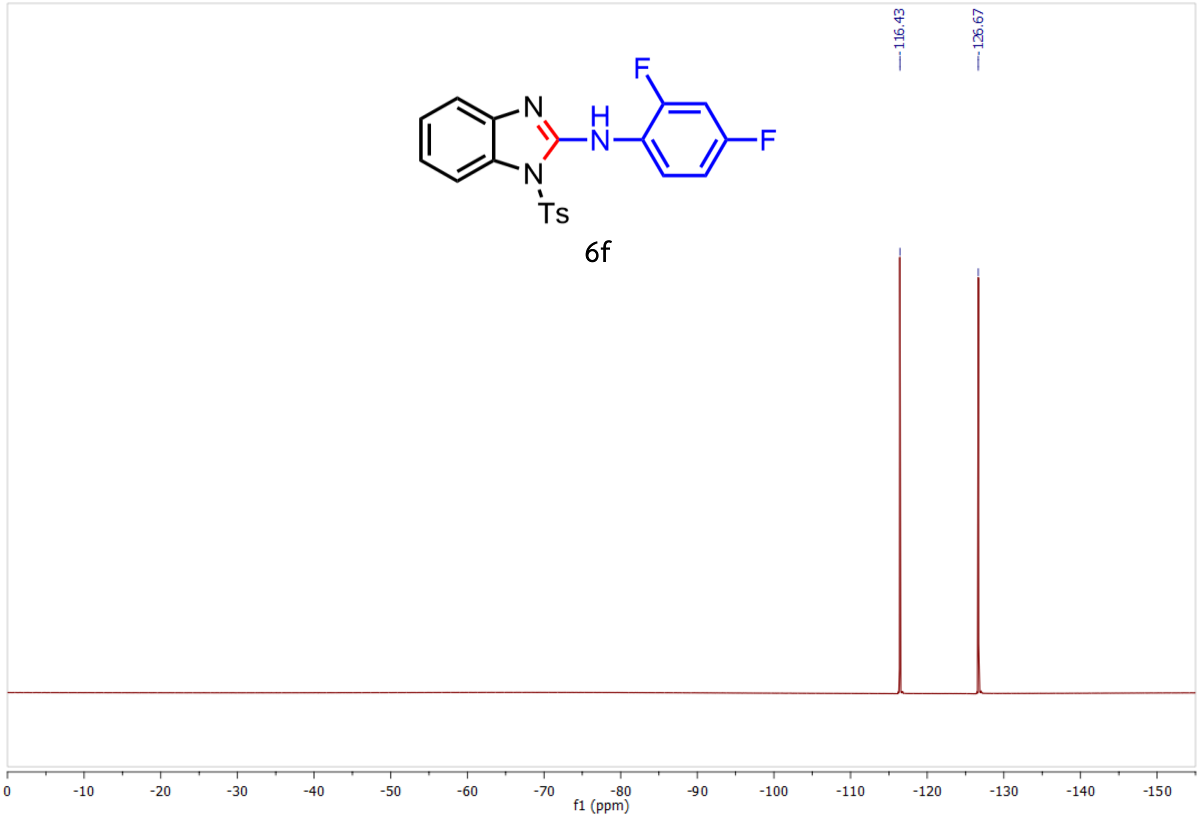


**Figure S48** $^{19}$F-NMR spectrum of **6f** ($CDCl_3$, 471 MHz)

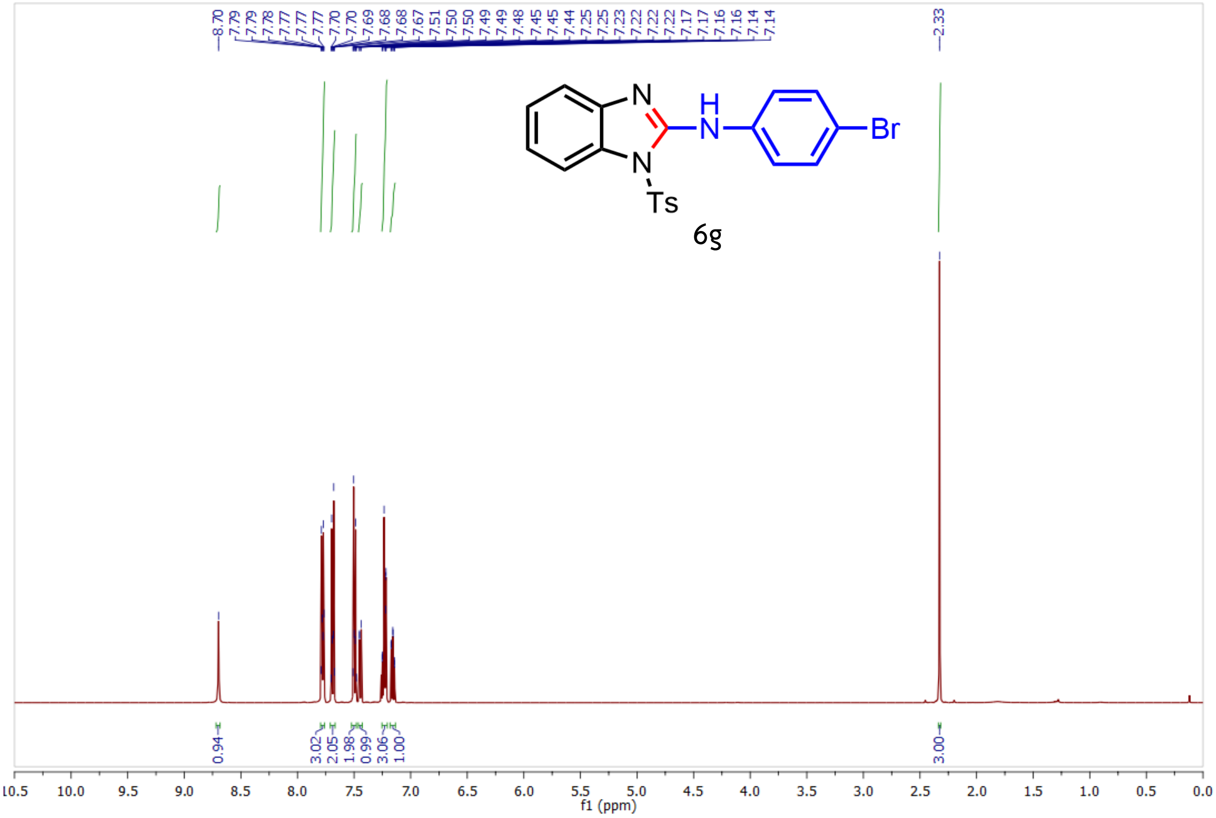


**Figure S49** $^{1}$H-NMR spectrum of **6g** ($CDCl_3$, 500 MHz)

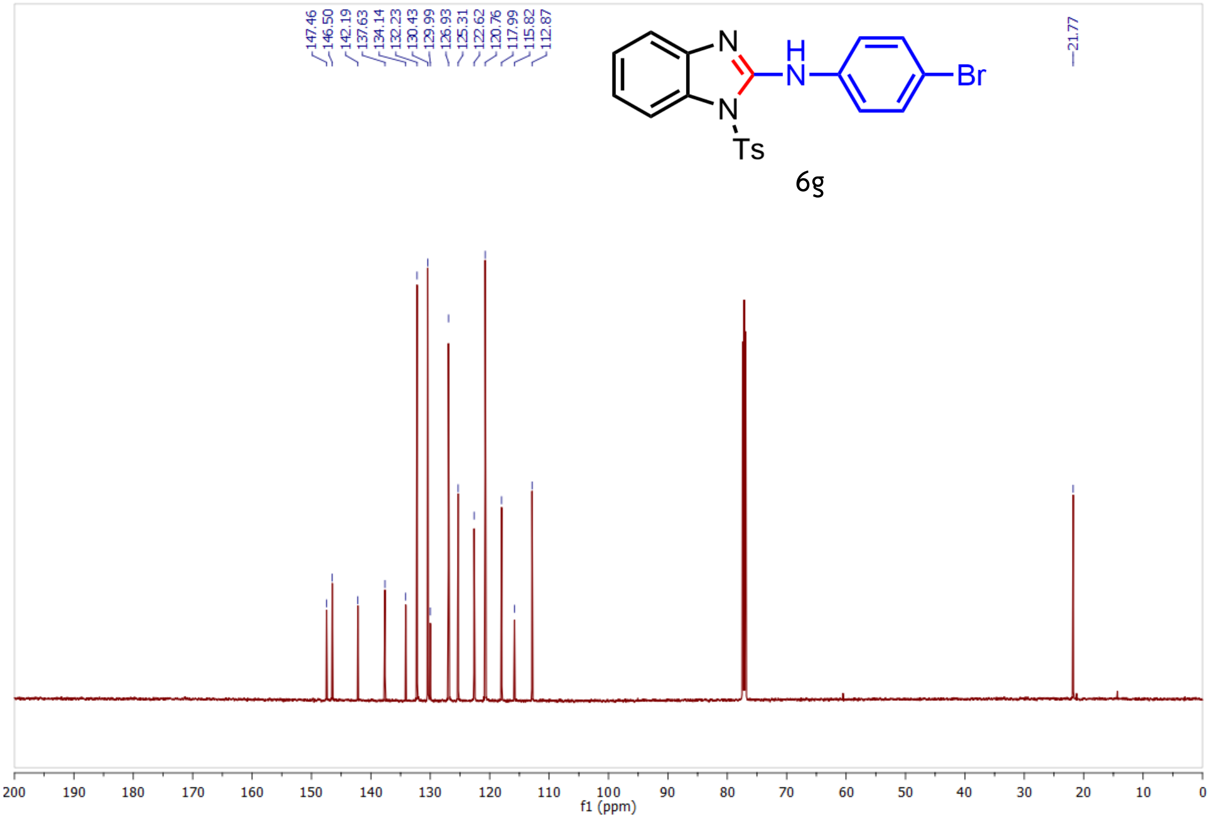


**Figure S50** $^{13}$C-NMR spectrum of **6g** ($CDCl_3$, 126 MHz)

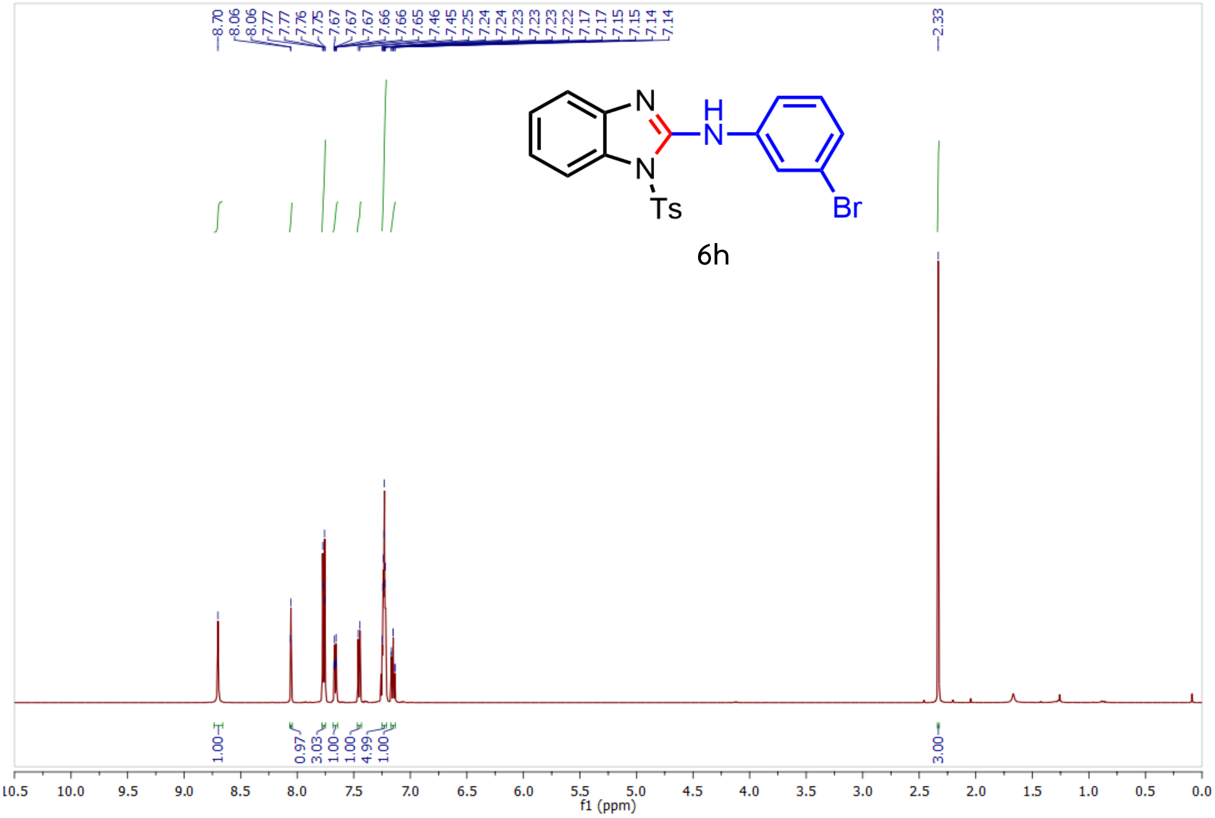

**Figure S51** $^{1}$H-NMR spectrum of **6h** ($CDCl_3$, 500 MHz)

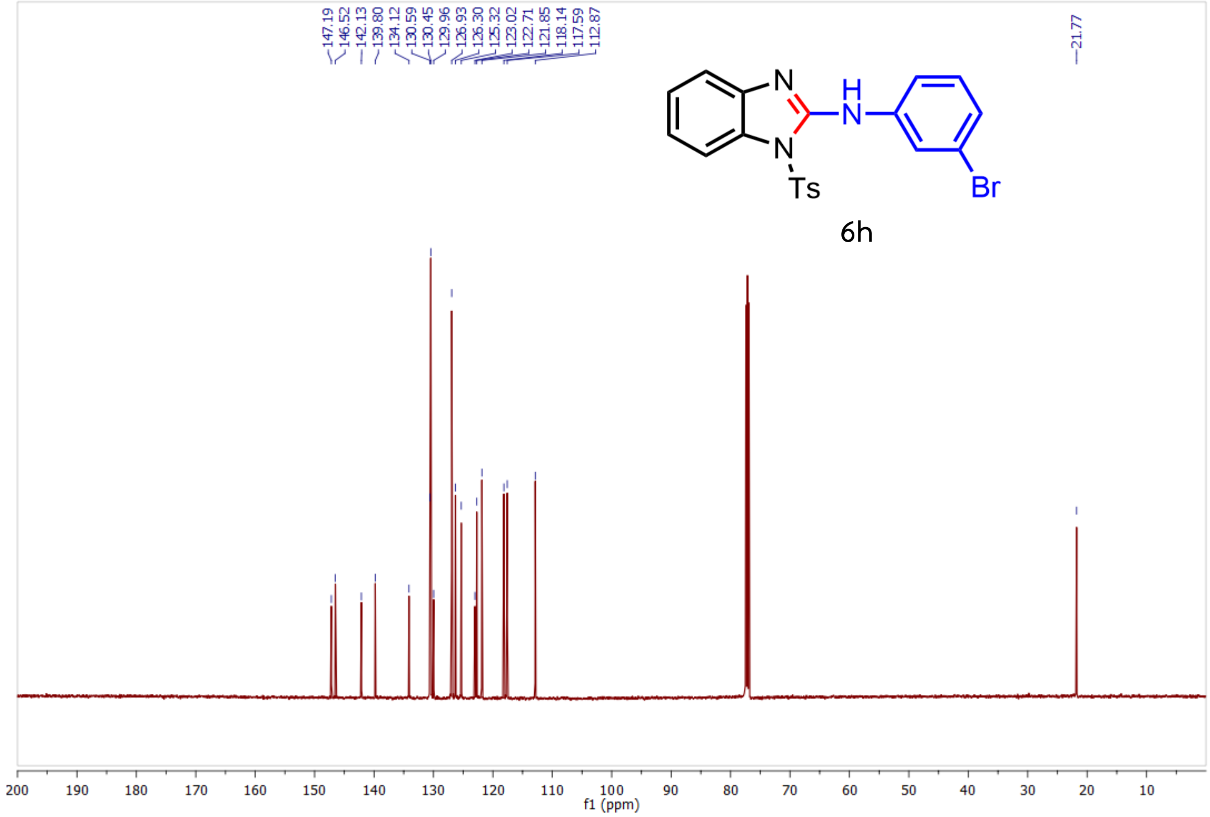

**Figure S52** $^{13}$C-NMR spectrum of **6h** ($CDCl_3$, 126 MHz)

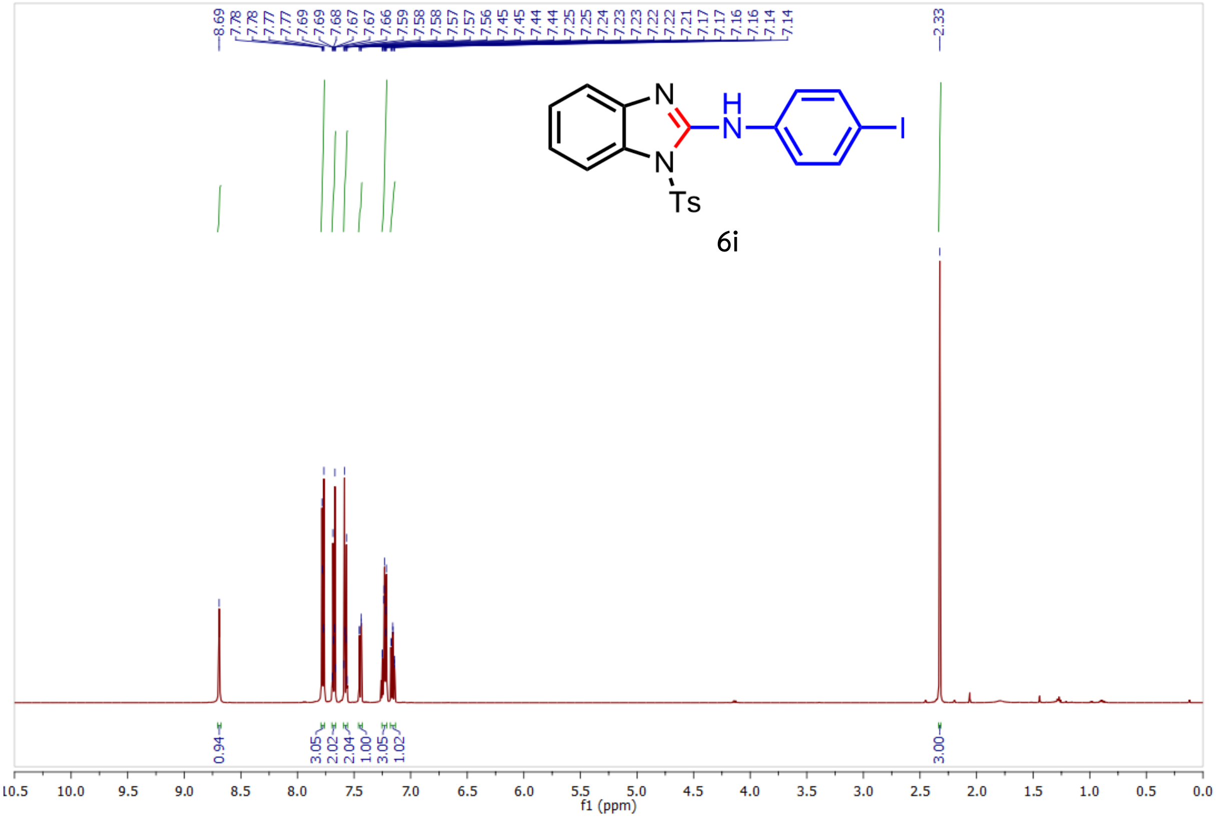


**Figure S53** $^{1}$H-NMR spectrum of **6i** ($CDCl_3$, 500 MHz)

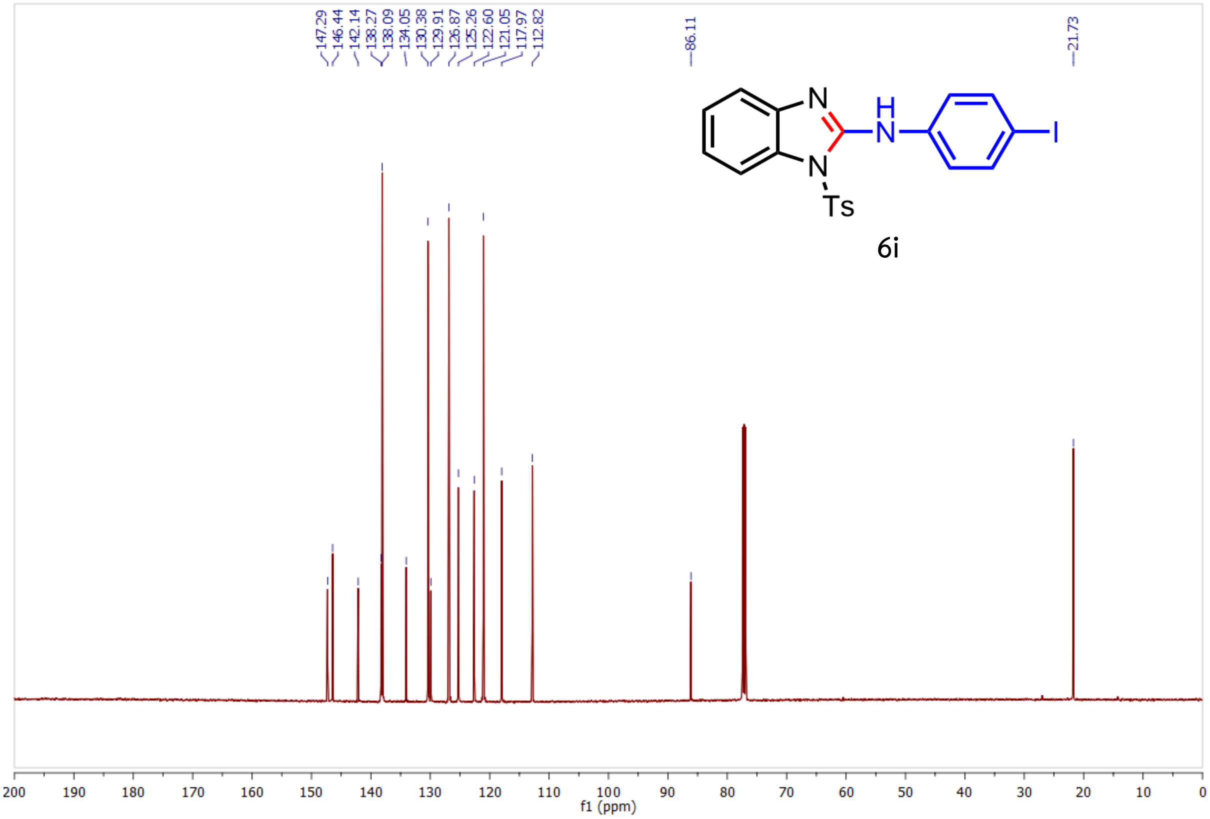


**Figure S54** $^{13}$C-NMR spectrum of **6i** ($CDCl_3$, 126 MHz)

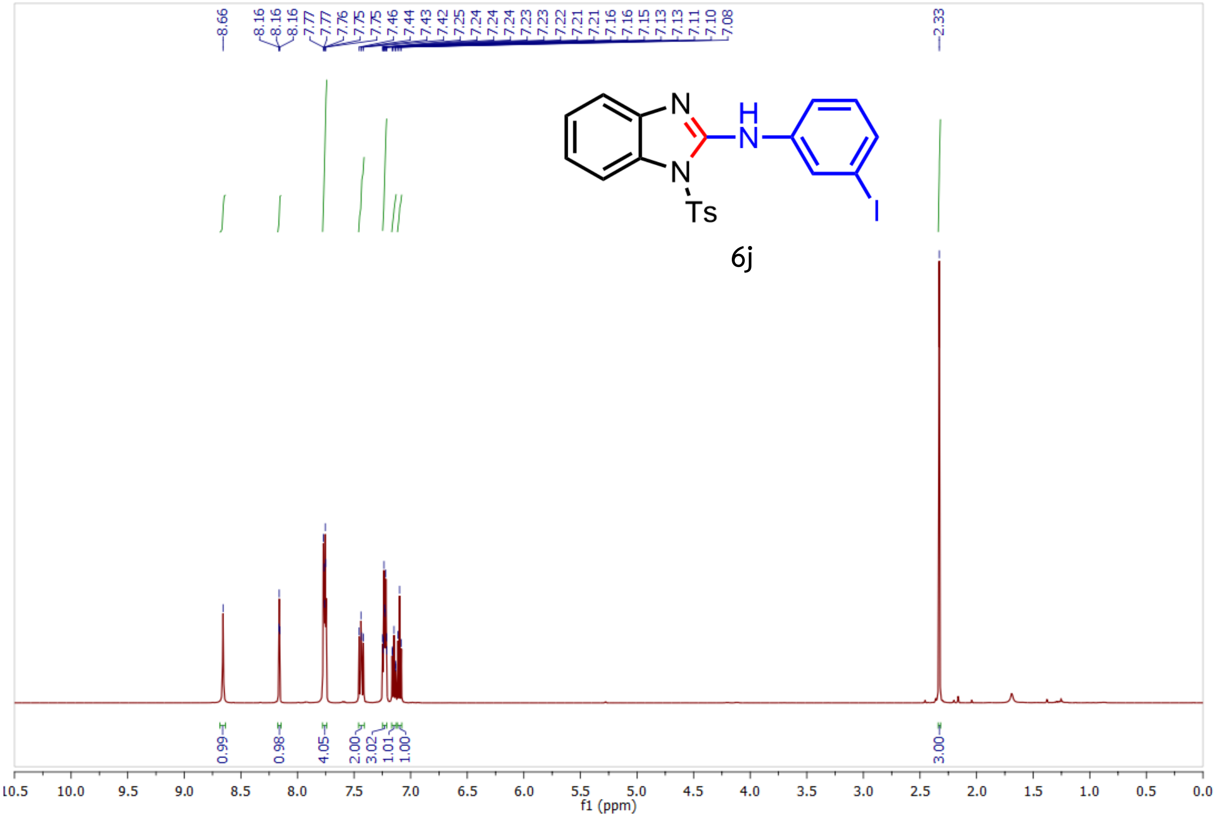


**Figure S55** $^{1}$H-NMR spectrum of **6j** ($CDCl_3$, 500 MHz)

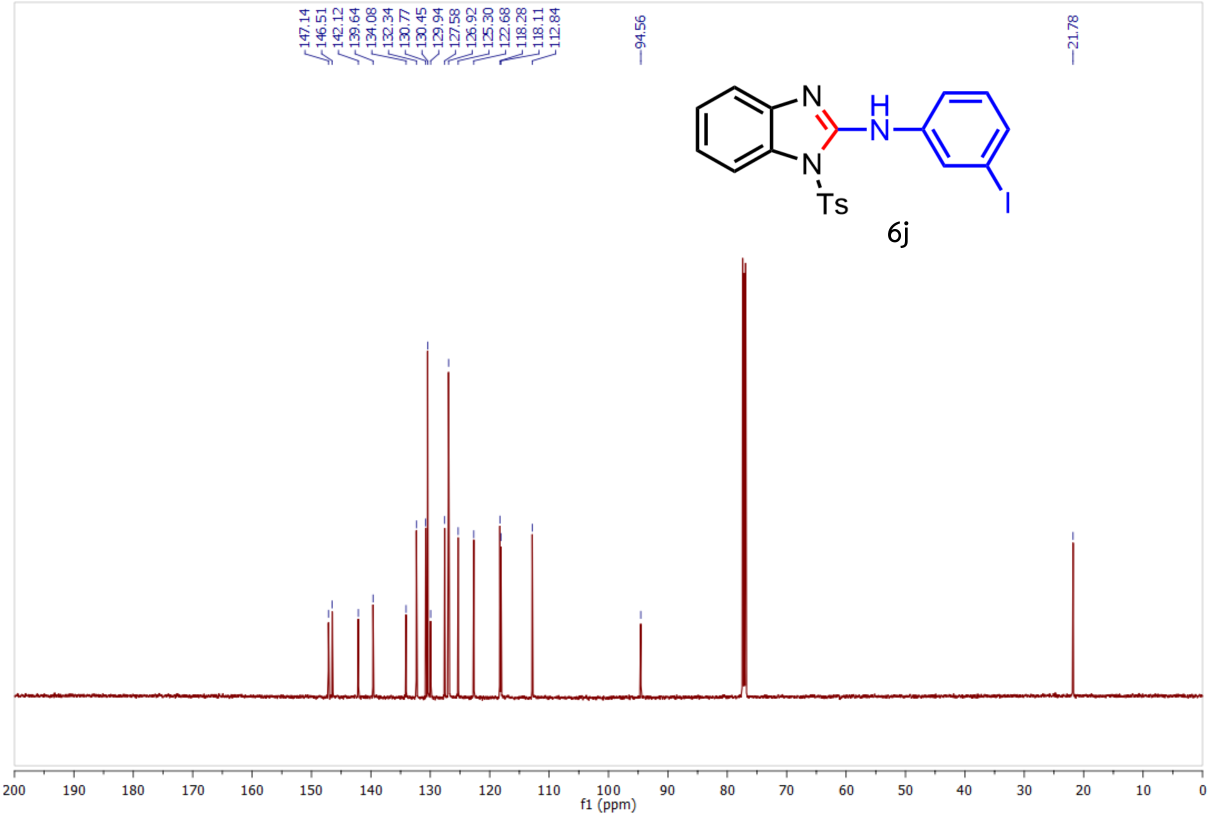


**Figure S56** $^{13}$C-NMR spectrum of **6j** ($CDCl_3$, 126 MHz)

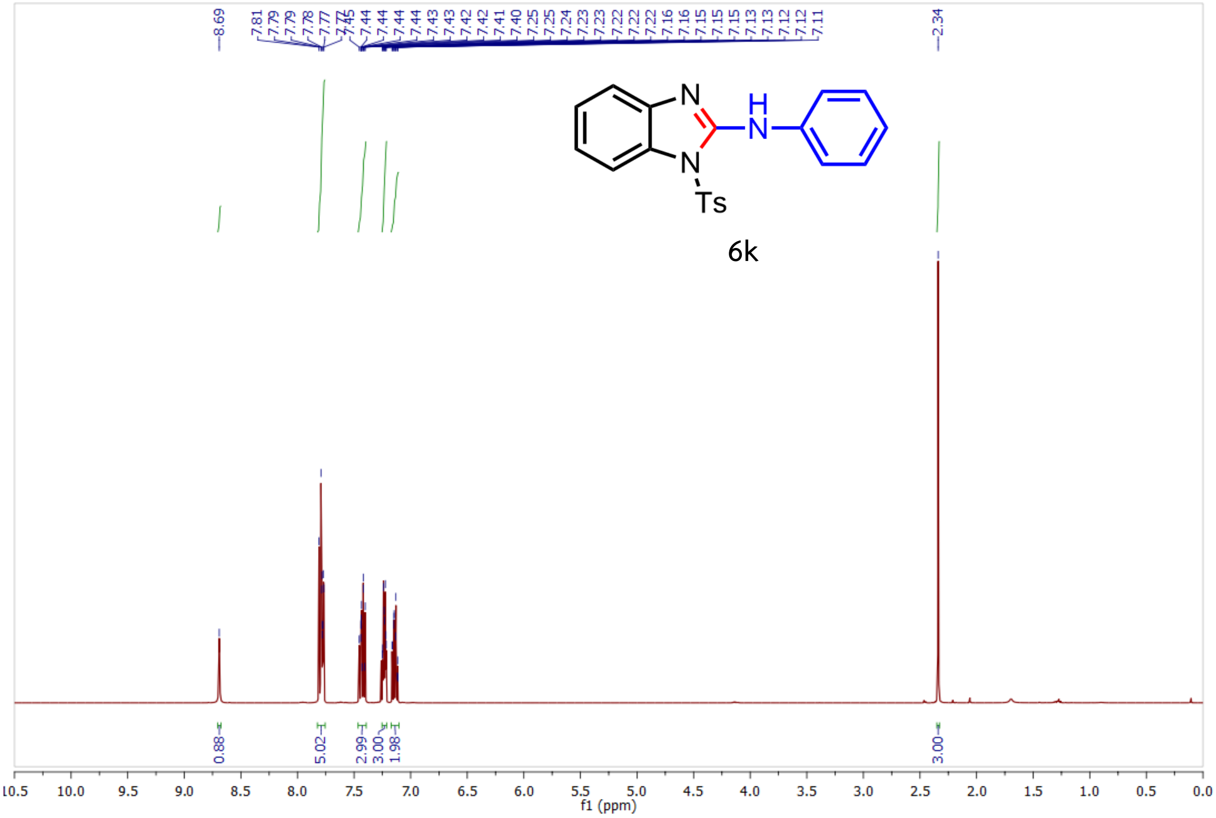


**Figure S57** ¹H-NMR spectrum of **6k** ($CDCl_3$, 500 MHz)

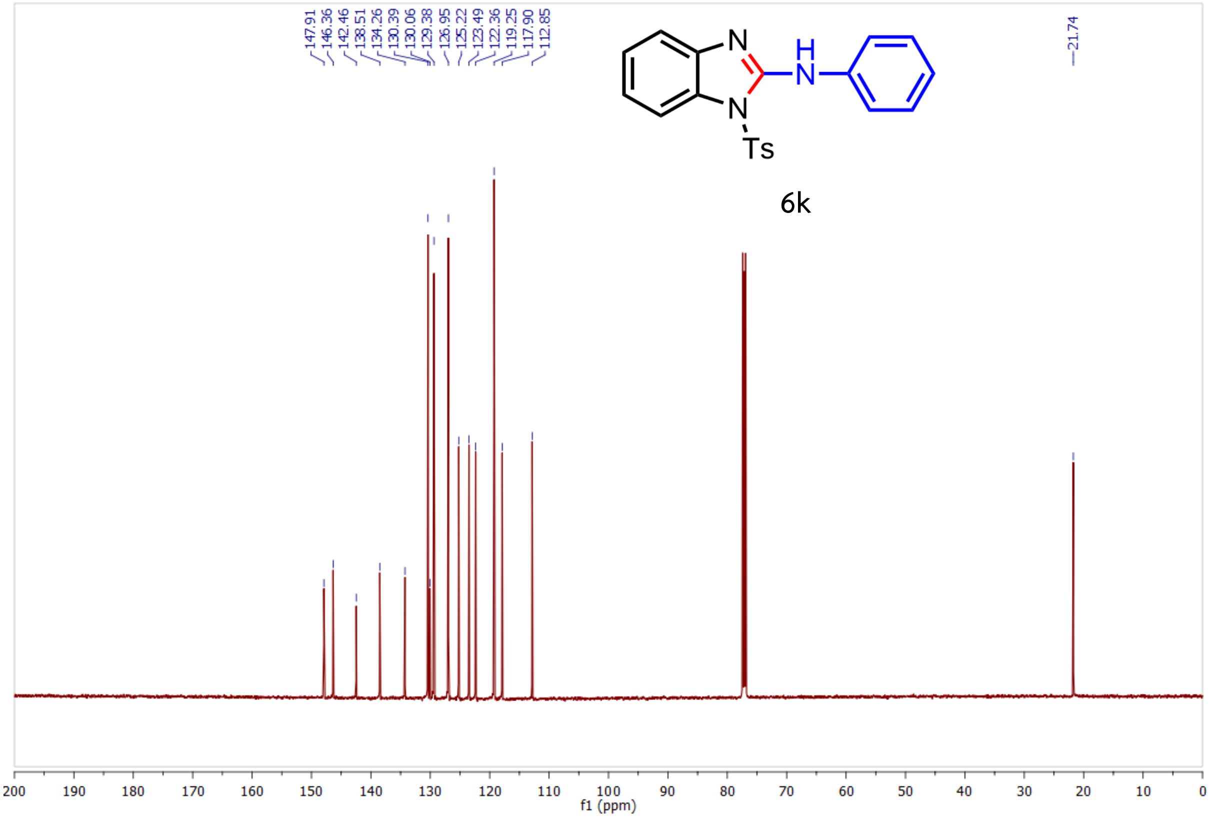


**Figure S58** ¹³C-NMR spectrum of **6k** ($CDCl_3$, 126 MHz)

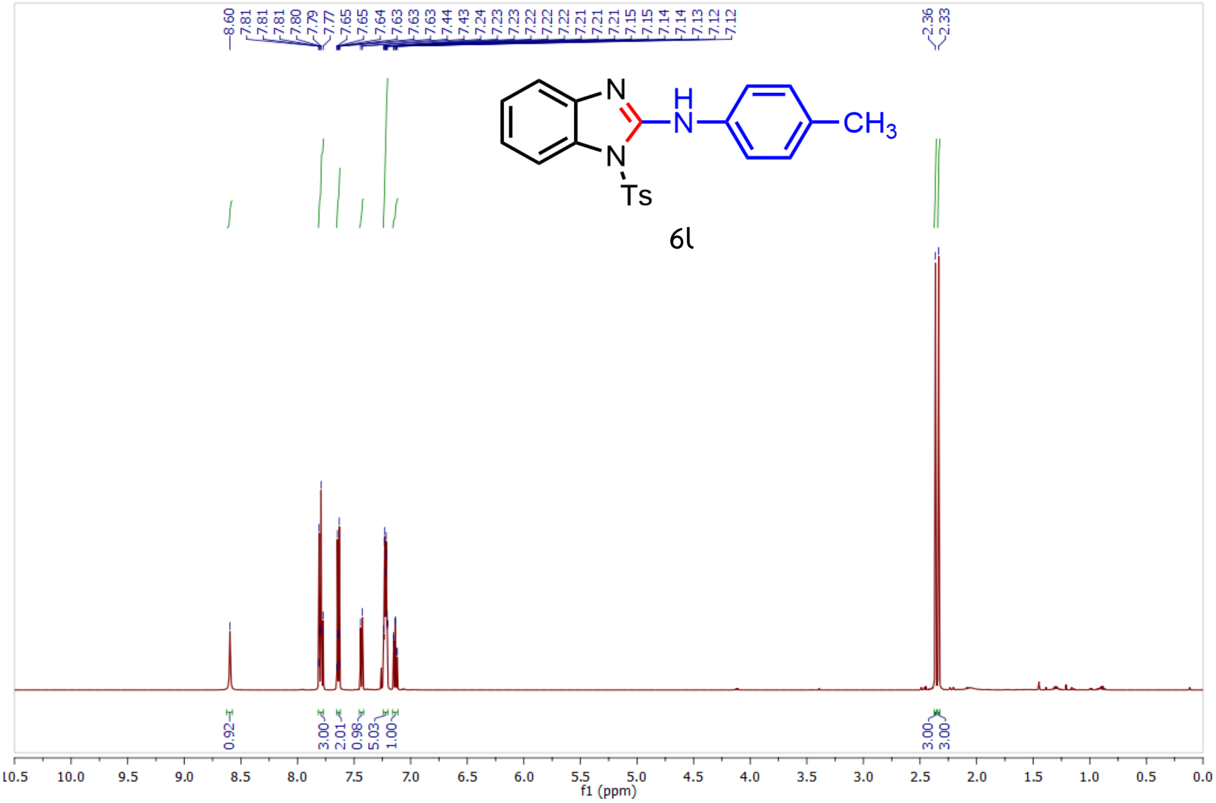


**Figure S59** $^{1}$H-NMR spectrum of **6l** ($CDCl_3$, 500 MHz)

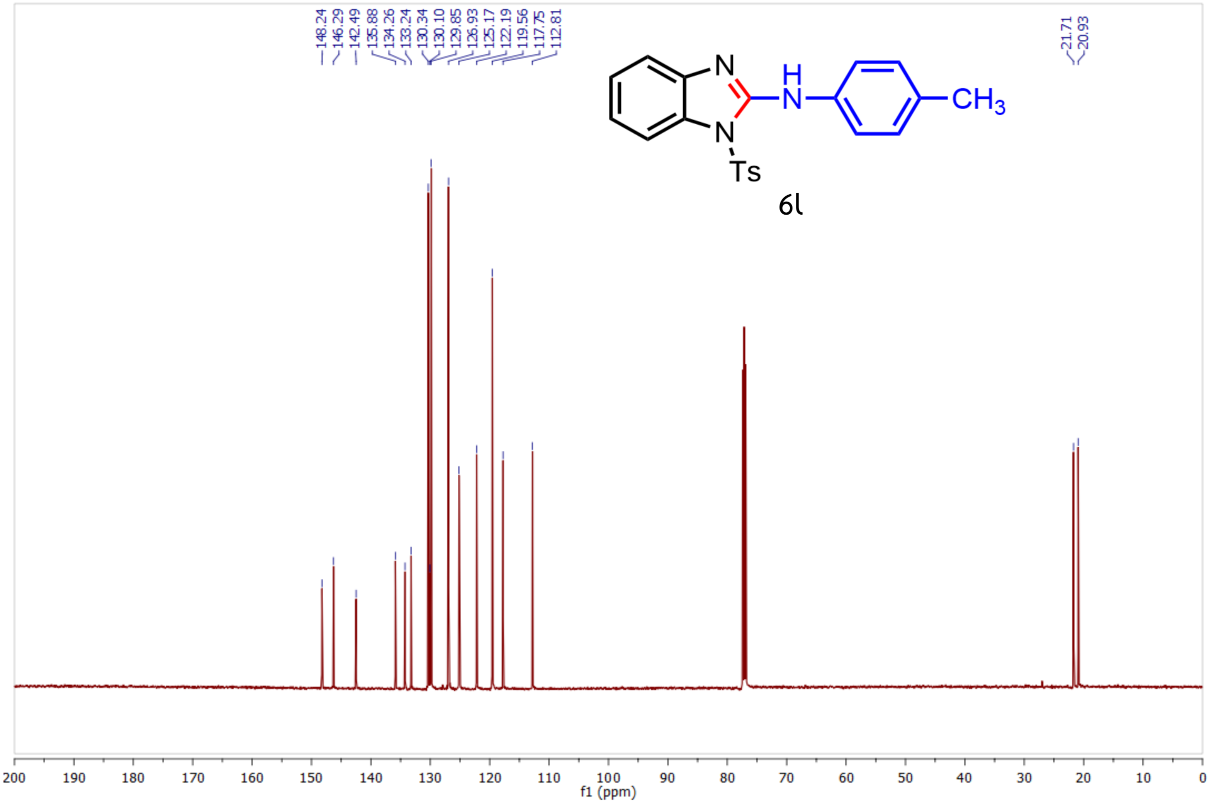


**Figure S60** $^{13}$C-NMR spectrum of **6l** ($CDCl_3$, 126 MHz)

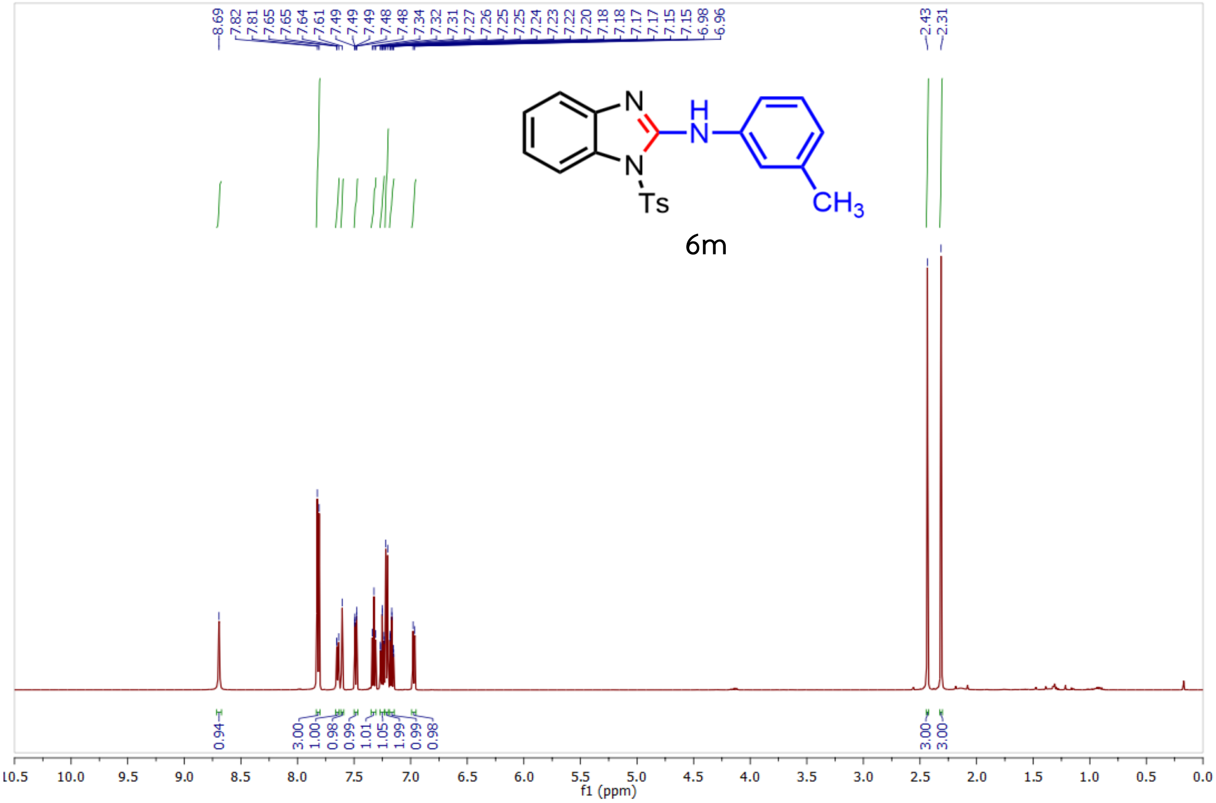


**Figure S61** $^{1}$H-NMR spectrum of **6m** ($CDCl_3$, 500 MHz)

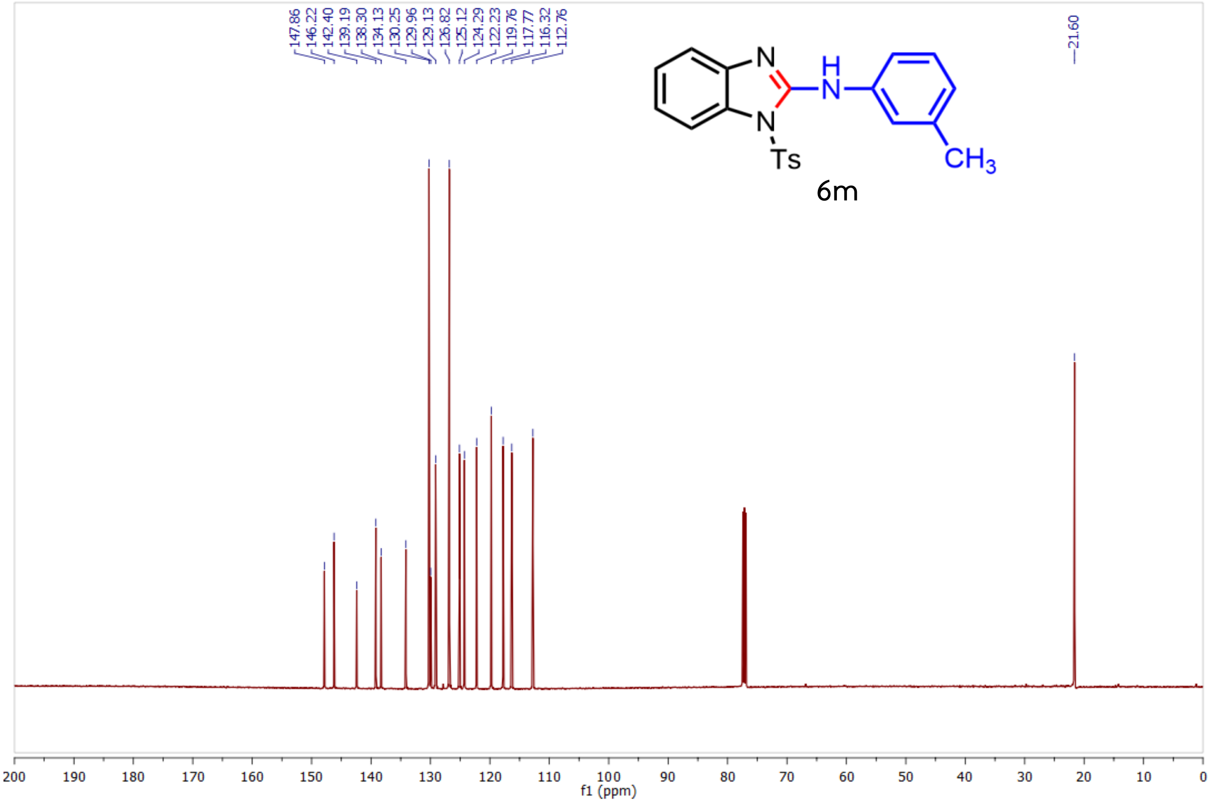


**Figure S62** $^{13}$C-NMR spectrum of **6m** ($CDCl_3$, 126 MHz)

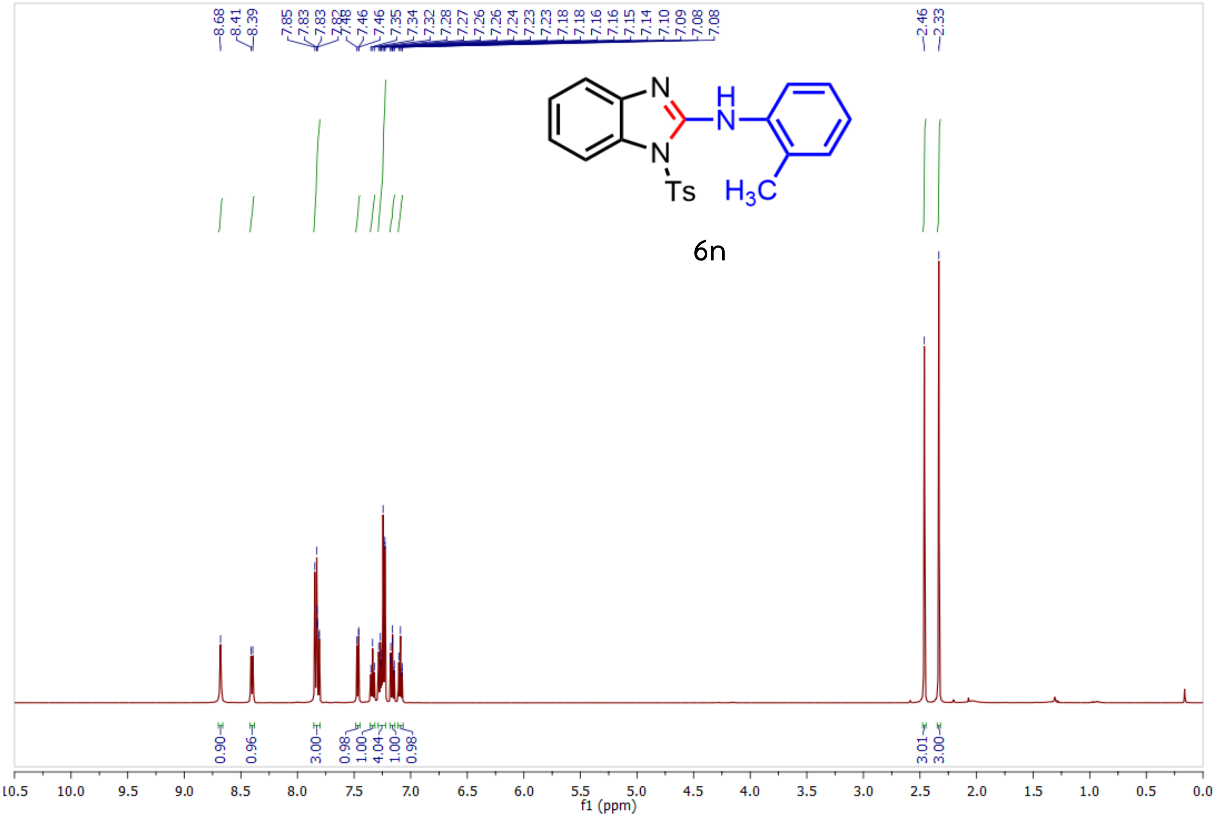


**Figure S63** $^{1}H$-NMR spectrum of **6n** ($CDCl_3$, 500 MHz)

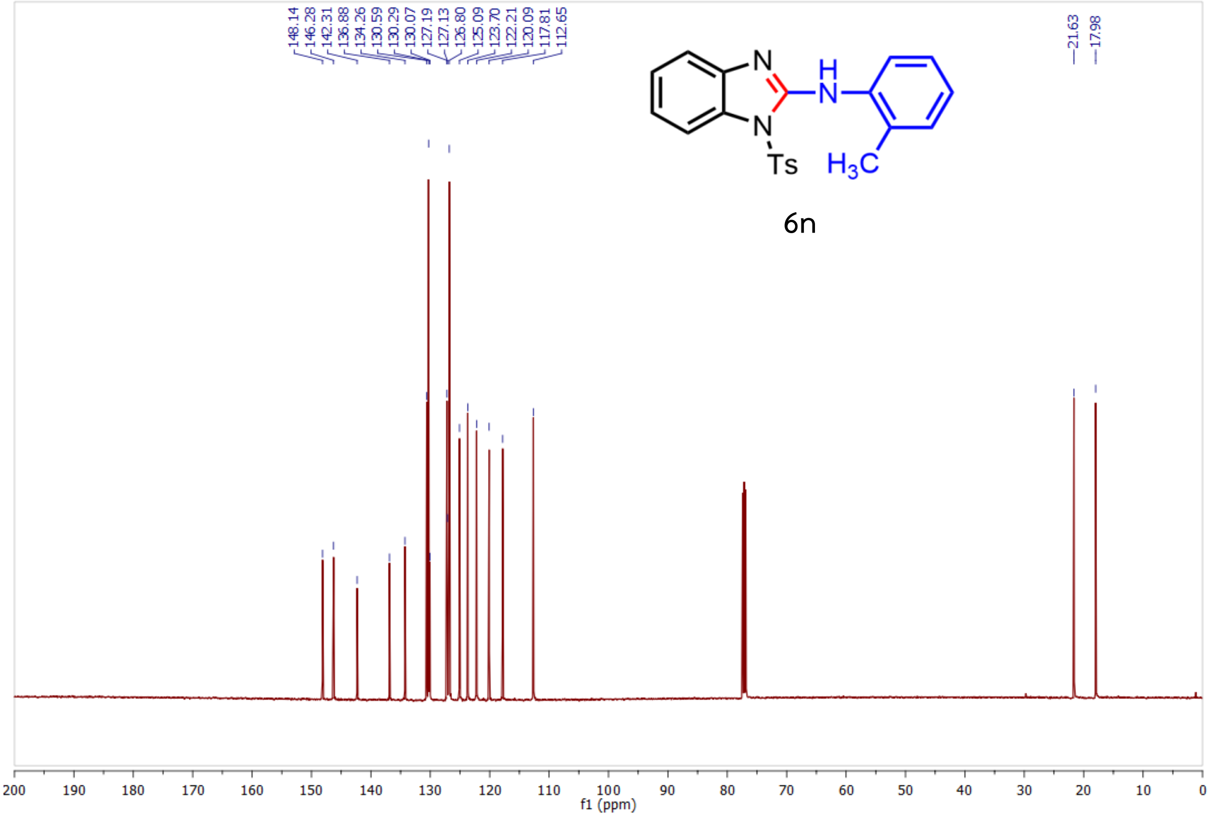


**Figure S64** $^{13}C$-NMR spectrum of **6n** ($CDCl_3$, 126 MHz)

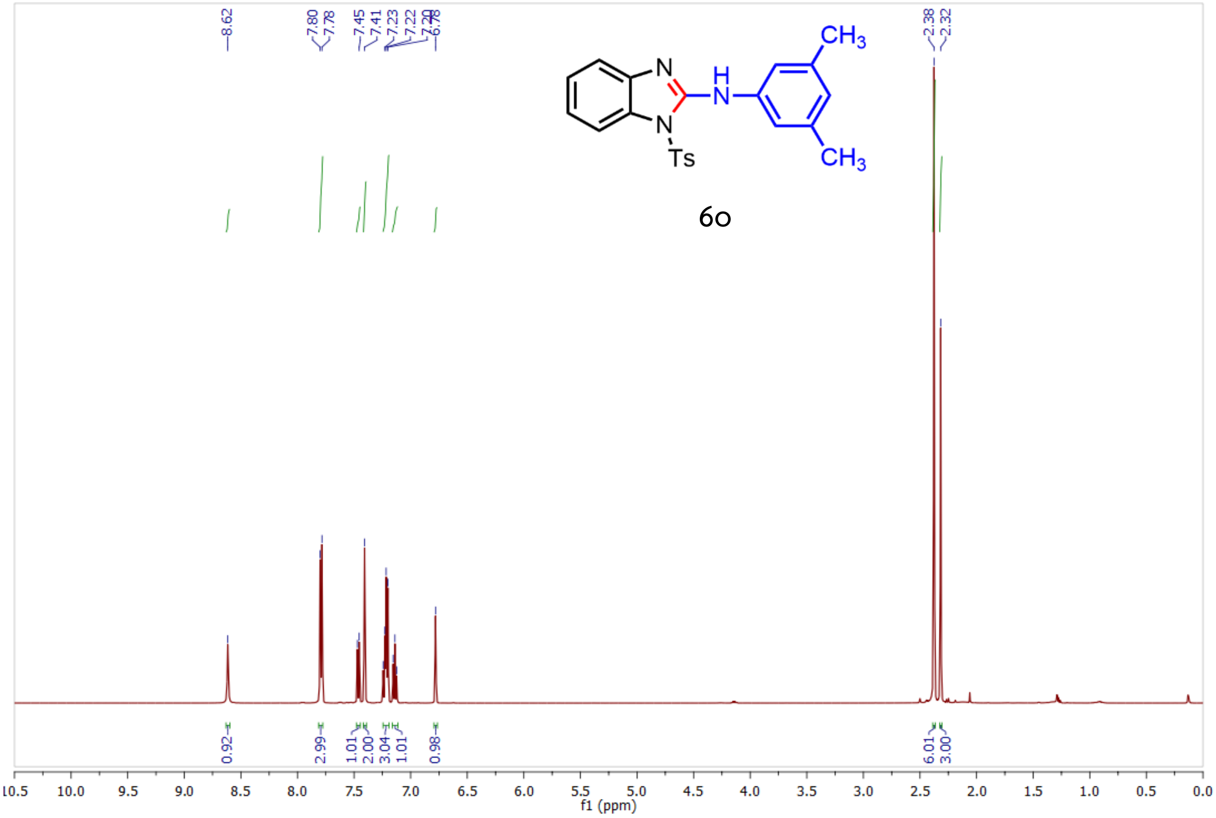


**Figure S65** ¹H-NMR spectrum of **6o** ($CDCl_3$, 500 MHz)

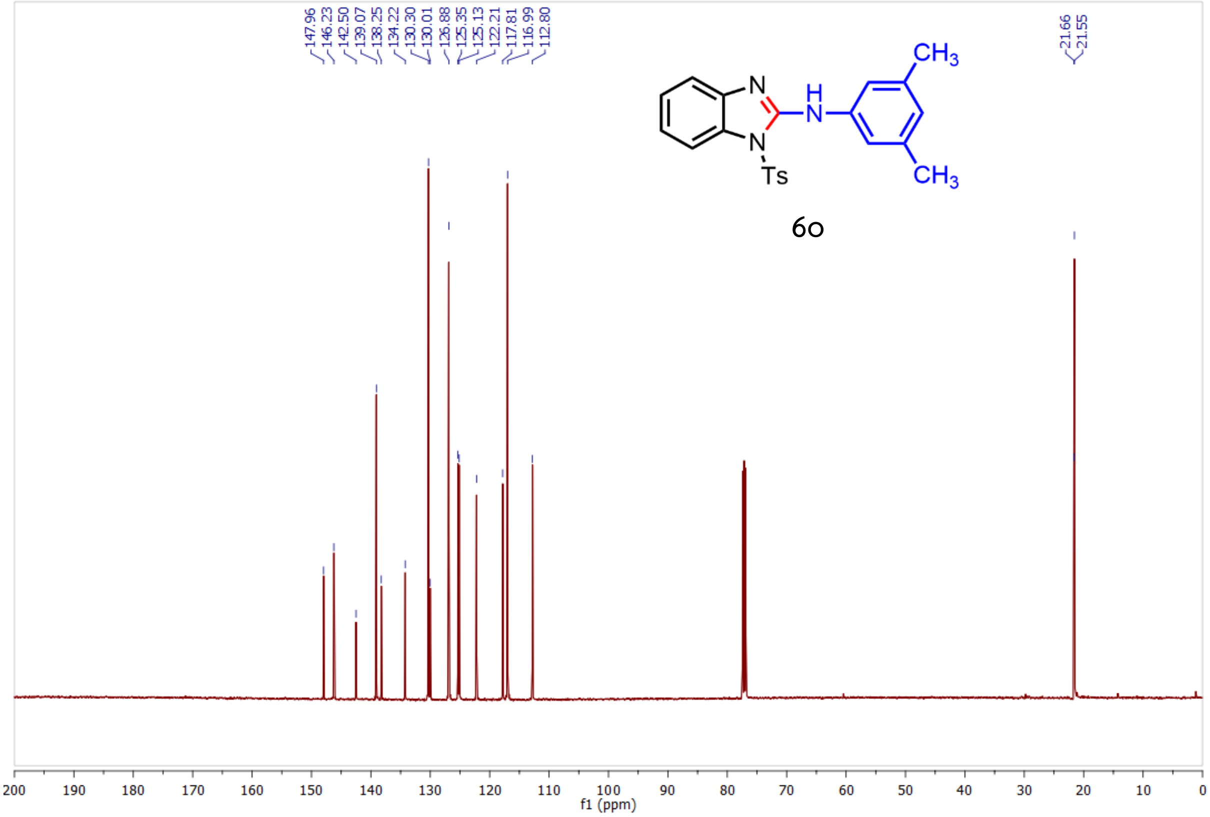


**Figure S66** ¹³C-NMR spectrum of **6o** ($CDCl_3$, 126 MHz)

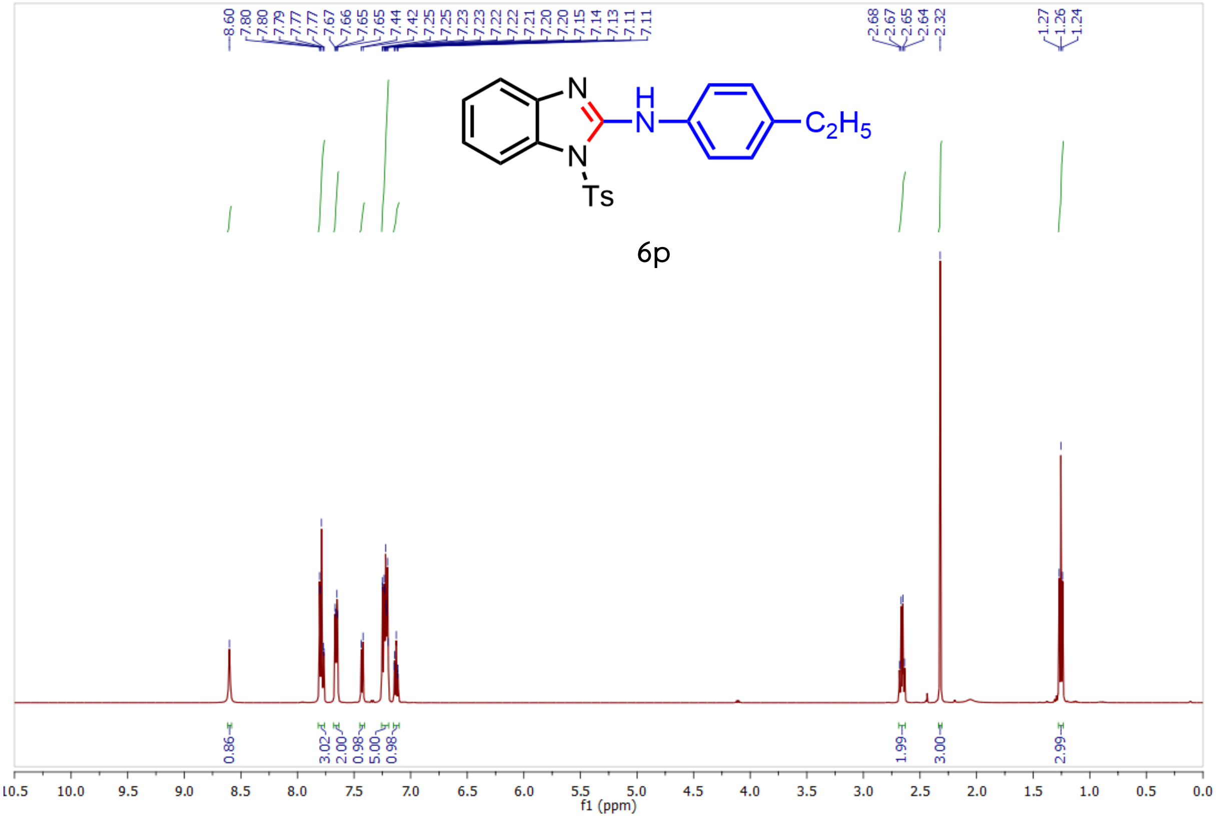


**Figure S67** $^{1}$H-NMR spectrum of **6p** ($CDCl_3$, 500 MHz)

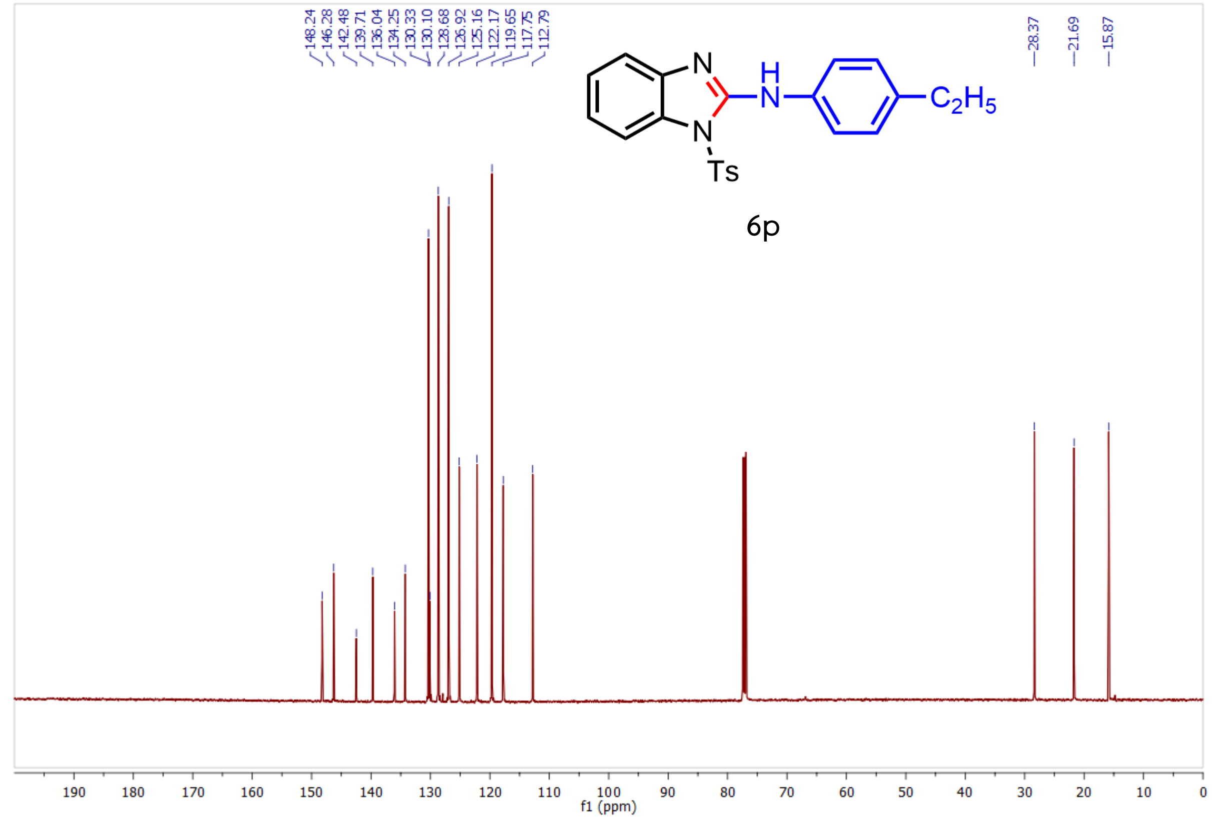


**Figure S68** $^{13}$C-NMR spectrum of **6p** ($CDCl_3$, 126 MHz)

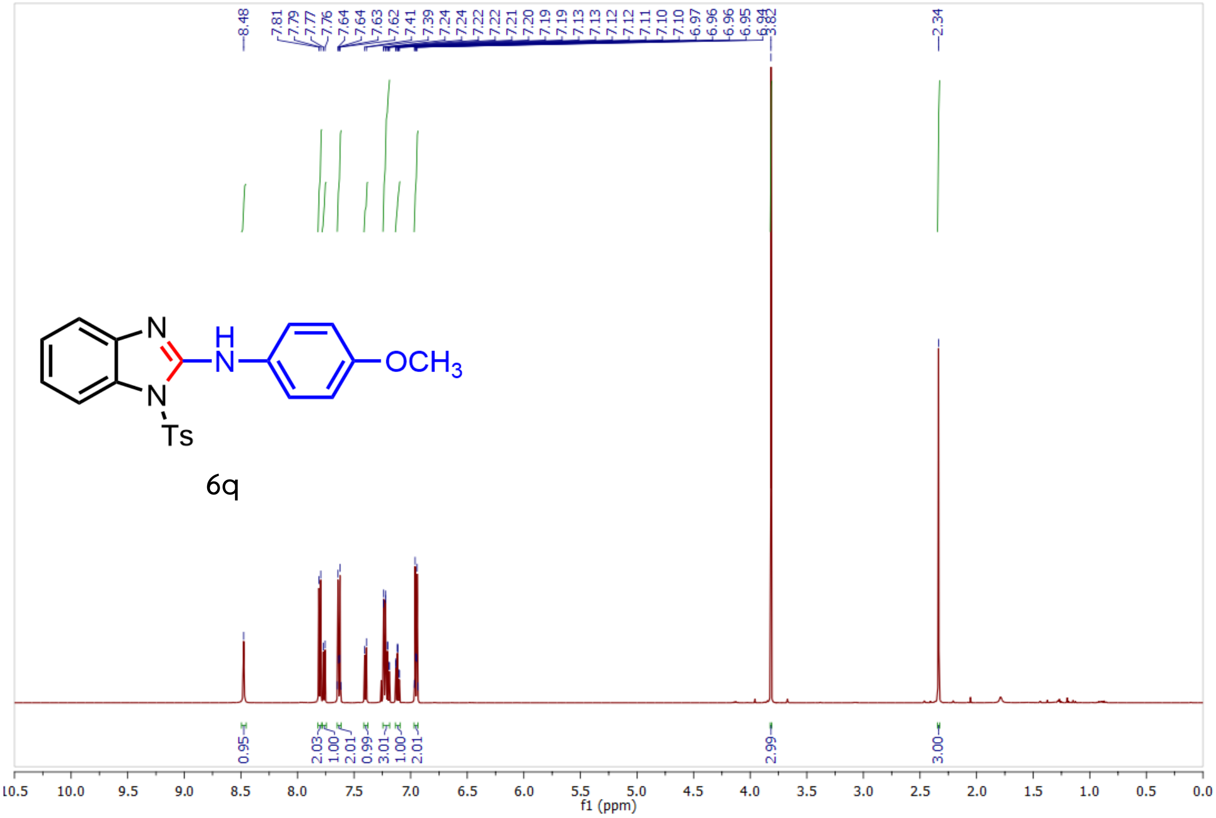


**Figure S69** $^{1}$H-NMR spectrum of **6q** ($CDCl_3$, 500 MHz)

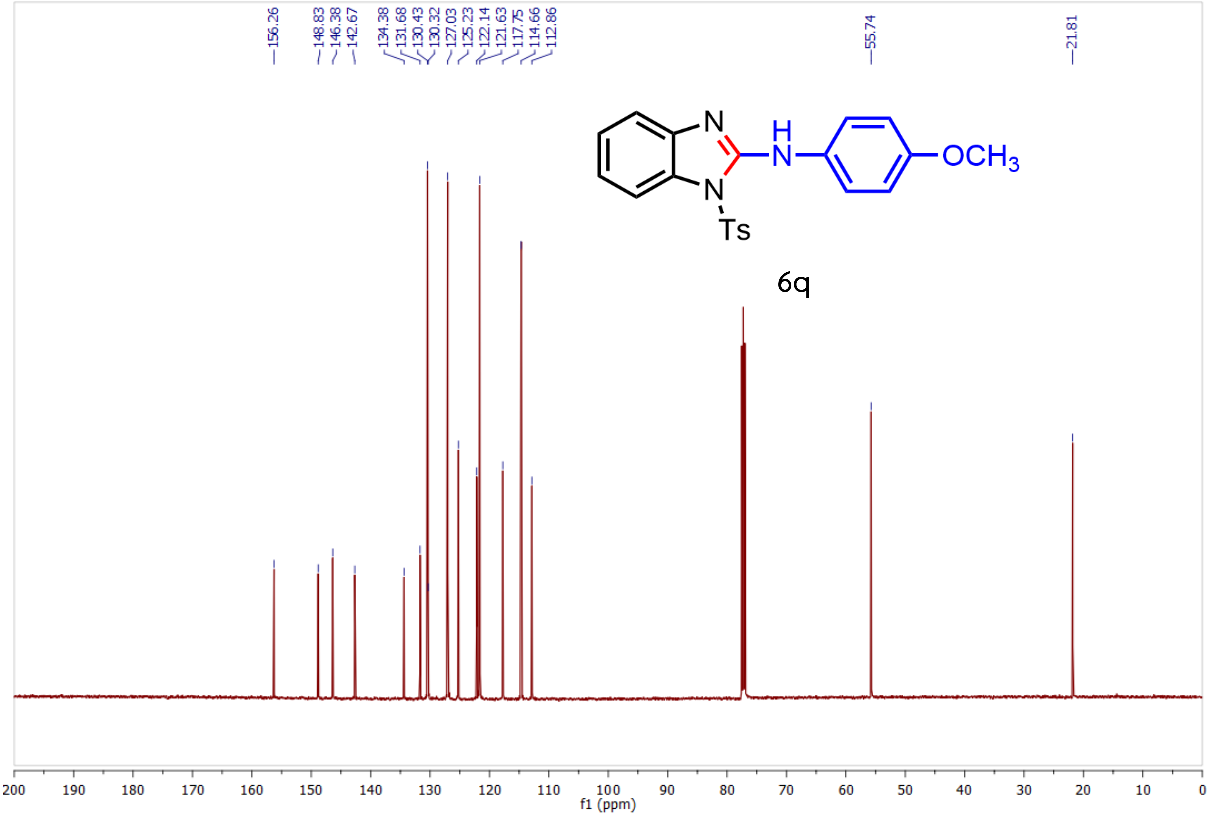


**Figure S70** $^{13}$C-NMR spectrum of **6q** ($CDCl_3$, 126 MHz)

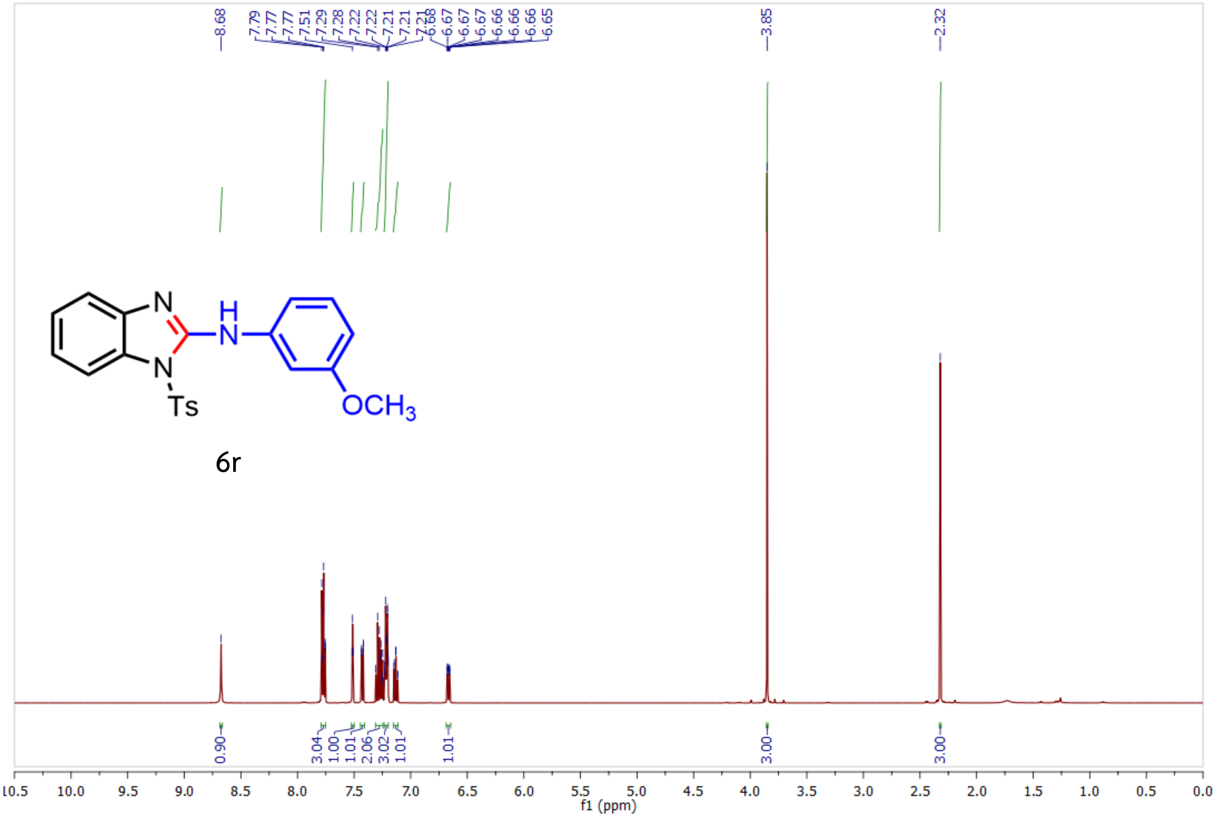


**Figure S71** $^{1}$H-NMR spectrum of **6r** ($CDCl_3$, 500 MHz)

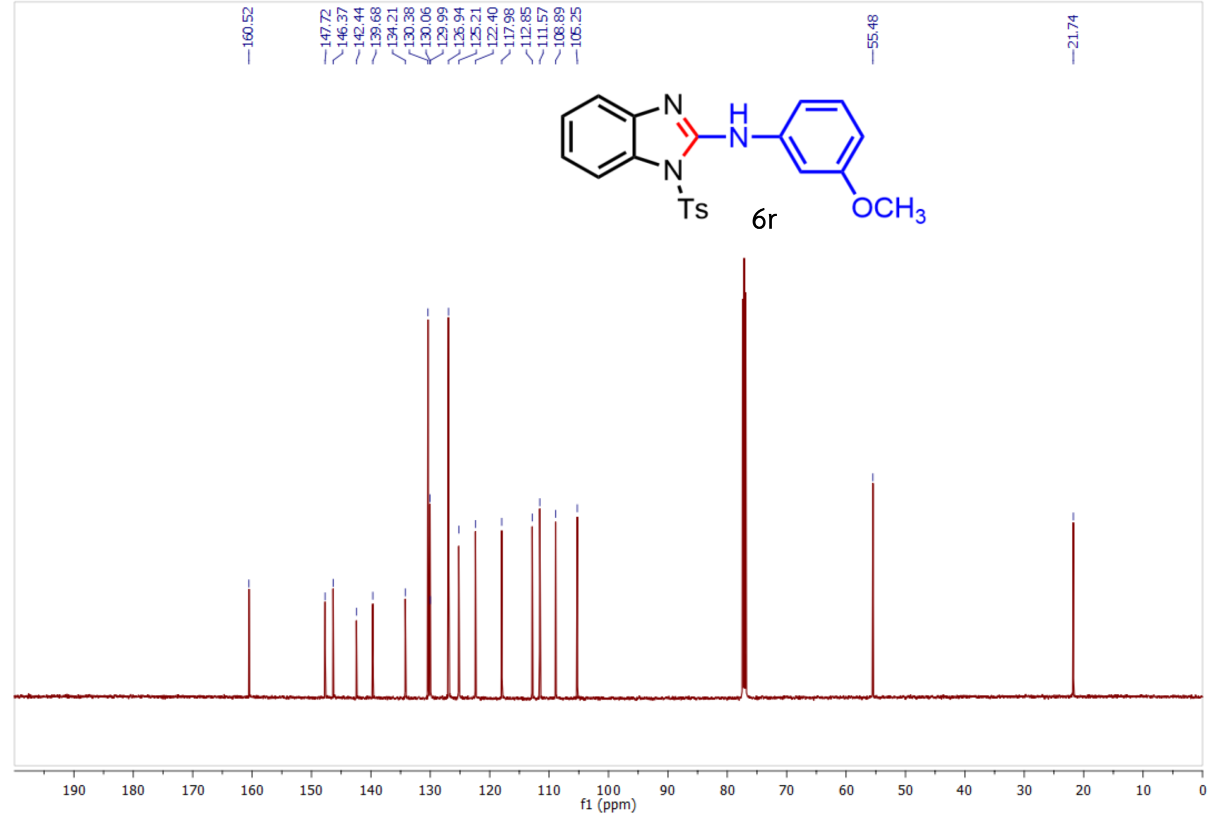


**Figure S72** $^{13}$C-NMR spectrum of **6r** ($CDCl_3$, 126 MHz)

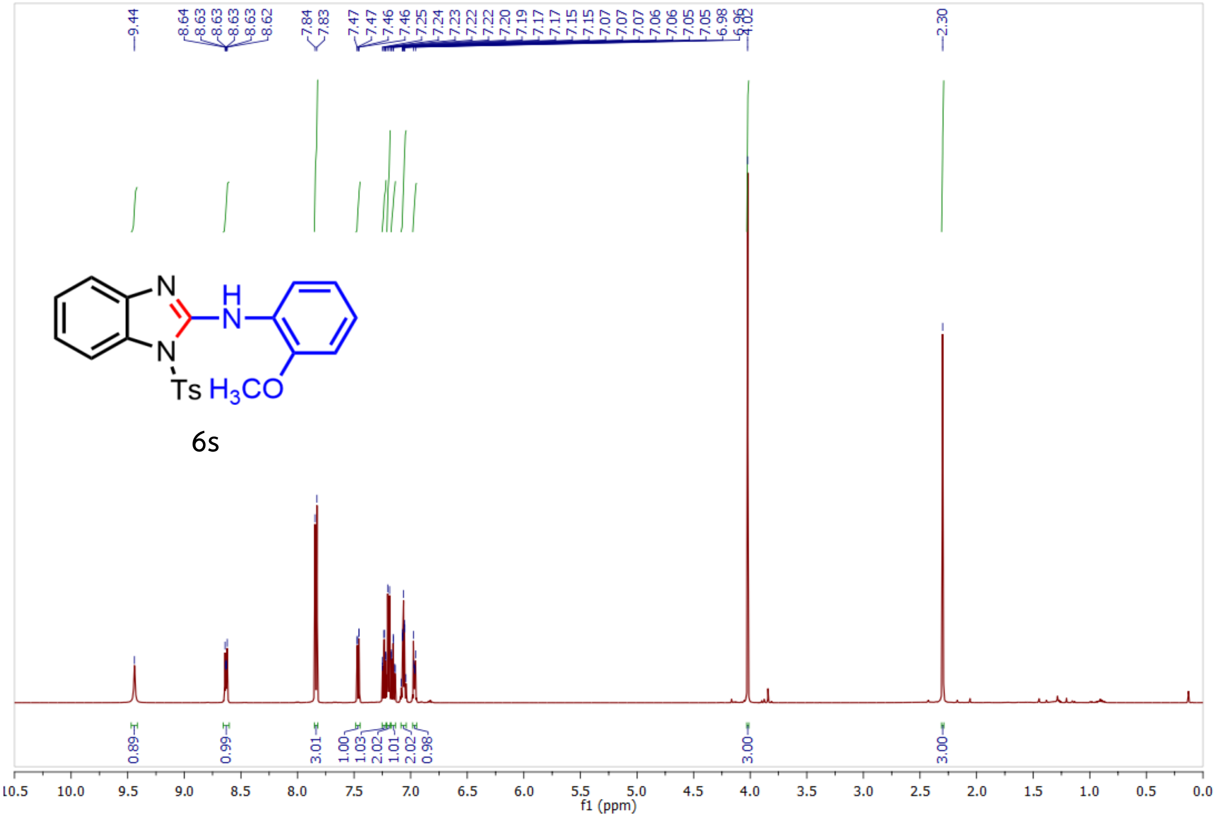

**Figure S73** $^{1}$H-NMR spectrum of **6s** ($CDCl_3$, 500 MHz)

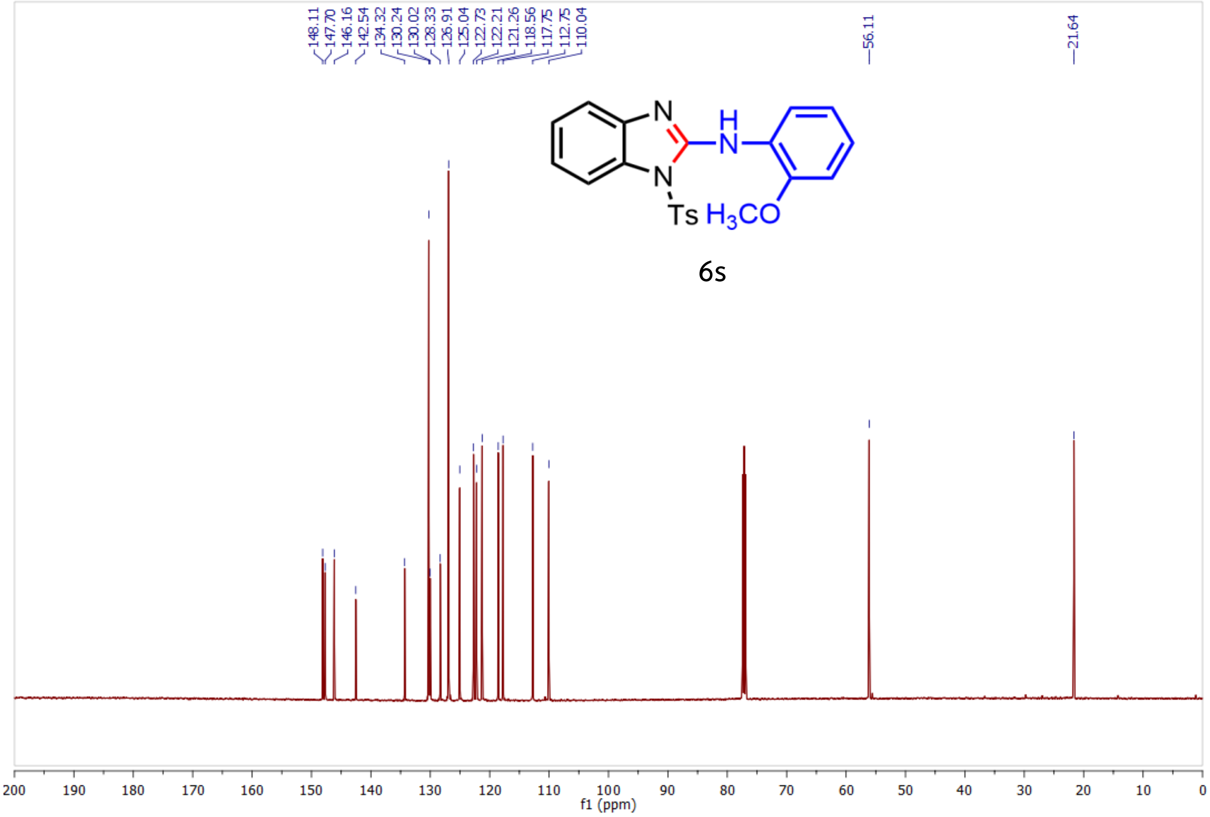

**Figure S74** $^{13}$C-NMR spectrum of **6s** ($CDCl_3$, 126 MHz)

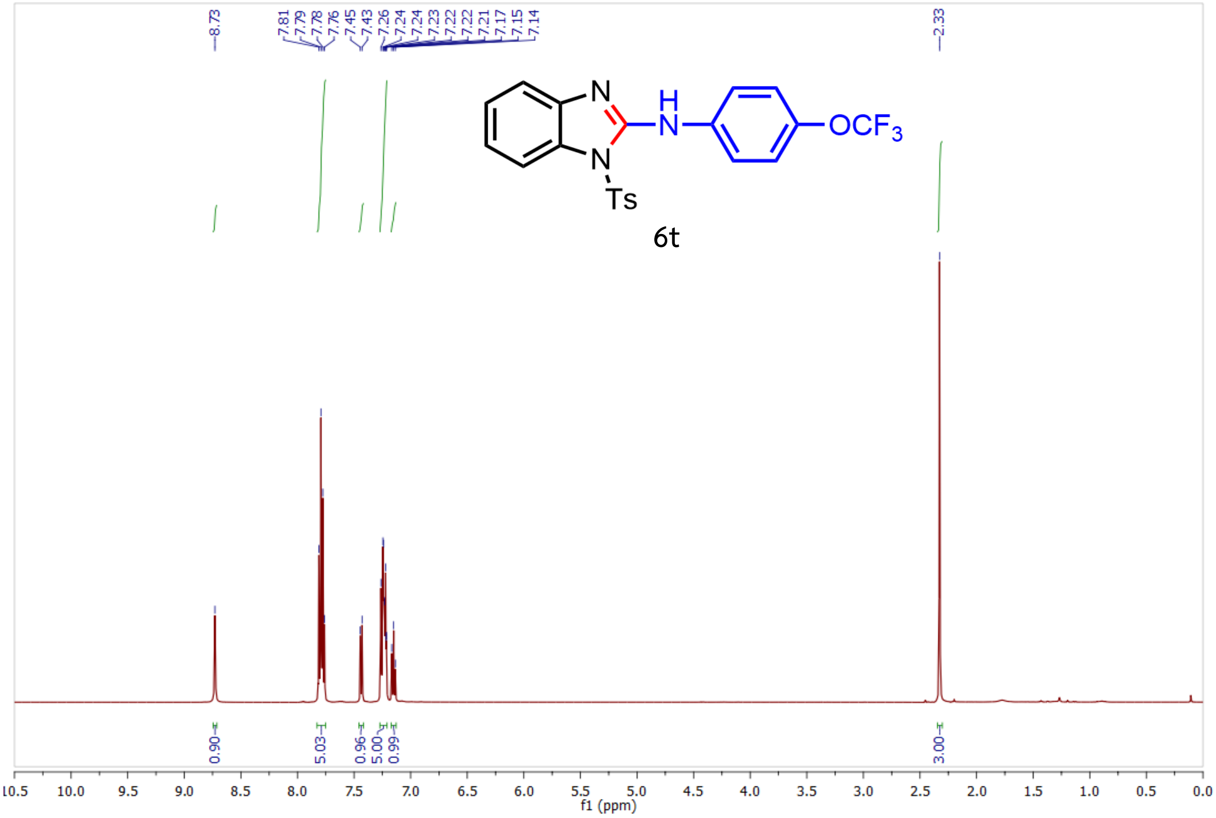


**Figure S75** $^{1}$H-NMR spectrum of **6t** ($CDCl_3$, 500 MHz)

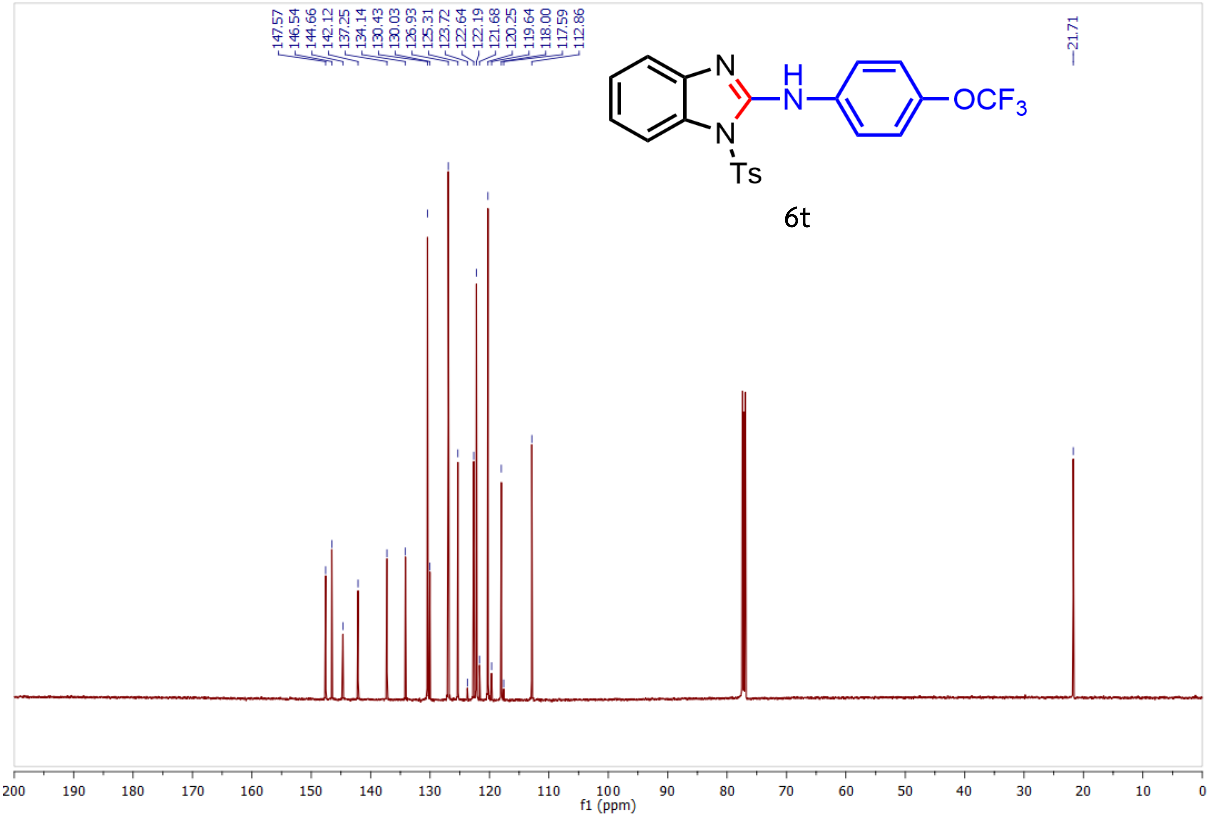


**Figure S76** $^{13}$C-NMR spectrum of **6t** ($CDCl_3$, 126 MHz)

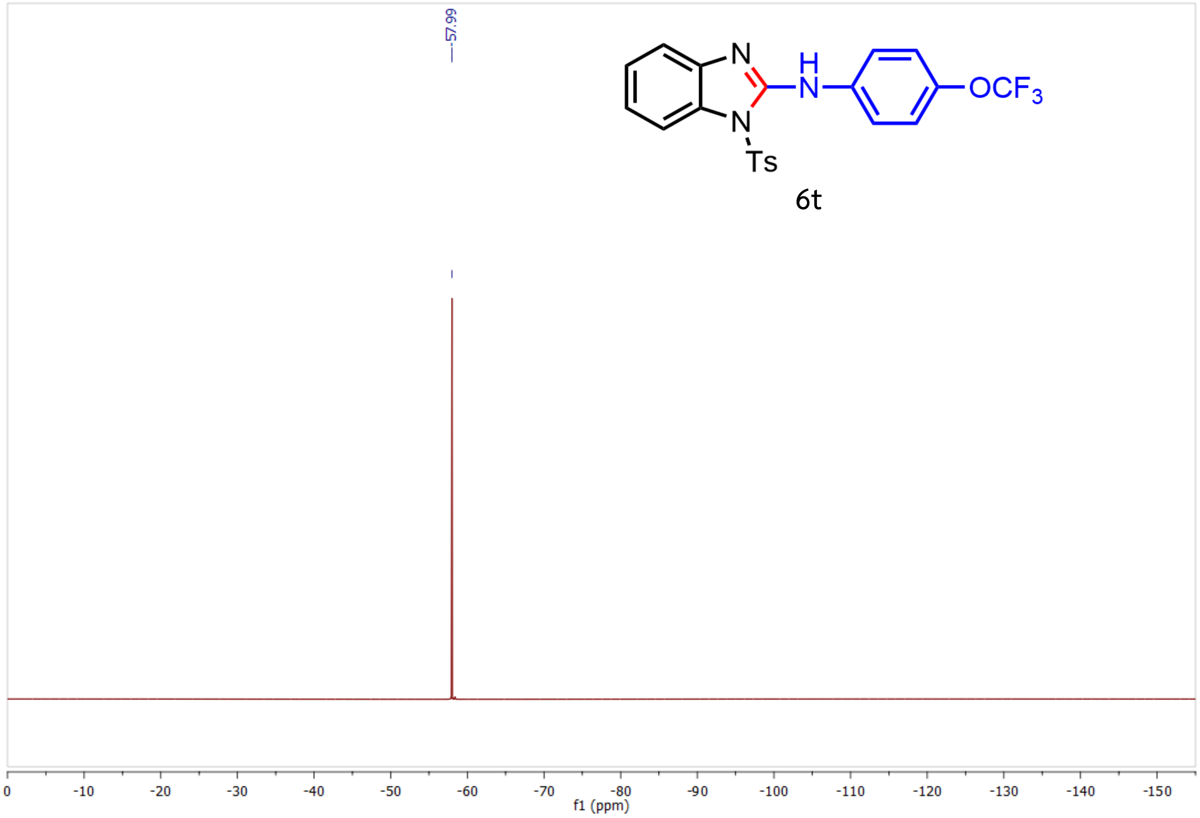


**Figure S77** $^{19}$F-NMR spectrum of **6t** ($CDCl_3$, 471 MHz)

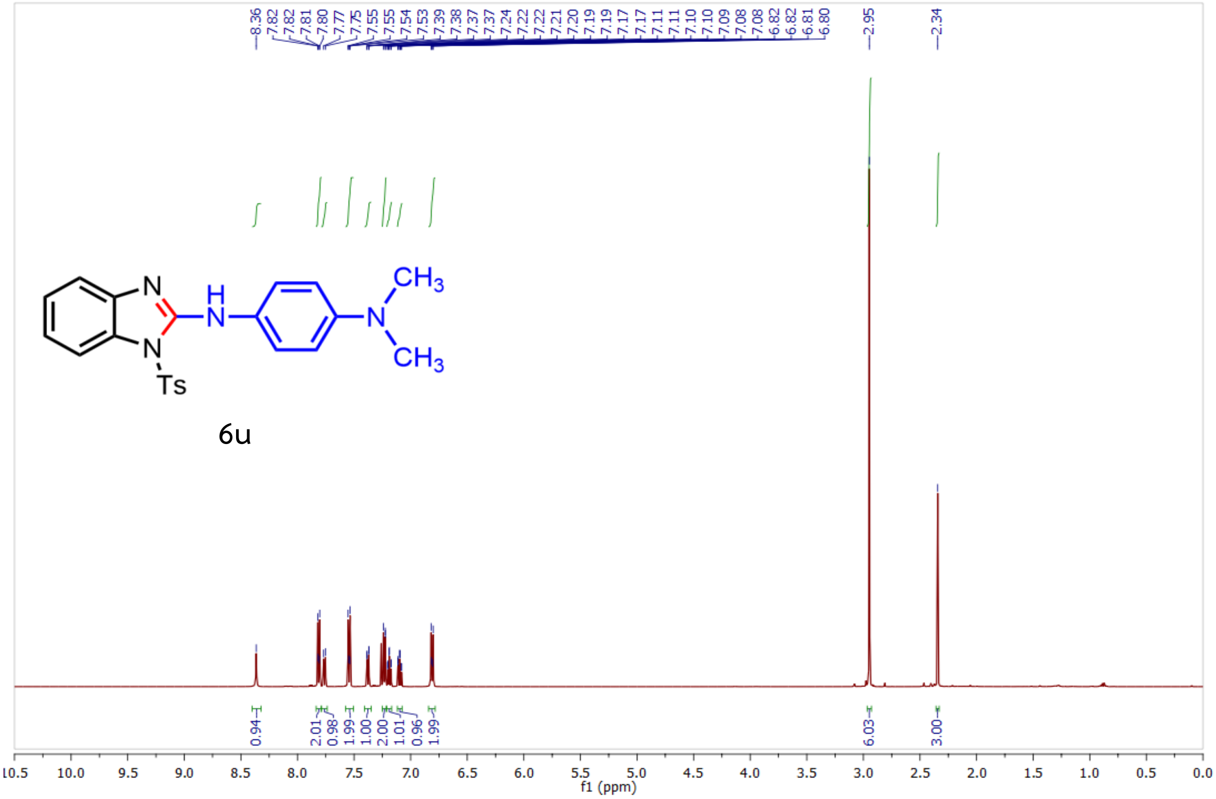

**Figure S78** $^{1}$H-NMR spectrum of **6u** ($CDCl_3$, 500 MHz)

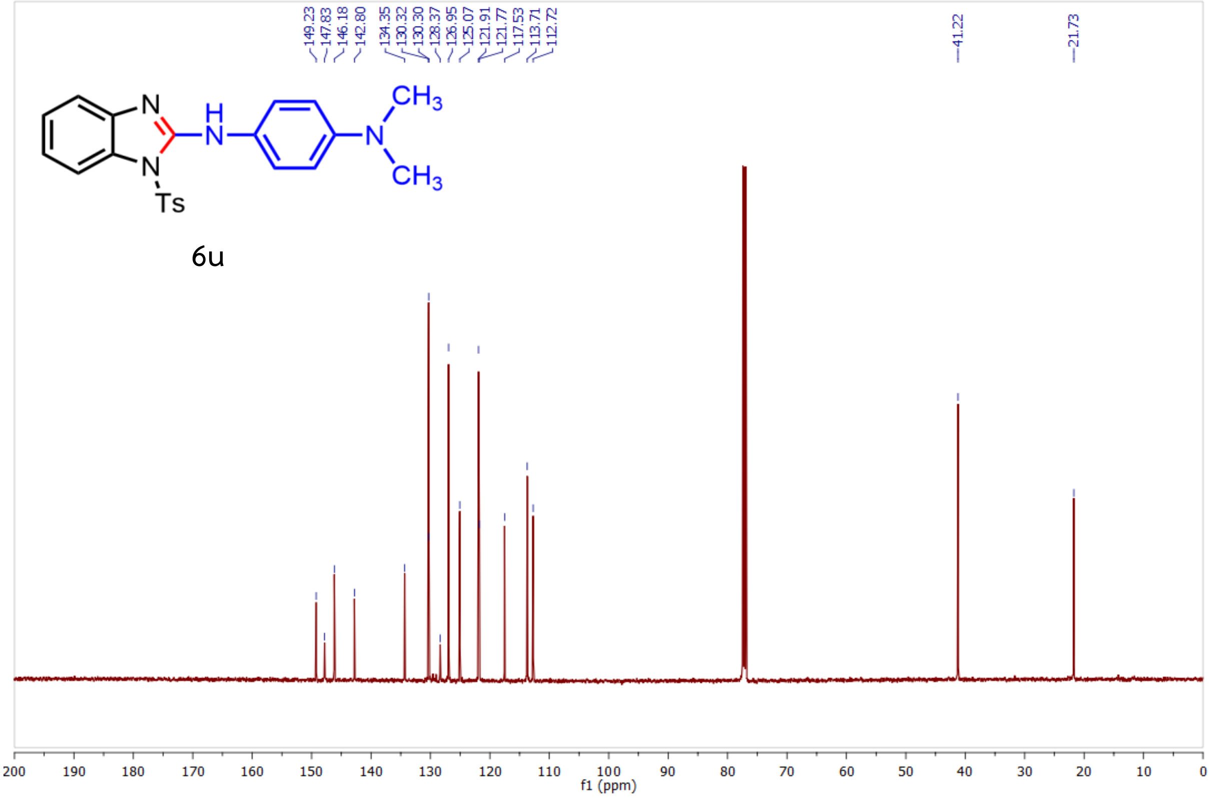

**Figure S79** $^{13}$C-NMR spectrum of **6u** ($CDCl_3$, 126 MHz)

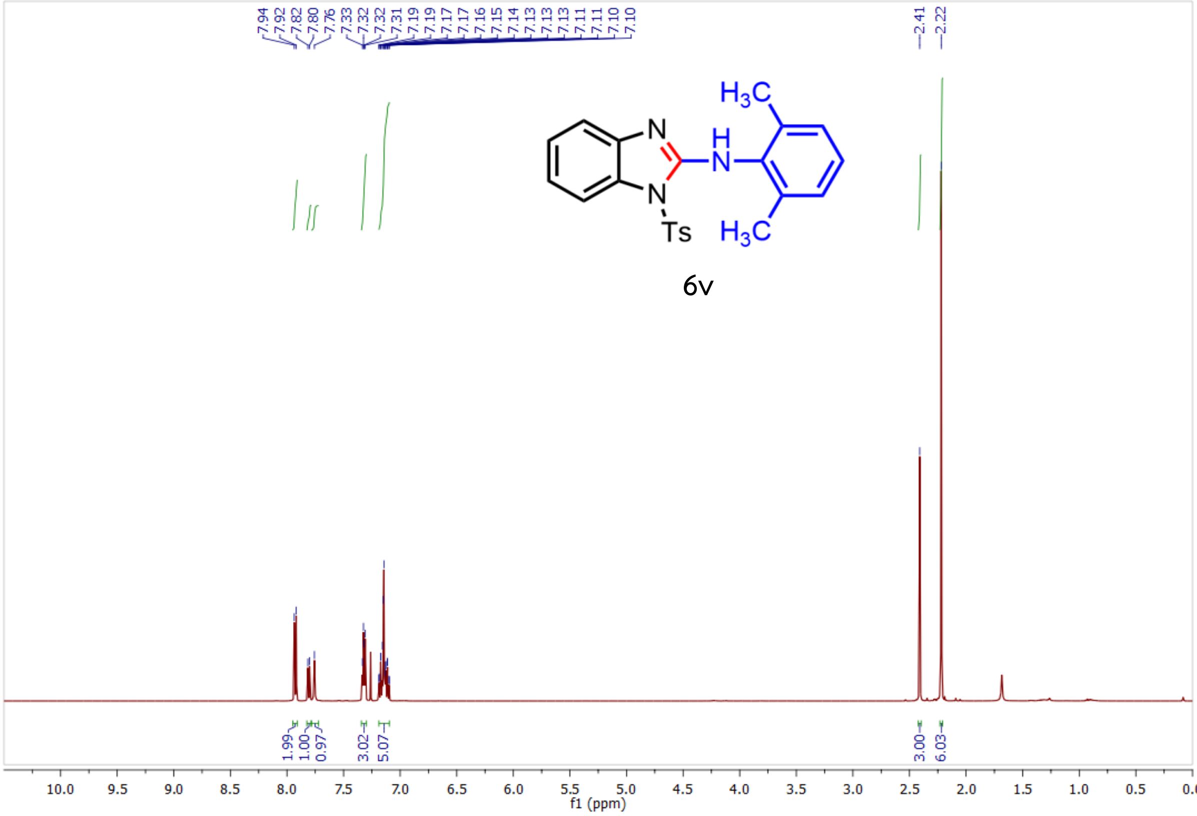


**Figure S80** $^{1}$H-NMR spectrum of **6v** ($CDCl_3$, 500 MHz)

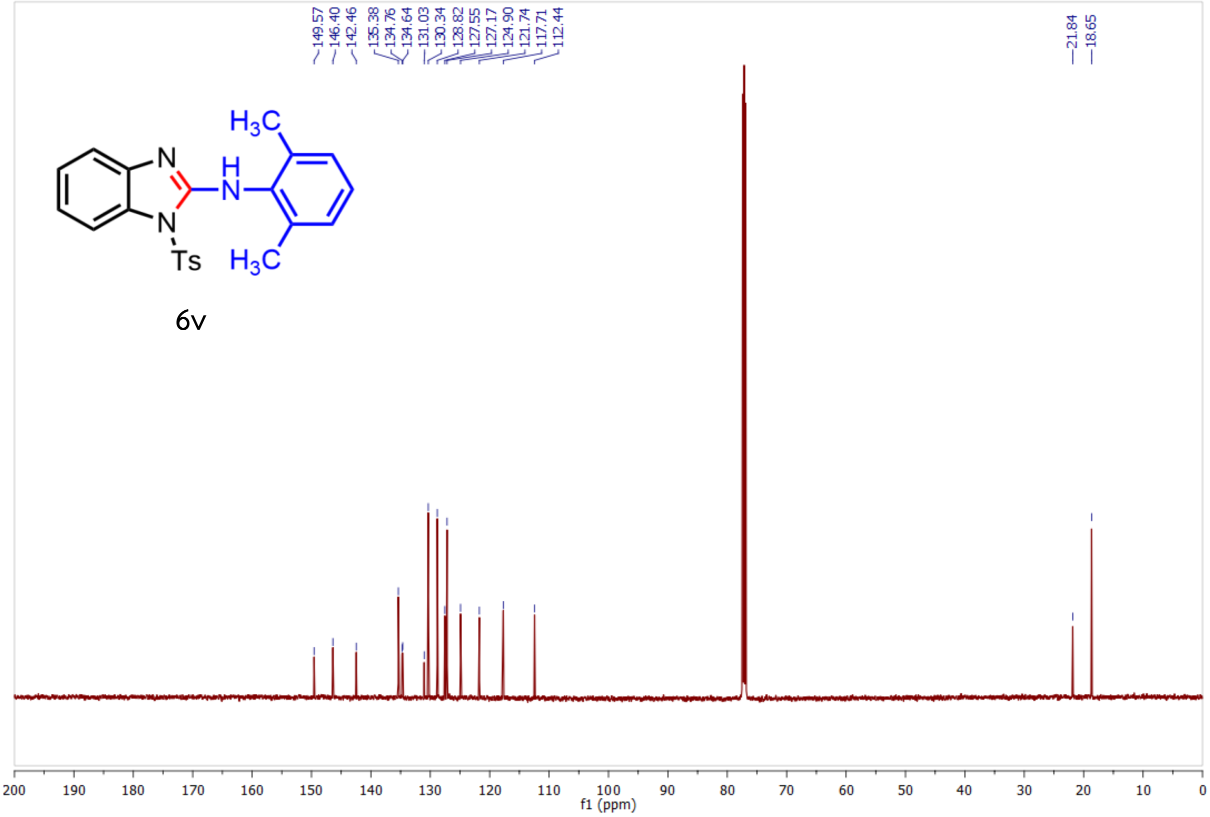


**Figure S81** $^{13}$C-NMR spectrum of **6v** ($CDCl_3$, 126 MHz)

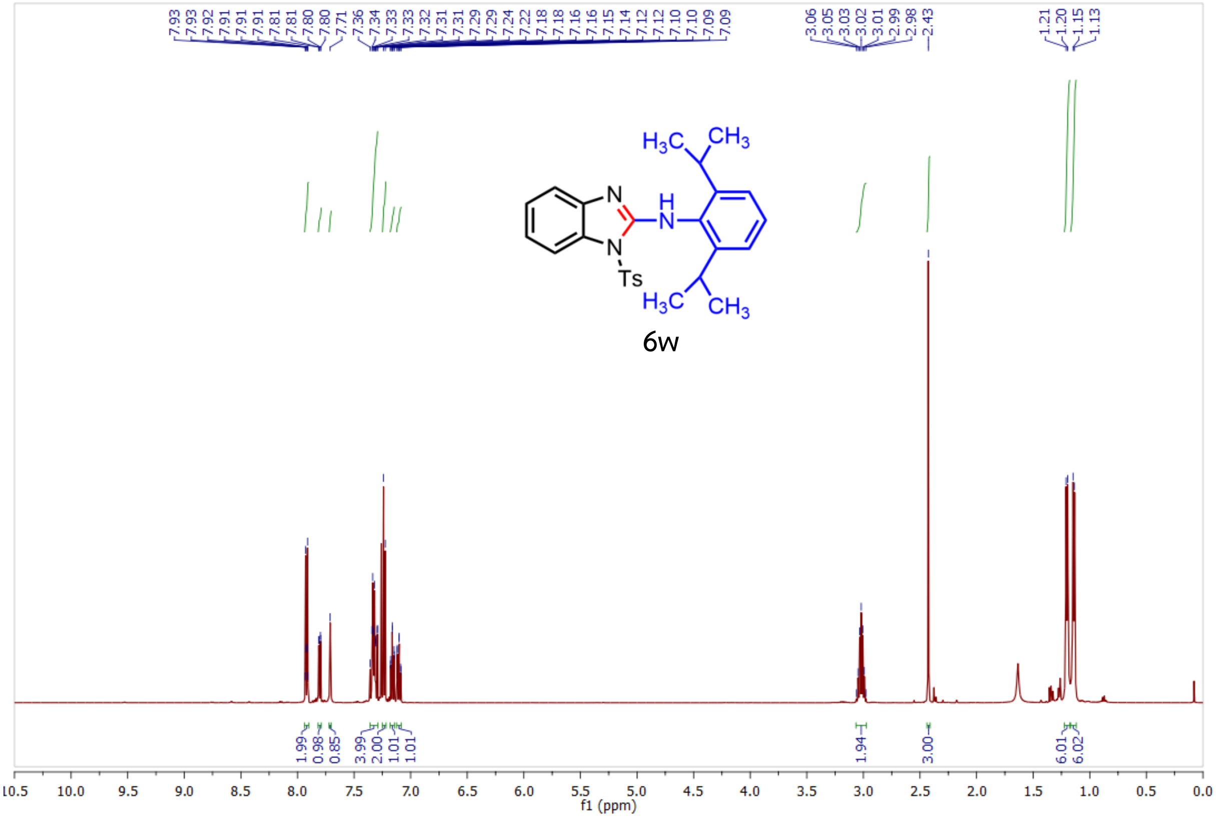

**Figure S82** $^{1}$H-NMR spectrum of **6w** ($CDCl_3$, 500 MHz)

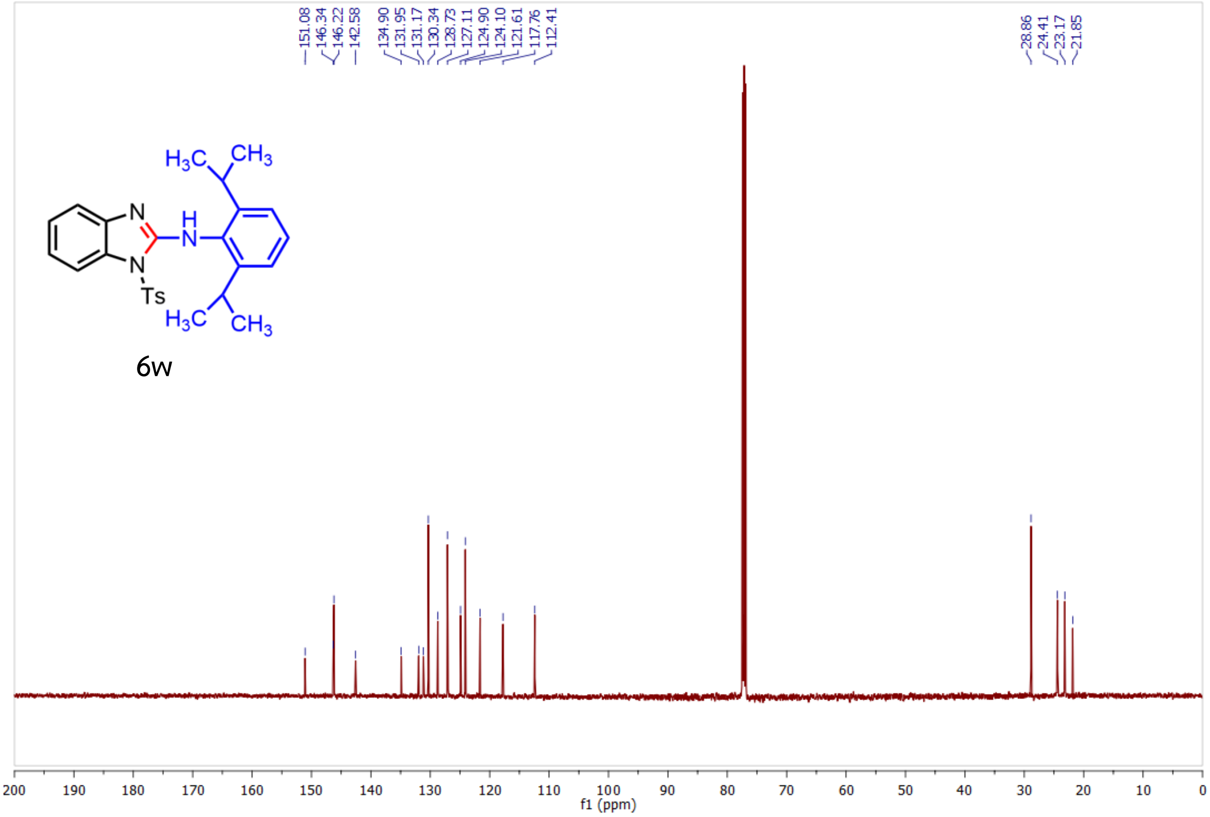

**Figure S83** $^{13}$C-NMR spectrum of **6w** ($CDCl_3$, 126 MHz)

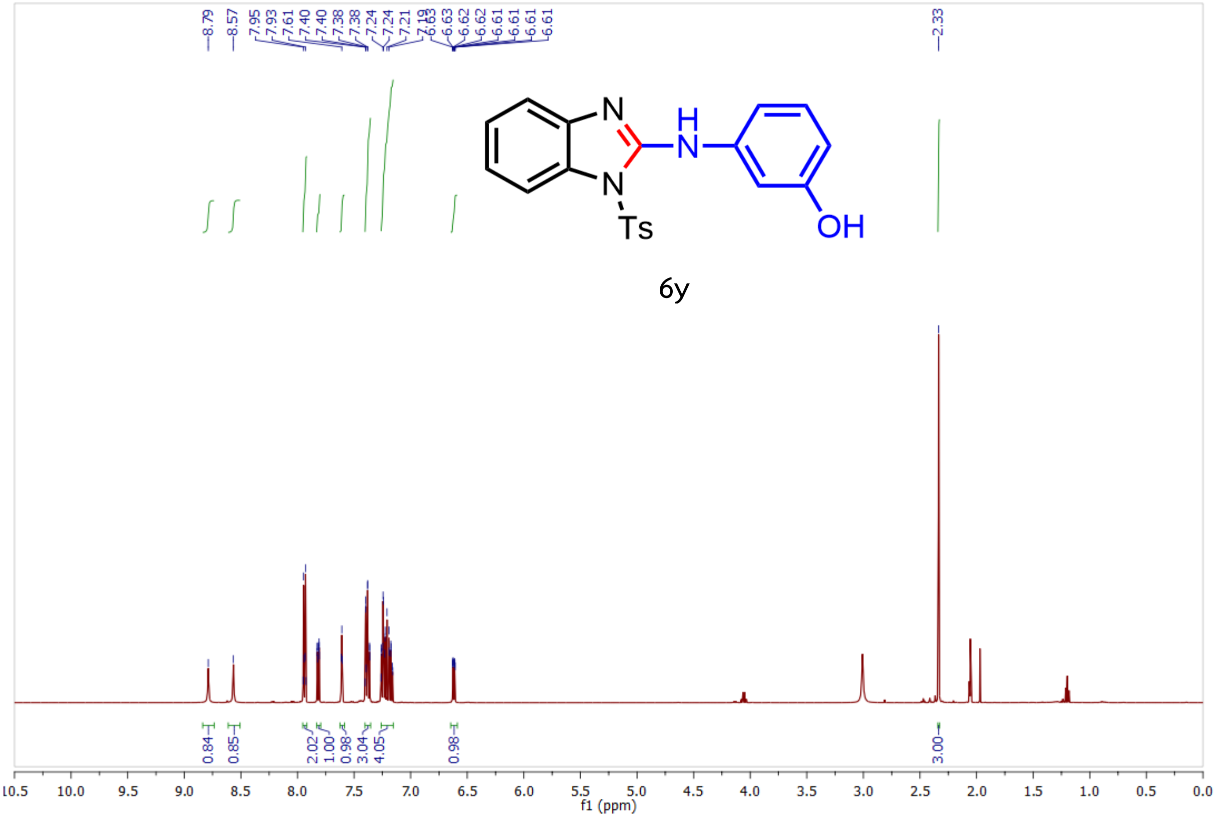


**Figure S84** $^{1}$H-NMR spectrum of **6y** (acetone-$d_6$ 500 MHz)

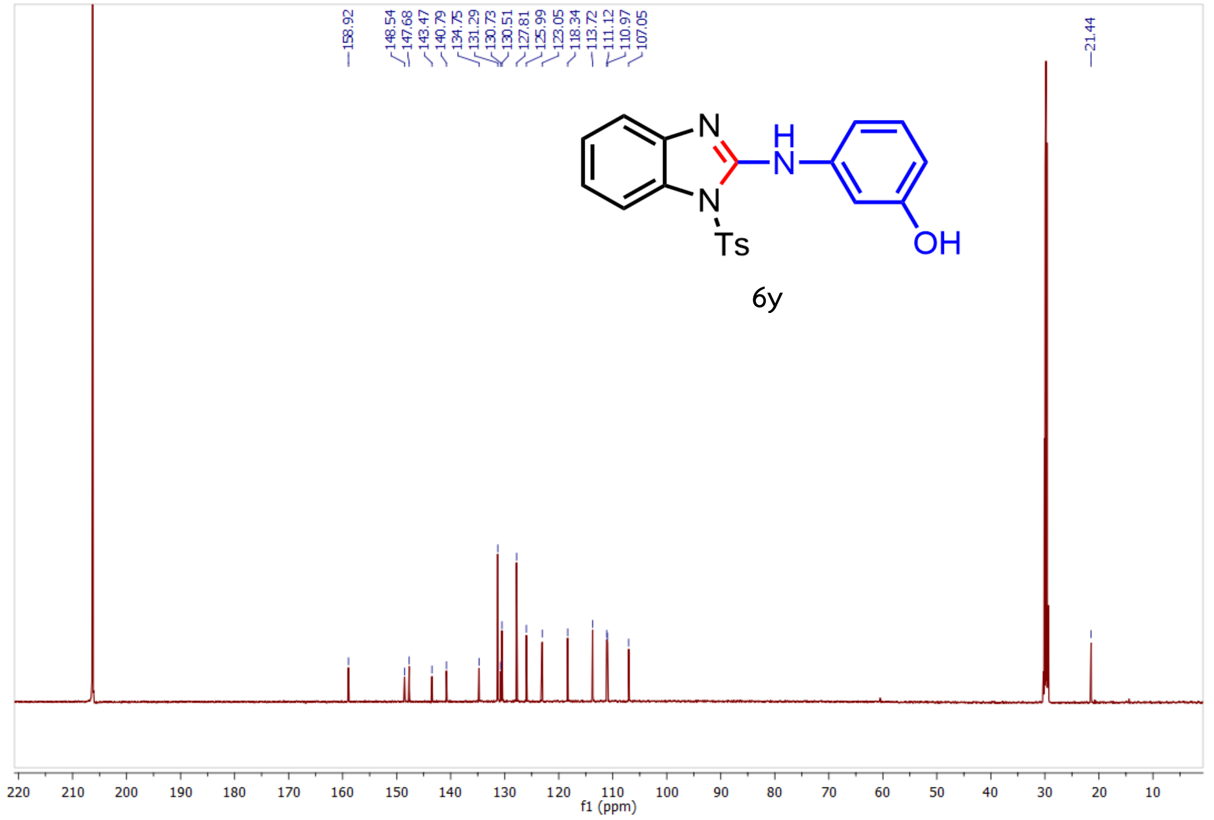


**Figure S85** $^{13}$C-NMR spectrum of **6y** (acetone-$d_6$, 126 MHz)

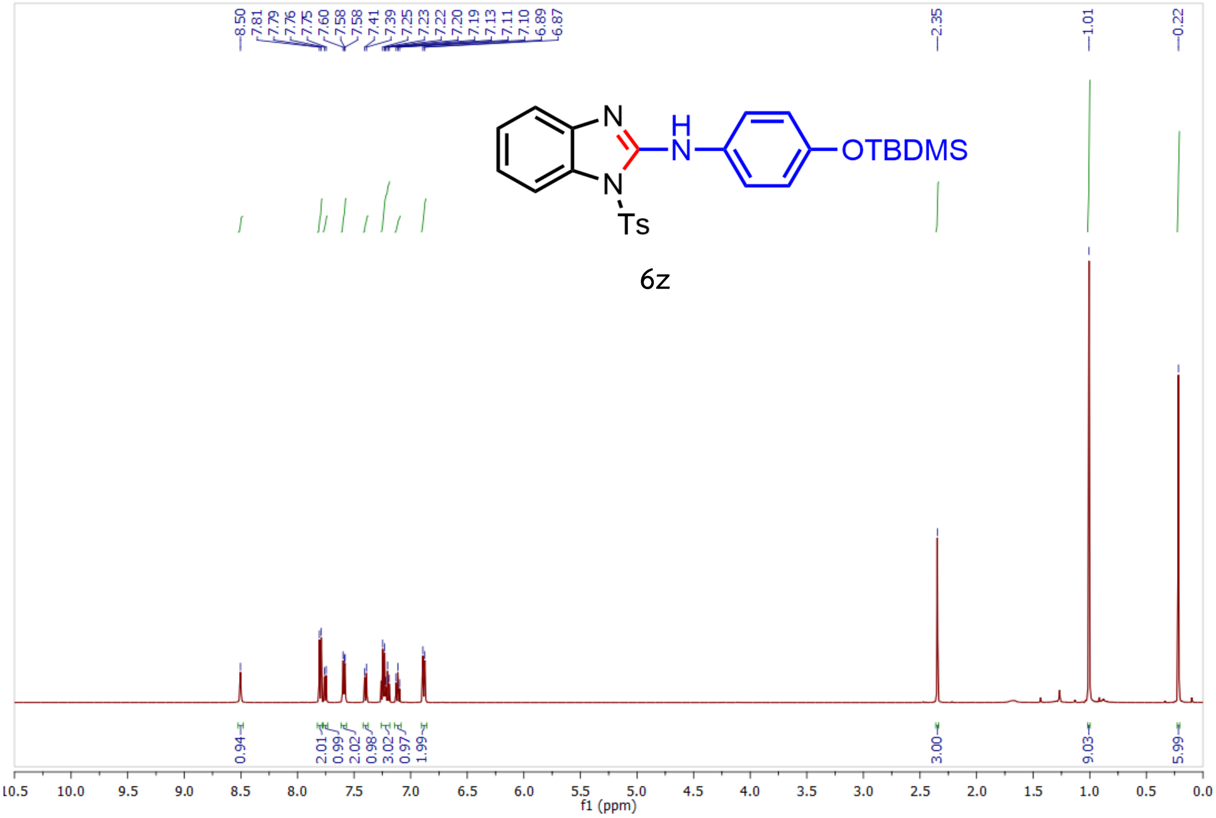

**Figure S86** $^{1}$H-NMR spectrum of **6z** ($CDCl_3$, 500 MHz)

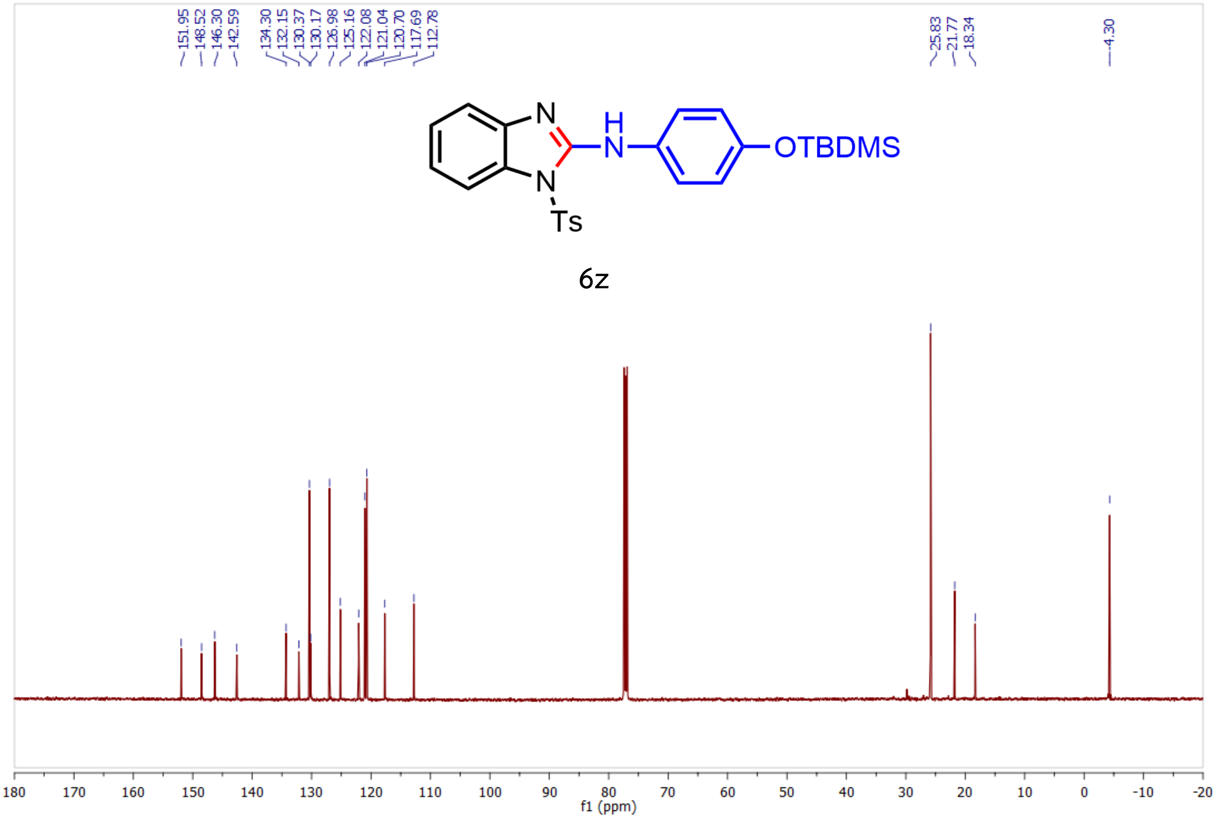

**Figure S87** $^{13}$C-NMR spectrum of **6z** ($CDCl_3$, 126 MHz)

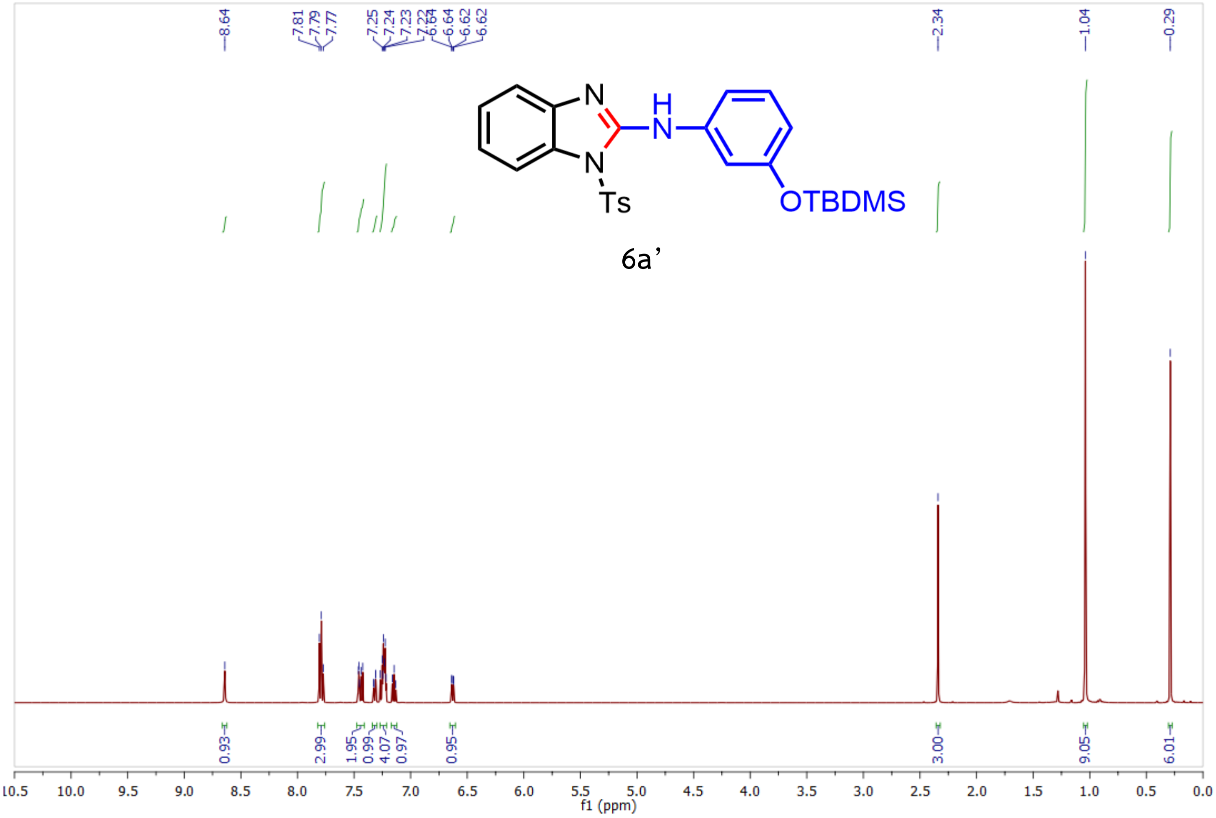


**Figure S88** $^{1}$H-NMR spectrum of **6a'** ($CDCl_3$, 500 MHz)

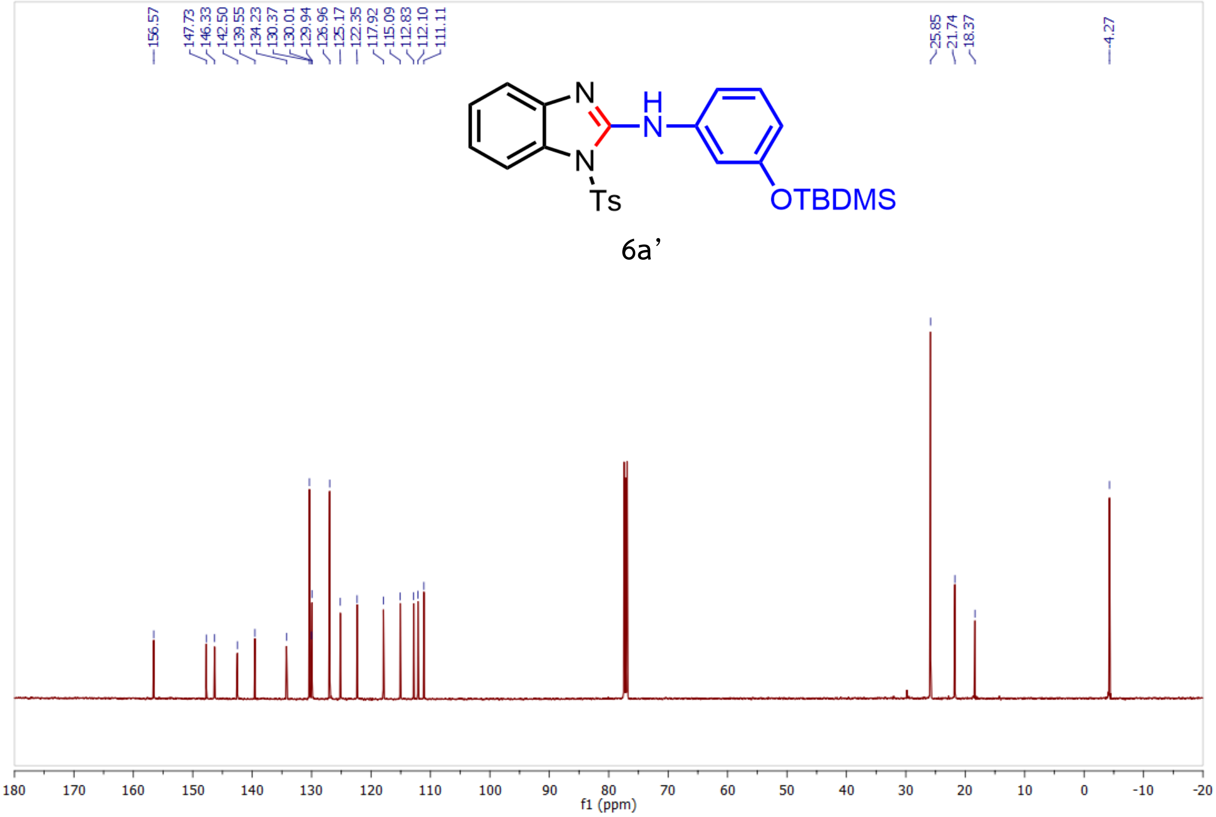


**Figure S89** $^{13}$C-NMR spectrum of **6a'** ($CDCl_3$, 126 MHz)

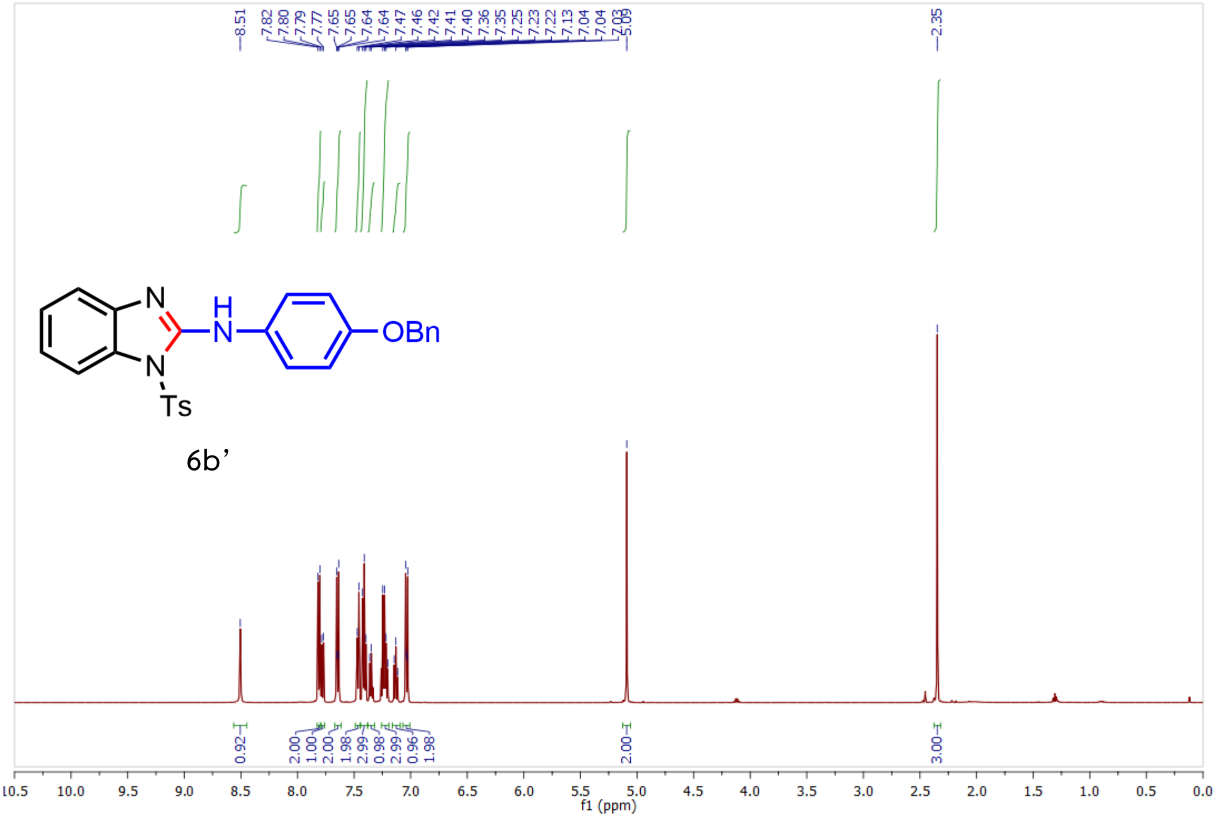


**Figure S90** $^{1}$H-NMR spectrum of **6b’** (CDCl$_3$, 500 MHz)

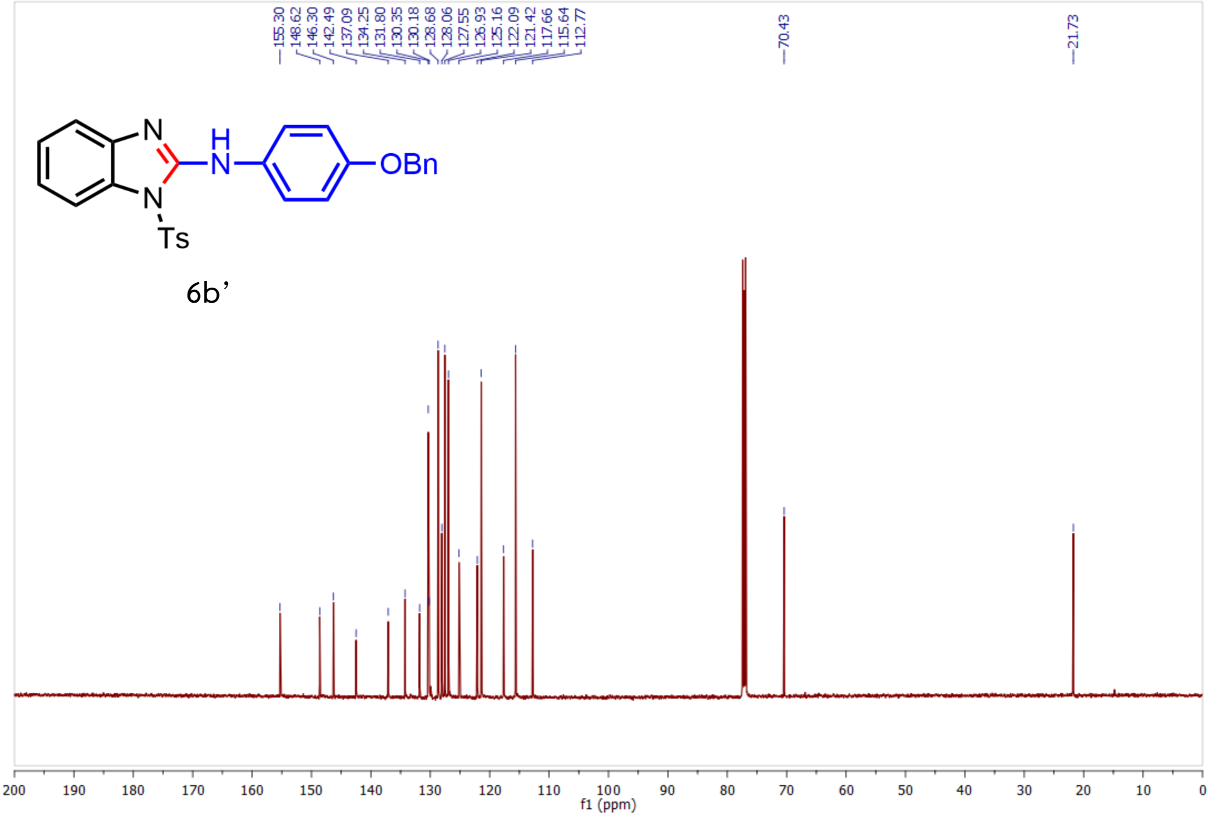


**Figure S91** $^{13}$C-NMR spectrum of **6b’** (CDCl$_3$, 126 MHz)

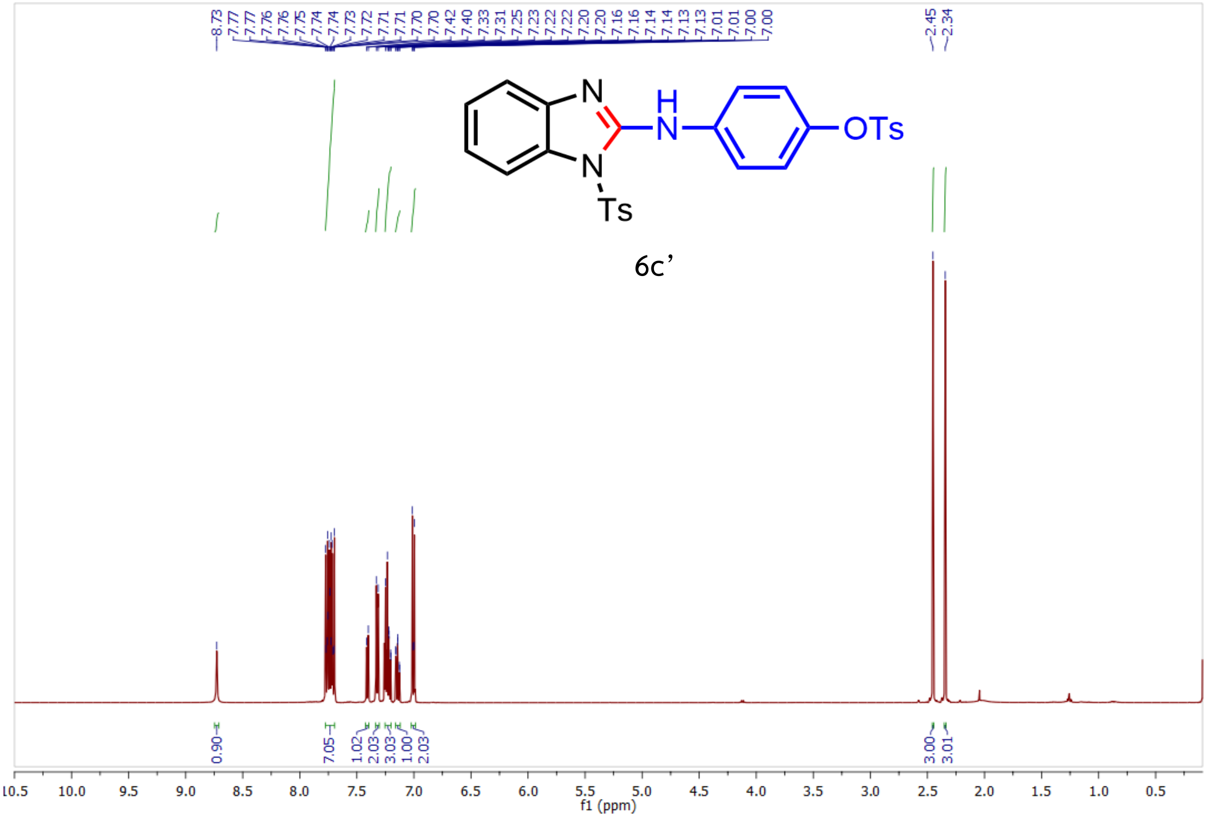

**Figure S92** $^{1}$H-NMR spectrum of **6c’** ($CDCl_3$, 500 MHz)

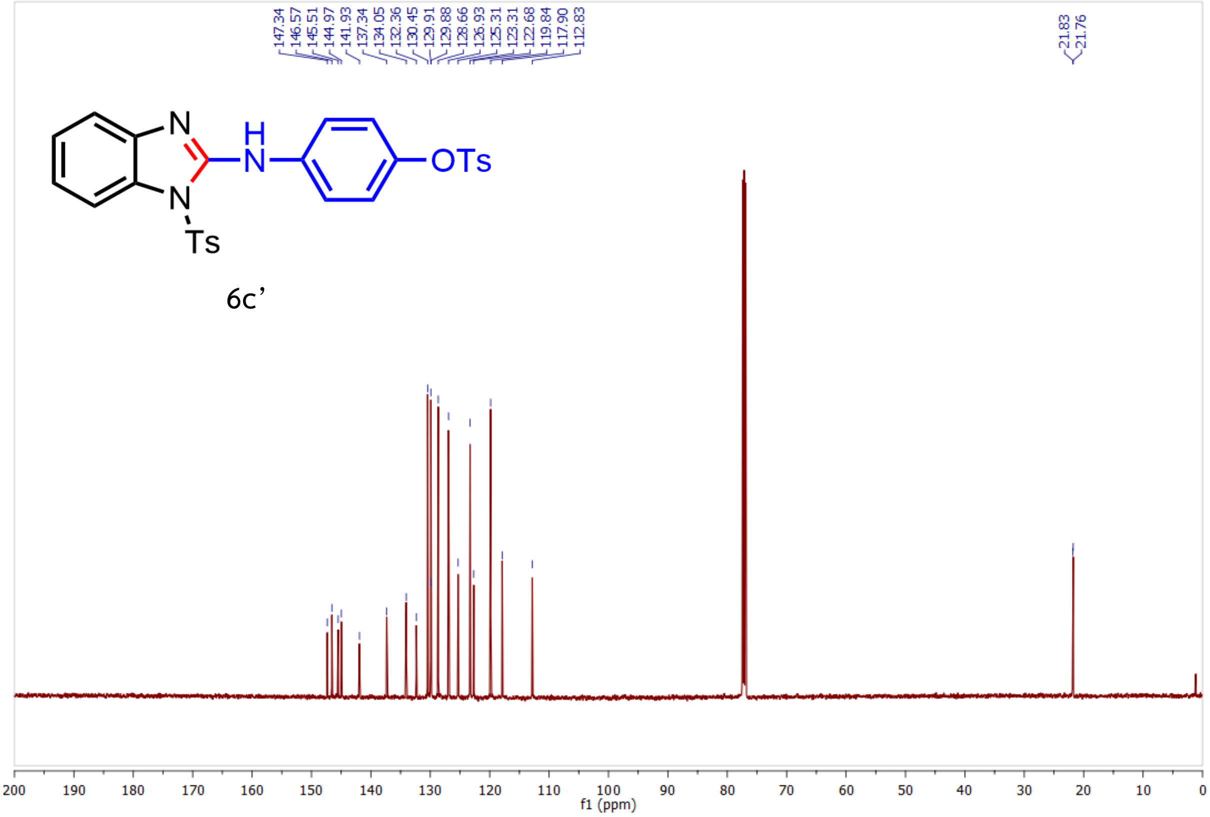

**Figure S93** $^{13}$C-NMR spectrum of **6c’** ($CDCl_3$, 126 MHz)

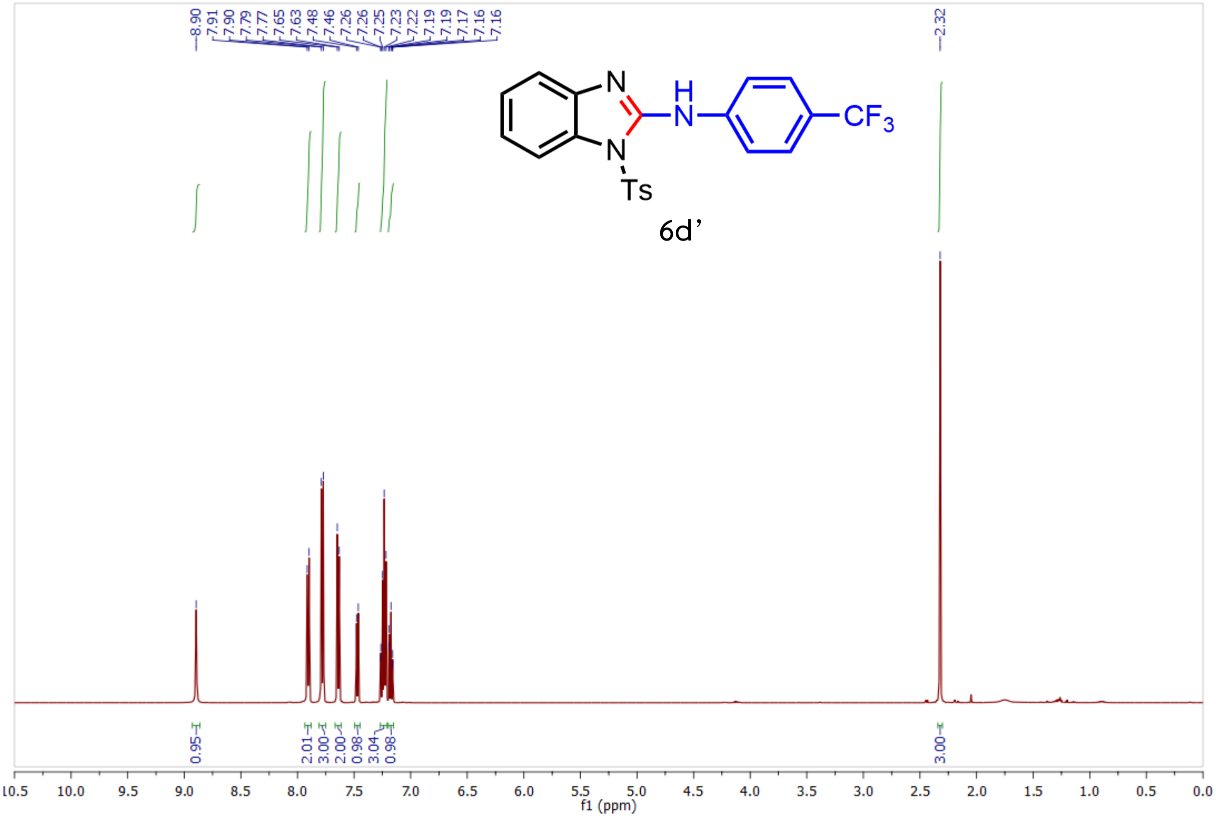


**Figure S94** $^{1}$H-NMR spectrum of **6d'** ($CDCl_3$, 500 MHz)

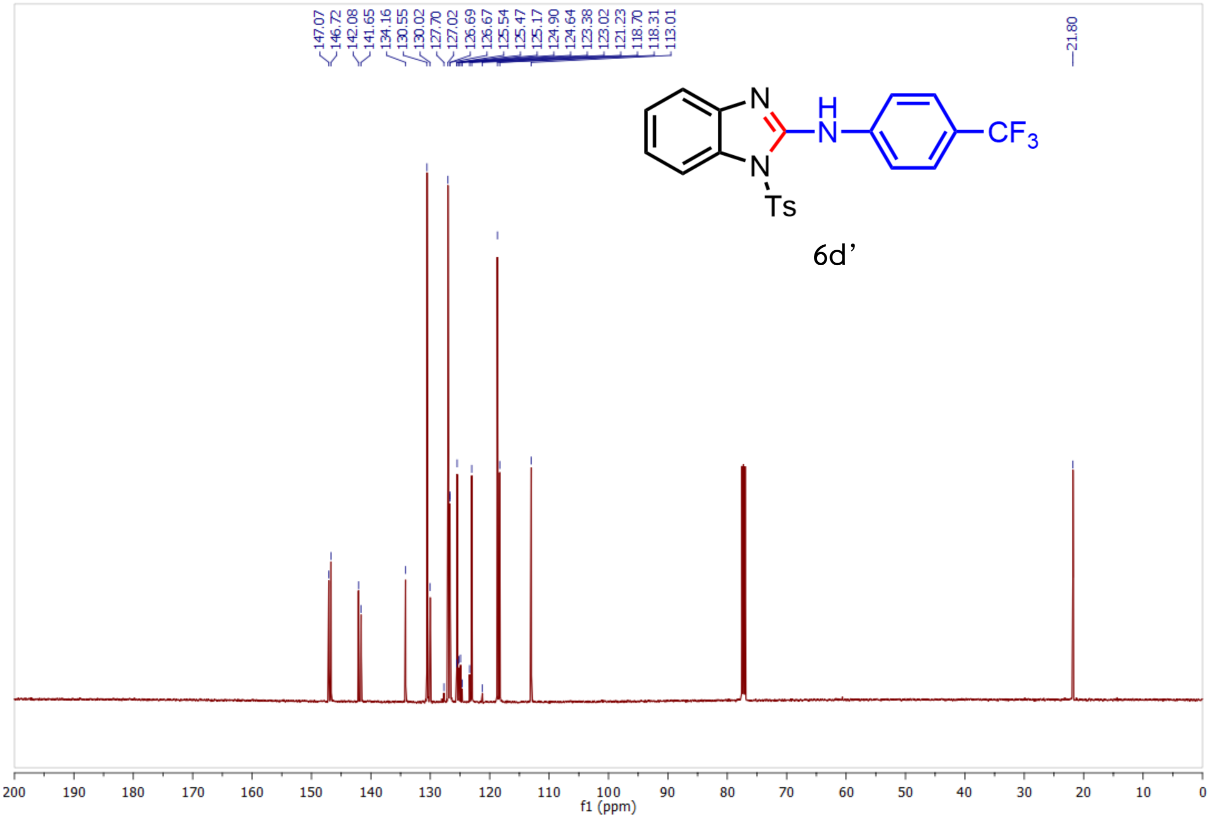


**Figure S95** $^{13}$C-NMR spectrum of **6d'** ($CDCl_3$, 126 MHz)

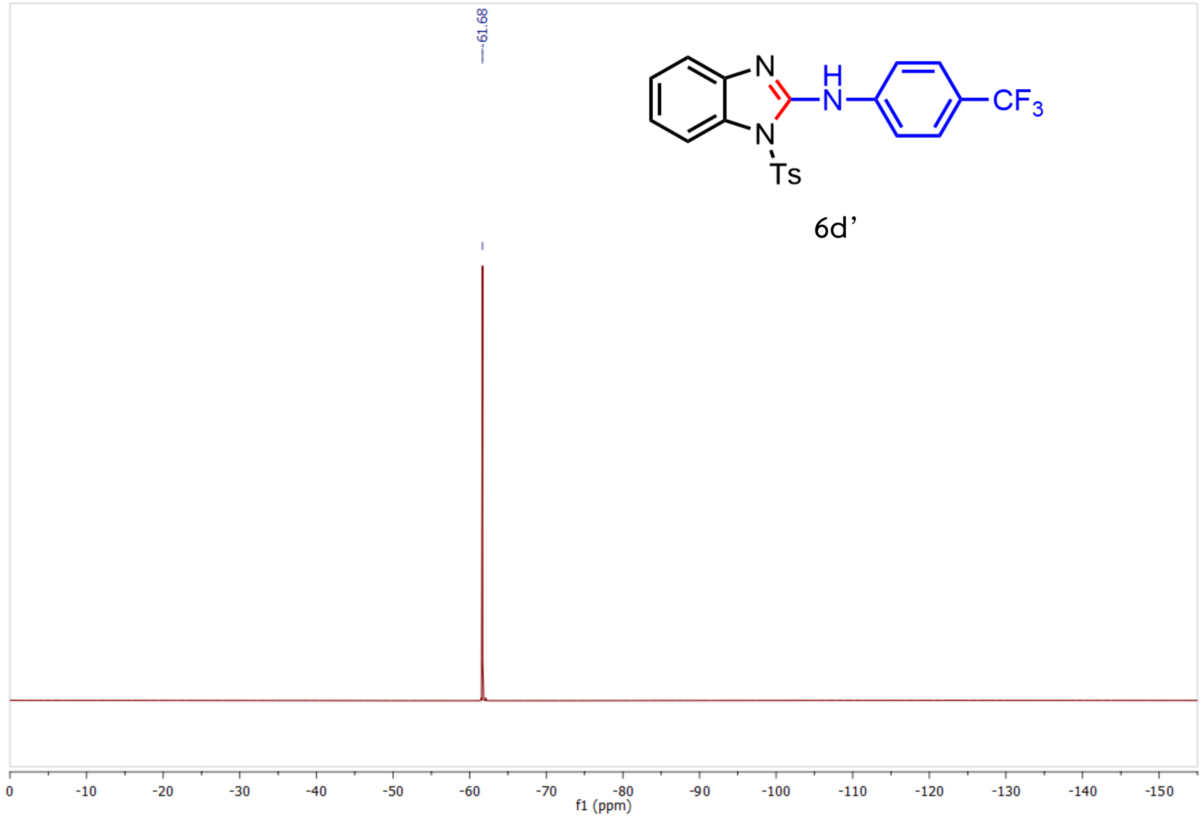


**Figure S96** $^{19}$F-NMR spectrum of **6d'** ($CDCl_3$, 471 MHz)

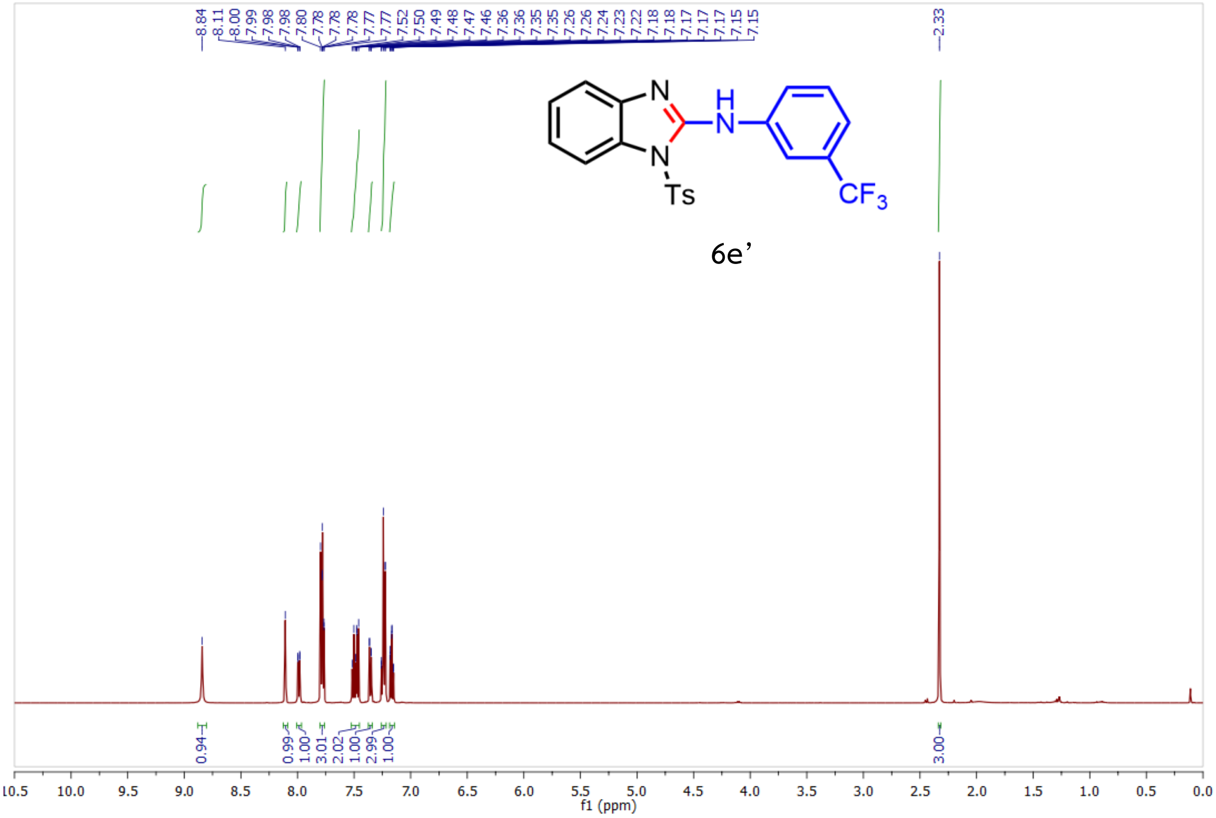

**Figure S97** $^{1}$H-NMR spectrum of **6e'** ($CDCl_3$, 500 MHz)

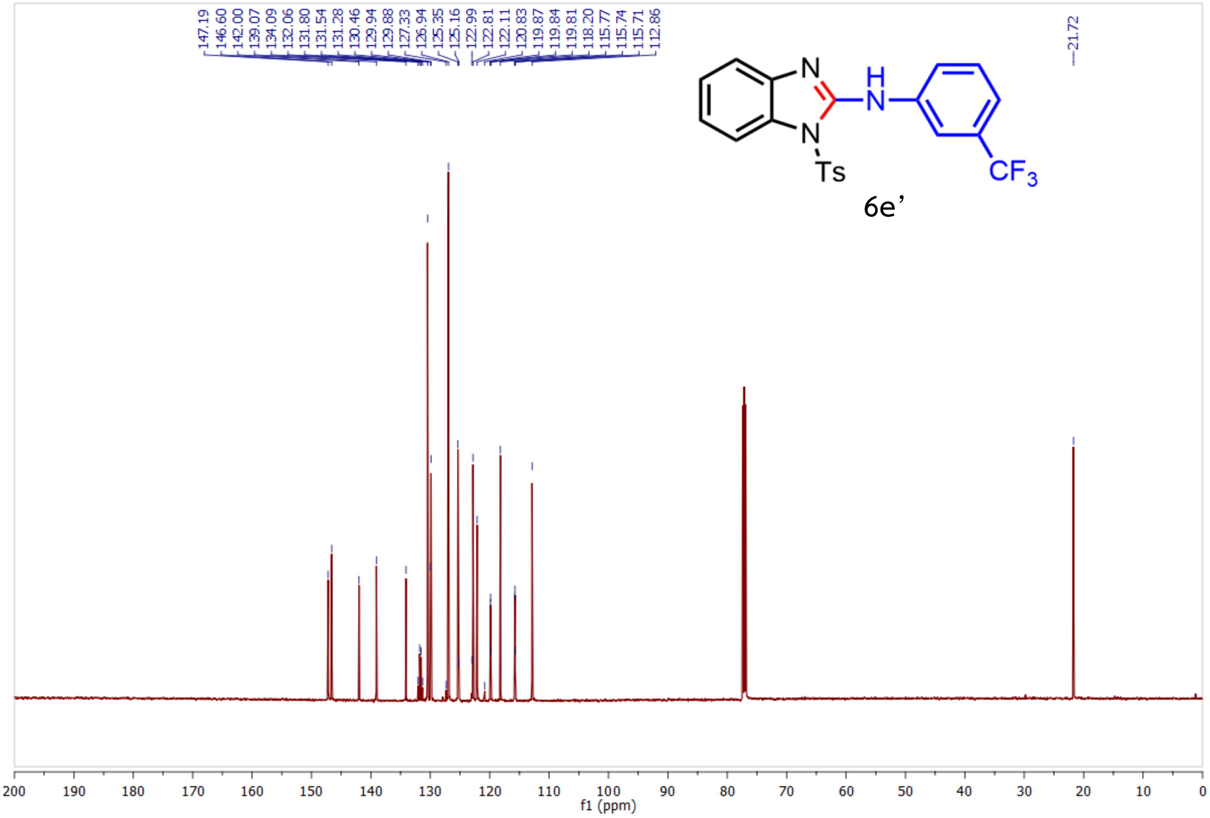

**Figure S98** $^{13}$C-NMR spectrum of **6e'** ($CDCl_3$, 126 MHz)

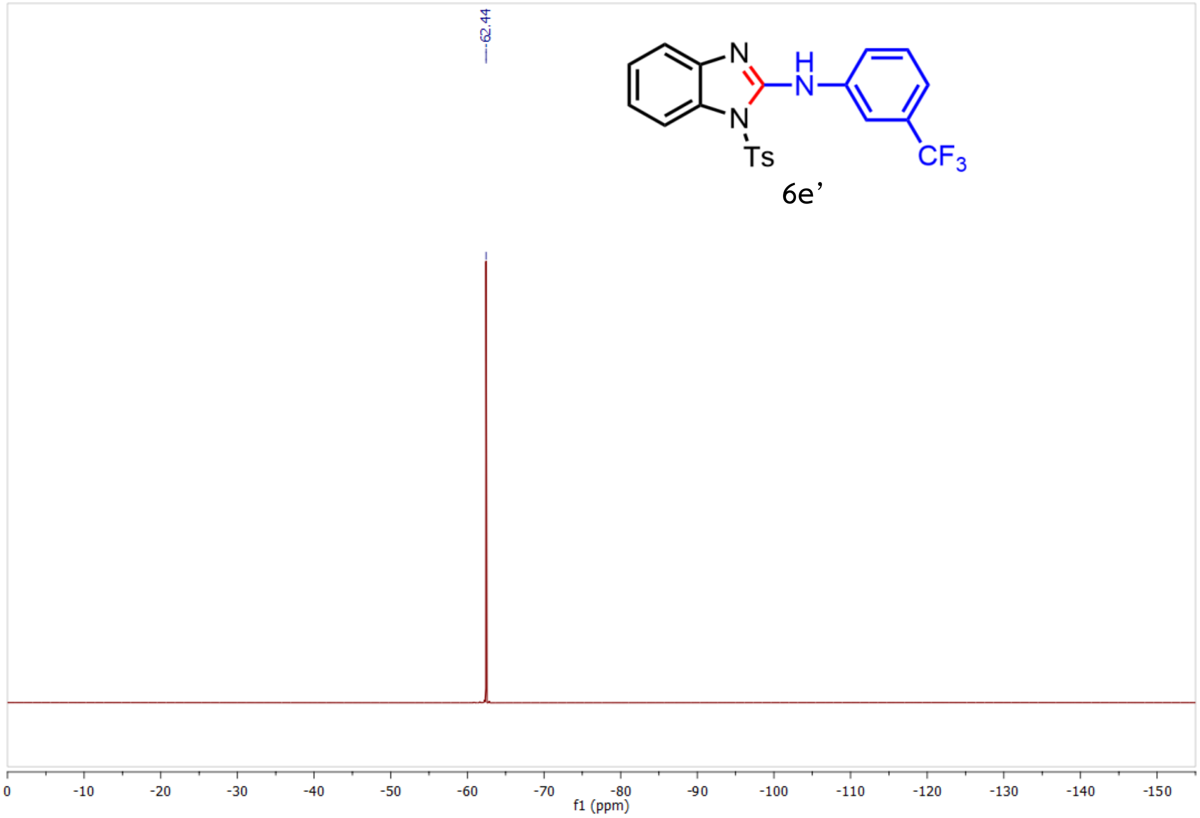


**Figure S99** $^{19}$F-NMR spectrum of **6e’** ($CDCl_3$, 471 MHz)

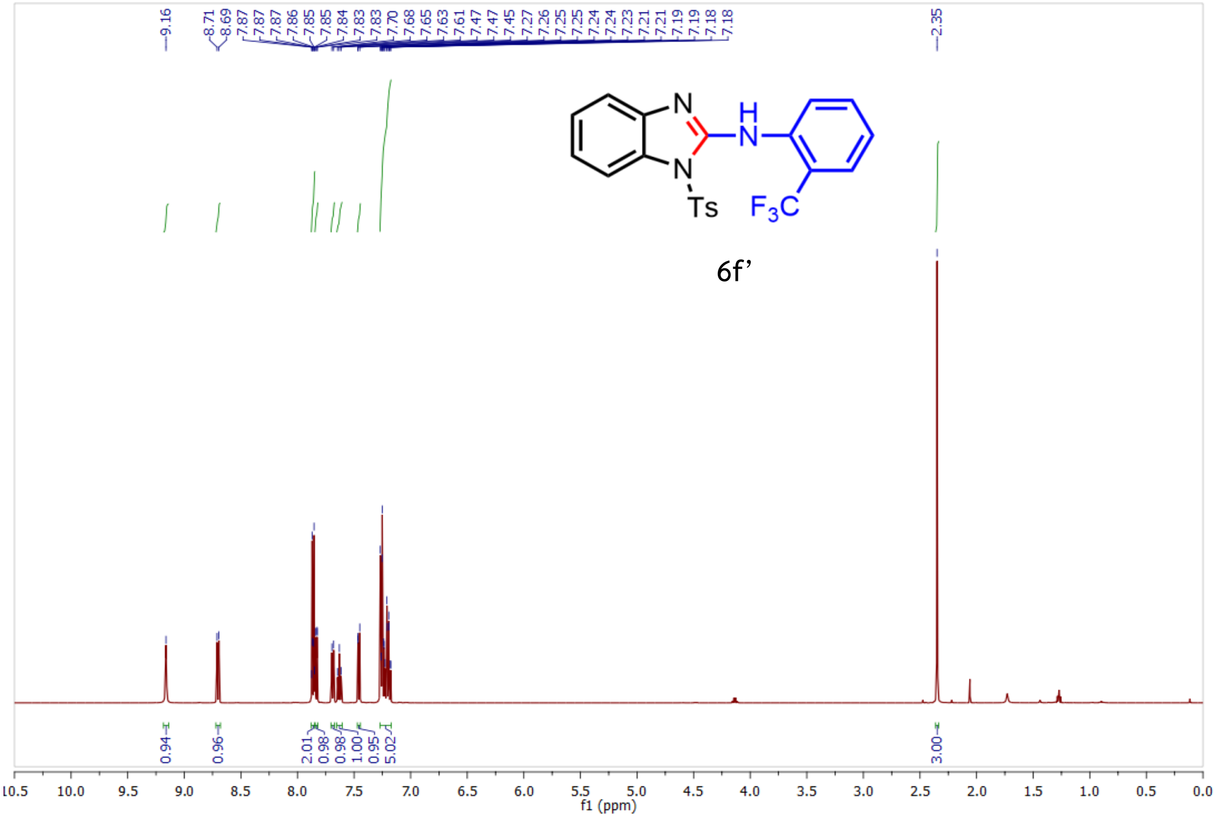


**Figure S100** $^{1}$H-NMR spectrum of **6f'** ($CDCl_3$, 500 MHz)

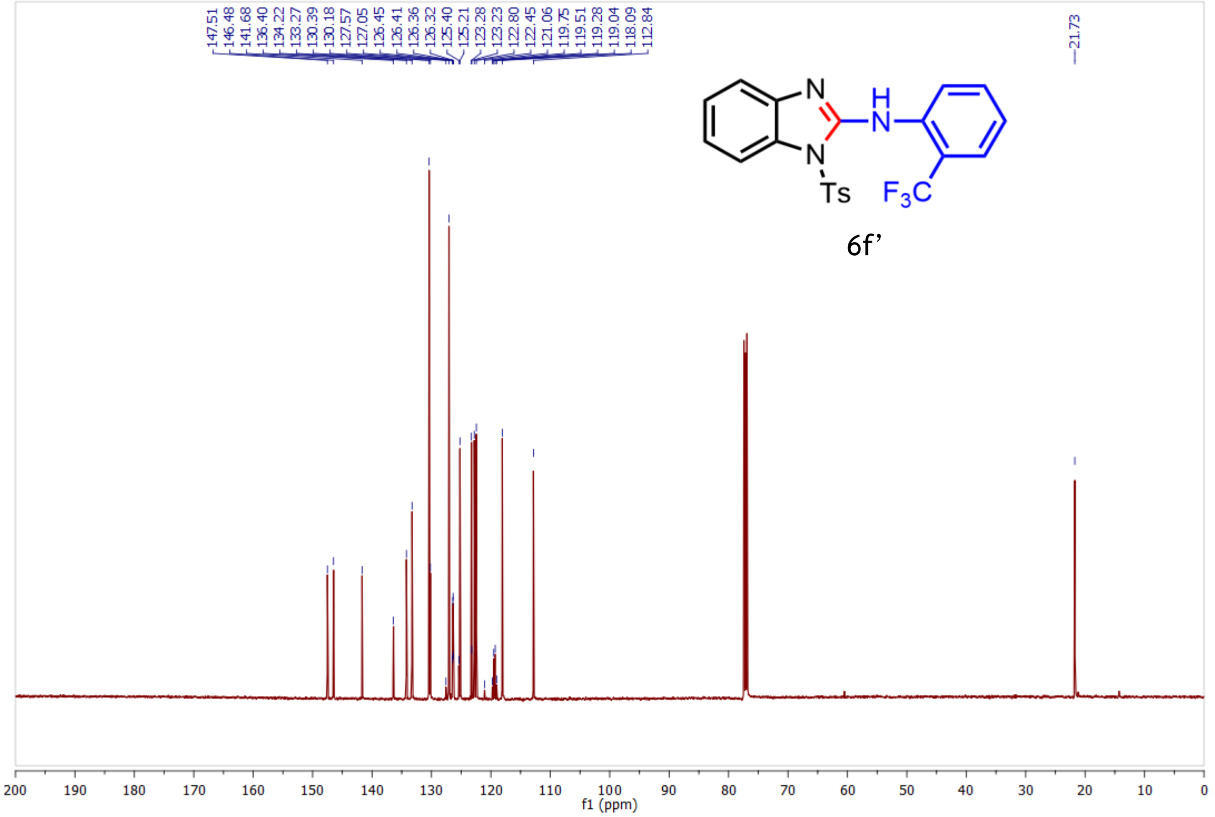


**Figure S101** $^{13}$C-NMR spectrum of **6f'** ($CDCl_3$, 126 MHz)

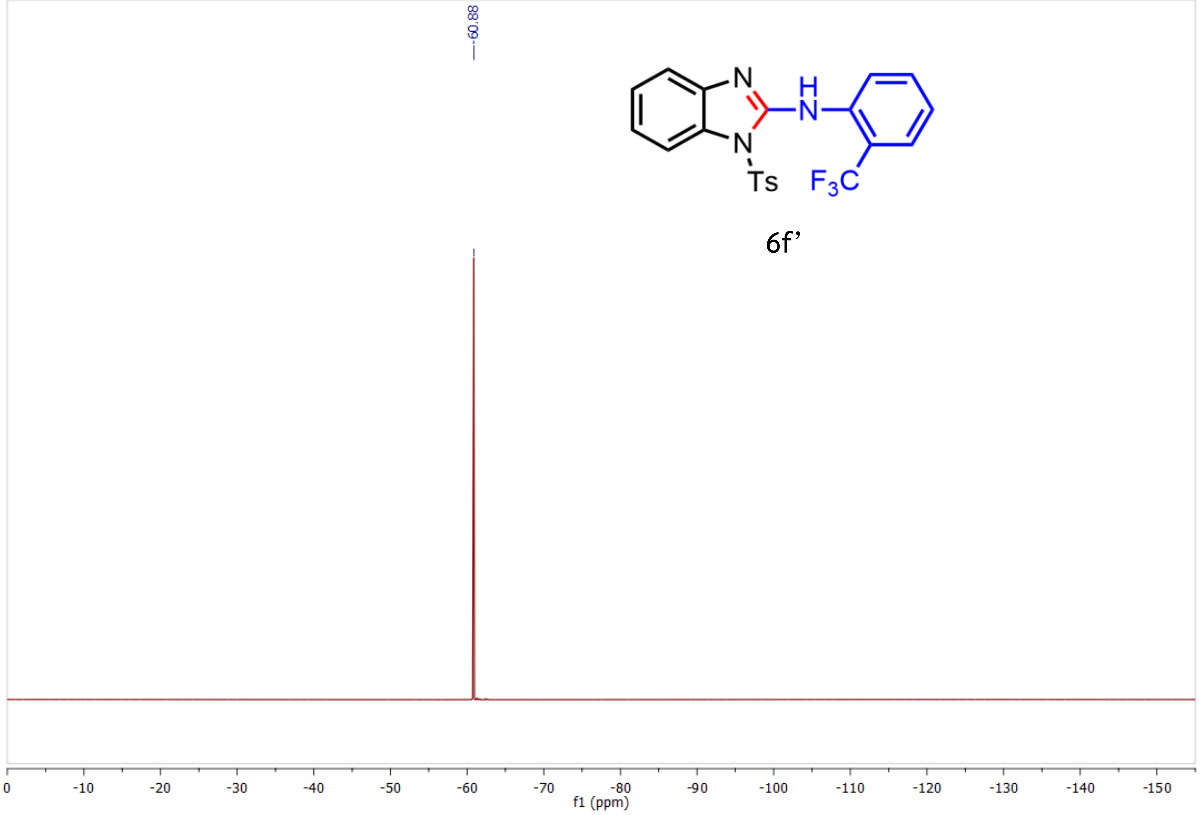


**Figure S102** $^{19}$F-NMR spectrum of **6f'** ($CDCl_3$, 471 MHz)

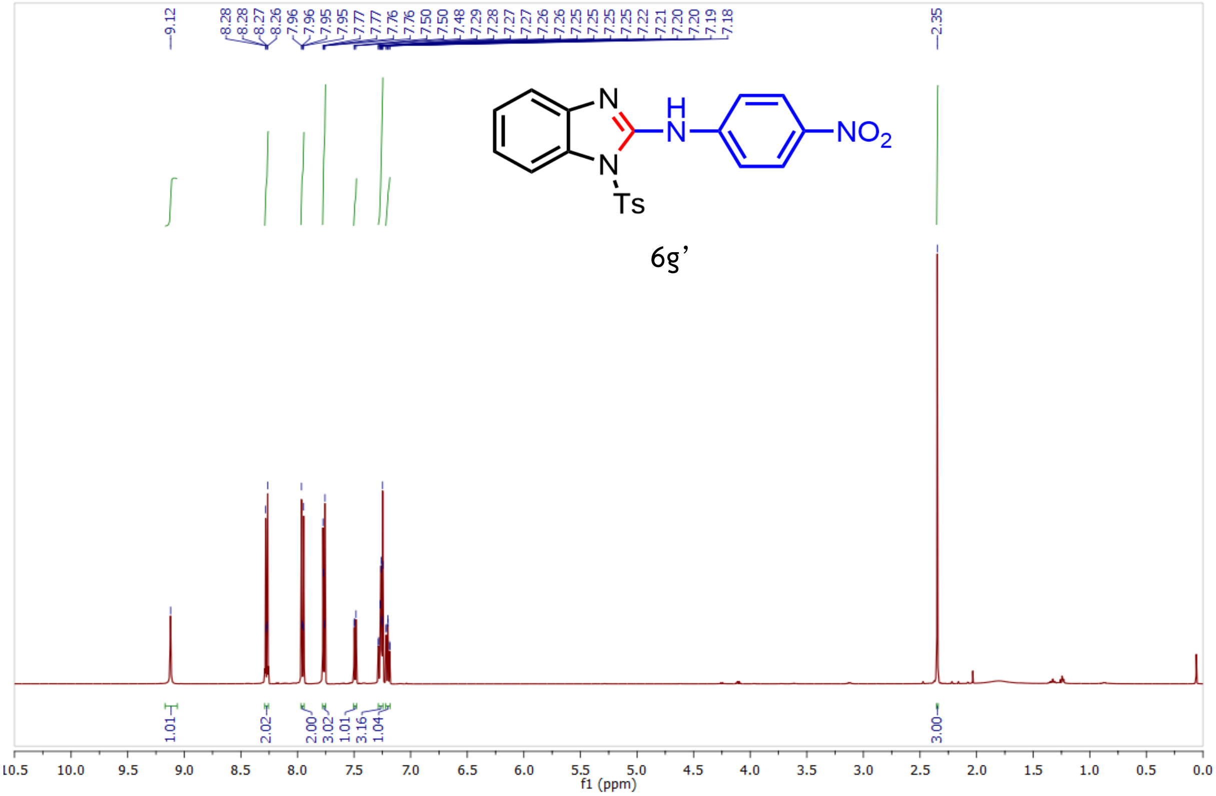


**Figure S103** $^{1}$H-NMR spectrum of **6g'** ($CDCl_3$, 500 MHz)

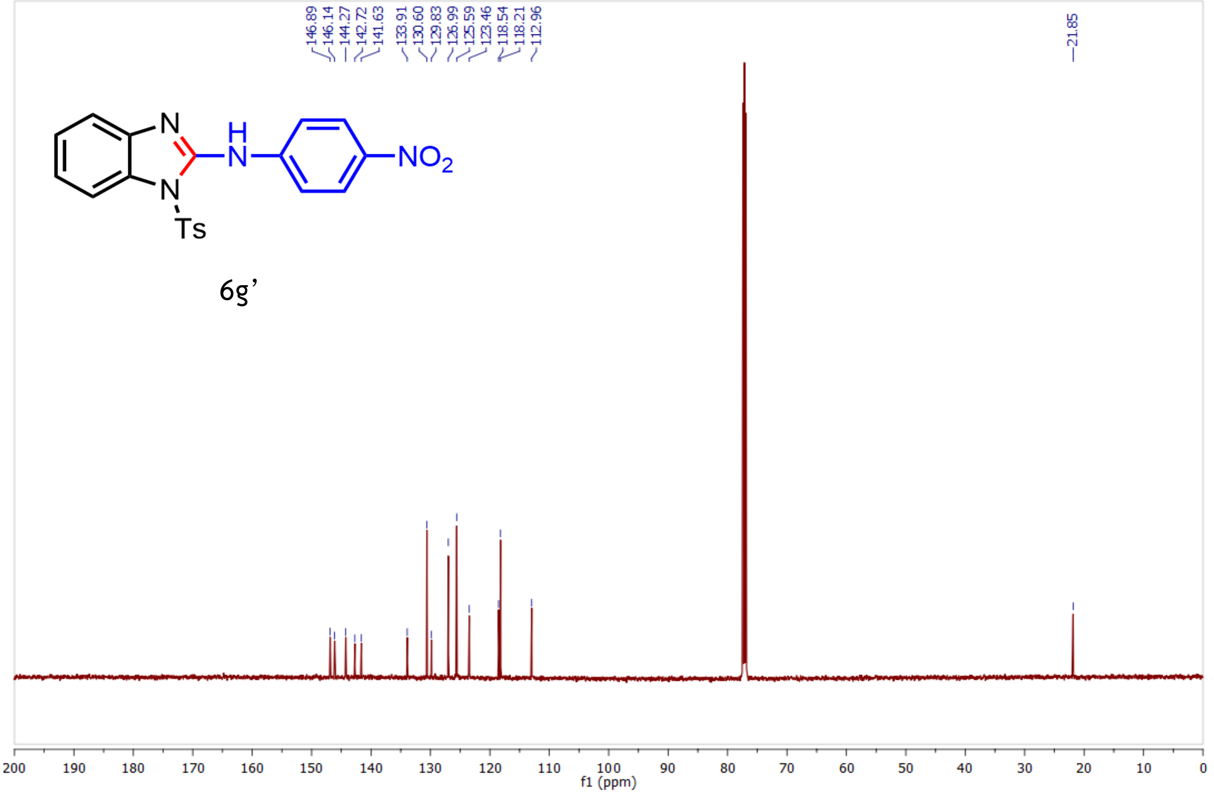


**Figure S104** $^{13}$C-NMR spectrum of **6g'** ($CDCl_3$, 126 MHz)

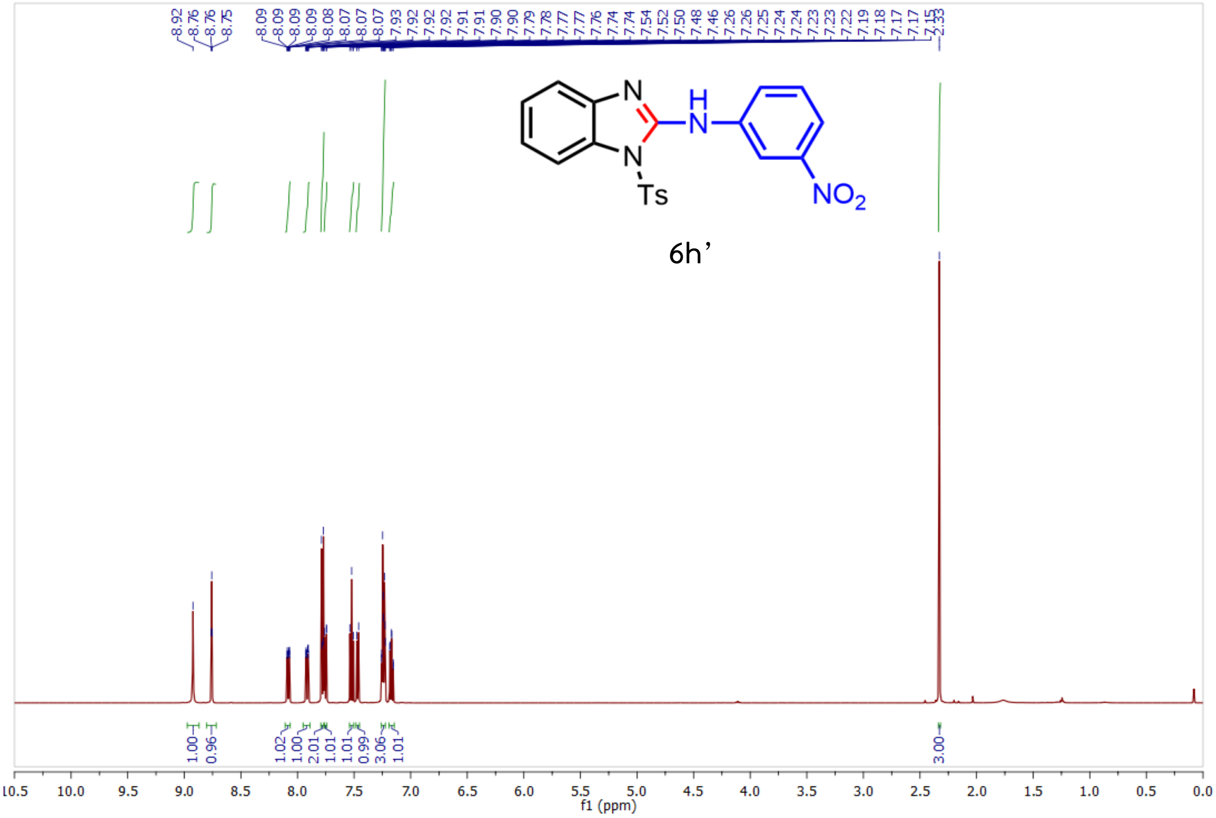

**Figure S105** $^{1}$H-NMR spectrum of **6h’** ($CDCl_3$, 500 MHz)

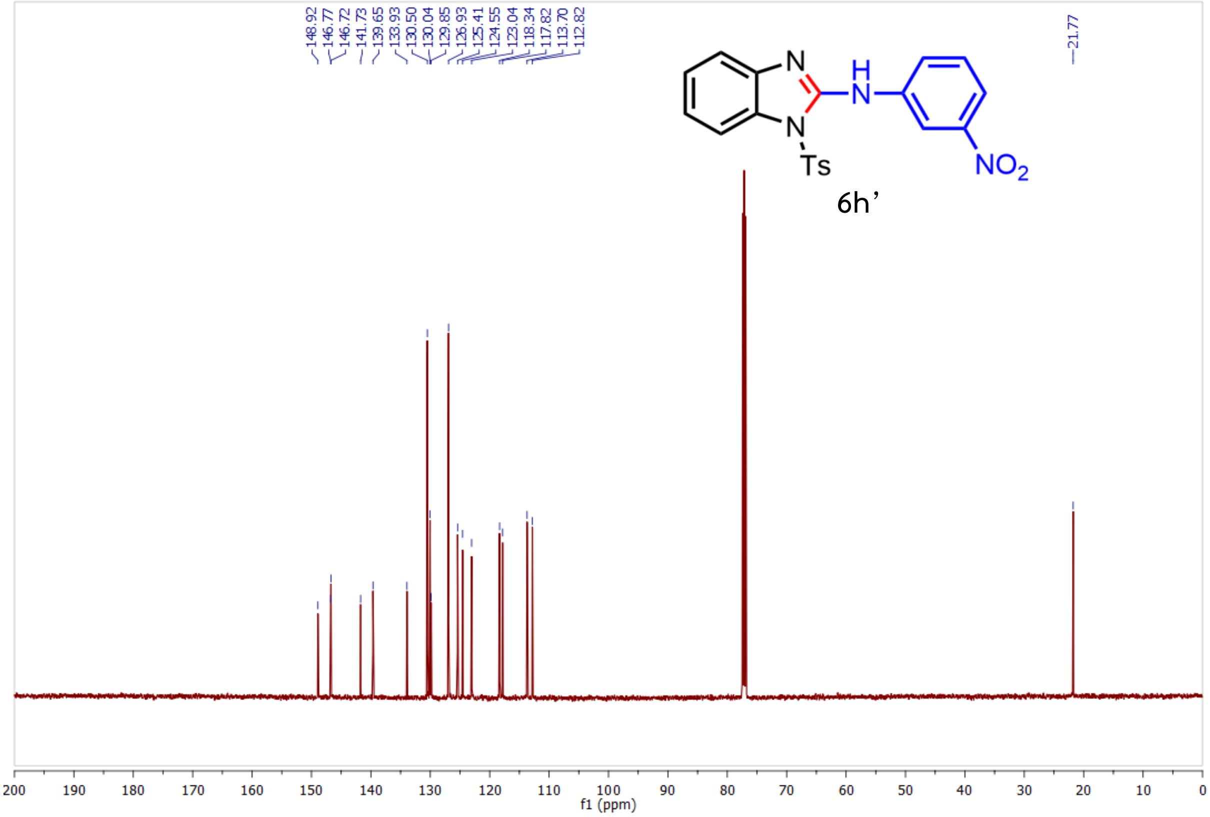

**Figure S106** $^{13}$C-NMR spectrum of **6h’** ($CDCl_3$, 126 MHz)

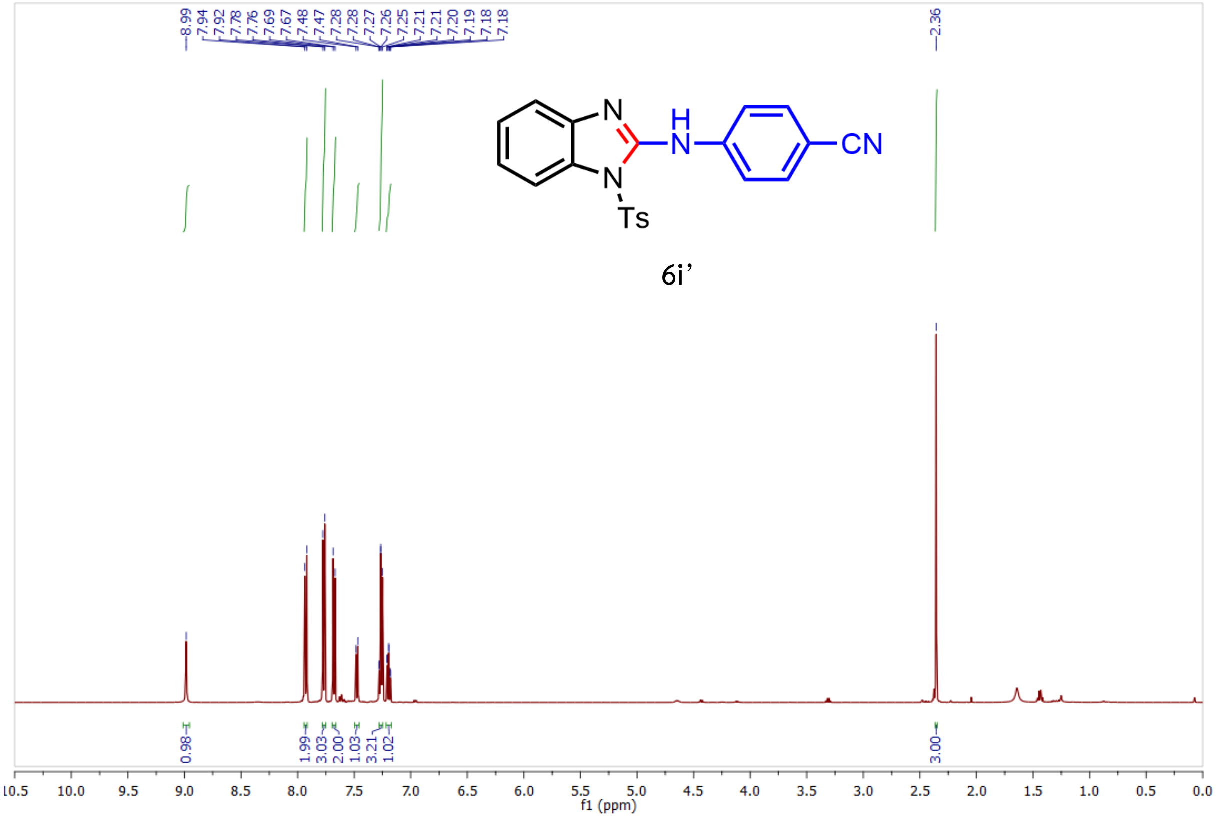


**Figure S107** $^{1}$H-NMR spectrum of **6i'** ($CDCl_3$, 500 MHz)

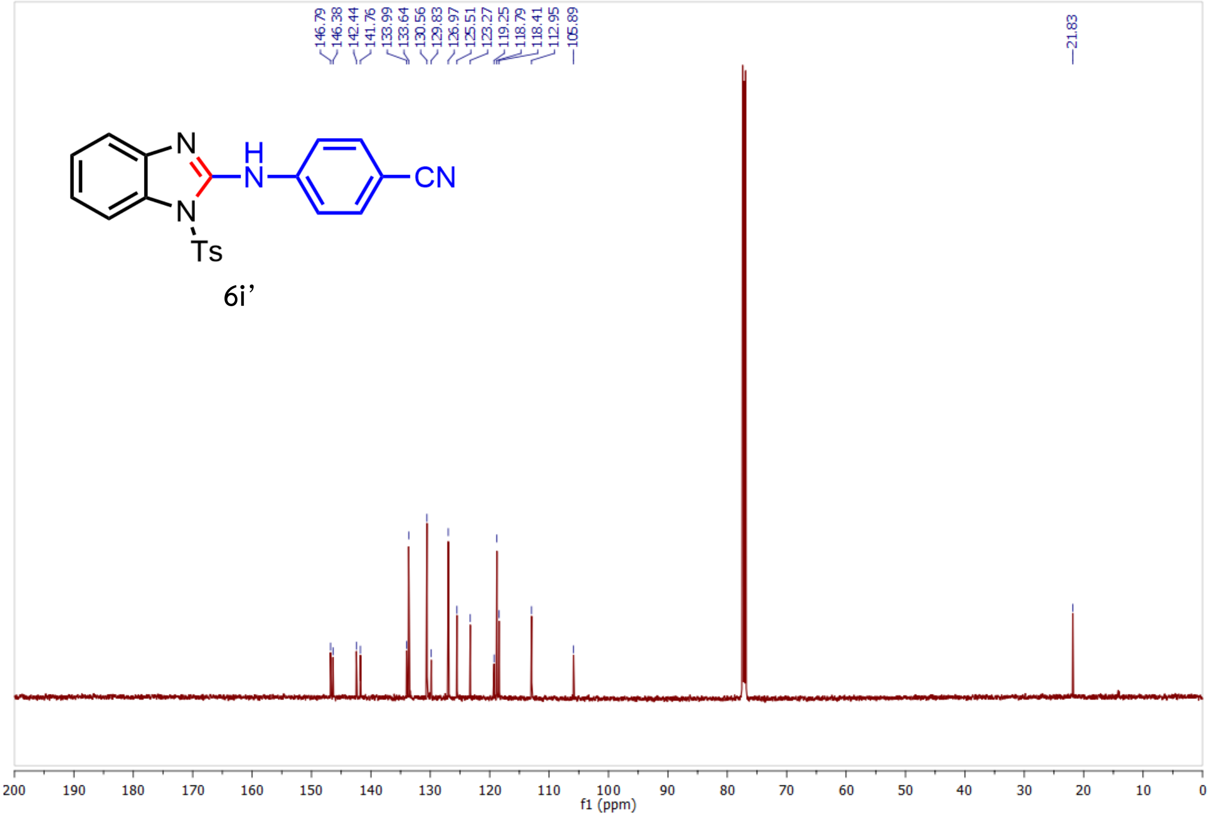


**Figure S108** $^{13}$C-NMR spectrum of **6i'** ($CDCl_3$, 126 MHz)

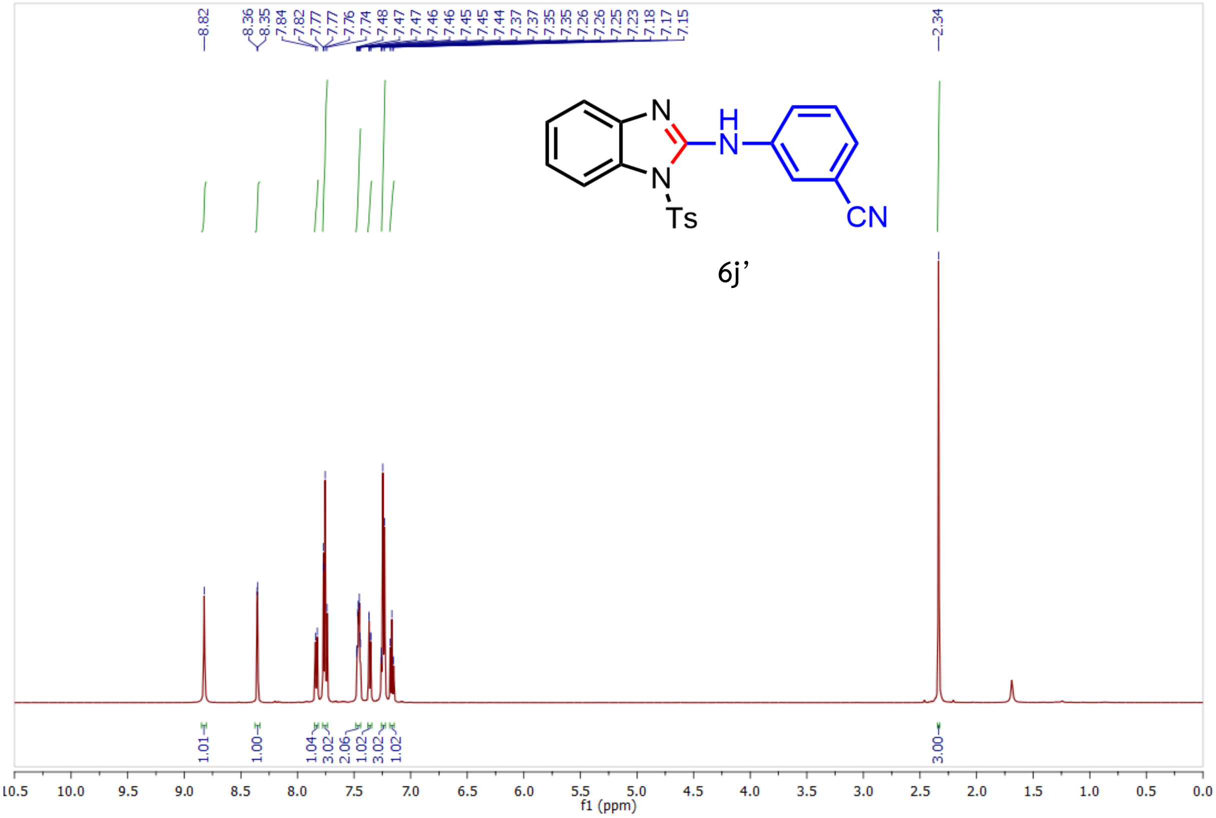


**Figure S109** $^{1}H$-NMR spectrum of **6j'** ($CDCl_3$, 500 MHz)

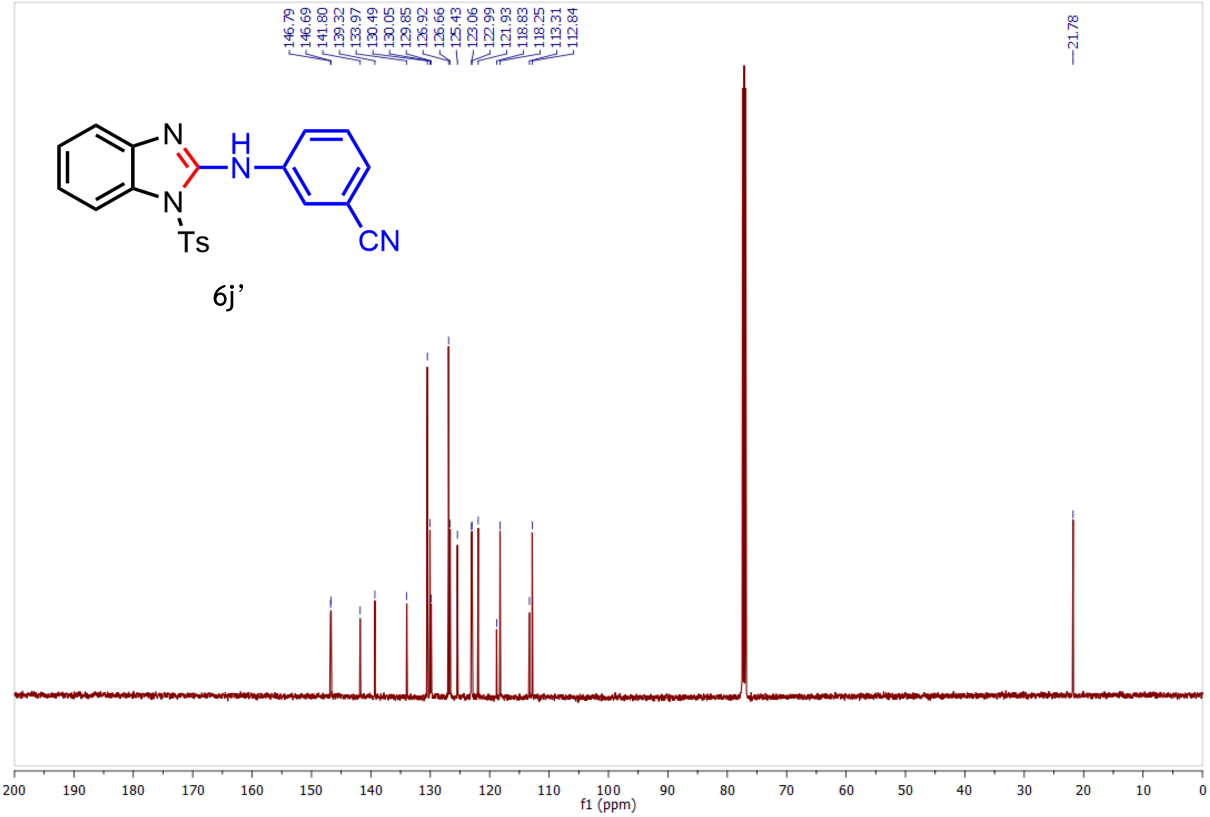


**Figure S110** $^{13}C$-NMR spectrum of **6j'** ($CDCl_3$, 126 MHz)

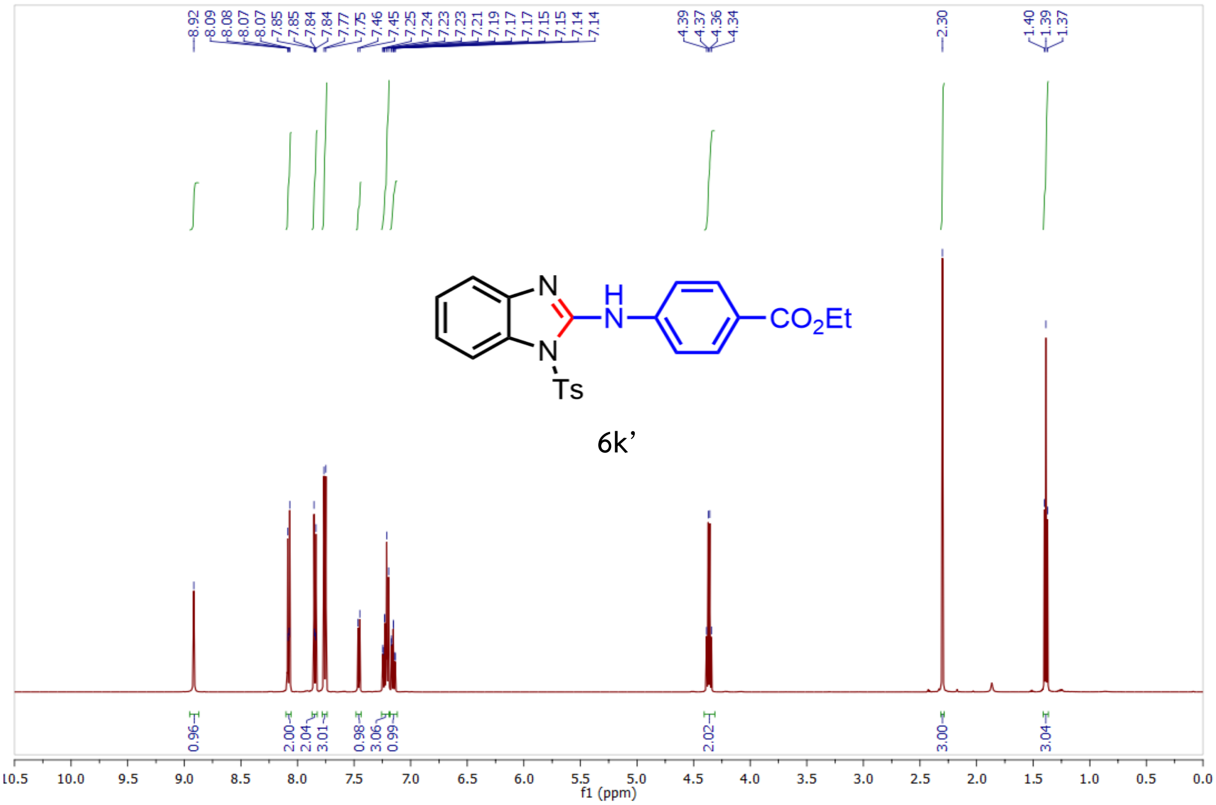


**Figure S111** $^{1}$H-NMR spectrum of **6k'** ($CDCl_3$, 500 MHz)

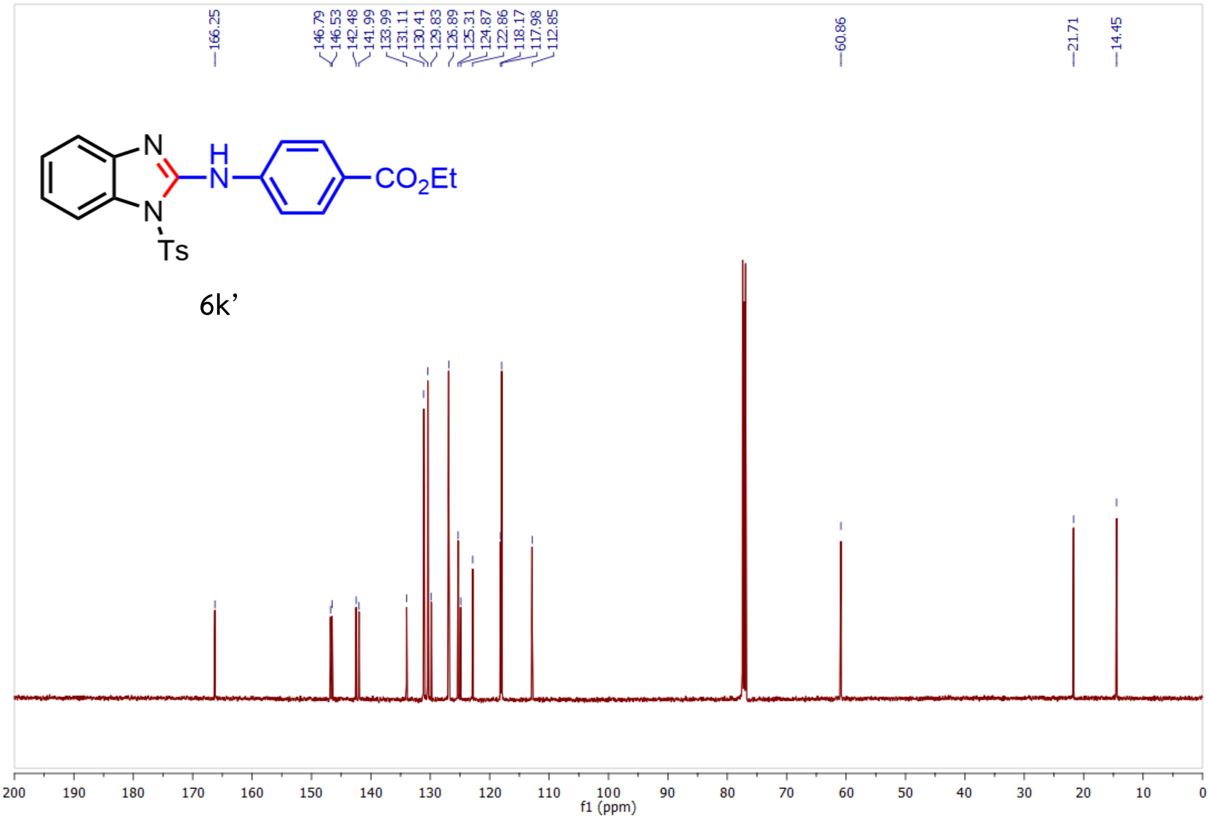


**Figure S112** $^{13}$C-NMR spectrum of **6k'** ($CDCl_3$, 126 MHz)

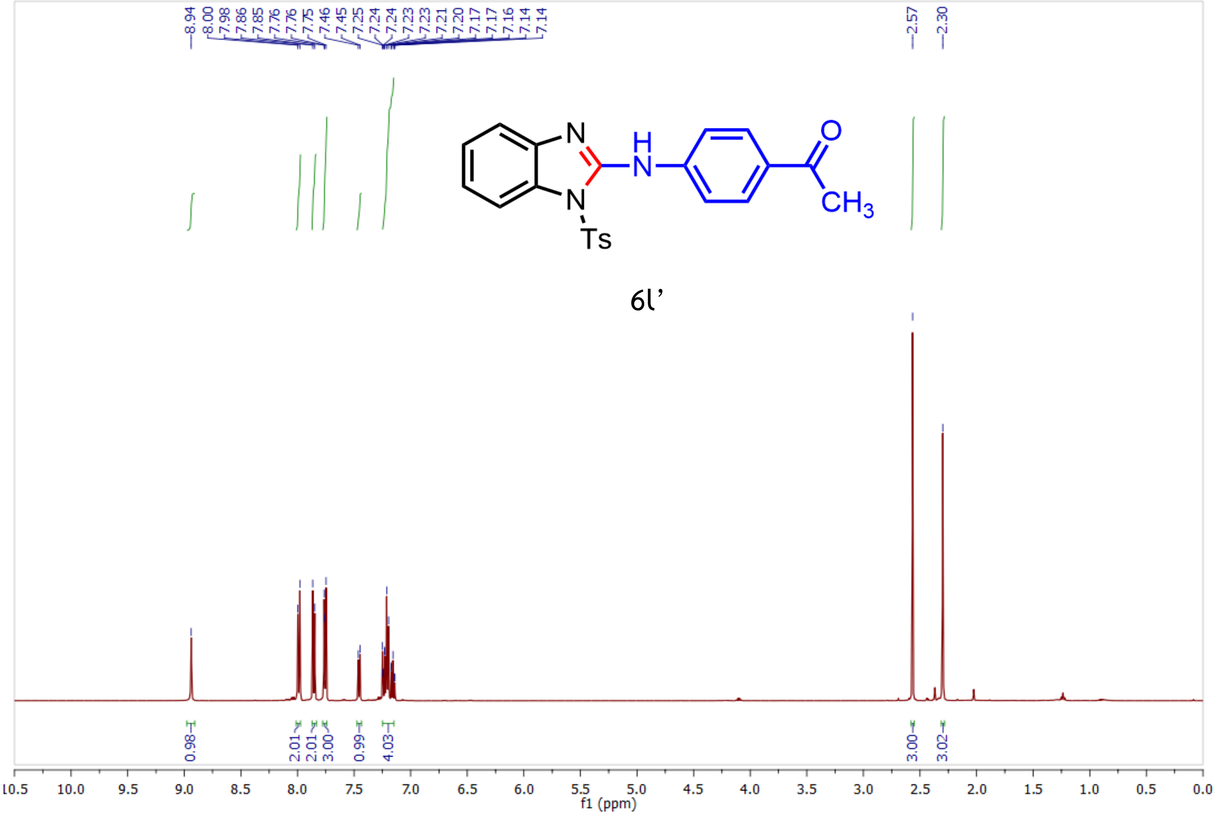


**Figure S113** 1H-NMR spectrum of **6l'** ($CDCl_3$, 500 MHz)

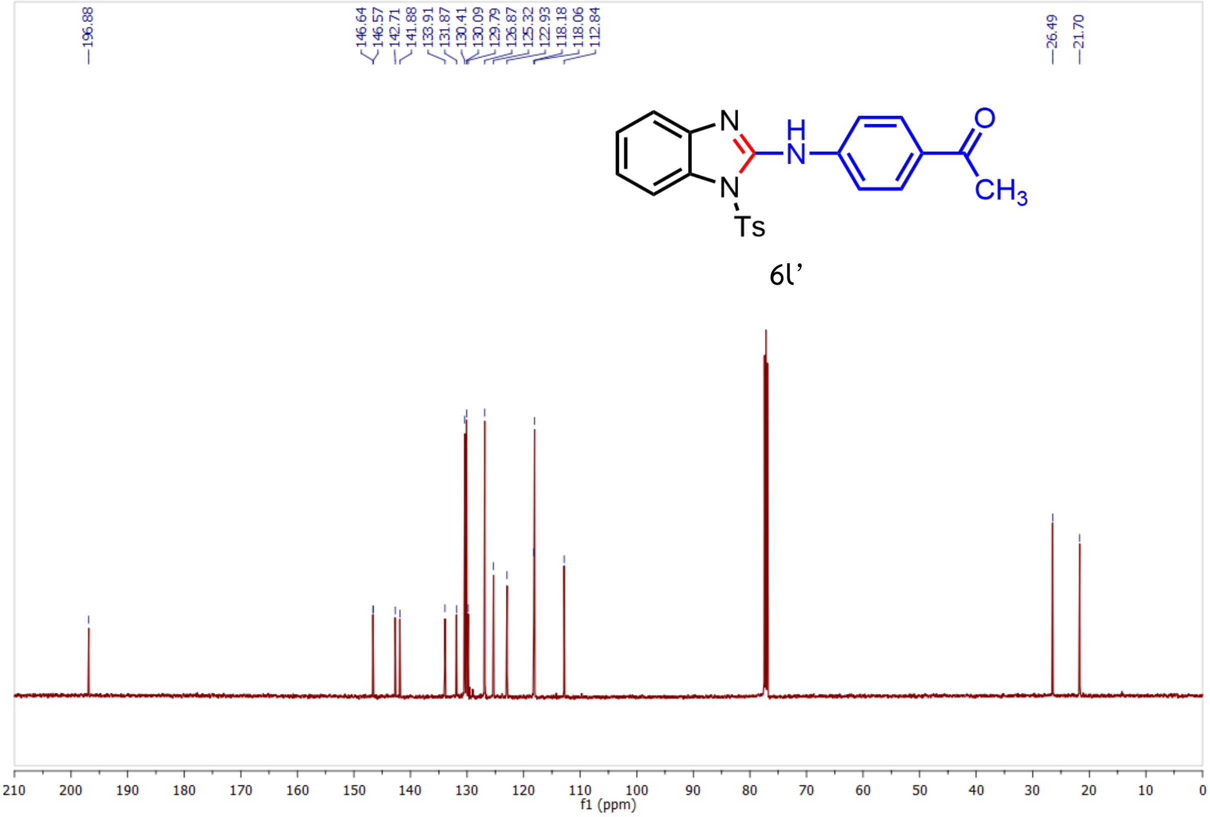


**Figure S114** 13C-NMR spectrum of **6l'** ($CDCl_3$, 126 MHz)

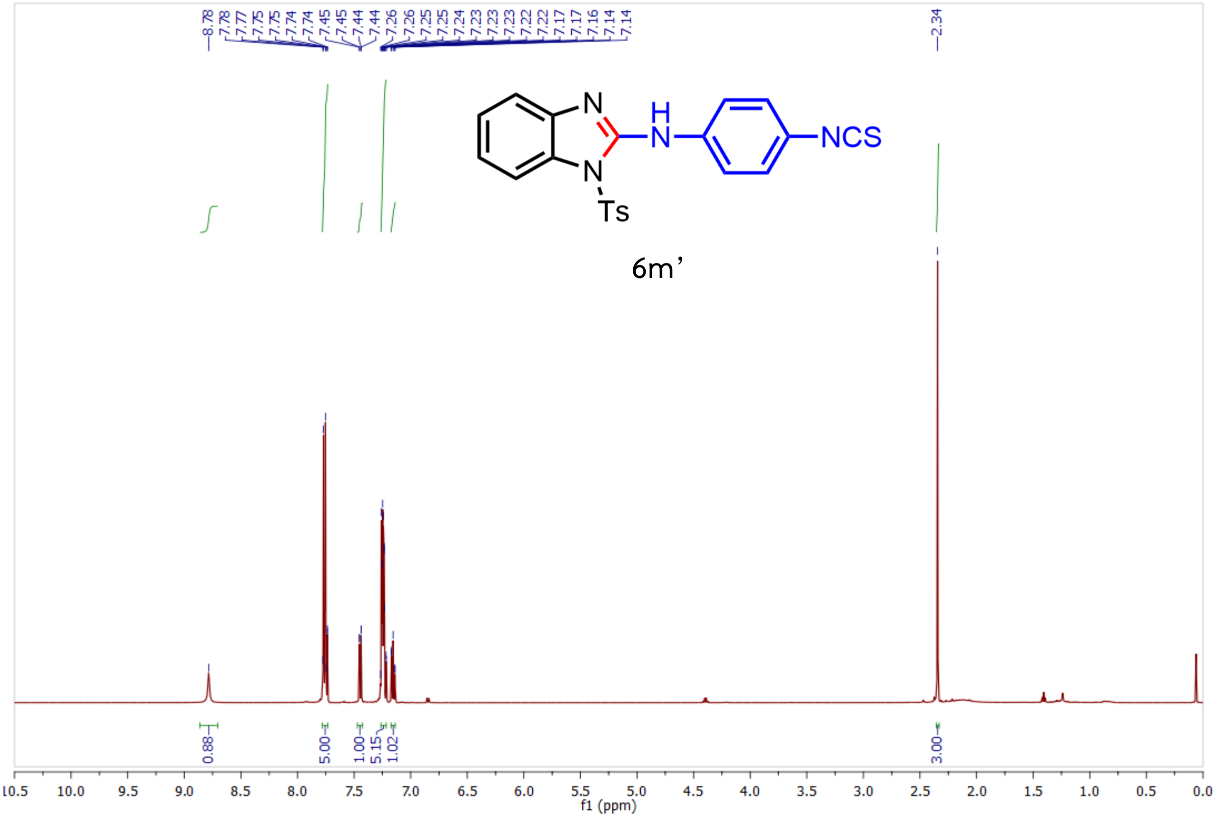

**Figure S115** $^{1}$H-NMR spectrum of **6m’** ($CDCl_3$, 500 MHz)

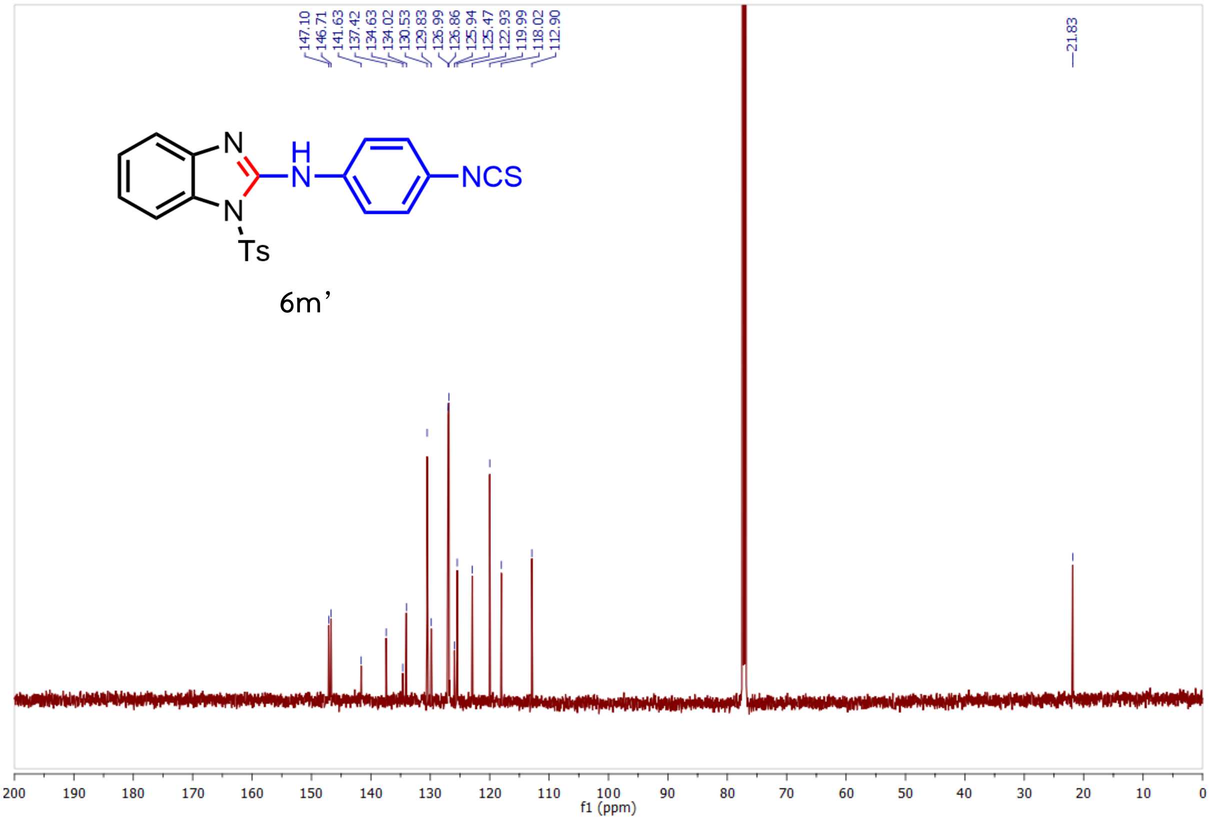

**Figure S116** $^{13}$C-NMR spectrum of **6m’** ($CDCl_3$, 126 MHz)

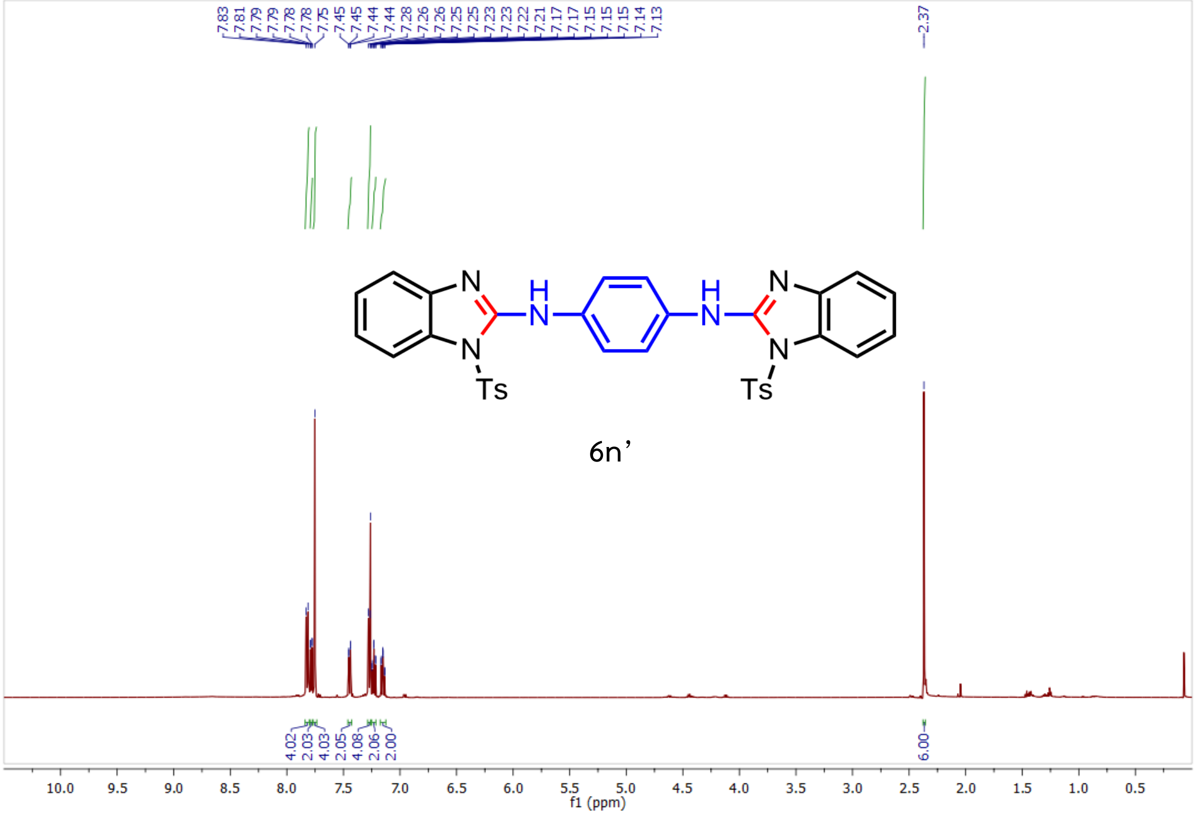


**Figure S117** $^{1}$H-NMR spectrum of **6n'** (CDC$_3$, 500 MHz)

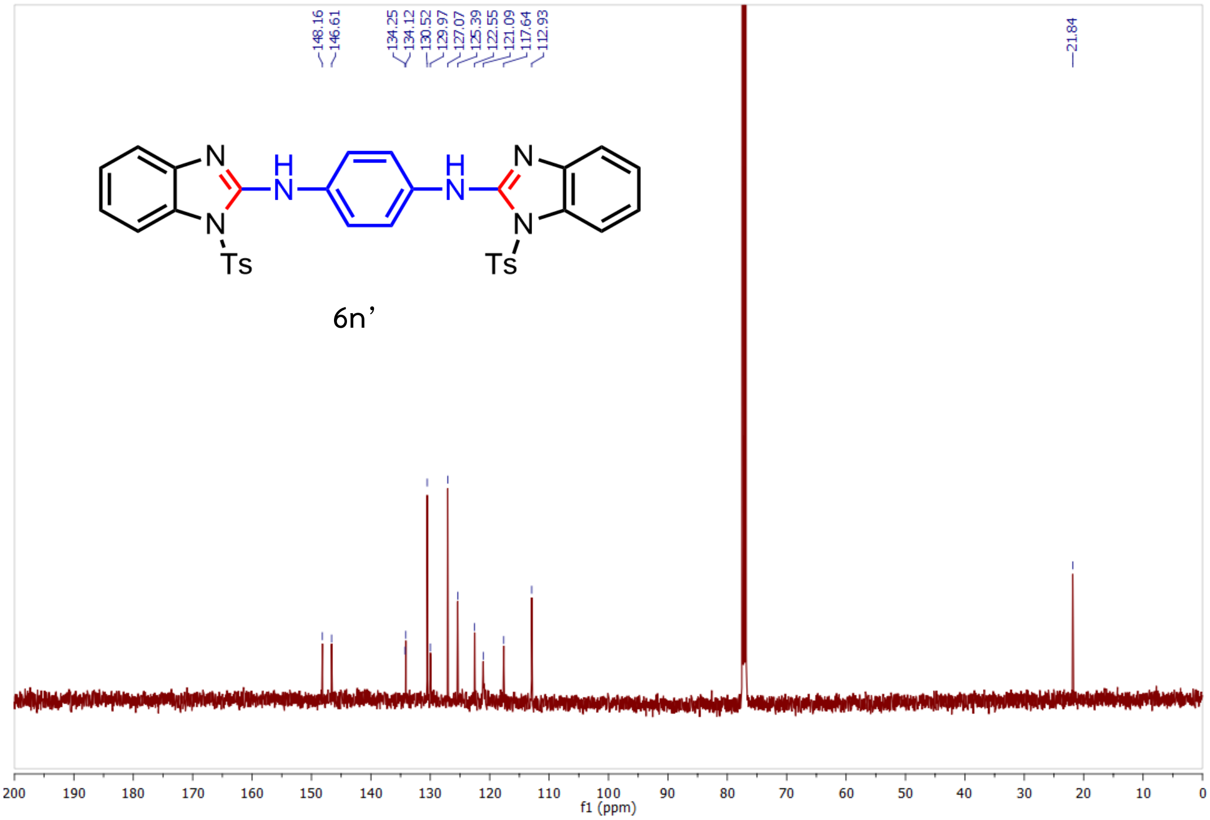


**Figure S118** $^{13}$C-NMR spectrum of **6n'** (CDCl$_3$, 126 MHz)

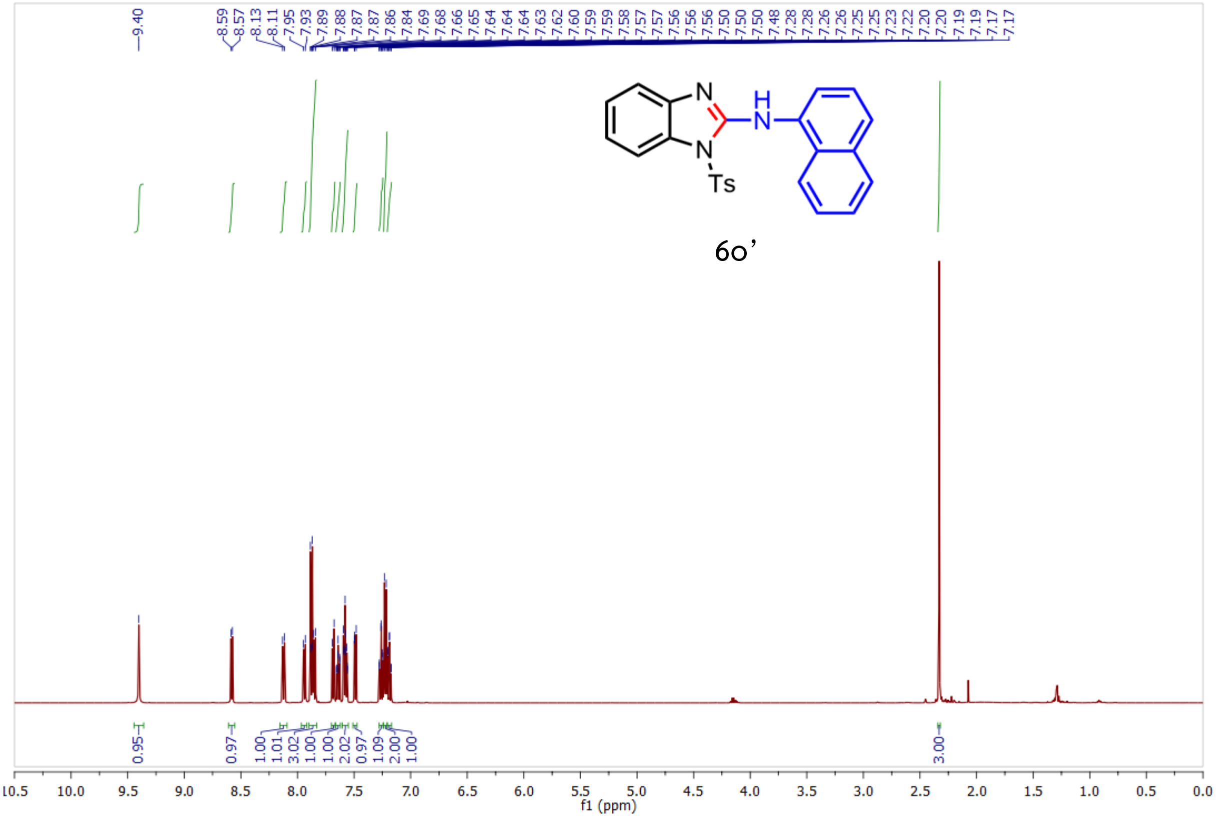


**Figure S119** $^{1}$H-NMR spectrum of **6o'** ($CDCl_3$, 500 MHz)

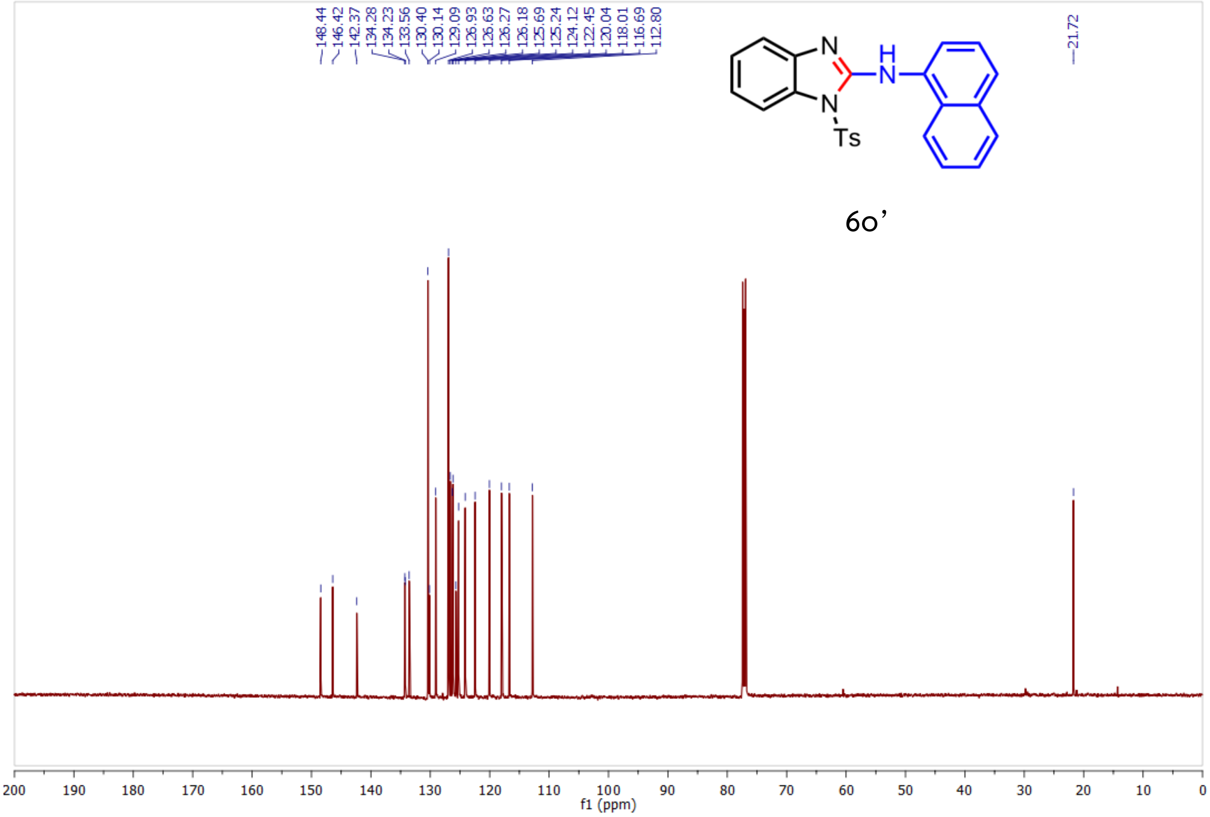


**Figure S120** $^{13}$C-NMR spectrum of **6o'** ($CDCl_3$, 126 MHz)

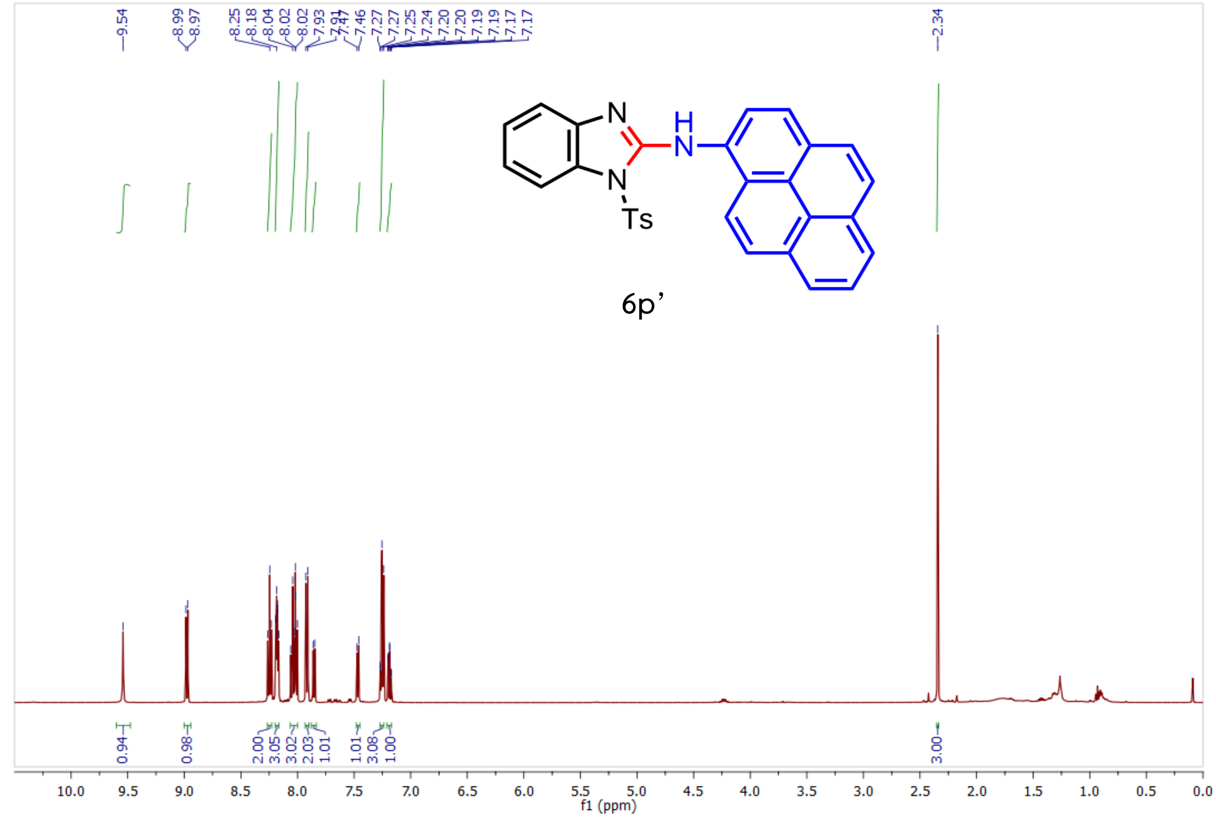


**Figure S121** $^{1}$H-NMR spectrum of **6p'** ($CDCl_3$, 500 MHz)

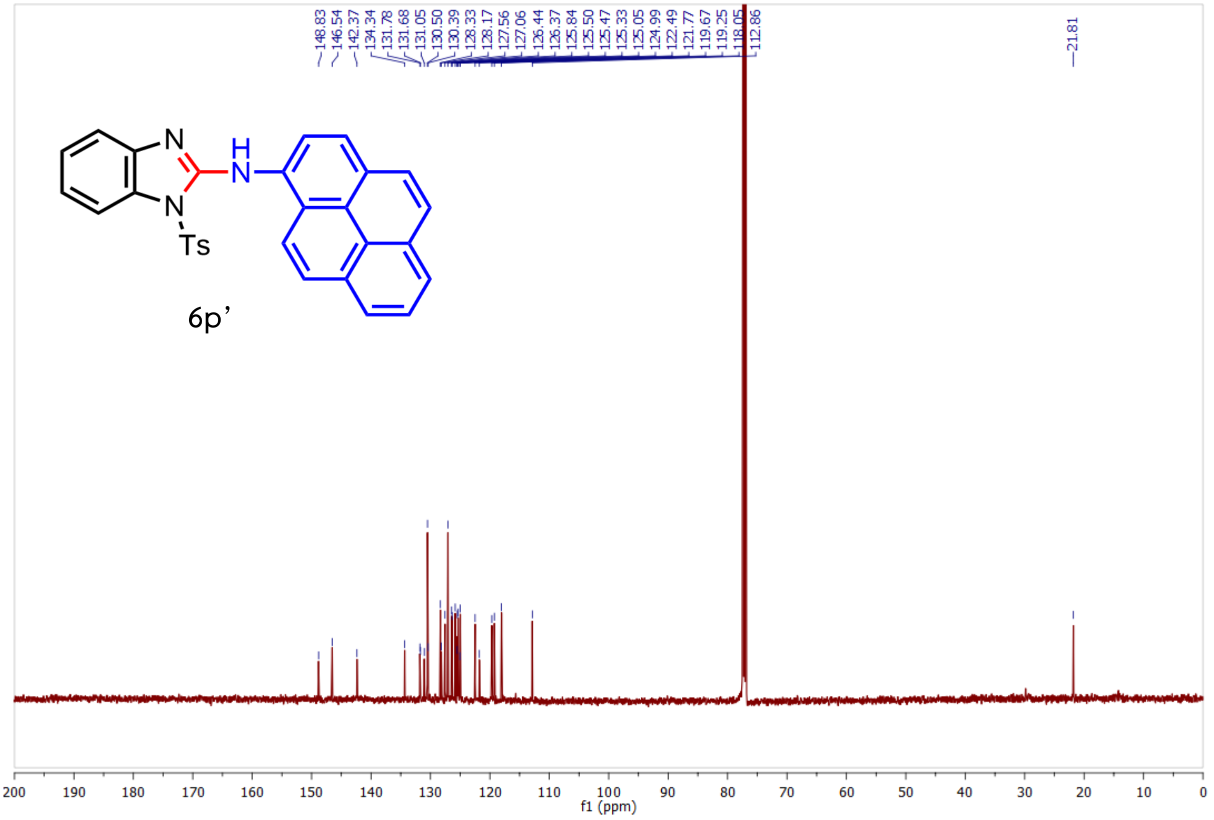


**Figure S122** $^{13}$C-NMR spectrum of **6p'** ($CDCl_3$, 126 MHz)

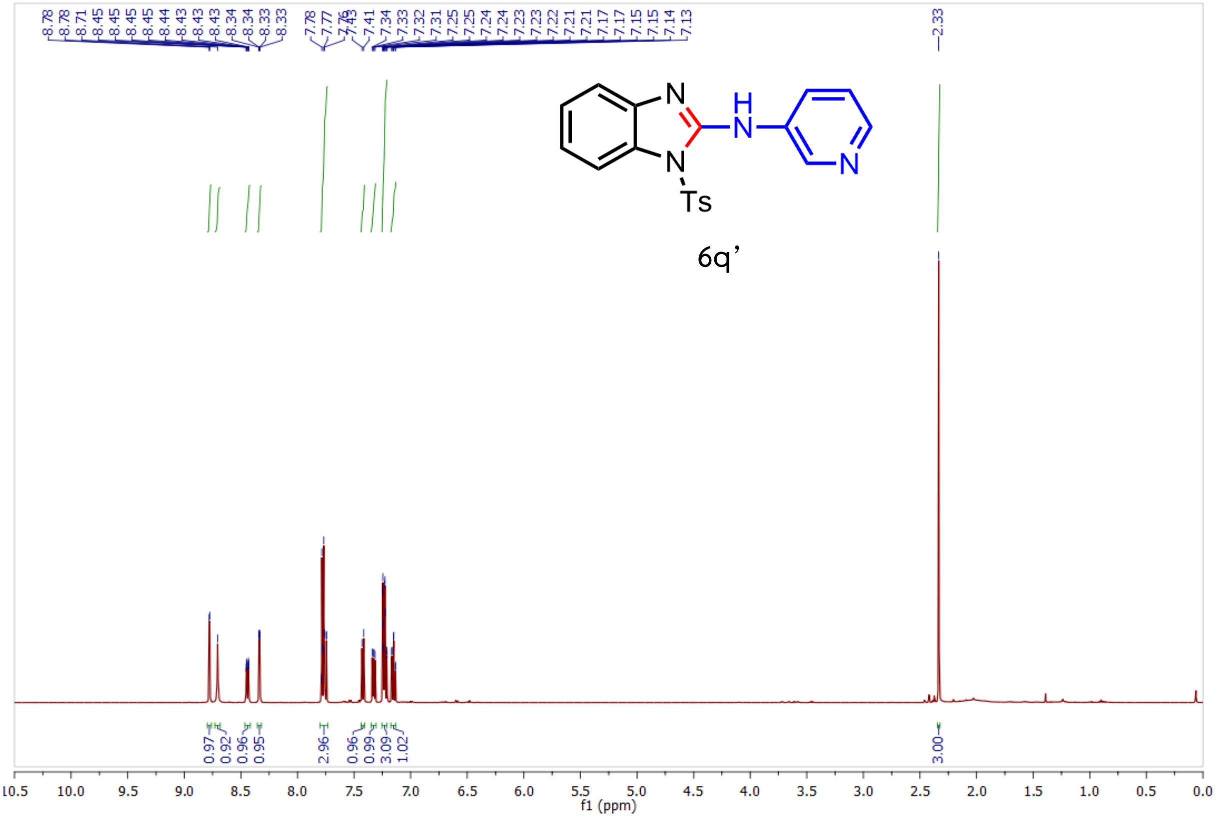


**Figure S123** $^{1}$H-NMR spectrum of **6q'** ($CDCl_3$, 500 MHz)

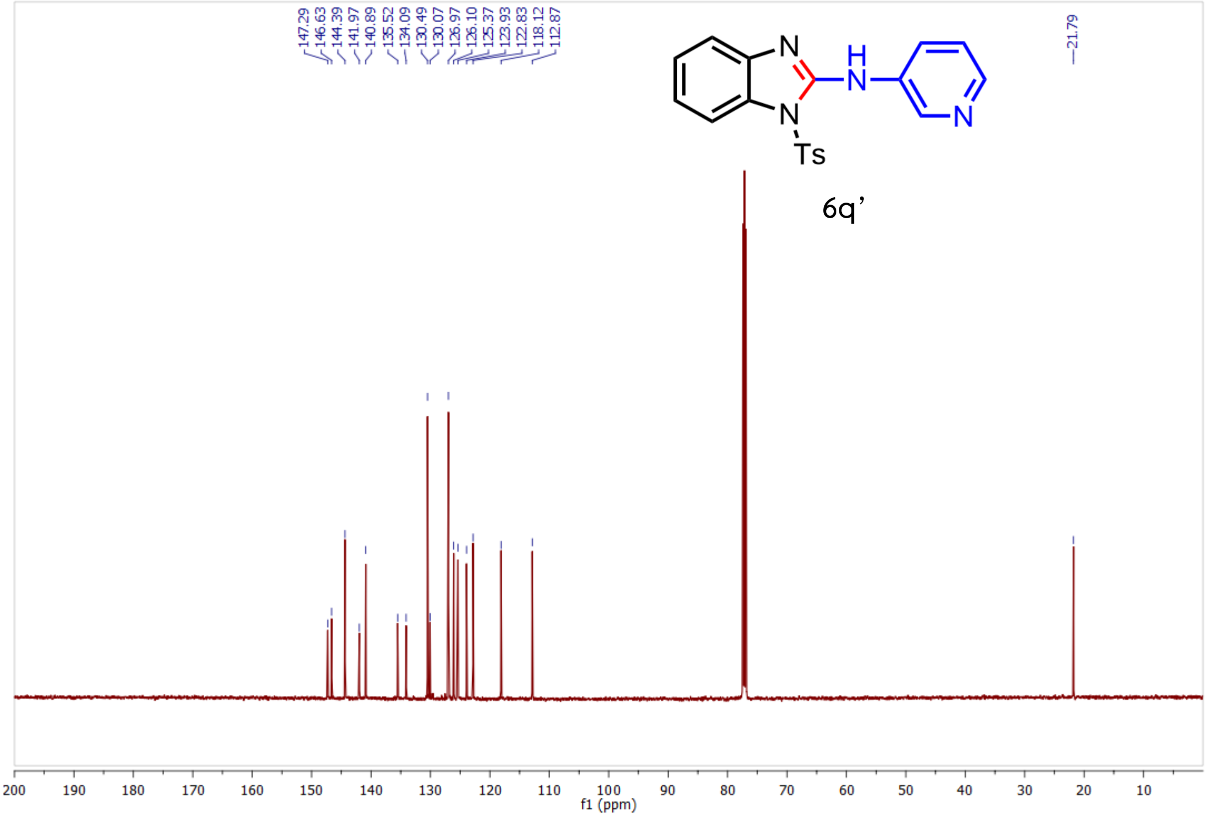


**Figure S124** $^{13}$C-NMR spectrum of **6q'** ($CDCl_3$, 126 MHz)

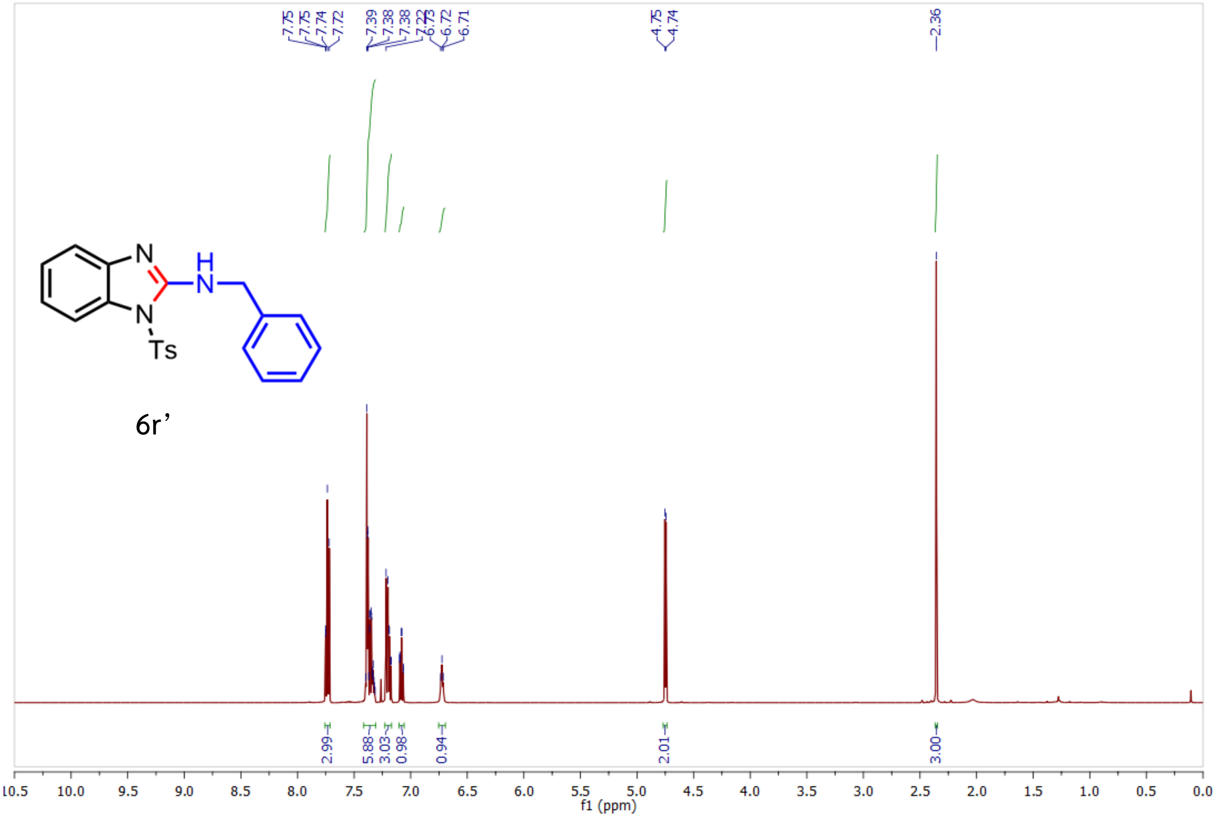

**Figure S125** $^{1}$H-NMR spectrum of **6r'** ($CDCl_3$, 500 MHz)

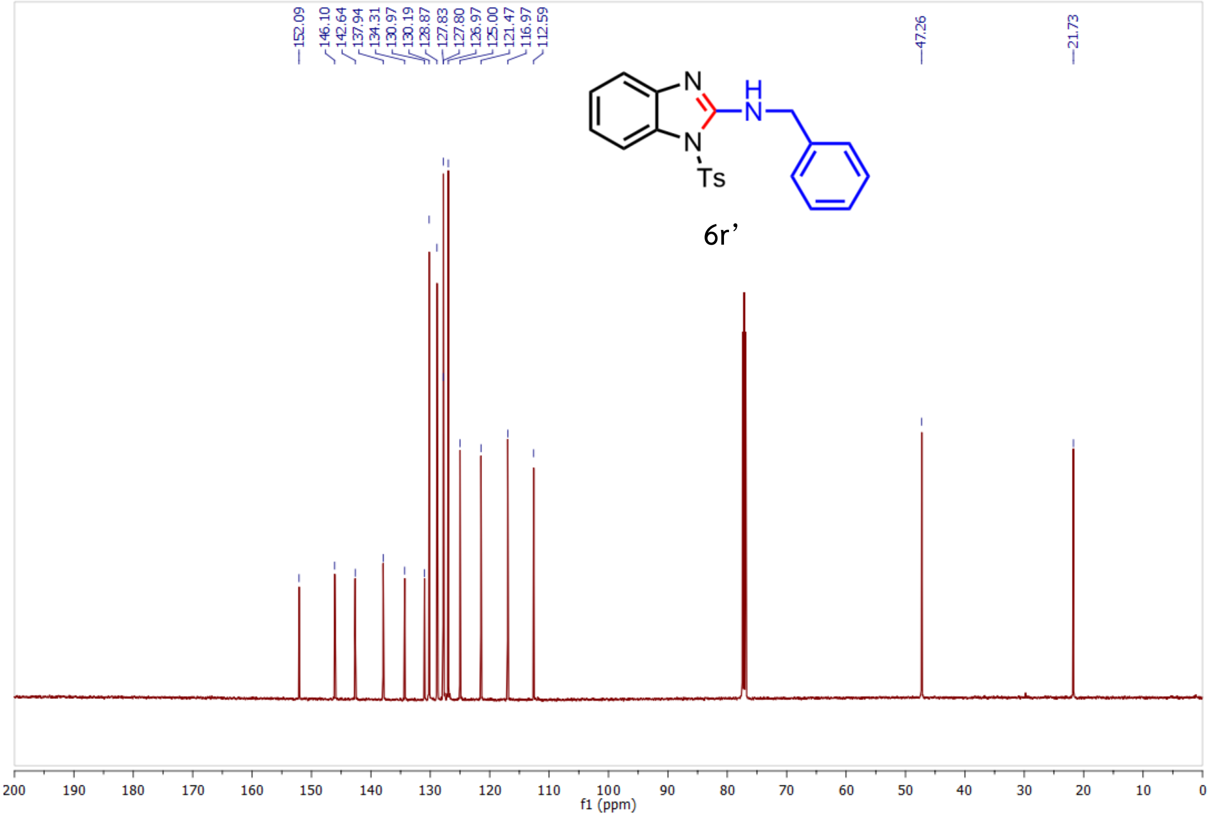

**Figure S126** $^{13}$C-NMR spectrum of **6r'** ($CDCl_3$, 126 MHz)

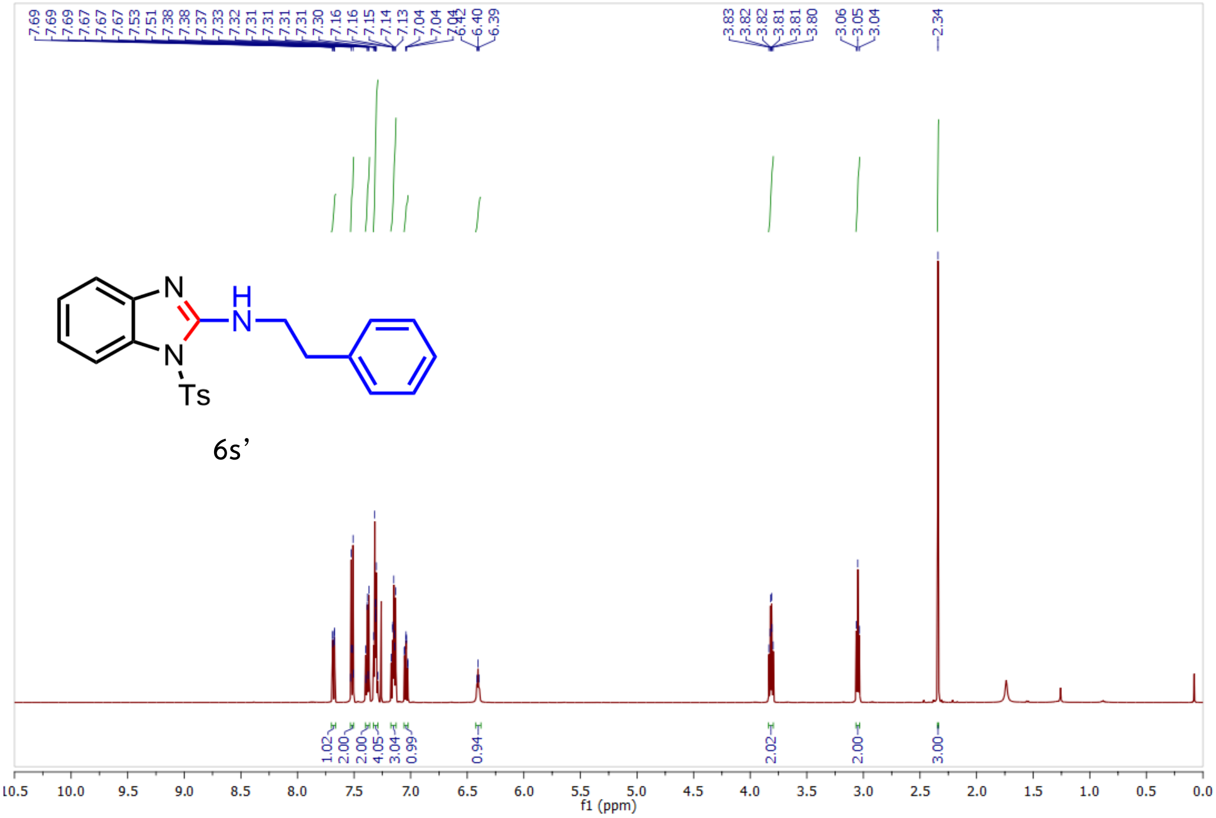

**Figure S127** $^{1}H$-NMR spectrum of **6s'** ($CDCl_3$, 500 MHz)

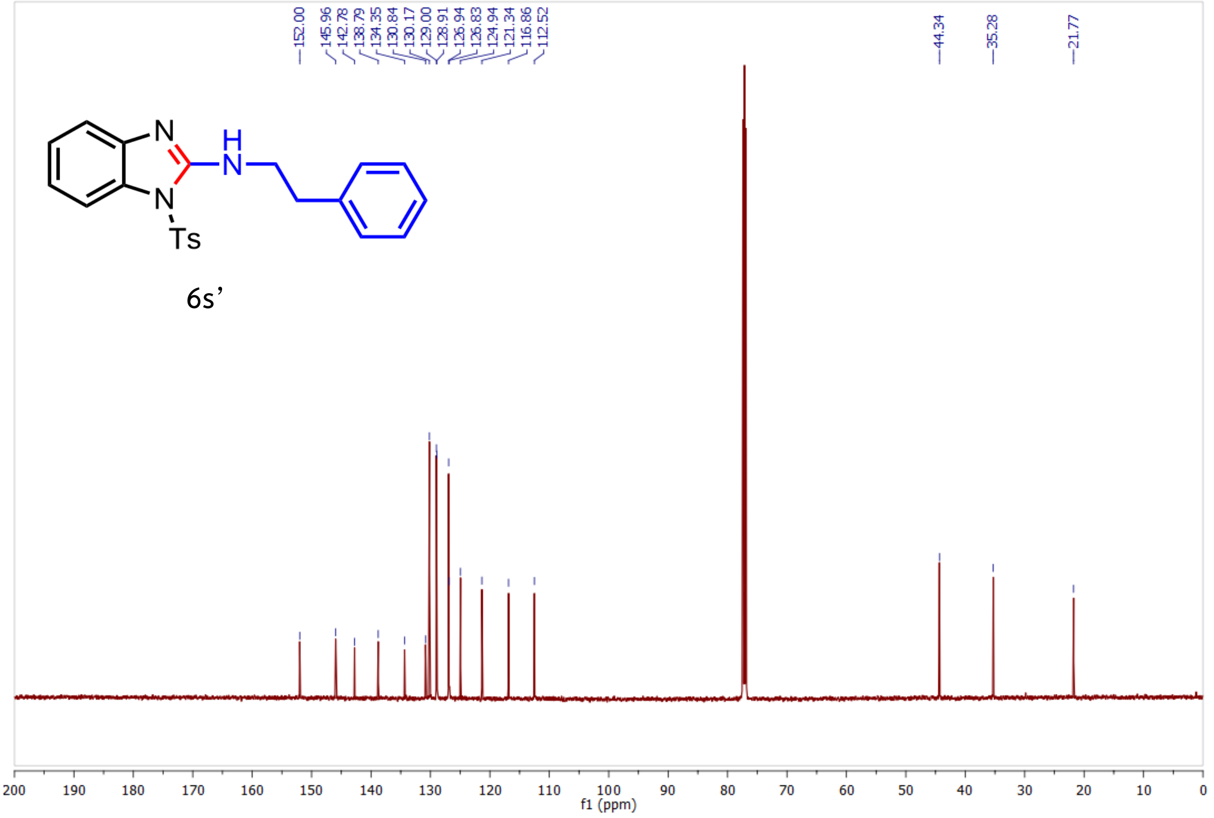

**Figure S128** $^{13}C$-NMR spectrum of **6s'** ($CDCl_3$, 126 MHz)

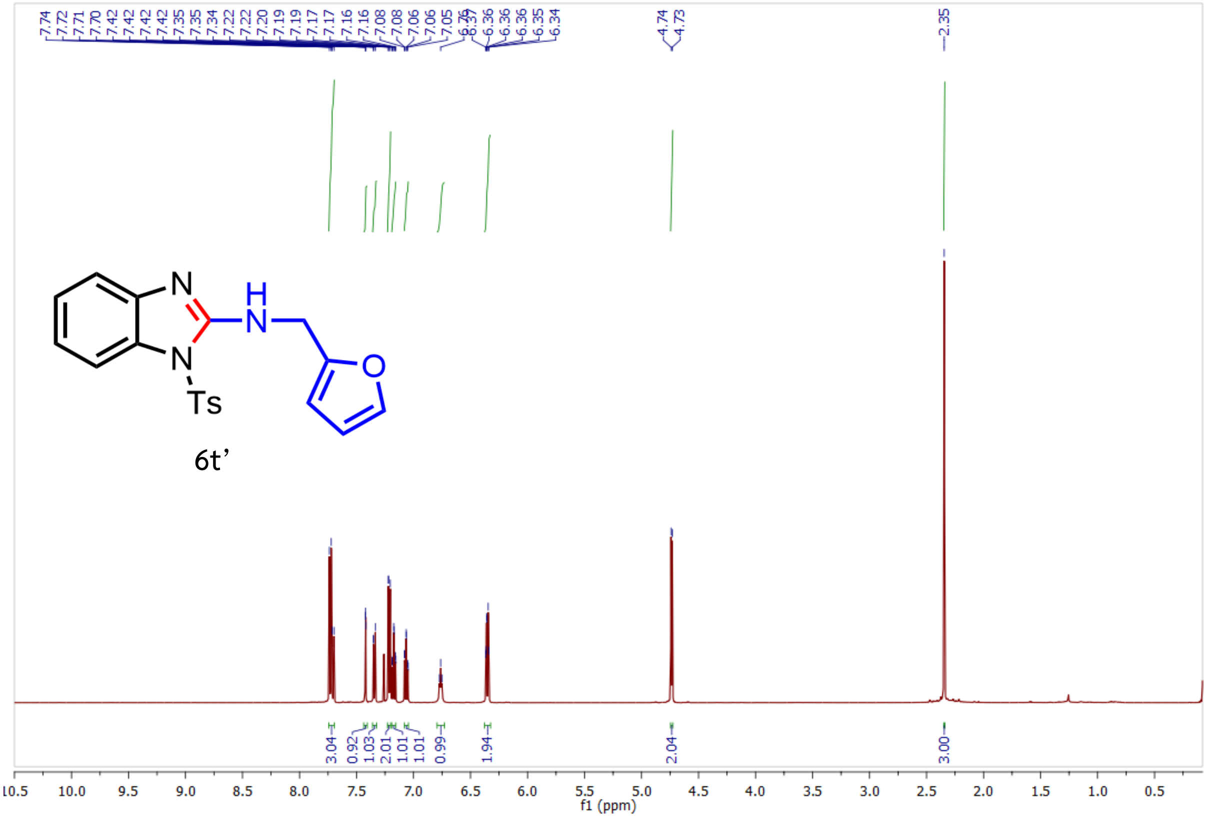

**Figure S129** $^{1}$H-NMR spectrum of **6t'** ($CDCl_3$, 500 MHz)

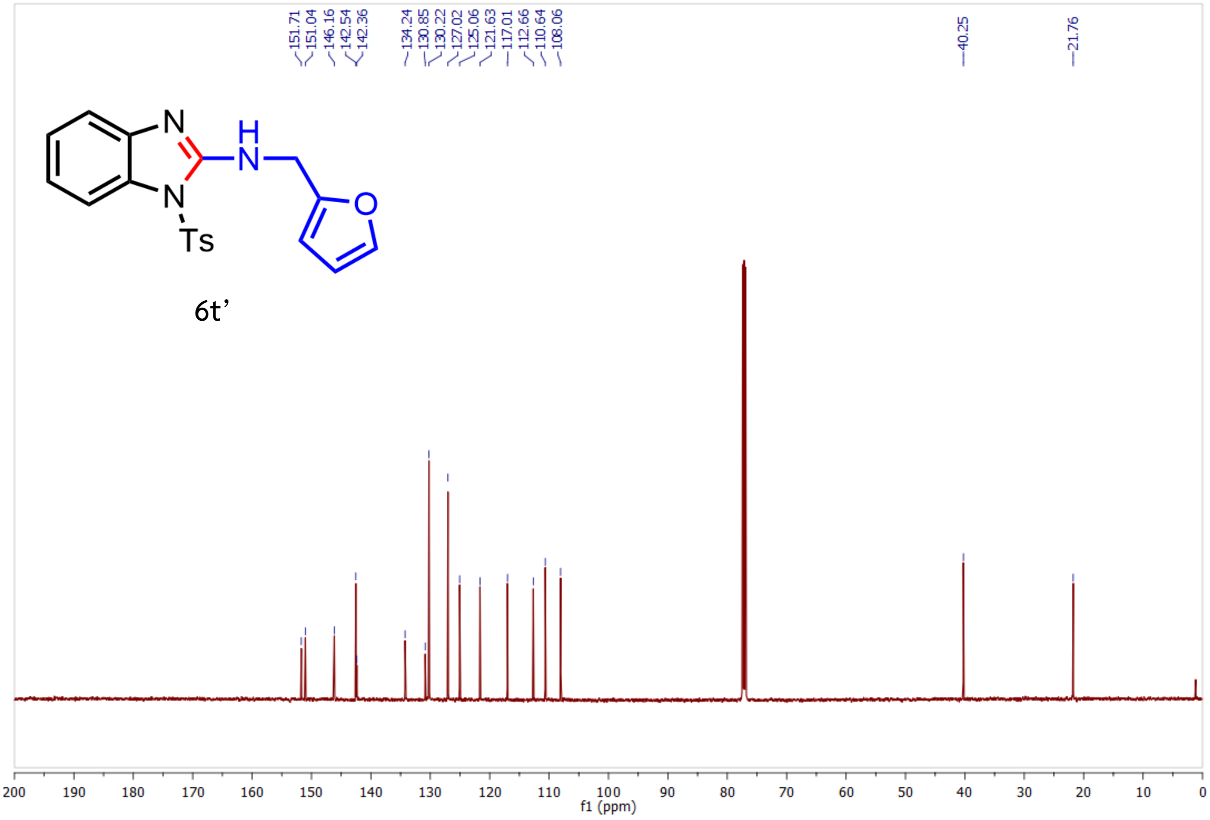

**Figure S130** $^{13}$C-NMR spectrum of **6t'** ($CDCl_3$, 126 MHz)

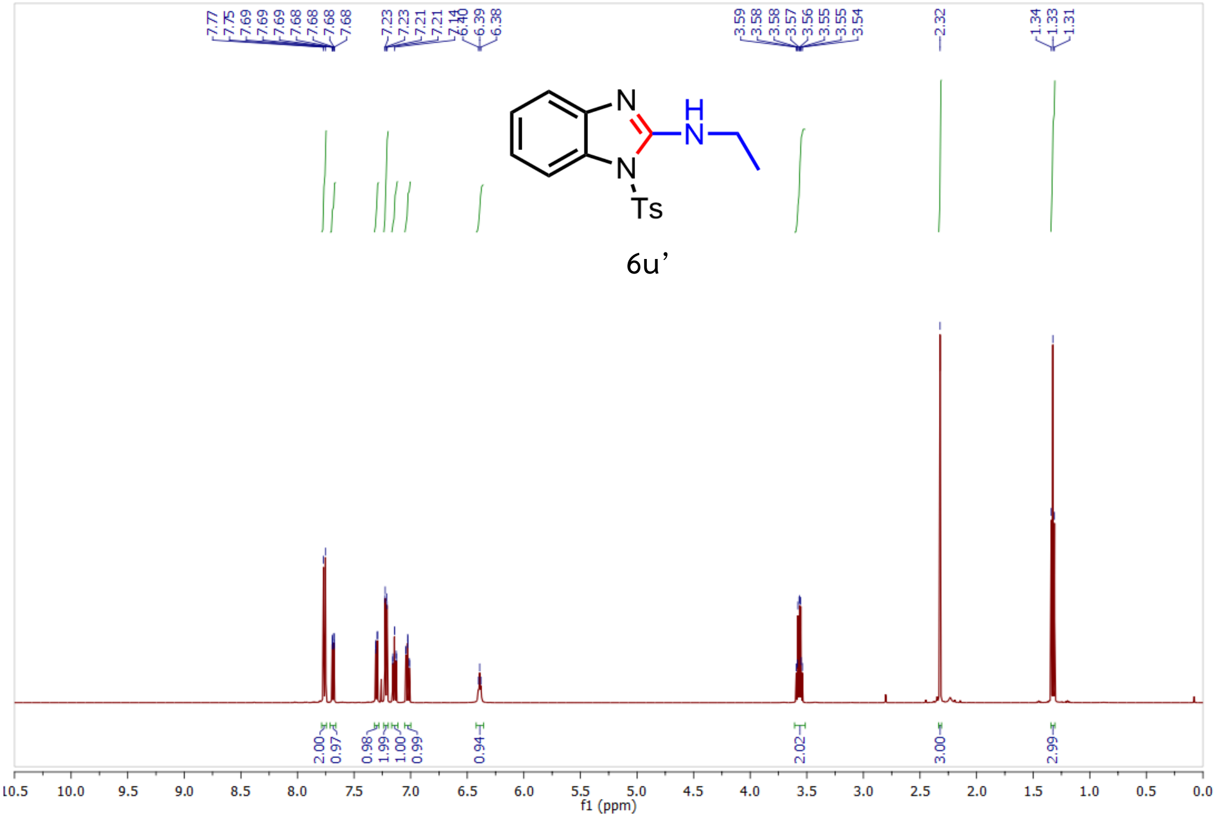

**Figure S131** $^{1}$H-NMR spectrum of **6u'** ($CDCl_3$, 500 MHz)

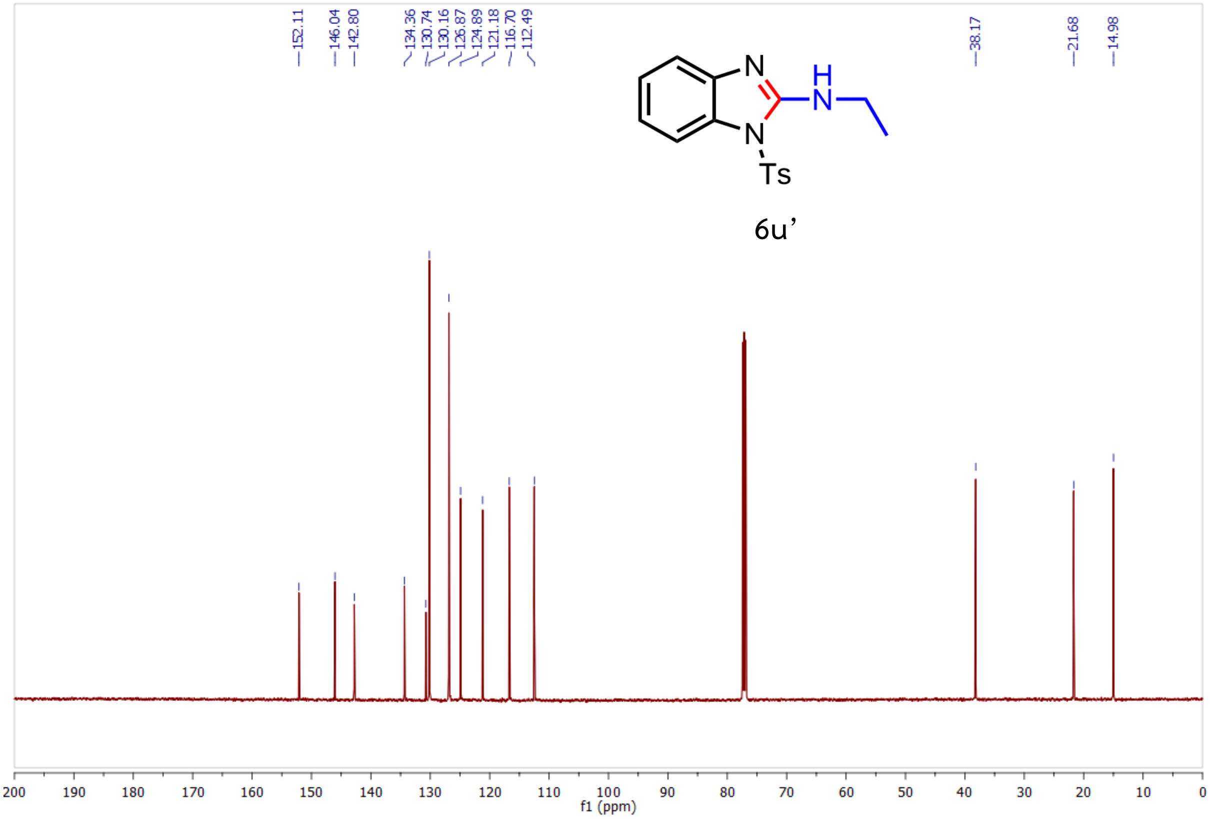

**Figure S132** $^{13}$C-NMR spectrum of **6u'** ($CDCl_3$, 126 MHz)

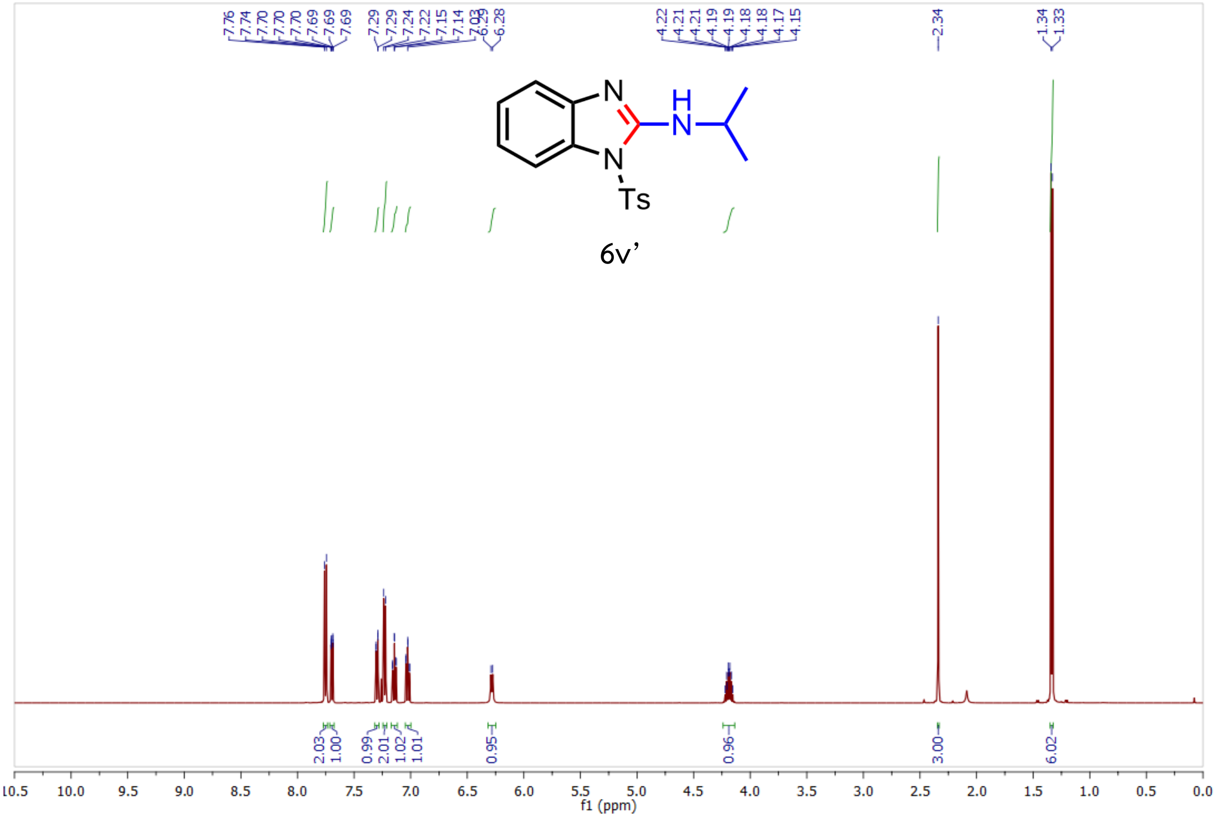

**Figure S133** $^{1}$H-NMR spectrum of **6v'** ($CDCl_3$, 500 MHz)

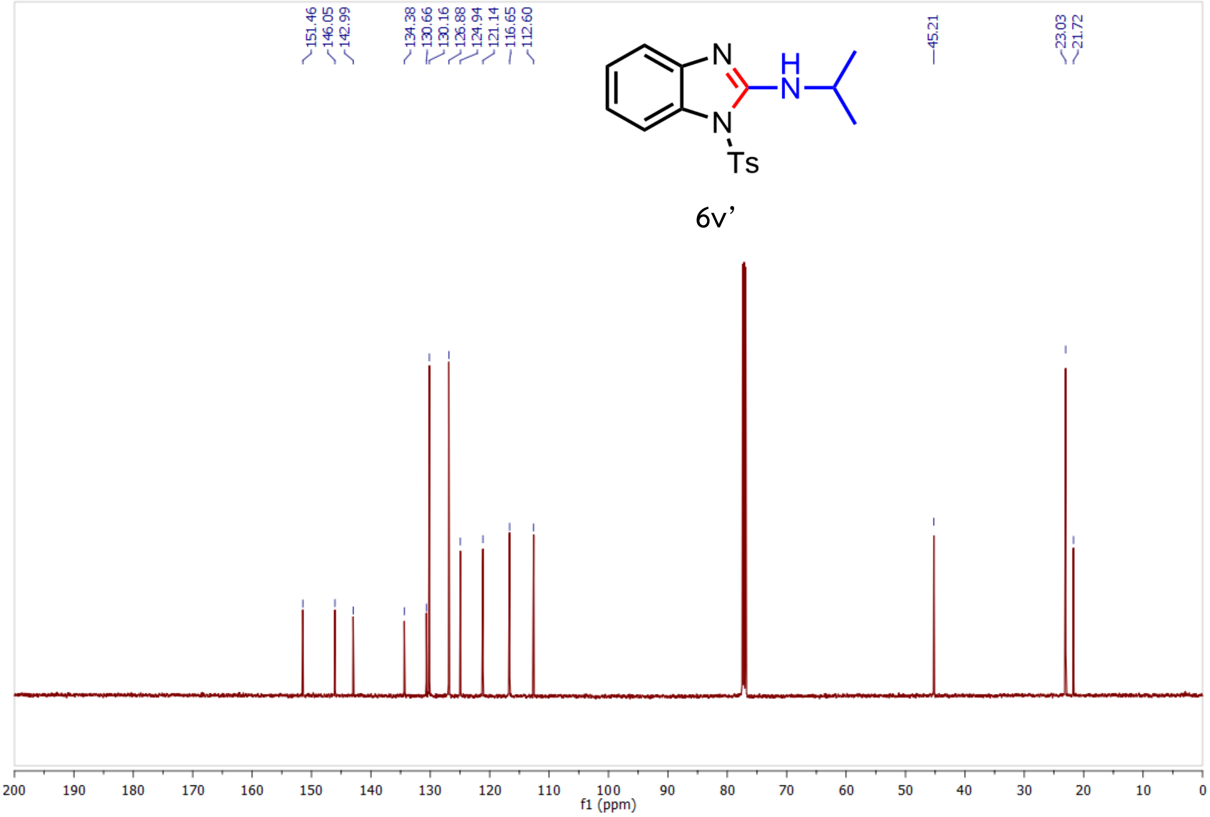

**Figure S134** $^{13}$C-NMR spectrum of **6v'** ($CDCl_3$, 126 MHz)

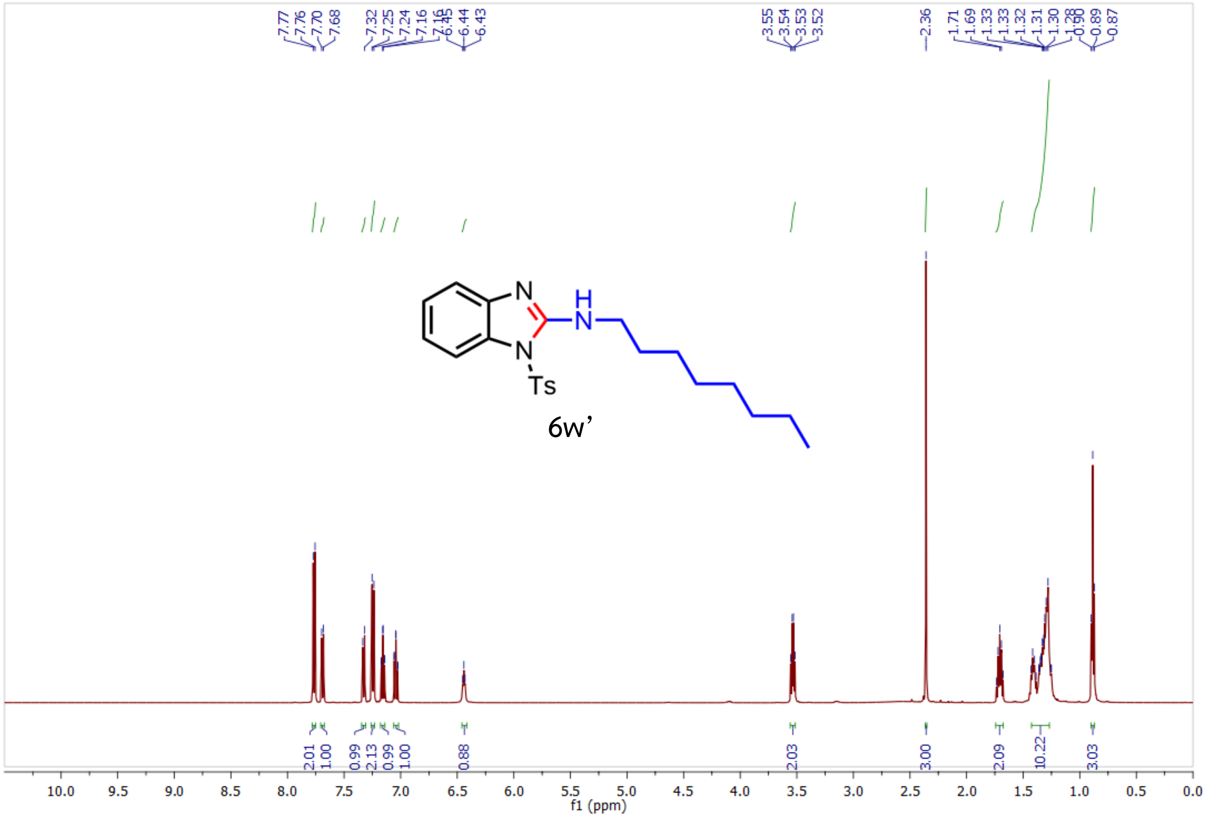

**Figure S135** $^{1}$H-NMR spectrum of **6w'** ($CDCl_3$, 500 MHz)

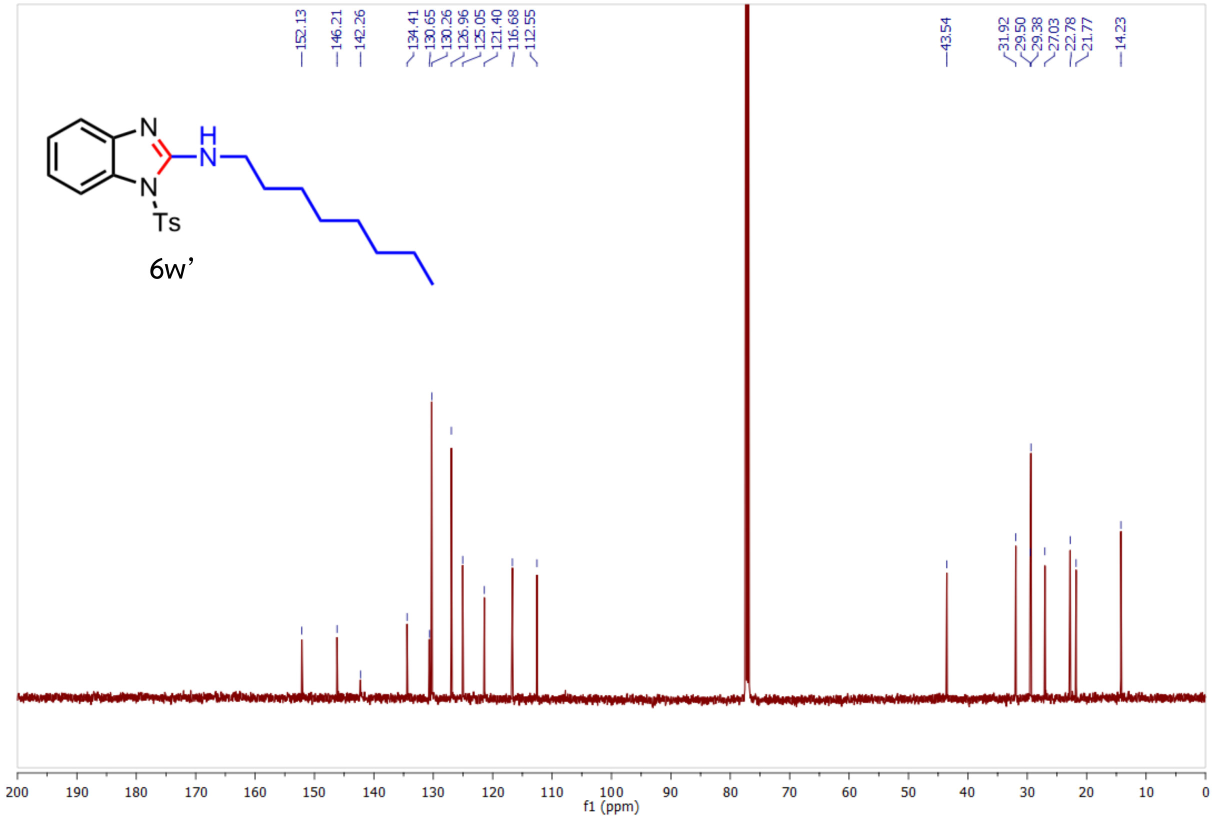

**Figure S136** $^{13}$C-NMR spectrum of **6w'** ($CDCl_3$, 126 MHz)

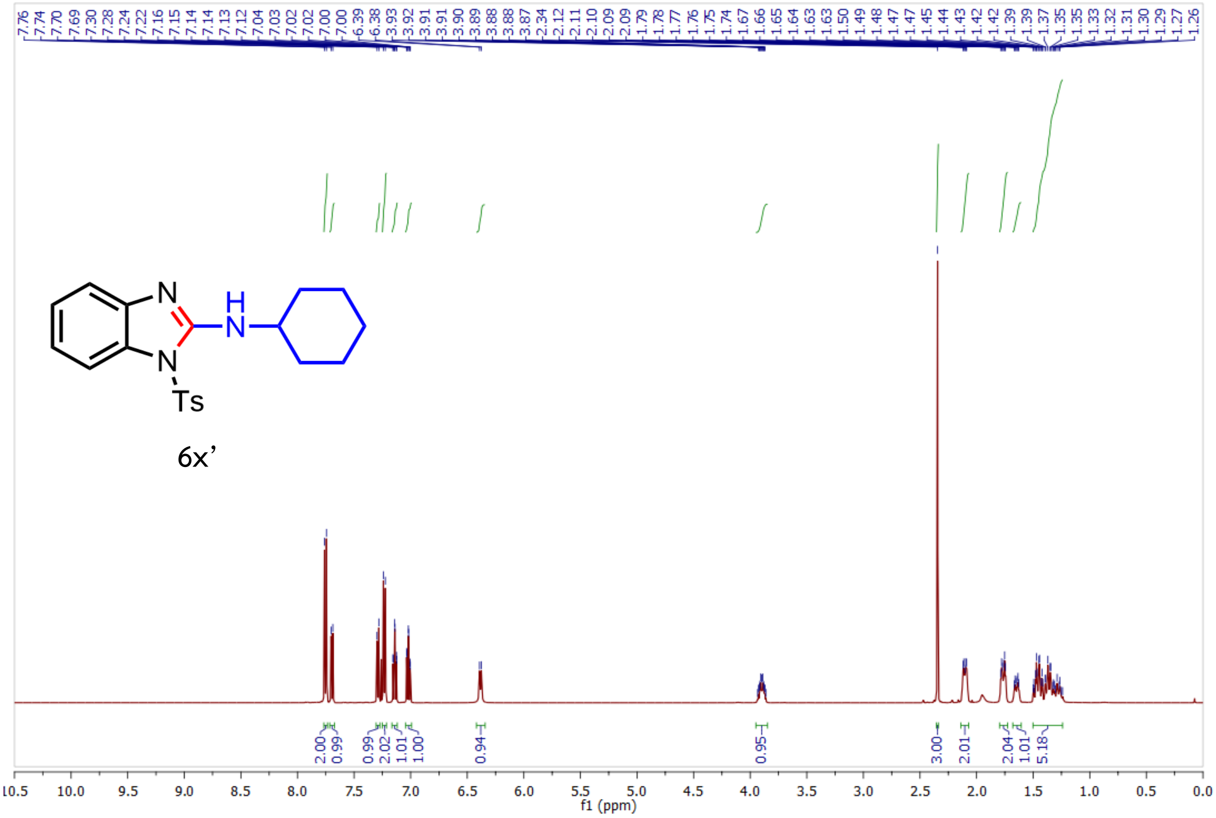


**Figure S137** $^{1}$H-NMR spectrum of **6x'** ($CDCl_3$, 500 MHz)

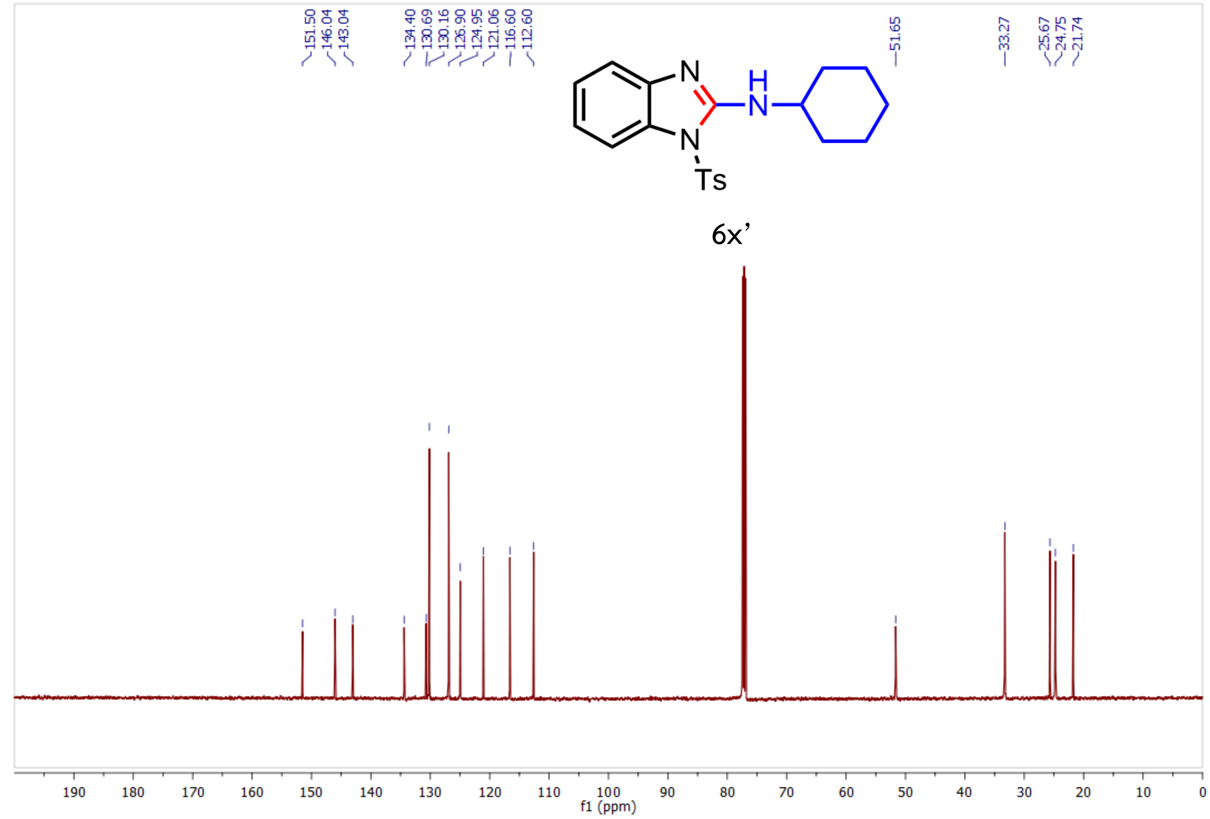


**Figure S138** $^{13}$C-NMR spectrum of **6x'** ($CDCl_3$, 126 MHz)

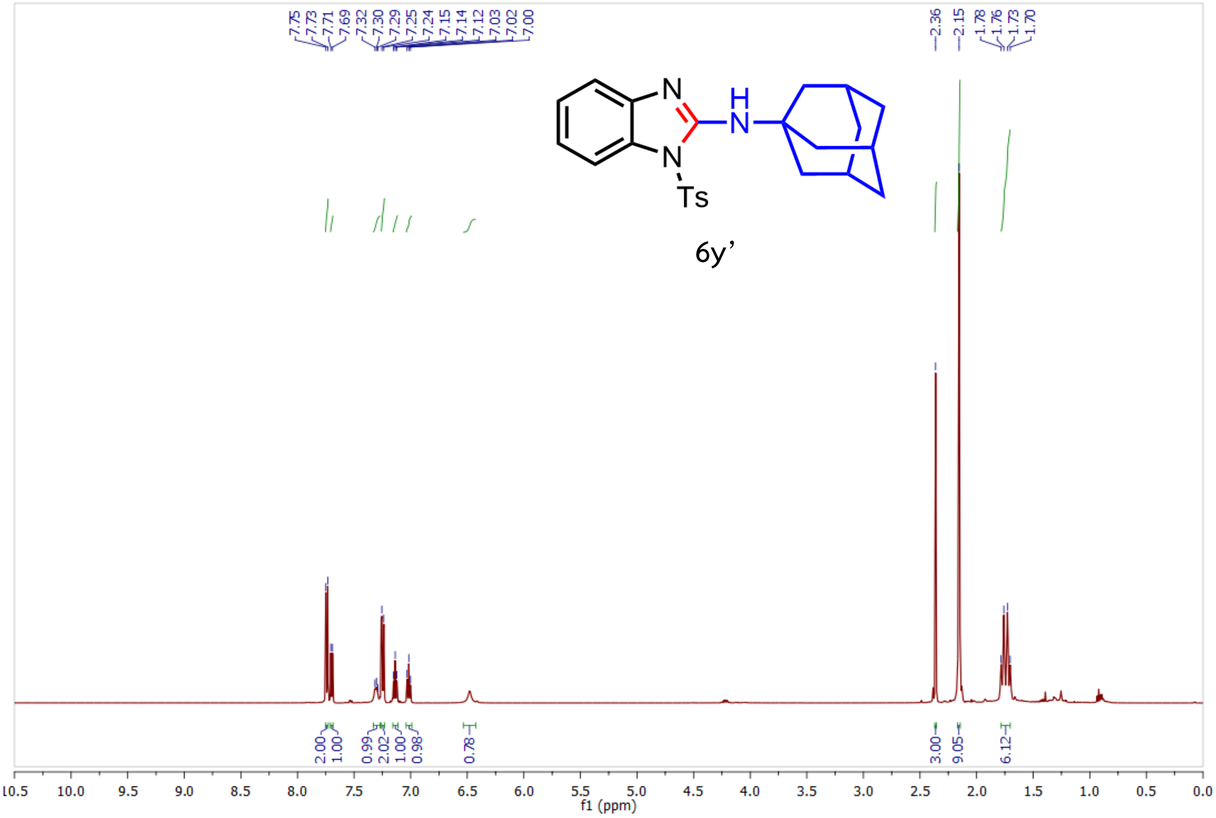


**Figure S139** $^{1}H$-NMR spectrum of **6y'** ($CDCl_3$, 500 MHz)

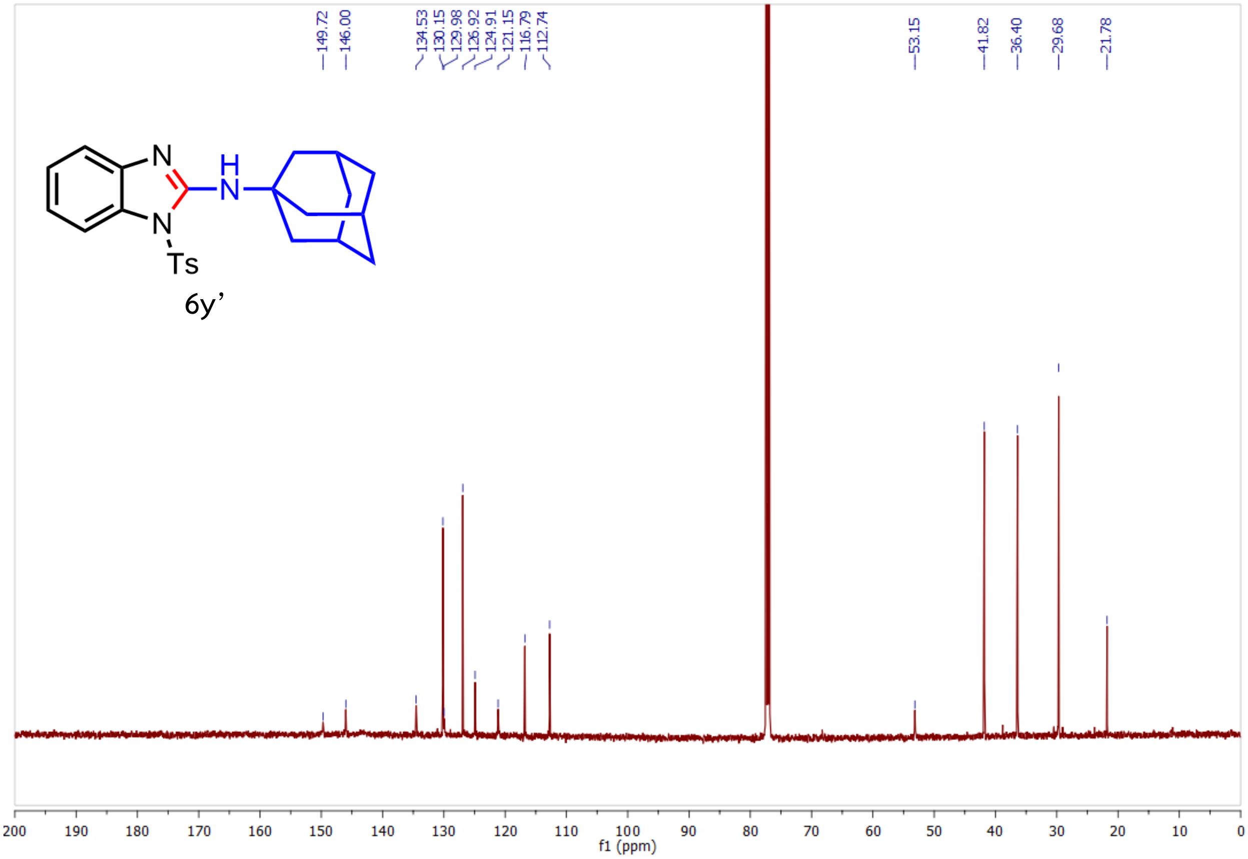


**Figure S140** $^{13}C$-NMR spectrum of **6y'** ($CDCl_3$, 126 MHz)

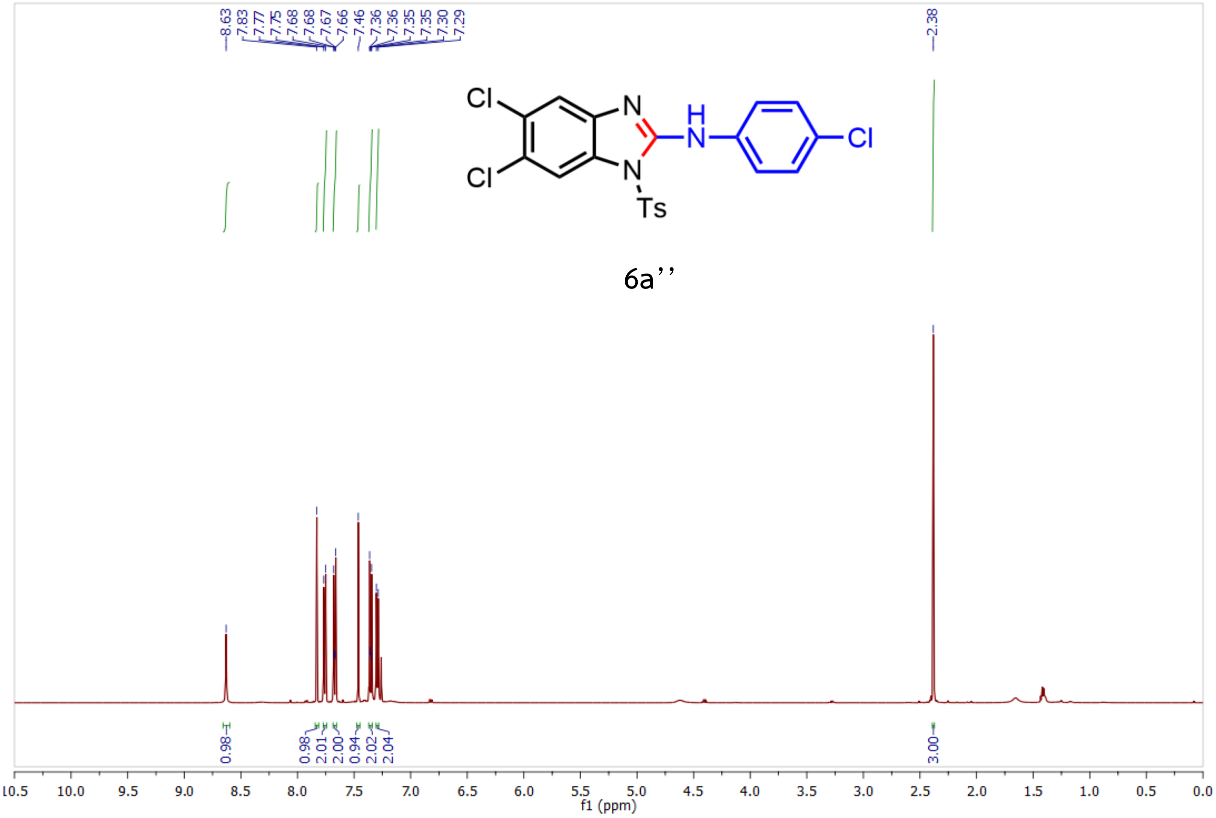


**Figure S141** $^{1}$H-NMR spectrum of **6a''** ($CDCl_3$, 500 MHz)

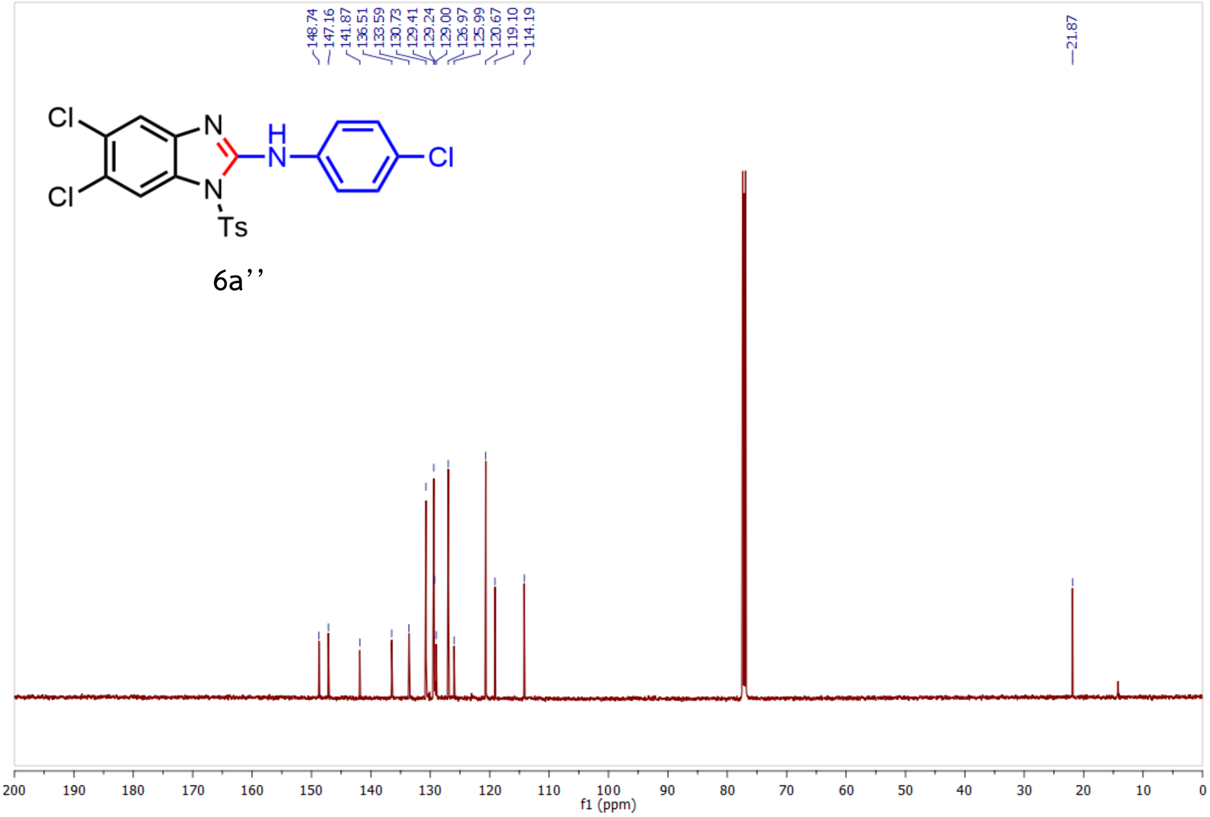


**Figure S142** $^{13}$C-NMR spectrum of **6a''** ($CDCl_3$, 126 MHz)

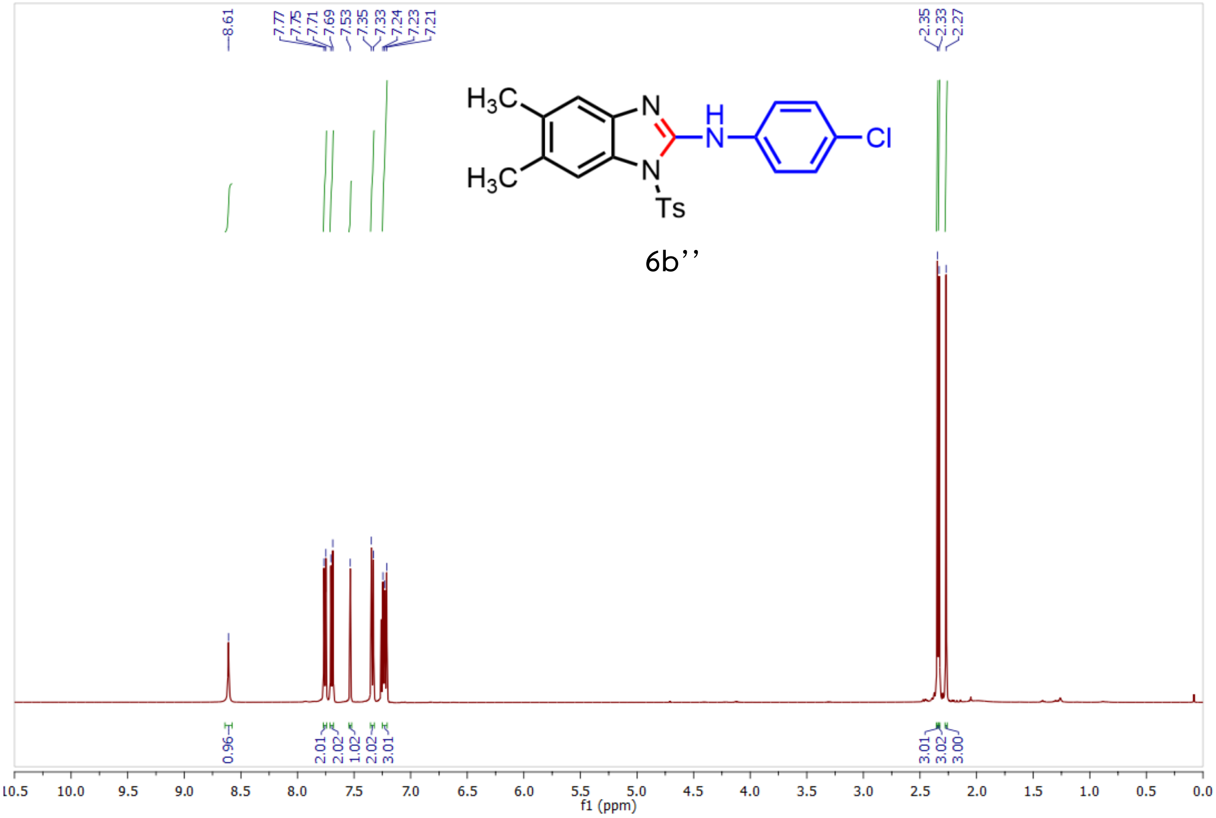


**Figure S143** $^{1}$H-NMR spectrum of **6b''** ($CDCl_3$, 500 MHz)

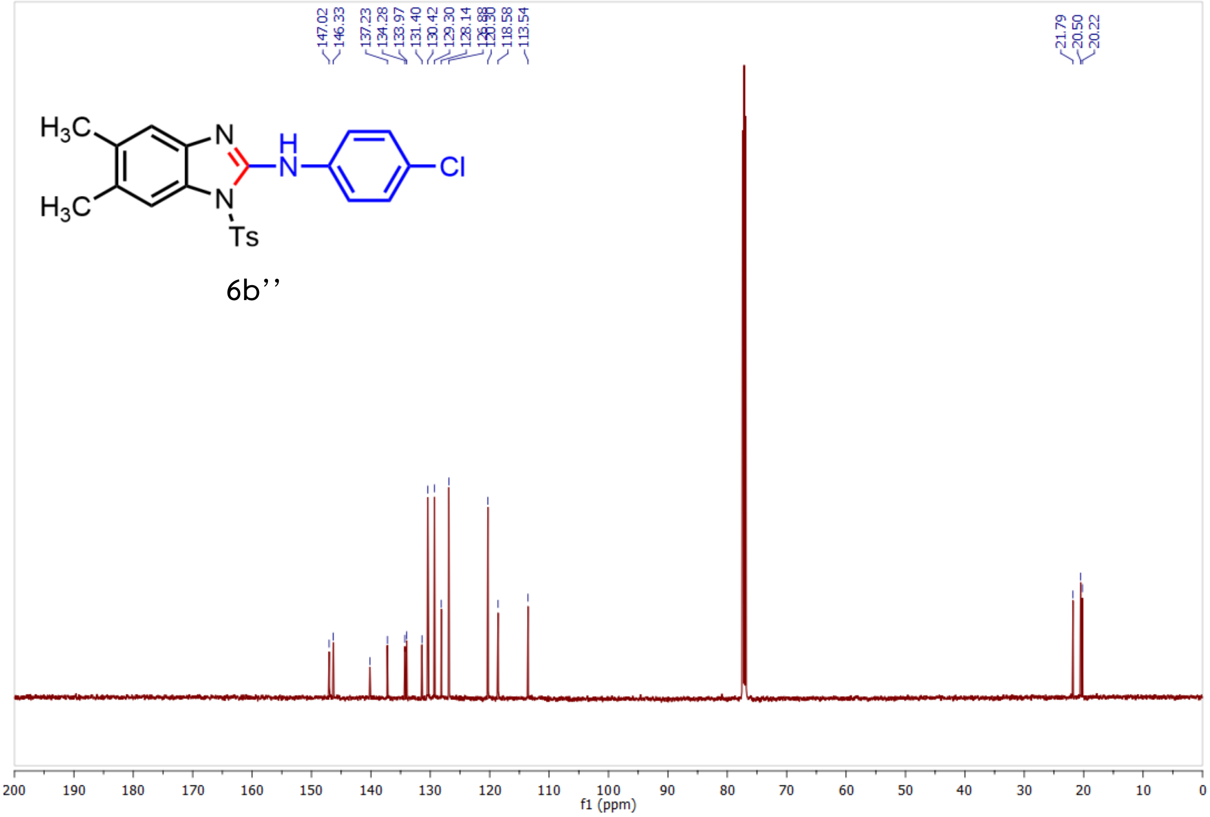


**Figure S144** $^{13}$C-NMR spectrum of **6b''** ($CDCl_3$, 126 MHz)

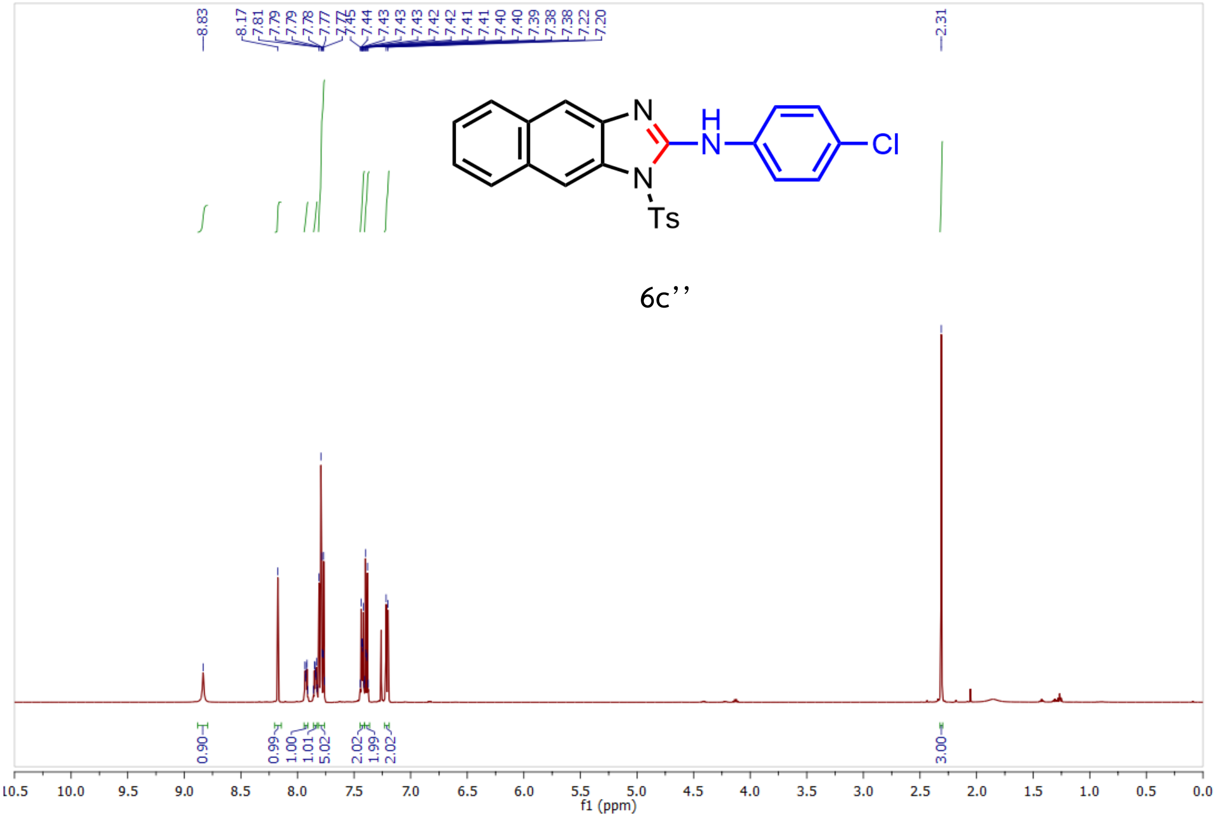


**Figure S145** $^1$H-NMR spectrum of **6c''** ($CDCl_3$, 500 MHz)

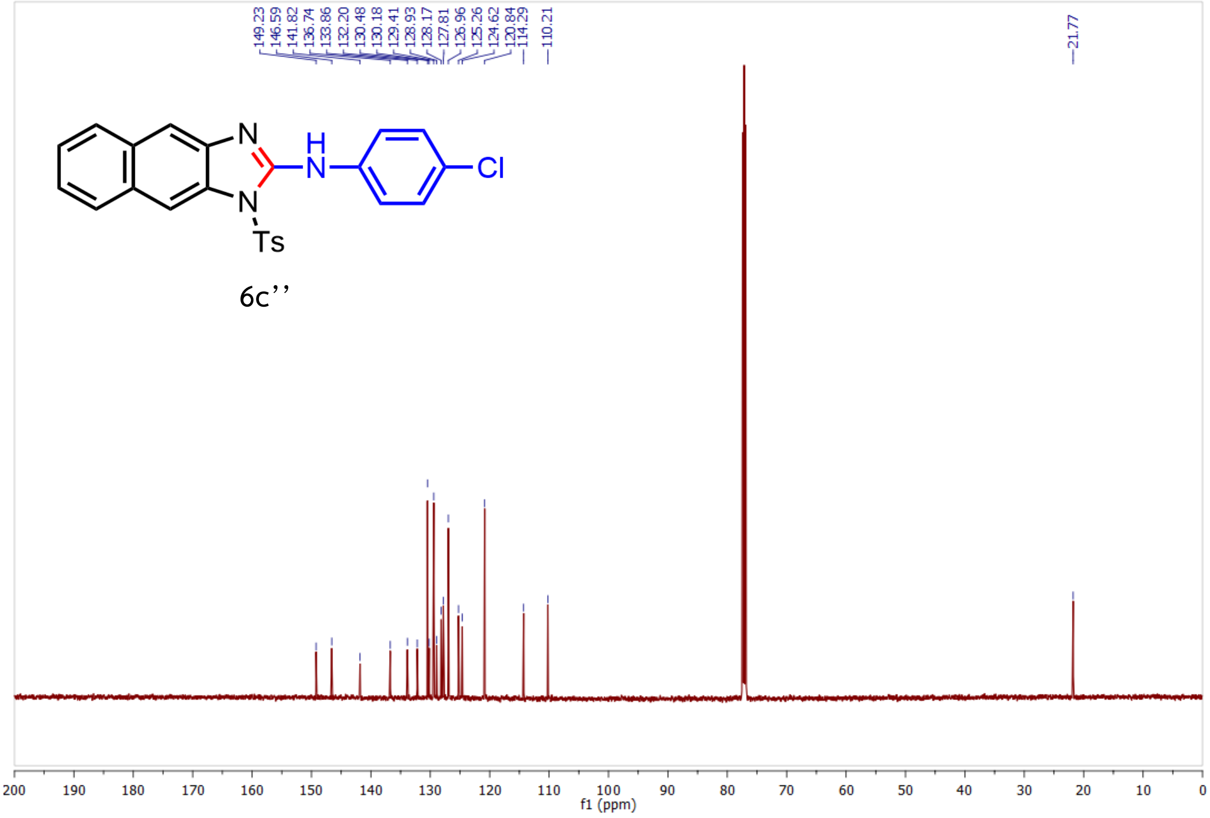


**Figure S146** $^{13}$C-NMR spectrum of **6c''** ($CDCl_3$, 126 MHz)

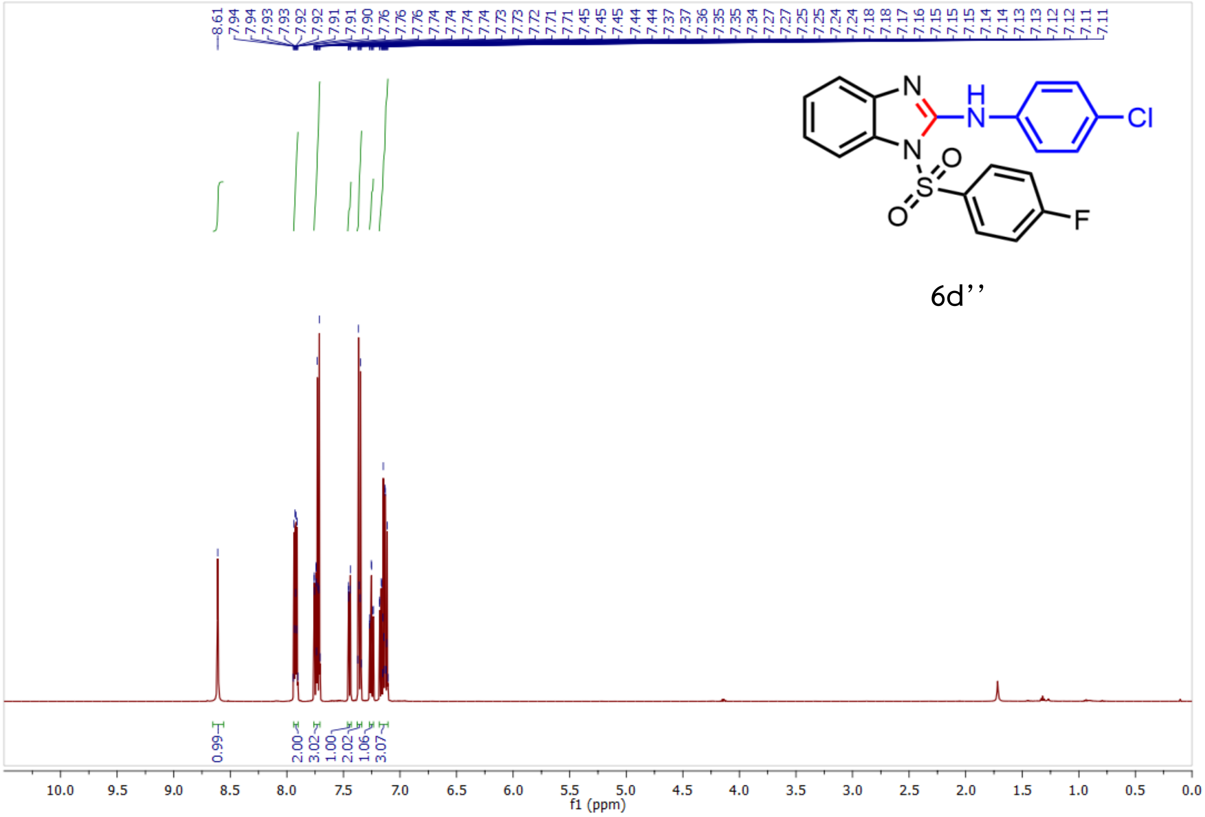

**Figure S147** $^{1}$H-NMR spectrum of **6d''** ($CDCl_3$, 500 MHz)

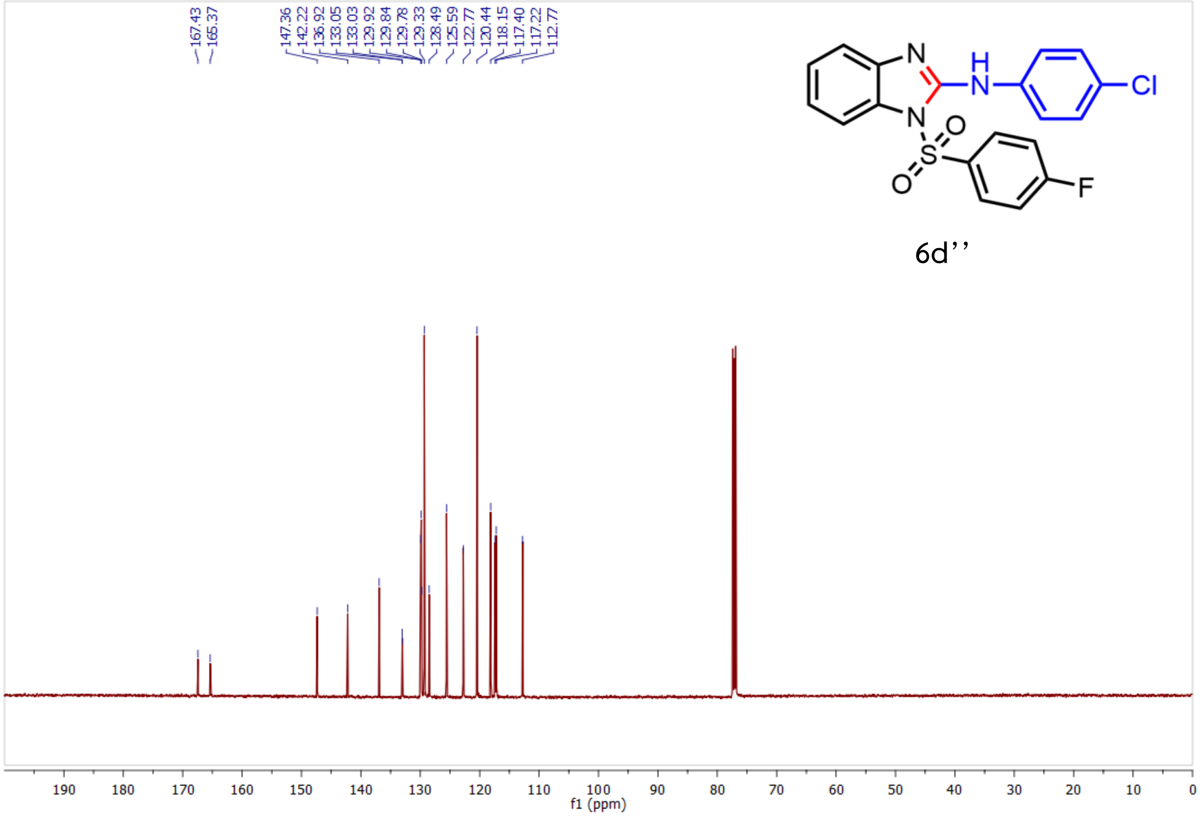

**Figure S148** $^{13}$C-NMR spectrum of **6d''** ($CDCl_3$, 126 MHz)

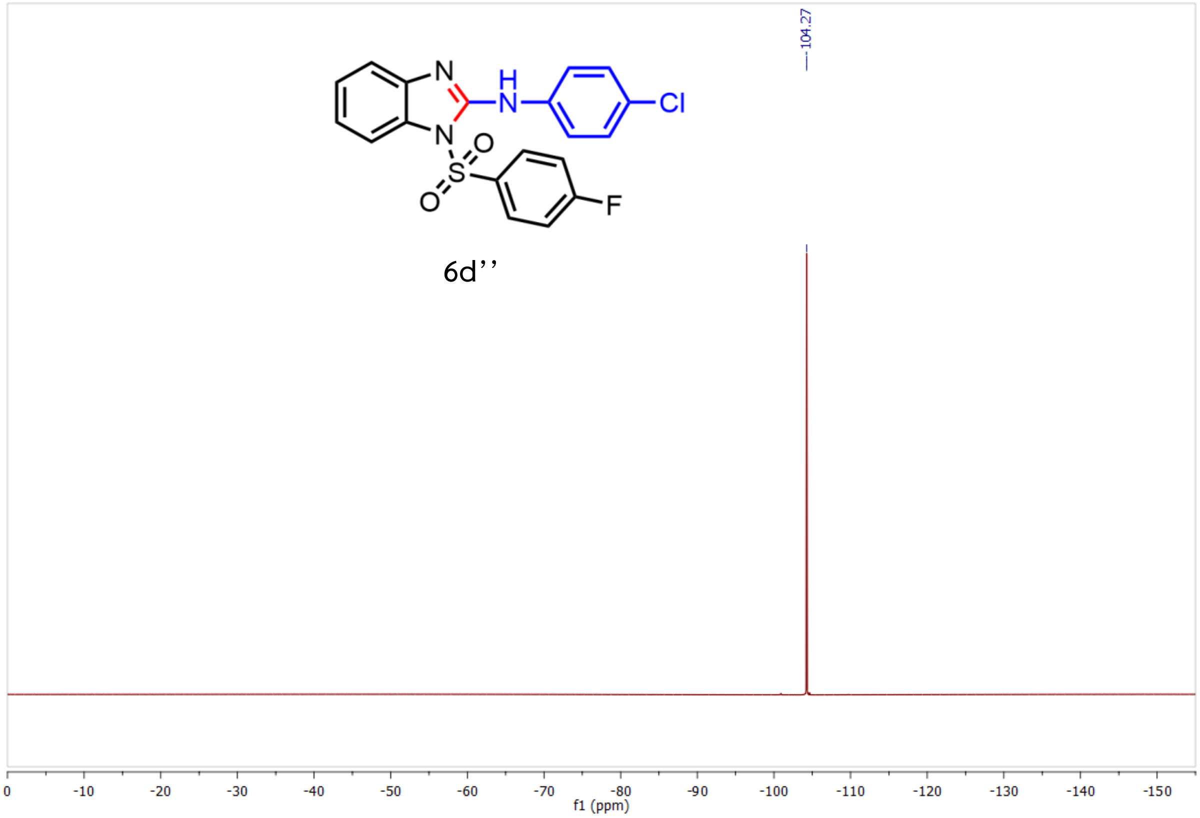


**Figure S149** $^{19}$F-NMR spectrum of **6d''** ($CDCl_3$, 471 MHz)

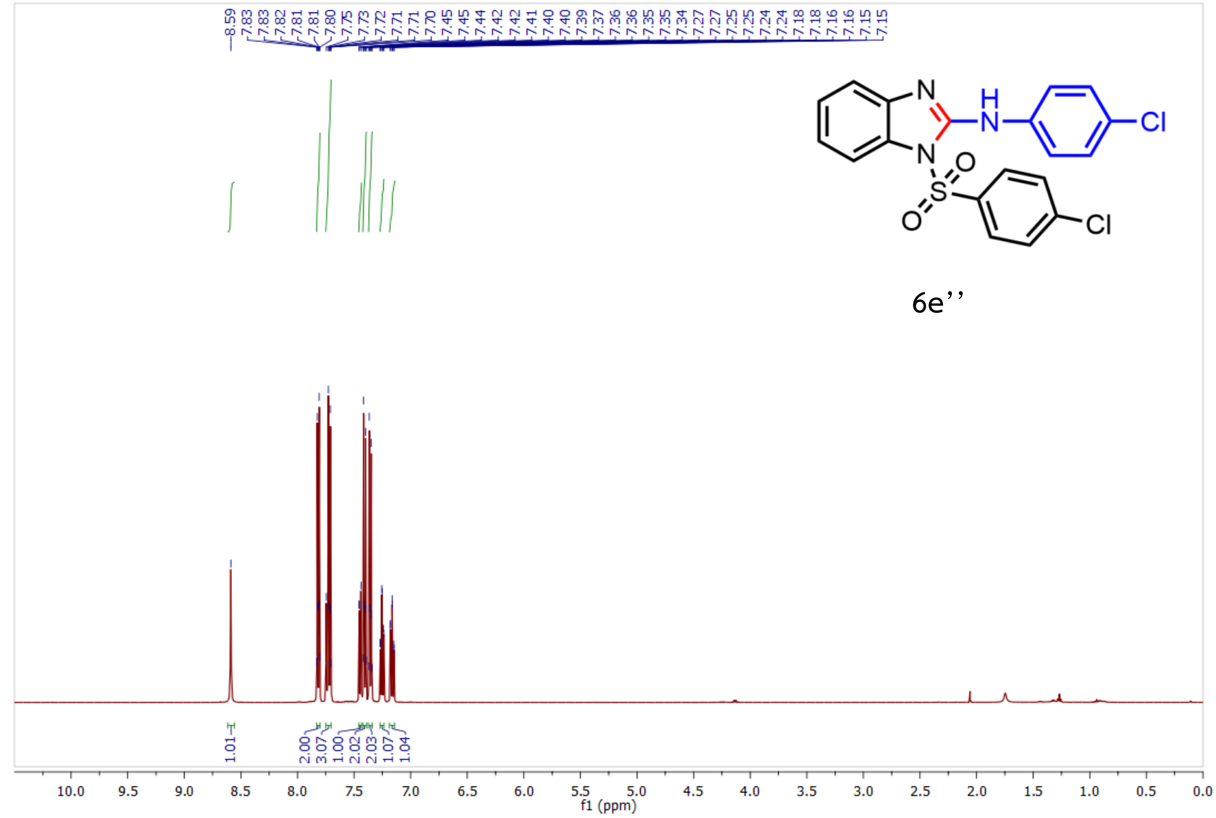

**Figure S150** $^{1}$H-NMR spectrum of **6e''** ($CDCl_3$, 500 MHz)

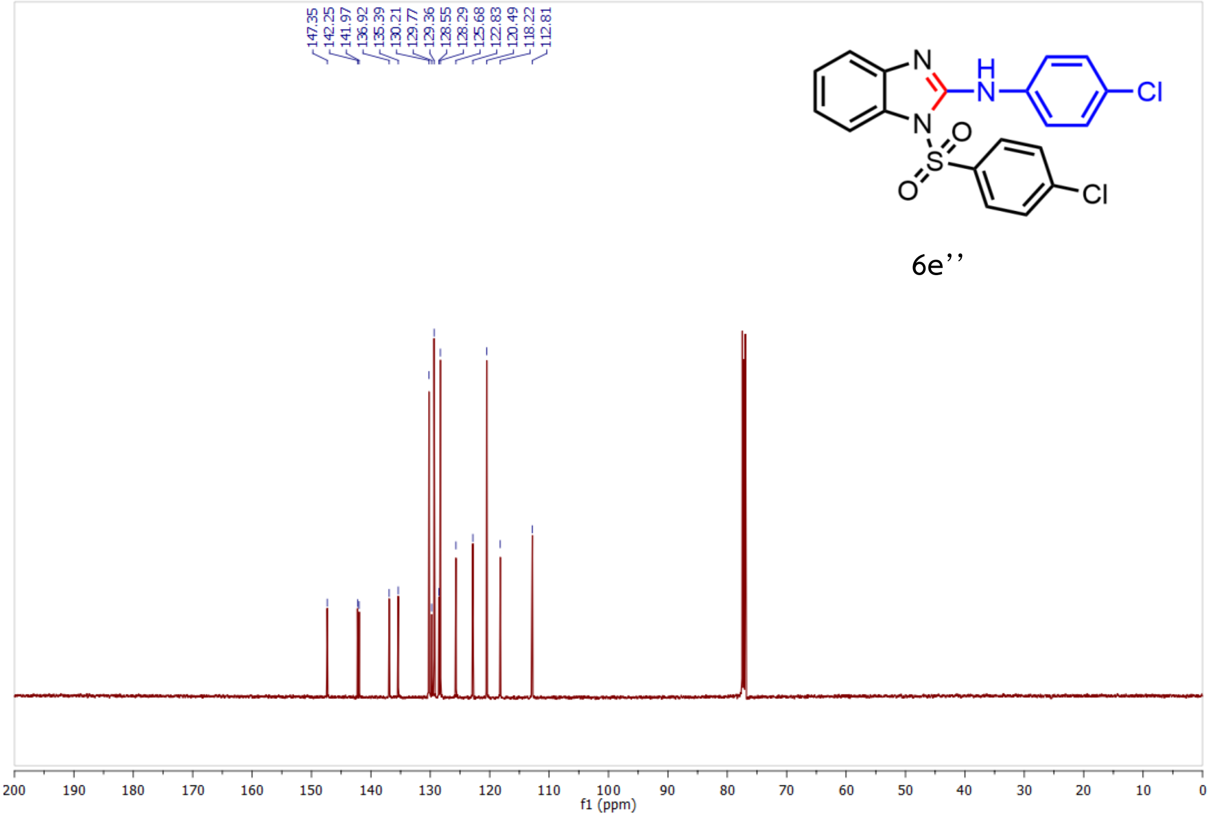

**Figure S151** $^{13}$C-NMR spectrum of **6e''** ($CDCl_3$, 126 MHz)

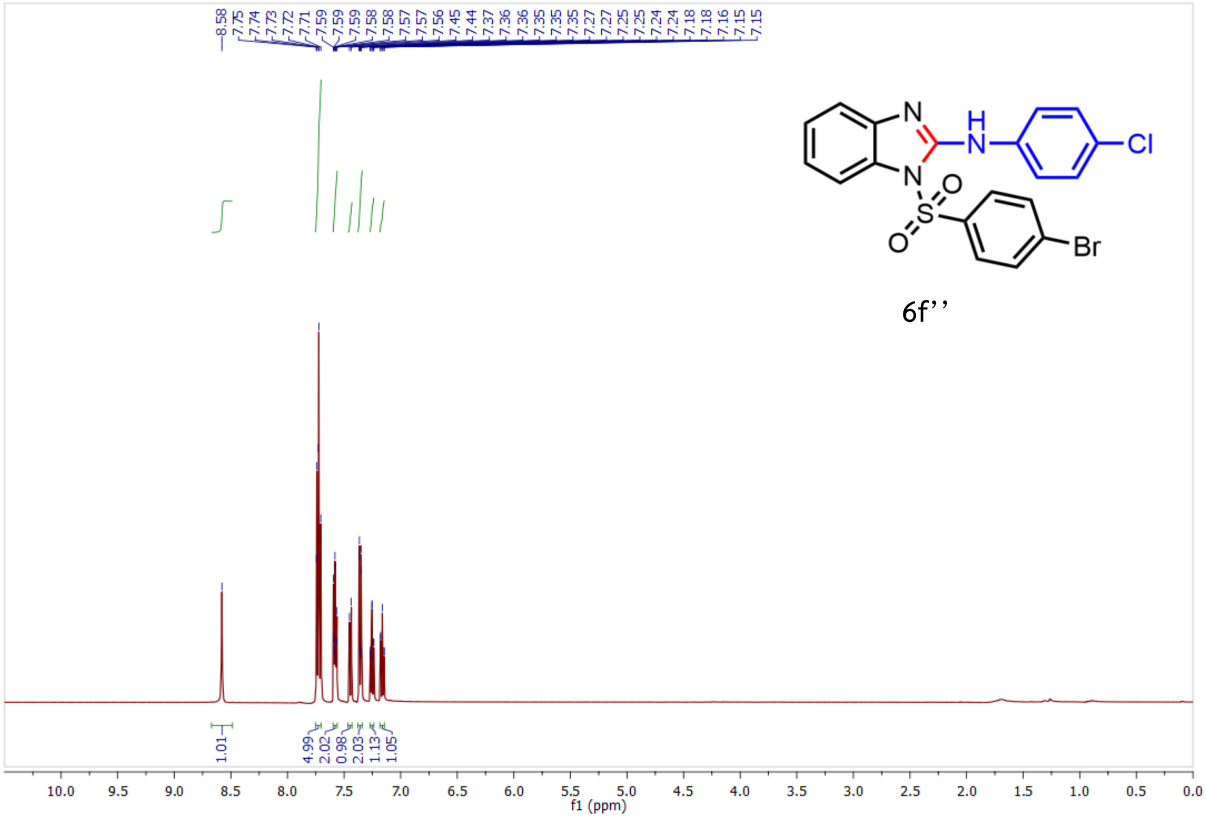

**Figure S152** $^{1}$H-NMR spectrum of **6f''** ($CDCl_3$, 500 MHz)

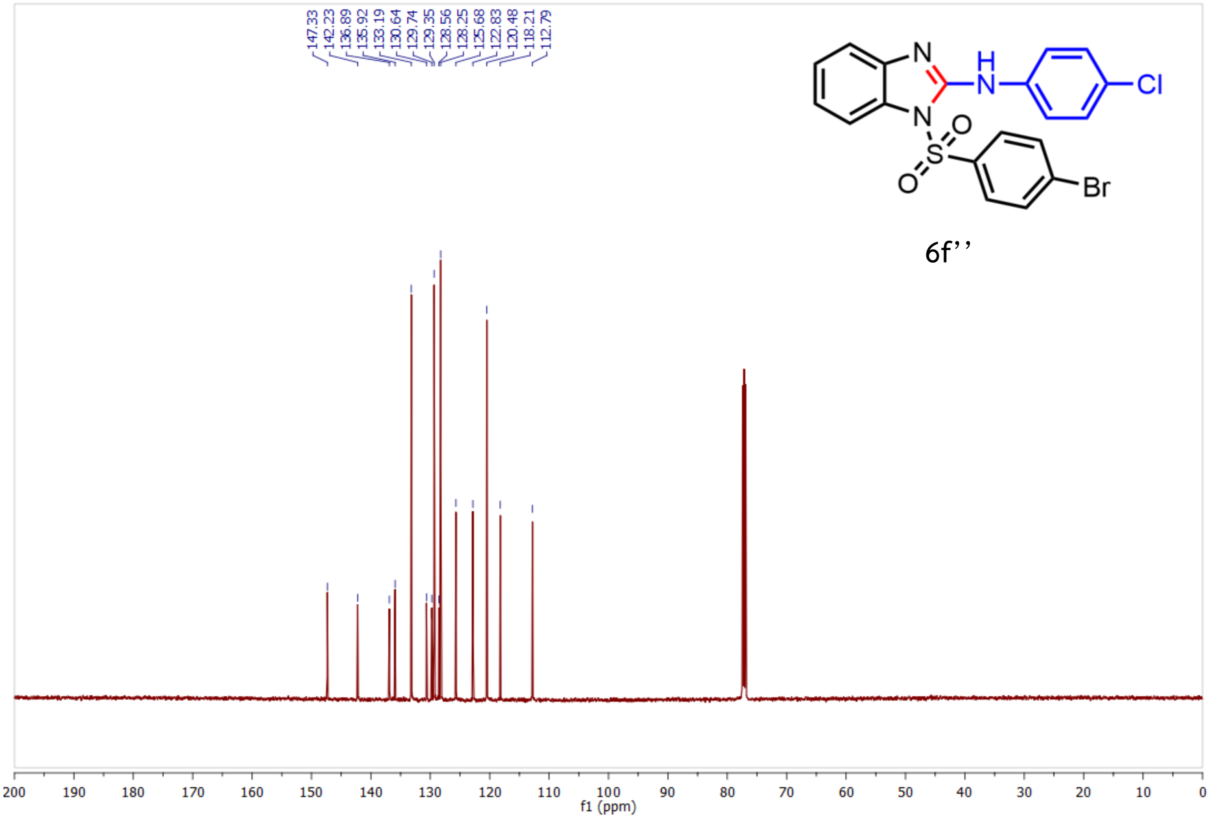

**Figure S153** $^{13}$C-NMR spectrum of **6f''** ($CDCl_3$, 126 MHz)

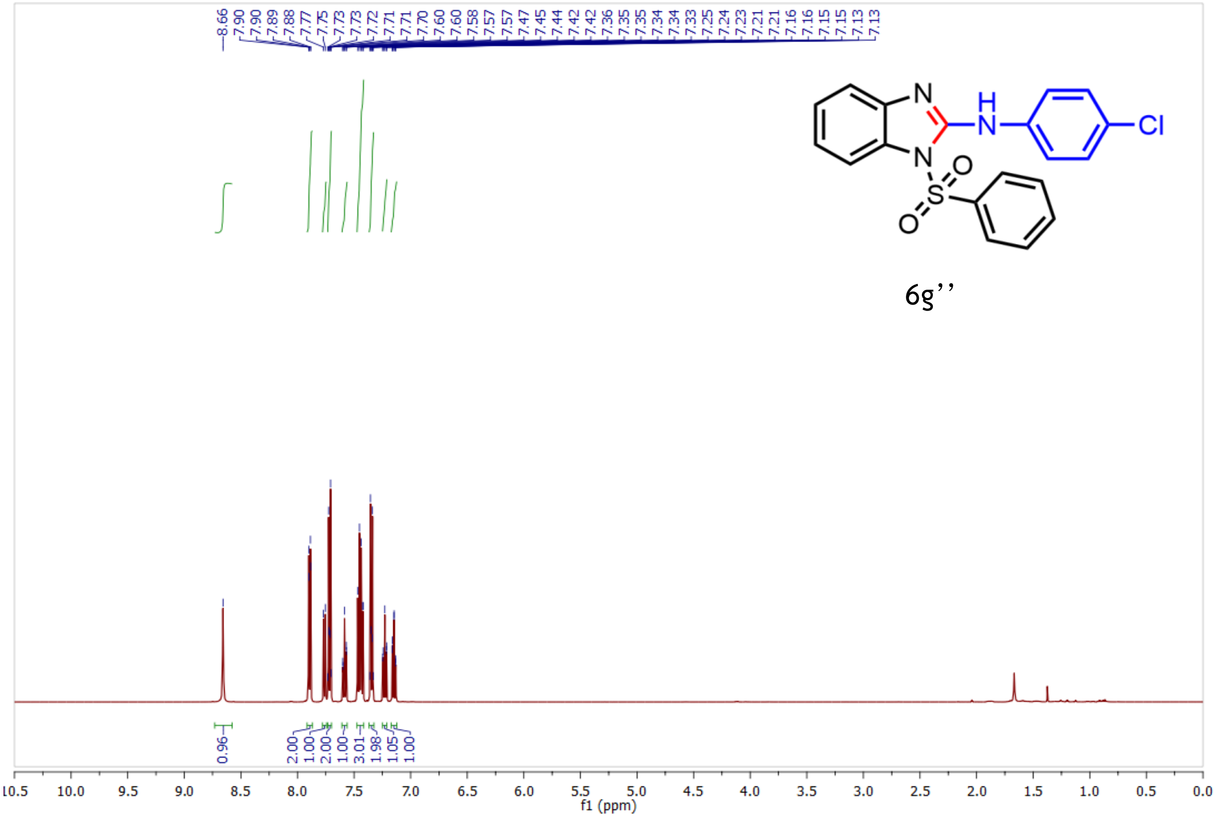

**Figure S154** $^{1}$H-NMR spectrum of **6g''** ($CDCl_3$, 500 MHz)

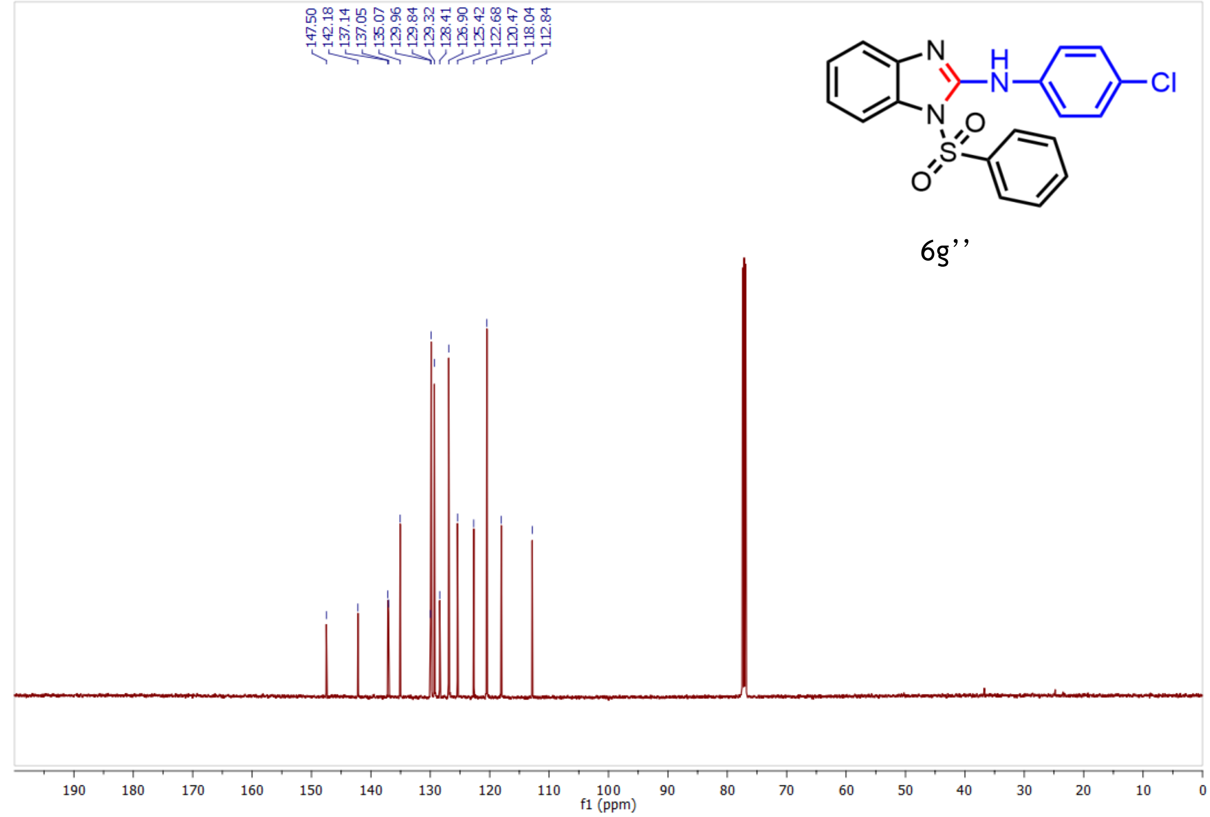

**Figure S155** $^{13}$C-NMR spectrum of **6g''** ($CDCl_3$, 126 MHz)

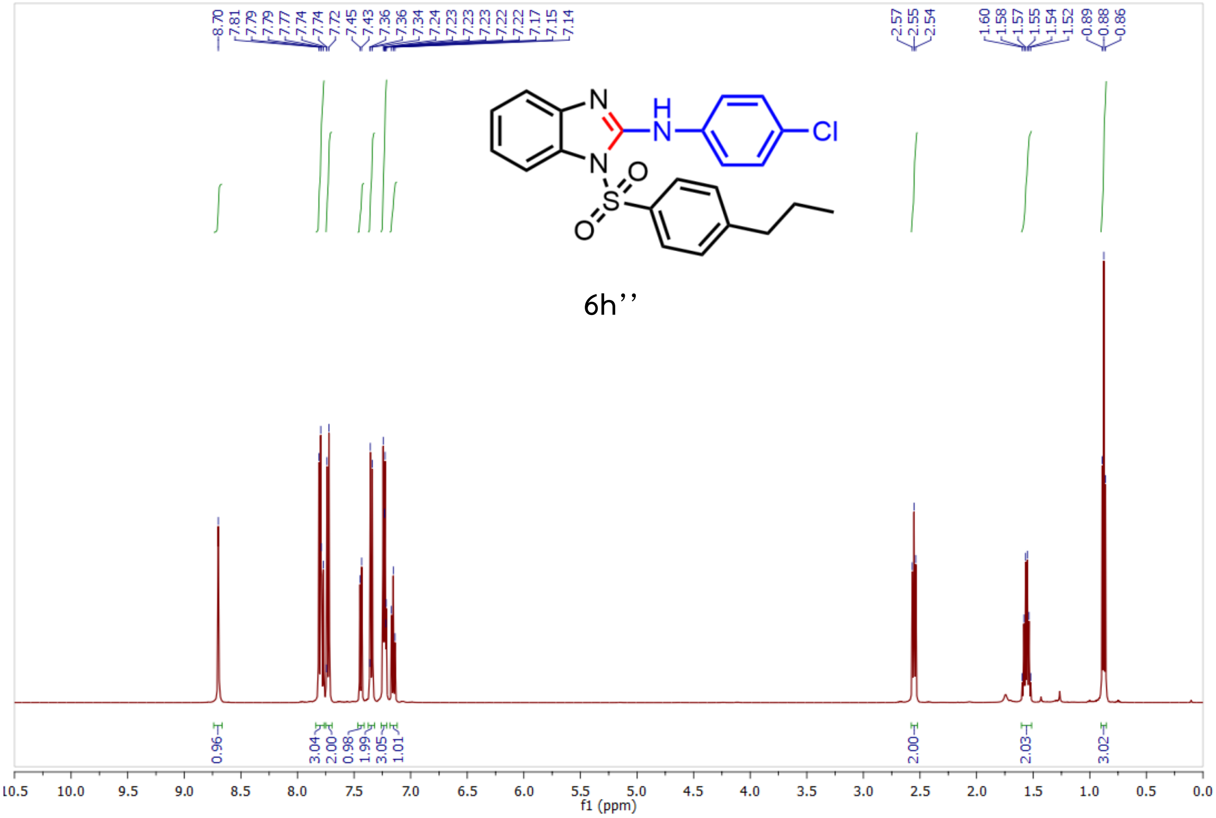

**Figure S156** $^{1}$H-NMR spectrum of **6h''** ($CDCl_3$, 500 MHz)

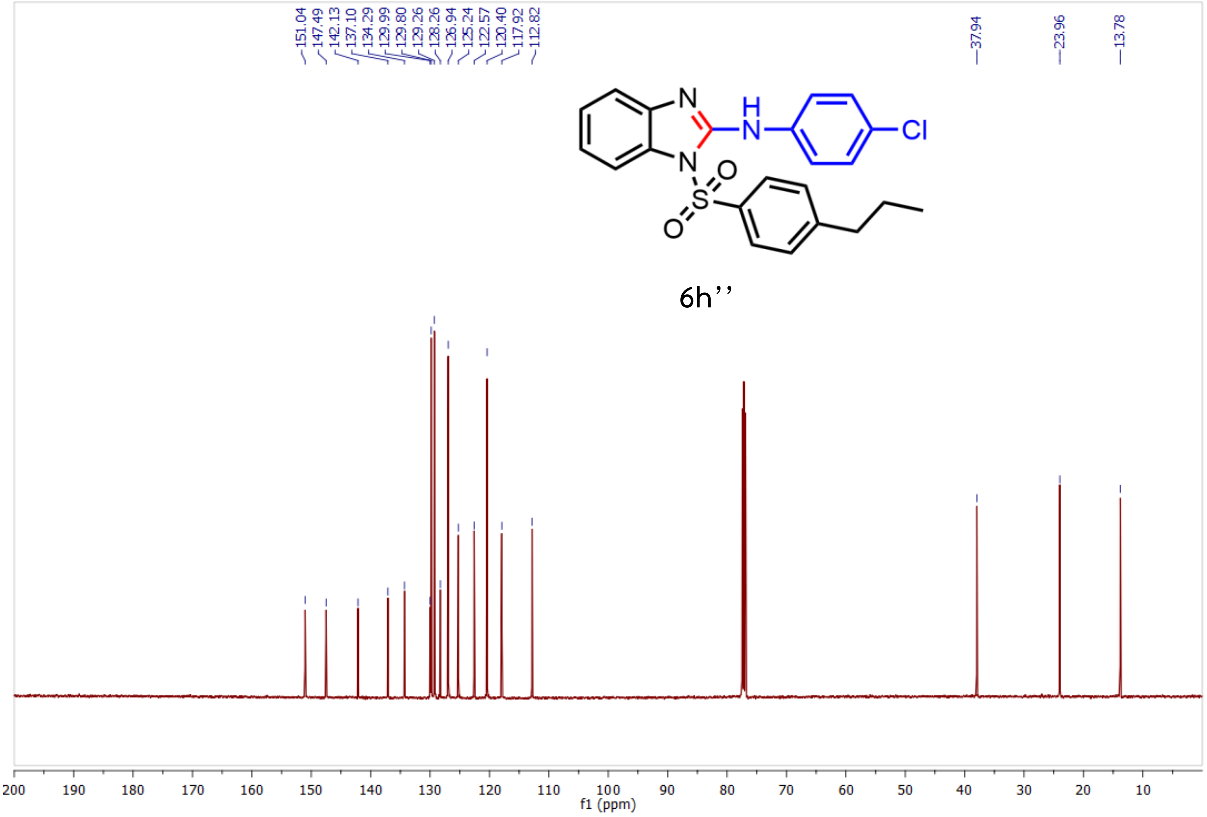

**Figure S157** $^{13}$C-NMR spectrum of **6h''** ($CDCl_3$, 126 MHz)

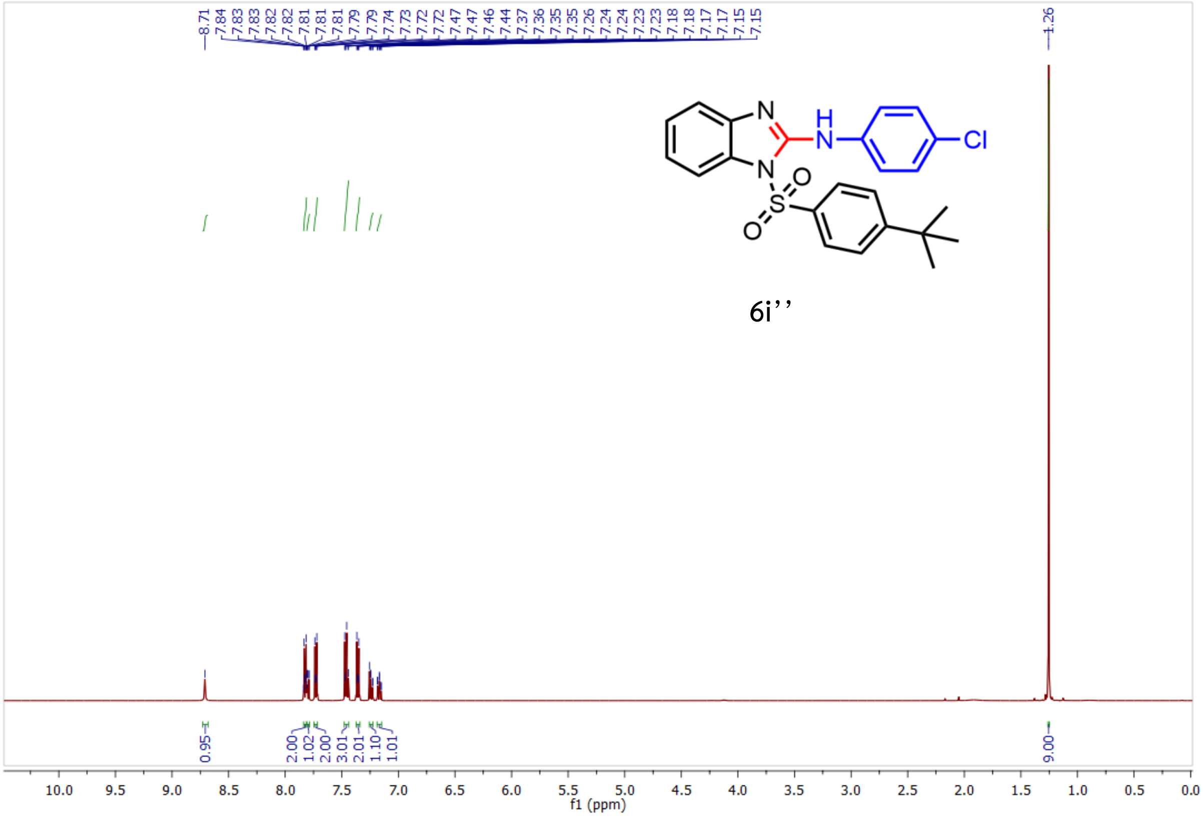


**Figure S158** $^{1}$H-NMR spectrum of **6i''** ($CDCl_3$, 500 MHz)

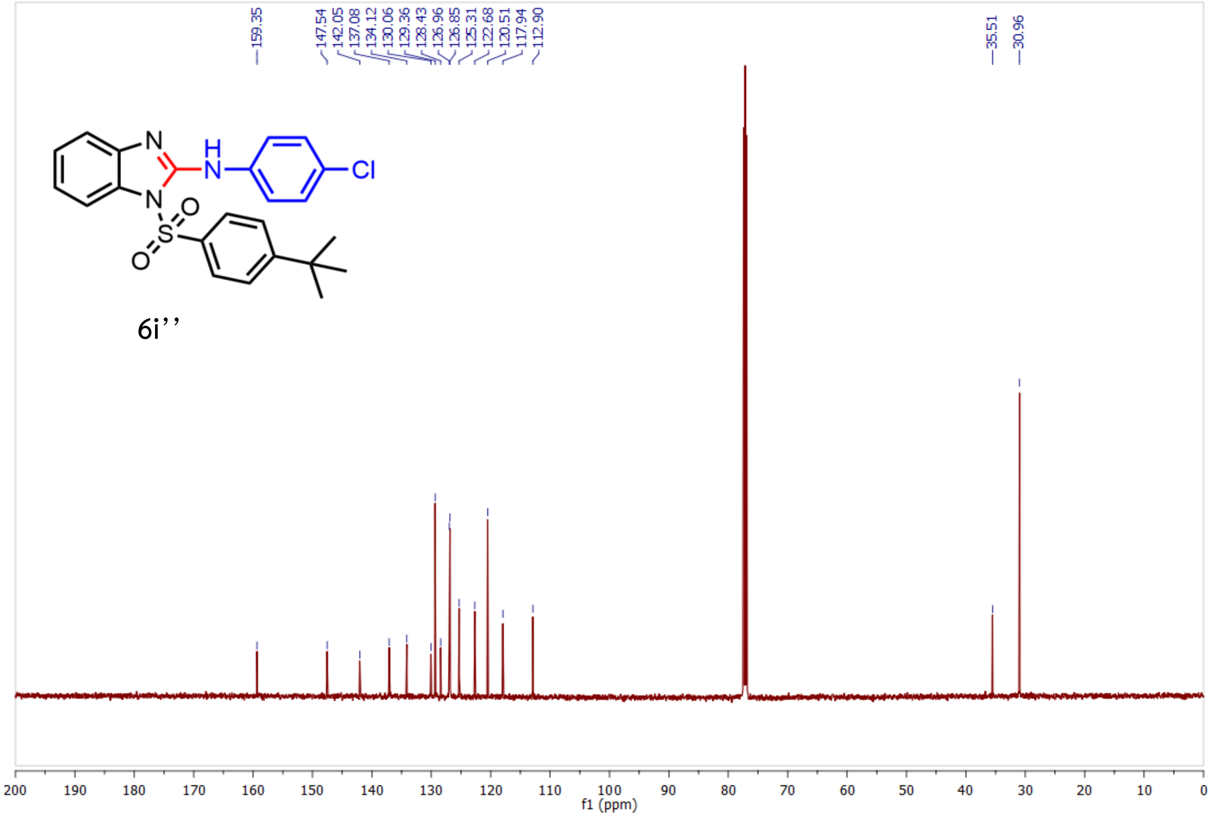


**Figure S159** $^{13}$C-NMR spectrum of **6i''** ($CDCl_3$, 126 MHz)

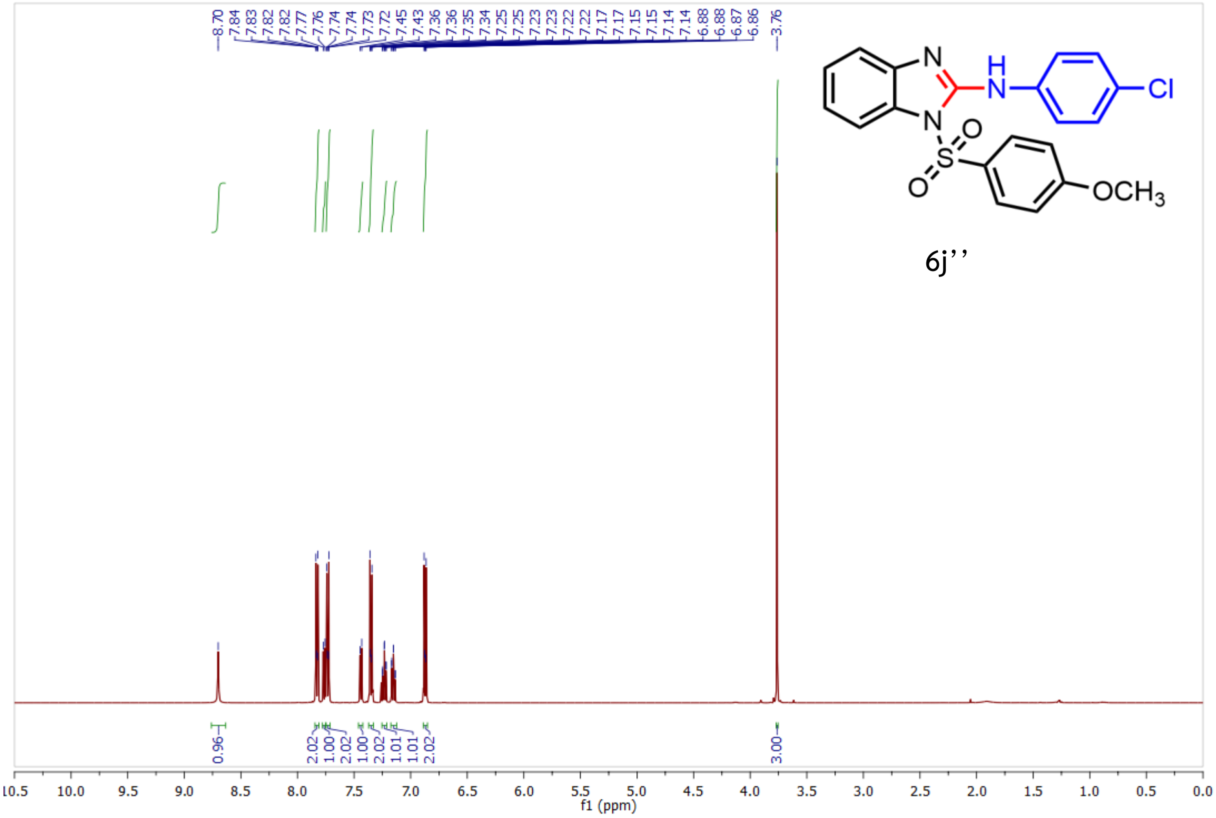

Figure S160 $^{1}$H-NMR spectrum of **6j''** ($CDCl_3$, 500 MHz)

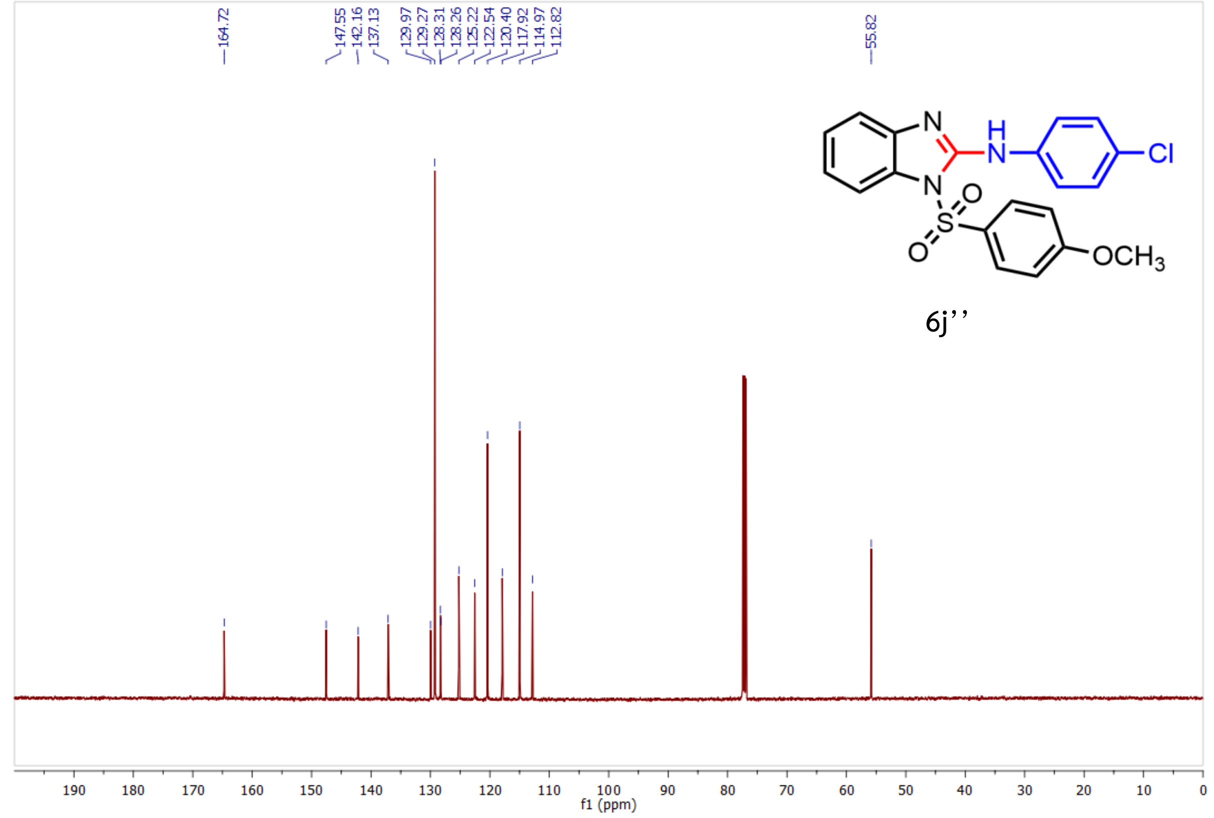

Figure S161 $^{13}$C-NMR spectrum of **6j''** ($CDCl_3$, 126 MHz)

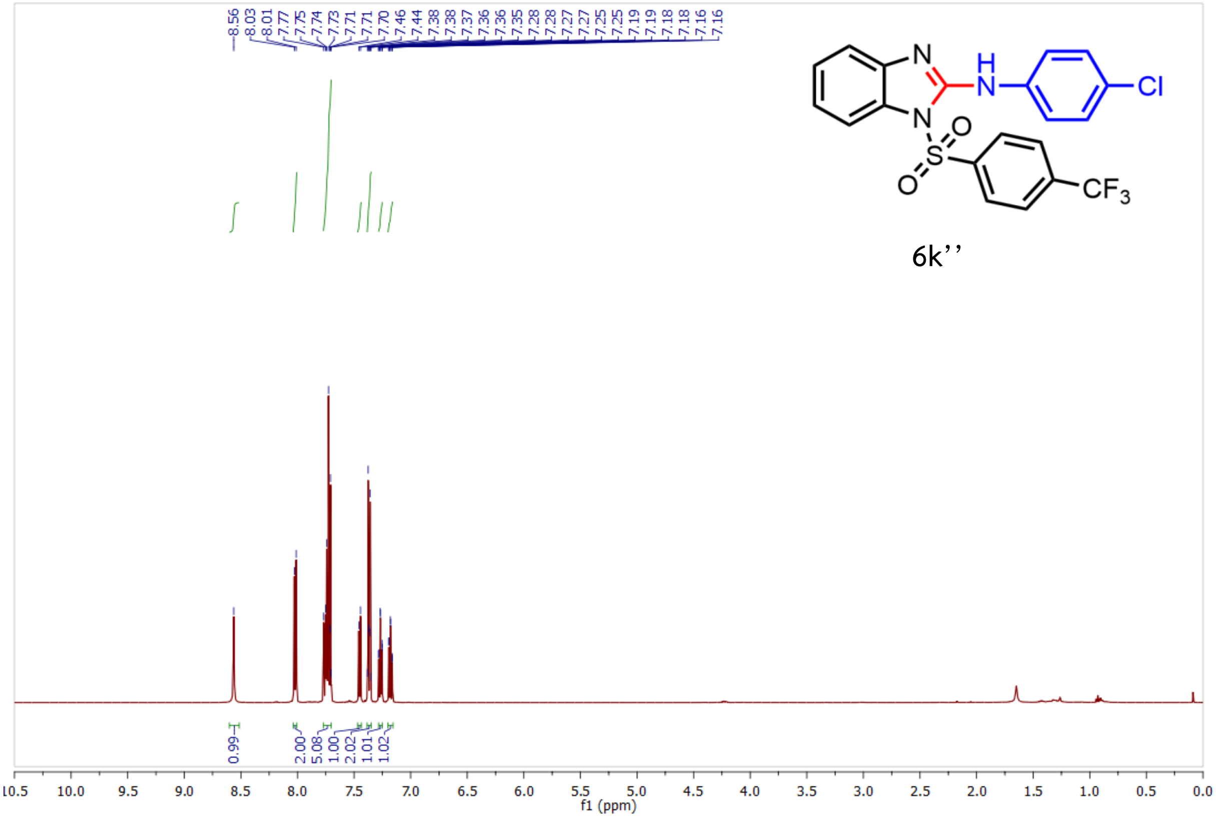


**Figure S162** $^{1}$H-NMR spectrum of **6k''** ($CDCl_3$, 500 MHz)

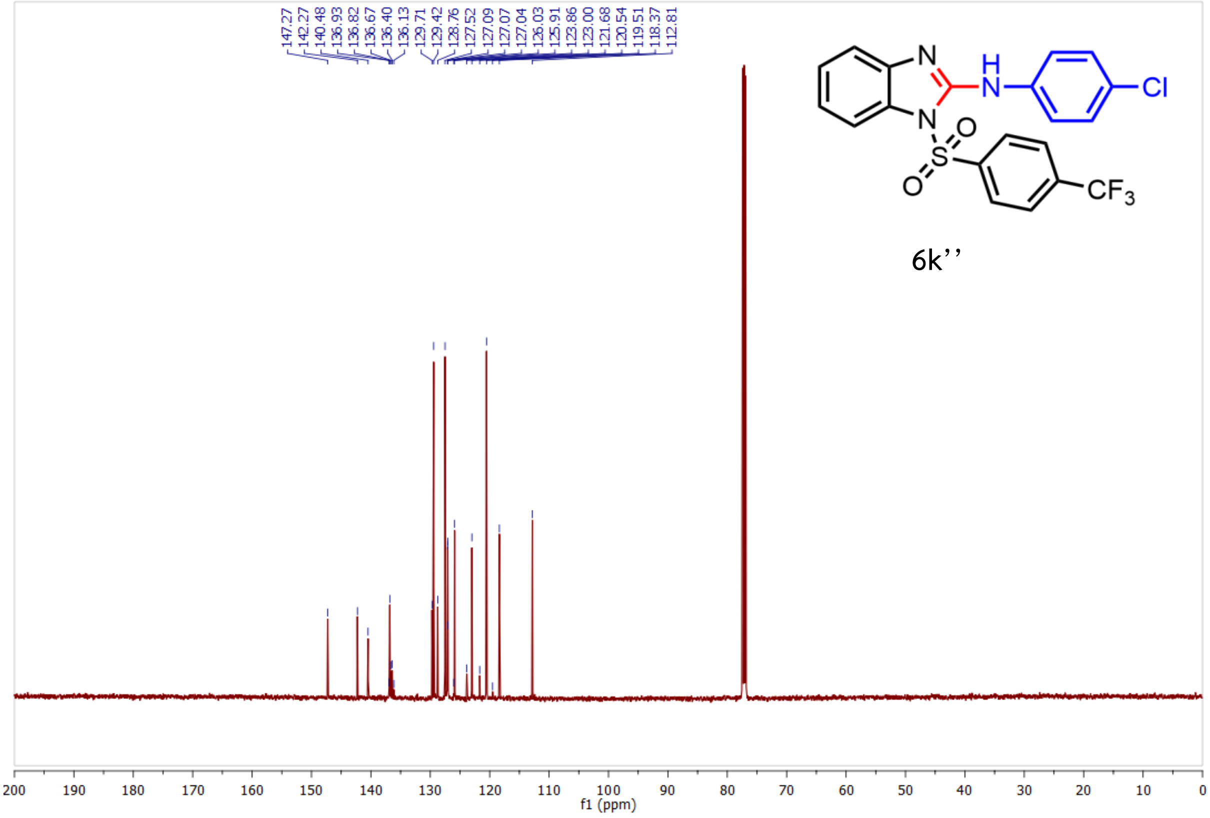


**Figure S163** $^{13}$C-NMR spectrum of **6k''** ($CDCl_3$, 126 MHz)

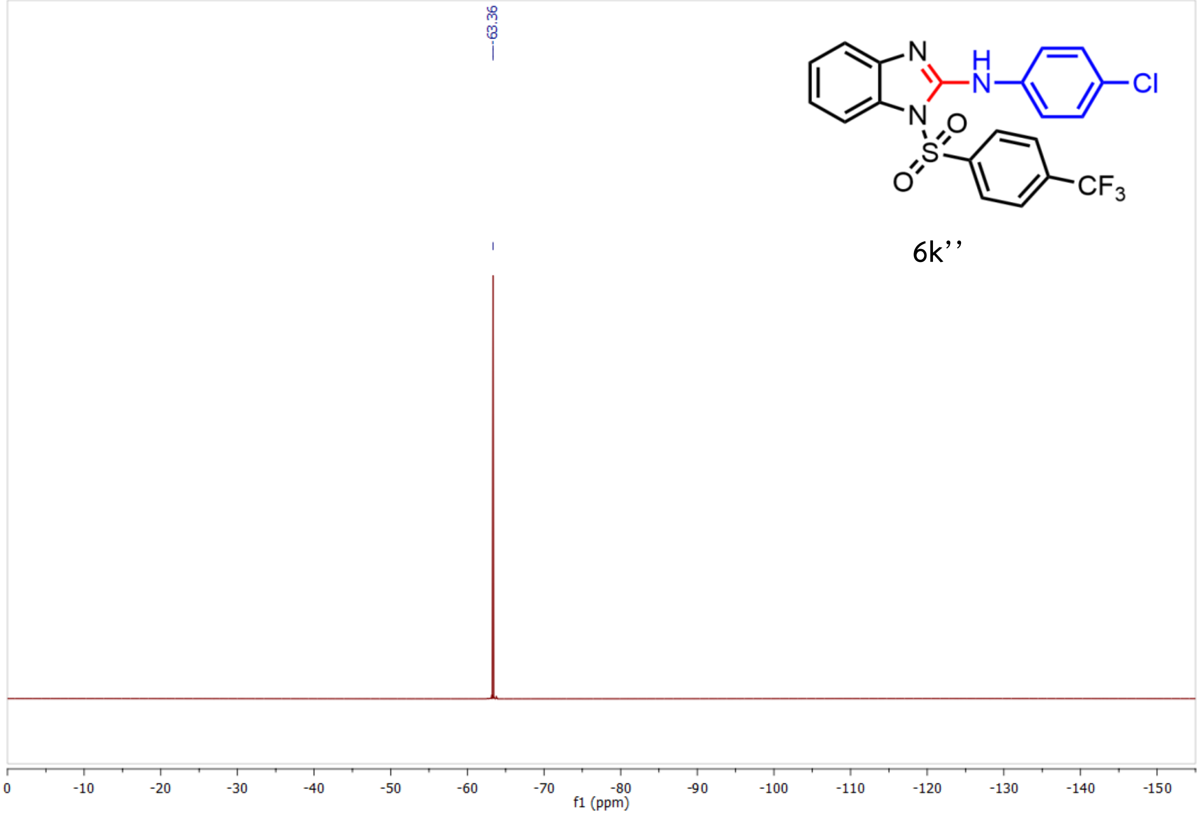


**Figure S164** $^{19}$F-NMR spectrum of **6k''** ($CDCl_3$, 471 MHz)

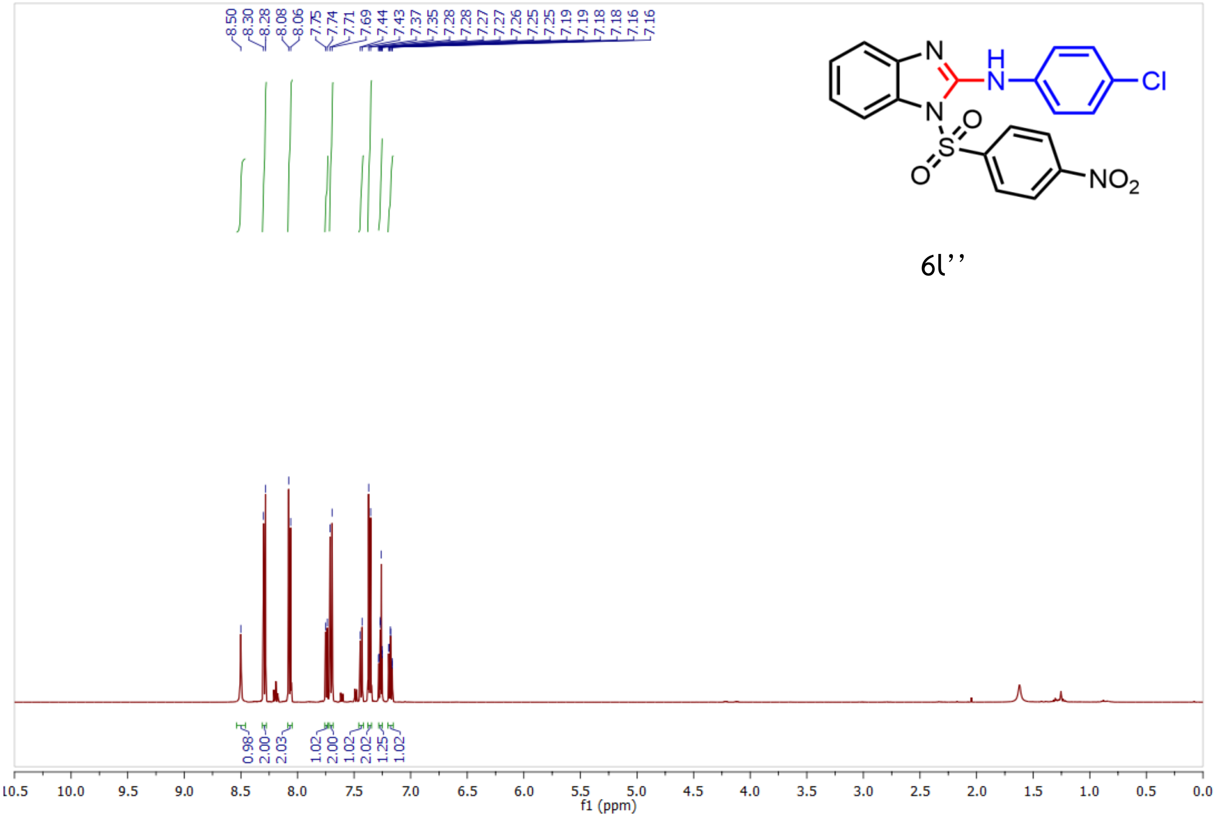


**Figure S165** $^{1}$H-NMR spectrum of **6l''** ($CDCl_3$, 500 MHz)

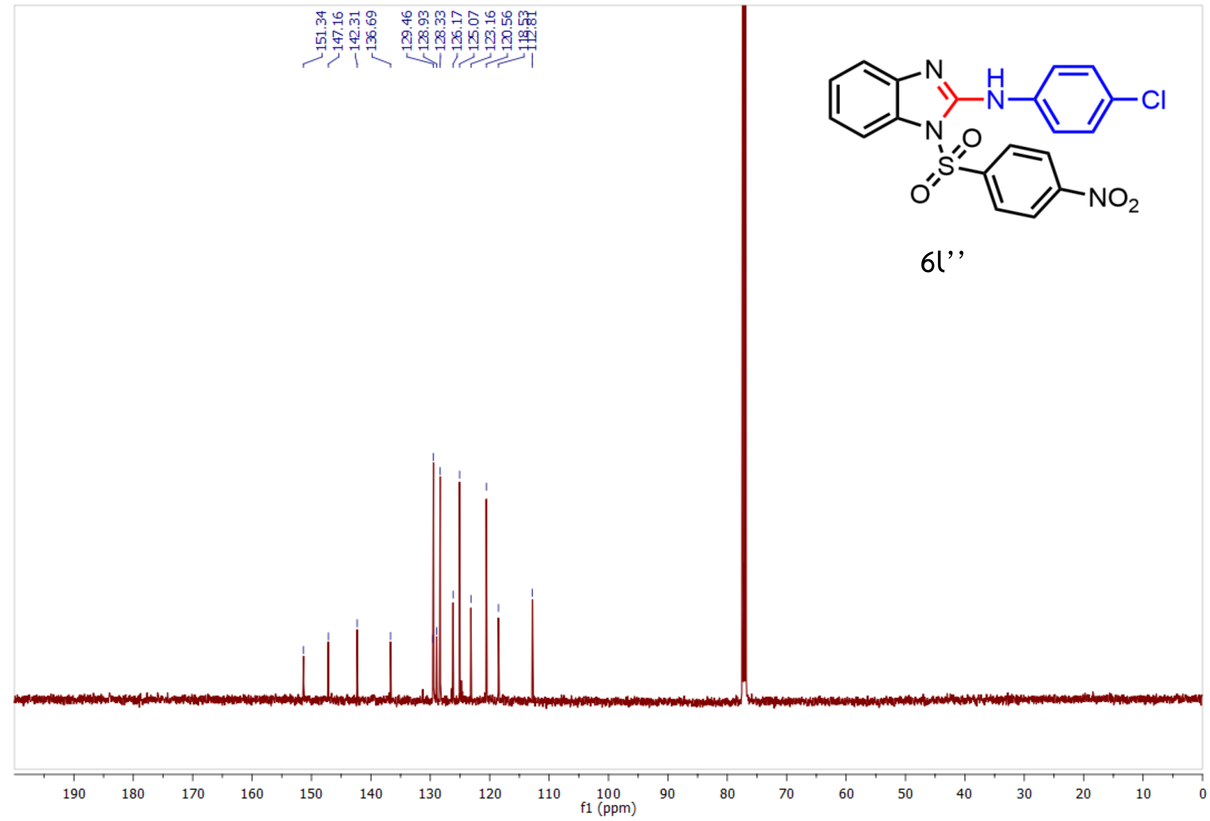


**Figure S166** $^{13}$C-NMR spectrum of **6l''** ($CDCl_3$, 126 MHz)

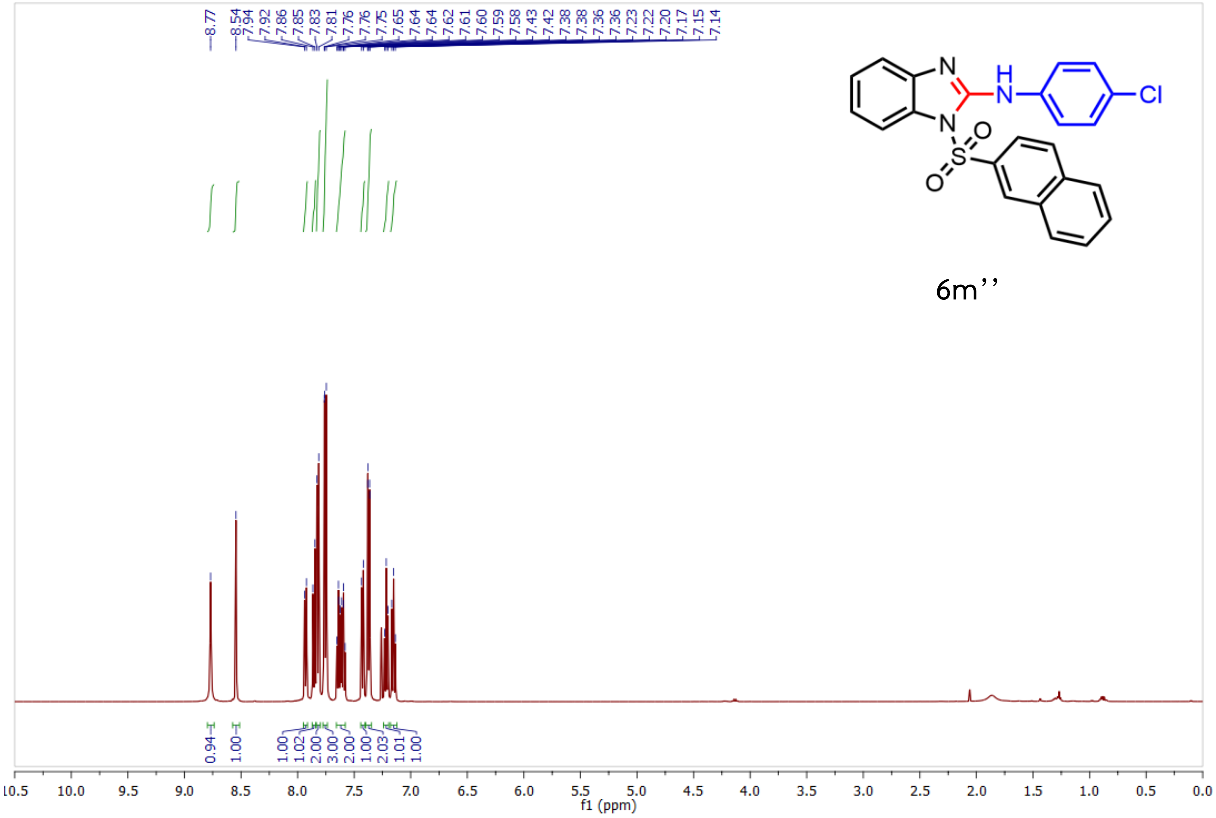


**Figure S167** $^{1}$H-NMR spectrum of **6m''** ($CDCl_3$, 500 MHz)

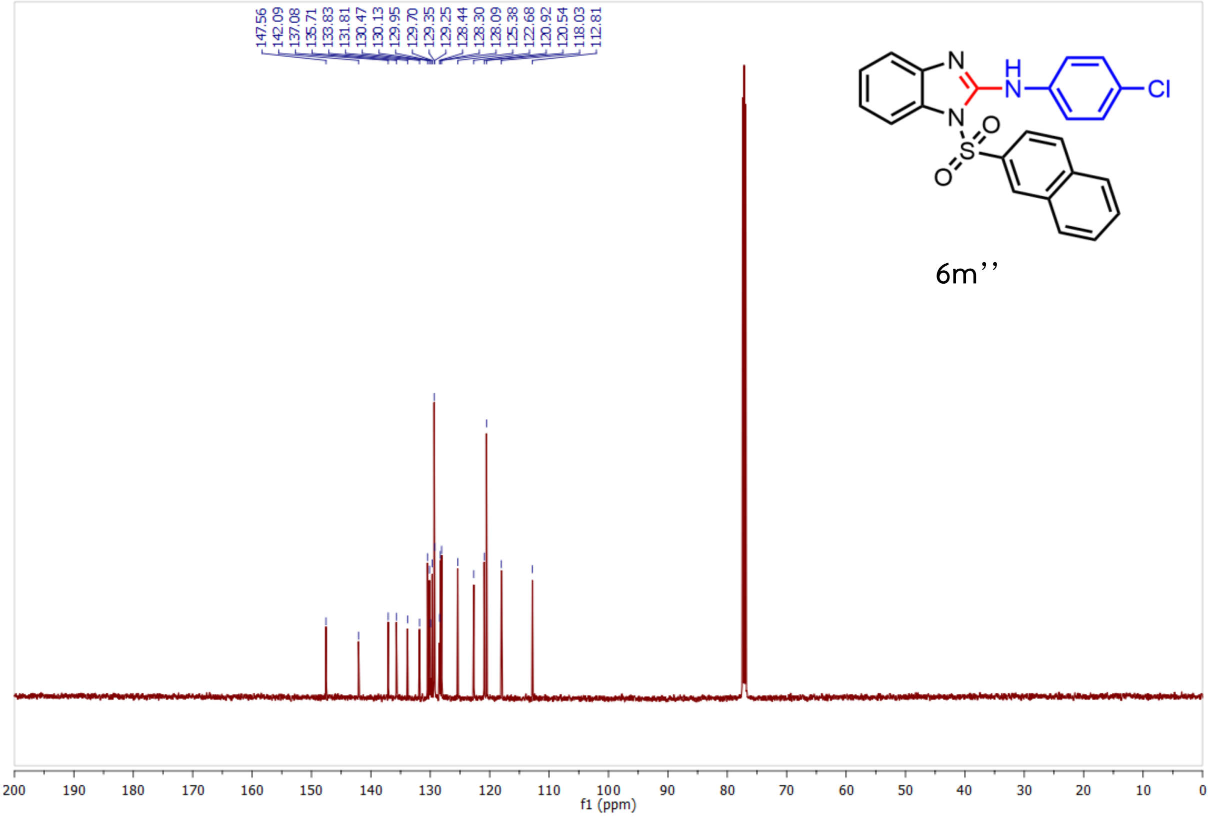


**Figure S168** $^{13}$C-NMR spectrum of **6m''** ($CDCl_3$, 126 MHz)

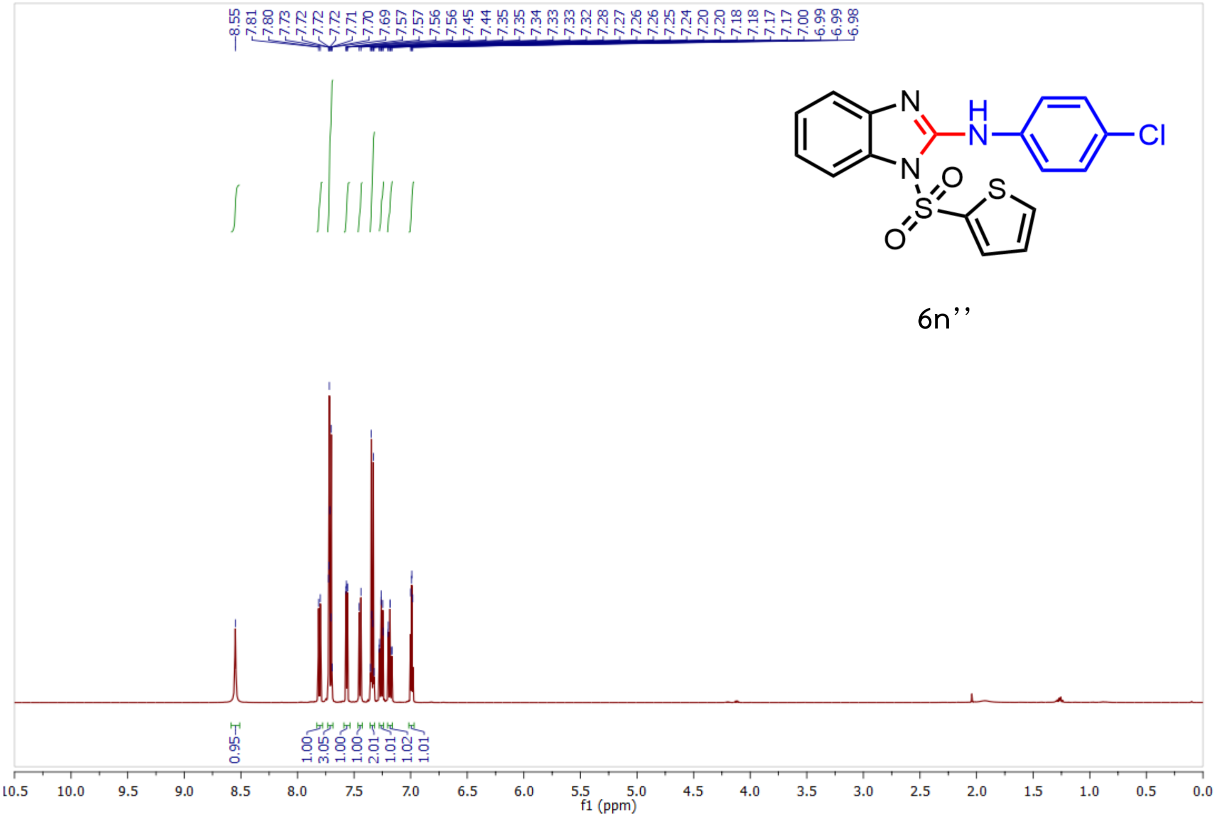


**Figure S169** $^{1}$H-NMR spectrum of **6n''** ($CDCl_3$, 500 MHz)

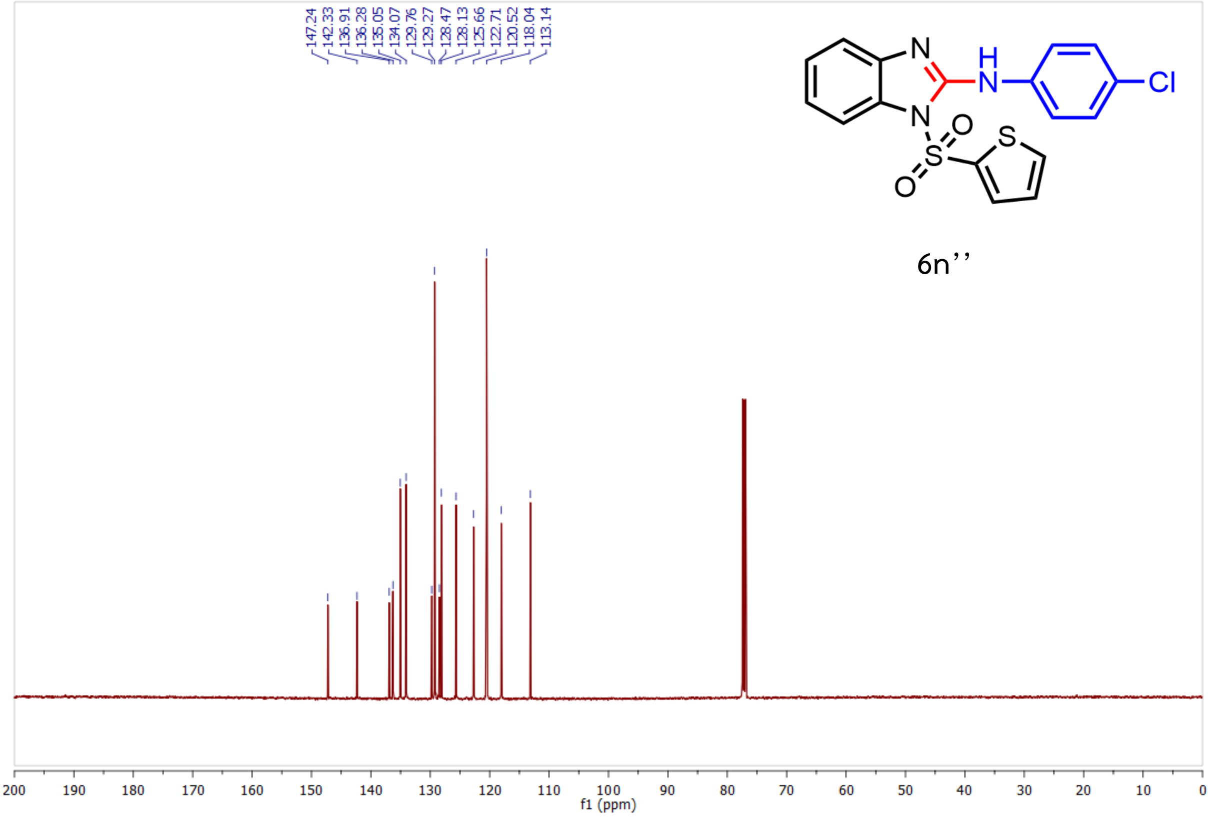


**Figure S170** $^{13}$C-NMR spectrum of **6n''** ($CDCl_3$, 126 MHz)

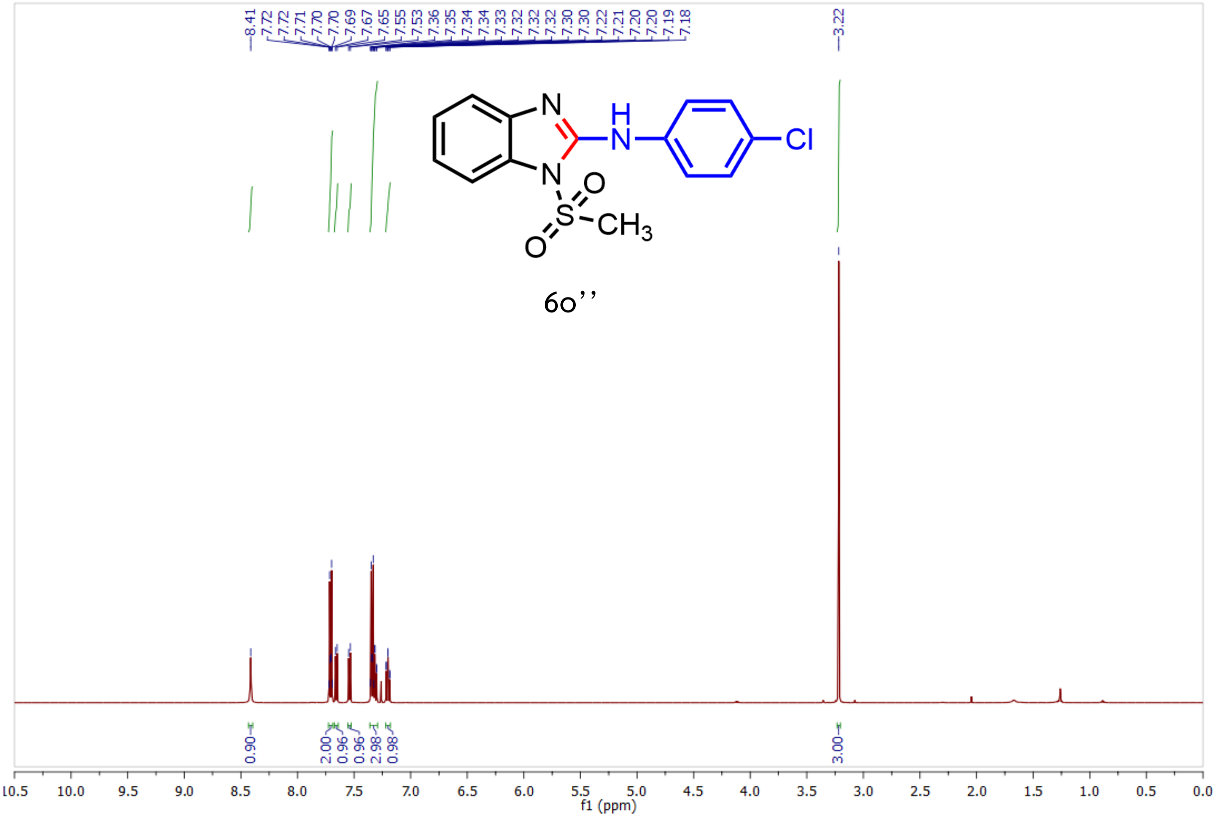

**Figure S171** $^{1}$H-NMR spectrum of **6o''** ($CDCl_3$, 500 MHz)

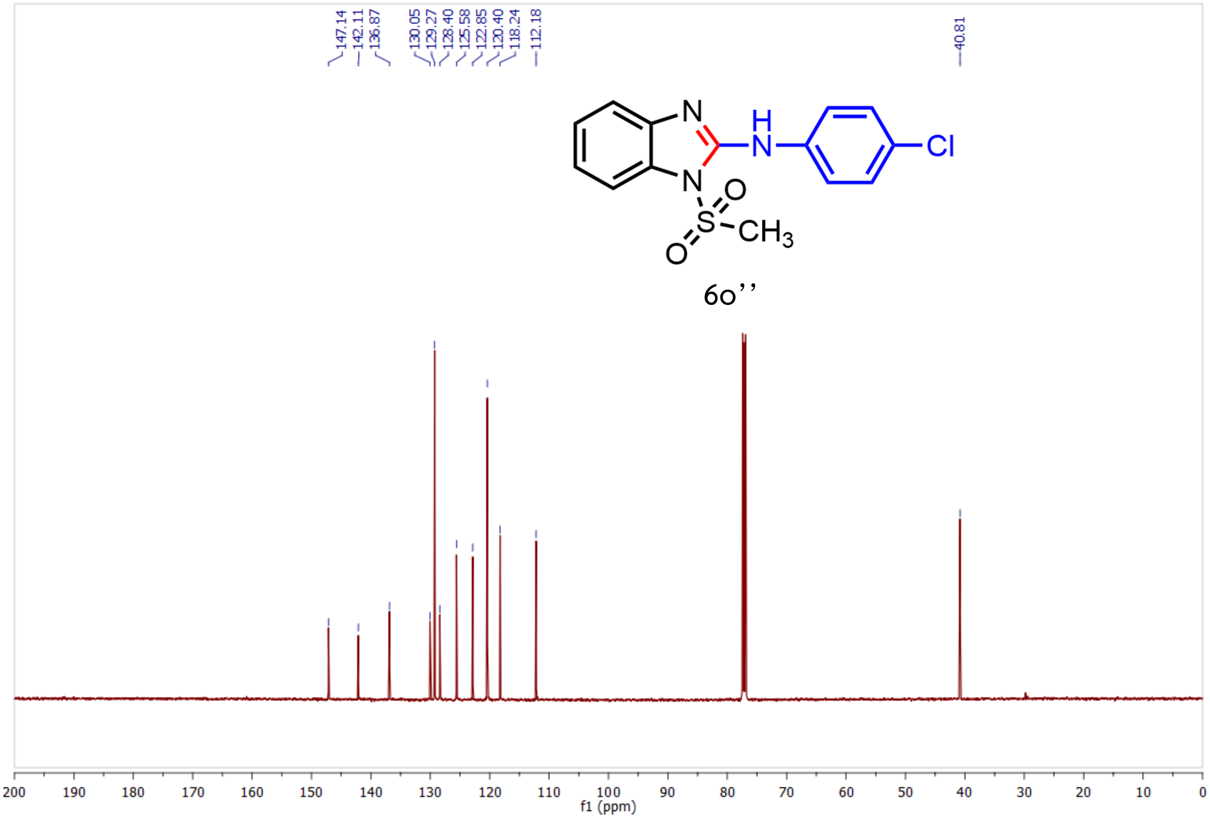

**Figure S172** $^{13}$C-NMR spectrum of **6o''** ($CDCl_3$, 126 MHz)

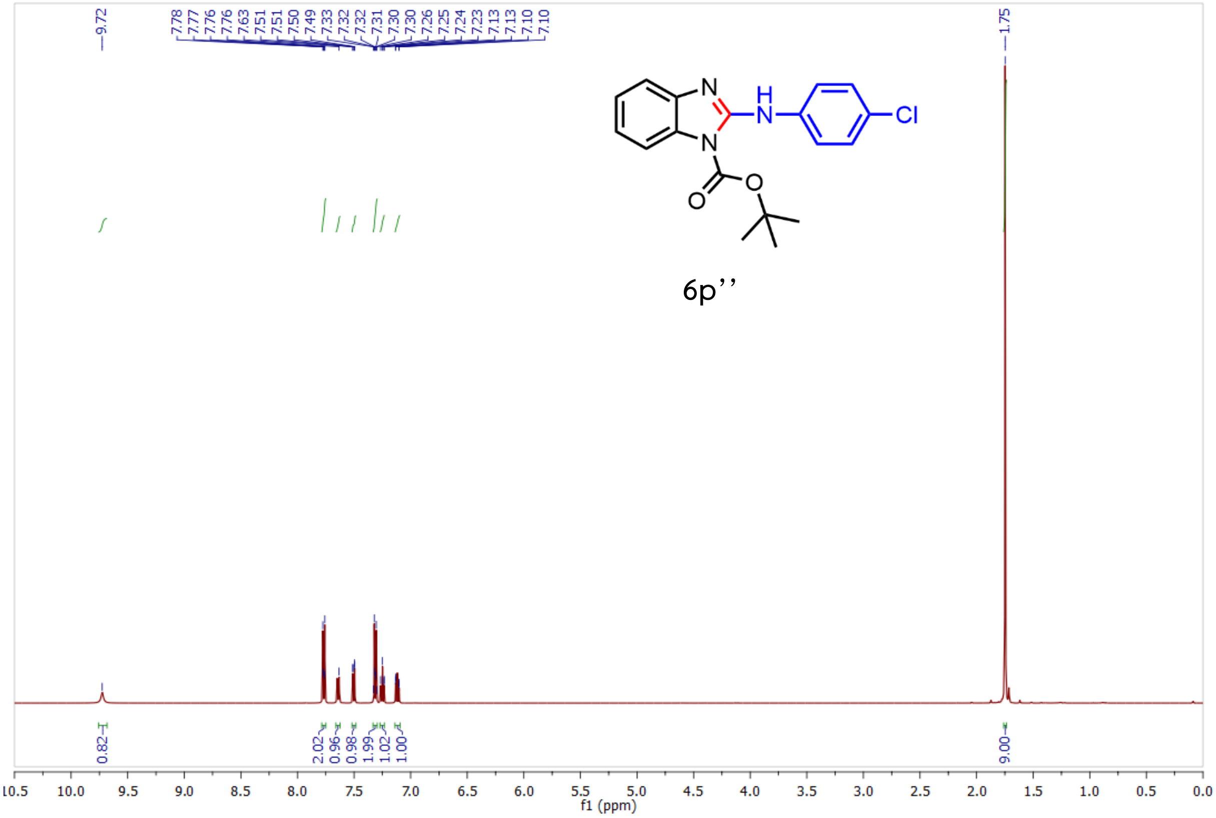

Figure S173 $^{1}$H-NMR spectrum of **6p''** ($CDCl_3$, 500 MHz)

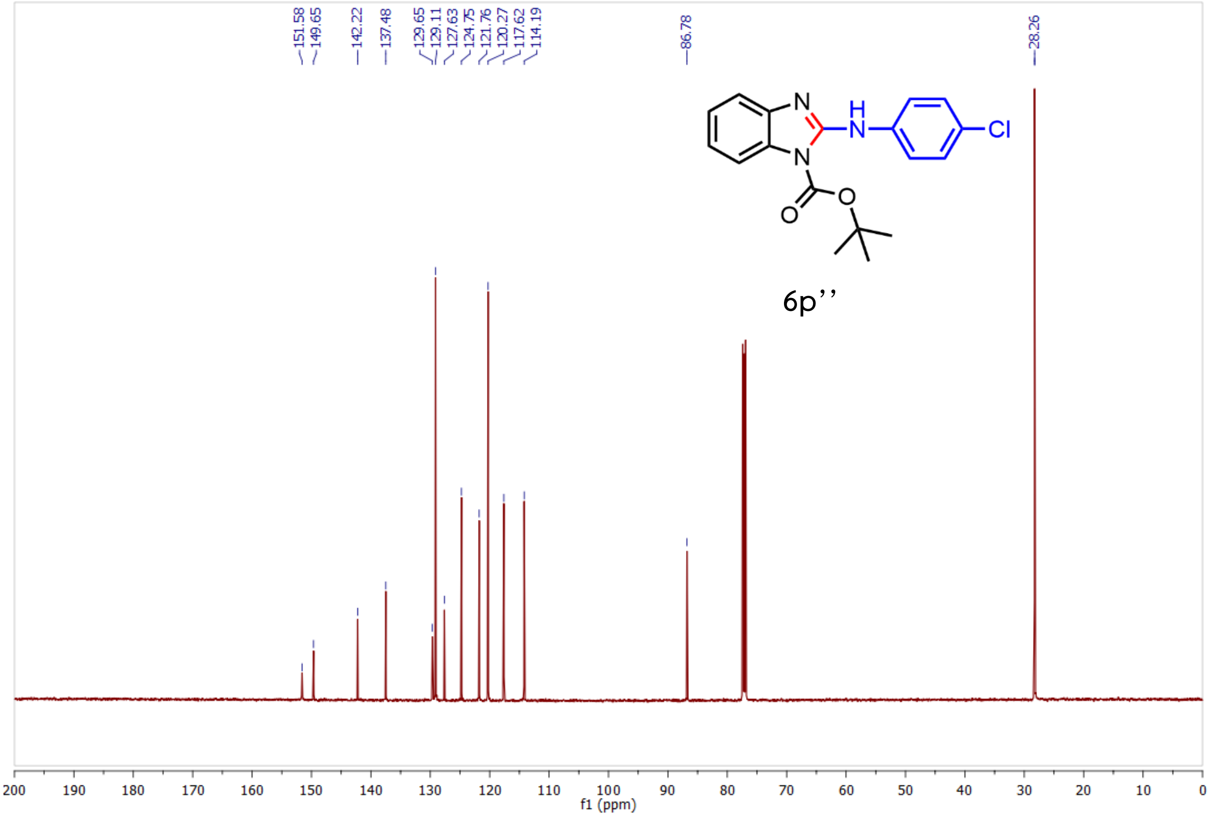

Figure S174 $^{13}$C-NMR spectrum of **6p''** ($CDCl_3$, 126 MHz)

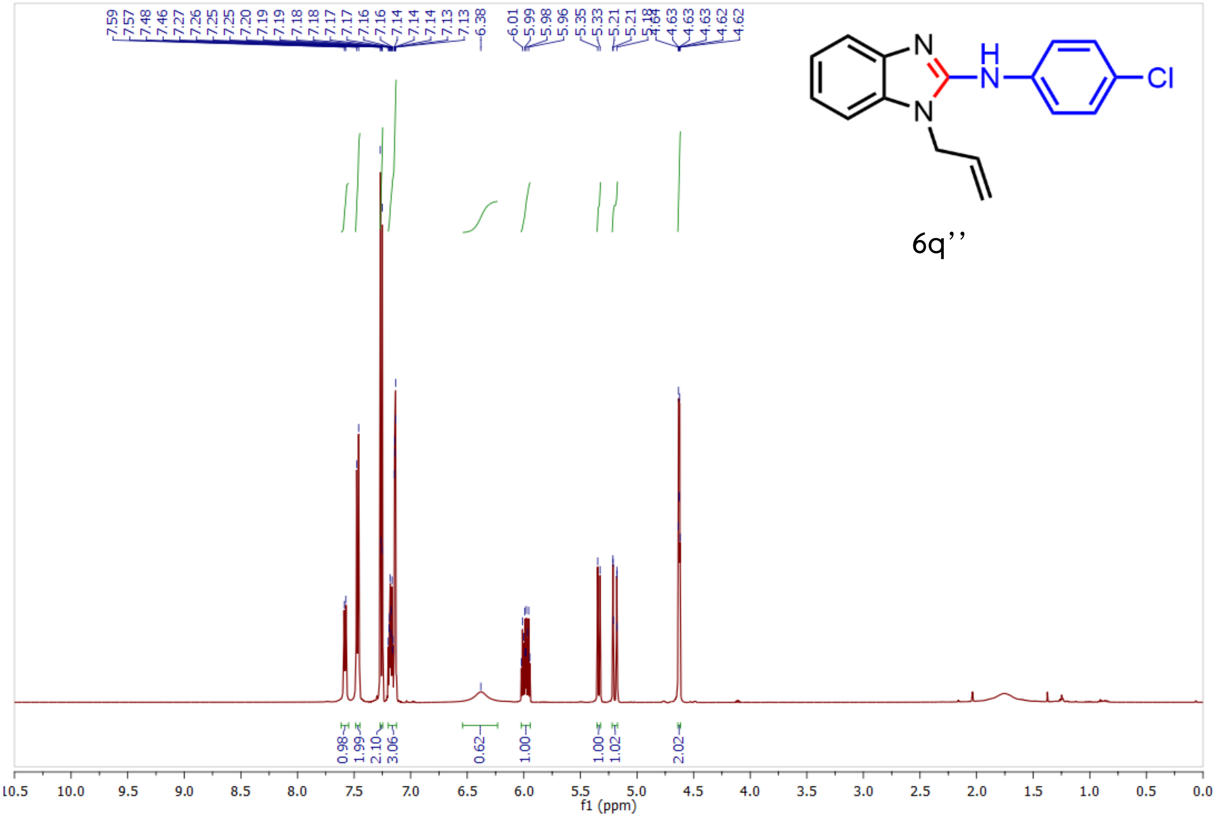

**Figure S175** $^{1}$H-NMR spectrum of **6q''** ($CDCl_3$, 500 MHz)

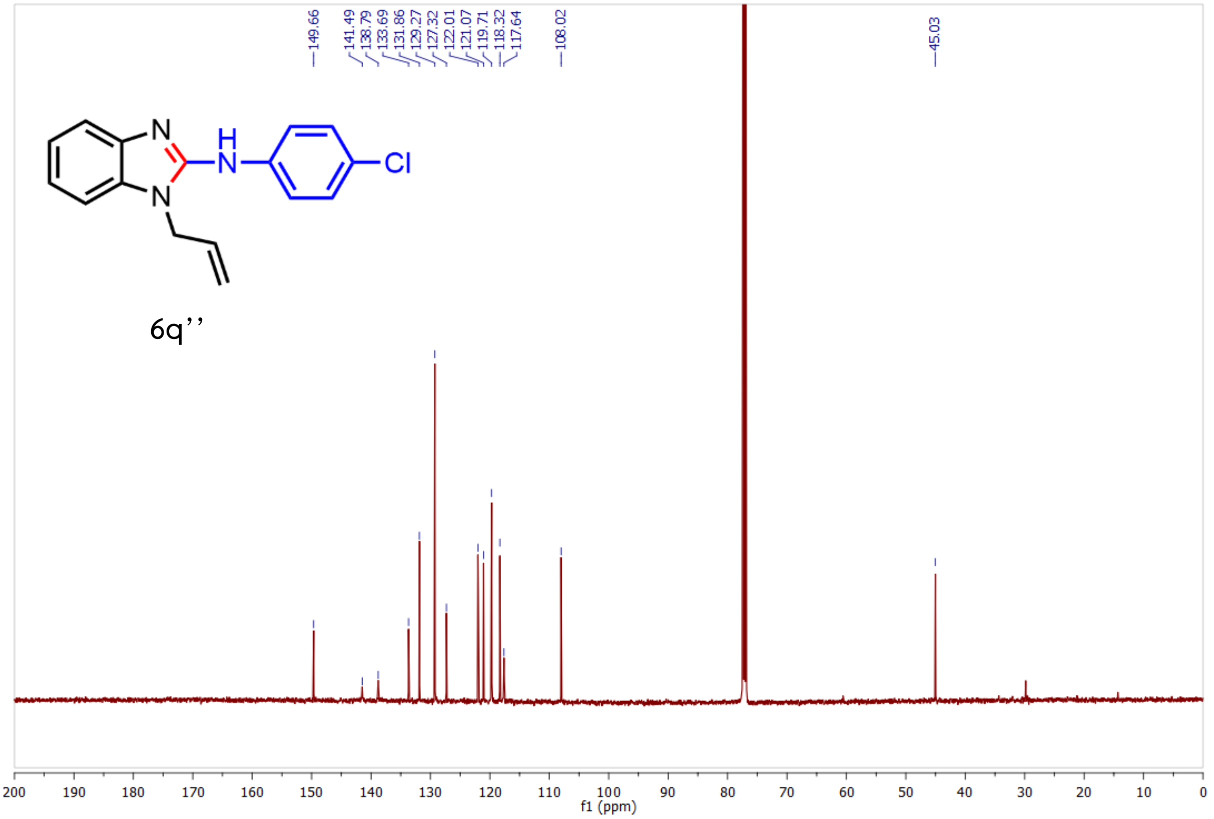

**Figure S176** $^{13}$C-NMR spectrum of **6q''** ($CDCl_3$, 126 MHz)

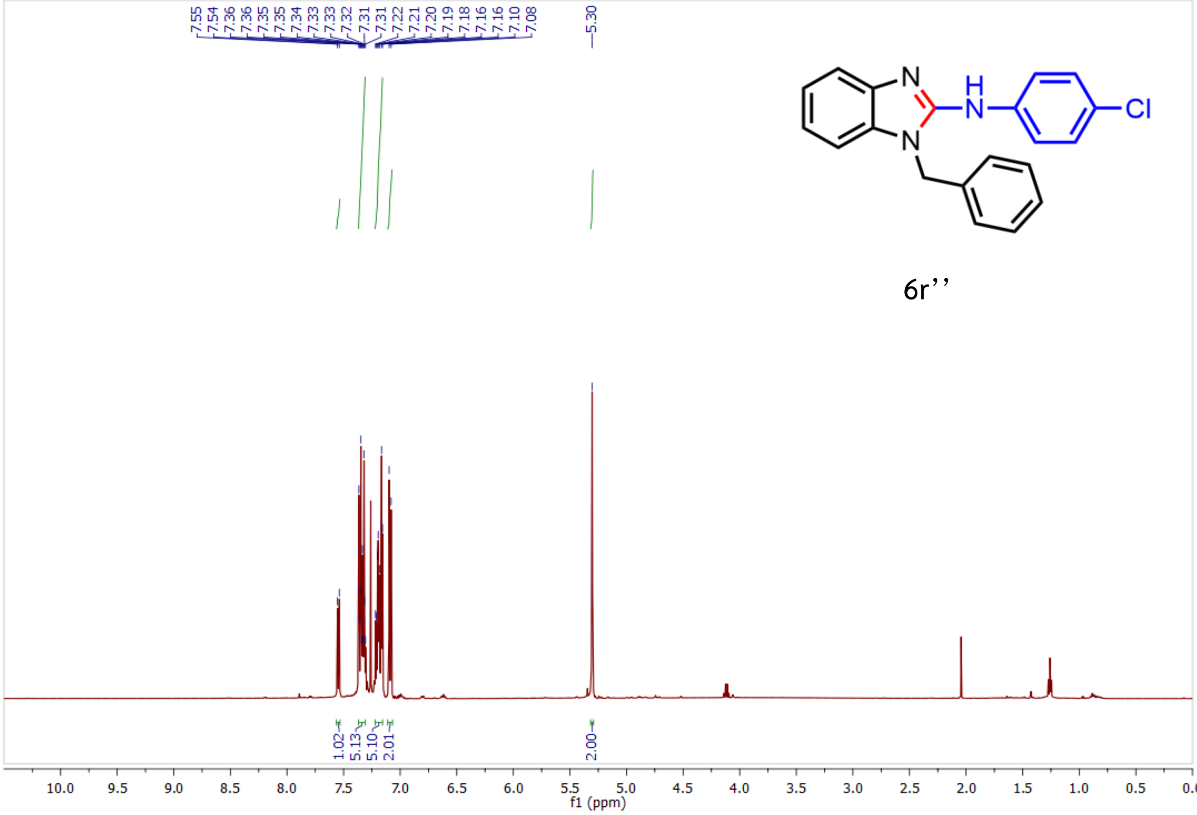


**Figure S177** $^{1}$H-NMR spectrum of **6r''** ($CDCl_3$, 500 MHz)

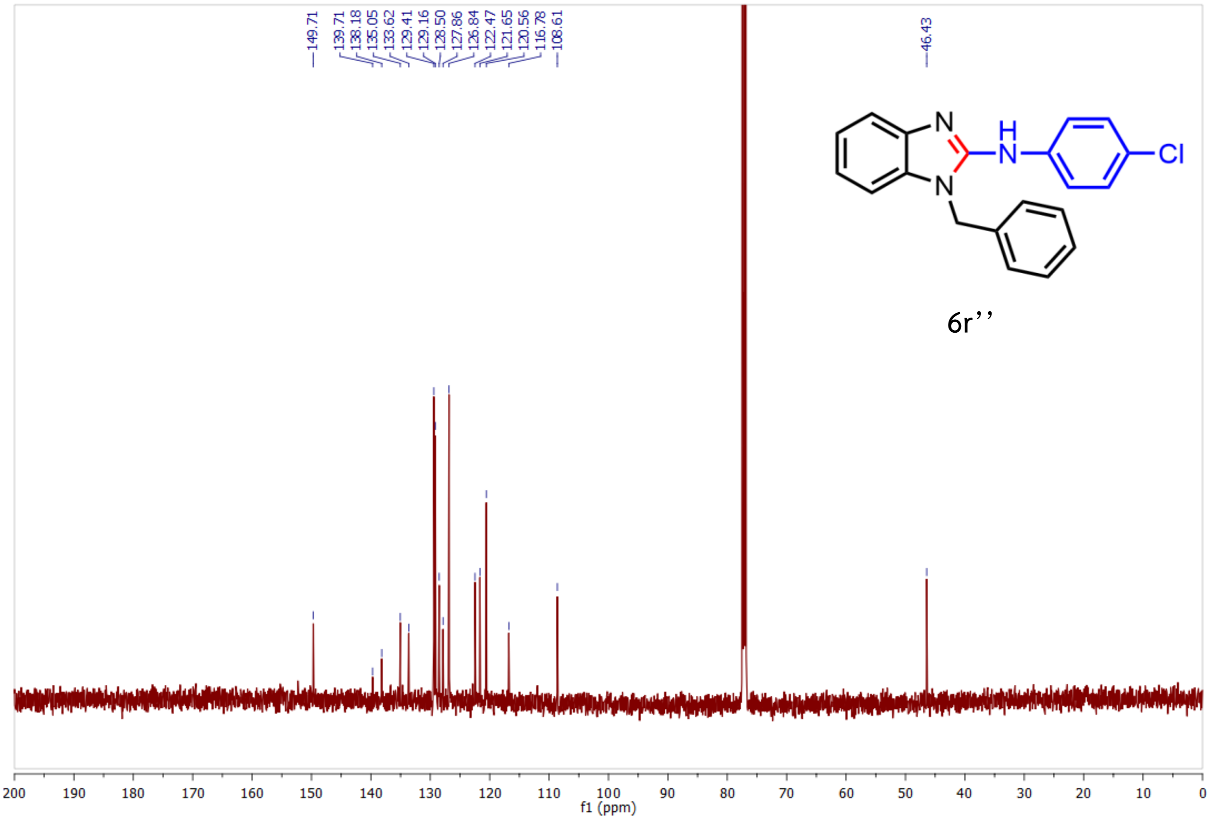


**Figure S178** $^{13}$C-NMR spectrum of **6r''** ($CDCl_3$, 126 MHz)

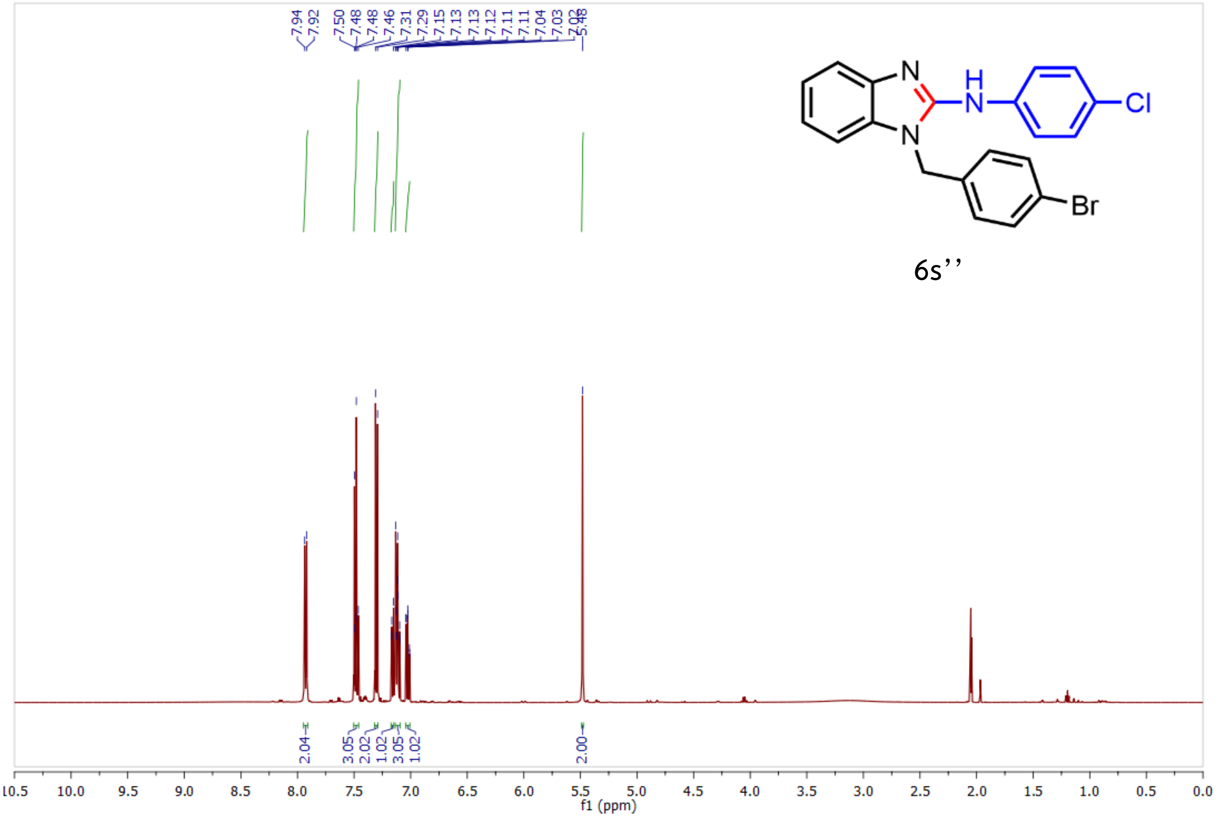


**Figure S179** $^{1}$H-NMR spectrum of **6s''** (acetone-$d_6$, 500 MHz)

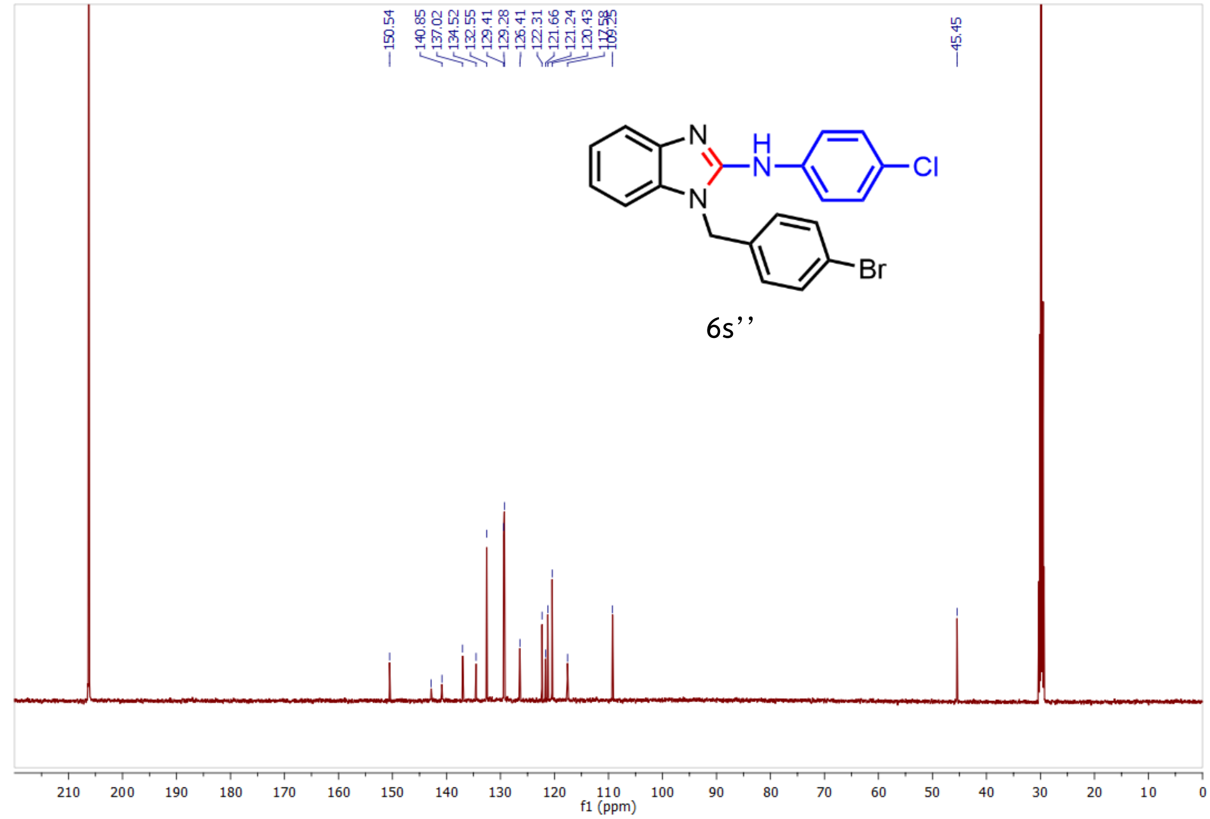


**Figure S180** $^{13}$C-NMR spectrum of **6s''** (acetone-$d_6$, 126 MHz)

# Generic Display Report

**Analysis Info**

| | | | |
|---|---|---|---|
| Analysis Name | D:\Data\Data Service\221219\NHTs_RE2_01_6654.d | Acquisition Date | 12/19/2022 4:00:28 PM |
| Method | nv_pos_5min_profile_190214.m | Operator | CU. |
| Sample Name | NHTs | Instrument | micrOTOF-Q |
| Comment | | | |

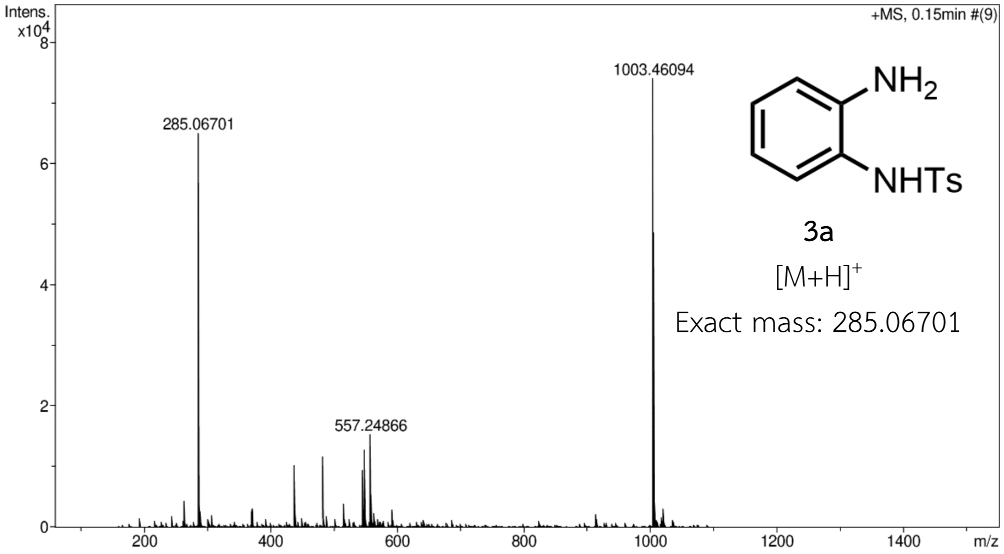


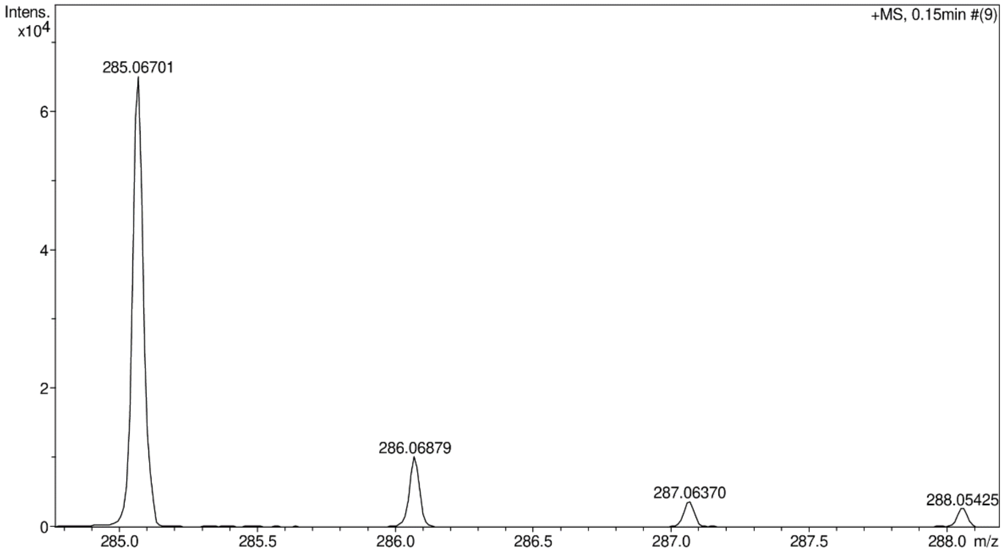


Bruker Compass DataAnalysis 4.0 printed: 12/19/2022 8:38:25 PM Page 1 of 1

**Figure S181** ESI-HRMS spectrum of **3a**

# Generic Display Report

**Analysis Info**

| | | | |
|---|---|---|---|
| Analysis Name | D:\Data\Data Service\230925\Ts-Cl-thiourea_RB3_01_7741.d | Acquisition Date | 9/25/2023 4:57:11 PM |
| Method | nv_pos_5min_profile_190214.m | Operator | CU. |
| Sample Name | Ts-Cl-thiourea | Instrument | micrOTOF-Q |
| Comment | | | |

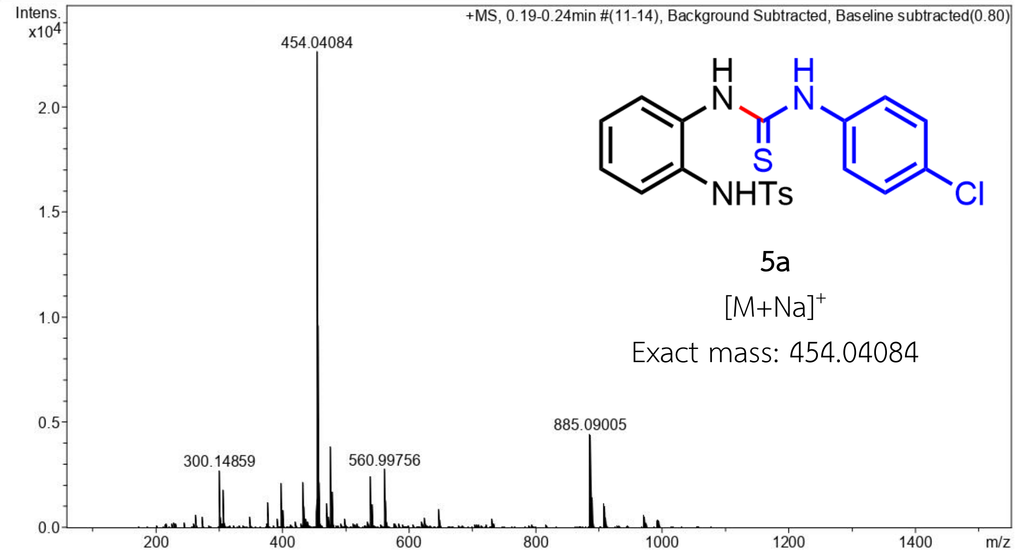


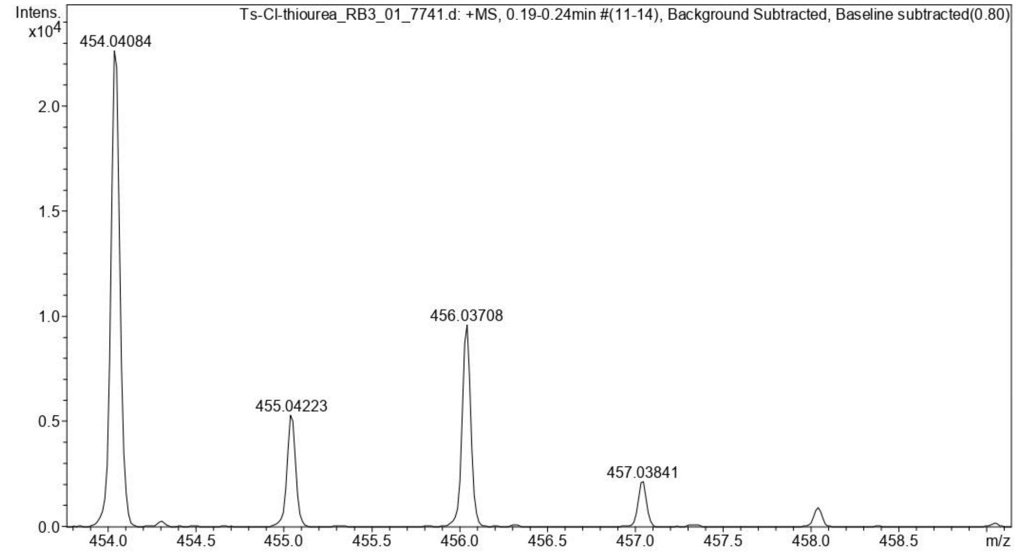


Bruker Compass DataAnalysis 4.0 printed: 9/25/2023 6:23:55 PM Page 1 of 1

**Figure S182** ESI-HRMS spectrum of **5a**

# Generic Display Report

| Analysis Info | | Acquisition Date | 3/4/2024 3:05:35 PM |
|---|---|---|---|
| Analysis Name | D:\Data\Data Service\240304\NHTs+4-Cl-NCO-Urea_RB2_01_9044.d | | |
| Method | nv_pos_5min_profile_190214.m | Operator | CU. |
| Sample Name | NHTs+4-Cl-NCO-Urea | Instrument | micrOTOF-Q |
| Comment | | | |

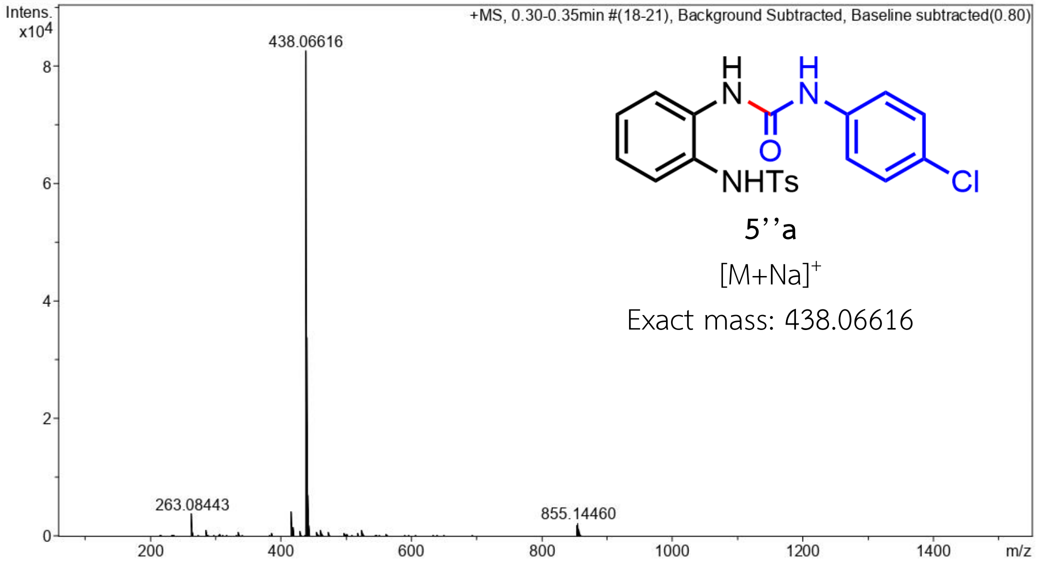


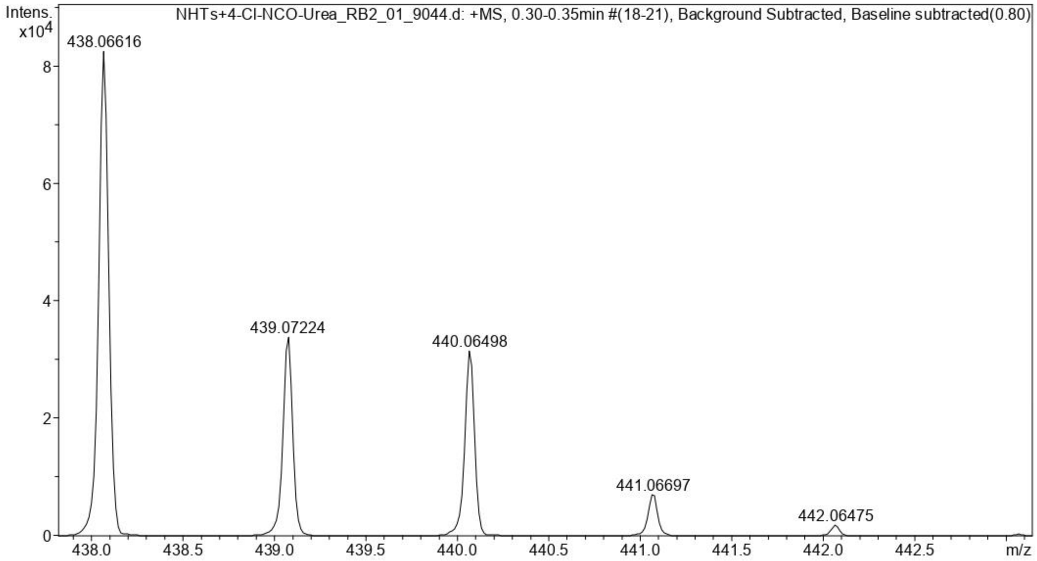


Bruker Compass DataAnalysis 4.0 printed: 3/4/2024 3:13:50 PM Page 1 of 1

**Figure S183** ESI-HRMS spectrum of **5’’a**

## Generic Display Report

**Analysis Info**

Analysis Name: D:\Data\Data Service\221219\NHTs+4-Cl-NCS_RA4_01_6623.d
Method: nv_pos_5min_profile_190214.m
Sample Name: NHTs+4-Cl-NCS
Comment:

Acquisition Date: 12/19/2022 12:40:26 PM
Operator: CU.
Instrument: micrOTOF-Q

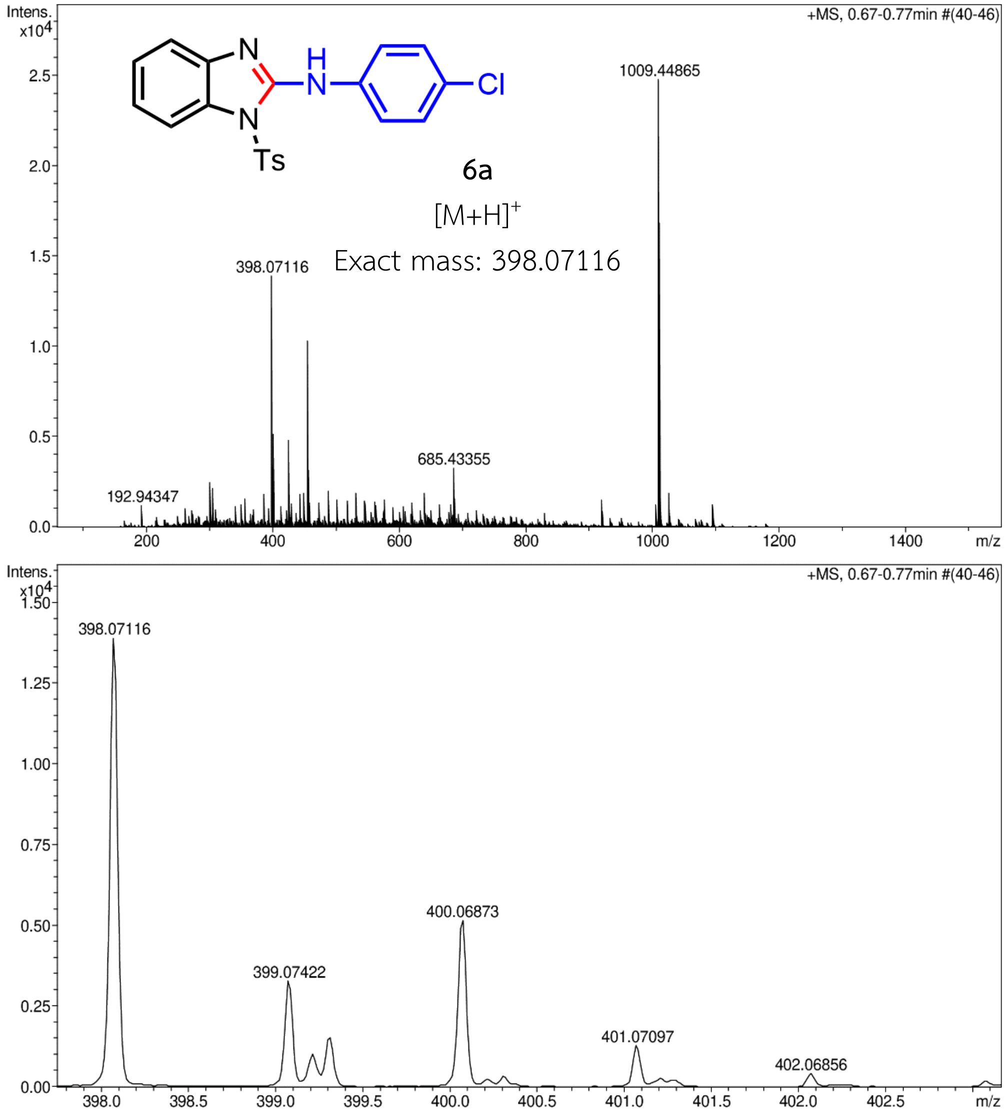


Bruker Compass DataAnalysis 4.0 printed: 12/19/2022 6:05:35 PM Page 1 of 1

**Figure S184** ESI-HRMS spectrum of **6a**

## Generic Display Report

**Analysis Info**

| | | | |
|---|---|---|---|
| Analysis Name | D:\Data\Data Service\221219\NHTs+2-Cl-NCS_RC5_01_6640.d | Acquisition Date | 12/19/2022 2:30:13 PM |
| Method | nv_pos_5min_profile_190214.m | Operator | CU. |
| Sample Name | NHTs+2-Cl-NCS | Instrument | micrOTOF-Q |
| Comment | | | |

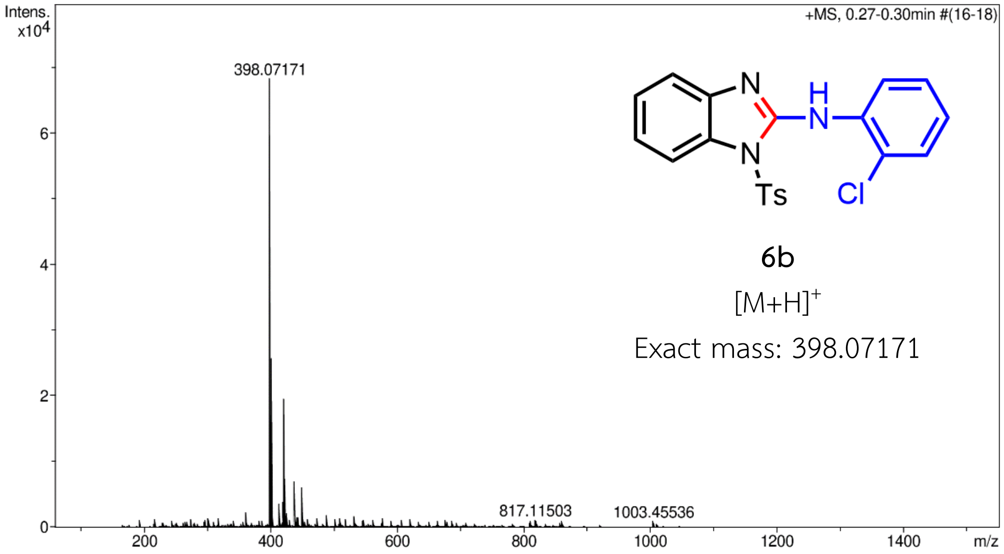


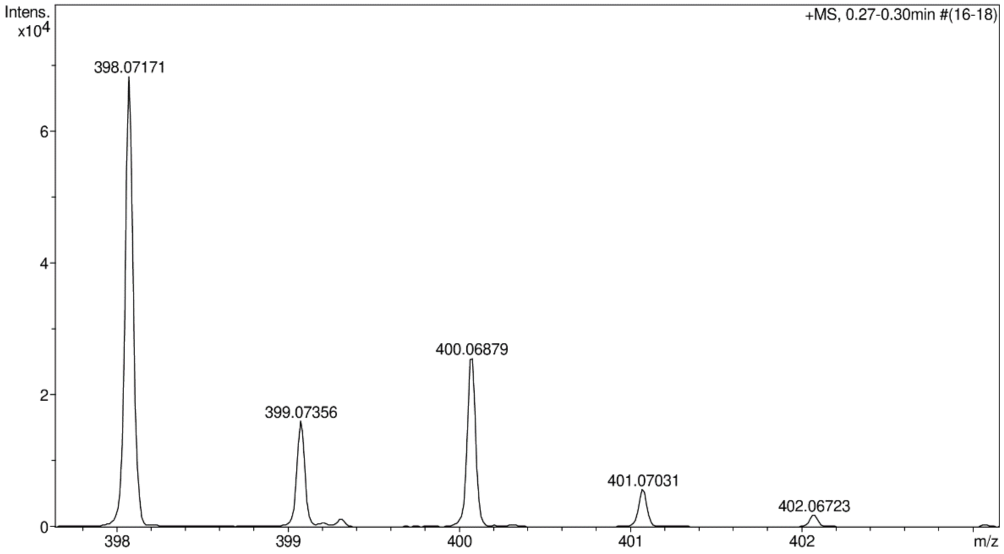


Bruker Compass DataAnalysis 4.0 printed: 12/19/2022 7:18:15 PM Page 1 of 1

**Figure S185** ESI-HRMS spectrum of **6b**

## Generic Display Report

| Analysis Info | | Acquisition Date | 7/24/2023 12:40:08 PM |
|---|---|---|---|
| Analysis Name | D:\Data\Data Service\230724\light 2Cl 4Cl_RA3_01_7297.d | | |
| Method | nv_pos_5min_profile_190214.m | Operator | CU. |
| Sample Name | light 2Cl 4Cl | Instrument | micrOTOF-Q |
| Comment | | | |

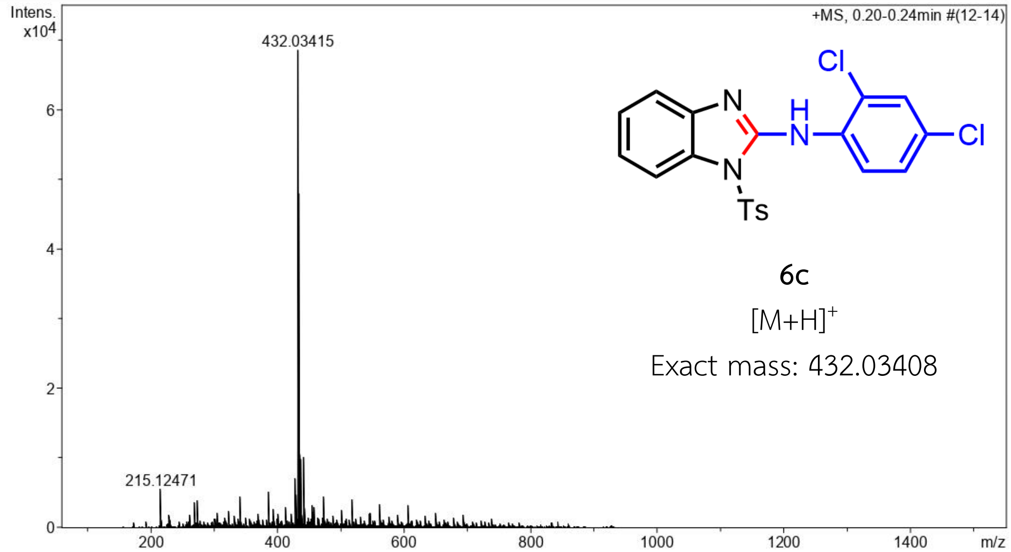


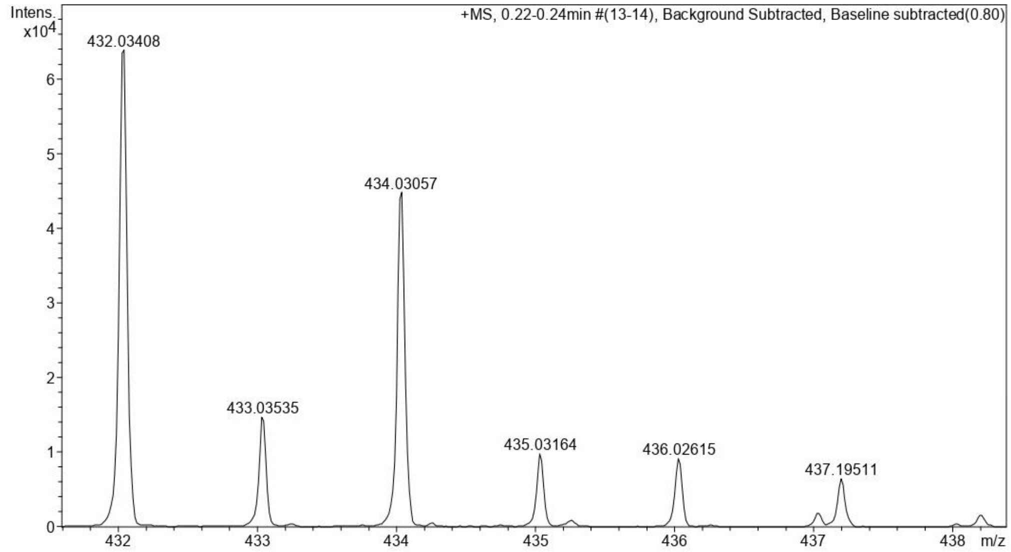


Bruker Compass DataAnalysis 4.0 printed: 7/24/2023 1:33:25 PM Page 1 of 1

**Figure S186** ESI-HRMS spectrum of **6c**

## Generic Display Report

| Analysis Info | | Acquisition Date | 12/19/2022 1:06:17 PM |
|---|---|---|---|
| Analysis Name | D:\Data\Data Service\221219\NHTs+4-F-NCS_RA8_01_6627.d | | |
| Method | nv_pos_5min_profile_190214.m | Operator | CU. |
| Sample Name | NHTs+4-F-NCS | Instrument | micrOTOF-Q |
| Comment | | | |

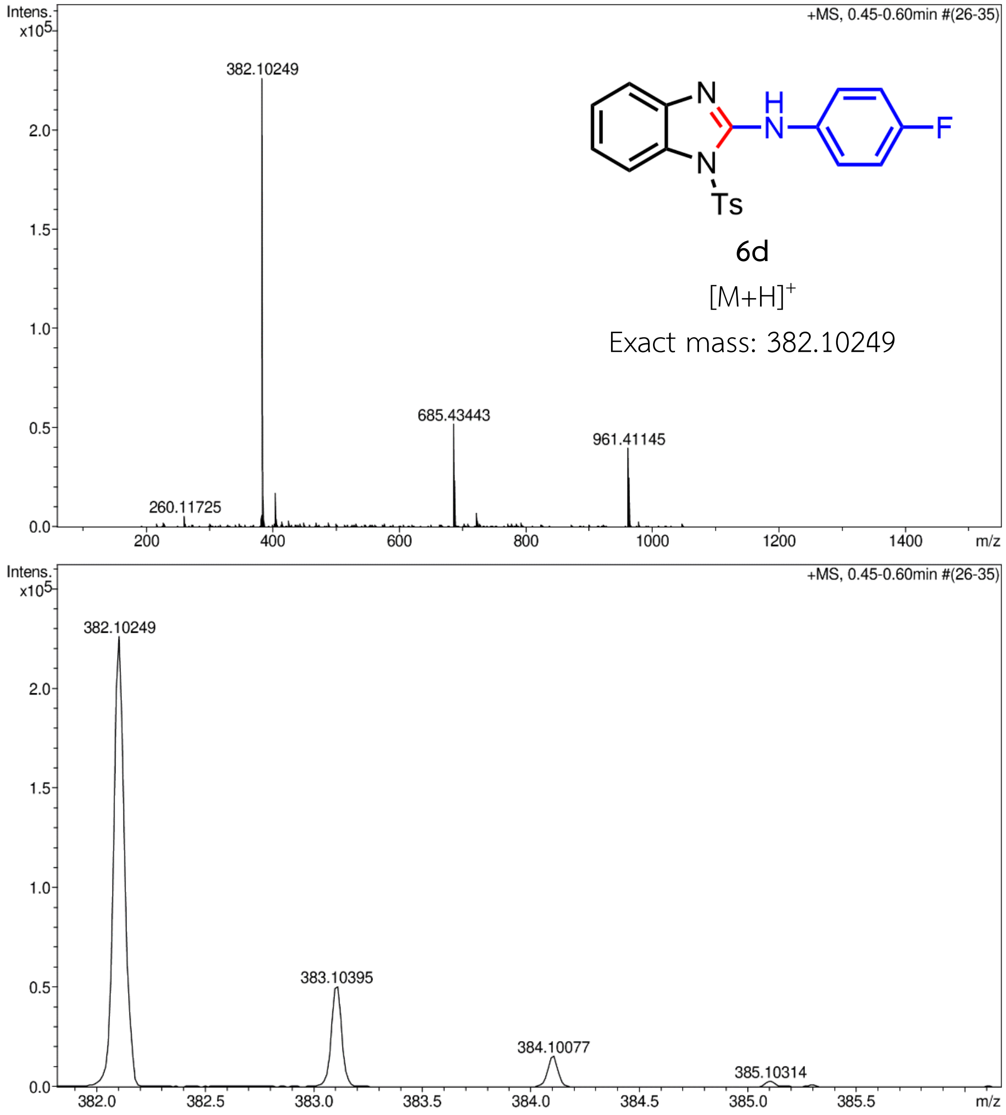


Bruker Compass DataAnalysis 4.0 printed: 12/19/2022 6:22:09 PM Page 1 of 1

**Figure S187** ESI-HRMS spectrum of **6d**

# Generic Display Report

| | | | |
|---|---|---|---|
| **Analysis Info** | | Acquisition Date | 12/19/2022 2:17:19 PM |
| Analysis Name | D:\Data\Data Service\221219\NHTs+2-F-NCS_RC3_01_6638.d | | |
| Method | nv_pos_5min_profile_190214.m | Operator | CU. |
| Sample Name | NHTs+2-F-NCS | Instrument | micrOTOF-Q |
| Comment | | | |

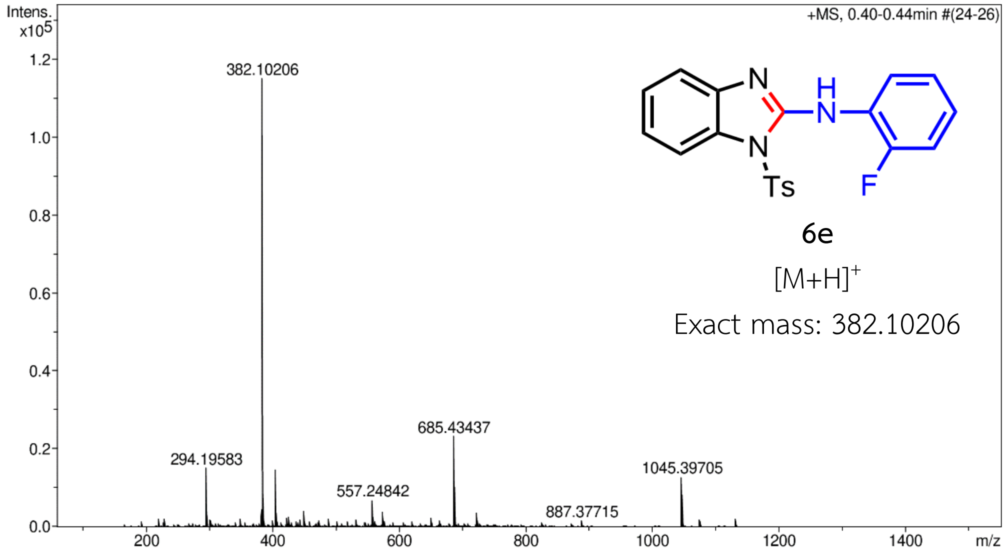


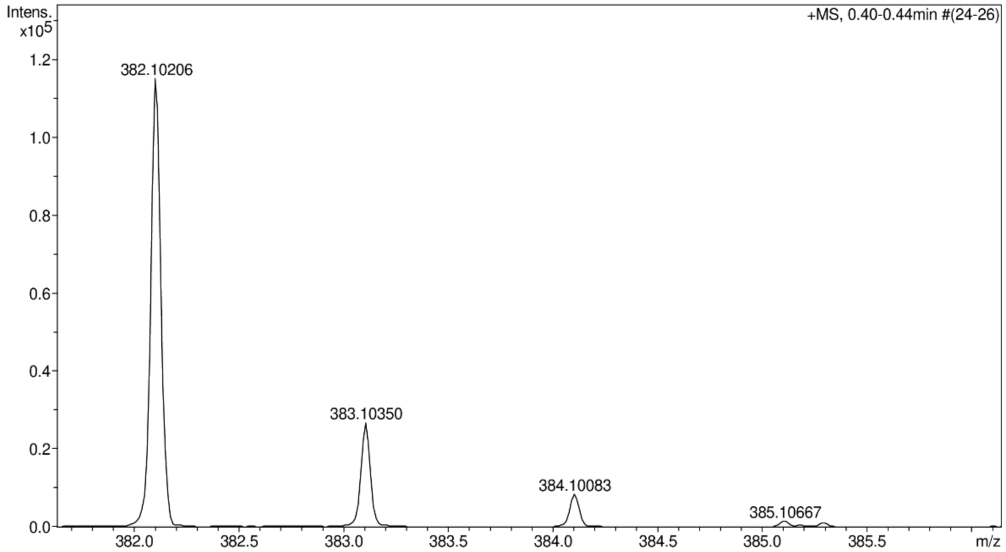


Bruker Compass DataAnalysis 4.0 printed: 12/19/2022 7:11:15 PM Page 1 of 1

**Figure S188** ESI-HRMS spectrum of **6e**

## Generic Display Report

| | | | |
|---|---|---|---|
| **Analysis Info** | | Acquisition Date | 7/24/2023 12:33:40 PM |
| Analysis Name | D:\Data\Data Service\230724\light 2F 4F_RA2_01_7296.d | | |
| Method | nv_pos_5min_profile_190214.m | Operator | CU. |
| Sample Name | light 2F 4F | Instrument | micrOTOF-Q |
| Comment | | | |

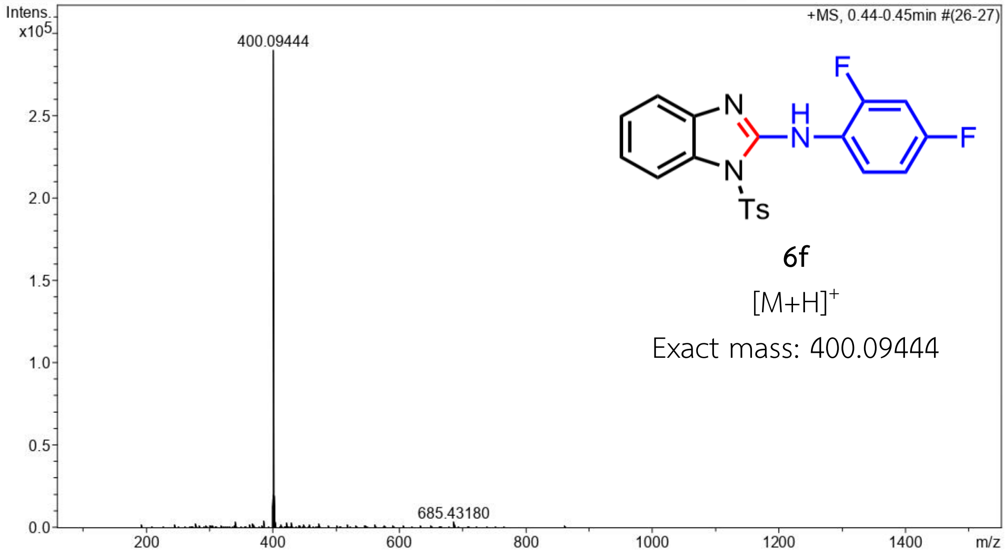


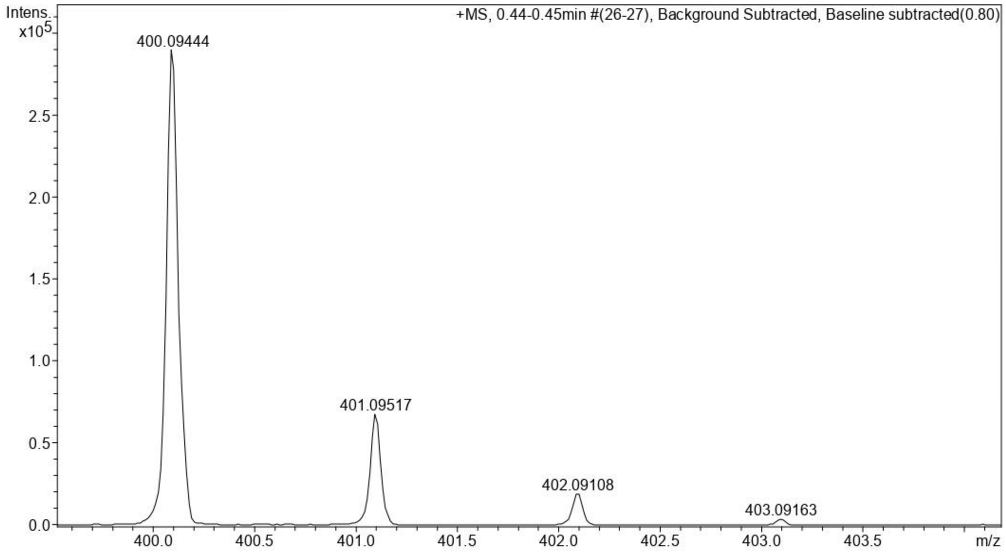


Bruker Compass DataAnalysis 4.0 printed: 7/24/2023 1:25:29 PM Page 1 of 1

**Figure S189** ESI-HRMS spectrum of **6f**

# Generic Display Report

| **Analysis Info** | | Acquisition Date | 12/19/2022 12:27:32 PM |
|---|---|---|---|
| Analysis Name | D:\Data\Data Service\221219\NHTs+4-Br-NCS_RA3_01_6621.d | | |
| Method | nv_pos_5min_profile_190214.m | Operator | CU. |
| Sample Name | NHTs+4-Br-NCS | Instrument | micrOTOF-Q |
| Comment | | | |

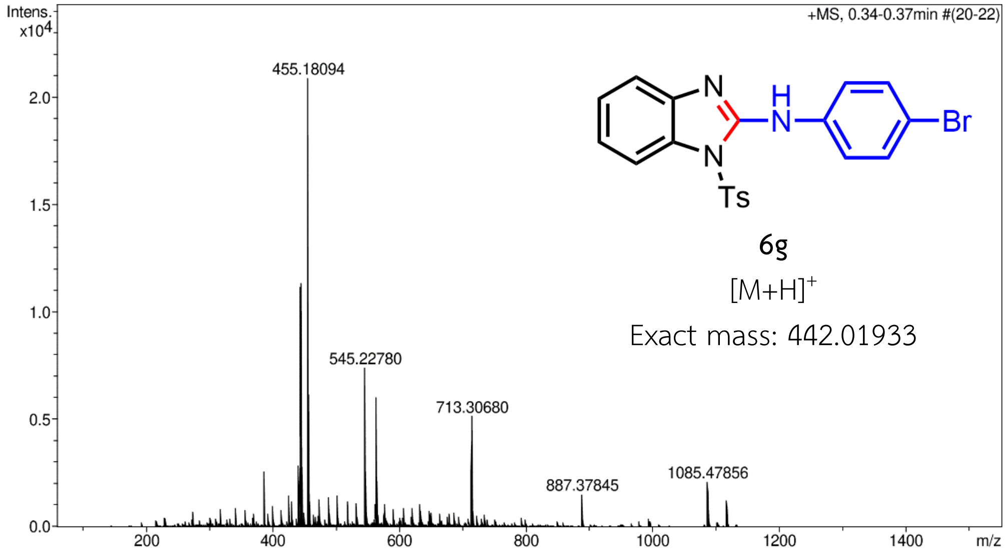


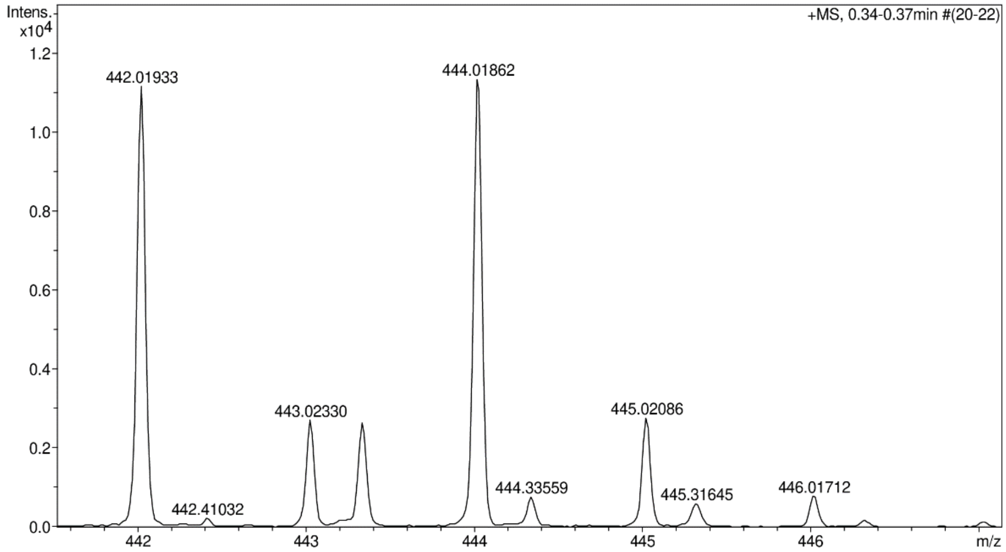




**Figure S190** ESI-HRMS spectrum of **6g**

## Generic Display Report

| Analysis Info | | Acquisition Date | 12/19/2022 3:34:38 PM |
|---|---|---|---|
| Analysis Name | D:\Data\Data Service\221219\NHTs+3-Br-NCS_RD6_01_6650.d | | |
| Method | nv_pos_5min_profile_190214.m | Operator | CU. |
| Sample Name | NHTs+3-Br-NCS | Instrument | micrOTOF-Q |
| Comment | | | |

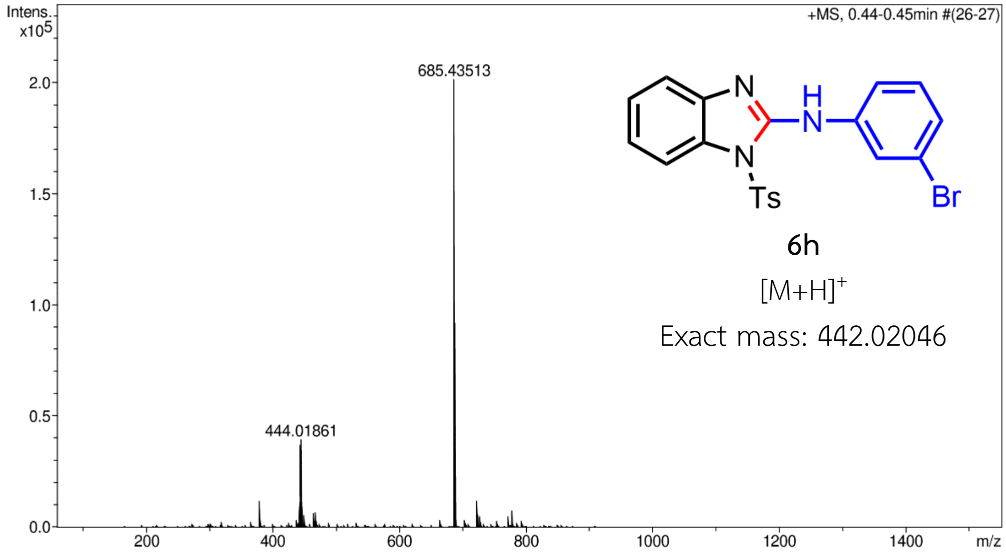


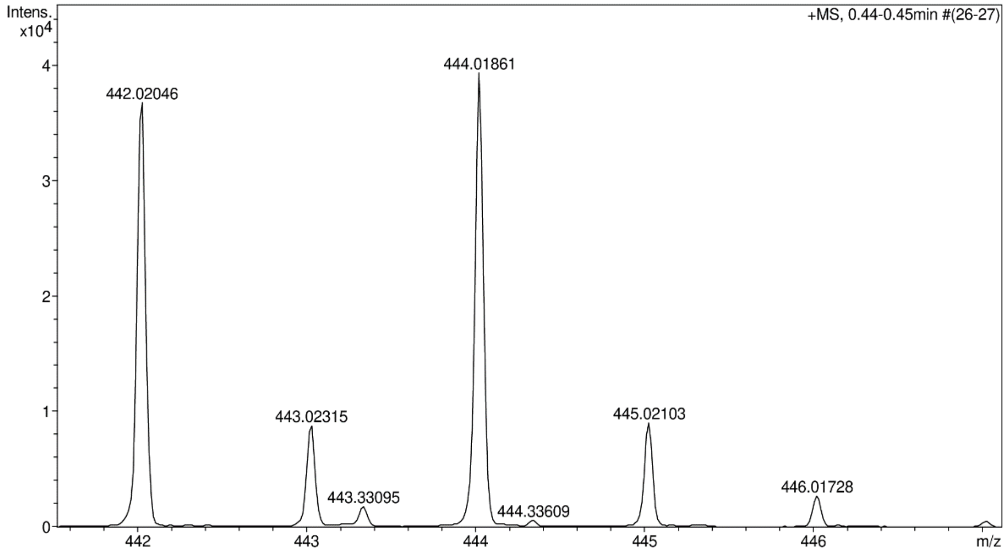


Bruker Compass DataAnalysis 4.0 printed: 12/19/2022 8:04:58 PM Page 1 of 1

**Figure S191** ESI-HRMS spectrum of **6h**

# Generic Display Report

**Analysis Info**

| | | | |
|---|---|---|---|
| Analysis Name | D:\Data\Data Service\221219\NHTs+4-I-NCS_RA7_01_6626.d | Acquisition Date | 12/19/2022 12:59:49 PM |
| Method | nv_pos_5min_profile_190214.m | Operator | CU. |
| Sample Name | NHTs+4-I-NCS | Instrument | micrOTOF-Q |
| Comment | | | |

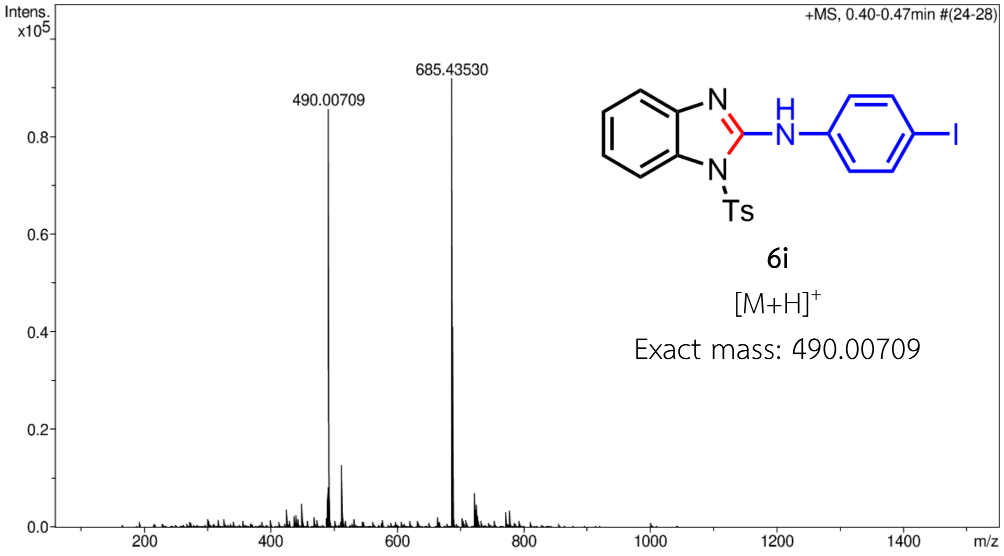


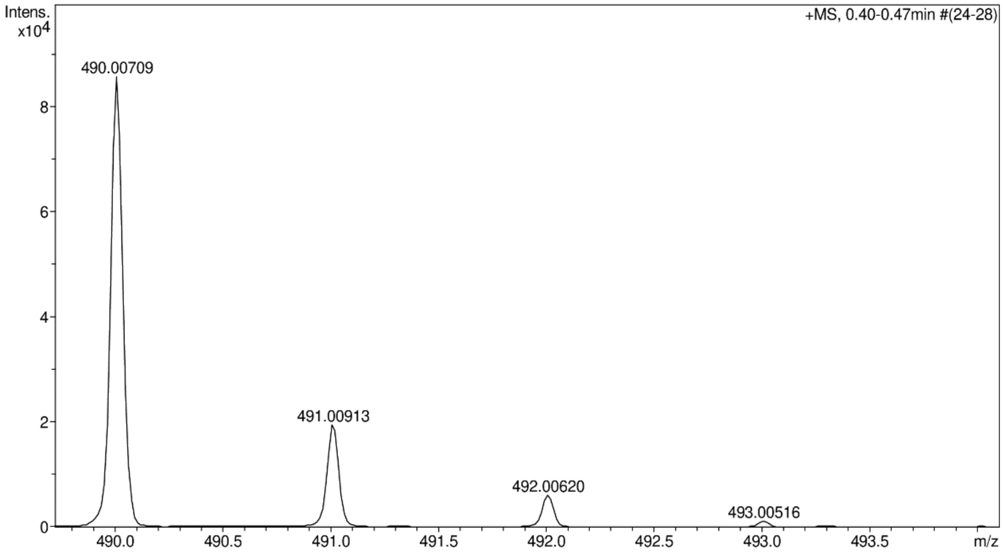


Bruker Compass DataAnalysis 4.0 printed: 12/19/2022 6:18:25 PM Page 1 of 1

**Figure S192** ESI-HRMS spectrum of **6i**

## Generic Display Report

**Analysis Info**

| | | | |
|---|---|---|---|
| Analysis Name | D:\Data\Data Service\221219\NHTs+3-I-NCS_RC1_01_6636.d | Acquisition Date | 12/19/2022 2:04:24 PM |
| Method | nv_pos_5min_profile_190214.m | Operator | CU. |
| Sample Name | NHTs+3-I-NCS | Instrument | micrOTOF-Q |
| Comment | | | |

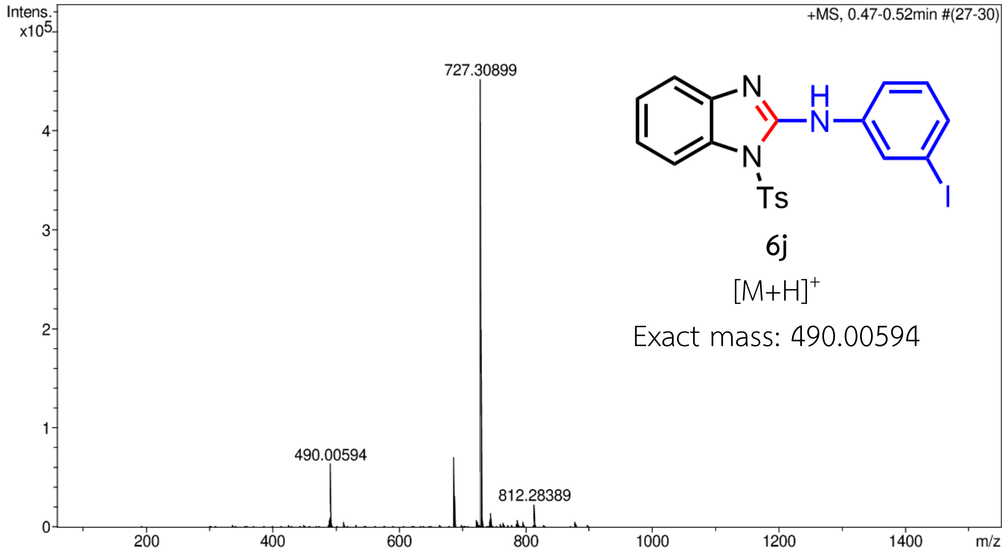


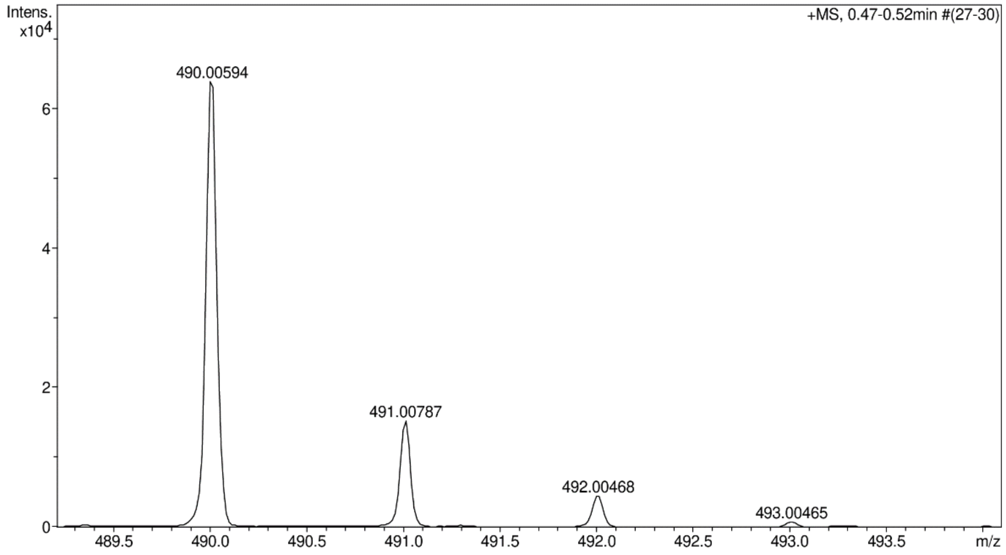


Bruker Compass DataAnalysis 4.0 printed: 12/19/2022 7:03:35 PM Page 1 of 1

**Figure S193** ESI-HRMS spectrum of **6j**

## Generic Display Report

**Analysis Info**

| | | | |
|---|---|---|---|
| Analysis Name | D:\Data\Data Service\221219\NHTs+Ph-NCS_RA2_01_6619.d | Acquisition Date | 12/19/2022 12:14:37 PM |
| Method | nv_pos_5min_profile_190214.m | Operator | CU. |
| Sample Name | NHTs+Ph-NCS | Instrument | micrOTOF-Q |
| Comment | | | |

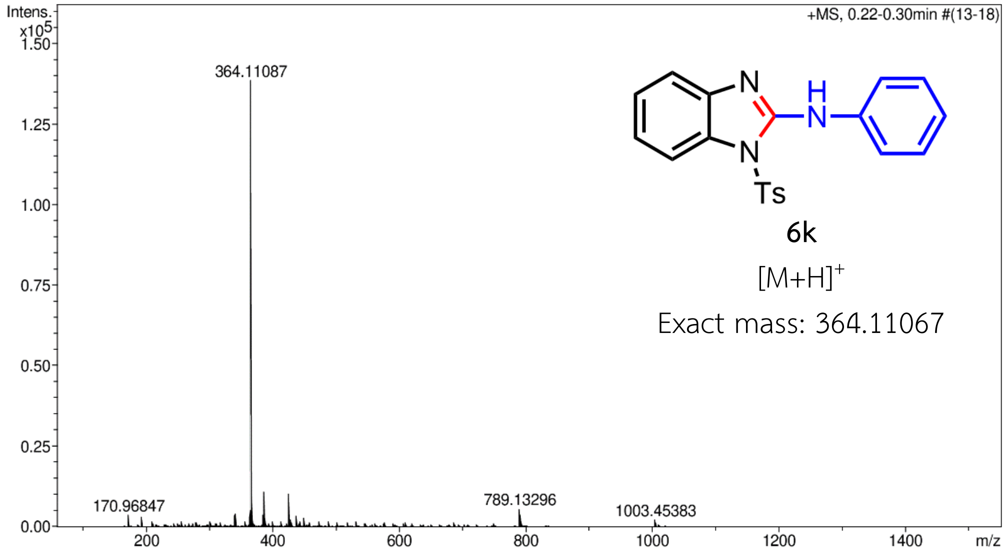


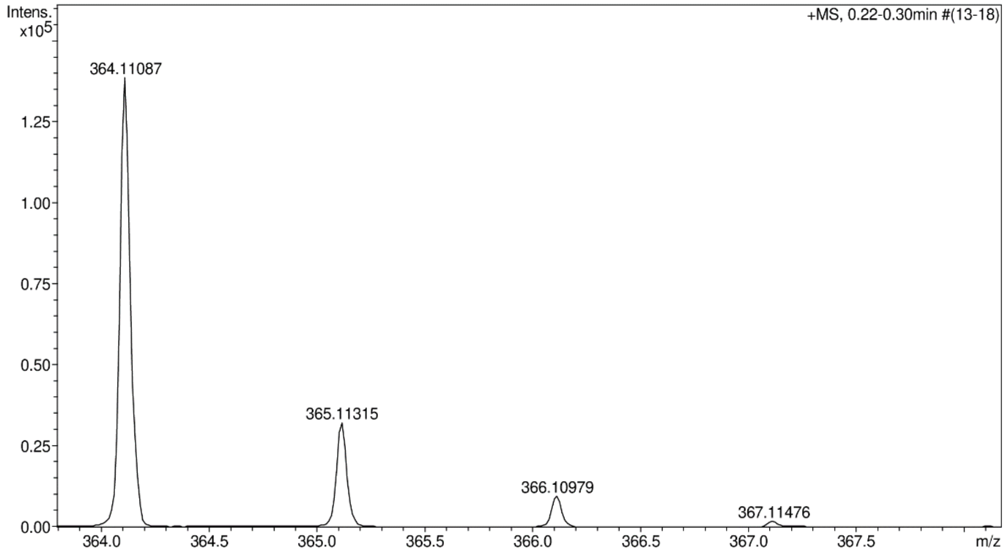




**Figure S194** ESI-HRMS spectrum of **6k**

## Generic Display Report

**Analysis Info**

| | | | |
|---|---|---|---|
| Analysis Name | D:\Data\Data Service\221219\NHTs+4-Me-NCS_RB2_01_6629.d | Acquisition Date | 12/19/2022 1:19:11 PM |
| Method | nv_pos_5min_profile_190214.m | Operator | CU. |
| Sample Name | NHTs+4-Me-NCS | Instrument | micrOTOF-Q |
| Comment | | | |

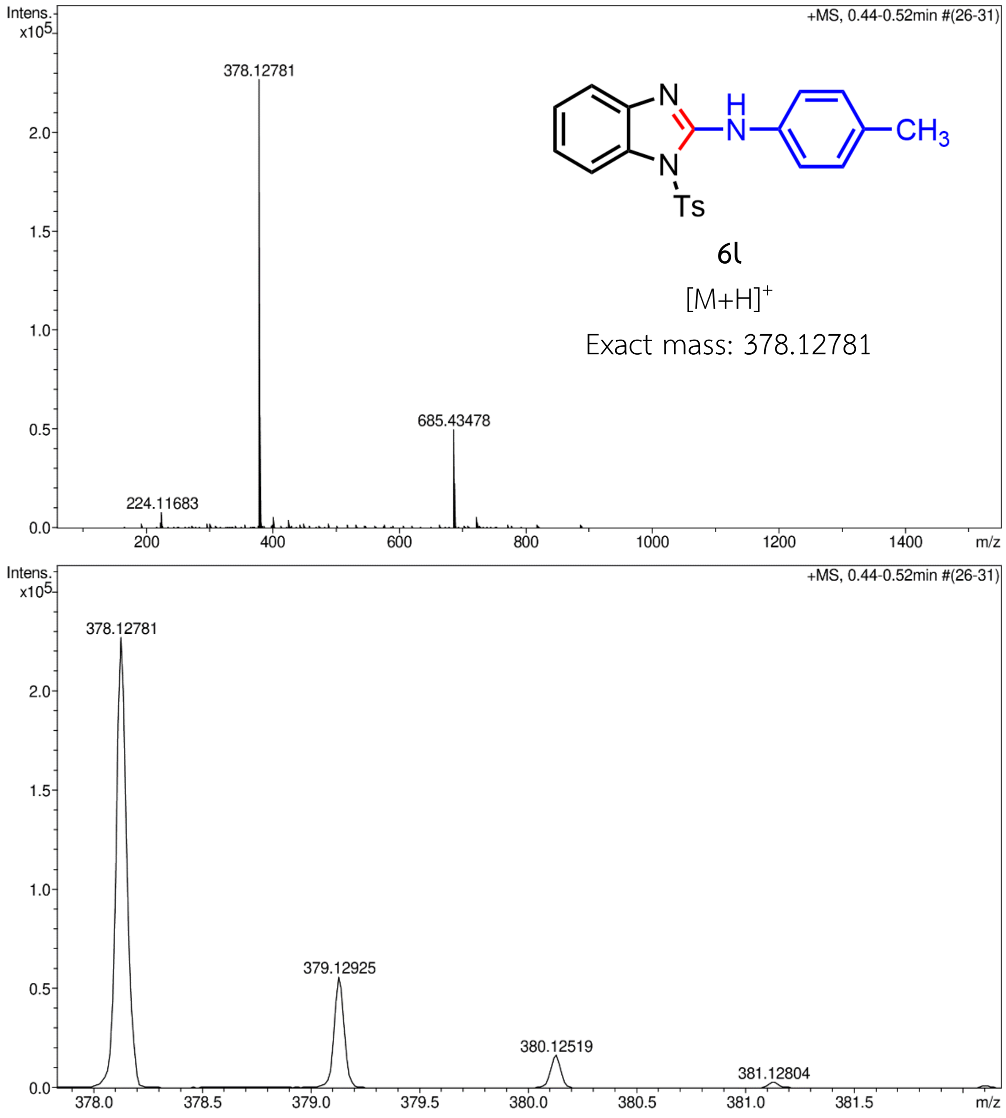


Bruker Compass DataAnalysis 4.0 printed: 12/19/2022 6:34:46 PM Page 1 of 1

**Figure S195** ESI-HRMS spectrum of **6l**

# Generic Display Report

| Analysis Info | | Acquisition Date | 12/19/2022 1:57:56 PM |
|---|---|---|---|
| Analysis Name | D:\Data\Data Service\221219\NHTs+3-Me-NCS_RB8_01_6635.d | | |
| Method | nv_pos_5min_profile_190214.m | Operator | CU. |
| Sample Name | NHTs+3-Me-NCS | Instrument | micrOTOF-Q |
| Comment | | | |

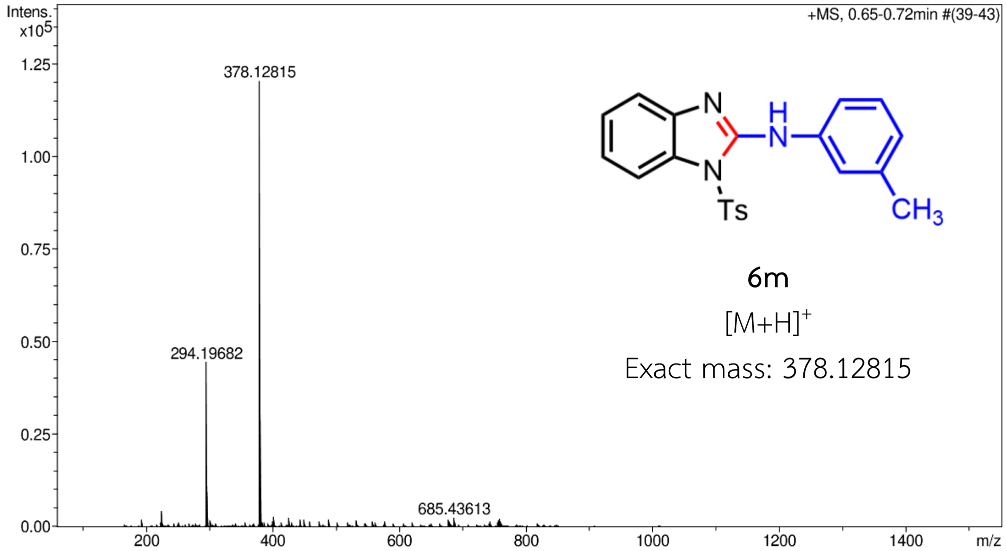


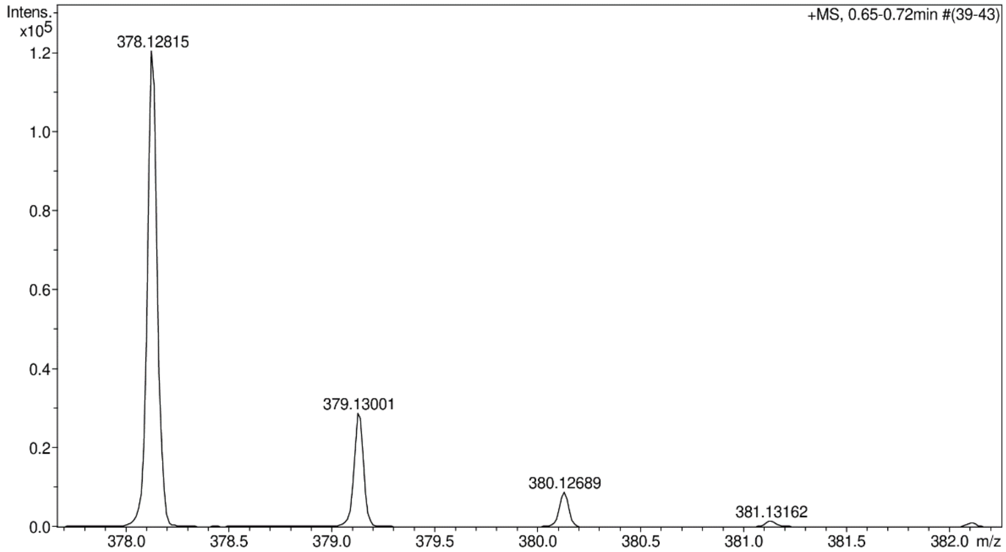




**Figure S196** ESI-HRMS spectrum of **6m**

## Generic Display Report

**Analysis Info**

| | | | |
|---|---|---|---|
| Analysis Name | D:\Data\Data Service\221219\NHTs+2-Me-NCS_RC2_01_6637.d | Acquisition Date | 12/19/2022 2:10:51 PM |
| Method | nv_pos_5min_profile_190214.m | Operator | CU. |
| Sample Name | NHTs+2-Me-NCS | Instrument | micrOTOF-Q |
| Comment | | | |

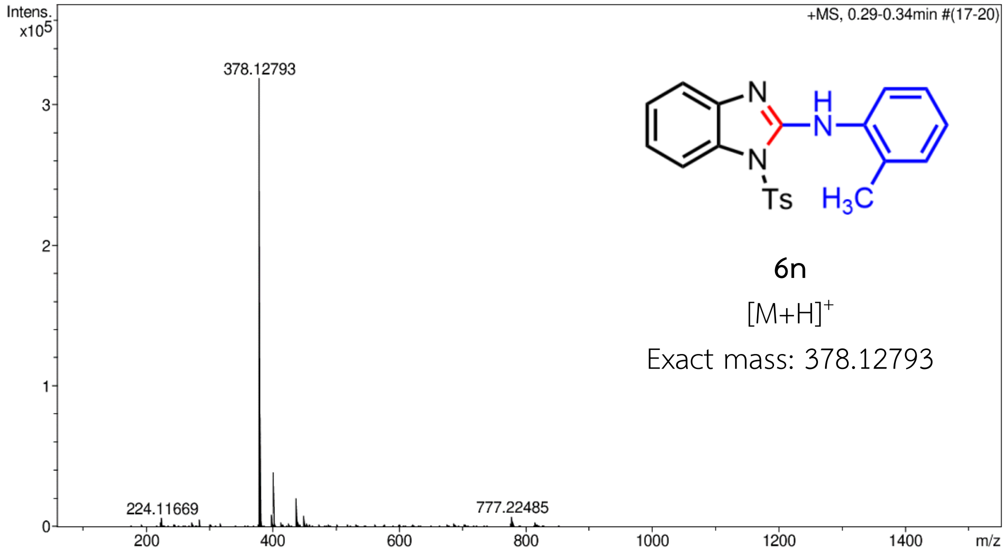


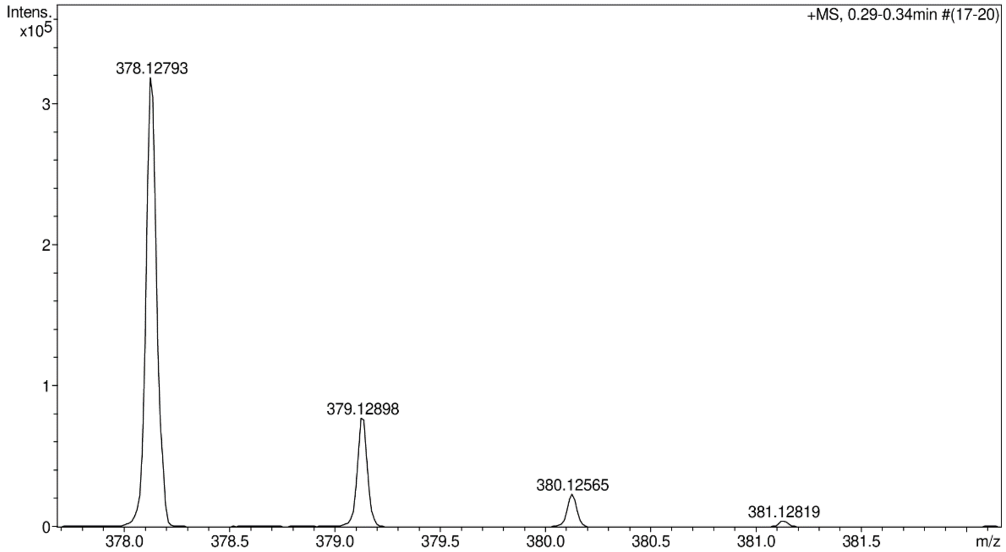


Bruker Compass DataAnalysis 4.0 printed: 12/19/2022 7:07:31 PM Page 1 of 1

**Figure S197** ESI-HRMS spectrum of **6n**

## Generic Display Report

**Analysis Info**

Analysis Name D:\Data\Data Service\221219\NHTs+3,5-diMe-NCS_RB5_01_6632.d
Method nv_pos_5min_profile_190214.m
Sample Name NHTs+3,5-diMe-NCS
Comment

Acquisition Date 12/19/2022 1:38:34 PM
Operator CU.
Instrument micrOTOF-Q

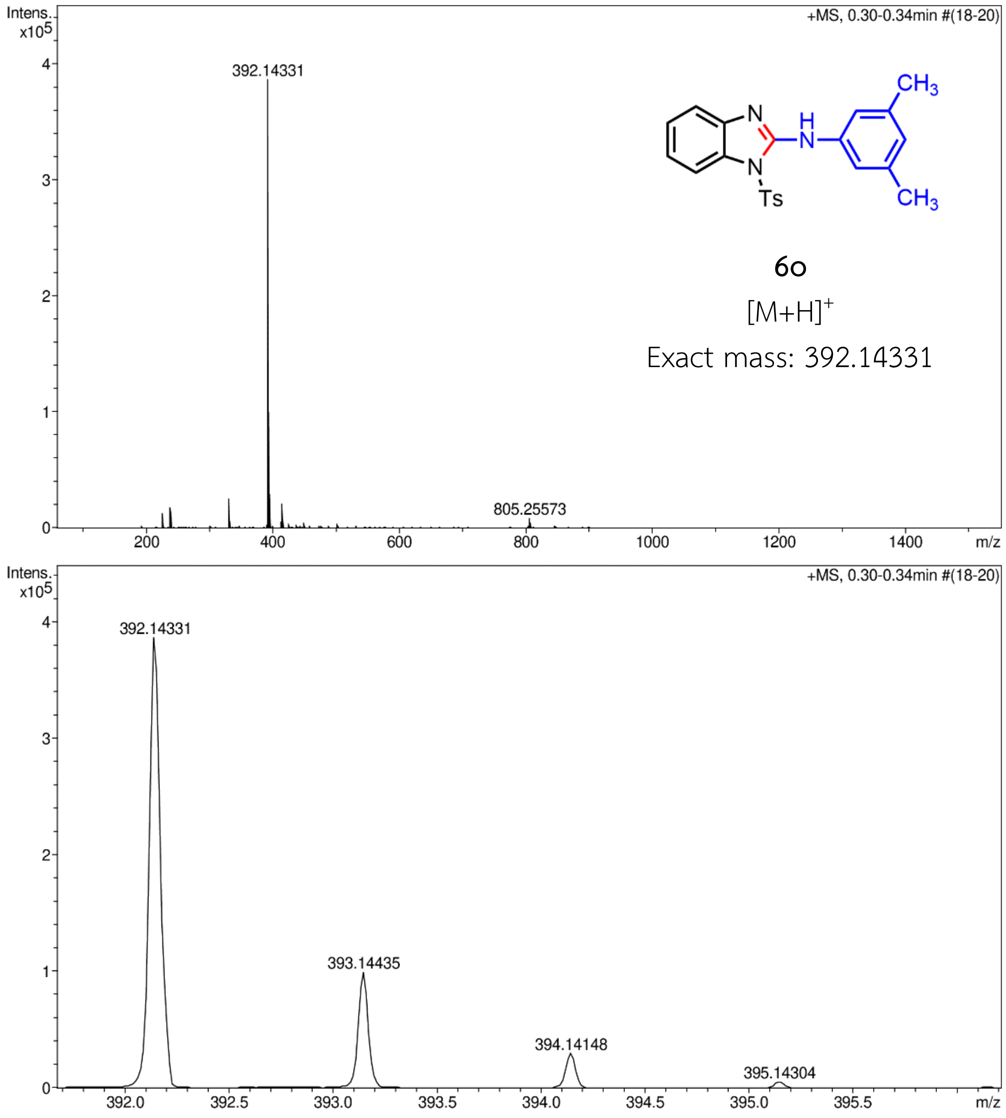


Bruker Compass DataAnalysis 4.0 printed: 12/19/2022 6:46:30 PM Page 1 of 1

**Figure S198** ESI-HRMS spectrum of **6o**

## Generic Display Report

**Analysis Info**

| | | | |
|---|---|---|---|
| Analysis Name | D:\Data\Data Service\221219\NHTs+4-Et-NCS_RB3_01_6630.d | Acquisition Date | 12/19/2022 1:25:39 PM |
| Method | nv_pos_5min_profile_190214.m | Operator | CU. |
| Sample Name | NHTs+4-Et-NCS | Instrument | micrOTOF-Q |
| Comment | | | |

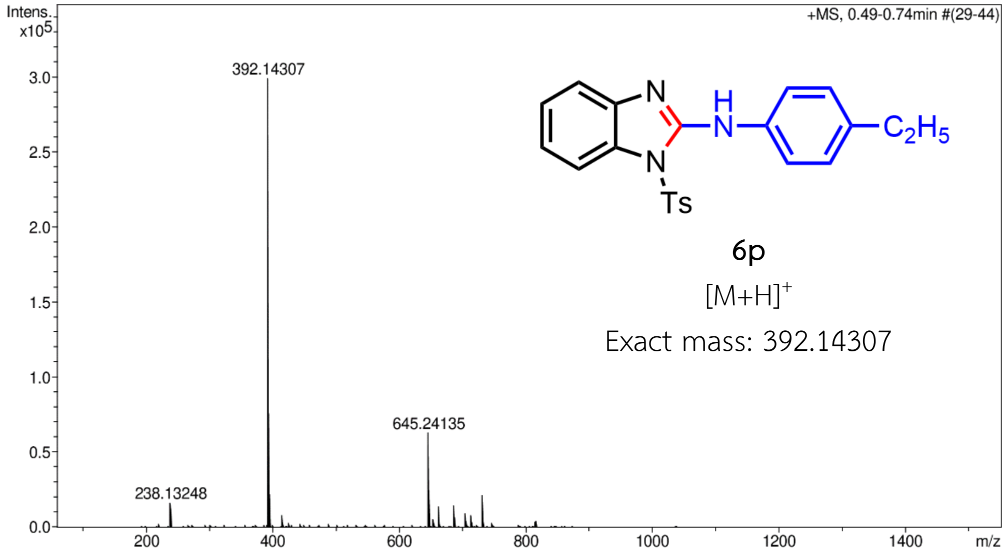


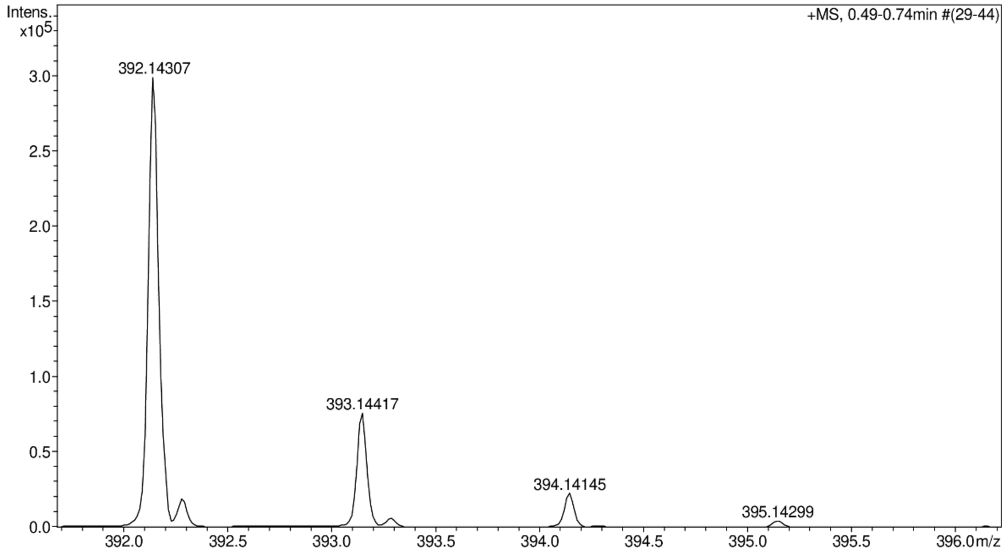


Bruker Compass DataAnalysis 4.0 printed: 12/19/2022 6:38:58 PM Page 1 of 1

**Figure S199** ESI-HRMS spectrum of **6p**

# Generic Display Report

| Analysis Info | | Acquisition Date | 12/19/2022 12:46:54 PM |
|---|---|---|---|
| Analysis Name | D:\Data\Data Service\221219\NHTs+4-OMe-NCS_RA5_01_6624.d | | |
| Method | nv_pos_5min_profile_190214.m | Operator | CU. |
| Sample Name | NHTs+4-OMe-NCS | Instrument | micrOTOF-Q |
| Comment | | | |

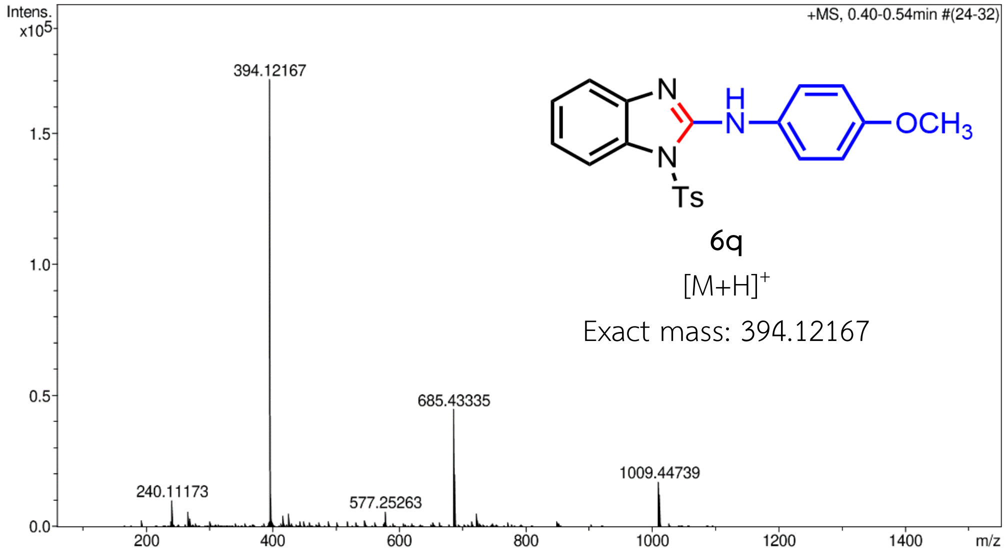


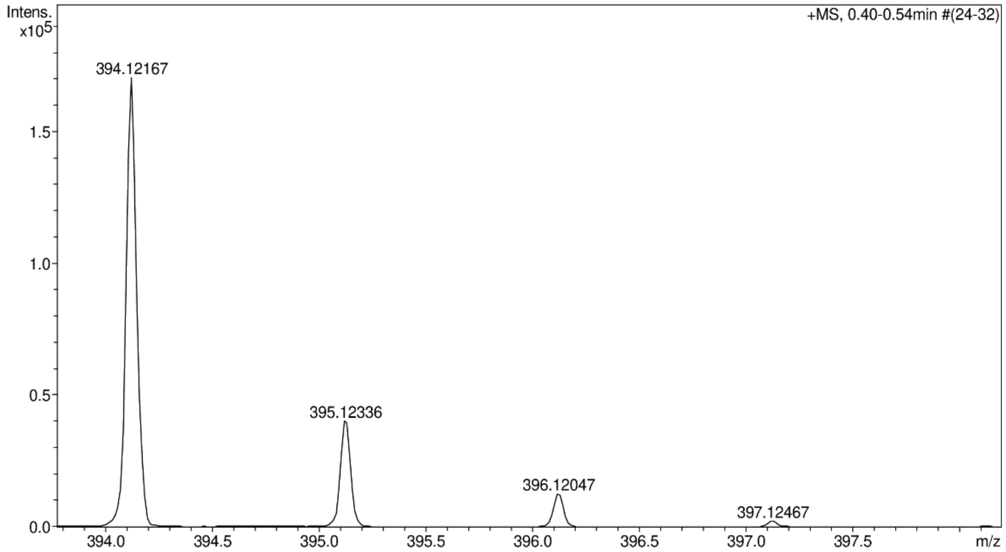


Bruker Compass DataAnalysis 4.0 printed: 12/19/2022 6:09:42 PM Page 1 of 1

**Figure S200** ESI-HRMS spectrum of **6q**

# Generic Display Report

**Analysis Info**

| | | | |
|---|---|---|---|
| Analysis Name | D:\Data\Data Service\221219\NHTs+3-OMe-NCS_RB7_01_6634.d | Acquisition Date | 12/19/2022 1:51:29 PM |
| Method | nv_pos_5min_profile_190214.m | Operator | CU. |
| Sample Name | NHTs+3-OMe-NCS | Instrument | micrOTOF-Q |
| Comment | | | |

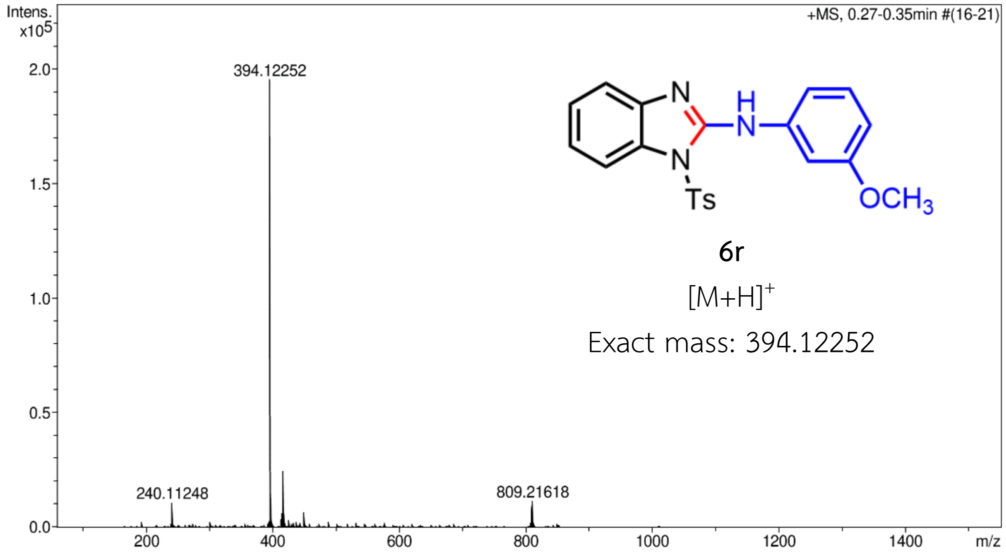


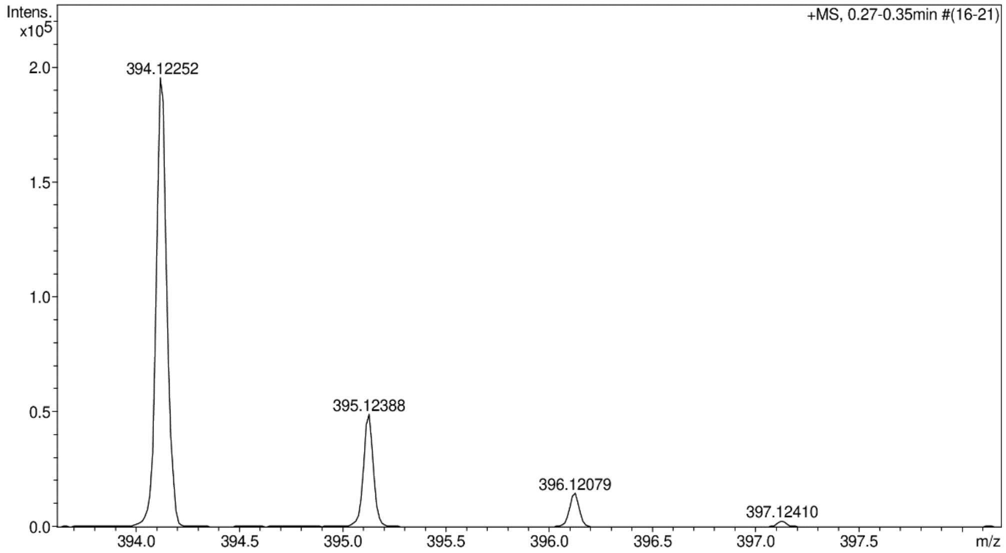


Bruker Compass DataAnalysis 4.0 printed: 12/19/2022 6:54:26 PM Page 1 of 1

**Figure S201** ESI-HRMS spectrum of **6r**

# Generic Display Report

| | | | |
|---|---|---|---|
| **Analysis Info** | | Acquisition Date | 12/19/2022 2:23:46 PM |
| Analysis Name | D:\Data\Data Service\221219\NHTs+2-OMe-NCS_RC4_01_6639.d | | |
| Method | nv_pos_5min_profile_190214.m | Operator | CU. |
| Sample Name | NHTs+2-OMe-NCS | Instrument | micrOTOF-Q |
| Comment | | | |

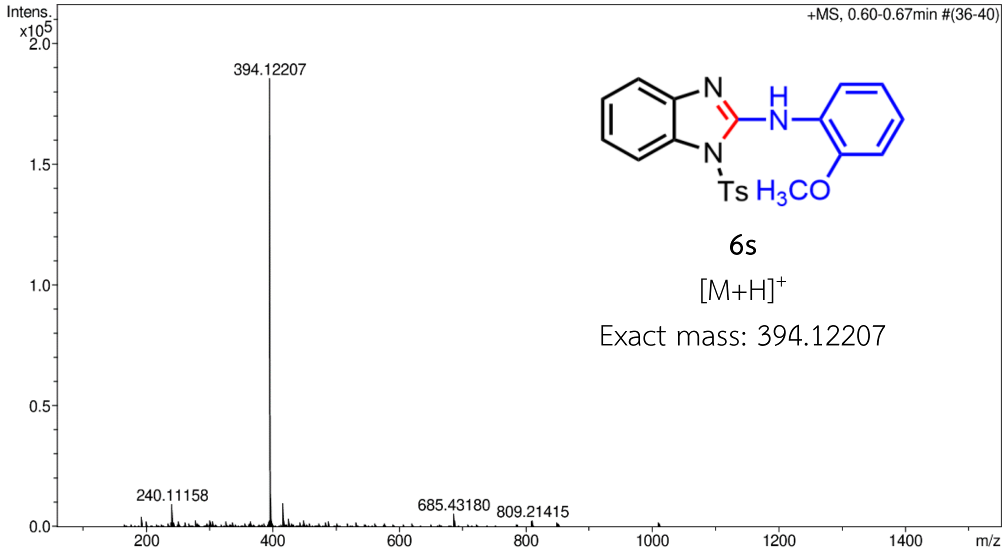


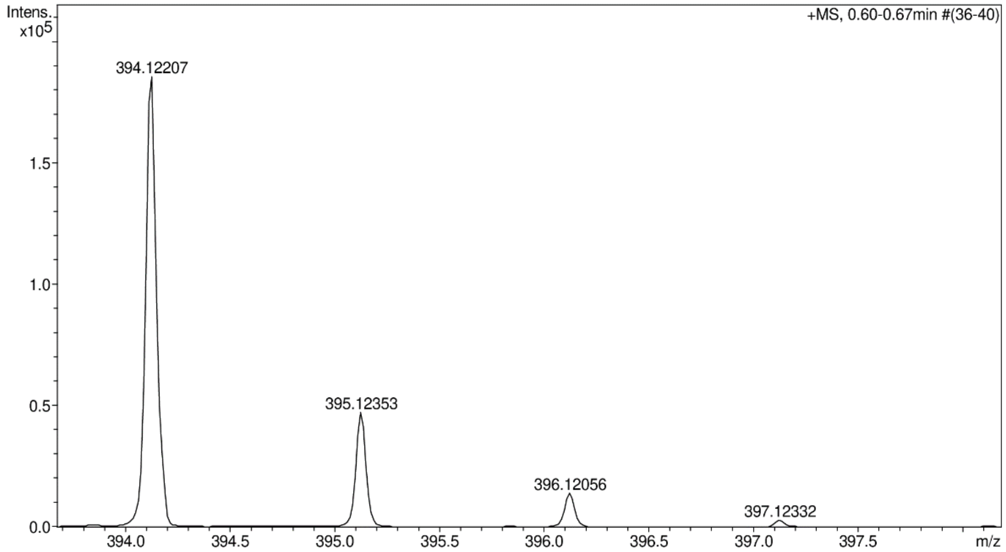


Bruker Compass DataAnalysis 4.0 printed: 12/19/2022 7:14:48 PM Page 1 of 1

**Figure S202** ESI-HRMS spectrum of **6s**

## Generic Display Report

| Analysis Info | | Acquisition Date | 12/19/2022 1:32:07 PM |
|---|---|---|---|
| Analysis Name | D:\Data\Data Service\221219\NHTs+4-OCF3-NCS_RB4_01_6631.d | | |
| Method | nv_pos_5min_profile_190214.m | Operator | CU. |
| Sample Name | NHTs+4-OCF3-NCS | Instrument | micrOTOF-Q |
| Comment | | | |

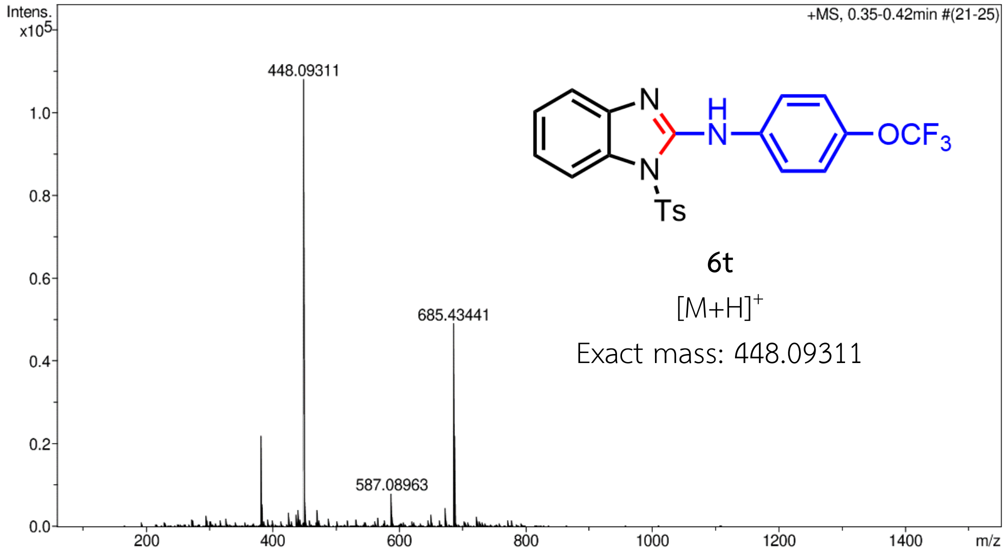


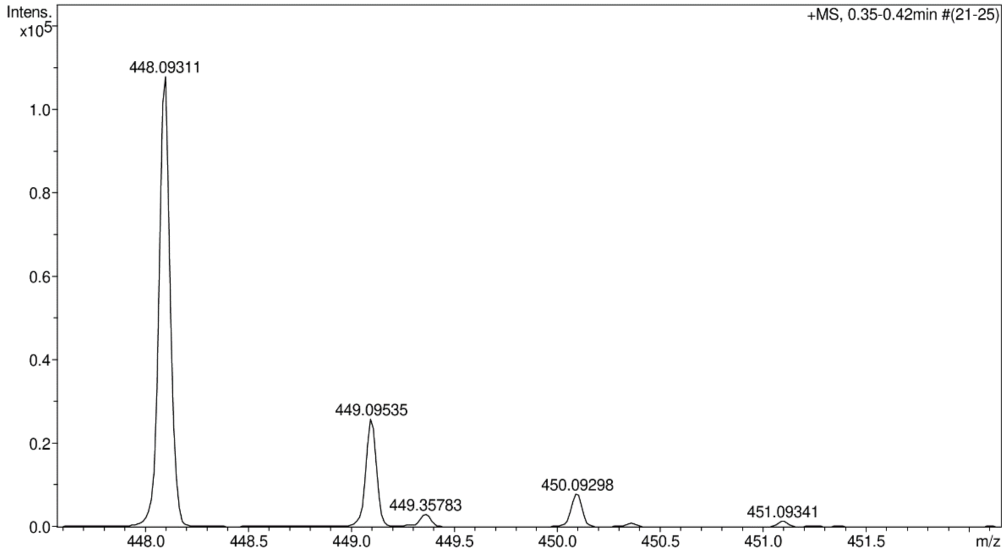


Bruker Compass DataAnalysis 4.0 printed: 12/19/2022 6:42:45 PM Page 1 of 1

**Figure S203** ESI-HRMS spectrum of **6t**

# Generic Display Report

**Analysis Info**

| | | | |
|---|---|---|---|
| Analysis Name | D:\Data\Data Service\230918\NHTs+4-diCH3-amino_RA3_01_7717.d | Acquisition Date | 9/18/2023 2:46:06 PM |
| Method | nv_pos_5min_profile_190214.m | Operator | CU. |
| Sample Name | NHTs+4-diCH3-amino | Instrument | micrOTOF-Q |
| Comment | | | |

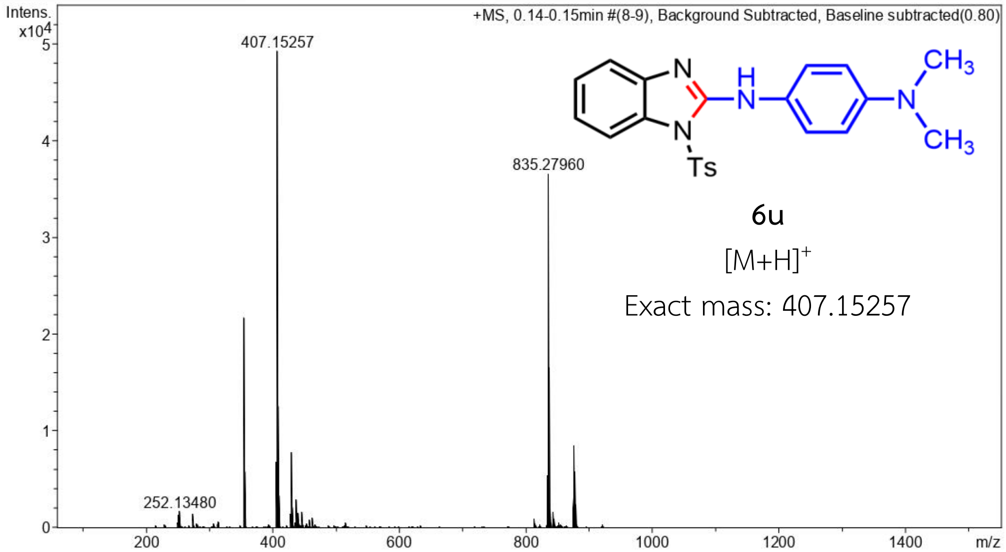


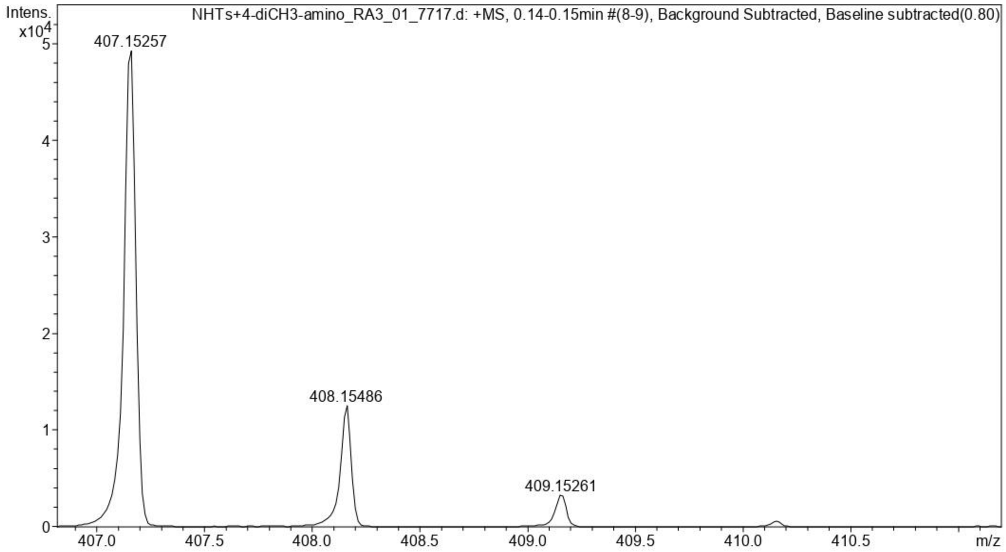


Bruker Compass DataAnalysis 4.0 printed: 9/18/2023 4:20:20 PM Page 1 of 1

**Figure S204** ESI-HRMS spectrum of **6u**

## Generic Display Report

**Analysis Info** Acquisition Date 2/13/2023 12:17:11 PM

Analysis Name D:\Data\Data Service\230213\NHTs+2,6-diMe-NCS_RA4_01_6750.d

Method nv_pos_5min_profile_190214.m Operator CU.

Sample Name NHTs+2,6-diMe-NCS Instrument micrOTOF-Q

Comment

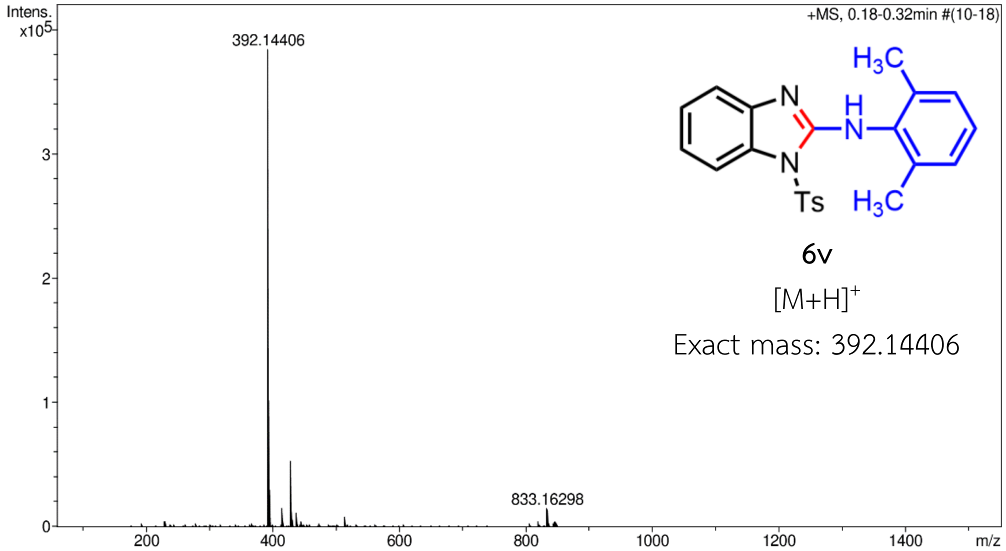


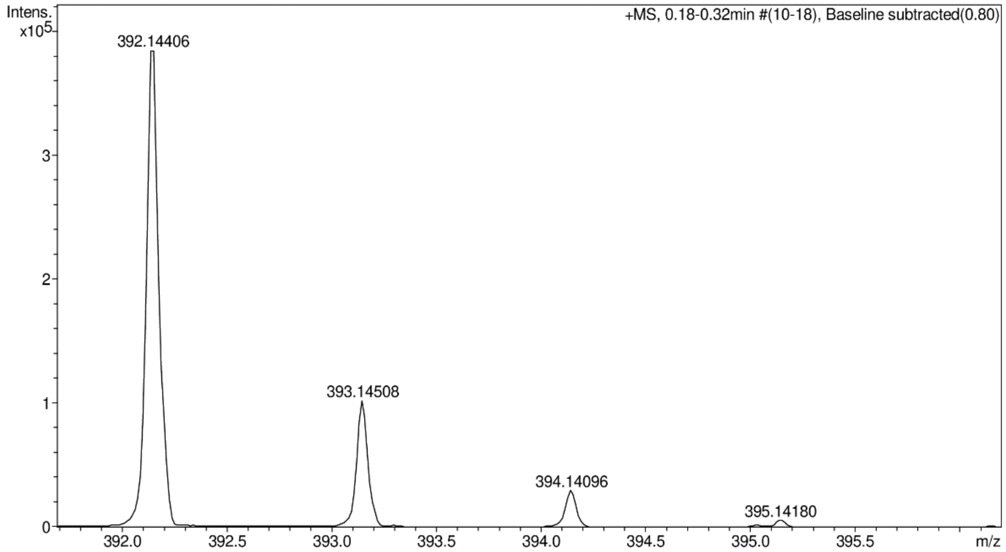


Bruker Compass DataAnalysis 4.0 printed: 2/13/2023 4:13:28 PM Page 1 of 1

**Figure S205** ESI-HRMS spectrum of **6v**

## Generic Display Report

**Analysis Info**

| | | | |
|---|---|---|---|
| Analysis Name | D:\Data\Data Service\221219\NHTs+1,3diiPr-NCS_RD4_01_6648.d | Acquisition Date | 12/19/2022 3:21:43 PM |
| Method | nv_pos_5min_profile_190214.m | Operator | CU. |
| Sample Name | NHTs+1,3diiPr-NCS | Instrument | micrOTOF-Q |
| Comment | | | |

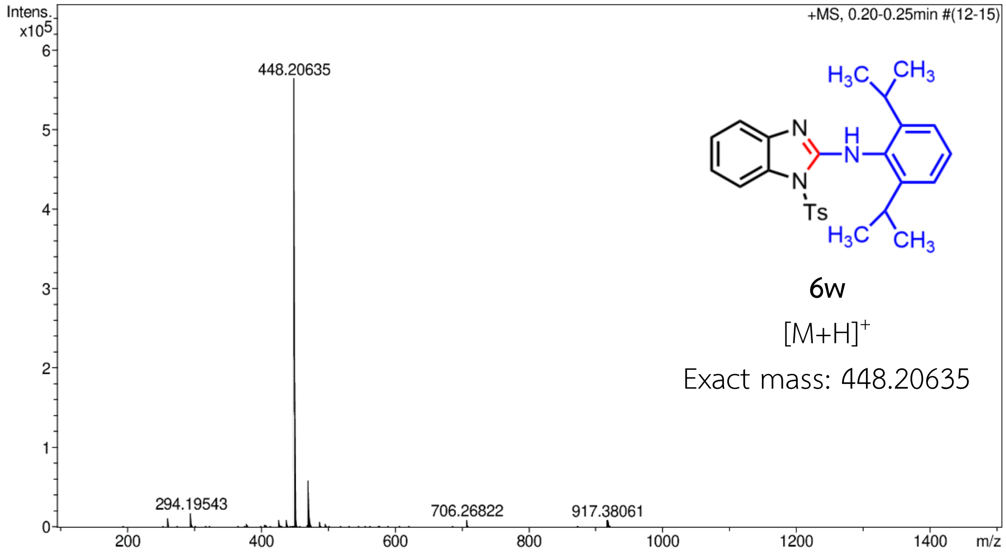


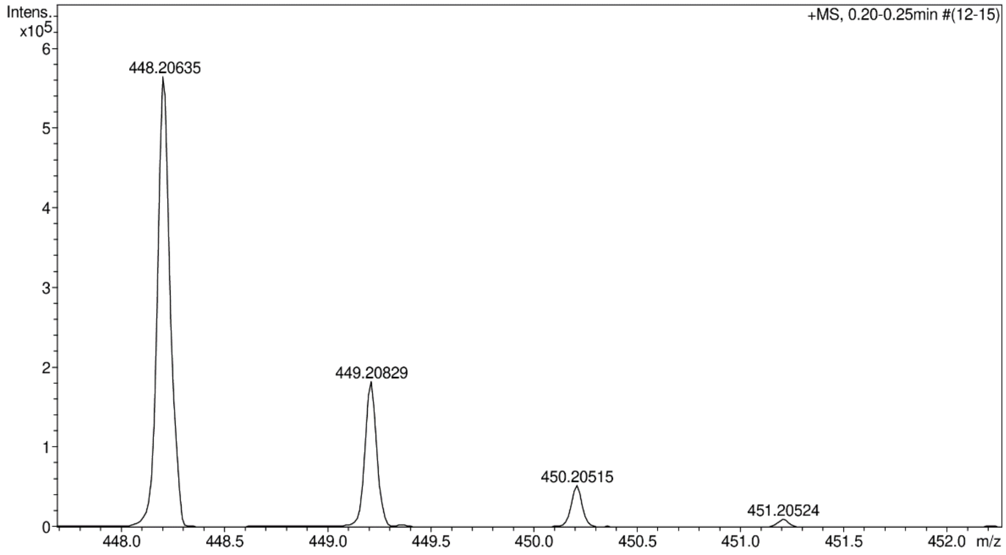


Bruker Compass DataAnalysis 4.0 printed: 12/19/2022 7:50:52 PM Page 1 of 1

**Figure S206** ESI-HRMS spectrum of **6w**

## Generic Display Report

**Analysis Info**

| | | | |
|---|---|---|---|
| Analysis Name | D:\Data\Data Service\230925\NHTs+3-OH-NCS_RA6_01_7733.d | Acquisition Date | 9/25/2023 10:57:50 AM |
| Method | nv_pos_5min_profile_190214.m | Operator | CU. |
| Sample Name | NHTs+3-OH-NCS | Instrument | micrOTOF-Q |
| Comment | | | |

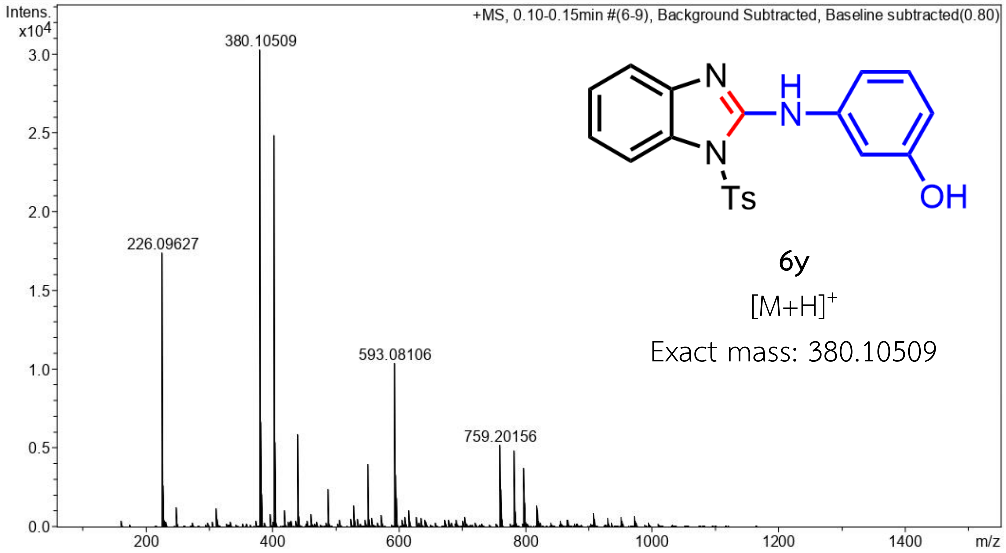


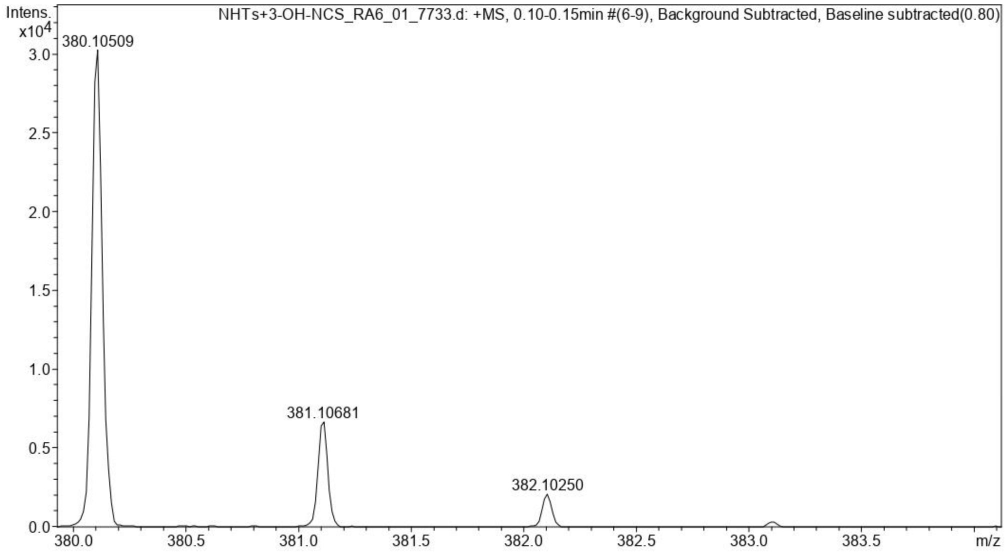


Bruker Compass DataAnalysis 4.0 printed: 9/25/2023 4:45:31 PM Page 1 of 1

**Figure S207** ESI-HRMS spectrum of **6y**

## Generic Display Report

**Analysis Info**

| | | | |
|---|---|---|---|
| Analysis Name | D:\Data\Data Service\230124\NHTs+4-OTBDMS-NCS_RA8_01_6709.d | Acquisition Date | 1/24/2023 4:39:58 PM |
| Method | nv_pos_5min_profile_190214.m | Operator | CU. |
| Sample Name | NHTs+4-OTBDMS-NCS | Instrument | micrOTOF-Q |
| Comment | | | |

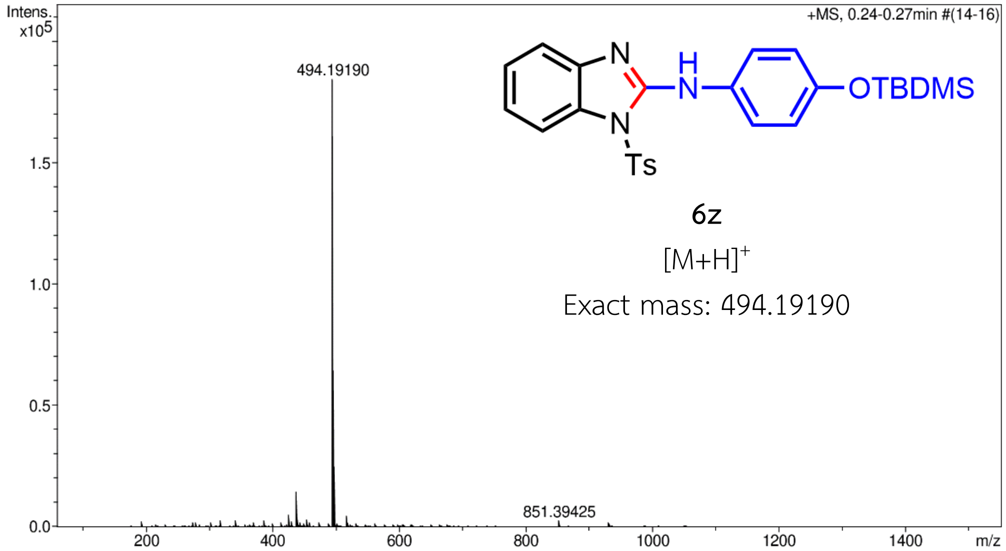


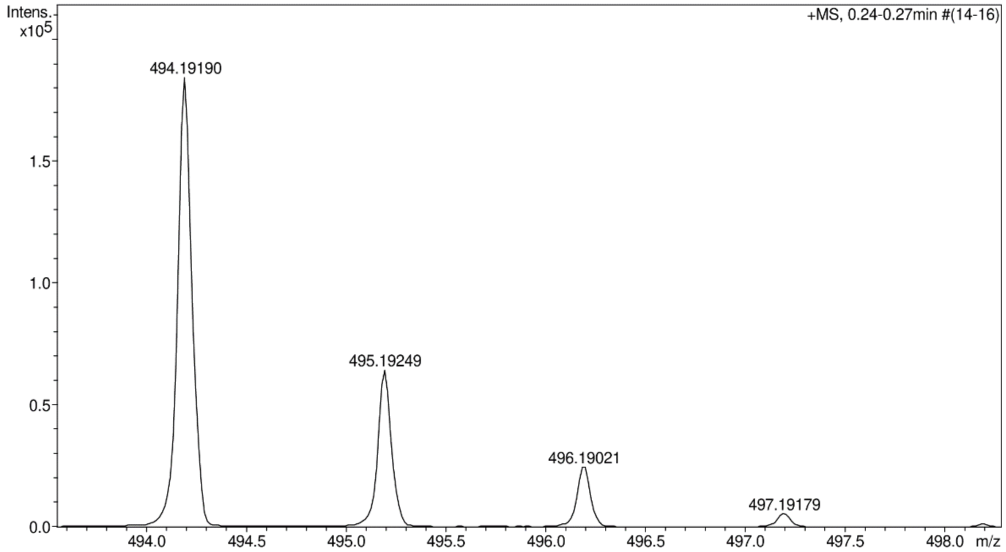


Bruker Compass DataAnalysis 4.0 printed: 1/24/2023 5:37:18 PM Page 1 of 1

**Figure S208** ESI-HRMS spectrum of **6z**

# Generic Display Report

| | | | |
|---|---|---|---|
| **Analysis Info** | | Acquisition Date | 1/24/2023 4:48:51 PM |
| Analysis Name | D:\Data\Data Service\230124\NHTs+3-OTBDMS-NCS_RB1_01_6710.d | | |
| Method | nv_pos_5min_profile_190214.m | Operator | CU. |
| Sample Name | NHTs+3-OTBDMS-NCS | Instrument | micrOTOF-Q |
| Comment | | | |

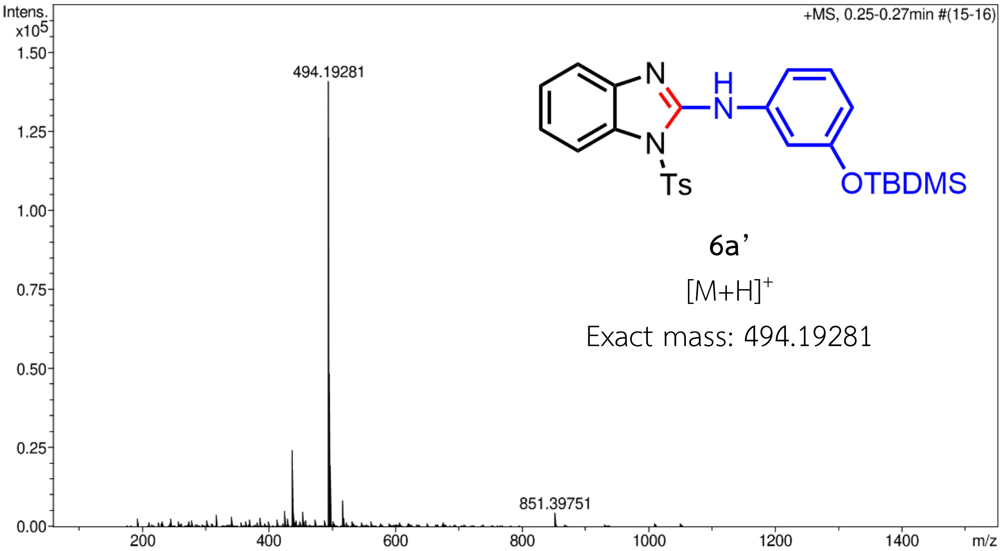


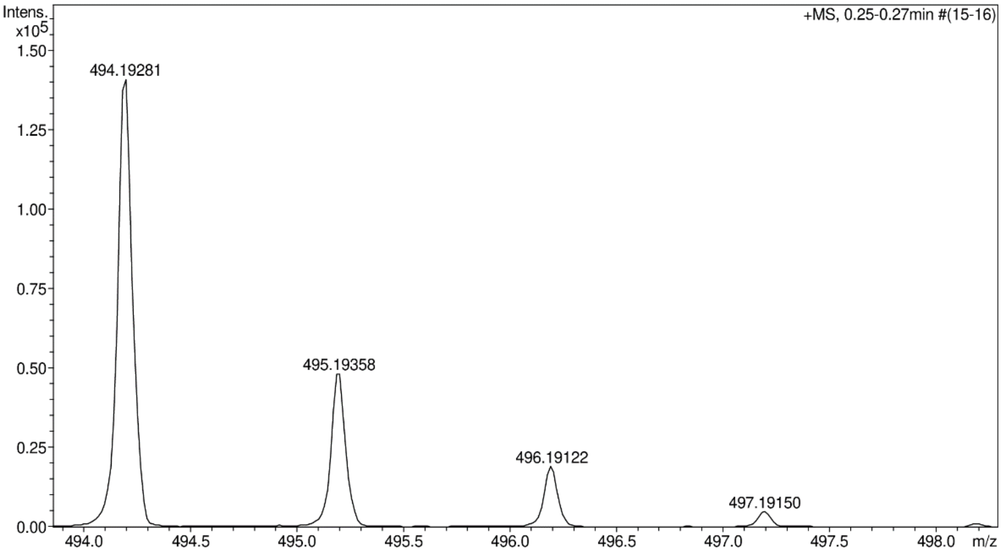




**Figure S209** ESI-HRMS spectrum of **6a'**

# Generic Display Report

**Analysis Info**

| | | | |
|---|---|---|---|
| Analysis Name | D:\Data\Data Service\230925\NHTs+4-OBn-NCS_RA4_01_7731.d | Acquisition Date | 9/25/2023 10:44:55 AM |
| Method | nv_pos_5min_profile_190214.m | Operator | CU. |
| Sample Name | NHTs+4-OBn-NCS | Instrument | micrOTOF-Q |
| Comment | | | |

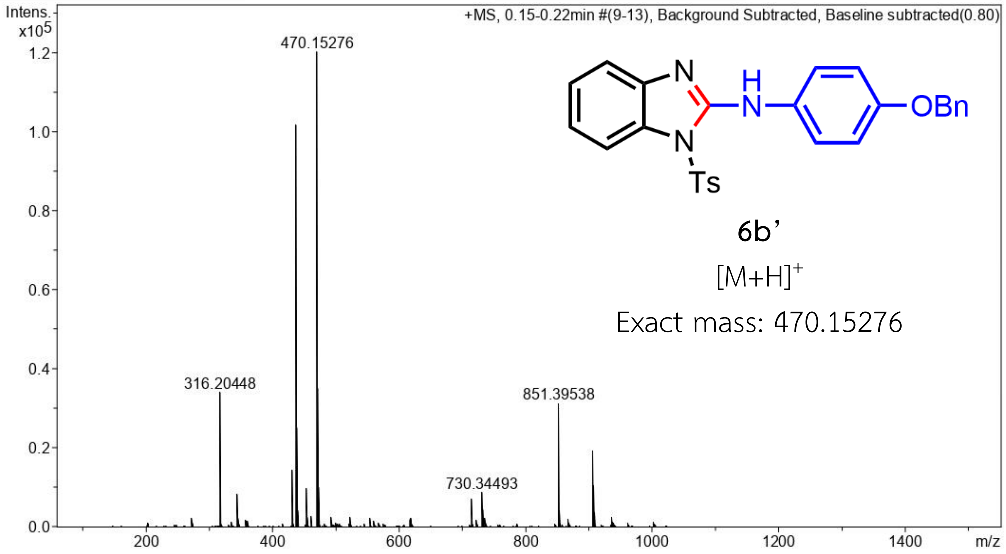


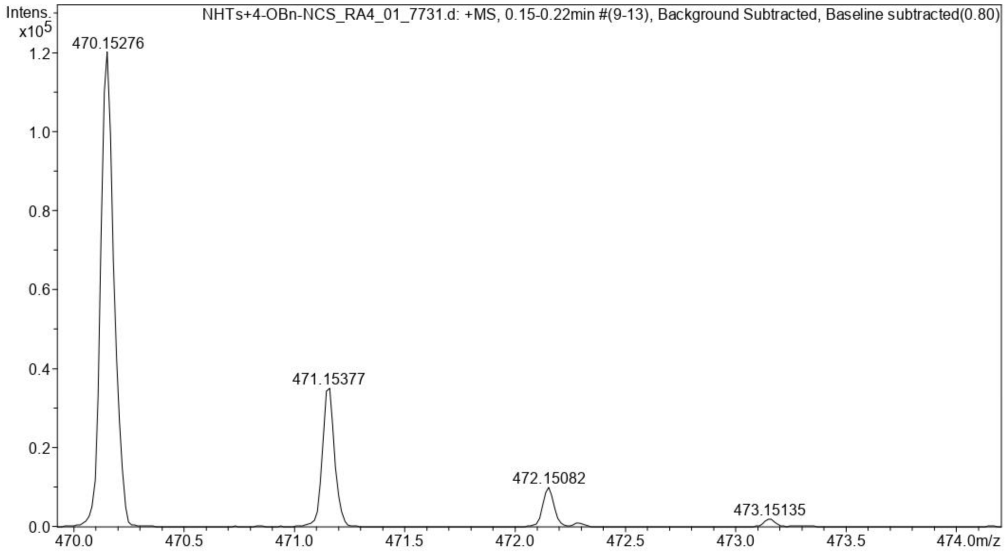


Bruker Compass DataAnalysis 4.0 printed: 9/25/2023 4:23:35 PM Page 1 of 1

**Figure S210** ESI-HRMS spectrum of **6b'**

## Generic Display Report

| | | | |
|---|---|---|---|
| **Analysis Info** | | Acquisition Date | 9/25/2023 4:44:16 PM |
| Analysis Name | D:\Data\Data Service\230925\NHTs+4-OTs-NCS_RB2_01_7739.d | | |
| Method | nv_pos_5min_profile_190214.m | Operator | CU. |
| Sample Name | NHTs+4-OTs-NCS | Instrument | micrOTOF-Q |
| Comment | | | |

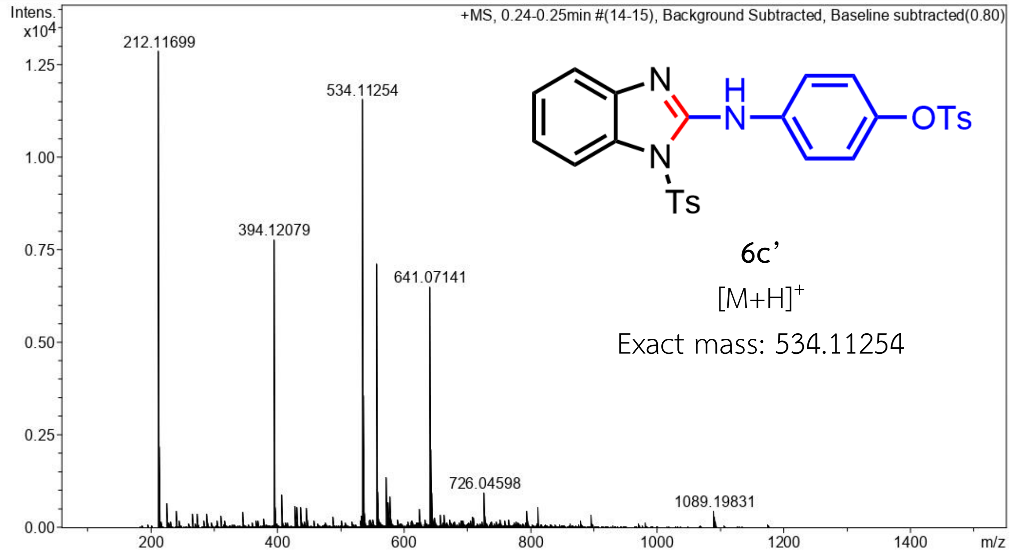


**6c'**

$[M+H]^+$

Exact mass: 534.11254

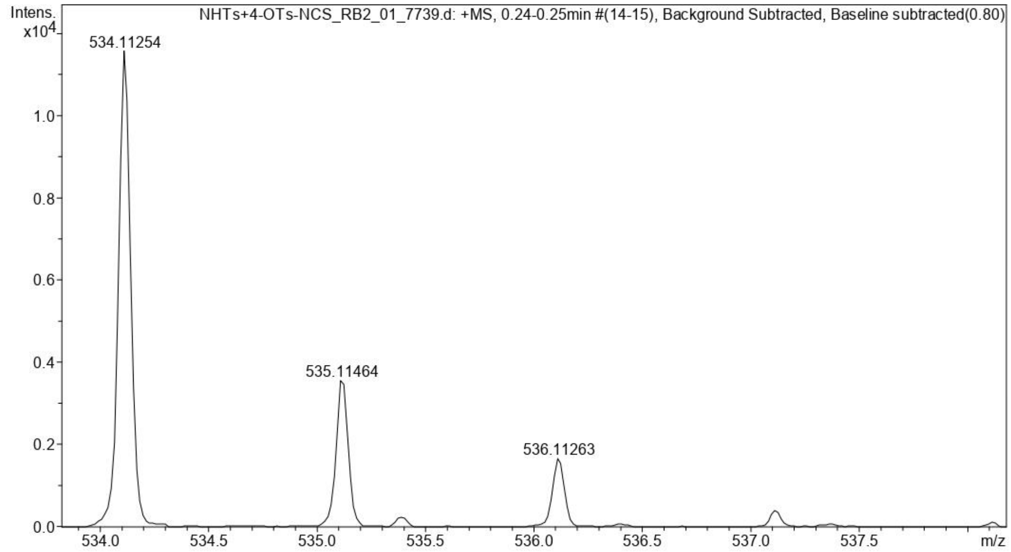


Bruker Compass DataAnalysis 4.0 printed: 9/25/2023 6:19:56 PM Page 1 of 1

**Figure S211** ESI-HRMS spectrum of **6c'**

## Generic Display Report

**Analysis Info**

| | | | |
|---|---|---|---|
| Analysis Name | D:\Data\Data Service\221219\NHTs+4-CF3-NCS_RA6_01_6625.d | Acquisition Date | 12/19/2022 12:53:21 PM |
| Method | nv_pos_5min_profile_190214.m | Operator | CU. |
| Sample Name | NHTs+4-CF3-NCS | Instrument | micrOTOF-Q |
| Comment | | | |

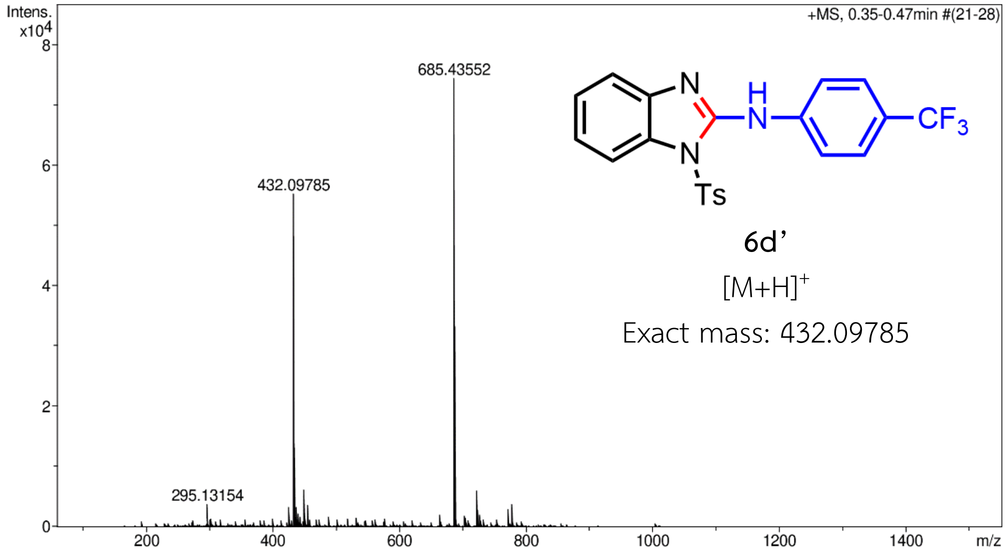


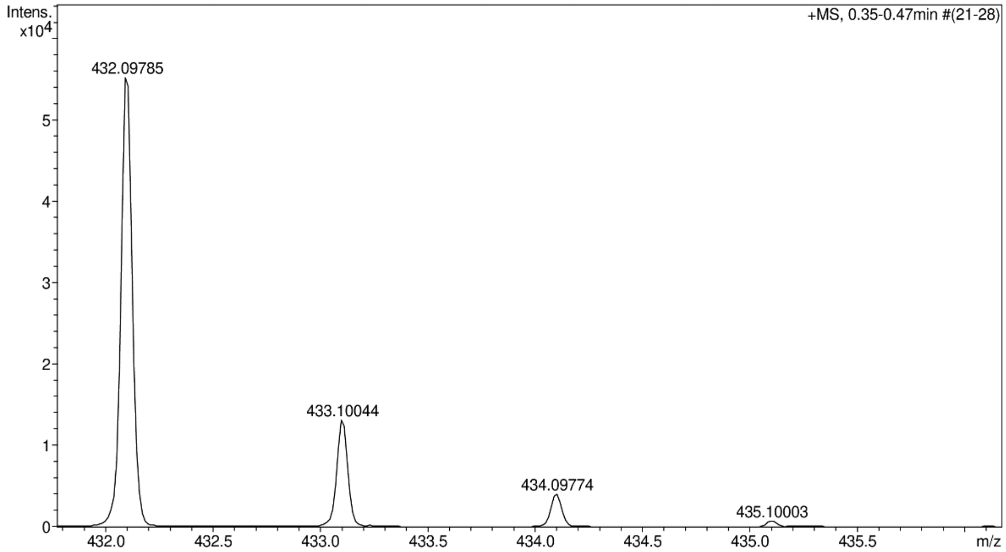


Bruker Compass DataAnalysis 4.0 printed: 12/19/2022 6:13:58 PM Page 1 of 1

**Figure S212** ESI-HRMS spectrum of **6d'**

# Generic Display Report

**Analysis Info**

| | | | |
|---|---|---|---|
| Analysis Name | D:\Data\Data Service\221219\NHTs+3-CF3-NCS_RB6_01_6633.d | Acquisition Date | 12/19/2022 1:45:01 PM |
| Method | nv_pos_5min_profile_190214.m | Operator | CU. |
| Sample Name | NHTs+3-CF3-NCS | Instrument | micrOTOF-Q |
| Comment | | | |

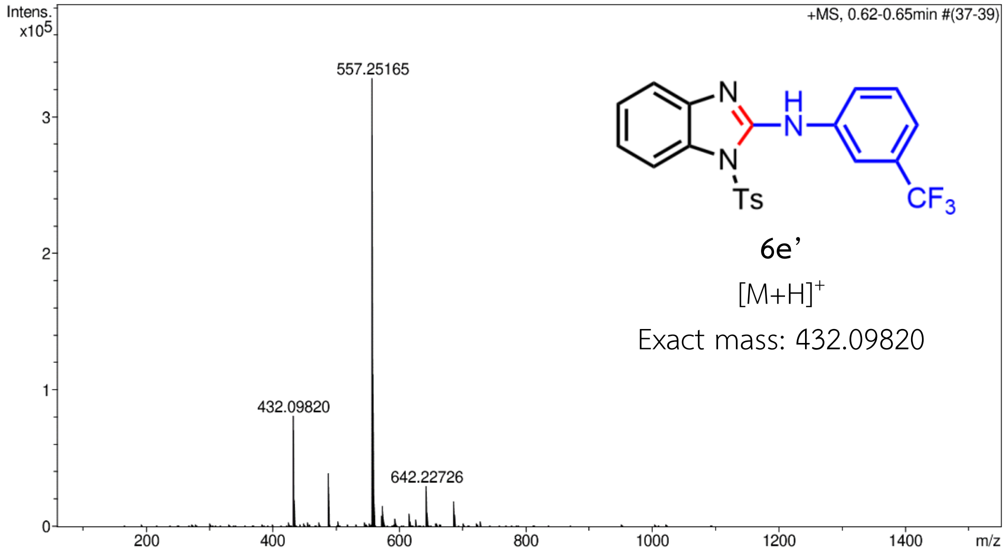


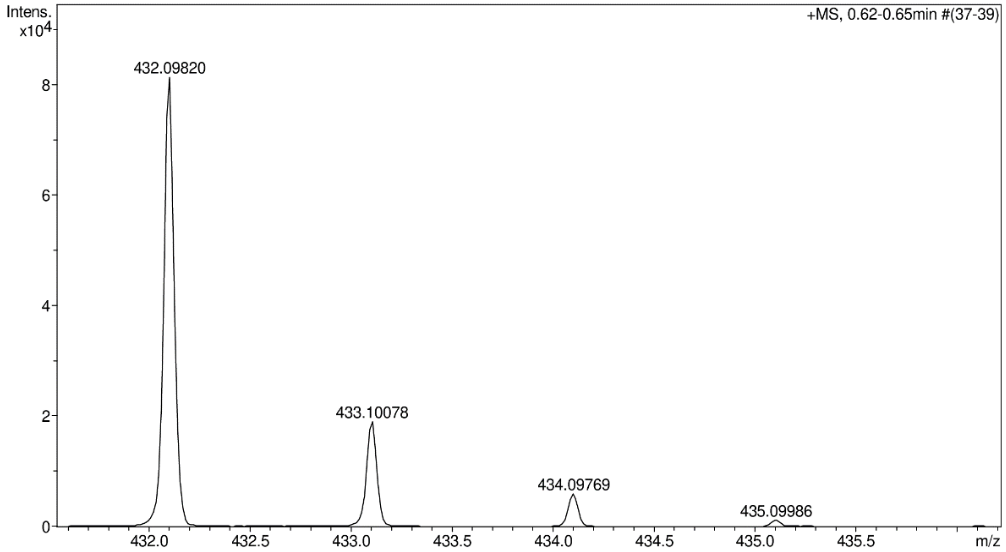


Bruker Compass DataAnalysis 4.0 printed: 12/19/2022 6:51:03 PM Page 1 of 1

**Figure S213** ESI-HRMS spectrum of **6e'**

## Generic Display Report

**Analysis Info**

| | | | |
|---|---|---|---|
| Analysis Name | D:\Data\Data Service\221219\NHTs+2-CF3-NCS_RD1_01_6645.d | Acquisition Date | 12/19/2022 3:02:31 PM |
| Method | nv_pos_5min_profile_190214.m | Operator | CU. |
| Sample Name | NHTs+2-CF3-NCS | Instrument | micrOTOF-Q |
| Comment | | | |

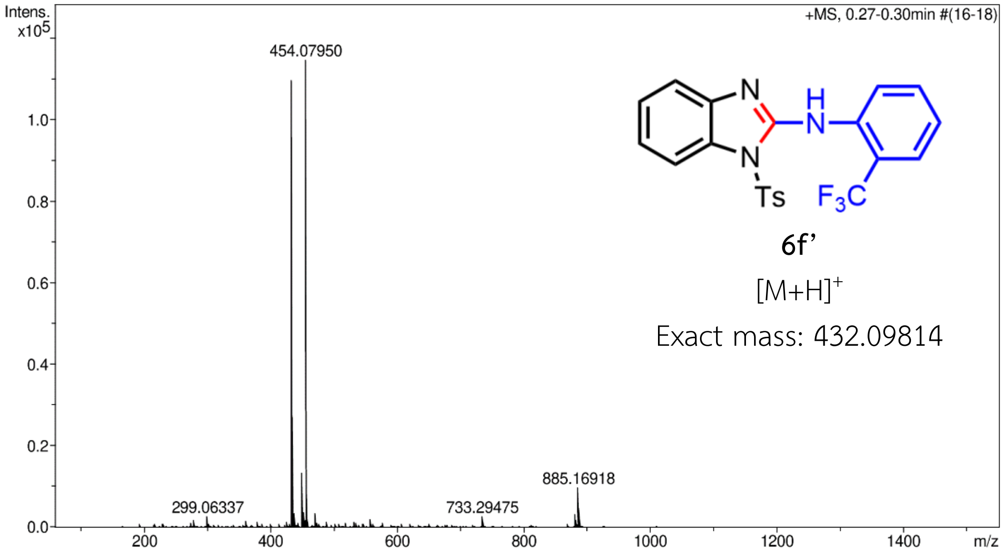


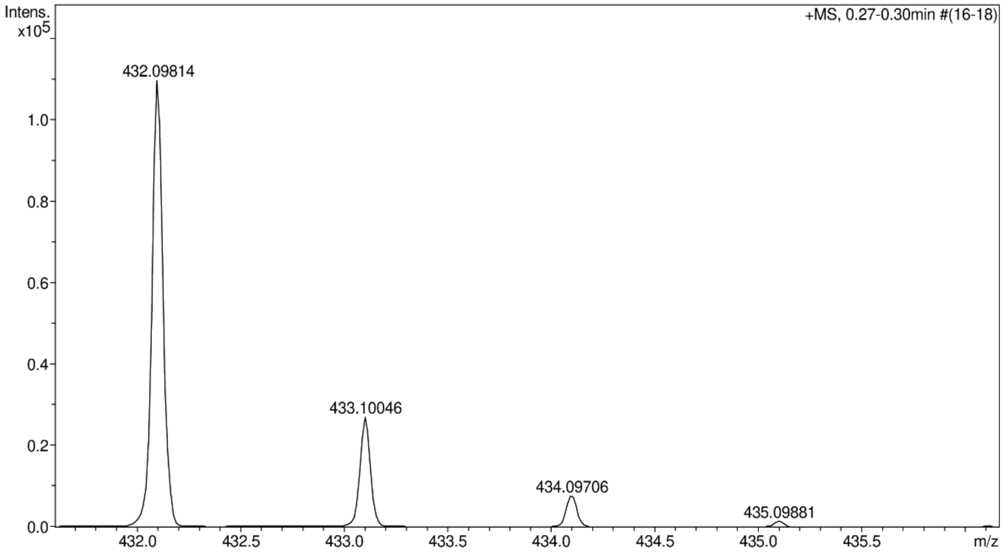


Bruker Compass DataAnalysis 4.0 printed: 12/19/2022 7:39:05 PM Page 1 of 1

**Figure S214** ESI-HRMS spectrum of **6f'**

# Generic Display Report

**Analysis Info**

| | | | |
|---|---|---|---|
| Analysis Name | D:\Data\Data Service\221219\NHTs+4-NO2-NCS_RC8_01_6644.d | Acquisition Date | 12/19/2022 2:56:03 PM |
| Method | nv_pos_5min_profile_190214.m | Operator | CU. |
| Sample Name | NHTs+4-NO2-NCS | Instrument | micrOTOF-Q |
| Comment | | | |

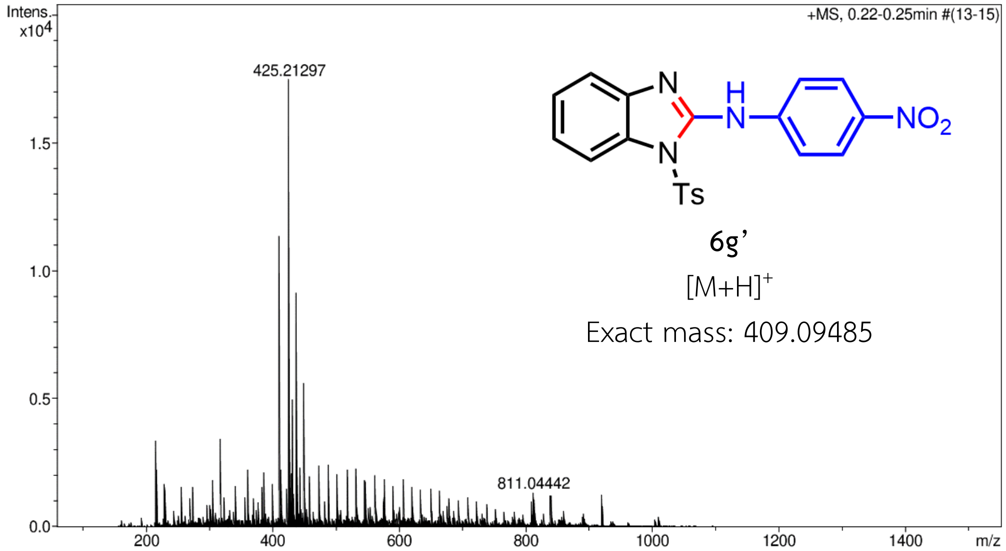


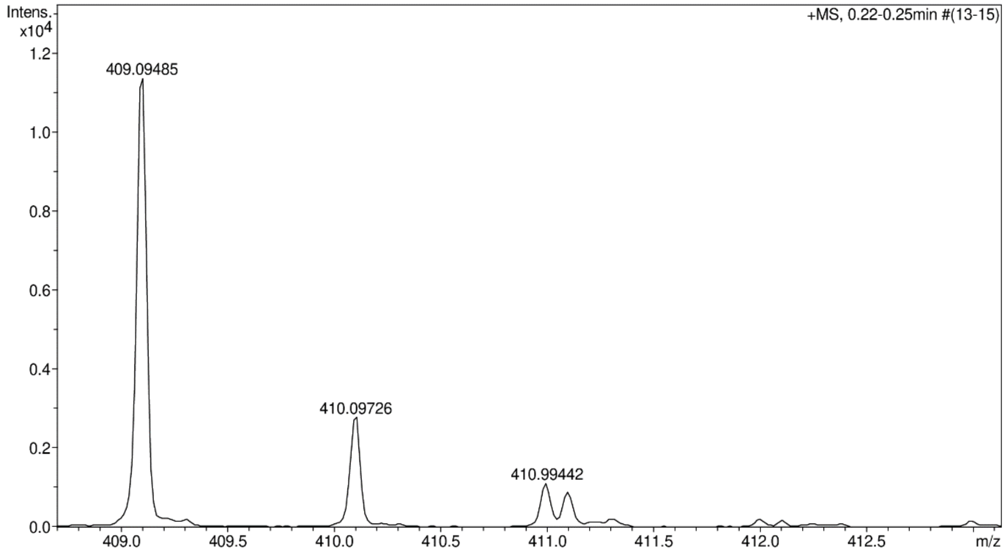


Bruker Compass DataAnalysis 4.0 printed: 12/19/2022 7:34:22 PM Page 1 of 1

**Figure S215** ESI-HRMS spectrum of **6g'**

## Generic Display Report

| | | | |
|---|---|---|---|
| **Analysis Info** | | Acquisition Date | 12/19/2022 2:49:36 PM |
| Analysis Name | D:\Data\Data Service\221219\NHTs+3-NO2-NCS_RC7_01_6643.d | | |
| Method | nv_pos_5min_profile_190214.m | Operator | CU. |
| Sample Name | NHTs+3-NO2-NCS | Instrument | micrOTOF-Q |
| Comment | | | |

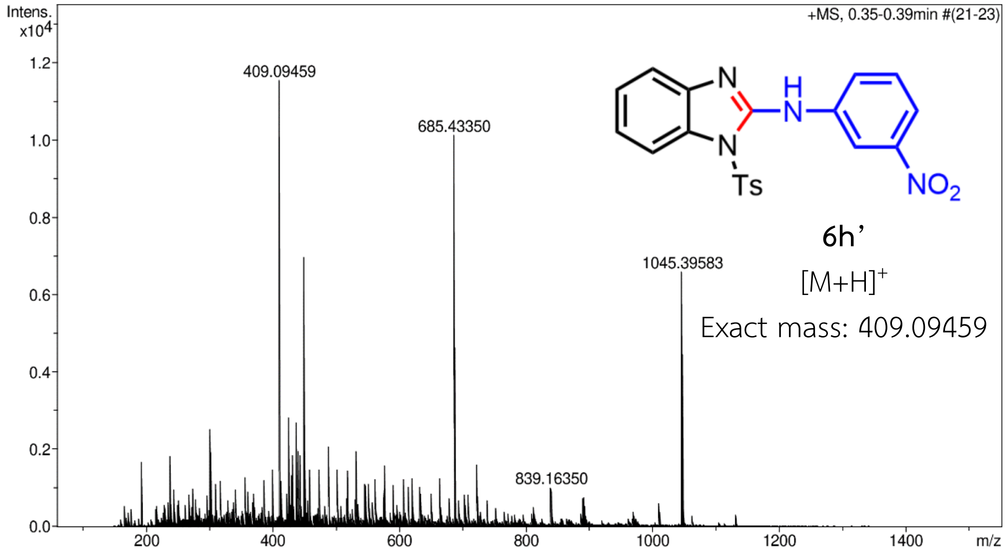


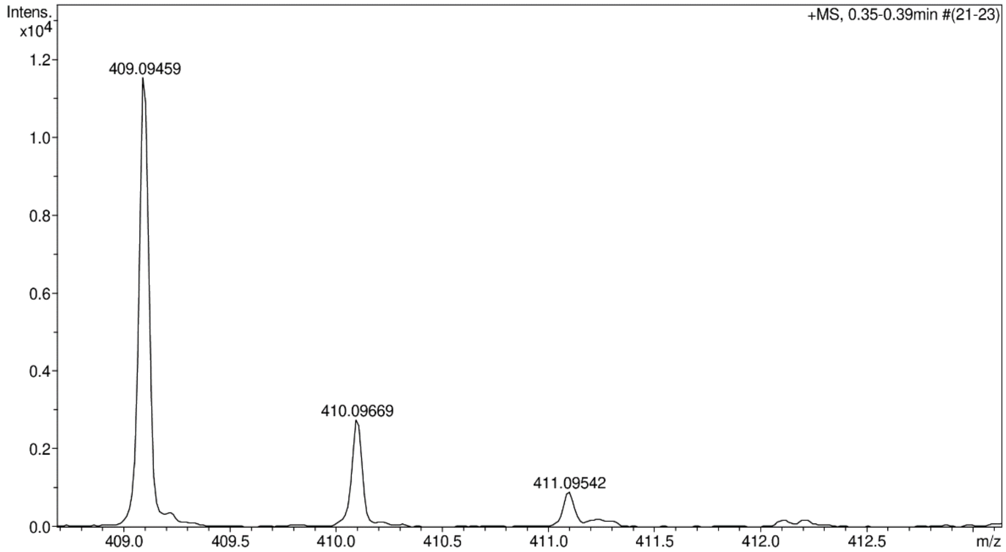


Bruker Compass DataAnalysis 4.0 printed: 12/19/2022 7:27:39 PM Page 1 of 1

**Figure S216** ESI-HRMS spectrum of **6h'**

# Generic Display Report

| Analysis Info | | Acquisition Date | 12/19/2022 3:28:10 PM |
|---|---|---|---|
| Analysis Name | D:\Data\Data Service\221219\NHTs+4-CN-NCS_RD5_01_6649.d | | |
| Method | nv_pos_5min_profile_190214.m | Operator | CU. |
| Sample Name | NHTs+4-CN-NCS | Instrument | micrOTOF-Q |
| Comment | | | |

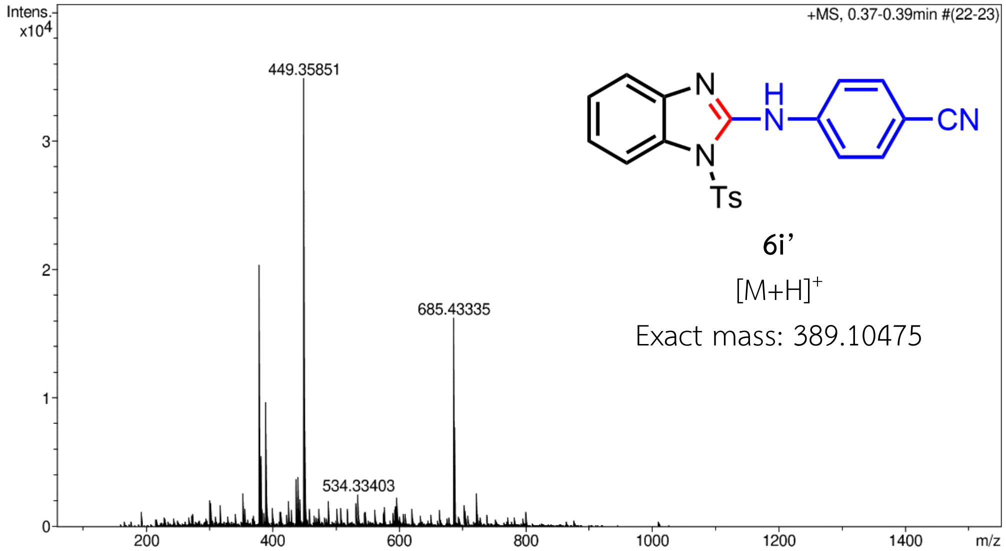


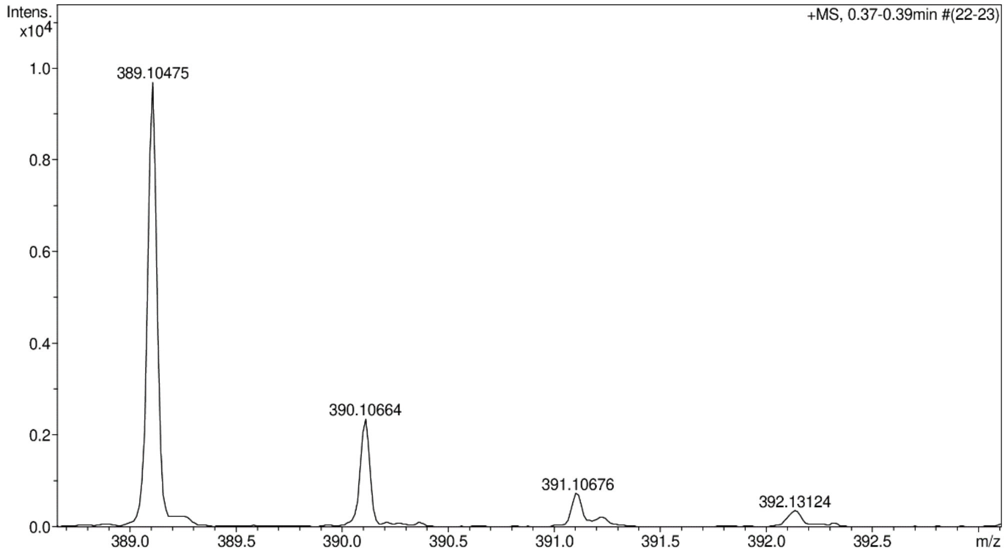


Bruker Compass DataAnalysis 4.0 printed: 12/19/2022 7:59:25 PM Page 1 of 1

**Figure S217** ESI-HRMS spectrum of **6i'**

## Generic Display Report

| | | | |
|---|---|---|---|
| **Analysis Info** | | Acquisition Date | 1/6/2023 5:59:43 PM |
| Analysis Name | D:\Data\Data Service\230106\NHTs+3-Cn-NCS_RB2_01_6682.d | | |
| Method | nv_pos_5min_profile_190214.m | Operator | CU. |
| Sample Name | NHTs+3-Cn-NCS | Instrument | micrOTOF-Q |
| Comment | | | |

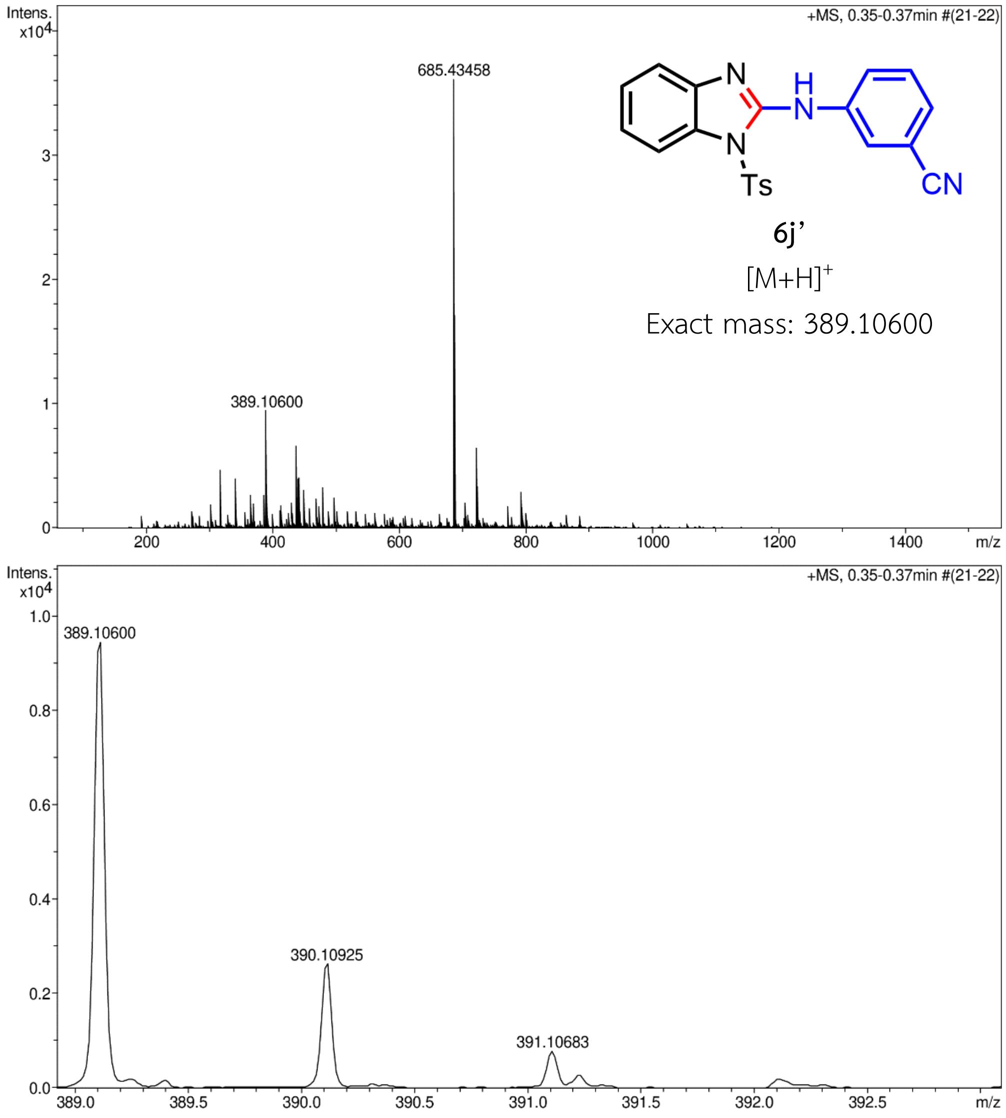


Bruker Compass DataAnalysis 4.0 printed: 1/6/2023 6:32:37 PM Page 1 of 1

**Figure S218** ESI-HRMS spectrum of **6j'**

## Generic Display Report

**Analysis Info**

| | | | |
|---|---|---|---|
| Analysis Name | D:\Data\Data Service\230106\NHTs+benzocaine-NCS_RA3_01_6679.d | Acquisition Date | 1/6/2023 5:40:21 PM |
| Method | nv_pos_5min_profile_190214.m | Operator | CU. |
| Sample Name | NHTs+benzocaine-NCS | Instrument | micrOTOF-Q |
| Comment | | | |

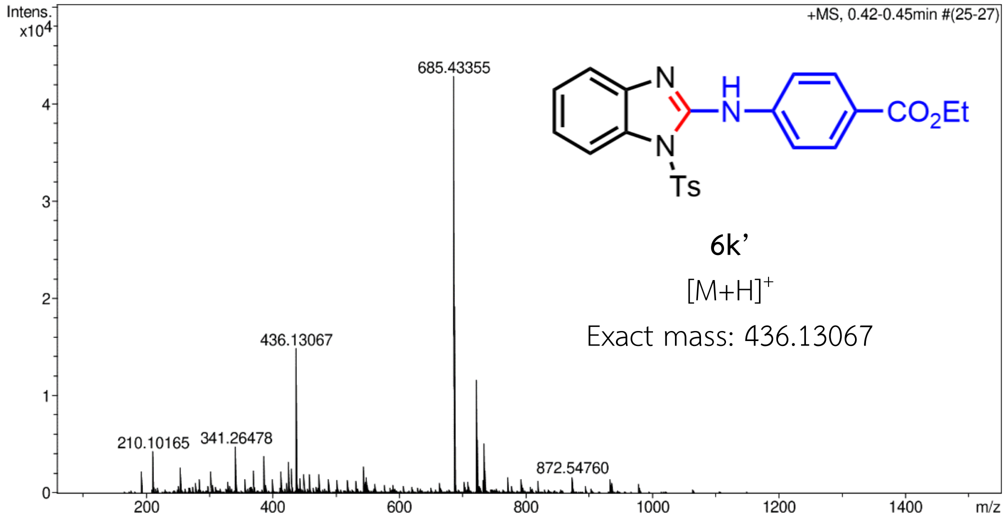


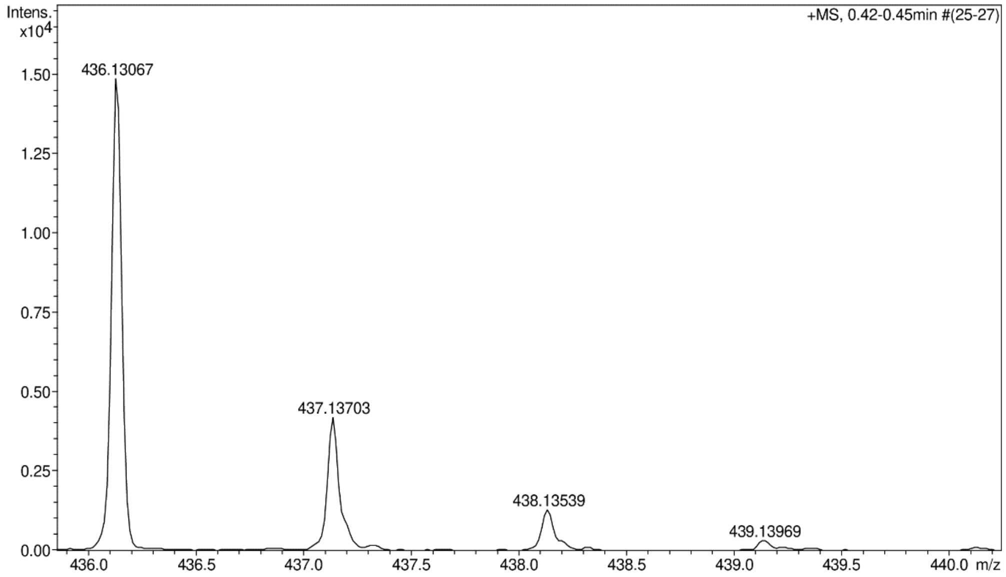


Bruker Compass DataAnalysis 4.0 printed: 1/6/2023 6:02:23 PM Page 1 of 1

**Figure S219** ESI-HRMS spectrum of **6k'**

# Generic Display Report

| **Analysis Info** | | Acquisition Date | 7/24/2023 12:52:53 PM |
| --- | --- | --- | --- |
| Analysis Name | D:\Data\Data Service\230724\light acetophenone_RA5_01_7299.d | | |
| Method | nv_pos_5min_profile_190214.m | Operator | CU. |
| Sample Name | light acetophenone | Instrument | micrOTOF-Q |
| Comment | | | |

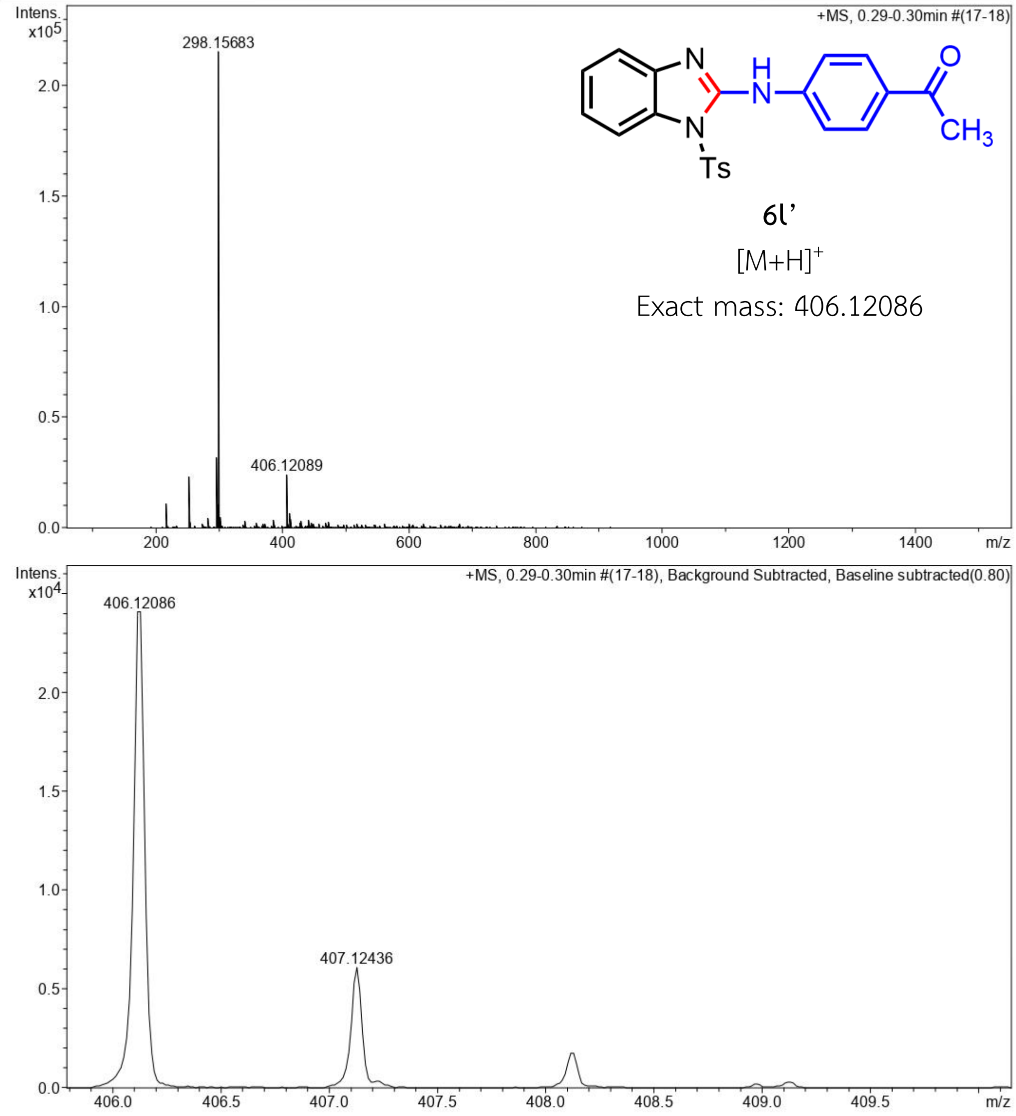


Bruker Compass DataAnalysis 4.0 printed: 7/24/2023 1:49:57 PM Page 1 of 1

**Figure S220** ESI-HRMS spectrum of **6l’**

# Generic Display Report

**Analysis Info**

| | | | |
|---|---|---|---|
| | | Acquisition Date | 12/19/2022 3:54:00 PM |
| Analysis Name | D:\Data\Data Service\221219\NHTs+1,4-di-NCS_RE1_01_6653.d | | |
| Method | nv_pos_5min_profile_190214.m | Operator | CU. |
| Sample Name | NHTs+1,4-di-NCS | Instrument | micrOTOF-Q |
| Comment | | | |

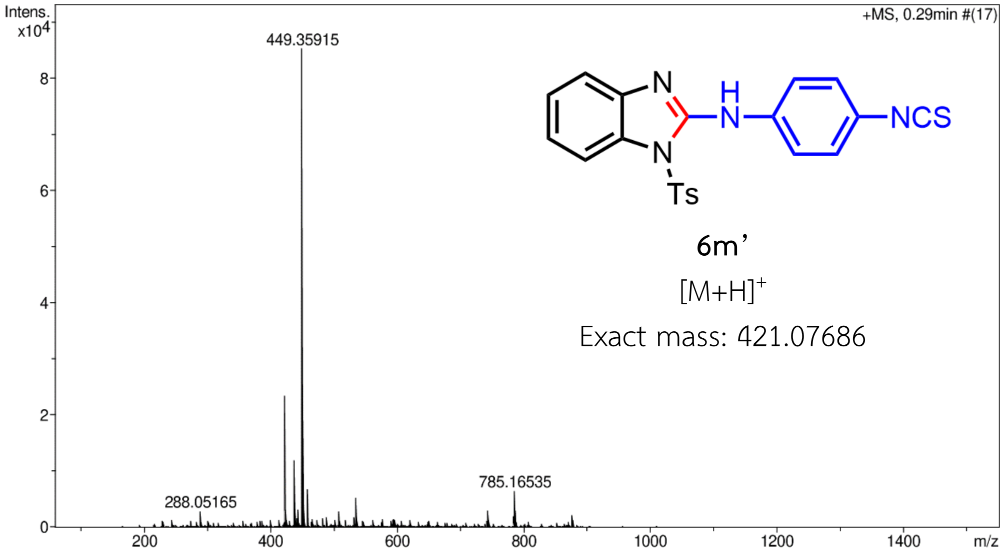


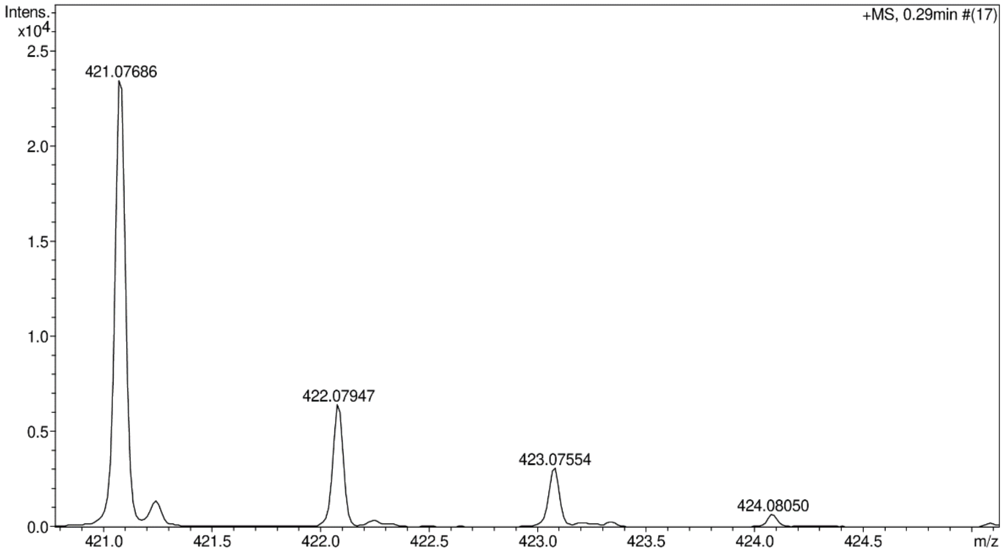


Bruker Compass DataAnalysis 4.0 printed: 12/19/2022 8:30:44 PM Page 1 of 1

**Figure S221** ESI-HRMS spectrum of **6m'**

# Generic Display Report

**Analysis Info**

| | | | |
|---|---|---|---|
| Analysis Name | D:\Data\Data Service\240304\NHTs+1,4-NCS-dimer_RA4_01_9034.d | Acquisition Date | 3/4/2024 1:58:39 PM |
| Method | nv_pos_5min_profile_190214.m | Operator | CU. |
| Sample Name | NHTs+1,4-NCS-dimer | Instrument | micrOTOF-Q |
| Comment | | | |

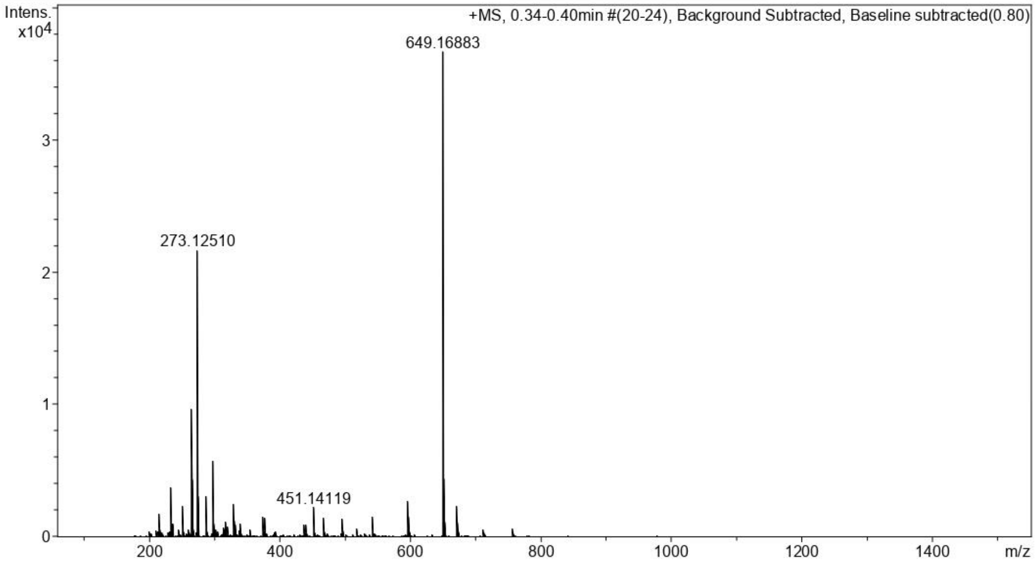


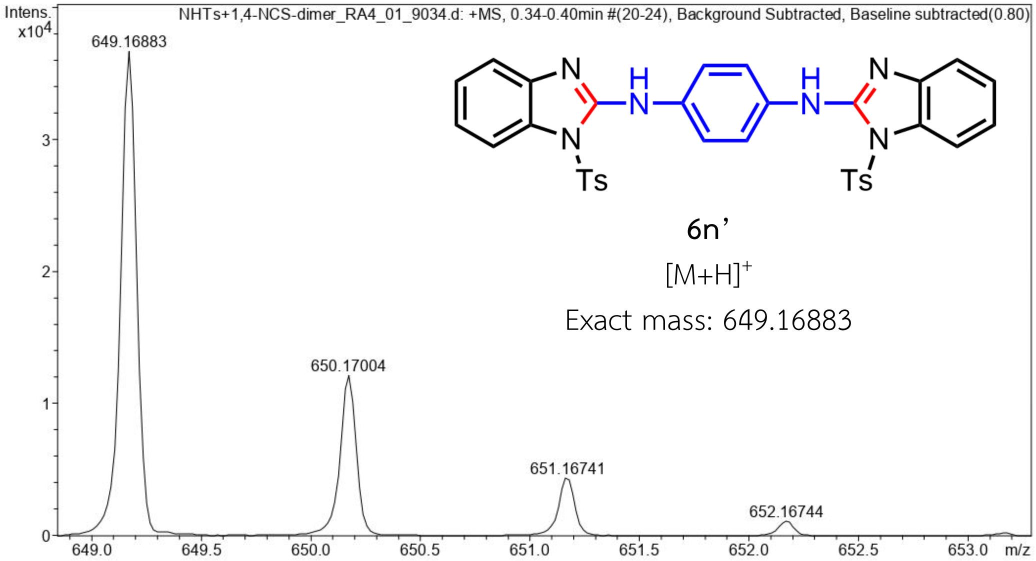


Bruker Compass DataAnalysis 4.0 printed: 3/4/2024 2:09:07 PM Page 1 of 1

**Figure S222** ESI-HRMS spectrum of **6n’**

## Generic Display Report

**Analysis Info**

| | | | |
|---|---|---|---|
| Analysis Name | D:\Data\Data Service\221219\NHTs+1-naphthyl-NCS_RB1_01_6628.d | Acquisition Date | 12/19/2022 1:12:44 PM |
| Method | nv_pos_5min_profile_190214.m | Operator | CU. |
| Sample Name | NHTs+1-naphthyl-NCS | Instrument | micrOTOF-Q |
| Comment | | | |

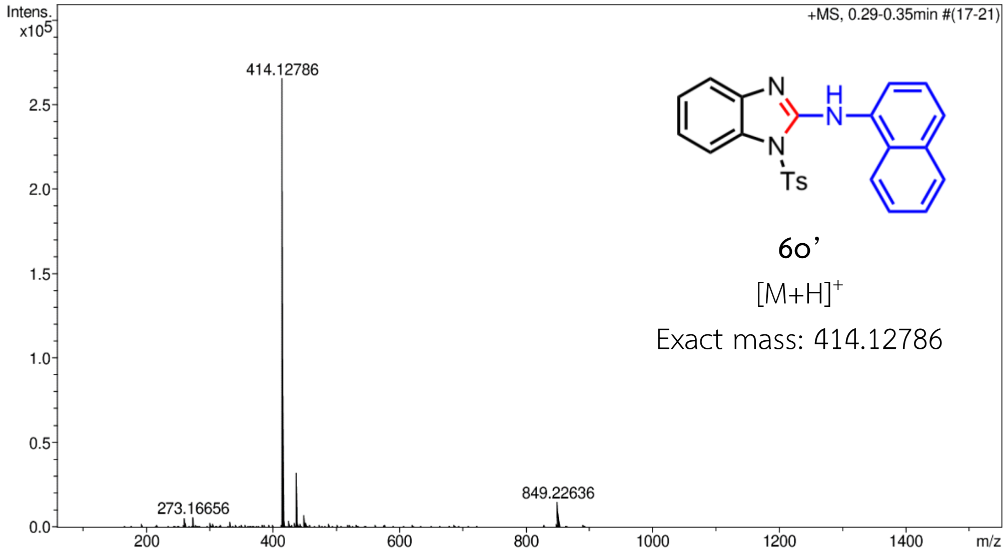


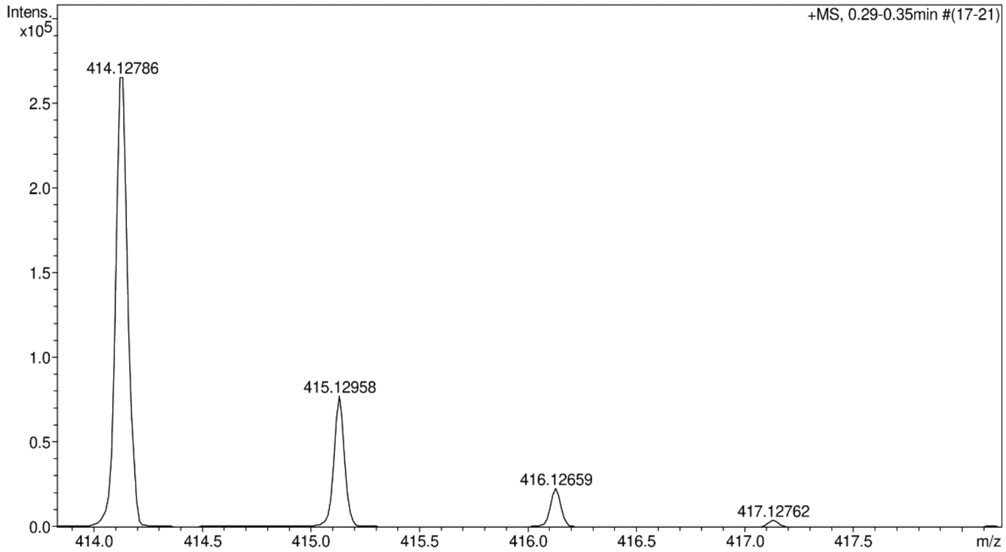


Bruker Compass DataAnalysis 4.0 printed: 12/19/2022 6:31:00 PM Page 1 of 1

**Figure S223** ESI-HRMS spectrum of **6o'**

## Generic Display Report

| Analysis Info | | Acquisition Date | 7/24/2023 12:46:35 PM |
|---|---|---|---|
| Analysis Name | D:\Data\Data Service\230724\light pyrene_RA4_01_7298.d | | |
| Method | nv_pos_5min_profile_190214.m | Operator | CU. |
| Sample Name | light pyrene | Instrument | micrOTOF-Q |
| Comment | | | |

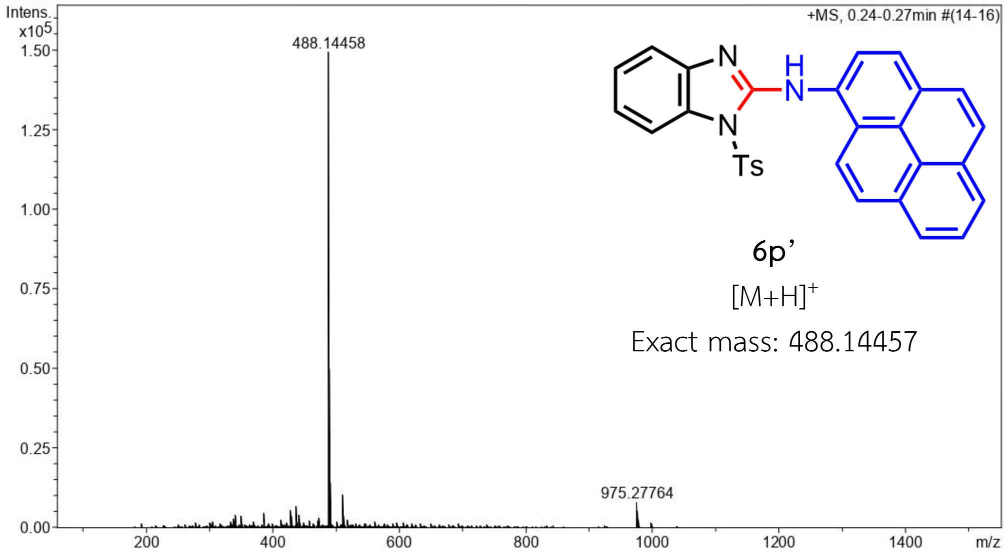


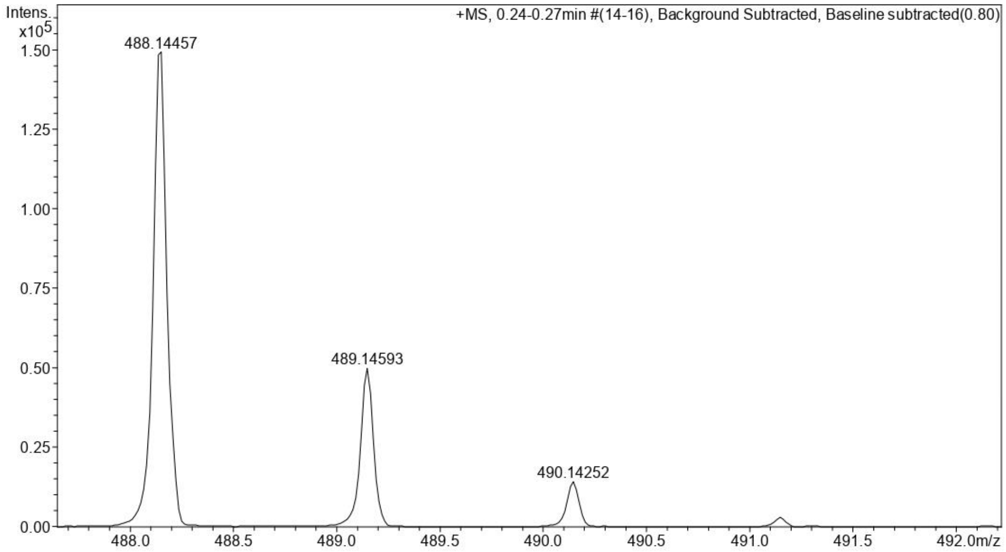


Bruker Compass DataAnalysis 4.0 printed: 7/24/2023 1:40:51 PM Page 1 of 1

**Figure S224** ESI-HRMS spectrum of **6p'**

## Generic Display Report

**Analysis Info**

| | | | |
|---|---|---|---|
| Analysis Name | D:\Data\Data Service\221219\NHTs+3-pyridyl-NCS_RC6_01_6641.d | Acquisition Date | 12/19/2022 2:36:41 PM |
| Method | nv_pos_5min_profile_190214.m | Operator | CU. |
| Sample Name | NHTs+3-pyridyl-NCS | Instrument | micrOTOF-Q |
| Comment | | | |

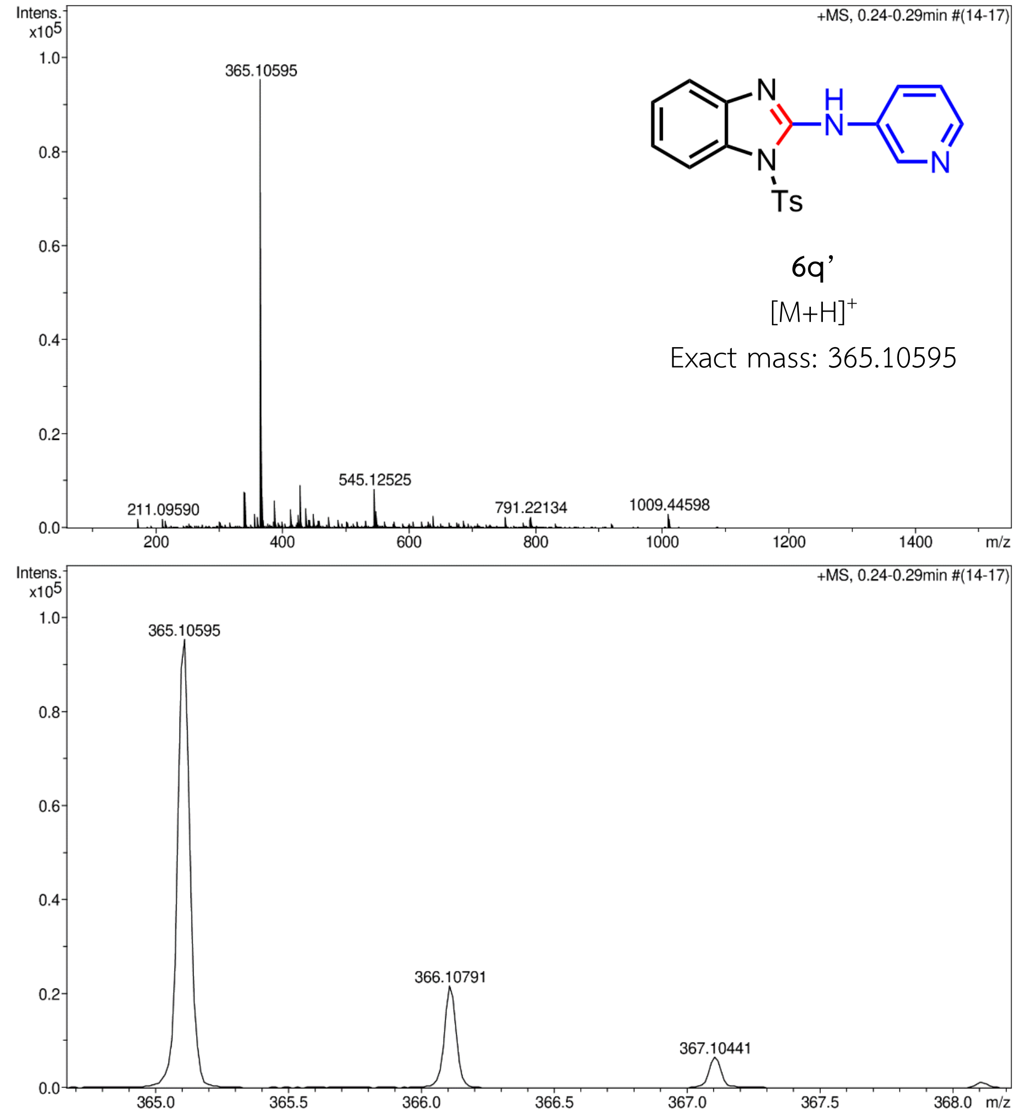


Bruker Compass DataAnalysis 4.0 printed: 12/19/2022 7:21:29 PM Page 1 of 1

**Figure S225** ESI-HRMS spectrum of **6q'**

# Generic Display Report

**Analysis Info**

| | | | |
|---|---|---|---|
| Analysis Name | D:\Data\Data Service\221219\NHTs+benzyl-NCS_RD2_01_6646.d | Acquisition Date | 12/19/2022 3:08:57 PM |
| Method | nv_pos_5min_profile_190214.m | Operator | CU. |
| Sample Name | NHTs+benzyl-NCS | Instrument | micrOTOF-Q |
| Comment | | | |

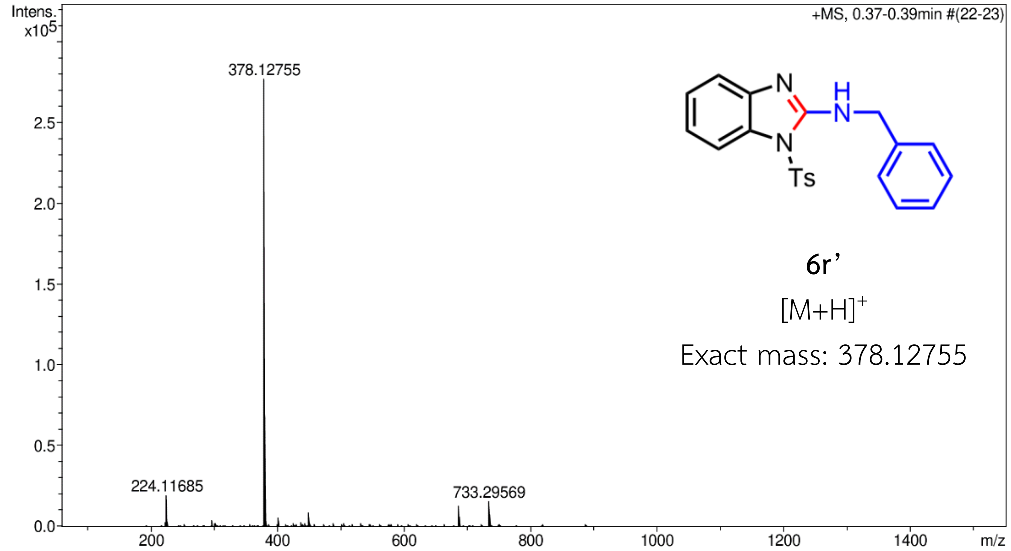


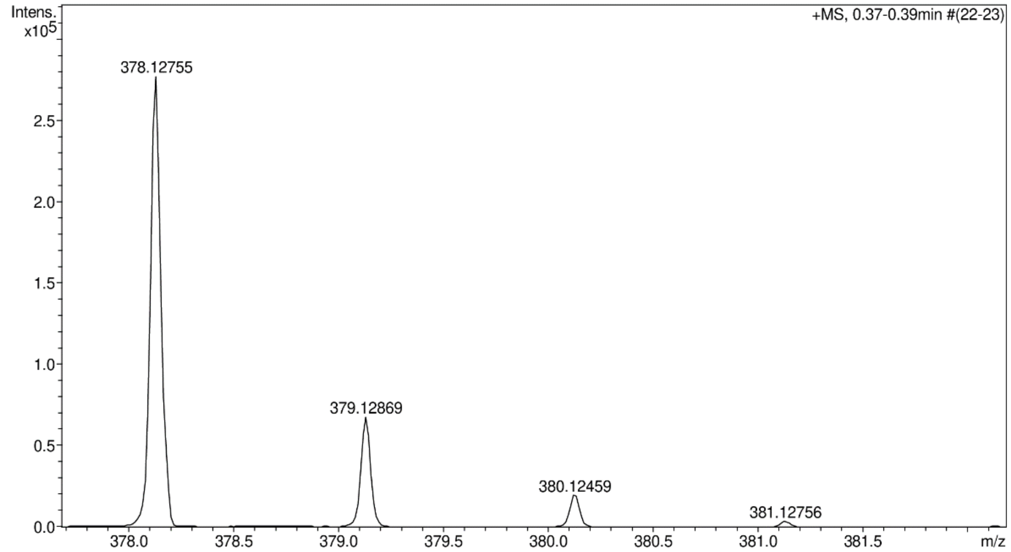


Bruker Compass DataAnalysis 4.0 printed: 12/19/2022 7:42:54 PM Page 1 of 1

**Figure S226** ESI-HRMS spectrum of **6r'**

# Generic Display Report

| **Analysis Info** | | Acquisition Date | 12/19/2022 3:15:16 PM |
|---|---|---|---|
| Analysis Name | D:\Data\Data Service\221219\NHTs+phenethyl-NCS_RD3_01_6647.d | | |
| Method | nv_pos_5min_profile_190214.m | Operator | CU. |
| Sample Name | NHTs+phenethyl-NCS | Instrument | micrOTOF-Q |
| Comment | | | |

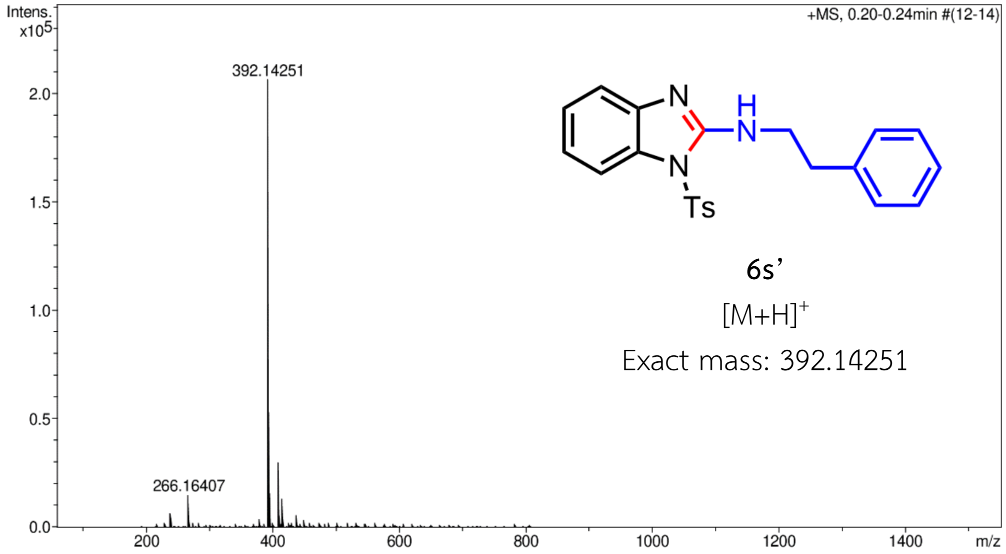


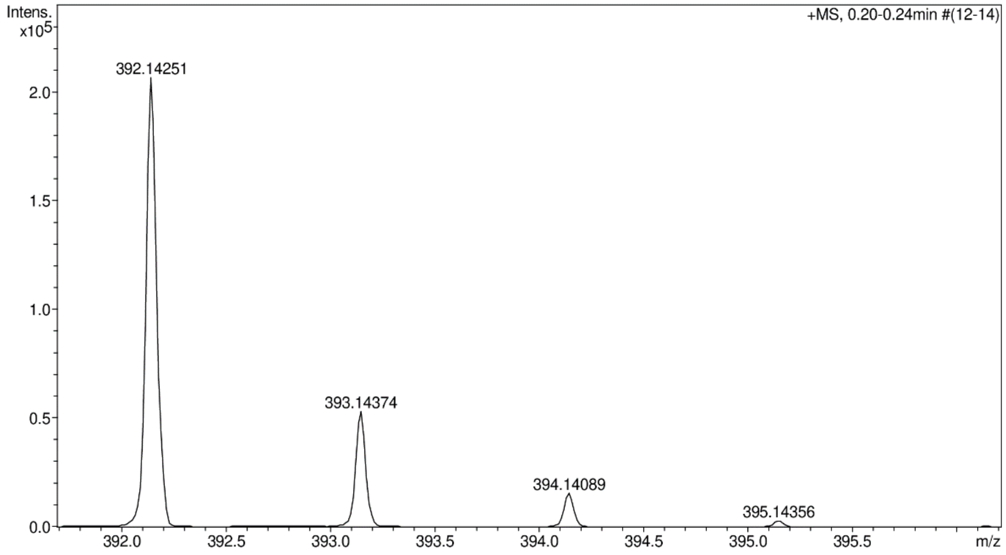


Bruker Compass DataAnalysis 4.0 printed: 12/19/2022 7:47:04 PM Page 1 of 1

**Figure S227** ESI-HRMS spectrum of **6s'**

## Generic Display Report

| | | | |
|---|---|---|---|
| **Analysis Info** | | Acquisition Date | 9/25/2023 11:23:30 AM |
| Analysis Name | D:\Data\Data Service\230925\NHTs+furan-NCS_RB1_01_7737.d | | |
| Method | nv_pos_5min_profile_190214.m | Operator | CU. |
| Sample Name | NHTs+furan-NCS | Instrument | micrOTOF-Q |
| Comment | | | |

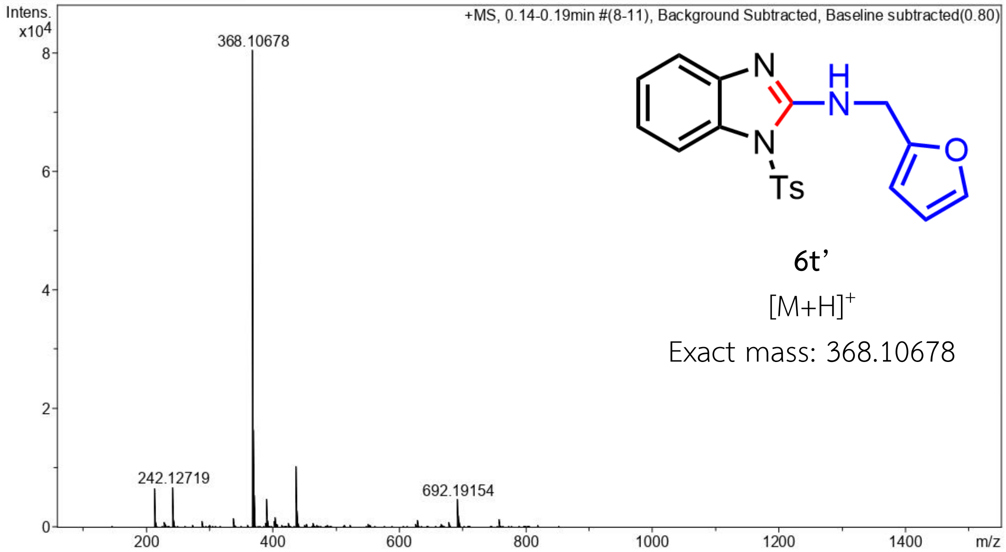


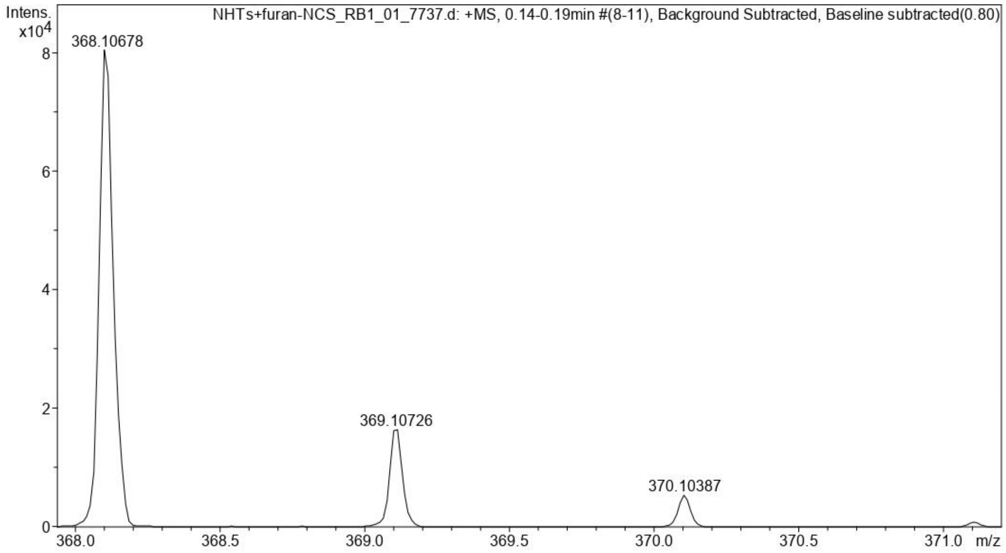


Bruker Compass DataAnalysis 4.0 printed: 9/25/2023 5:51:22 PM Page 1 of 1

**Figure S228** ESI-HRMS spectrum of **6t'**

# Generic Display Report

**Analysis Info**

| | | | |
|---|---|---|---|
| Analysis Name | D:\Data\Data Service\230124\NHTs+ethyl NCS_RB2_01_6711.d | Acquisition Date | 1/24/2023 4:55:19 PM |
| Method | nv_pos_5min_profile_190214.m | Operator | CU. |
| Sample Name | NHTs+ethyl NCS | Instrument | micrOTOF-Q |
| Comment | | | |

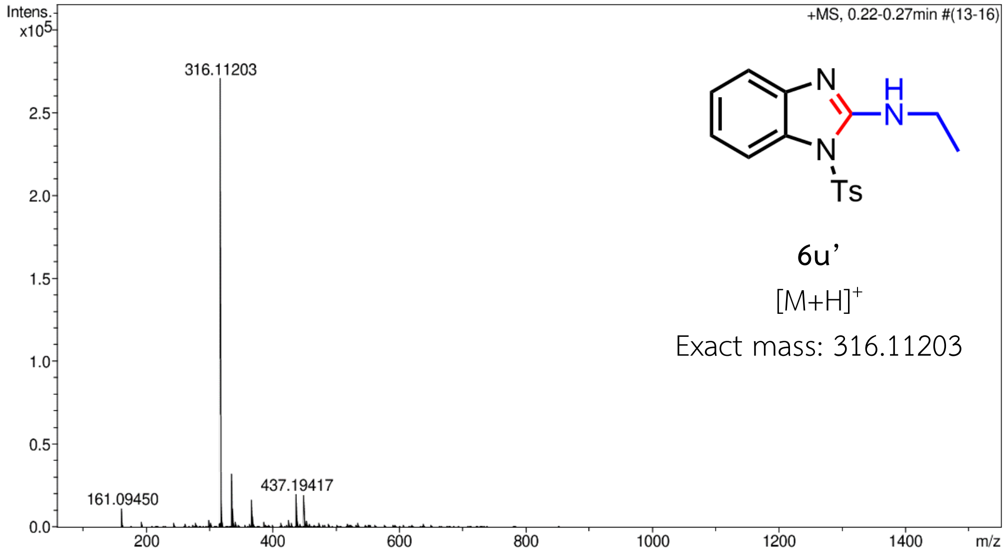


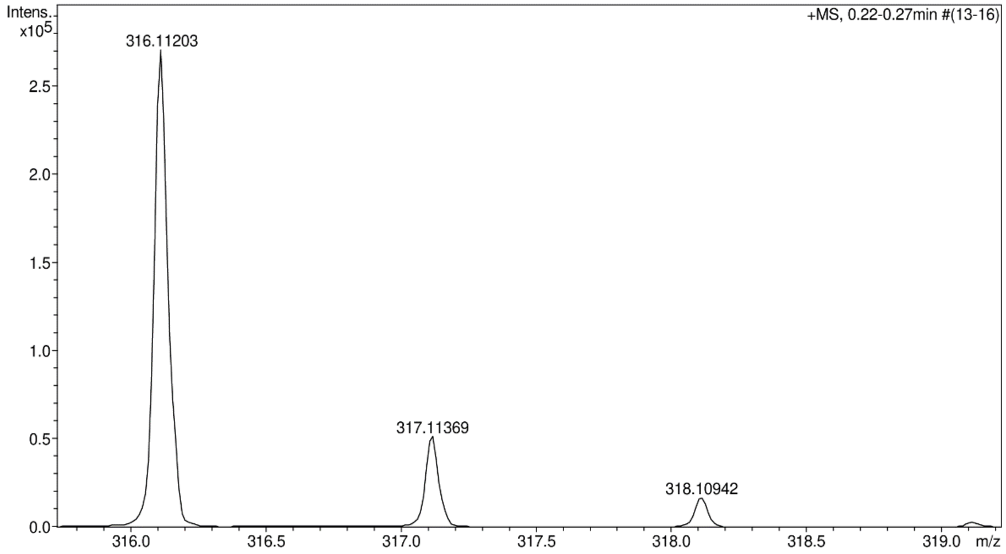


Bruker Compass DataAnalysis 4.0 printed: 1/24/2023 5:44:57 PM Page 1 of 1

**Figure S229** ESI-HRMS spectrum of **6u'**

# Generic Display Report

**Analysis Info**

| | | | |
|---|---|---|---|
| Analysis Name | D:\Data\Data Service\230124\NHTs+isopropyl-NCS_RB3_01_6712.d | Acquisition Date | 1/24/2023 5:01:46 PM |
| Method | nv_pos_5min_profile_190214.m | Operator | CU. |
| Sample Name | NHTs+isopropyl-NCS | Instrument | micrOTOF-Q |
| Comment | | | |

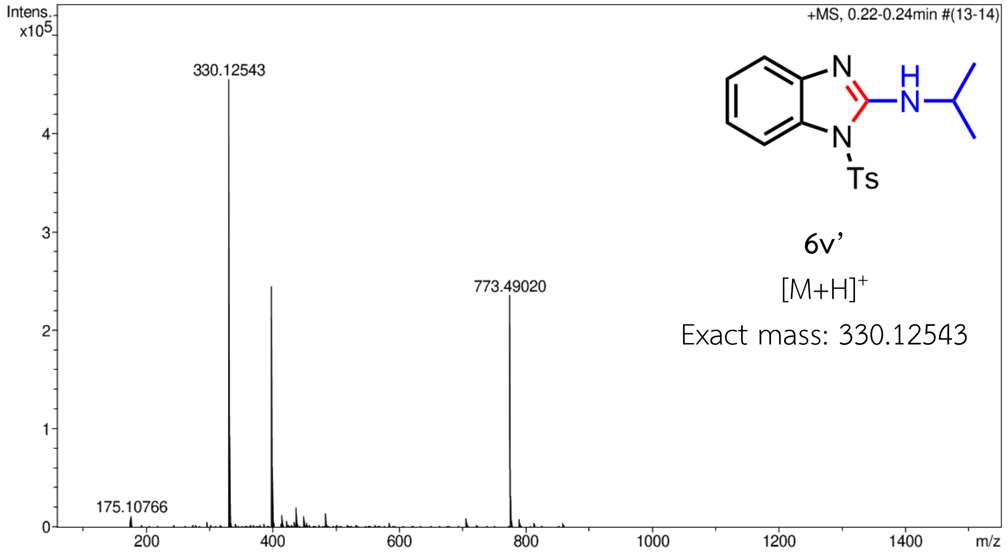


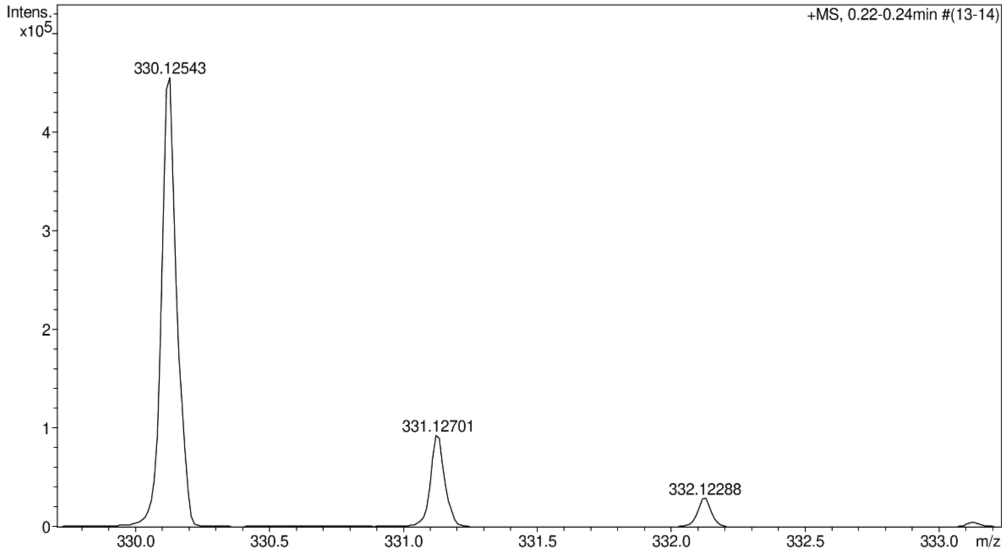


Bruker Compass DataAnalysis 4.0 printed: 1/24/2023 6:56:05 PM Page 1 of 1

**Figure S230** ESI-HRMS spectrum of **6v’**

## Generic Display Report

| | | | |
|---|---|---|---|
| **Analysis Info** | | Acquisition Date | 7/1/2024 11:44:17 AM |
| Analysis Name | D:\Data\Data Service\240701\NHTs+octyl NCS_RA5_01_9121.d | | |
| Method | nv_pos_5min_profile_190214.m | Operator | CU. |
| Sample Name | NHTs+octyl NCS | Instrument | micrOTOF-Q |
| Comment | | | |

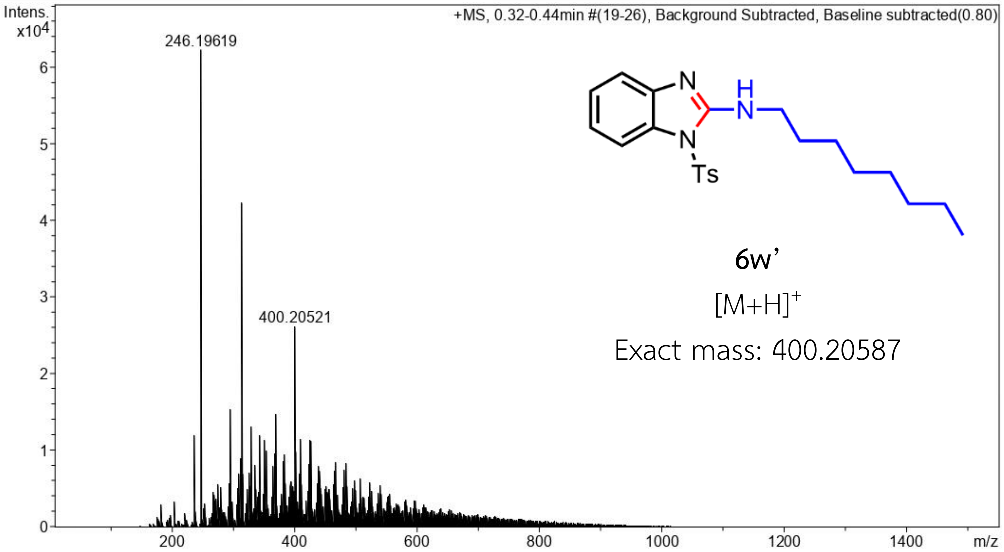


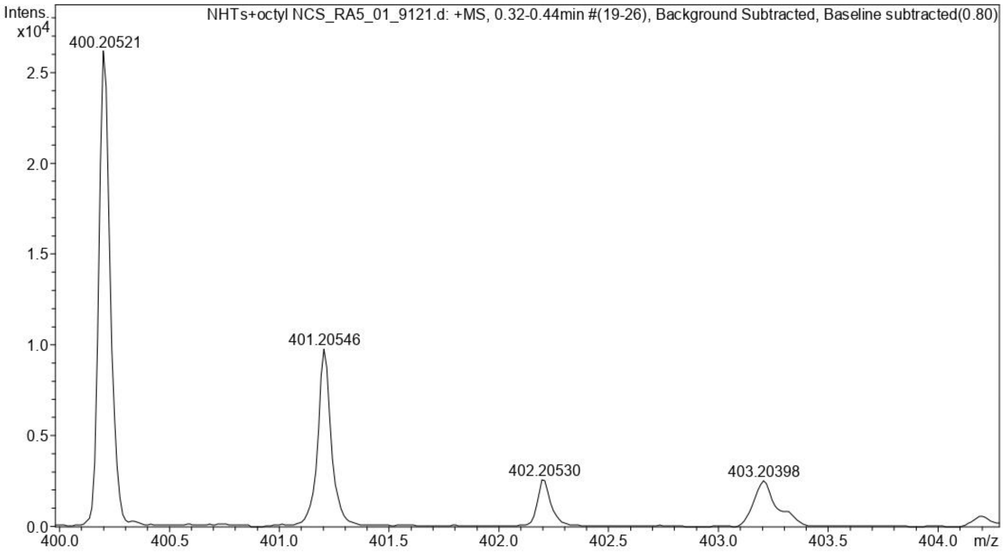


Bruker Compass DataAnalysis 4.0 printed: 7/1/2024 3:48:34 PM Page 1 of 1

**Figure S231** ESI-HRMS spectrum of **6w'**

## Generic Display Report

| | | | |
|---|---|---|---|
| **Analysis Info** | | Acquisition Date | 1/24/2023 5:08:14 PM |
| Analysis Name | D:\Data\Data Service\230124\NHTs+cyclohexyl-NCS_RB4_01_6713.d | | |
| Method | nv_pos_5min_profile_190214.m | Operator | CU. |
| Sample Name | NHTs+cyclohexyl-NCS | Instrument | micrOTOF-Q |
| Comment | | | |

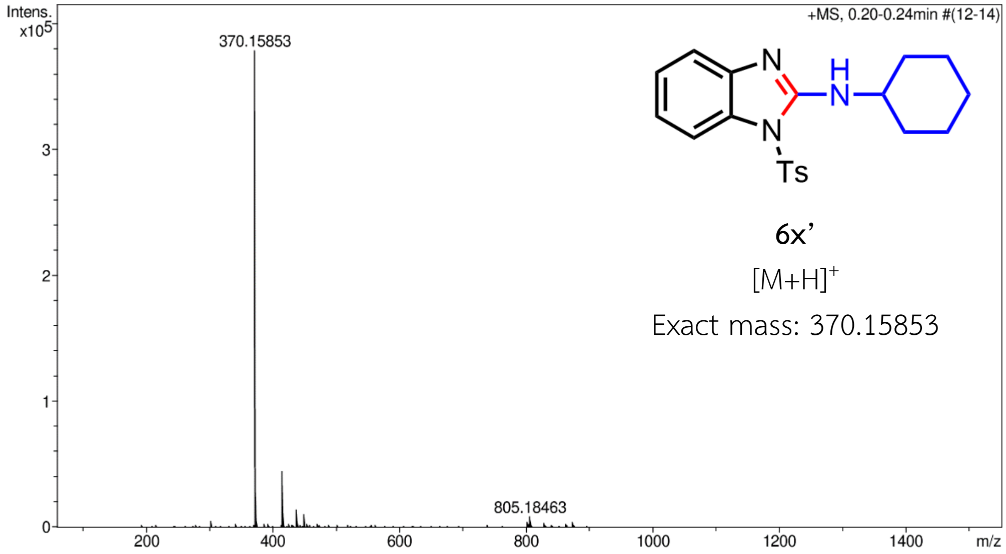


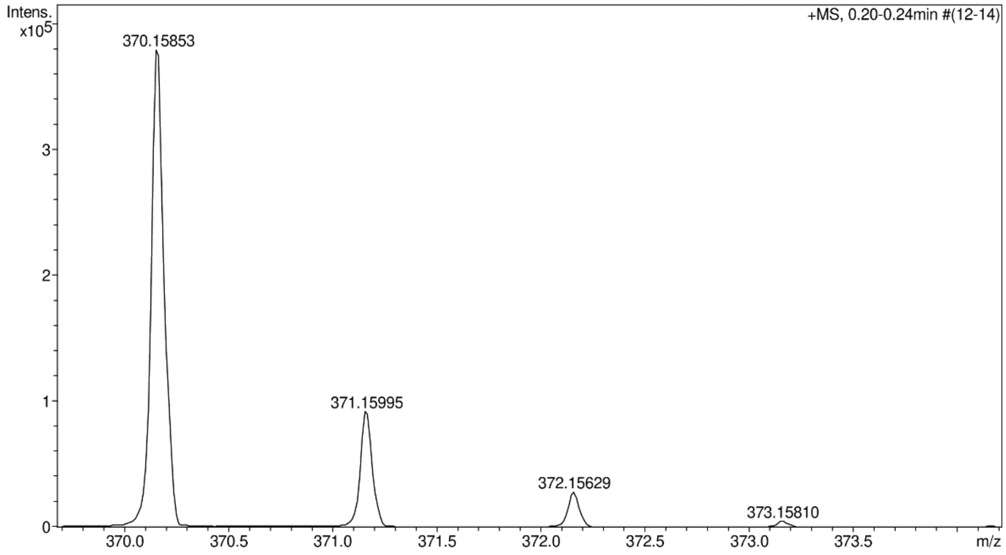




**Figure S232** ESI-HRMS spectrum of **6x'**

## Generic Display Report

| Analysis Info | | Acquisition Date | 7/15/2024 9:48:12 AM |
|---|---|---|---|
| Analysis Name | D:\Data\Data Service\240715\NHTs+adamantyl NCS_RA3_01_9190.d | | |
| Method | nv_pos_5min_profile_190214.m | Operator | CU. |
| Sample Name | NHTs+adamantyl NCS | Instrument | micrOTOF-Q |
| Comment | | | |

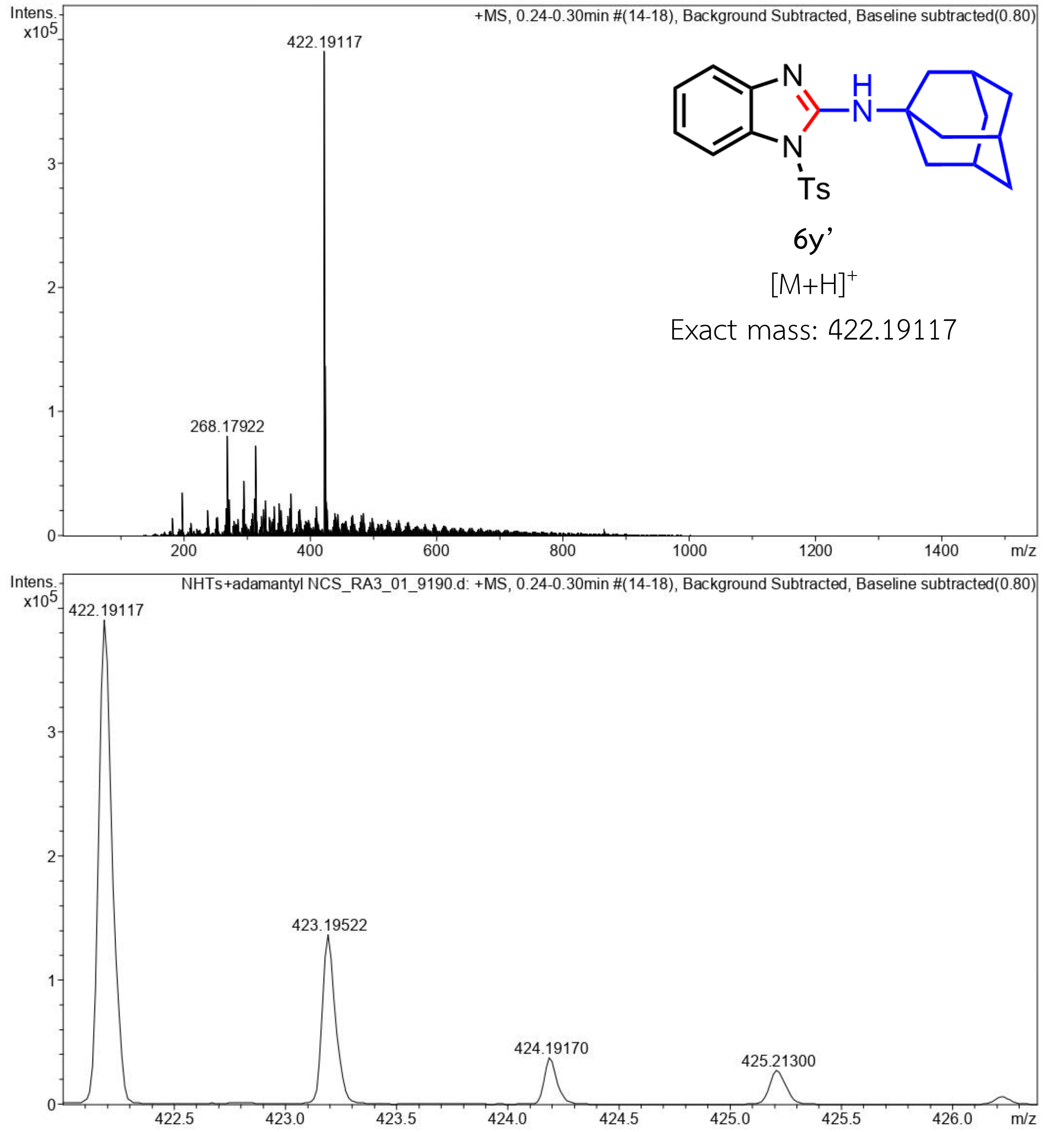


Bruker Compass DataAnalysis 4.0 printed: 7/15/2024 9:57:49 AM Page 1 of 1

**Figure S233** ESI-HRMS spectrum of **6y'**

# Mass Spectrum List Report

**Analysis Info**

| | | | |
|---|---|---|---|
| Analysis Name | D:\Data\Data Service\230925\4,5-diCl-Ts+4-Cl-NCS_RA2_01_7729.d | Acquisition Date | 9/25/2023 10:32:00 AM |
| Method | nv_pos_5min_profile_190214.m | Operator | CU. |
| Sample Name | 4,5-diCl-Ts+4-Cl-NCS | Instrument / Ser# | micrOTOF-Q 10335 |
| Comment | | | |

**Acquisition Parameter**

| | | | | | |
|---|---|---|---|---|---|
| Source Type | ESI | Ion Polarity | Positive | Set Nebulizer | 3.0 Bar |
| Focus | Not active | Set Capillary | 4000 V | Set Dry Heater | 200 ℃ |
| Scan Begin | 100 m/z | Set End Plate Offset | -500 V | Set Dry Gas | 8.0 l/min |
| Scan End | 1500 m/z | Set Collision Cell RF | 250.0 Vpp | Set Divert Valve | Waste |

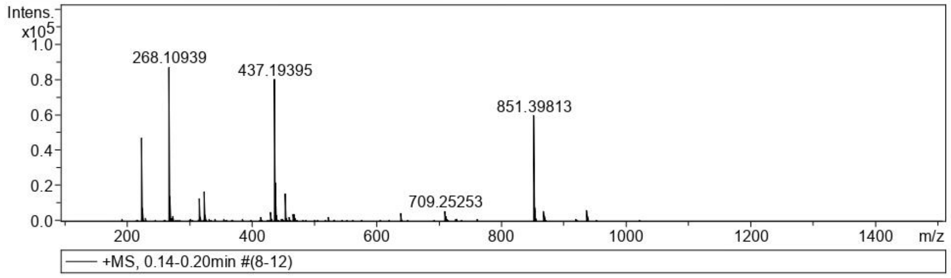


| # | m/z | Res. | S/N | I | FWHM |
|---|---|---|---|---|---|
| 1 | 224.11845 | 6547 | 3687.9 | 47351 | 0.03423 |
| 2 | 225.12019 | 7070 | 563.2 | 7250 | 0.03184 |
| 3 | 268.10939 | 6609 | 6031.3 | 87009 | 0.04057 |
| 4 | 269.11053 | 6993 | 955.7 | 13828 | 0.03848 |
| 5 | 273.16773 | 6939 | 183.0 | 2681 | 0.03937 |
| 6 | 316.21003 | 7714 | 792.7 | 12912 | 0.04099 |
| 7 | 317.21150 | 7493 | 149.9 | 2453 | 0.04234 |
| 8 | 324.16999 | 7549 | 1007.3 | 16715 | 0.04294 |
| 9 | 325.17210 | 7607 | 206.6 | 3441 | 0.04275 |
| 10 | 430.24246 | 8349 | 188.1 | 4914 | 0.05153 |
| 11 | 437.19395 | 7388 | 3196.2 | 80316 | 0.05918 |
| 12 | 438.19551 | 8304 | 857.5 | 21444 | 0.05277 |
| 13 | 439.19683 | 8668 | 140.1 | 3498 | 0.05067 |
| 14 | 453.16638 | 8469 | 686.8 | 15805 | 0.05351 |
| 15 | 454.16874 | 8652 | 167.8 | 3849 | 0.05249 |
| 16 | 460.26216 | 8148 | 110.4 | 2448 | 0.05649 |
| 17 | 465.99157 | 8470 | 191.3 | 4084 | 0.05501 |
| 18 | 467.98864 | 9140 | 203.2 | 4284 | 0.05120 |
| 19 | 522.16736 | 8758 | 157.5 | 2259 | 0.05962 |
| 20 | 638.31824 | 8926 | 330.1 | 4316 | 0.07152 |
| 21 | 709.25253 | 9123 | 410.2 | 5748 | 0.07775 |
| 22 | 710.25319 | 9321 | 176.6 | 2477 | 0.07620 |
| 23 | 711.25177 | 9100 | 225.5 | 3148 | 0.07816 |
| 24 | 851.39813 | 8187 | 3724.6 | 59882 | 0.10400 |
| 25 | 852.39985 | 8636 | 1911.1 | 30949 | 0.09870 |
| 26 | 853.39930 | 9346 | 499.0 | 8149 | 0.09131 |
| 27 | 867.36874 | 9205 | 301.4 | 5404 | 0.09423 |
| 28 | 868.37102 | 9135 | 150.9 | 2731 | 0.09506 |
| 29 | 936.37214 | 9780 | 407.3 | 5982 | 0.09575 |
| 30 | 937.37197 | 9740 | 196.9 | 2869 | 0.09624 |

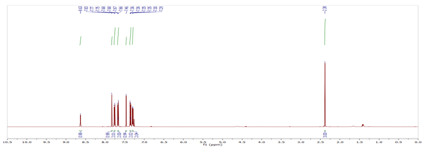

**6a''**

$[M+H]^+$

Exact mass: 465.99157

Bruker Compass DataAnalysis 4.0 printed: 9/25/2023 3:56:07 PM Page 1 of 1

**Figure S234** ESI-HRMS spectrum of **6a''**

# Generic Display Report

| | | | |
|---|---|---|---|
| **Analysis Info** | | Acquisition Date | 9/18/2023 3:11:37 PM |
| Analysis Name | D:\Data\Data Service\230918\4,5-diMe-Ts+4-Cl-NCS_RA6_01_7721.d | | |
| Method | nv_pos_5min_profile_190214.m | Operator | CU. |
| Sample Name | 4,5-diMe-Ts+4-Cl-NCS | Instrument | micrOTOF-Q |
| Comment | | | |

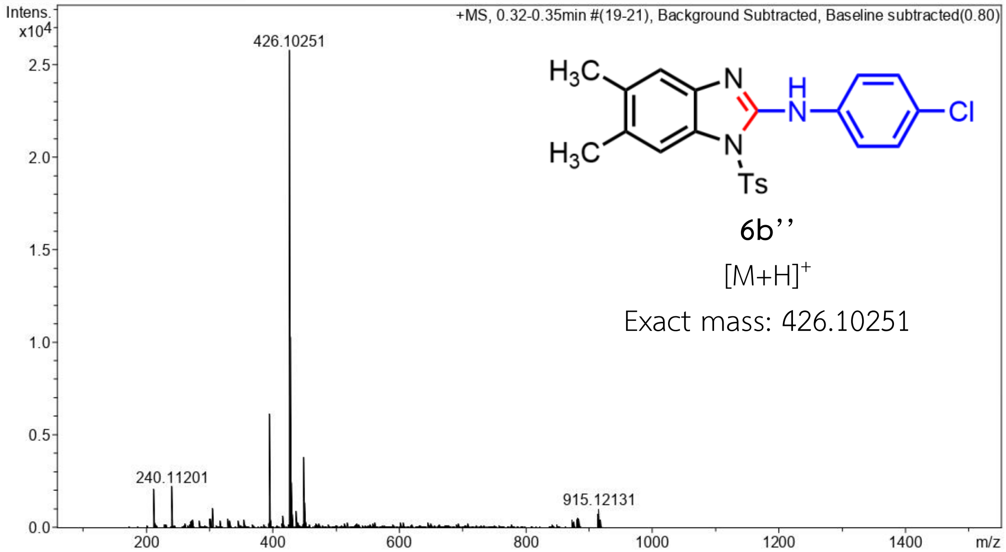


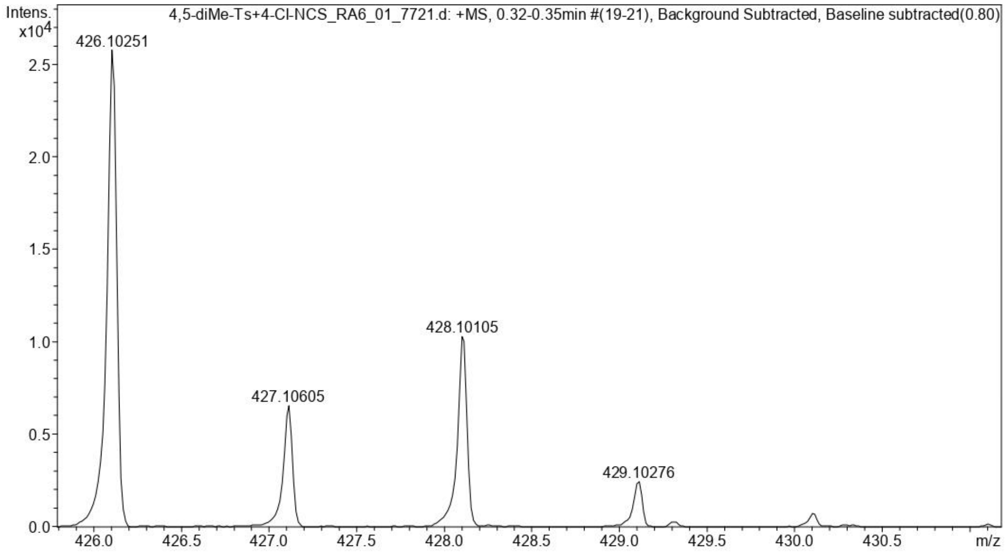


Bruker Compass DataAnalysis 4.0 printed: 9/18/2023 4:31:24 PM Page 1 of 1

**Figure S235** ESI-HRMS spectrum of **6b''**

# Generic Display Report

**Analysis Info**

| | | | |
|---|---|---|---|
| Analysis Name | D:\Data\Data Service\230925\2,3-NH-nap-Ts+4-Cl-NCS_RA5_01_7732.d | Acquisition Date | 9/25/2023 10:51:22 AM |
| Method | nv_pos_5min_profile_190214.m | Operator | CU. |
| Sample Name | 2,3-NH-nap-Ts+4-Cl-NCS | Instrument | micrOTOF-Q |
| Comment | | | |

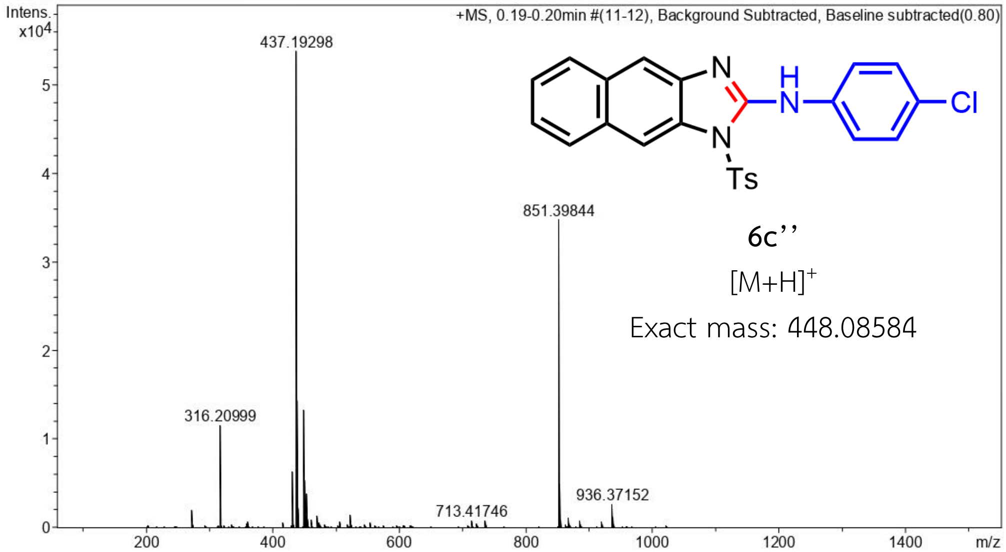


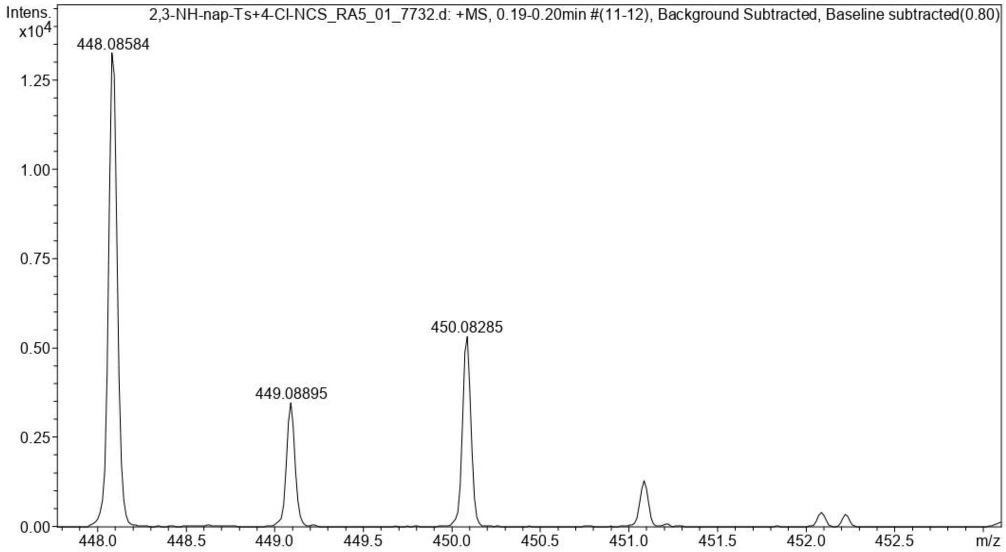


Bruker Compass DataAnalysis 4.0 printed: 9/25/2023 4:37:32 PM Page 1 of 1

**Figure S236** ESI-HRMS spectrum of **6c''**

# Generic Display Report

**Analysis Info**

| | | | |
|---|---|---|---|
| Analysis Name | D:\Data\Data Service\230213\light-target-1F_RA6_01_6752.d | Acquisition Date | 2/13/2023 12:31:01 PM |
| Method | nv_pos_5min_profile_190214.m | Operator | CU. |
| Sample Name | light-target-1F | Instrument | micrOTOF-Q |
| Comment | | | |

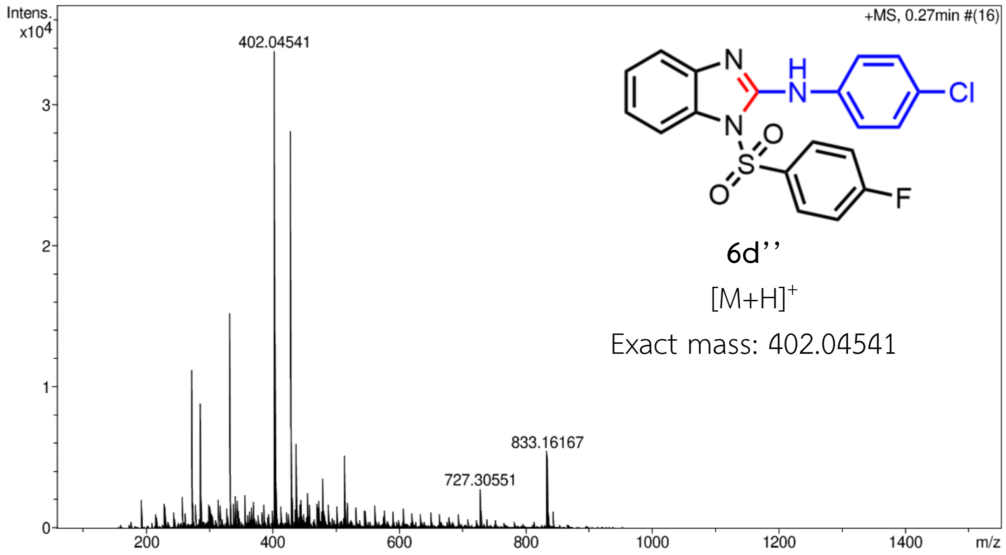


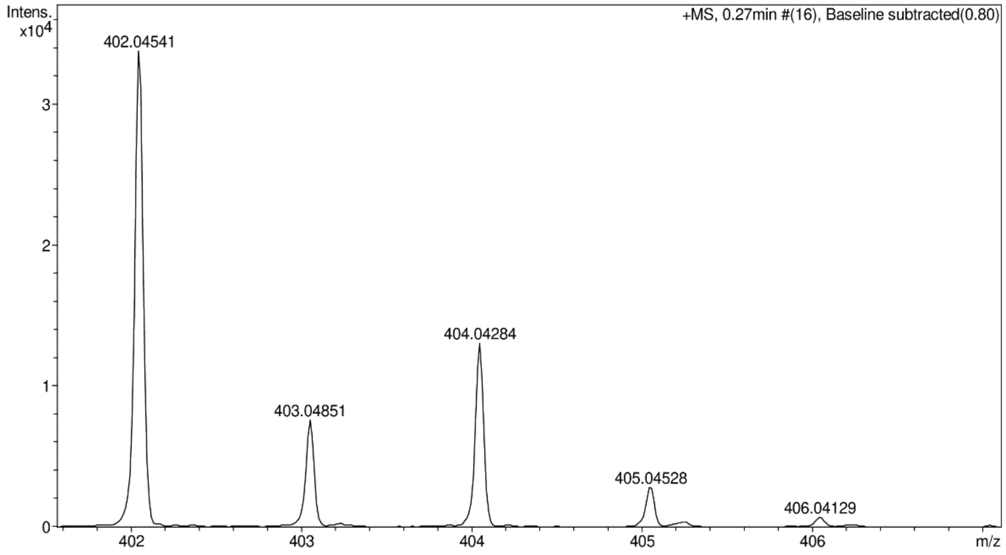


Bruker Compass DataAnalysis 4.0 printed: 2/13/2023 3:18:50 PM Page 1 of 1

**Figure S237** ESI-HRMS spectrum of **6d''**

## Generic Display Report

| Analysis Info | | Acquisition Date | 2/13/2023 12:43:47 PM |
| --- | --- | --- | --- |
| Analysis Name | D:\Data\Data Service\230213\light-target-2Cl_RA7_01_6754.d | | |
| Method | nv_pos_5min_profile_190214.m | Operator | CU. |
| Sample Name | light-target-2Cl | Instrument | micrOTOF-Q |
| Comment | | | |

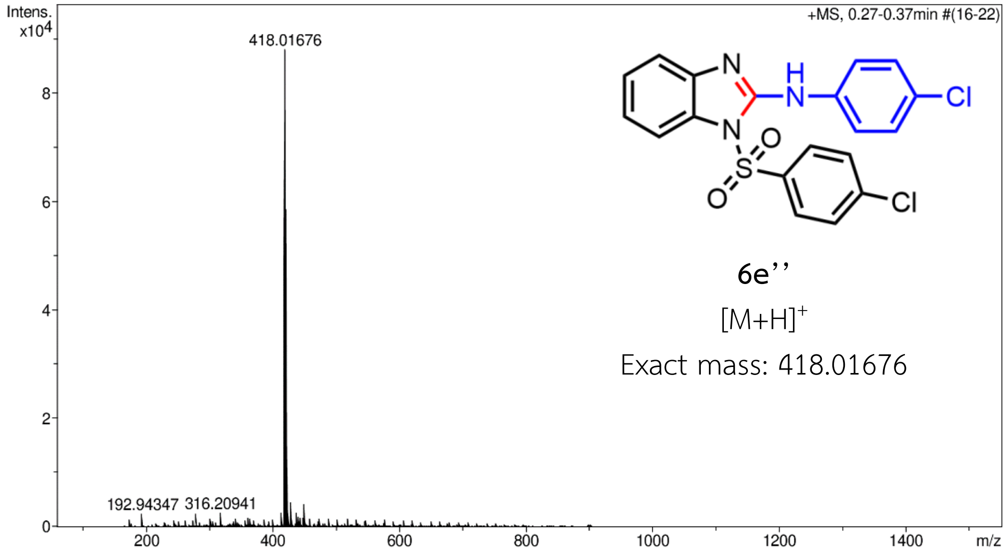


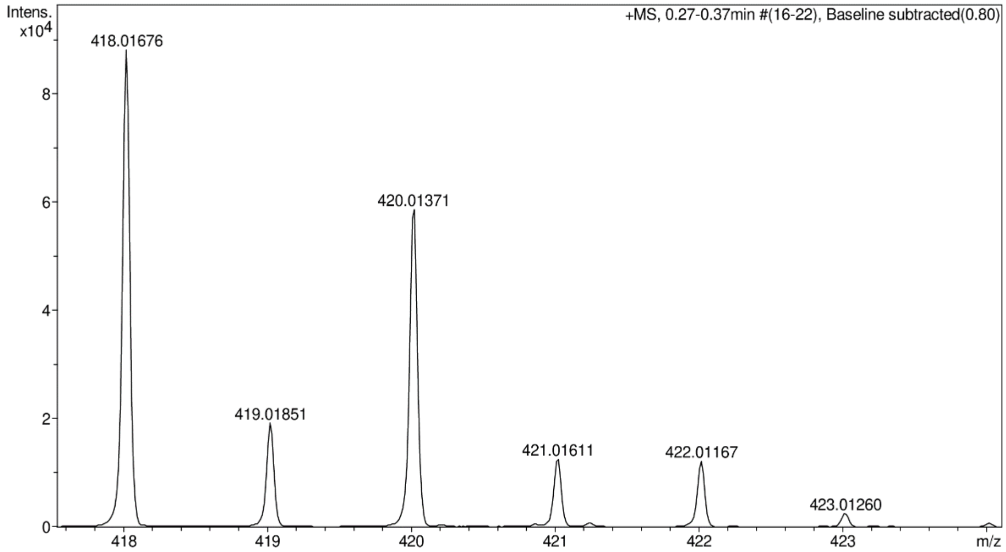


Bruker Compass DataAnalysis 4.0 printed: 2/13/2023 3:30:39 PM Page 1 of 1

**Figure S238** ESI-HRMS spectrum of **6e''**

## Generic Display Report

| | | | |
|---|---|---|---|
| **Analysis Info** | | Acquisition Date | 2/13/2023 12:50:15 PM |
| Analysis Name | D:\Data\Data Service\230213\light-target-3Br_RA8_01_6755.d | | |
| Method | nv_pos_5min_profile_190214.m | Operator | CU. |
| Sample Name | light-target-3Br | Instrument | micrOTOF-Q |
| Comment | | | |

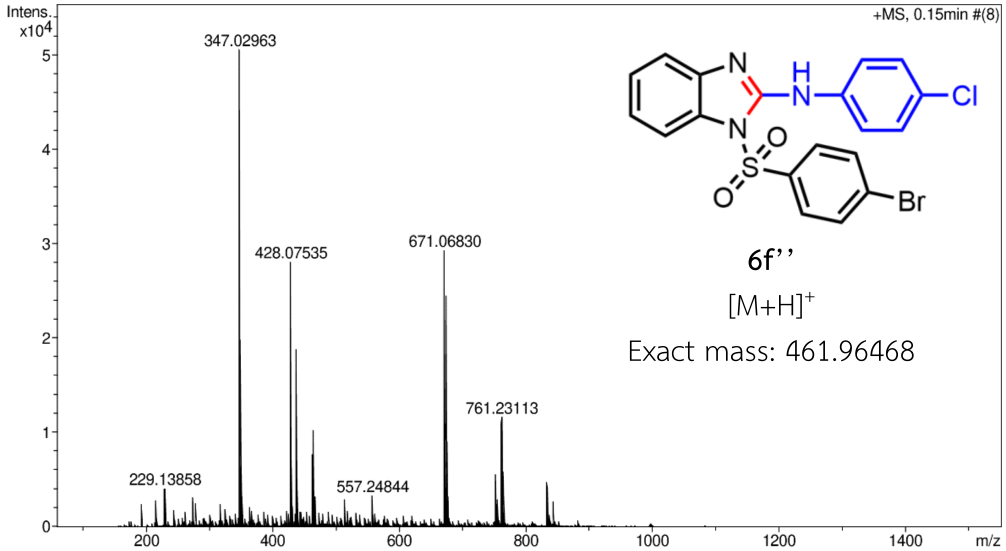


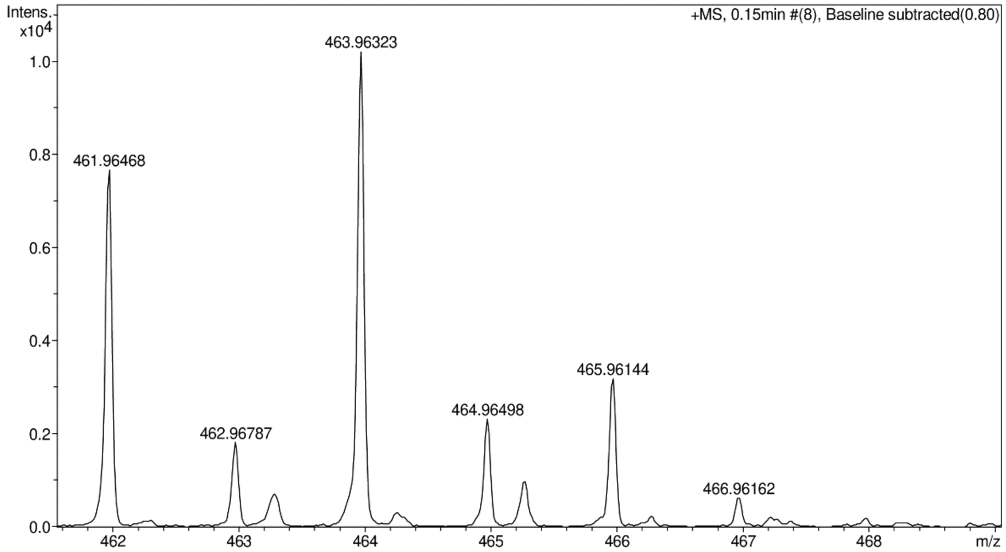


Bruker Compass DataAnalysis 4.0 printed: 2/13/2023 4:08:59 PM Page 1 of 1

**Figure S239** ESI-HRMS spectrum of **6f''**

Generic Display Report

| Analysis Info | | Acquisition Date | 1/6/2023 4:01:46 PM |
|---|---|---|---|
| Analysis Name | D:\Data\Data Service\230106\NHSO2Ph+4-Cl-NCS_RA4_01_6673.d | | |
| Method | nv_pos_5min_profile_190214.m | Operator | CU. |
| Sample Name | NHSO2Ph+4-Cl-NCS | Instrument | micrOTOF-Q |
| Comment | | | |

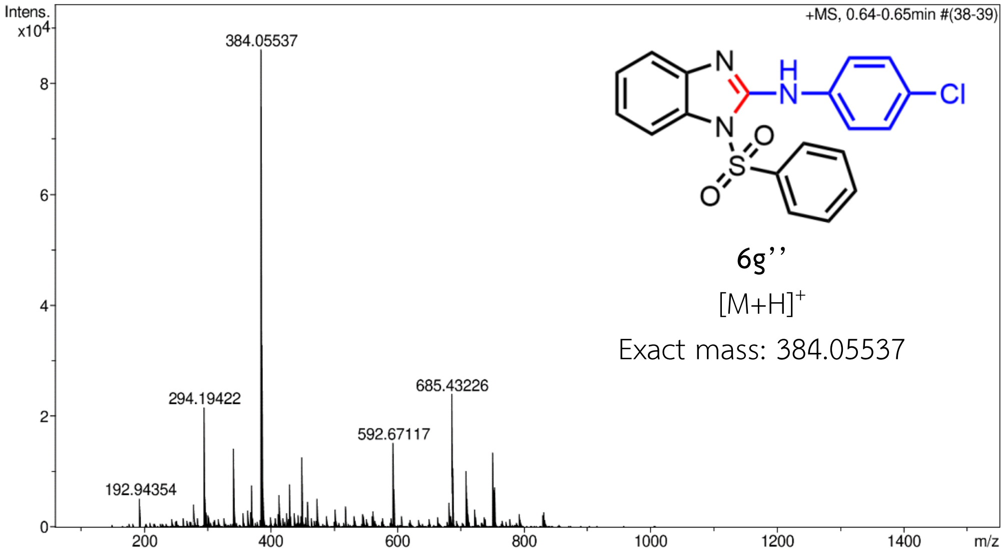


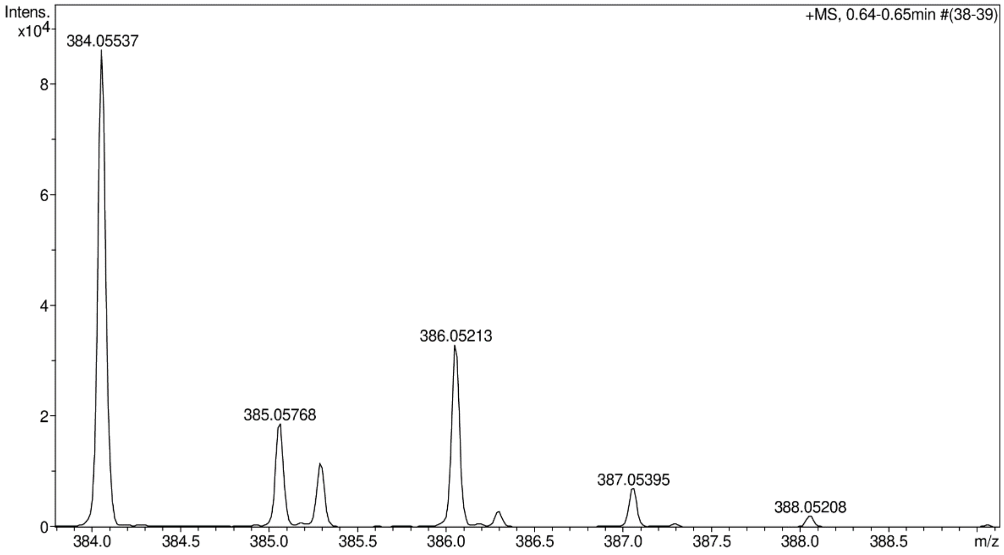


Bruker Compass DataAnalysis 4.0 printed: 1/6/2023 4:38:24 PM Page 1 of 1

**Figure S240** ESI-HRMS spectrum of **6g''**

## Generic Display Report

**Analysis Info**

| | | | |
|---|---|---|---|
| Analysis Name | D:\Data\Data Service\230213\light-target-propyl_RA5_01_6751.d | Acquisition Date | 2/13/2023 12:24:34 PM |
| Method | nv_pos_5min_profile_190214.m | Operator | CU. |
| Sample Name | light-target-propyl | Instrument | micrOTOF-Q |
| Comment | | | |

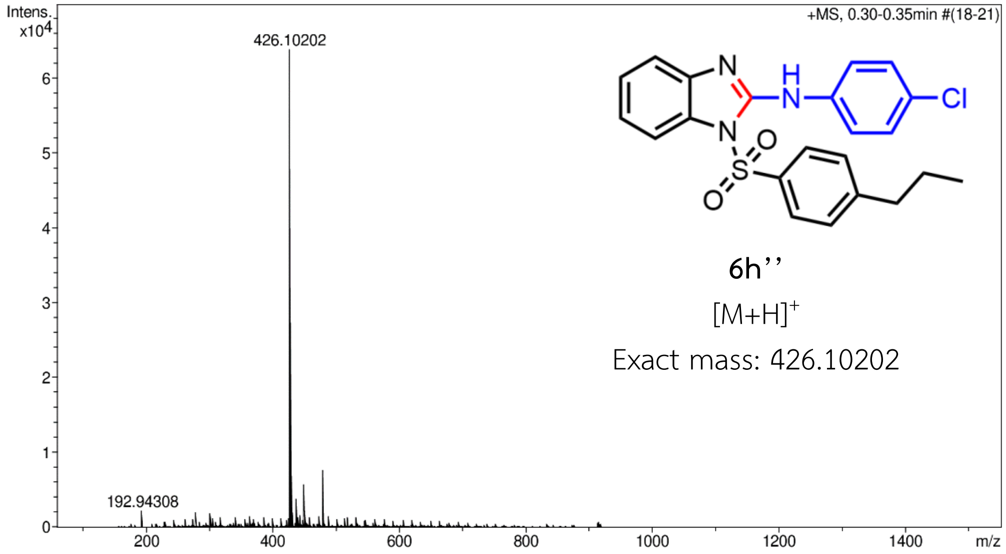


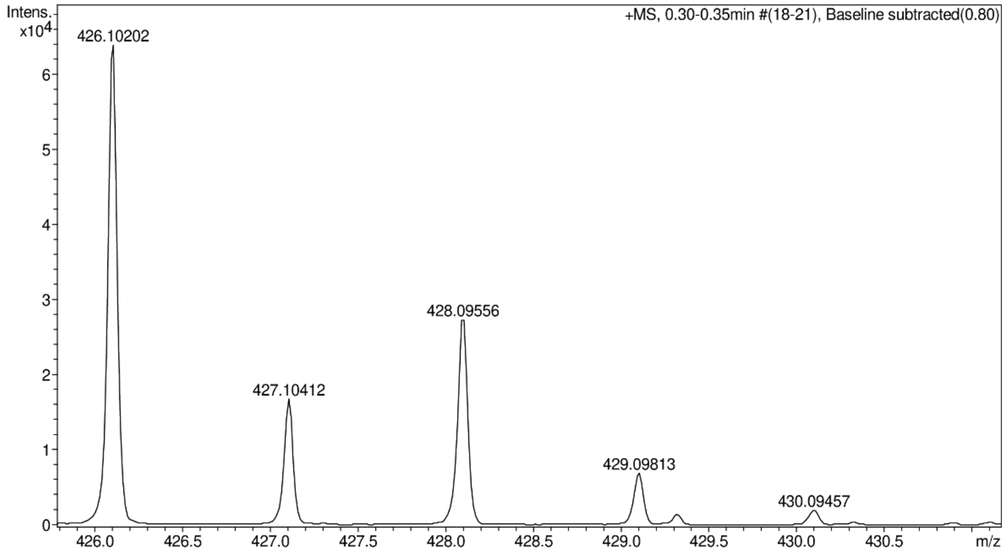


Bruker Compass DataAnalysis 4.0 printed: 2/13/2023 2:54:17 PM Page 1 of 1

**Figure S241** ESI-HRMS spectrum of **6h''**

## Generic Display Report

| | | | |
|---|---|---|---|
| **Analysis Info** | | Acquisition Date | 9/25/2023 10:38:27 AM |
| Analysis Name | D:\Data\Data Service\230925\NH-t-butyl+4-Cl-NCS_RA3_01_7730.d | | |
| Method | nv_pos_5min_profile_190214.m | Operator | CU. |
| Sample Name | NH-t-butyl+4-Cl-NCS | Instrument | micrOTOF-Q |
| Comment | | | |

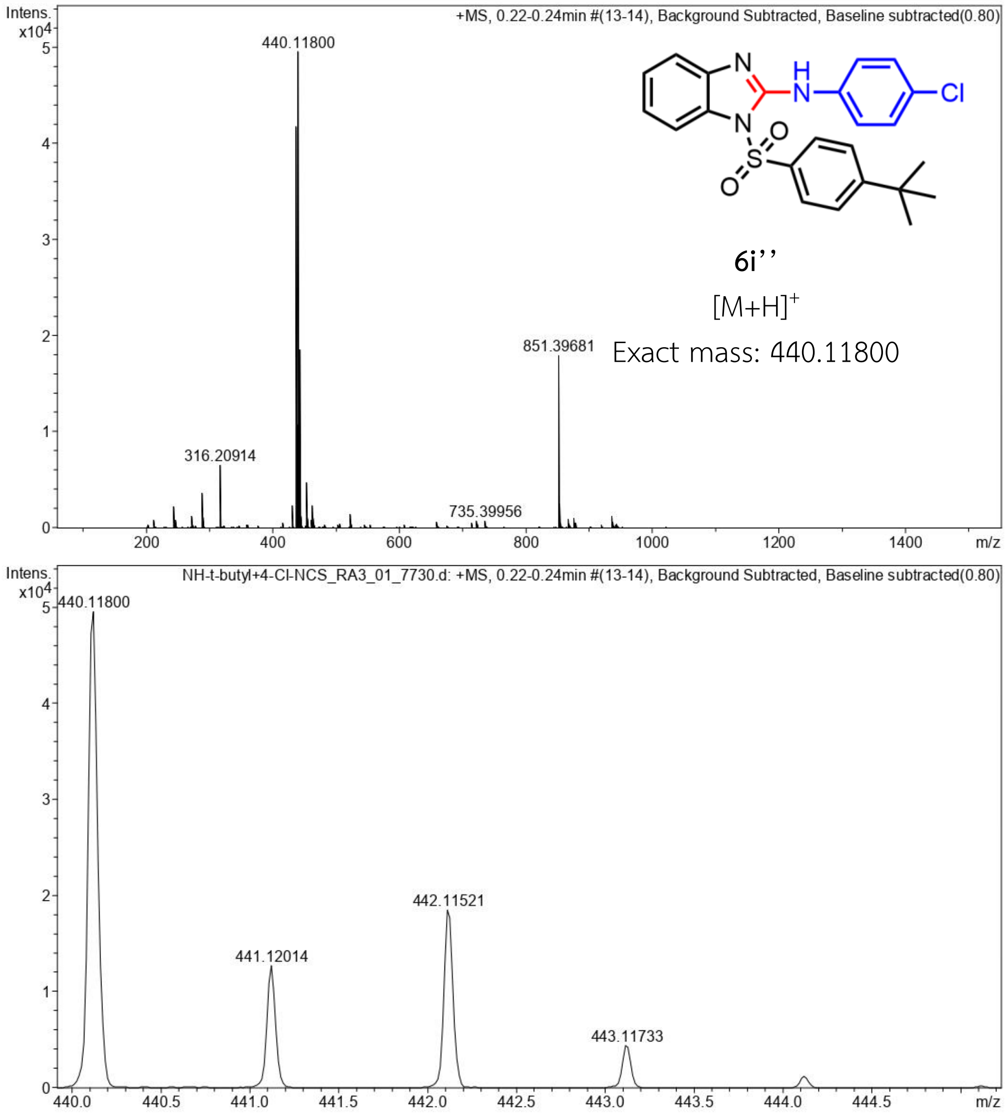


Bruker Compass DataAnalysis 4.0 printed: 9/25/2023 4:13:52 PM Page 1 of 1

**Figure S242** ESI-HRMS spectrum of **6i''**

# Generic Display Report

**Analysis Info**

| | | | |
|---|---|---|---|
| Analysis Name | D:\Data\Data Service\230925\NHSO2Ph-OMe+4-Cl-NCS_RA8_01_7736.d | Acquisition Date | 9/25/2023 11:17:03 AM |
| Method | nv_pos_5min_profile_190214.m | Operator | CU. |
| Sample Name | NHSO2Ph-OMe+4-Cl-NCS | Instrument | micrOTOF-Q |
| Comment | | | |

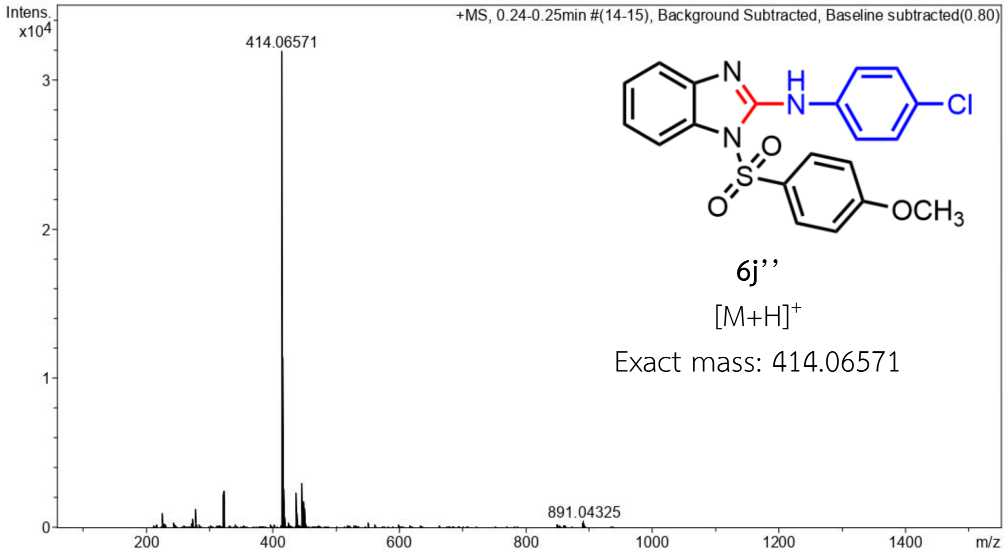


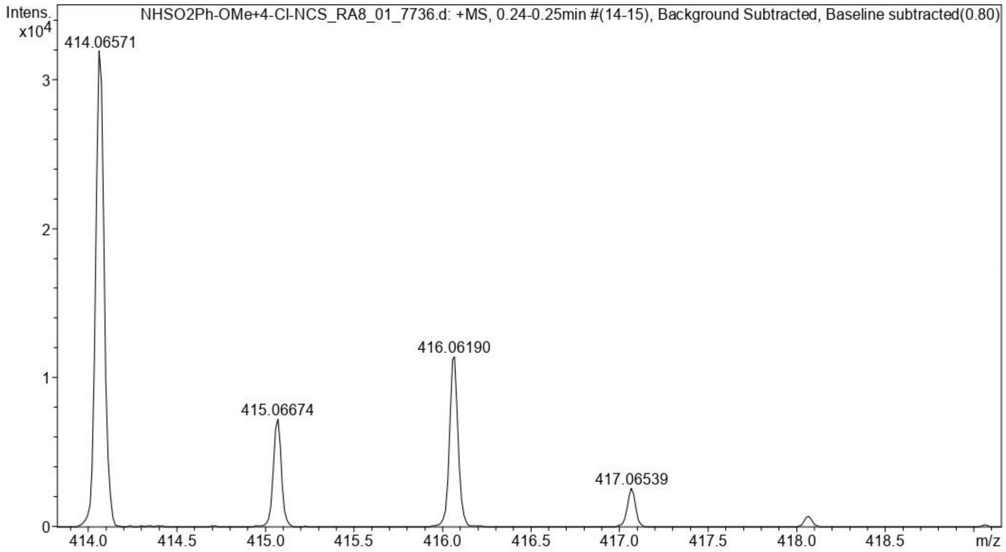


Bruker Compass DataAnalysis 4.0 printed: 9/25/2023 5:46:52 PM Page 1 of 1

**Figure S243** ESI-HRMS spectrum of **6j''**

# Generic Display Report

| | | | |
|---|---|---|---|
| **Analysis Info** | | Acquisition Date | 9/18/2023 3:05:09 PM |
| Analysis Name | D:\Data\Data Service\230918\NHSO2Ph-CF3+4-Cl-NCS_RA5_01_7720.d | | |
| Method | nv_pos_5min_profile_190214.m | Operator | CU. |
| Sample Name | NHSO2Ph-CF3+4-Cl-NCS | Instrument | micrOTOF-Q |
| Comment | | | |

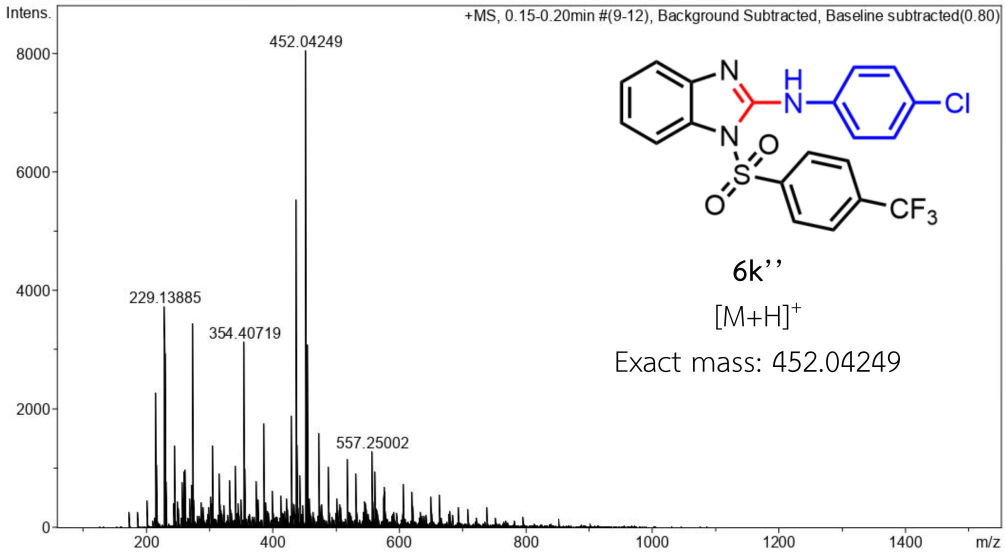


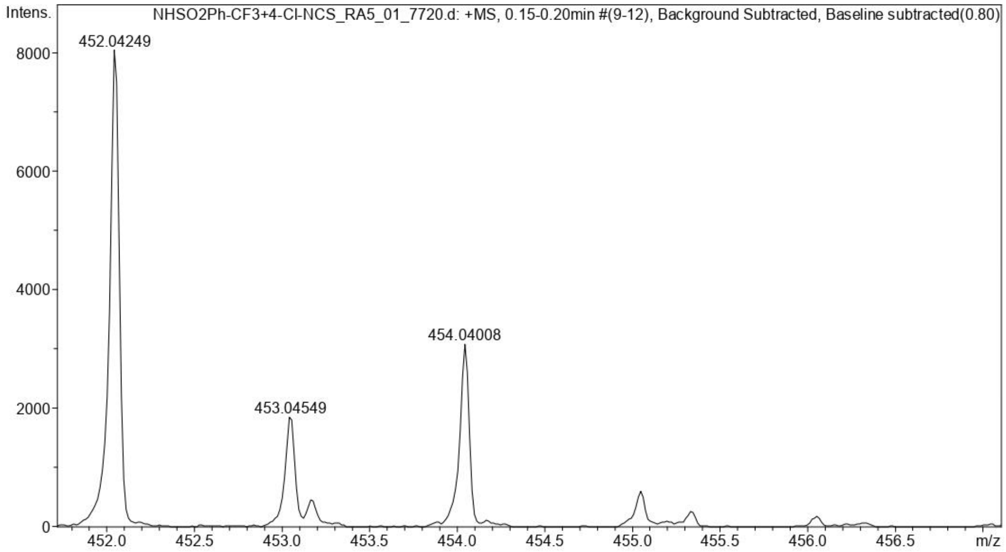


| | | |
|---|---|---|
| Bruker Compass DataAnalysis 4.0 | printed: 9/18/2023 4:27:06 PM | Page 1 of 1 |

**Figure S244** ESI-HRMS spectrum of **6k''**

## Generic Display Report

**Analysis Info**

| | | | |
|---|---|---|---|
| Analysis Name | D:\Data\Data Service\240304\NH+4NO2+4-Cl-NCS_RA8_01_9050.d | Acquisition Date | 3/4/2024 4:35:58 PM |
| Method | nv_pos_5min_profile_190214.m | Operator | CU. |
| Sample Name | NH+4NO2+4-Cl-NCS | Instrument | micrOTOF-Q |
| Comment | | | |

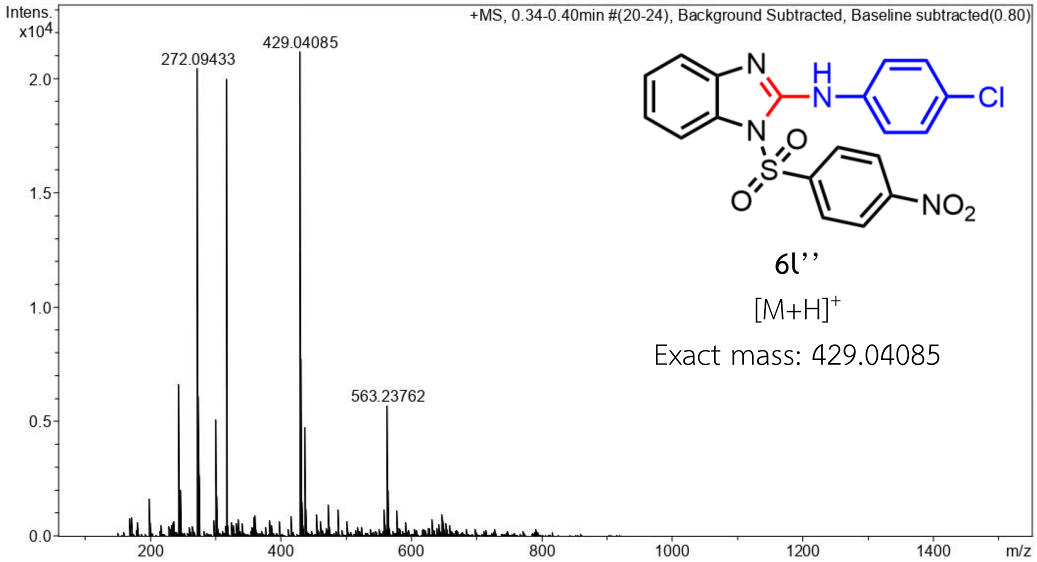


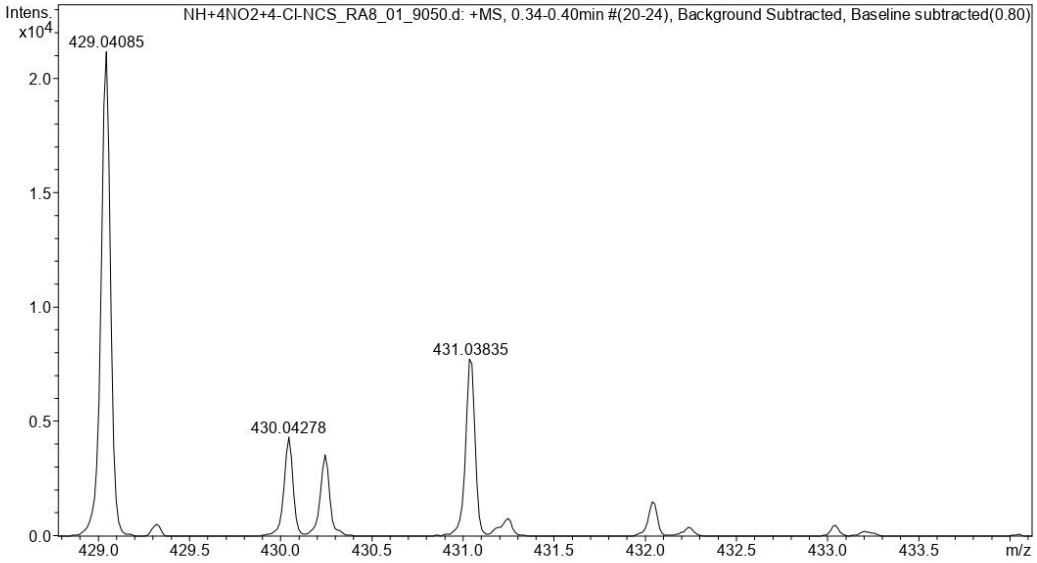


Bruker Compass DataAnalysis 4.0 printed: 3/4/2024 6:02:57 PM Page 1 of 1

**Figure S245** ESI-HRMS spectrum of **6l''**

## Generic Display Report

**Analysis Info**

| | | | |
|---|---|---|---|
| Analysis Name | D:\Data\Data Service\230918\NH-2-napthyl+4-Cl-NCS_RA2_01_7715.d | Acquisition Date | 9/18/2023 2:33:30 PM |
| Method | nv_pos_5min_profile_190214.m | Operator | CU. |
| Sample Name | NH-2-napthyl+4-Cl-NCS | Instrument | micrOTOF-Q |
| Comment | | | |

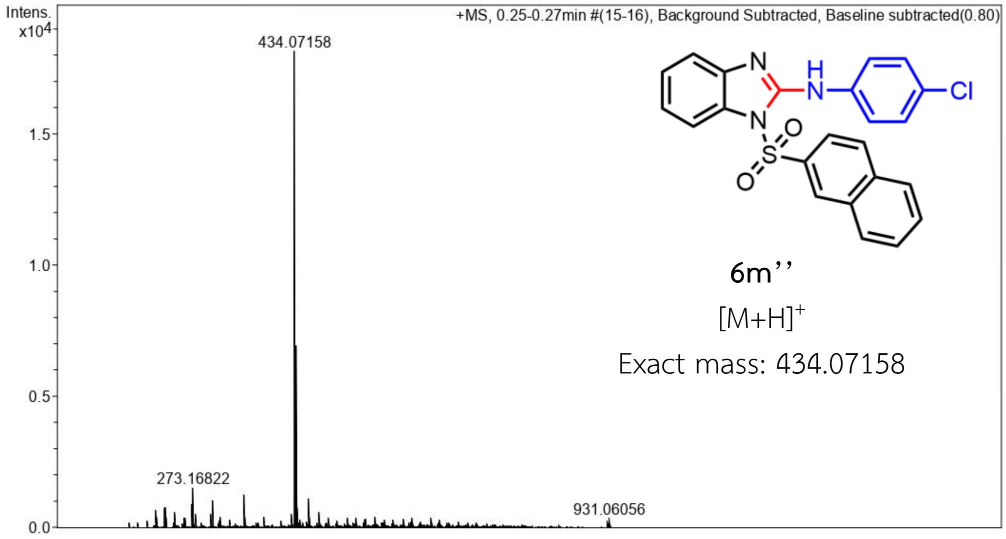


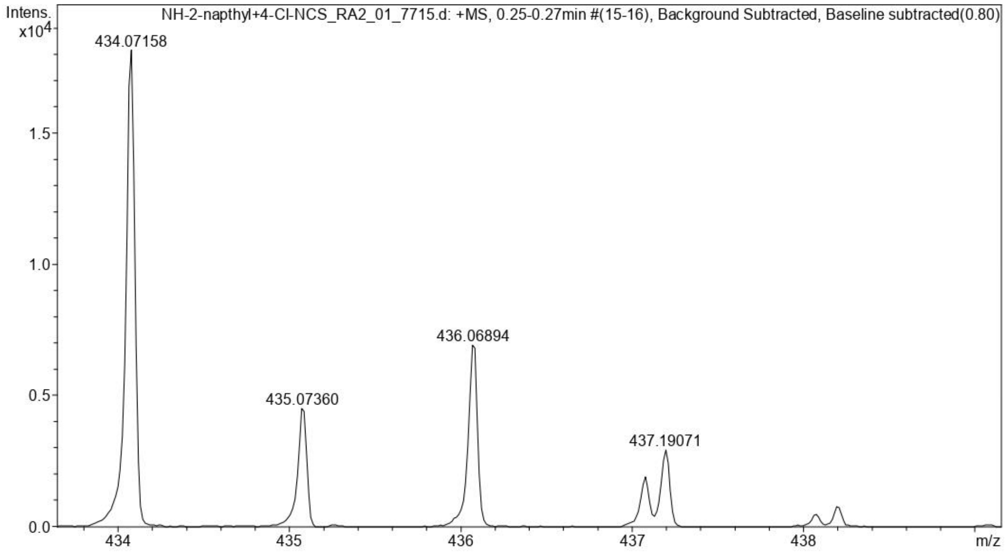


Bruker Compass DataAnalysis 4.0 printed: 9/18/2023 4:15:33 PM Page 1 of 1

**Figure S246** ESI-HRMS spectrum of **6m''**

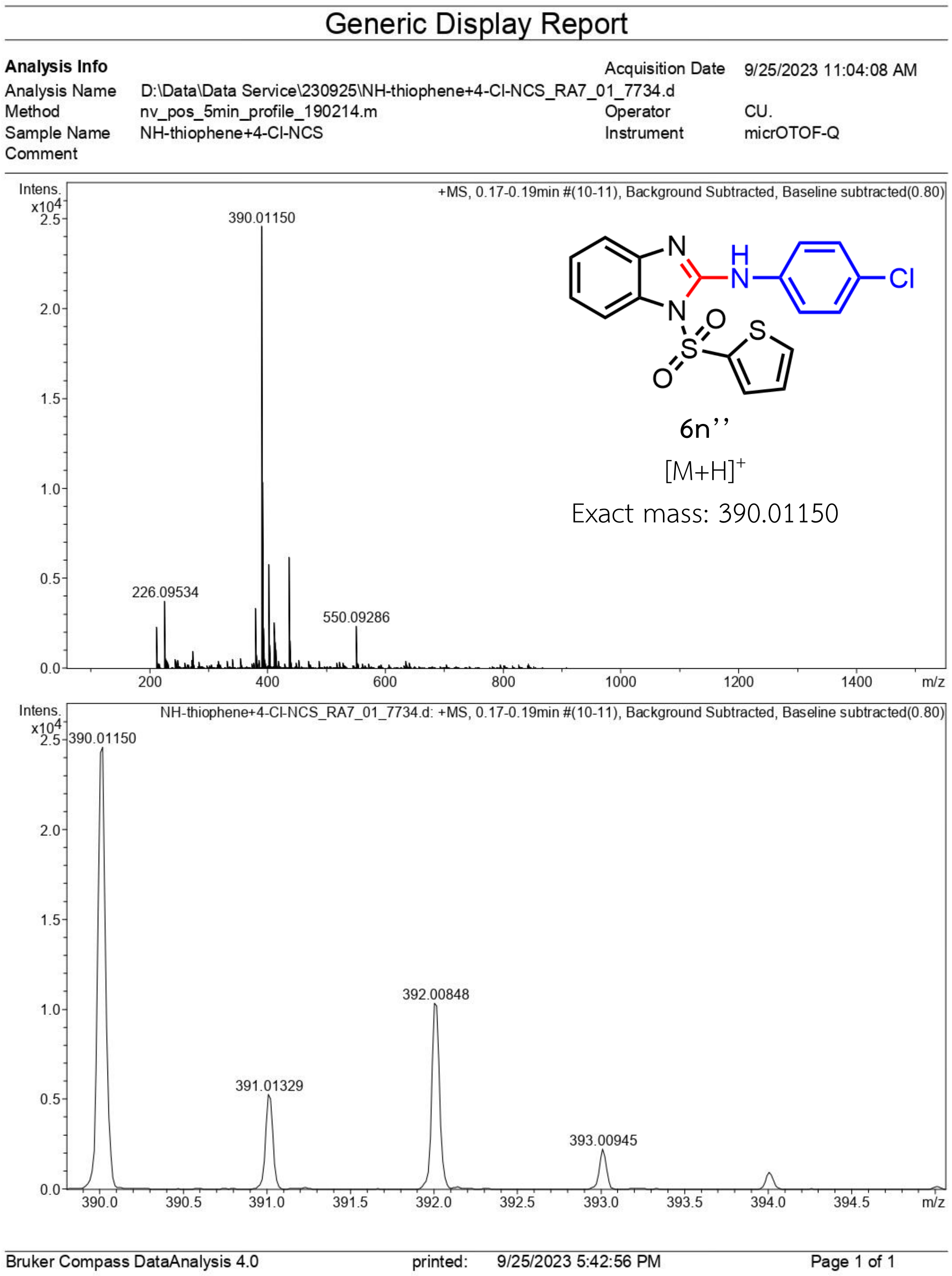


**Figure S247** ESI-HRMS spectrum of **6n''**

## Generic Display Report

| | | | |
|---|---|---|---|
| **Analysis Info** | | Acquisition Date | 1/6/2023 5:53:16 PM |
| Analysis Name | D:\Data\Data Service\230106\NHMs+4-Cl-NCS_RB1_01_6681.d | | |
| Method | nv_pos_5min_profile_190214.m | Operator | CU. |
| Sample Name | NHMs+4-Cl-NCS | Instrument | micrOTOF-Q |
| Comment | | | |

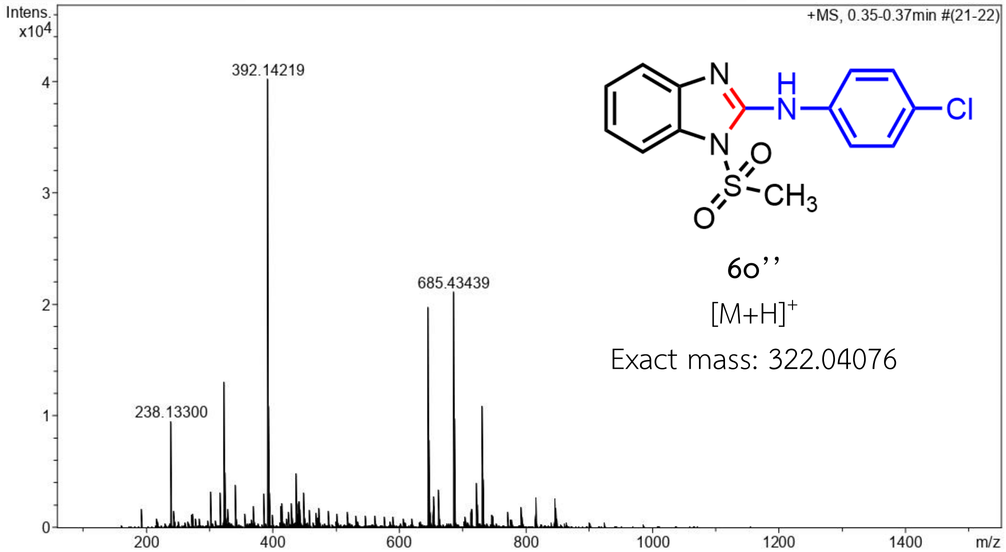


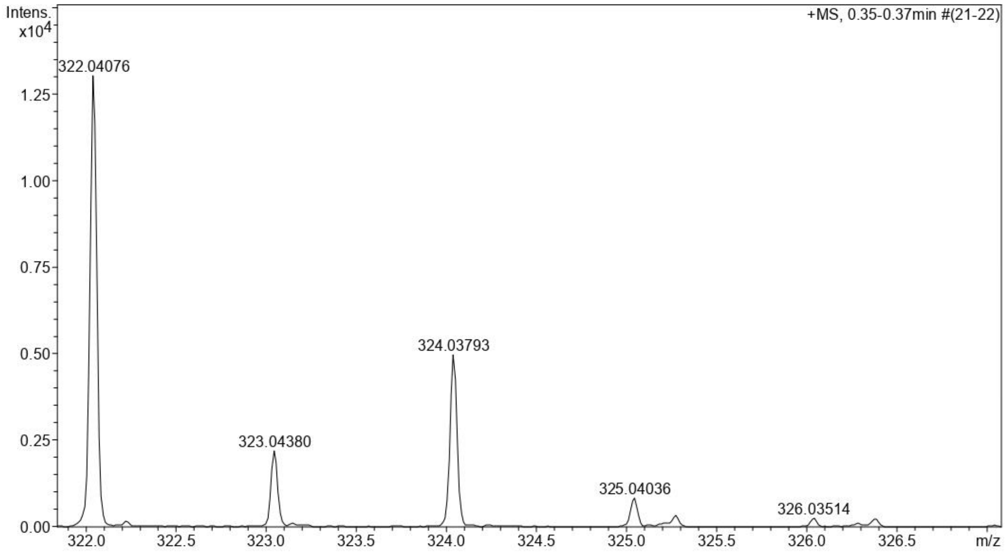


Bruker Compass DataAnalysis 4.0 printed: 1/6/2023 6:12:12 PM Page 1 of 1

**Figure S248** ESI-HRMS spectrum of **6o''**

# Generic Display Report

**Analysis Info**

| | | | |
|---|---|---|---|
| Analysis Name | D:\Data\Data Service\221219\NHBoc+4-Cl-NCS_RD8_01_6652.d | Acquisition Date | 12/19/2022 3:47:33 PM |
| Method | nv_pos_5min_profile_190214.m | Operator | CU. |
| Sample Name | NHTs+4-Cl-NCS | Instrument | micrOTOF-Q |
| Comment | | | |

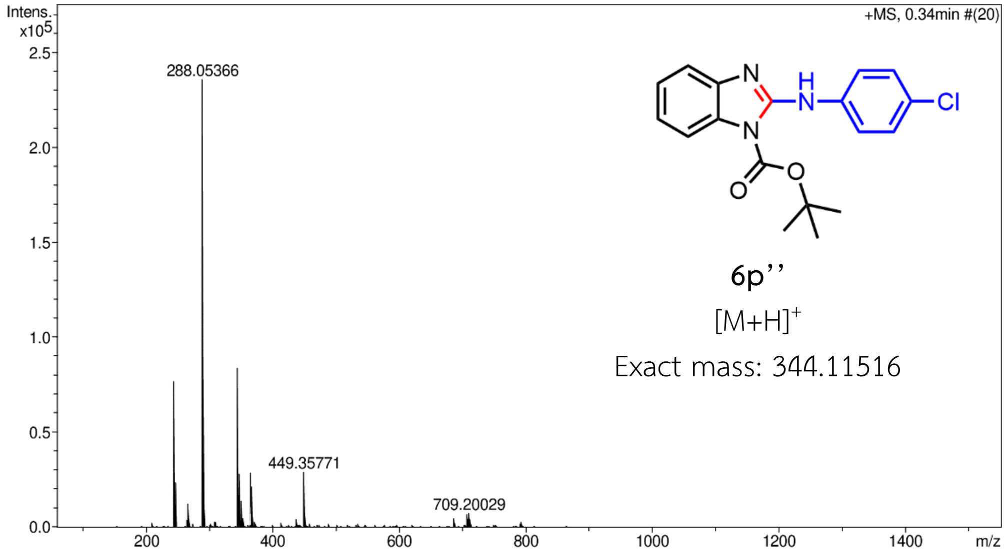


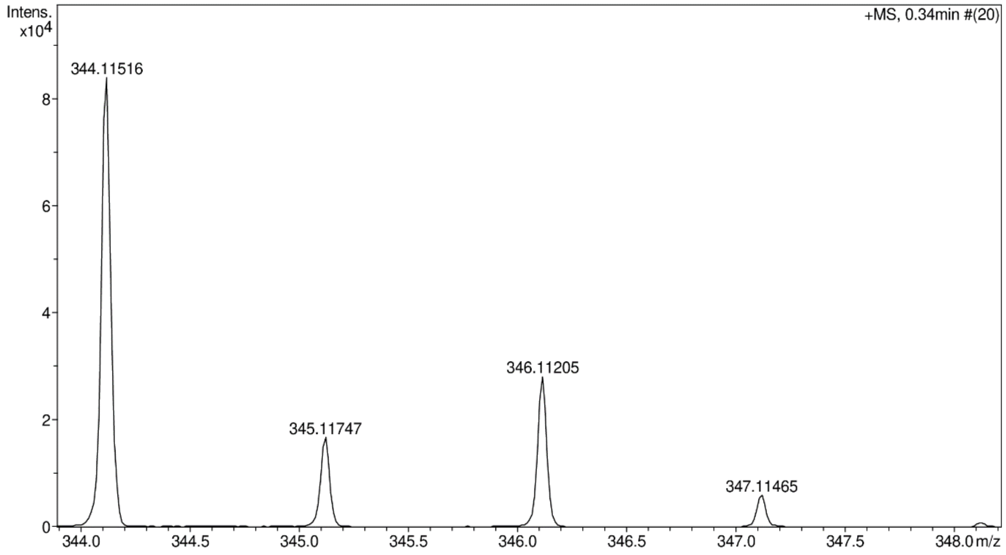


Bruker Compass DataAnalysis 4.0 printed: 12/19/2022 8:23:37 PM Page 1 of 1

**Figure S249** ESI-HRMS spectrum of **6p''**

# Generic Display Report

**Analysis Info**

| | | | |
|---|---|---|---|
| Analysis Name | D:\Data\Data Service\230124\NHAllyl+4-Cl-NCS_RB5_01_6714.d | Acquisition Date | 1/24/2023 5:14:32 PM |
| Method | nv_pos_5min_profile_190214.m | Operator | CU. |
| Sample Name | NHAllyl+4-Cl-NCS | Instrument | micrOTOF-Q |
| Comment | | | |

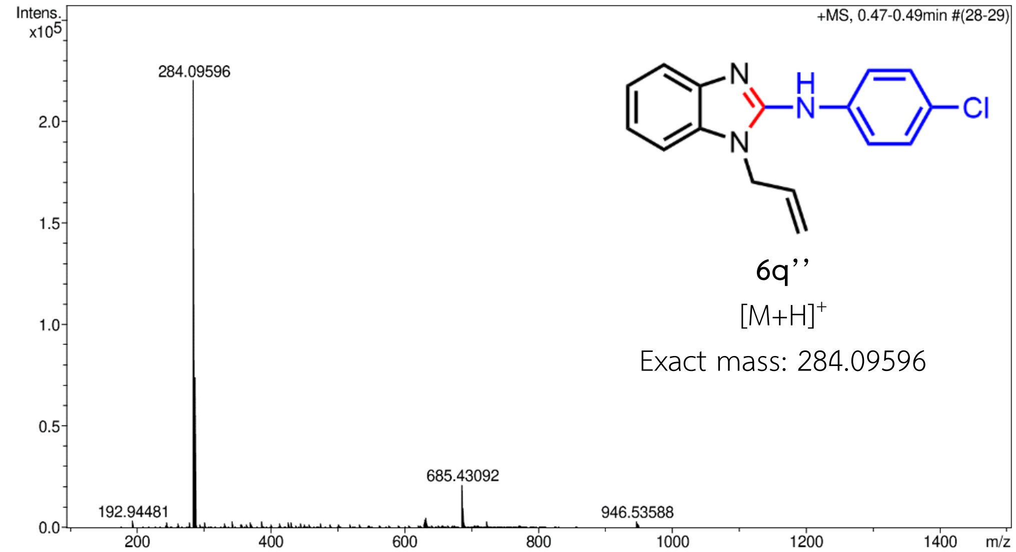


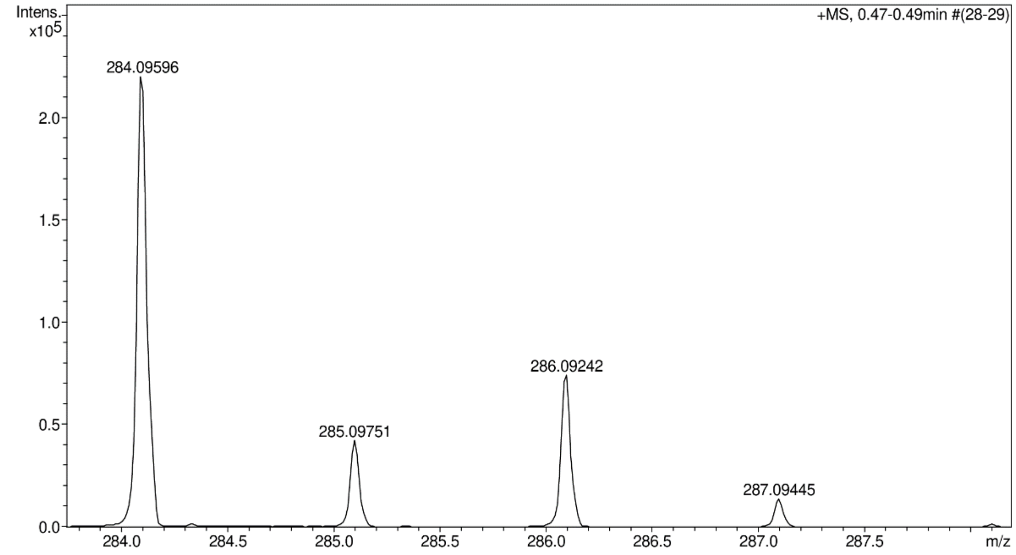


Bruker Compass DataAnalysis 4.0 printed: 1/24/2023 6:00:44 PM Page 1 of 1

**Figure S250** ESI-HRMS spectrum of **6q''**

# Generic Display Report

**Analysis Info**

| | | | |
|---|---|---|---|
| Analysis Name | D:\Data\Data Service\240304\NHBn+4-Cl-NCS_RA6_01_9037.d | Acquisition Date | 3/4/2024 2:20:51 PM |
| Method | nv_pos_5min_profile_190214.m | Operator | CU. |
| Sample Name | NHBn+4-Cl-NCS | Instrument | micrOTOF-Q |
| Comment | | | |

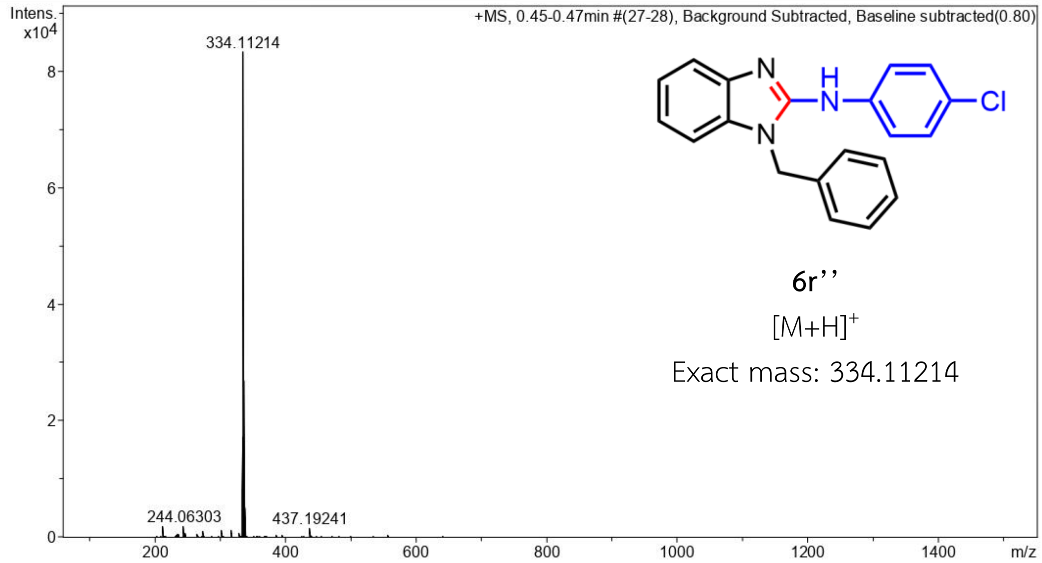


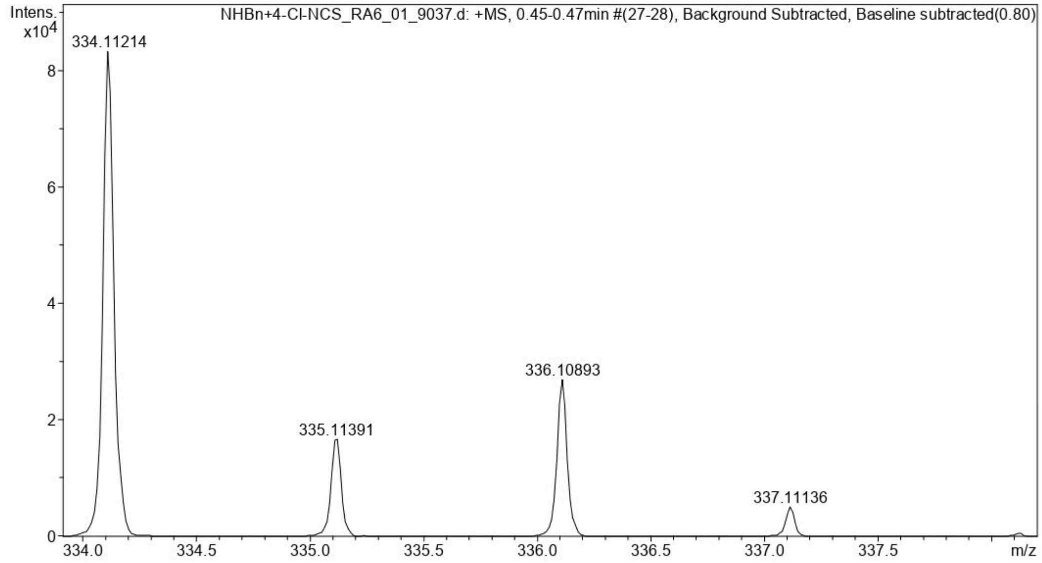


Bruker Compass DataAnalysis 4.0 printed: 3/4/2024 2:32:08 PM Page 1 of 1

**Figure S251** ESI-HRMS spectrum of **6r''**

# Generic Display Report

**Analysis Info**

| | | | |
|---|---|---|---|
| Analysis Name | D:\Data\Data Service\240304\NHBrBn+4-Cl-NCS_RA7_01_9038.d | Acquisition Date | 3/4/2024 2:27:18 PM |
| Method | nv_pos_5min_profile_190214.m | Operator | CU. |
| Sample Name | NHBrBn+4-Cl-NCS | Instrument | micrOTOF-Q |
| Comment | | | |

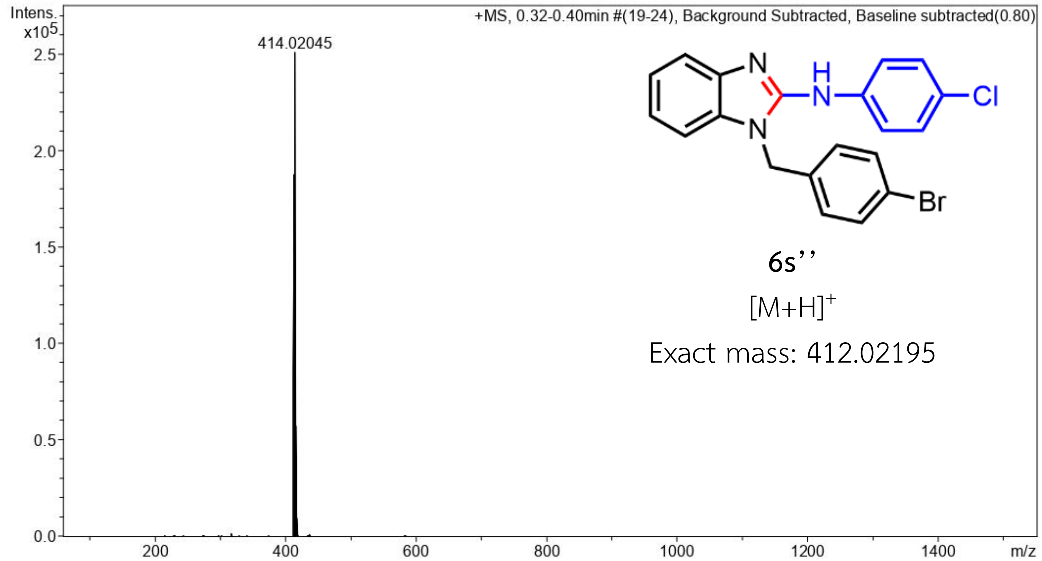


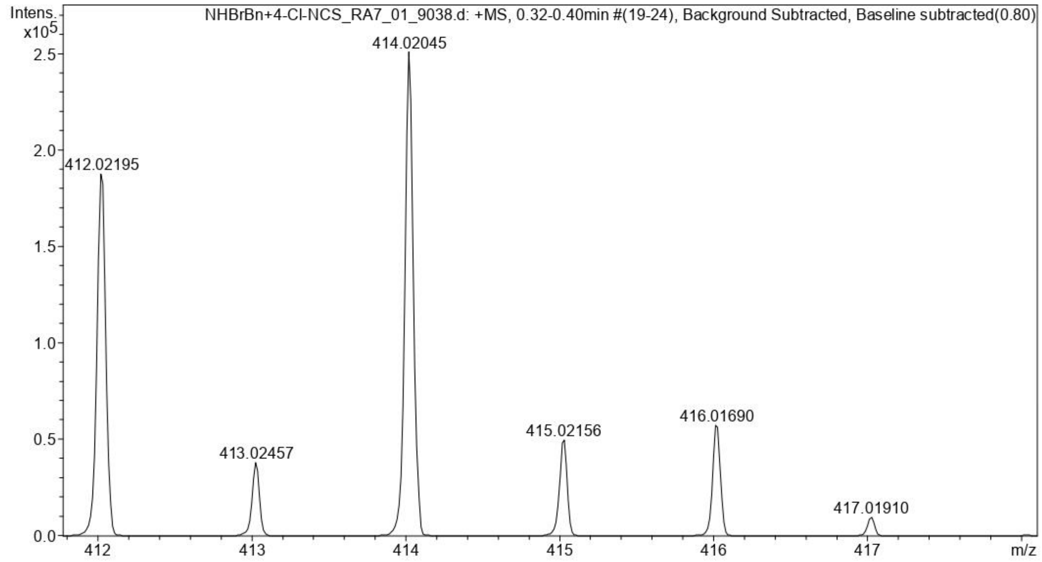


Bruker Compass DataAnalysis 4.0 printed: 3/4/2024 2:35:47 PM Page 1 of 1

**Figure S252** ESI-HRMS spectrum of **6s''**

## References


1. Fu, S.; Jaing, H.; Deng, Y.; Zeng, W., Palladium-Catalyzed Intramolecular Sulfonamidation/Oxidation of Imines: Access to Multifunctional Benzimidazoles. *Adv. Synth. Catal.* **2011**, *353*, 2795-2804.
2. Esaki, H.; Hattori, T.; Tsubone, A.; Mibayashi, S.; Sakata, T.; Sawama, Y.; Monguchi, Y.; Yasuda, H.; Nosaka, K.; Sajiki, H., Chemoselective Hyrdrogenation Catalyzed by Pd on Spherical Carbon. *ChemCatChem* **2013**, *5*, 3629-3625.
3. Hossain, M. S.; Le, C. Q.; Joseph, E.; Nguyen, T. Q.; Johnson-Winters, K.; Foss, Jr. F. W., Convenient synthesis of deazaflavin cofactor FO and its activity in $F_{420}$-dependent NADP reductase. *Org. Biomol. Chem.* **2015**, *13*, 5082-5085.
4. Li, Z. -Y.; Ma, H. -Z.; Han, C.; Xi, H. -T.; Meng, Q.; Chen, X.; Sun, X. -Q., Synthesis of Isothiocyanates by Reaction of Amines with Phenyl Chlorothiono-formate via One-Pot or Two-Step Process. *Synthesis*, **2013**, *45*, 1667-1674.
5. Techapanalai, S.; Annuur, R. M.; Sukwattanasinitt, M.; Wacharasindhu, S., One-Pot Synthesis of Isothiocyanates from Amines Mediated by Carbon Tetrabromide. *ChemistrySelect* **2023**, *8*, e202302045.
6. Sun, N.; Li, B.; Shao, J.; Mo, W.; Hu, B.; Shen, Z.; Hu, X., A general and facile one-pot process of isothiocyanates from amines under aqueous condition. *Beilstein J. Org. Chem.* **2012**, *8*, 61-70.
7. Frazier, K. M.; Swager, T. M., Robust Cyclohexanone Selective Chemiresistors Based on Single-Walled Carbon Nanotubes. *Anal. Chem.* **2013**, *85*, 7154.7158.
8. Knaggs, S.; Malkin, H.; Osborn, H. M. I.; Williams, N. A. O.; Yaqoob, P., New prodrugs derived from 6-aminodopamine and 4-aminophenol as candidates for melanocyte-directed enzyme prodrug therapy (MDEPT). *Org. Biomol. Chem.* **2005**, *3*, 4002-4010.
9. Minegishi, H.; Futamura, Y.; Fukashiro, S.; Muroi, M.; Kawatani, M.; Osada, H.; Nakamura, H., Methyl 3-((6-Methoxy-1,4-dihydroindeno[1,2-*c*]pyrazol-3-yl)amino)benzoate (GN39482) as a Tubulin Polymerization Inhibitor Identified by MorphoBase and ChemProteoBase Profiling Methods. *J. Med. Chem.* **2015**, *58*, 4230-4241.

10. Fu, Z.; Yuan, W.; Chen, N.; Yang, Z.; Xu, J., $Na_2S_2O_8$-mediated efficient synthesis of isothiocyanates from primary amines in water. *Green Chem.* **2018**, *20*, 4484-4491.

11. Nguyen, K. T.; Huynh, T. N. T.; Ratanathawornkiti, K.; Juthathan, M.; Thamyongkit, P.; Sukwattanasinitt, M.; Wacharasindhu, S., NaI-mediated electrochemical cyclization-desulfurization for the synthesis of *N*-substituted 2-aminobenzimidazoles. *J. Org. Chem.* **2024**, *89*, 1591-1608.

12. Wan, Z. -K.; Ousman, E. F.; Papaioannou, N.; Saiah, E., Phosphonium-mediated cyclization of *N*-(2-aminophenyl)thioureas: efficient synthesis of 2-aminobenzimidazoles. *Tetrahedron Lett.* **2011**, *52*, 4149-4152.
